# Loss of neurodevelopmental-associated miR-592 impairs neurogenesis and causes social interaction deficits

**DOI:** 10.1038/s41419-022-04721-z

**Published:** 2022-04-01

**Authors:** Yu Fu, Yang Zhou, Yuan-Lin Zhang, Bo Zhao, Xing-Liao Zhang, Wan-Ting Zhang, Yi-Jun Lu, Aiping Lu, Jun Zhang, Jing Zhang

**Affiliations:** 1 Research Centre for Translational Medicine at East Hospital, School of Life Science and Technology, Tongji University, 200010 Shanghai, China; 2 Key Laboratory of Spine and Spinal Cord Injury Repair and Regeneration of Ministry of Education, Orthopaedic Department of Tongji Hospital, School of Medicine, Tongji University, 200065 Shanghai, China; 3 Research Centre for Translational Medicine at East Hospital, School of Medicine, Tongji University, 200010 Shanghai, China; 4 Shanghai Institute of Stem Cell Research and Clinical Translation, 200092 Shanghai, China

**Keywords:** Developmental neurogenesis, Autism spectrum disorders

## Abstract

microRNA-592 (miR-592) has been linked to neurogenesis, but the influence of miR-592 knockout in vivo remains unknown. Here, we report that miR-592 knockout represses IPC-to-mature neuron transition, impairs motor coordination and reduces social interaction. Combining the RNA-seq and tandem mass tagging-based quantitative proteomics analysis (TMT protein quantification) and luciferase reporter assays, we identified MeCP2 as the direct targetgene of miR-592 in the mouse cortex. In Tg*(MECP2)* mice, lipofection of miR-592 efficiently reduced *MECP2* expression in the brains of Tg*(MECP2)* mice at E14.5. Furthermore, treatment with miR-592 partially ameliorated the autism-like phenotypes observed in adult Tg(*MECP2*) mice. The findings demonstrate that miR-592 might play a novel role in treating the neurodevelopmental-associated disorder.

## Graphical abstract

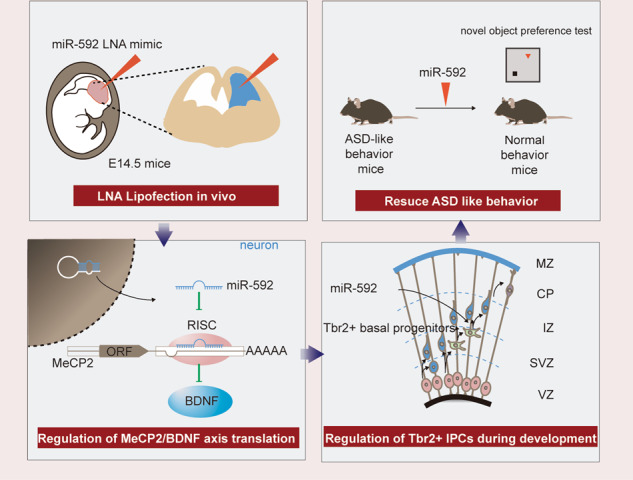

## Introduction

Accumulating evidence has indicated that some miRNAs are directly related to neurodevelopment, and loss of miRNAs function may lead to neurological disorders [1, 2]. miRNAs, 19- to 25-nucleotide-long RNAs, can regulate gene expression at the post-transcriptional level and result in translational repression or degradation [3]. miR-592 is in the untranslated exon region of glutamate metabotropic receptor 8 (Grm8), which is associated with ASD [4, 5]. In a large-scale CNV/miRNA genes association autism research data, dysregulation of hsa-miR-592 expression has been found in ASD patients [6]. We previously identified miR-592 as neural-enriched miRNA which could induce astrogliogenesis differentiation arrest or/and enhance neurogenesis in vitro [7]. By microarray analysis, loss of miR-592 in embryonic stem cells (ESCs) influenced ESC pluripotency and neurotrophin signalling pathway [8]. Moreover, the abnormal miR-592 expression has been found in glioma and Alzheimer’s disease [9, 10]. Together, these findings suggest that miR-592 contributes to pathogenesis in neurodevelopmental disorders. To date, whether miR-592 deficiency leads directly to neurodevelopmental disorders is unclear.

Here, we generated miR-592 knockout (miR-592^−/−^) mice to investigate the impact of miR-592 in vivo. Adult miR-592^−/−^ mice were characterized as reduced motor coordination and social interaction. Combining the bioinformatics analysis confirmed that methyl-CpG-binding protein 2 (MeCP2) was a targetgene of miR-592. MeCP2, an X-linked gene encoding the methylcytosine binding protein, is known to be the therapeutic target of ASD [11]. Overexpression of *MECP2* causes MeCP2 duplication syndrome (MDS) and loss of *MECP2* cause Rett syndrome (RTT), which is both accompanied by autism-like features [12, 13]. Studies on restoring *MECP2* in adult Tg(*MECP2*) mice have precedents. Notwithstanding, abnormal *MECP2* expression of adult mice was still prominent 4 weeks after the initiation of treatment [14].

In our study, normalized *MECP2* expression levels have been started to be observed in miR-592-treated developing mouse brains after 1 week. Behaviour experiments showed that miR-592 treatment partly ameliorated social interaction deficits. In summary, these findings highlight the novel roles of miR-592 in neurodevelopment.

## Materials and methods

### Mice

All procedures were conducted in accordance with the guidelines of the National Health and Medical Research Council of China and were approved by the animal ethics review board of Tongji University.

**Fig. 1:**
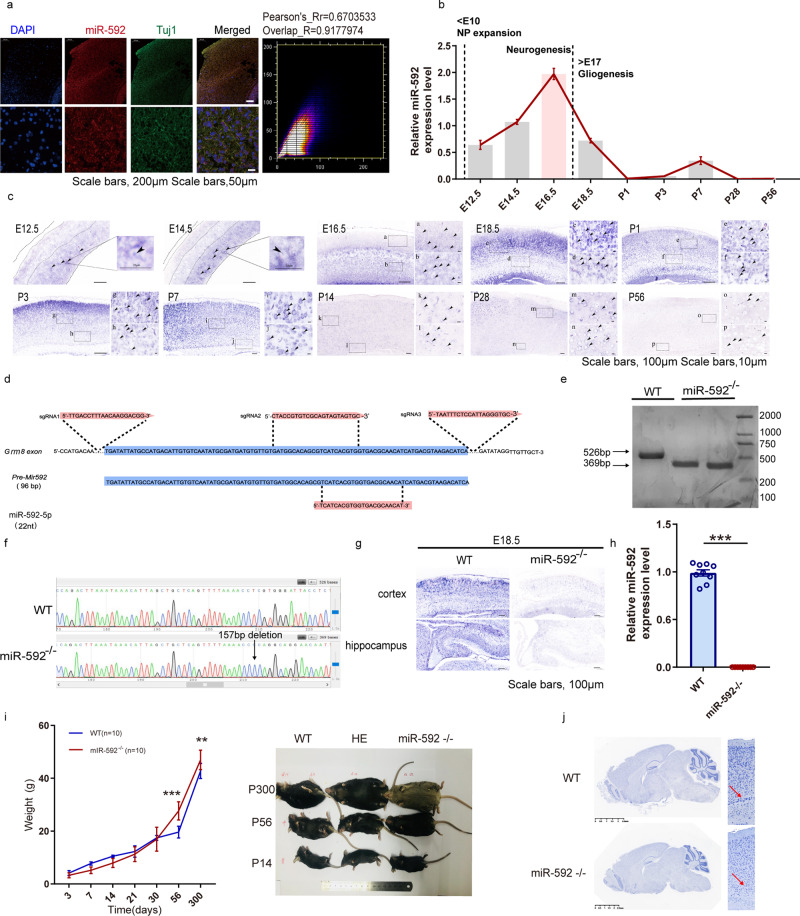
Expression pattern of miR-592 indicated an important role in brain development. **a** Distribution of miR-592 in mature neurons in cortical slices of 8-week-old WT mice as revealed by co-immunofluorescence experiments with an antibody against Tuj1 (green) and FISH of miR-592 (red). The amplification images are shown at the bottom of each group. Scale bars = 200 μm (upper), 50 μm (lower). Experiments were carried out using three biological replicates. Each sample was observed in three fields of view. **b** RT-PCR analysis of miR-592 expression in the developing cerebral cortex. The data are presented as the fold change compared with the expression level at E14.5. All data are presented as mean ± S.E.M. Experiments were repeated nine times (three biological and three analytical repeats). **c** Distribution of miR-592 in sagittal sections of the developing cerebral cortex at E12.5 to P56. ISH experiments were carried out using three biological replicates. Each sample was observed in three fields of view. **d** Illustration of the sgRNAs designed for miR-592^−/−^ mice. The arrows mark the sgRNA-targeting sequences. **e**, **f** Genotyping (**e**) and Sanger-sequencing analysis (**f**) of miR-592^−/−^ mice. The expected fragment sizes were as follows: WT, 526 bp; miR-592^−/−^ mice, 369 bp. **g**, **h** In situ hybridization (**g**) and RT-PCR (**h**) showing effective knockdown of miR-592. All data are presented as mean ± S.E.M. ***p < 0.001 (unpaired two-tailed *t* test). Experiments were carried out using three biological replicates. Each sample was observed in three fields of view or three analytical repeats. **i** Body weight changes over time (days). All data are presented as mean ± S.D. **p < 0.01, ***p < 0.001 (Student’s *t* test). Experiments were carried out using ten biological replicates. **j** Nissl staining showings evidence of cortical developmental malformations. Experiments were carried out using three biological replicates. Each sample was observed in three fields of view.

miR-592 knockout mice were generated using CRISPR-Cas9 targeting. Shaorong Gao Laboratory provided an established setup protocol [15]. The sgRNAs are shown in Fig. 1d. In vitro transcription of customized sgRNAs was performed using MEGAshortscript T7 kit (Thermo Fisher Scientific, Waltham, MA, USA). The source of Cas9-mRNA was GeneArt CRISPR Nuclease mRNA (Thermo Fisher Scientific, Waltham, MA, USA). The knockout efficiency was checked by PCR using DNA extracted from toe tissue from 2-week-old founder mice. The PCR primers used for miR-592^−/−^ mouse genotyping is listed in Table 1.

**Table 1:** Primers.

Gene	Sense	Antisense	Figure
miR-592 (genotyping)	5’-CCATGACAACCGACCCTTGA-3’	5’-AGCAACAACCTATATCCCACGG-3’	Fig. 1e
MeCP2 (genotyping)	5’-CGCTCCGCCCTATCTCTGA-3’	5’-ACAGATCGGATAGAAGACTC-3’	Data not shown
MeCP2 (dual-Luciferase)	5’-TGTTCTTCCTGGTGACTCTG-3’	5’-CCCTTGTCCTACTCTATGGT-3’	Fig. 5b
U6 (RT-PCR)	5’-CGCTTCGGCAGCACATATAC-3’	5’-AATTTGCGTGTCATCCTTGC-3’	Fig. 1b
miR-592 (RT-PCR)	5’-AAGGGATTCTGATGTTGGTCACAC-3’	5’-GCTGTCAACGATACGCTACGTAACG-3’	Fig. 1b
Sox2 (RT-PCR)	5’-TCTCAAACTGTGCATAATGGAGTAA-3’	5’-CCCTTTTATTTTCCGTAGTTGTATT-3’	Fig. 2d
Map2 (RT-PCR)	5’-CAATCTTCACATTACCACCTCCA-3’	5’-CTCTAAAGAACATCCGTCAC-3’	Fig. 2d
TBR1 (RT-PCR)	5’-GCTTCGTCACAGTTTCGATGG-3’	5’-CCGTTGGTAATGACCGGGTG-3’	Fig. 2d
TBR2 (RT-PCR)	5’-GCAATAAGATGTACGTTCACCCA-3’	5’-GCAGAGACTGCAACACTATCAT-3’	Fig. 2d
CTIP2 (RT-PCR)	5’-CCCGACCCTGATCTACTCAC-3’	5’-GGAGGTGGACTGCTCTTGT-3’	Fig. 2d
PAX6 (RT-PCR)	5’-TACCAGTGTCTACCAGCCAAT-3’	5’-TGCACGAGTATGAGGAGGTCT-3’	Fig. 2d
MeCP2 (RT-PCR)	5’-TTCTATTCTGGGCTTTTGATTTGT-3’	5’-CCCTTGTCCTACTCTATGGTTATCA-3’	Figs. 4g, h and 5c
Pten (RT-PCR)	5’-TGGATTCGACTTAGACTTGACCT-3’	5’-GCGGTGTCATAATGTCTCTCAG-3’	Fig. 4g
Lrrc7 (RT-PCR)	5’-CAAGCTCTACGGAAACTAAGCA-3’	5’-ACACCGTTTTTACTGATGTCGAG-3’	Fig. 4g
FOXP1 (RT-PCR)	5’-CACCTCAGGTTATCACTCCTCA-3’	5’-AGCTGCAACTGTTCCTGTTGT-3’	Fig. 4g
GAPDH (RT-PCR)	5’-GCTGTCAACGATACGCTACGTAACG-3’	5’-TGAAGGGGTCGTTGATCG-3’	Fig. 4g
Grm8 (RT-PCR)	5’-TCCCTTCCCTCTCCAACCTAACATG-3’	5’-CCACGCTCTTCCAATCCTCTTTCC-3’	Fig. S1e
BDNF (RT-PCR)	5’-TCATACTTCGGTTGCATGAAGG-3’	5’-AGACCTCTCGAACCTGCCC-3’	Fig. S4h

Tg(*MECP2*) mice were generously gifted from Dr. Zilong Qiu and BTBR T + Itpr3tf/J (BTBR) mice (cat#.002282) were purchased from the Jackson Laboratory. The PCR primers used for Tg(*MECP2*) mouse genotyping are also listed in Table 1.

### In situ hybridization (ISH)

ISH and fluorescence in situ hybridization (FISH) combined with immunostaining were performed, with reference to a previous report [16]. The brains of miR-592^−/−^ mice were dissected as soon as the animals were euthanized and fixed overnight in 4% paraformaldehyde and then embedded in paraffin. The brains were sectioned at a thickness of 4 μm. Qiagen synthesized the Mmu-miR-592-5p miRCURY LNA miRNA Detection probe (5DiGN-CATCATCGCATATTGACACAAT-3DiG_N, Qiagen, Hilden, Germany) and at hybridization 40 °C for 2 h.

For ISH, paraffin sections with the probe were incubated with anti-digoxigenin AP (1:400; Roche, Switzerland) at 37 °C for 50 min. Alkaline phosphatase activity was detected by developing the slides in BCIP/NBT. For FISH, a Cy3 tyramide signal amplification (TSA) kit (1:100; PerkinElmer, USA) allowed approximately 500-fold amplification of the original signal. The following antibody was used, anti-digoxigenin-POD (1:100, Roche, Switzerland). The sections were washed in DEPC water and mounted with DAPI or processed for immunohistochemistry. The results were checked with bright-field or fluorescence microscopy. The time course of expression was kept identical for a given experiment.

### Histology

Eight-week-old mice were used for histological analysis. Three mice were randomly selected from each group for histological examination. The hearts, livers, spleens, lungs, kidneys, testes, and brains of miR-592^−/−^ mice were dissected immediately after the animals were euthanized [17]. Tissues from at least three mice of each genotype were stained with HE. For Nissl staining, slides were incubated with 0.1% Nissl dye for 10 min.

### Immunofluorescence (IF) staining

IF staining was performed as previously reported in the literature [18]. The primary antibodies used were anti-Pax6 (1:350, Abcam, UK, ab195045), anti-pH3 (1:1000, Abcam, UK, ab267372), anti-SOX2 (1:200, Abcam, UK, ab79351), anti-TBR2 (1:100, Abcam, UK, ab183991), anti-MAP2 (1:500, Abcam, UK, ab32454), anti-Tuj1 (1:1000, Abcam, UK, ab7751), anti-GFAP (1:1000, Abcam, UK, ab7260), anti-Olig2 (1:1000, Abcam, UK, ab109186), anti-Iba1 (1:500, Abcam, UK, ab178846), anti-Syn1 (1:500, Abcam, UK, ab32532), anti-PSD95 (1:500, Abcam, UK, ab76115),anti-MeCP2 (1:500, Abcam, UK, ab50005), and anti-BDNF (1:200, Abcam, UK, ab108319). The secondary antibodies used were an Alexa-Fluor®488-conjugated goat anti-rabbit antibody (1:500, Invitrogen, Carlsbad, USA, Carlsbad, USA) and an AlexaFluor®546-conjugated goat anti-mouse antibody (1:500, Invitrogen, Carlsbad, USA). For bromodeoxyuridine/5-bromo-2′-deoxyuridine (BrdU) labelling, E14.5 pregnant mice were intraperitoneally given BrdU (Sigma, USA) at 25 mg/kg body weight. Animals were sacrificed within 30 min of injection. Cleaved caspase-3 staining was done using an anti-cleaved-caspase-3 antibody (1:100, Abcam, UK, ab32042). TUNEL staining was performed using TUNEL apoptosis detection kit.

### Behavioural studies

Mice were placed in white plastic cages with freely accessible water and food in an SPF animal house at 22 ± 2 °C with a 12 h light/dark cycle. All behavioural experiments were performed using 25–30 g male mice. All mice were age- and sex-matched and acclimated to the environment for at least 1 week. Eight mice were randomly selected from each group for behavioural examination. Behavioural experiments were performed from 9:00 to 17:00. The animals were habituated to the test room for two hours before starting the behavioural experiments. The video of the experiment was rated by two participants who were blinded to the treatment group after the behavioural evaluation.

The open-field test (OFT) was carried out as the methods in previous studies [19]. Mice were video recorded for 5 min as soon as placed individually in the middle of the squared box (42 × 42 × 42 cm). Between each experiment, the square box was cleaned with 75% alcohol and wiped dry, to remove odours. The recording and behaviour analyses were carried out using EthoVision XT version 13 (Noldus Information Technology, Wageningen, the Netherlands).

The rotarod test (RT) was conducted according to the methods in a previous study [20]. The rod initially rotated at 4 rpm, gradually increasing to a maximum of 40 rpm over a 5 min period. Two days before the experiment, mice were trained on the apparatus in two or three trials, with a 1 min break between trials. The mice were placed on the rotarod, and the latency to fall (cm) was recorded.

An elevated plus-maze (EPM) test was conducted according to the methods in a previous study [21]. The EPM had two closed arms (60×20×40 cm) and two open arms (arms not enclosed by walls). The mice were allowed to explore the maze freely for 5 min. The number of times each arm was accessed and the time spent were recorded.

For the light–dark transition (LDT) test, a cage (50×30×30 cm) was separated into two parts: a light and a dark chamber [22]. The mice were first placed in the dark chamber and allowed to freely explore between the two chambers for 5 min. The time spent in the chamber was documented.

For a self-grooming test, mice were left alone in a box (26 × 26 × 26 cm) for 10 min [19]. The total time all body parts were groomed was added up from a video record.

For the novel object preference (NOP) test [23], an opaque walled box(26 × 26 × 26 cm) was placed into a soundproof box. The test was performed in two steps, in the adaptive step, the mice were given 10 min to explore two familiar objects in the opaque walled box. Then one object was replaced with a different one, mice were given another 10 min to explore two objects automatically. The time spent in exploring the novel object was recorded.

For the three-chambered test, wire mesh cylinders were placed into a three-chambered apparatus (60 × 40 × 21 cm). In the adaptation task, the mice were given 10 min for exploring the three-chambered apparatus. In the sociability test session, an unfamiliar same-sex mouse called stranger1 had been placed in the wire mesh cylinders. The test mouse was placed in the central chamber and allowed to freely explore these three chambers for 10 min. In the social novelty preference test, a second novel mouse, stranger2, was placed in the opposite chamber, which was empty in the previous session. The test mouse could freely explore the chambers for 10 min in this session. The video was recorded for 10 min, and the time of interaction between two mice was recorded [24].

For the Morris water maze (MWM) task, a circular tank (188 cm in diameter and 50 cm in height) was filled with water to a depth of 40 cm [20]. A platform (*d* = 10 cm) was submerged 1.5 cm below the water’s surface in the northeast quadrant of the maze, and mice were placed into the tank from the far end of the maze. If a mouse failed to find the platform within 1 min, it was manually guided to the platform. The mice were trained each day for five days before the final test. The task performance measures, including average velocity, duration, and the distance travelled in the centre, were recorded for analysis.

### RNA-seq and TMT-based quantitative proteomic analysis

Total RNAs were extracted from cortical tissues of eight-week-WT mice and miR-592^−/−^ mice were extracted with a MiRNeasy Mini Kit (Qiagen, Hilden, Germany). Nine RNA samples from three individuals of each genotype (WT mice, heterozygote, miR-592^−/−^ mice) were performed by Berry Genomics Corporation, Beijing, China. Only six RNA samples (WT and miR-592^−/−^ mice) were used in the subsequent analysis. Around 44 million to 60 million paired-end reads were obtained for each sample after quality control. The RNA-Seq raw data files have been uploaded in NCBI (BioProject accession no. PRJNA801399; BioSample accession nos. SAMN25342029). For TMT-based quantitative proteomic analysis, 8-week-mouse cortical tissues were quantified BCA (Thermo Fisher Scientific, Waltham, MA USA), dissected and extracted by RIPA buffer for the whole proteins. Protein integrity was evaluated by SDS-PAGE. As RNA-seq, six protein samples from three individuals of each genotype were performed by Wayen Biotechnologies. The TMT-based quantitative proteomic analysis raw data files have been uploaded in iProX (https://www.iprox.cn/page/PSV023.html;?url=1643453114835XUyi, Program ID IPX0004057000). GO functional enrichment analysis was performed using Metascape software. Kyoto Encyclopedia of Genes and Genomes (KEGG) analysis was applied using the KEGG pathway database (https://www.genome.jp/kegg/pathway.html) and the DAVID (https://david.ncifcrf.gov/).

### MeCP2 reporter system construction

The predicted regulation of MeCP2 by miR-592 was investigated using a pmirGLO Dual-Luciferase miRNA Target Expression Vector (Promega, Madison, WI, USA). The PCR primers for the MeCP2 3’-UTR are listed in Table 1. They were used to generate the mouse MeCP2 3’-UTR sequence containing the miR-592 target site. The DNA fragments were purified in a 1.5% agarose gel using a MinElute Gel Extraction Kit (Qiagen, Hilden, Germany) and then inserted downstream to the luciferase gene at XbaI enzyme-digested vector pGL3-Control (Promega, Madison, WI, USA). 293T cells were seeded at a density of 5 × 10^5^ cells per well in 6-well plates. When the cells reached 80 to 90% confluence, the 293T cells were co-transfected with wild type MeCP2 3’-UTR (MeCP2 3’-UTR) or mutant MeCP2 3’-UTR (MT MeCP2 3’-UTR), and with miR-592 LNA mimic (Qiagen, Hilden, Germany), or miRNA mimic negative control (Qiagen, Hilden, Germany). Xfect Transfection Reagent (Clontech, Takara Bio USA) was used for transfection.

### Neural progenitor cell (NPC) and neuron culture

NPCs were isolated from the cortices of E14.5 miR-592^−/−^ mice and WT mice as described previously [7]. The brains were removed from the skulls and the cortices were minced and incubated with 0.05% trypsin at 37 °C for 25 min. For NPCs culture, cells were cultured in neurobasal medium containing 2% B27 (Thermo Fisher Scientific, Waltham, MA USA), 1% N2, 20 ng/ml EGF, 20 ng/ml FGF, and penicillin/streptomycin. The cells were fed every 2 days. The neural spheres were counted under microscopy after 7 days. For neuron culture, the transfected NPCs were switched to neuronal differentiation medium, and differentiation was assessed 4 days later. Neuronal differentiation medium contained 0.5 mM glutamine and penicillin/streptomycin. Neurons were plated in poly-D-lysine (Sigma, USA) coated 6-well plates (1 × 10^6^).

### Real-time PCR (RT-PCR)

Total RNA was extracted using TRIzol. A MiRNeasy Mini Kit (Qiagen, Hilden, Germany) was used to extract miRNA and total RNA from tissues and cells. Then, RT-PCR was conducted using TB Green® Advantage® RT-PCR Premix (Takara Bio Inc, Shiga, Japan). The results were quantified by using the 2−ΔΔCq method. U6 and GAPDH were used as housekeeping genes. The primer sequences used are also listed in Table 1.

### Protein extraction and western blot analysis

Brain tissue was lysed with RIPA buffer (Thermo Fisher Scientific, Waltham, MA, USA) with protease inhibitors and phosphatase inhibitors (Thermo Fisher Scientific, Waltham, MA, USA). The total soluble protein was quantified with a BCA Protein Assay Kit (Thermo Fisher Scientific, Waltham, MA, USA). Protein samples were loaded onto 8% or 10% SDS-PAGE gels, electrophoresis, and then transformed to PVDF membrane after fixation. The following antibodies were applied: anti-MeCP2 (1:5000, Abcam, UK, ab50005), anti-BDNF (1:1000, Abcam, UK, ab108319), anti-AKT (1:1000, Cell Signaling, 9272S), anti-Phospho-Akt (Ser473) (1:1000, Absin, abs130002), anti-mGluR8 (1:1000, Affinity, DF7121), anti-GAPDH (1:1000, Abcam, UK, ab8245), goat anti-rabbit IgG-HRP and Cy3-conjugated goat anti-mouse (1:5000, CST, Beverly, MA, USA), and Alexa Fluor®555 goat anti-rabbit IgG(H + L) (1:5000, Invitrogen, Carlsbad, USA). The membranes were treated following the ECL WB Protocol (Bio-Rad, Milan, Italy). The original images were recorded and analysed with Odyssey Infrared Imaging System.

### LNA lipofection in vivo experiments

LNA lipofection in vivo experiments were conducted with reference to previous reports [25–27]. In brief, miR-592 mimic solution (concentration) was mixed with 8 μl of Invivofectamine® 3.0 Reagent (Invitrogen, Carlsbad, USA) and incubated at 50 °C for 30 min. For the positive control okadaic acid (OKA) was dissolved in a final concentration of 2% DMSO. At E14.5, pregnant mice were anaesthetized with isoflurane. The uterine horns were exposed. A lateral ventricle of each embryo was injected using pulled glass capillaries (World Precision Instruments Inc, China) with Fast Green (BBI, Shanghai, China) combined with the LNA lipofection mix or OKA at 1 µl each. Finally, 20 pmol miR-592 mimic or the NC with a total volume of 1 μl were injected period into the one side lateral ventricle of the mouse. Since Lipofectamine mediated the transfection, no electric field was applied following the injection into telencephalic vesicles [28, 29]. The mice were euthanized at various time points after lipofection, and the brains of the mice were dissected for immunofluorescence staining.

### Statistical analysis

Statistical analysis was performed with GraphPad Prism software. The data were presented as the mean ± S.D. or ±S.E.M. as indicated in the figure legends. *P* values <0.05 were considered to indicate a statistically significant difference.

## Results

### Expression pattern of miR-592 indicated an important role in brain development

We first examined the expression of miR-592 in the central nervous system (CNS). To investigate the location of miR-592 in CNS, we first performed FISH on adult mice brains. Colocalization of miR-592 with Tuj1 revealed that miR-592 was expressed in adult mouse neurons of the cortex (Fig. 1a). Accordingly, the expression patterns of miR-592 in WT mice were detected by RT-PCR. The expression of miR-592 increased from E12.5 to E14.5, reached a peak at E16.5, then decreased to a low level around P1 and persisted through adulthood (Fig. 1b). The peak suggests that miR-592 plays an essential role in the early neurogenesis of mice [30]. In ISH, miR-592 was detected mainly in the cell bodies of the ventricular zone (VZ) and subventricular zone (SVZ) at E12.5 and E14.5, while E16.5 to P7, miR-592 was detected most abundant in the intermediate zone (IZ) and cortical plate (CP). miR-592 levels were declined precipitously after P14 (Fig. 1c and Fig. S1a). Given this, miR-592 knockout mouse line was generated by CRISPR/Cas9 with three sgRNAs leading to a 157-bp deletion in *Grm8* (Fig. 1d). PCR genotyping, ISH and RT-PCR results showed effective knockout in miR-592^−/−^ mice (Fig. 1e–h). Isoform-sequencing (Iso-seq) was used to monitor structural variants of *Grm8* transcripts and results indicated that *Grm8* was not affected by knocking out miR-592 (Fig. S1c–g). miR-592^−/−^ mice were viable and fertile, thereby, miR-592^−/−^ mice were mated with WT mice. The heterozygous strains were then cross-bred and used in subsequent trials to reduce the possibility of off-target events [31]. However, an abnormal Mendelian ratio and sex ratio were observed in the progeny of heterozygote outcrosses (Fig. S1b). Further results showed that deletion of the miR-592 in mice caused a 13% arrest of embryonic development (Fig. S1h). We then examined mouse weights at different time points. miR-592^−/−^ mice weighed less than WT mice at the early time points but dramatically increased afterward (Fig. 1i). Nissl staining obtained from aborted miR-592^−/−^ mice displayed neurodevelopmental delay (Fig. S1i, j). In the surviving miR-592^−/−^ mice, there were also irregular processes in the cortices (Fig. 1j). The above results indicate that abnormal developmental symptoms are exhibited after miR-592 knockout.

### miR-592 deletion impairs cortical neurogenesis and adult lineage choice

**Fig. 2:**
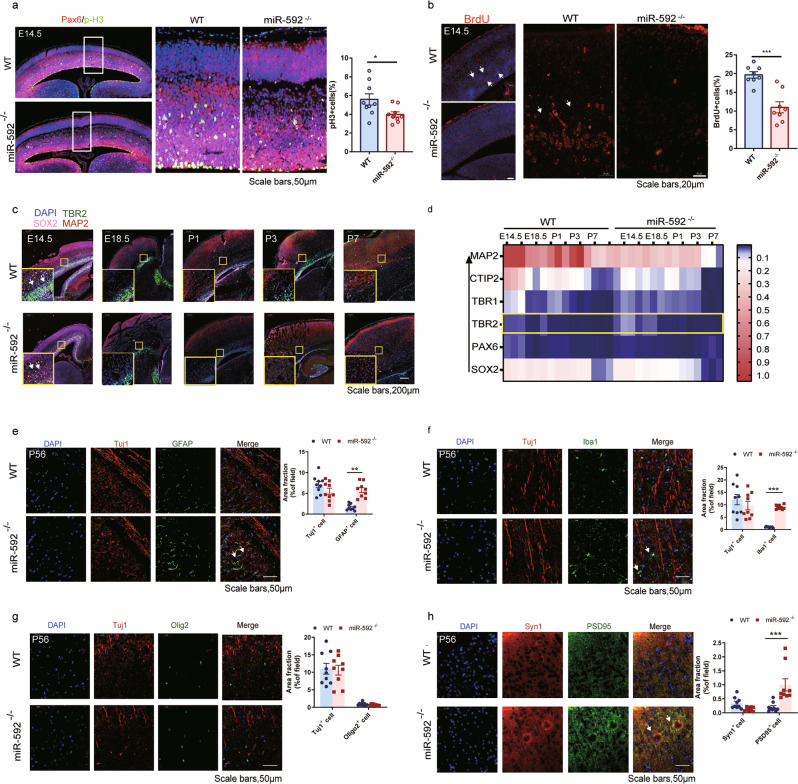
miR-592 deletion impairs cortical neurogenesis and adult lineage choice. **a** Double immunofluorescence staining. Pax6 staining (red immunofluorescence), p-H3 staining (green immunofluorescence), and DAPI staining (blue fluorescence). Unpaired two-tailed *t* test. Error bars represent S.E.M. Experiments were carried out using three biological replicates. Each sample was observed in three fields of view. **b** BrdU staining (red immunofluorescence) and DAPI staining (blue fluorescence). Unpaired two-tailed *t* test. Error bars represent S.E.M. Experiments were carried out using three biological replicates. Each sample was observed in three fields of view. **c** E14.5–P7 sagittal sections were immunostained for the radial glial marker SOX2 (pink), the IPCs marker TBR2 (green), and the neuronal marker MAP2 (red). Nuclei were labelled with DAPI. **d** E14.5–P7 RT-PCR showed that TBR2^+^ cells were dysregulated in the SVZ in miR-592^−/−^ mice beginning at E14.5. VZ/SVZ ventricular/subventricular zone (PAX6 and SOX2), IZ the intermediate zone (TBR2), CP cortical plate (TBR1 and CITP2), MZ marginal zone (MAP2). **e**–**h** Double immunofluorescence staining. **e** Tuj1 staining (red immunofluorescence), GFAP staining (green immunofluorescence), and DAPI staining (blue fluorescence). **f** Tuj1 staining (red immunofluorescence), Iba1 staining (green immunofluorescence), and DAPI staining (blue fluorescence). **g** Tuj1 staining (red immunofluorescence), Olig2 staining (green immunofluorescence), and DAPI staining (blue fluorescence). **h** Syn1 staining (red immunofluorescence), PSD95 staining (green immunofluorescence), and DAPI staining (blue fluorescence). All data were analysed by two-way ANOVA followed by Tukeys multiple comparison test. Error bars represent S.E.M. *p < 0.05, **p < 0.01, ***p < 0.001. The averages of nine different fields of view were calculated for each animal (counts from three fields of three sagittal slices).

All other organs examined in adult miR-592^−/−^ mice were histologically unremarkable (Fig. S2a). Therefore, we focused mainly on miR-592^−/−^ mouse brains. We first examined the NPC levels in the developing cortices of miR-592^−/−^ mice. We used p-H3 and Pax6 to label mitotic cells and apical neural progenitor cells (APCs) in the VZ/SVZ of E14.5 mouse brains [32]. Interestingly, ectopic expression of Pax6 was induced by miR-592 knockout in E14.5 mouse brains. The percentage of p-H3^+^ cells were significantly decreased in miR-592^−/−^ mice compared with WT (Fig. 2a). Then we carried out BrdU labelling studies at E14.5. The results showed a significant decrease in the percentage of BrdU^+^ cells out of total cells in the VZ/SVZ regions of E14.5 miR-592^−/−^ brains, suggesting a decreased cell proliferation rate in the miR-592^−/−^ cerebral cortex (Fig. 2b). Next, we examined the intermediate progenitor cells (IPCs) markered by TBR2 [33]. Remarkably, we observed that nearly complete colocalization was observed between TBR2^+^ and SOX2^+^ cells in miR-592^−/−^ mice at E14.5 and TBR2^+^ IPCs were reduced during neurogenesis (Fig. 2c, d). SOX2 labels both APCs and IPCs [34–36]. It suggests that miR-592 deletion may affect quiescent NPC activation and their lineage choice in the developing cortices.

The sagittal section of adult miR-592^−/−^ mice and WT mice were immunostained for Nestin, Ki67, Tuj1, GFAP, Iba-1, Olig2, and CD31 to test whether the irregular lineage choice affected terminal differentiation (Fig. 2e–h and Fig. S2b, f). Nestin^+^ and Ki67^+^ double-positive cells were also significantly decreased in adult miR-592^−/−^ *mice* compared with WT (Fig. S2b). Remarkably, an imbalance in neuronal versus glial lineage differentiation was detected. The numbers of astrocytes (GFAP^+^) or microglia (Iba-1^+^) were markedly increased in the cortices of adult miR-592^−/−^ mice, whereas those of neuron (Tuj1^+^) were relatively decreased (Fig. 2e, f). We next investigated whether this change in miR-592^−/−^ mice showed a similar trend on both sides of the brain. P56 coronal section results of WT mice and miR-592^−/−^ mice showed that both the left and right brains hemispheres exhibited increased astrocytosis (Fig. S2c). These results indicated that miR-592 may be also involved in the activation of astrocytes and microglia. Since neuroinflammation may be associated with neuronal apoptosis, we next examined whether miR-592 deletion was able to reduce neuronal apoptosis in the cortex. As expected, cleaved caspase 3 and NeuN-double-positive cells levels increased in the cortices of miR-592^−/−^ mice (Fig. S2d). This result was confirmed by TUNEL^+^ cells which significantly increased in the cortices of miR-592^−/−^ mice compared with that seen in WT mice (Fig. S2e). Meanwhile, Glial cells have been associated with synaptic pruning and modulation of neurotransmission [37–39]. PSD95 and Syn1 immunohistochemistry were used to determine whether miR-592 knockout affected dendritogenesis and synapse development. PSD95 immunostaining demonstrated significantly increased intensities (Fig. 2h). In addition, no noticeable change in CD31^+^ endothelial cells was observed in miR-592^−/−^ mice (Fig. S2f). We conclude that miR-592 knockout impairs cortical neurogenesis and adult lineage choice.

### Genetic deletion of miR-592 reduces motor coordination and social interaction

**Fig. 3:**
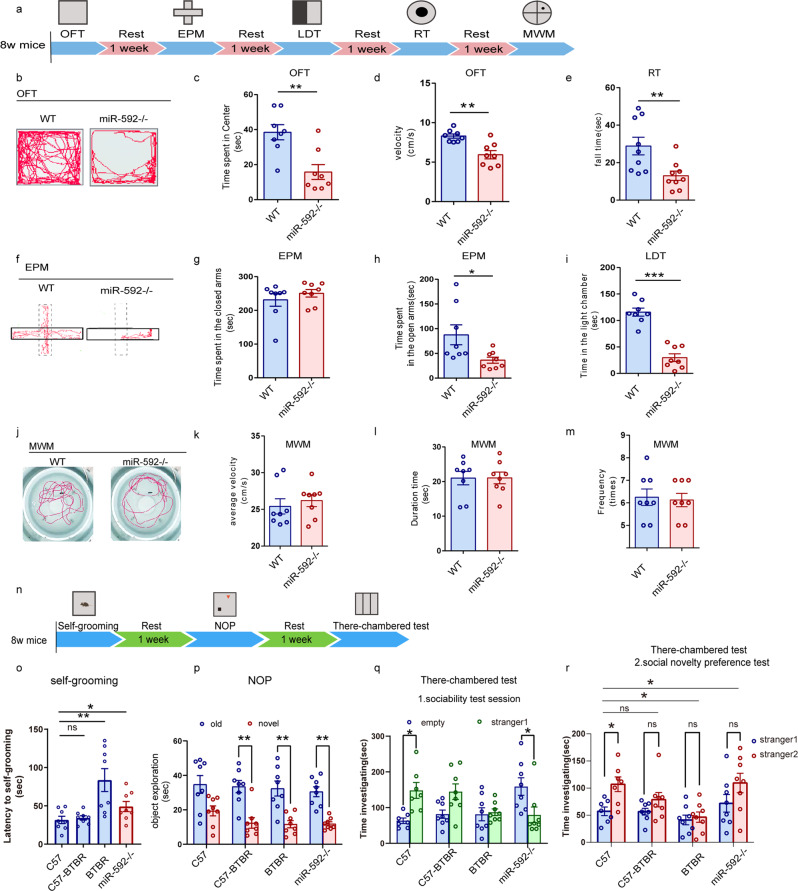
Genetic deletion of miR-592 reduces motor coordination and social interaction. **a** Timeline of tests for extensive behaviours including movement and coordination, social behaviours, learning and memory. **b**–**d** OFT, open field test. **b** Representative images showing typical examples of the exploration behaviour of WT and miR-592^−/−^ mice during the first 5 min of the open field test. **c** The time spent in the centre and **d** the velocity was scored for each 5 min period (unpaired two-tailed *t* test, *n* = 8 mice for each genotype, error bars represent S.E.M.). **e** RT rotarod test. The time on the rotating bar was recorded in the rotarod test (unpaired two-tailed *t* test, *n* = 8 mice for each genotype, error bars represent S.E.M.). **f**–**h** EPM elevated plus maze test. **f** Representative images showing typical examples of the exploration behaviour of WT and miR-592^−/−^ mice during the first 5 min of the elevated plus-maze test are provided. The time spent by WT and miR-592^−/−^ mice in **g** closed arms and **h** open arms during the elevated plus-maze assay was quantified (unpaired two-tailed *t* test, *n* = 8 mice for each genotype, error bars represent S.E.M.). **i** LDT light–dark transition test. The latency to the first entry into the light chamber in the light/dark box assay is shown (unpaired two-tailed *t* test, *n* = 8 mice for each genotype, error bars represent S.E.M.). **j**–**m** MWM Morris Water Maze. **j** Representative images showing typical examples of exploring the behaviour of WT and miR-592^−/−^ mice during the first 10 min of the water maze task are provided. **k** The average velocity, **l** time spent in the target-quadrant areas, and **m** frequency of entry into the target quadrants were recorded after 7 days of training session (unpaired two-tailed *t* test, *n* = 8 mice for each genotype, error bars represent S.E.M.). **n** Timeline of tests for social interaction behaviours. **o** Self-grooming. Self-grooming assessed over a 10 min period (one-way ANOVA with Tukey’s multiple comparison test, *n* = 8 mice for each genotype, error bars represent S.E.M.). **p** NOP novel object preference test. Object exploration was allowed to test the novelty preference of WT and miR-592^−/−^ mice (two-way ANOVA with Tukey’s multiple comparison test, *n* = 8 mice for each genotype, error bars represent S.E.M.) **q**, **r** Three-chambered test. Experimental mice were socialized by exposure to a stranger mouse over a 3-day training period and then placed with a novel mouse. **q** Sociability test session. **r** Social novelty preference test. The time spent in the stranger mouse zone was quantified (two-way ANOVA with Tukey’s multiple comparison test, *n* = 8 mice for each genotype, error bars represent S.E.M.). *p < 0.05, **p < 0.01, ***p < 0.001.

To further verify the effects of miR-592 on the functions of cortical neurons, we next subjected adult animals to a battery of behavioural tests and investigated its impact on neurobehaviour (Fig. 3a). We found that miR-592^−/−^ mice arrived at the centre zone significantly less frequently than WT mice in the OFT (Fig. 3b–d). Motor coordination and balance deficits in miR-592^−/−^ mice were confirmed in RT, in which the time to fall from a rot rod was tested (Fig. 3e). Compared to the WT mice the miR-592^−/−^ mice spent less time in the open arms of the EPM (Fig. 3f–h). Similarly, miR-592^−/−^ mice showed limited curiosity, spending less time in the light chamber in LDT than the WT mice (Fig. 3i). We further investigated whether learning and cognition were affected in miR-592^−/−^ mice. No significant differences were detected between the WT group and the miR-592^−/−^ group in the distance travelled in the centre and time spent in the centre area in the MWM (Fig. 3j–m). Therefore, we investigate whether miR-592 knockout impairs motor coordination and/or reduces social interaction in mice. BTBR mice were used as a positive control to adopt a standard measure of social interaction deficits [40–42]. Compared to WT littermates, miR-592^−/−^ mice showed more prolonged bouts of self-grooming (Fig. 3o). We next assessed the novelty preference of miR-592^−/−^ mice by NOP test and evaluated sociability using the three-chambered test. In the NOP test, miR-592^−/−^ mice presented limited preference for a new object (Fig. 3p and Fig. S5h). For the three-chambered test, in the sociability test session, we found that WT C57 mice have a clear tendency to be curious about stranger mice, while miR-592^−/−^ mice lacked interest in interaction with stranger mice (Fig. 3q). In the social novelty preference test, miR-592^−/−^ mice and BTBR mice show less preferences for stranger2 than WT mice (Fig. 3r). These data indicated that miR-592^−/−^ mice exhibited autism-like behaviours such as stereotyped behaviour, decreased novelty preference, and decreased sociability.

### Integrated bioinformatics analysis to identify targets of miR-592

**Fig. 4:**
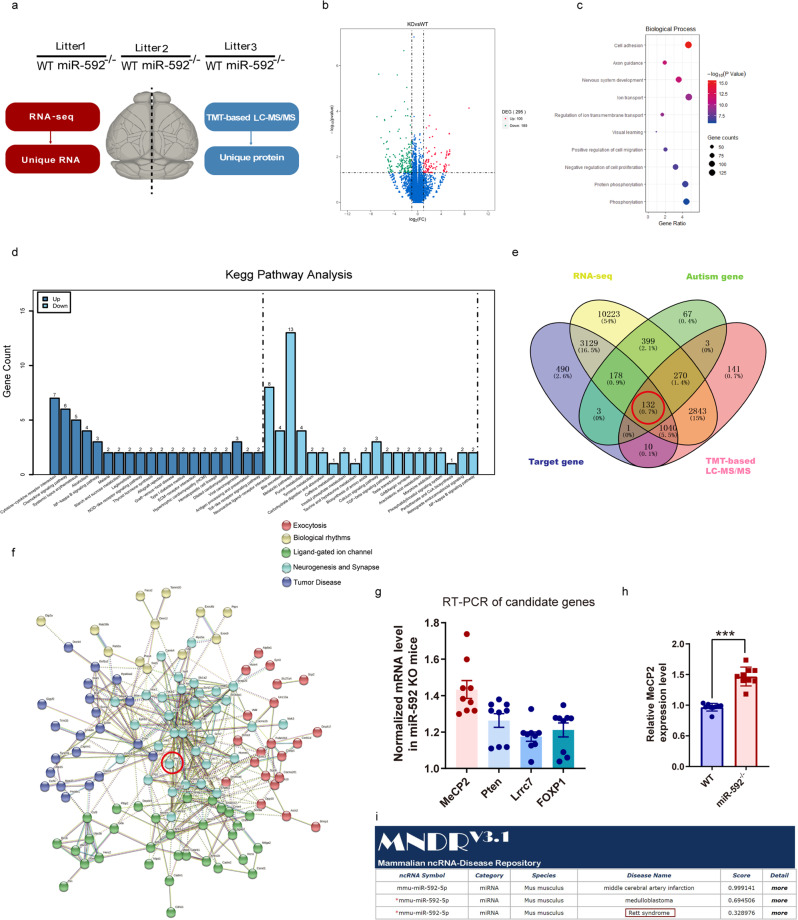
Integrated bioinformatics analysis to identify mRNA targets of miR-592. **a** Workflow of the RNA-seq and TMT proteomic analyses. Six mouse brain cortical samples from three litters (2 genotypes per littler) were used as described in the ‘Materials and methods’ section. **b** Volcano map of DEGs. Upregulated genes are represented by red spots, while downregulated genes are represented by green spots. **c** Bubble chart showing the biological process terms of RNA-seq. **d** GO enrichment analysis of the DEGs identified in the RNA-seq analysis (*n* = 106 for upregulated DE genes; *n* = 189 for downregulated DE genes). All the results of the analysis are presented in Fig. 4e, f. **e** Venn diagram. Venn diagram illustrating the potential miR-592 targetgenes profile that intersected with the RNA-seq DEG set, TMT DEG set, the targetgene set, and the highest-ranking autism risk gene set. **f** Cluster analysis of 132 predicted genes using STRING (http://string-db.org). Some proteins, such as MeCP2, have many interacting partners (hubs). **g**, **h** RT-PCR. **g** Candidate targets’ mRNA levels in WT and miR-592^−/−^ mice cortices. Experiments were repeated nine times (three biological and three analytical repeats). **h** miR-592 regulated MeCP2 in WT and miR-592^−/−^ mice cortices. MeCP2 expression normalized to GAPDH expression was assayed. Unpaired two-tailed *t* test. All data are presented as mean ± S.E.M. ***p < 0.001. Experiments were repeated nine times (three biological and three analytical repeats). **i** Critical diseases related to miR-592 from the MNDR V3.1 database (www.rna-society.org/mndr/).

We aimed to gain further insight into the abnormal molecular mechanism. We used adult miR-592^−/−^ mice and WT littermates for bioinformatics analysis (Fig. 4a and Fig. S3a–d). A total of 295 differentially expressed genes (DEGs) were recognized by RNA-seq analysis, of which 106 were upregulated and 189 were downregulated (Fig. 4b). We performed GO term enrichment and KEGG pathway enrichment analysis to elucidate the functional roles of these DEGs. The biological process (BP) analysis showed that the DEGs were significantly involved in cell adhesion, and axon guidance (Fig. 4c and Fig. S3e–g). KEGG pathway analysis showed that the upregulated pathways were remarkably involved in cytokine–cytokine receptor interaction and chemokine signalling pathway, which play essential roles in autism [43]. Simultaneously, the downregulated genes were enriched in metabolic pathways, and neuroactive ligand–receptor interaction (Fig. 4d). Replicates that possessed consistent TMT protein quantification results are shown (Fig. S3d). To identify targetgenes of miR-592 in the mouse brains, we proceeded to identify targetgenes in the TargetScan 7.1 database (Supplementary Table 2). The produced Venn diagram illustrates the potential miR-592 targetgenes that intersected with the RNA-seq DEG set (18216 gene names), TMT protein quantification DEG set (4465 protein names), targetgene set (4988 gene names), and highest-ranking autism gene set (1054 gene names). Notably, by overlapping with these genes, we found that 12.7% (132 of 1054) of the predicted candidate targetgenes were among the highest-ranking autism genes identified in the SFARI database (Fig. 4e). Next, we performed a PPI network investigation to analyse the functions of these candidates. The network was divided into five clusters, involving exocytosis, biological rhythms, ligand-gated ion channels, neurogenesis and synapses and tumour disease. As expected, the core cluster was involved in neurogenesis and synapses (Fig. 4f). Some genes in this cluster have been reported as hub genes in many ASD patients, such as MeCP2, phosphatase, tensin homologue (PTEN), leucine-rich repeat-containing 7 (Lrrc7), and forkhead box P1 (Foxp1). RT-PCR revealed that these genes were upregulated in miR-592^−/−^ mouse brains, especially MeCP2 (Fig. 4g, h). We searched the MNDR V3.1 database (www.rna-society.org/mndr/) which was previously reported as an ncRNA-disease database. The results showed that miR-592 defects might be associated with MeCP2-related RTT (Fig. 4i). Besides, MeCP2 was expressed at relatively high levels in neurons [44]. Thus, MeCP2 was selected for further investigation.

### MeCP2 is a targetgene of miR-592, and the MeCP2-BDNF axis is regulated by miR-592 during development

**Fig. 5:**
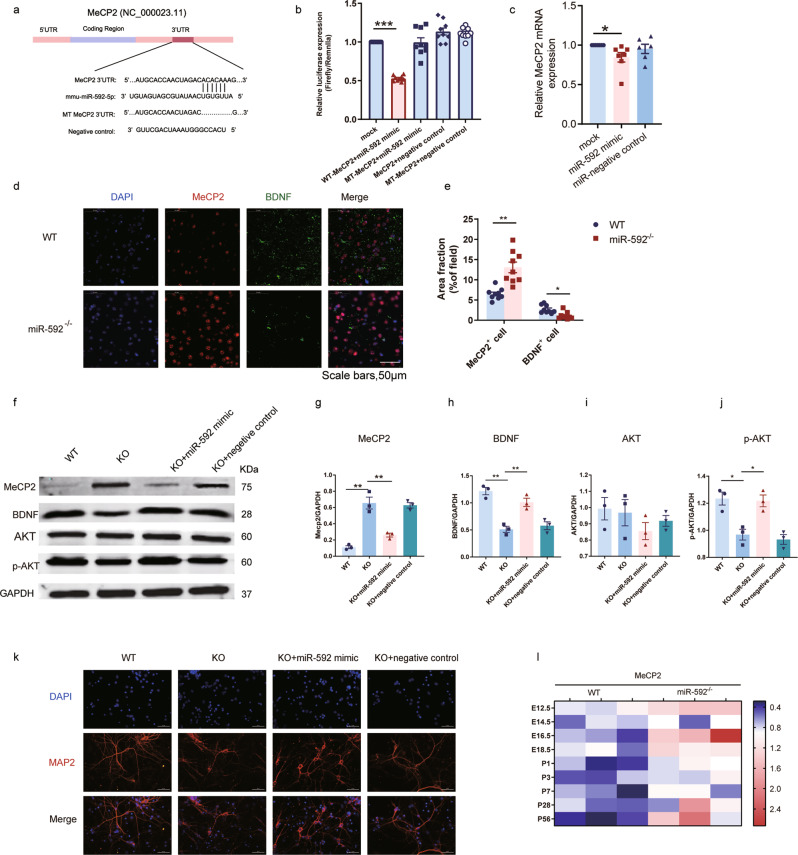
MeCP2 is a targetgene of miR-592, and the MeCP2–BDNF axis is regulated by miR-592 during development. **a** Schematic diagram of the exact position at which miR-592 targets the mouse MeCP2 3’-UTR. The seed match region was replaced in a site-directed mutation experiment. **b** The normalized luciferase (Renilla/firefly) values are shown. 293T cells were co-transfected with MeCP2 3’-UTR pGL3 plasmids and miR-592 mimics/NC, or mutant MeCP2 (MT- MeCP2) 3’-UTR pGL3 plasmids and miR-592 mimics/NC (one-way ANOVA with Tukey’s multiple comparison test, error bars represent S.E.M.). Experiments were repeated nine times (three biological and three analytical repeats). **c** RT-PCR. miR-592 regulated MeCP2 in cultured cortical neural progenitor cells. miR-592 mimics were transfected with Xfect. MeCP2 expression normalized to GAPDH expression was assayed by RT-PCR (one-way ANOVA with Tukey’s multiple comparison test, error bars represent S.E.M.). Experiments were repeated nine times (three biological and three analytical repeats). **d**, **e** The MeCP2–BDNF axis was regulated by miR-592 in the cortex. **d** Immunofluorescence staining was performed using MeCP2 and BDNF antibodies. The relative fluorescence intensity of MeCP2 elevated upon the loss of miR-592 in the cortex, and that of BDNF decreased upon the loss of miR-592 in the cortex (two-way ANOVA with Tukey’s multiple comparison test, error bars represent S.E.M.). Experiments were repeated nine times (three biological and three analytical repeats). **e** Image analysis and quantification were performed using the ImageJ software. **f**–**j** WB western blot. **f** miR-592 knockout resulted in a significant increase in MeCP2 and a significant decrease in BDNF (relative to GAPDH), which was associated with the reduced phosphorylation of AKT (P-AKT) without changes in total AKT levels (one-way ANOVA with Tukey’s multiple comparison test, error bars represent S.E.M.). Experiments were repeated nine times (three biological and three analytical repeats). Cultured cortical neurons of miR-592^−/−^ mice were rescued by transfection with the miR-592 mimic. Quantification of **g** MeCP2, **h** BDNF, **i** AKT, **j** p-AKT, normalized to GAPDH levels, and values were plotted. **k** Images of neurons that were induced after 48 h of transfection with either miR-592 mimic or NC. **l** miR-592 knockout resulted in increased expression of MeCP2 mRNA. *p < 0.05, **p < 0.01, ***p < 0.001.

To investigate miR-592-mediated mRNA regulation, we started with a dual luciferase assay in 293T cells. We co-transfected miR-592 with a pGL3 luciferase reporter vector constructed with a 300 bp sequence including the 3’-UTR seed region of MeCP2. Firefly and Renilla luciferase activity levels were detected and showed that MeCP2 was the direct targetgene of miR-592 (Fig. 5a). We found that miR-592 mimic overexpression caused significant downregulation of MeCP2 but had a limited effect on mutant MeCP2 (Fig. 5b). To further confirm whether MeCP2 was the targetgene in NPCs, we next transfected miR-592 into NPCs isolated from the embryonic cortices at E14.5. MeCP2 showed apparent downregulation at the mRNA level (Fig. 5c). The MeCP2 protein level was ~2-fold higher in miR-592^−/−^ mice than in WT littermates. The brain-derived neurotrophic factor (BDNF) expression level was ~3-fold lower in miR-592^−/−^ mice than in WT littermates (Fig. 5d, e). WB showed that the increase in MeCP2 in miR-592^−/−^ mice was related to phosphorylation of AKT, which was directly regulated by BDNF in the PI3K/AKT signalling pathway, with no alteration in the total protein level of AKT (Fig. 5f–j). miR-592 mimics and NC were transfected into NPCs of WT and miR-592^−/−^ mice, respectively. RT‐PCR results showed a remarkable increase in miR-592 expression after transfection (Fig. S4g). To initiate differentiation, the medium of dissociated NPCs was changed to neural differentiation medium. miR-592 overexpression resulted in an increase in MAP2 expression, indicating that miR-592 overexpression promoted the differentiation of NPCs into neurons (Fig. 5k). We next examined the expression of MeCP2 and BDNF during mouse brain development by RT-PCR. Normalized to GAPDH, the expression of MeCP2 in miR-592^−/−^ mice was higher than that in WT littermates at E12.5. This increase continued until adulthood (Fig. 5l and Fig. S4c–f). Thus, the results suggest that the MeCP2–BDNF axis is regulated by miR-592 during the developmental stage.

### Lipofection of miR-592 normalizes MeCP2 levels and partly reverses abnormal behaviour

**Fig. 6:**
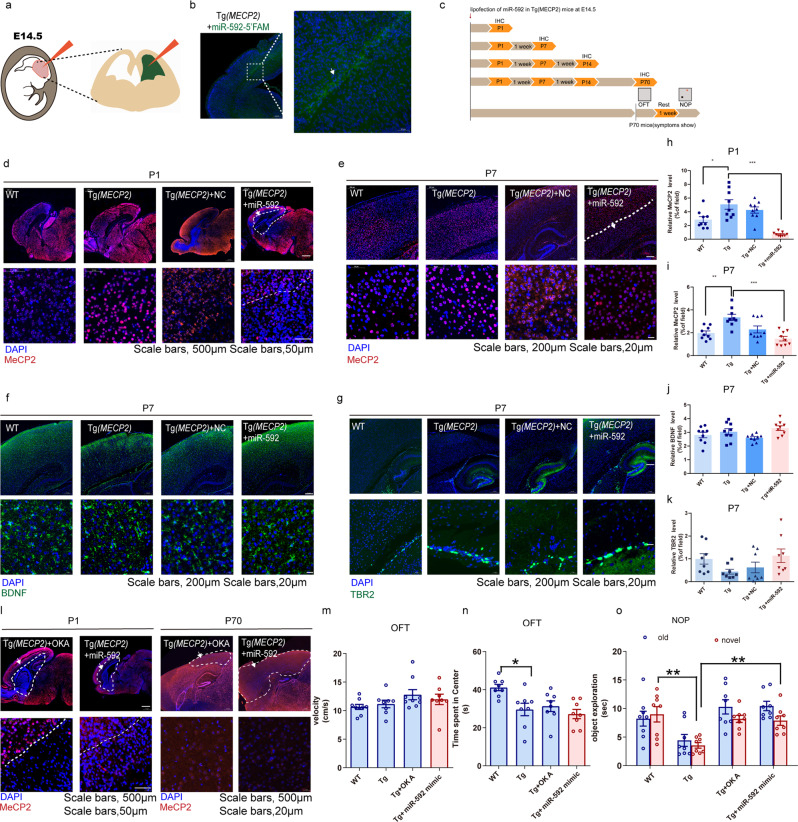
Phenotype reversal in Tg(*MECP2*) mice using miR-592. **a** Schematics of the LNA experiment in utero. Brains were injected with Lipofectamine/miR-592 mimic at E14.5. The injected side (visualized by adding fast green to the LNA mixture) was compared to the contralateral uninjected side. **b** Brains were injected with Lipofectamine/miR-592–5’FAM at E14.5. **c** Timeline of miR-592 mimic treatment, immunofluorescence and behavioural tests. **d**–**k** Immunofluorescence showing the MeCP2 response to miR-592 mimic treatment at **d** P1 and **e** P7. **f** BDNF staining (green immunofluorescence), **g** TBR2 staining (green immunofluorescence), and DAPI (blue fluorescence). **h**–**k** Quantified fluorescence intensity data are shown in the bar graph. **h** Quantification of MeCP2 expression by immunofluorescence at P1. **i** Quantification of MeCP2 expression by immunofluorescence at P7. **j** Quantification of BDNF expression by immunofluorescence at P7. **k** Quantification of TBR2 expression by immunofluorescence at P7. The averages of nine different fields of view were calculated for each animal (counts from three fields of view/ sagittal slice). **l** Immunofluorescence showing the MeCP2 response to miR-592 mimic and OKA treatment at P1 and P70 (one-way ANOVA with Tukey’s multiple comparison test, error bars represent S.E.M.). The averages of nine different fields of view were calculated for each animal (counts from three fields of view/ sagittal slice). **m** OFT open field test. The velocities of 70-day-old WT and Tg(*MECP2*) mice (12 weeks after OKA and miR-592 mimic treatment) were recorded (one-way ANOVA with Tukey’s multiple comparison test, *n* = 8, error bars represent S.E.M.). **n** OFT open field test. The time spent in the centre was recorded for 70-day-old WT and Tg(*MECP2*) mice (12 weeks after OKA and miR-592 mimic treatment) (one-way ANOVA with Tukey’s multiple comparison test, *n* = 8, error bars represent S.E.M.). **o** NOP novel object preference test. The curiosity behaviour of 70-day-old WT and Tg(*MECP2*) mice (12 weeks after OKA and miR-592 mimic treatment) was tested (two-way ANOVA with Tukey’s multiple comparison test, *n* = 8, error bars represent S.E.M.). *p < 0.05, **p < 0.01, ***p < 0.001.

Tg(*MECP2*) mice are distinguished by autism at approximately 10 weeks of age and express the gene at ~2-fold *MECP2* levels in the brain [45]. Restoration of normal *MECP2* levels has been shown to improve behavioural and histological deficits, making it an appealing treatment [46, 47]. Although a miR-592 decrease trend towards in Tg(*MECP2*) mice was observed, the difference did not reach statistical significance (Fig. S5a). We hypothesized that mice in the period of normal development would receive some benefit from miR-592 treatment. We used miR-592 mimics for lipofection to normalize the expression level of MeCP2 in vivo at E14.5 (Fig. 6a). 5’FAM miR‐592-labelled cells were detected in the cortex (Fig. 6b). Next, we tested different miR-592 mimic doses and found the safety margin of *MECP2* levels in a dose-dependent manner (Fig. S5b). After lipofecting the miR-592 mimic (20 pmol), the immunofluorescence staining result showed that the normalized cells were located within the brain parenchyma in proximity to the ventricle. We analysed the expression of MeCP2 in the mouse cortices at different time points after miR-592 lipofection. MeCP2 was effectively knocked down close to the level in the WT group at P1 and P7(Fig. 6d, e, h, i). Although a trend towards increased BDNF was observed at P7, the difference did not attain statistical significance (Fig. 6f, j). After lipofection of the miR-592 mimic, the decreasing trend of TBR2 in Tg(*MECP2*) mice was inhibited at P7 (Fig. 6g, k). Next, we investigated whether this effect was temporally constrained. MeCP2 was significantly downregulated Tg(*MECP2*) mice at P1 and P70 after miR-592 lipofection (Fig. 6l and Fig. S5c). OKA has been suggested to be a druggable regulator of MeCP2 stability, targeting of which partly rescues both MeCP2 overexpression and motor abnormalities in mouse models of MDS [48]. In the miR-592-treated group, MeCP2 was effectively knocked down close to the level in the OKA-treated group at P1 and P70 (Fig. S5c and Fig. 6l). Given the significant decrease in MeCP2 protein level after miR-592 treatment, we hypothesized that miR-592 could ameliorate the motor impairments and/or social interaction deficits in Tg(*MECP2*) mice. Numerous studies have shown that abnormal social behaviour in Tg(*MECP2*) mice can be detected by the open-field test and the novel object preference test [14, 49–51]. In the OFT, no improvement was demonstrated in the miR-592-treated group compared with the OKA-treated group, because the miR-592 mimic did not significantly change the velocity or time spent in the centre zone (Fig. 6m, n and Fig. S5f). The rescue effect was only apparent in the NOP test. We found that in Tg(*MECP2*) mice, the miR-592-treated exhibited more curiosity than the untreated Tg(*MECP2*) mice. Such behaviour resembled more closely to that observed in WT mice (Fig. 6o and Fig. S6g). Together, these results suggest that the overexpressed MeCP2 in MDS mice can be rescued by miR-592 and even partially rescue the MDS phenotype.

## Discussion

**Fig. 7:**
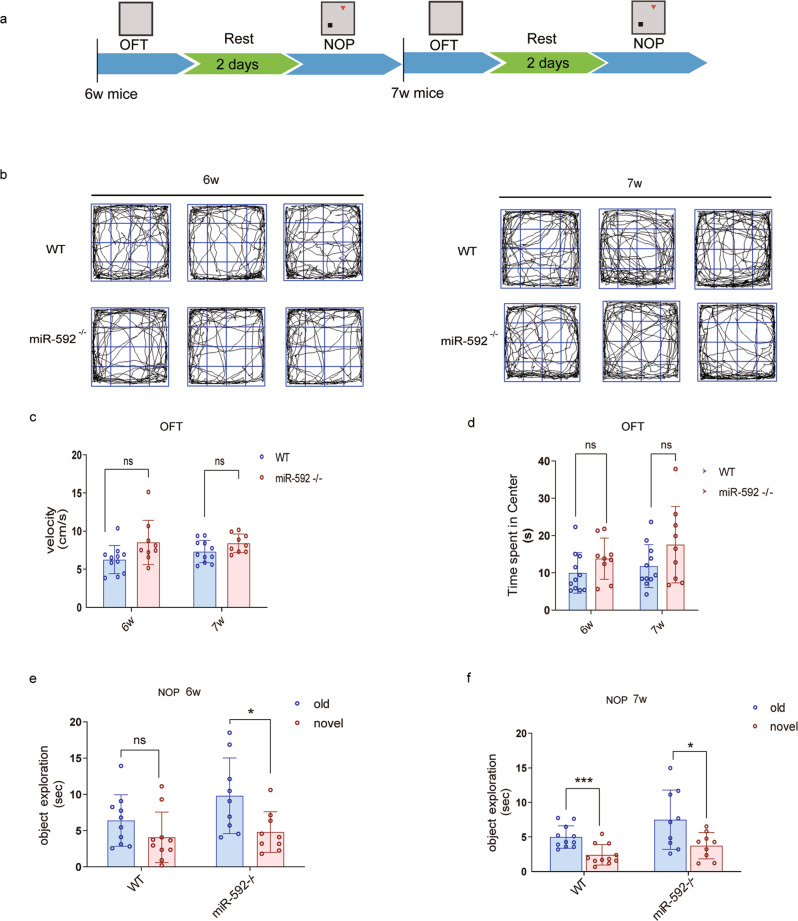
Genetic deletion of miR-592 reduced social interaction at early stage. **a** Timeline of younger behavioural tests. **b** OFT open field test. Representative images showing typical examples of the exploration behaviour of WT and miR-592^−/−^ mice during the first 5 min of the open field test. 6W: 6 weeks after birth. 7W: 7 weeks after birth. **c** OFT open field test. The velocity was scored for each 5 min period. 6W: 6 weeks after birth (*n* = 8). **d** The time spent in the centre was scored for each 5 min period. 7W: 7 weeks after birth (one-way ANOVA with Tukey’s multiple comparison test, *n* = 8, error bars represent S.E.M.). **e** NOP novel object preference test. The curiosity behaviour was performed to test the social novelty of WT and miR-592^−/−^ mice at the sixth week. 6W: 6 weeks after birth (two-way ANOVA with Tukey’s multiple comparison test, *n* = 8, error bars represent S.E.M.). **f** NOP novel object preference test. The curiosity behaviour was performed to test the social novelty of WT and miR-592^−/−^ mice at the seventh week. 7W: 7 weeks after birth (two-way ANOVA with Tukey’s multiple comparison test, *n* = 8, error bars represent S.E.M.). *p < 0.05, ***p < 0.001.

Several consistently deregulated miRNAs have been detected in ASD patient-derived samples, including hsa-miR-592 [1, 52]. By RNA-Seq, miR-592 expression in the hippocampi of MeCP2 KO mice was observed to be 1.67-fold higher than that in the hippocampi of WT mice. Conversely, expression of miR-592 decreased significantly in MeCP2 overexpressing neurons [44]. This biomarker characterization of miR-592 variants may serve the differential diagnosis of neurodevelopmental disorder. In the present study, we obtained direct evidence that miR-592 knockout caused motor impairments and/or social interaction deficits. To test whether motor impairments gave a false impression of the social preference, the behavioural experiments were performed at the sixth and seventh weeks. The difference in body weight between the miR-592^−/−^ mice and WT littermates was not statistically significant over both two periods (Fig. 7a). In the OFT, the difference between the two groups was not statistically significant over both periods (Fig. 7b–d). However, miR-592^−/−^ mice already exhibited limited novelty preference for a new object in 6w (Fig. 7e, f). Additionally, we observed that miR-592 knockout significantly influenced the expression of many recognized neurodevelopment and metabolism genes by systematic bioinformatics analysis. It may lead to differences in body weight during development. Evidence from recent research focussing on miR-592 and obesity further supports this conjecture [53].

Formerly, most studies concentrated on the relationship between miR-592 tumour-associated events [54]. Here, we showed that miR-592 knockout resulted in a reduction in IPC-to-mature neuron transition and ultimately resulted in distinct lineage outcomes in the adult cortex. A previous study has shown that most neurons, oligodendrocytes, and astrocytes originate from IPCs [55]. Around E16.5 (a peak in miR-592 expression), cortical RGCs undergo lineage specification changes [56, 57]. Overall, crosstalk between cell types is likely a critical part of molecular changes that limit neuron function and/or survival [58, 59]. Single-cell sequencing will better resolve specific neural cell-fate signatures [60].

miR-592 seems to take more regulation at MeCP2’s post-transcriptional level. MeCP2 has been reported to be a regulator of embryonic neurodevelopment processes, including neurogenesis and differentiation [44, 61–63]. Previous studies have shown that BDNF is a well-established neurotrophic factor, and it is the downstream effector of MeCP2 [64]. Therefore, we verified the MeCP2/BDNF/Akt pathway in miR-592^−/−^ mice, which is known to be early neurodevelopment and cell proliferation [65].

We sought a strategy to directly target MeCP2 with miR-592 at an early stage E14.5. Overall, our results reveal that miR-592 is an essential neurodevelopmental-associated endogenous miRNA. Delivering miR-592 to the CNS during development may be an effective strategy to ameliorate neurodevelopmental disorders with impaired MeCP2.

## Supplementary information

Supplymentary

Attribution of Authorship

Reproducibility checklist

Original Data File

Supplymentary table

## Data Availability

The data that support the findings of this study are available from the corresponding author upon reasonable request.
