# Supplementary material for: Loss of neurodevelopmental-associated miR-592 impairs neurogenesis and causes social interaction deficits
Source: Cell Death Dis. 2022 Apr 1;13(4):292. doi: 10.1038/s41419-022-04721-z (PMC8976077; doi:10.1038/s41419-022-04721-z)
Supplement: Supplementary file 1 — Supplymentary [file 41419_2022_4721_MOESM1_ESM.pdf]

Supplementary

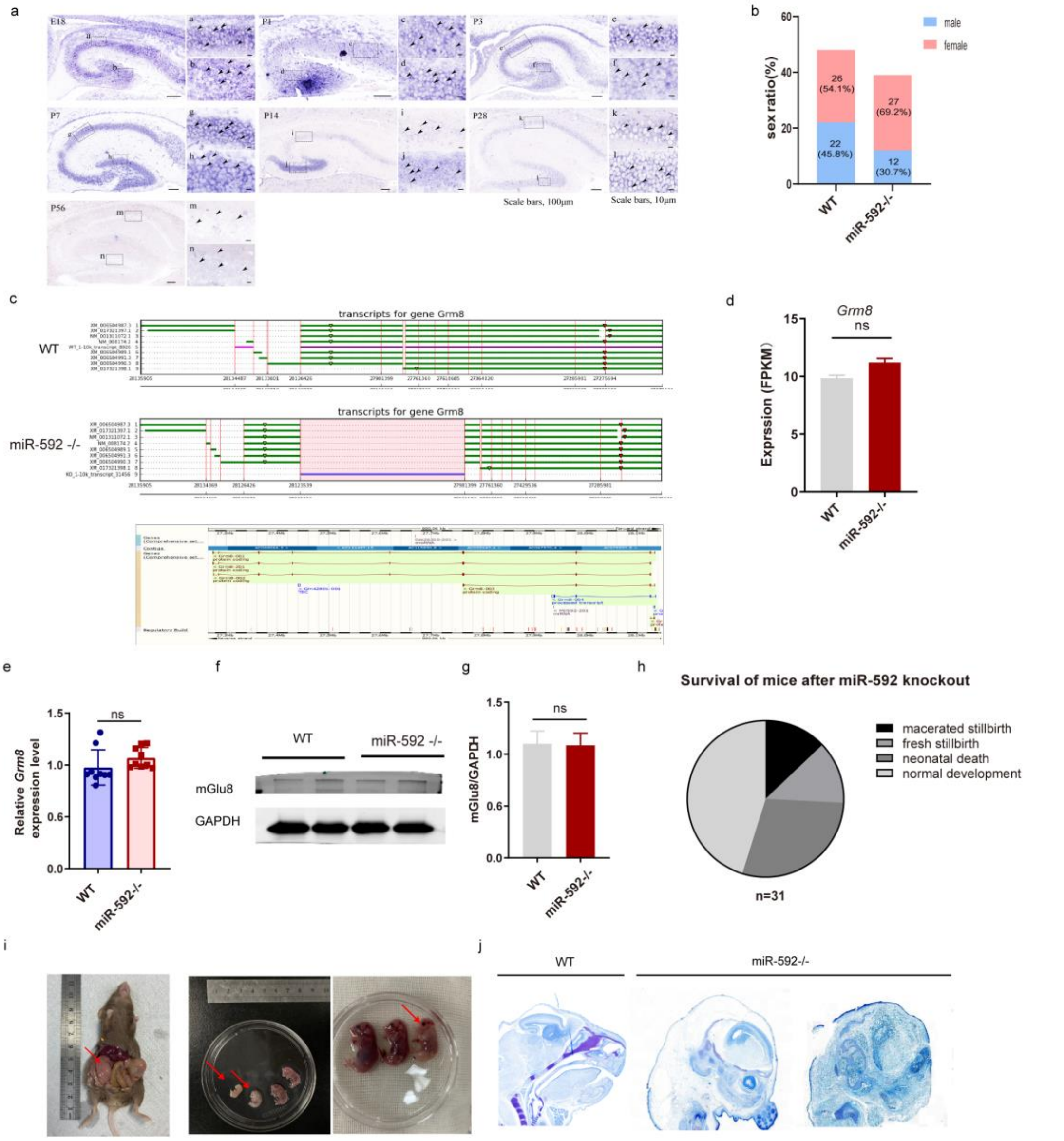

Fig. S1. Global deletion of miR-592 leads to elevated perinatal lethality. (a) Distribution of miR-592 in sagittal sections of the developing hippocampus at E12.5 to P56. (b) WT and miR-592$^{-/-}$ mice litter sex ratios. (c) Analysis by ISO-seq of miR-592 expression in the WT and miR-592$^{-/-}$ mice cerebral cortex. The structure of GRM8 changed by knock-out. (d) Analysis by ISO-seq of GRM8 expression in the developing cerebral cortex (Unpaired Two-Tailed *t*-test). Error bars represent S.E.M. Experiments were repeated nine times (three biological and three analytical repeats). (e) Analysis by RT-PCR of *GRM8* expression in the developing cerebral cortex (Unpaired Two-Tailed *t*-test). Error bars represent S.E.M. Experiments were repeated nine times (three biological and three analytical repeats). (f, g) Expression of mGlu8

in cortex evaluated by (f) WB and (g) the quantification histogram of mGlu8 level (Unpaired Two-Tailed *t*-test). Error bars represent S.E.M. Experiments were repeated nine times (three biological and two analytical repeats). (h) Overall survival of mice after miR-592 knockout. (i, j) (i) miR-592 associated with embryonic lethality, pregnancy-associated abnormalities. (j) Nissl staining shows evidence of cortical developmental malformations.

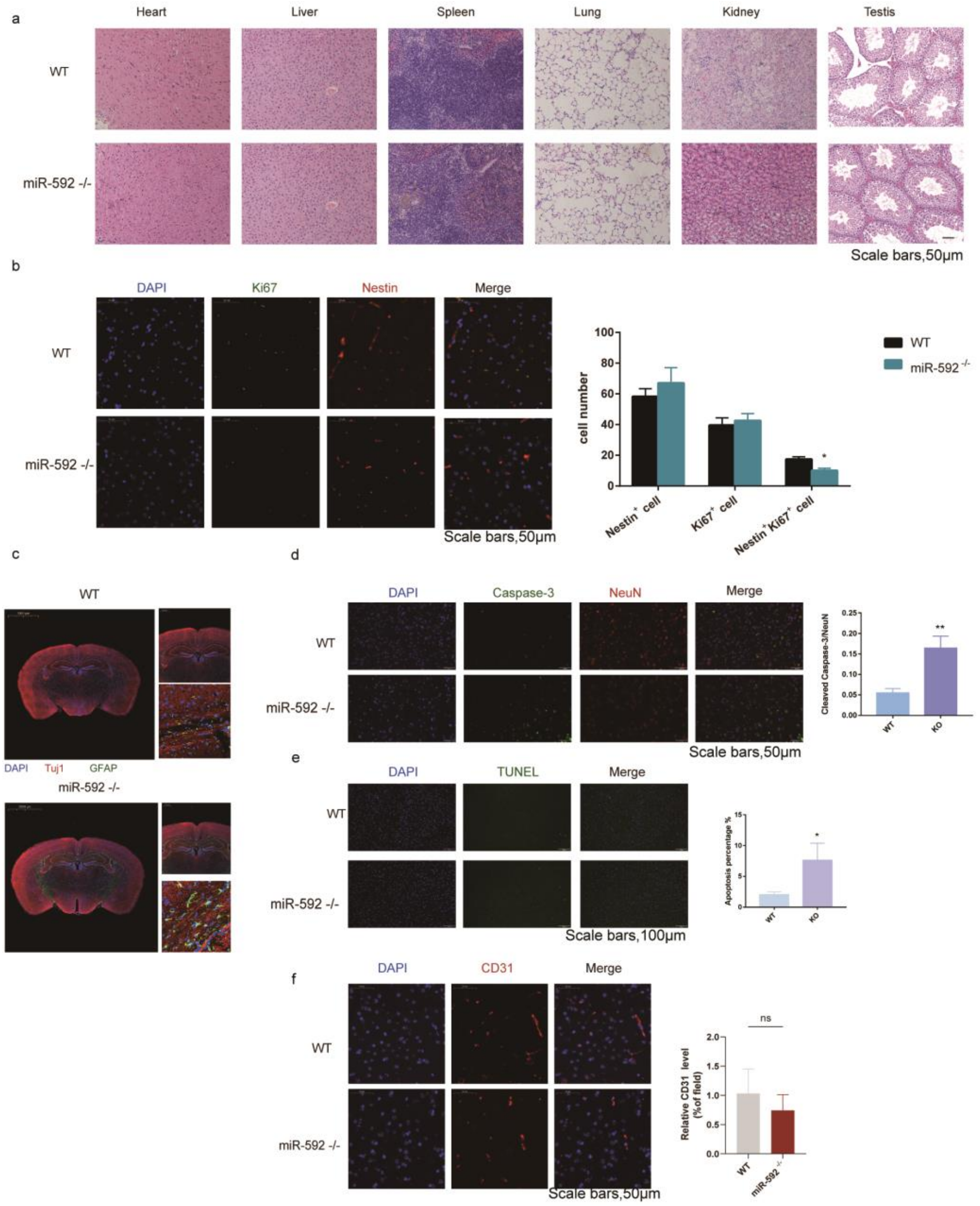

Fig. S2. Manipulation of IPCs fates by miR-592 results in distinct lineage outcomes in the adult cortex. (a) No abnormal morphology was observed in the heart, kidney, liver, lung, spleen, and testis. (b) Double immunofluorescence staining. Nestin staining (red immunofluorescence), Ki67 staining (green immunofluorescence), and DAPI staining (blue fluorescence). two-way ANOVA with Tukeys multiple comparison test. Error bars represent S.E.M. The averages of nine different fields of view were calculated for

each animal (counts from three fields of three sagittal slices). (c)Panoramic view of the frontal cortex in a coronal mouse brain section at P56, showing double immunofluorescence labeling for GFAP (green) and Tuj1 (red), inset shows a coronal map of the area. Both sides of the brain were similar. (d) Double immunofluorescence staining. NeuN staining (red immunofluorescence), Caspase-3 staining (green immunofluorescence), and DAPI staining (blue fluorescence). Unpaired two-tailed t-test. Error bars represent S.E.M. The averages of nine different fields of view were calculated for each animal (counts from three fields of three sagittal slices). (e) TUNEL staining (green immunofluorescence), and DAPI staining (blue fluorescence). Unpaired two-tailed t-test. Error bars represent S.E.M. Experiments were repeated nine times (three biological and three analytical repeats). (f) CD31 staining (red immunofluorescence) and blue fluorescence DAPI (Unpaired Two-Tailed *t*-test). Error bars represent S.E.M. The averages of nine different fields of view were calculated for each animal (counts from three fields of three sagittal slices). Quantification of CD31 expression by immunofluorescence.

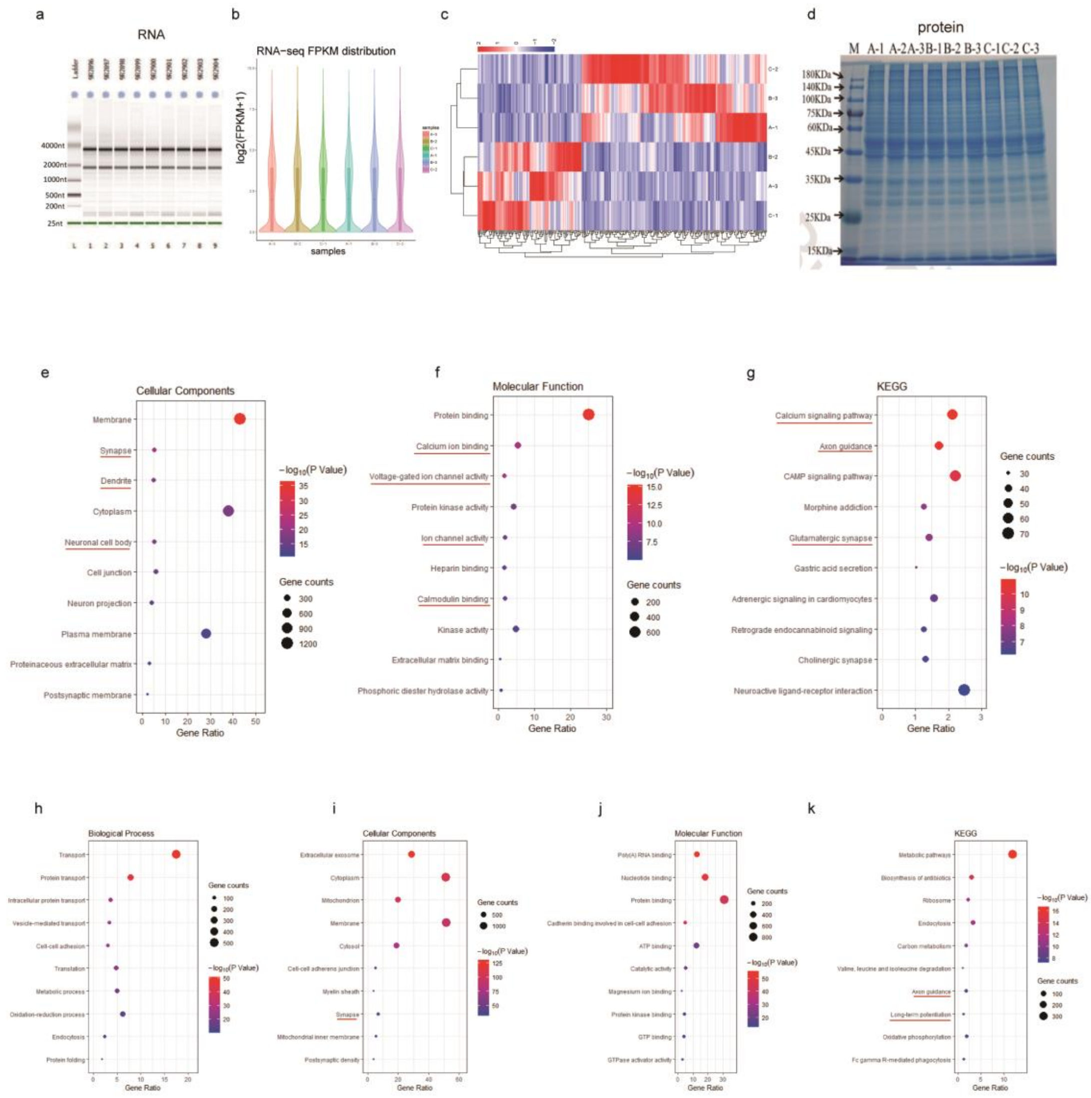

Fig. S3. Integrated bioinformatics analysis to identify mRNA targets of miR-592. (a) RNA electrophoresis image of the half brain cortical tissues homogenized in the Trizol buffer before RNA-seq analysis. (b) FPKM from RNA-seq is the means of WT and miR-592$^{-/-}$ mice in three replicates. (c) The heatmap was constructed based on FPKM values obtained by RNA-Seq data. (d) SDS-PAGE and Coomassie staining of another half brain cortical tissues homogenized in the lysis buffer before protein digestion for the proteomics analysis. (e-g) GO analysis of DEGs from RNA-seq data. A bubble chart was showing the terms of (e) cellular components, (f) molecular function, and (g) KEGG. (h-k) GO analysis of DEGs from proteomics analysis data. A bubble chart showed the terms of (h) biological process, (i) cellular components, (j) molecular function, and (k) KEGG.

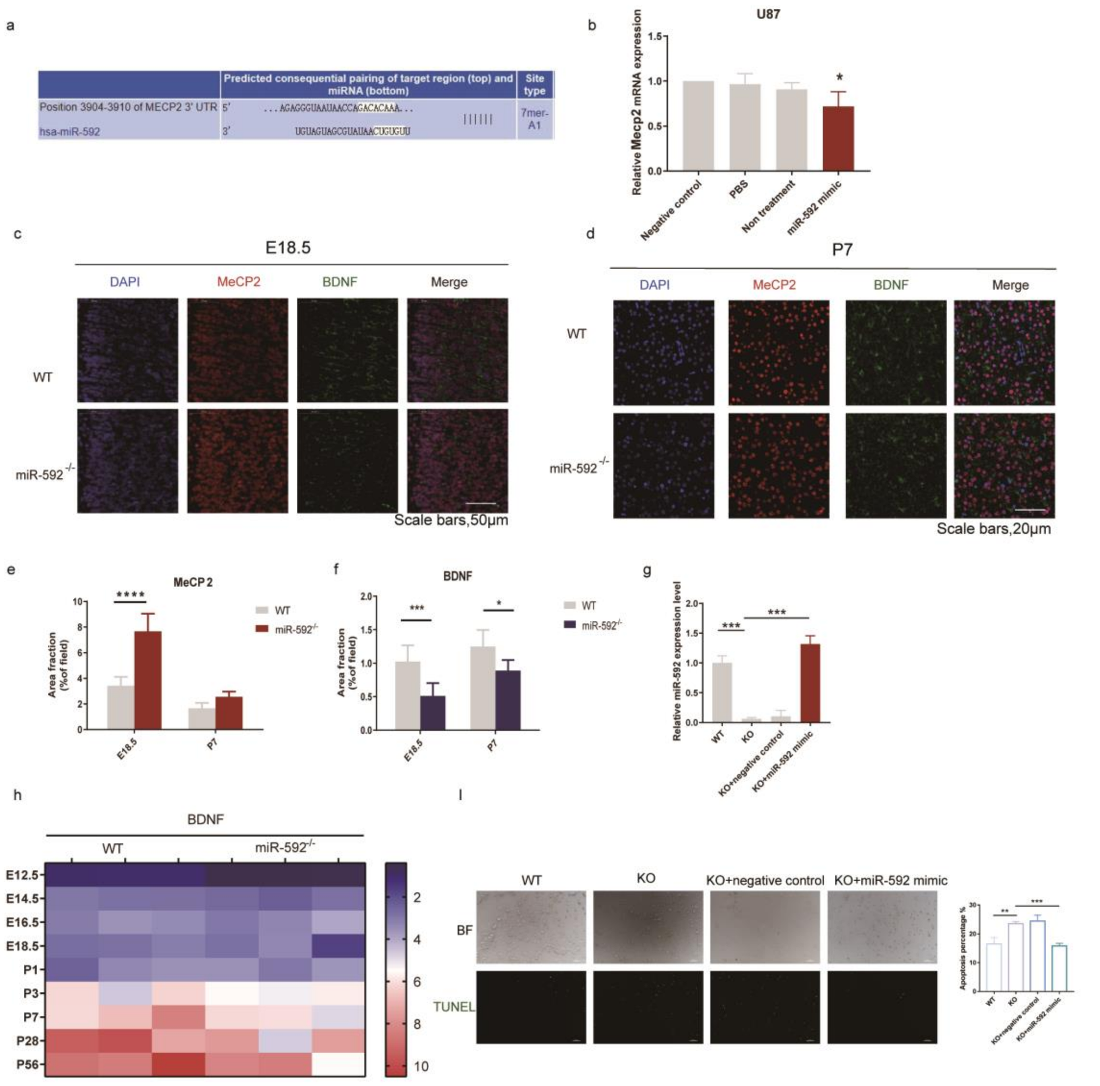

Fig. S4. MeCP2 is a target gene of miR-592 and MeCP2–BDNF axis regulated by miR-592 during development. (a, b) (a) Seed region of has-miR-592 in the 3′ UTR region of human MeCP2. (b) RT-PCR, has-miR-592 regulated MeCP2 in cultured U87 human glioblastoma cells (Unpaired Two-Tailed *t*-test.) Error bars represent S.E.M. Experiments were repeated nine times (three biological and three analytical repeats). (c, d) (c) MeCP2–BDNF axis was regulated by miR-592 in the cortex at E18.5 (One-way ANOVA with Bonferroni post hoc test, n = 3 mice for each genotype). (d) Quantification of MeCP2 and BDNF expression by immunofluorescence at E18.5. (e, f) (e) MeCP2–BDNF axis was regulated by miR-592 in the cortex at P7 (One-way ANOVA with Bonferroni post hoc test). Error bars represent S.E.M. The averages of nine different fields of view were calculated for each animal (counts from three fields of three sagittal slices). (f) Quantification of MeCP2 and BDNF expression by

immunofluorescence at E18.5. (g) RT-PCR analysis validation of increased miR-592 expression after transfection with miR-592 mimics (Unpaired Two-Tailed *t*-test). Experiments were repeated nine times (three biological and three analytical repeats). (h) miR-592 knockout resulted in the decreased expression of BDNF during development (n = 3). (I) TUNEL staining and TUNEL - positive cells rate. TUNEL staining (green immunofluorescence). One-way ANOVA with Tukeys multiple comparison test. Experiments were repeated nine times (three biological and three analytical repeats). BF, Bright field.

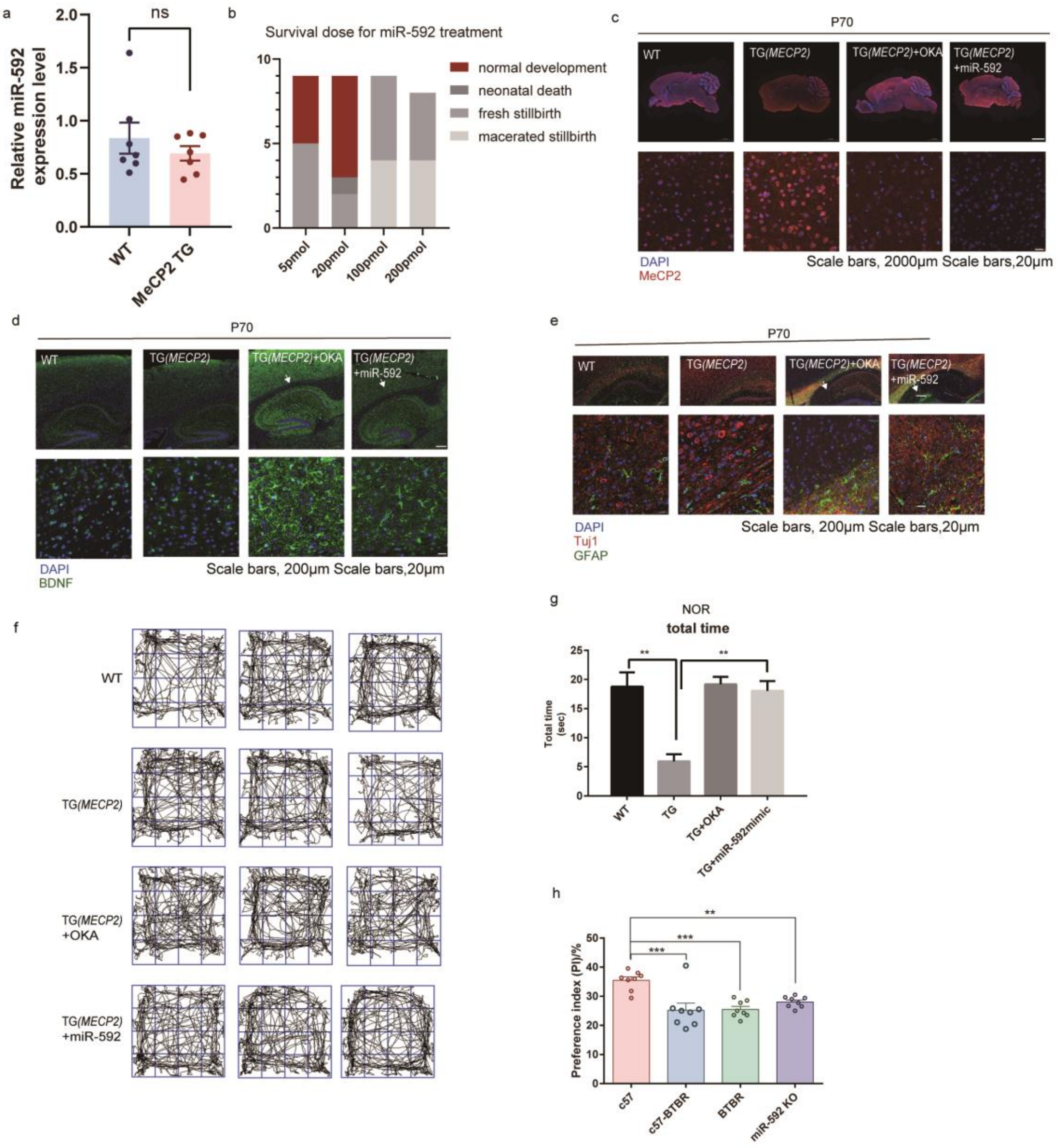

Fig. S5. Reversal of phenotypes in MeCP2 duplication mice using miR-592. (a) RT-PCR. miR-592 expression levels in the cortices of WT and Tg(*MECP2*) mice were examined by RT-PCR (unpaired two-tailed t-test). Experiments were repeated six times (three biological and two analytical repeats). (b) Survival dose for miR-592

treatment. Tg(*MECP2*) mice were lipofected with miR-592, randomized to ⩾8 mice per group and administered 5pmol, 20pmol, 100pmol, and 200pmol dose. (c)Immunofluorescence shows the MeCP2 (red immunofluorescence) response to miR-592 LNA mimic treatment at P70. (d)Immunofluorescence shows the BDNF (green immunofluorescence) response to miR-592 LNA mimic treatment at P70. (e) Immunofluorescence shows the GFAP (green immunofluorescence) and Tuj1 staining (green immunofluorescence) response to miR-592 LNA mimic treatment at P70. DAPI, blue fluorescence marking the cell nuclei. (f) Open field test. Representative images show typical examples of exploring the behavior of WT and Tg(*MECP2*) mice during the 10 min of the open field test. Activity behavior of 70-day-old mice in the open field test 11 weeks after OKA and miR-592 LNA mimic treatment. (g) Novel object preference test. The curiosity behaviour of 70-day-old WT and Tg(*MECP2*) mice (12 weeks after OKA and miR-592 mimic treatment) was tested (one-way ANOVA with Tukeys multiple comparison test, n = 8, error bars represent S.E.M). (h) Novel object preference test. Preference index (PI) for each mouse was determined by dividing the amount of time spent exploring the novel object by the total amount of time spent exploring both objects (PI = 100 * (Tnovel/Tnovel + Tfamiliar). (one-way ANOVA with Tukeys multiple comparison test, n = 8, error bars represent S.E.M).
