# Supplementary material for: Loss of neurodevelopmental-associated miR-592 impairs neurogenesis and causes social interaction deficits
Source: Cell Death Dis. 2022 Apr 1;13(4):292. doi: 10.1038/s41419-022-04721-z (PMC8976077; doi:10.1038/s41419-022-04721-z)
Supplement: Supplementary file 2 — Attribution of Authorship [file 41419_2022_4721_MOESM2_ESM.pdf]

DECLARATION OF CONTRIBUTIONS TO ARTICLE

ADMC

Manuscript Number: CDDIS-21-2362R

Journal Name: *Cell Death & Disease* (the 'Journal')

Proposed Title of the Contribution:

Jing Zhang, Jun Zhang, Yang Zhou mainly designed and led the process of the project. Yu Fu performed most of the experiments. Yuan-lin Zhang, Bo Zhao, Xing-Liao Zhang, Yi-jun Lu treated data. Yu Fu, Aiping Lu, and Wan-ting Zhang edited the content of the manuscript. (the 'Contribution)

Author(s):

Yu Fu, Yuan-lin Zhang, Yang Zhou, Bo Zhao, Xin-Liao Zhang, Wan-ting Zhang, Yi-jun Lu, Aiping Lu, Jun Zhang and Jing Zhang (the 'Authors')

For all *CDDis* articles, each person named as an author in the published version must be able to show he or she has contributed substantially to the article.

Authorship credit should be based on 1) substantial contributions to conception and design, acquisition of data, or analysis and interpretation of data; 2) drafting the article or revising it critically for important intellectual content; and 3) final approval of the version to be published. Authors should meet conditions 1, 2 and 3.

Any person who cannot be shown to have made a substantial contribution to the article cannot be listed as an author in the final version. The name of any person who is deemed to have made a minor contribution can, however, appear in the Acknowledgments section of the article.

Please complete the table below to indicate the contributions of all named authors to the manuscript.

Author Full Name:	Specification of Contribution to the Manuscript:
Jing Zhang, Jun Zhang, Yang Zhou and Yu Fu	mainly designed and led the process of the project
Dr. Chen J，Yu Fu	designed the Cas9 system and sgRNAs
Yu Fu, Yuan-lin Zhang, and Wan-ting Zhang	mainly performed the behavioral experiments
Xin-liao Zhang and Yi-jun Lu	mainly performed histological tests
Yu Fu, Bo Zhao, and Yuan-lian Zhang	mainly performed the molecular biology assays.
Yu Fu and Yuan-lian Zhang	mainly performed the lipofection
Bo Zhao and Yu Fu	did the work of data analysis
Yu Fu, Aiping Lu，and Wan-ting Zhang	edited the content of the manuscript

Please complete the table below to indicate the contributions of all named authors to the figures.

Figure 1:

Yu Fu contributed to Fig1a. Yang Zhou contributed to Fig1b. Yuan-lin Zhang contributed to Fig1c. Yu Fu, Jun Zhang and Jing Zhang contributed to Fig1e-f. Yuan-lin Zhang contributed to Fig1g. Yu Fu contributed to Fig1h. Yu Fu and Jing Zhang contributed to Fig1i. Yu Fu and Yang Zhou contributed to Fig1j.

Figure 2:

Jun Zhang, Jing Zhang, Yu Fu contributed to Fig2a and b. Fu, Yang Zhou, Xing-liao Zhang and Yi-jun Lu contributed to Fig2c-h.

Figure 3:

Yu Fu contributed to Fig3a. Yu Fu and Yuan-lin Zhang contributed to Fig3b-m. Yu Fu contributed to Fig3n. Yuan-lin Zhang, Wan-ting Zhang and Yu Fu contributed to Fig3o-r.

Figure 4:

Yu Fu contributed to Fig4a. Yu Fu and Yang Zhou contributed to Fig4b-d. Yu Fu and Bo Zhao contributed to Fig4e and f. Jun Zhang, Jing Zhang, Yu Fu contributed to Fig4g-i.

Figure 5:

Yu Fu contributed to Fig5a-c. Xing-liao Zhang and Yi-jun Lu contributed to Fig5d. Yu Fu and Bo Zhao contributed to Fig4f and j. Yuan-lin Zhang contributed to Fig5k. Jun Zhang and Jing Zhang and Yu Fu contributed to Fig5l.

Figure 6:

Yu Fu contributed to Fig6a-c. Jun Zhang, Jing Zhang, Xing-liao Zhang and Yi-jun Lu contributed to Fig6d-k. Yu Fu contributed to Fig6l. Yuan-lin Zhang and Wan-ting Zhang contributed to Fig5m-o.

Signed for and on behalf of the Author(s):	Print Name:	Date:
[illegible]	Yu Fu, Yuan-lin Zhang, Bo Zhao, Xin-Liao Zhang, Wan-ting Zhang,Jun Zhang and Jing Zhang	June 17, 2021
