# Supplementary figures and images for: Loss of neurodevelopmental-associated miR-592 impairs neurogenesis and causes social interaction deficits
Source: Cell Death Dis. 2022 Apr 1;13(4):292. doi: 10.1038/s41419-022-04721-z (PMC8976077; doi:10.1038/s41419-022-04721-z)

MeCP2

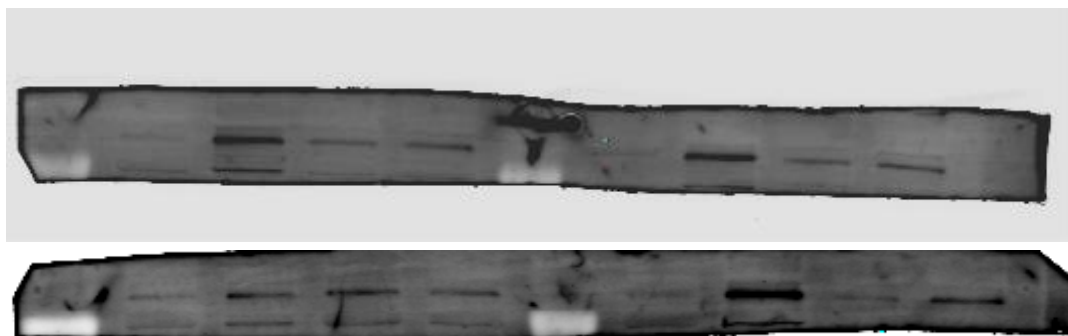

BDNF

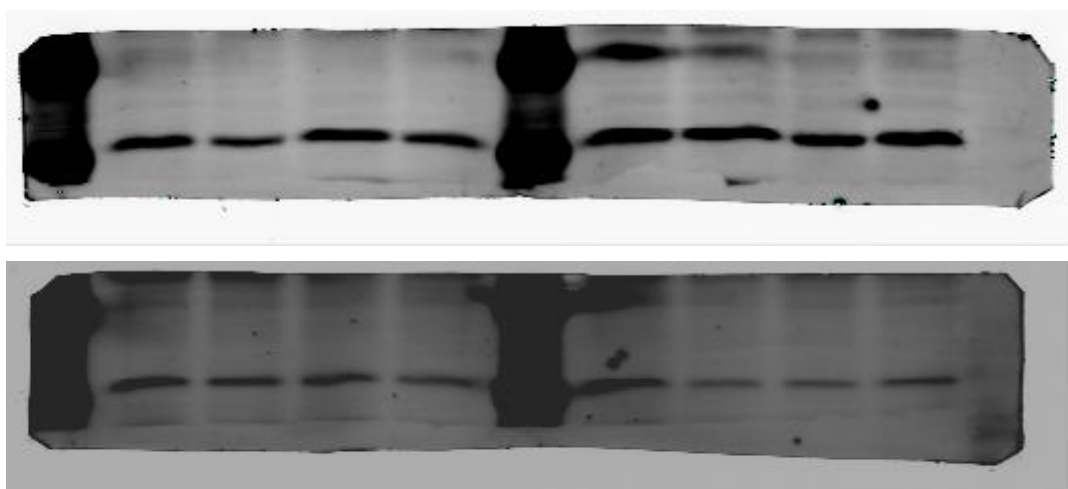

AKT

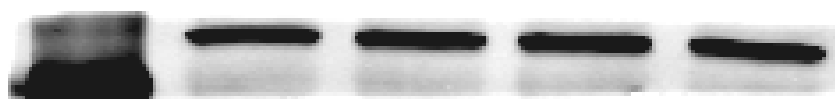

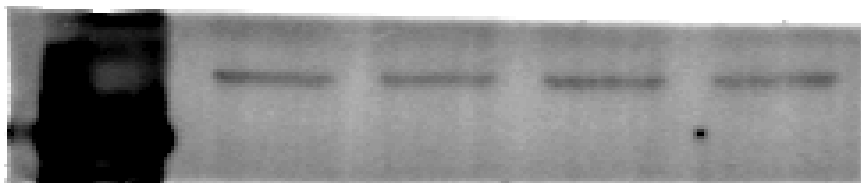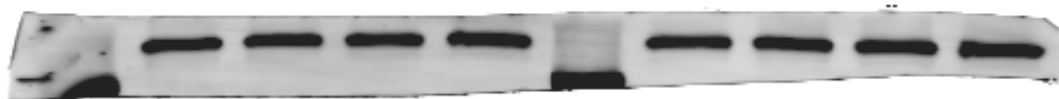

p-AKT

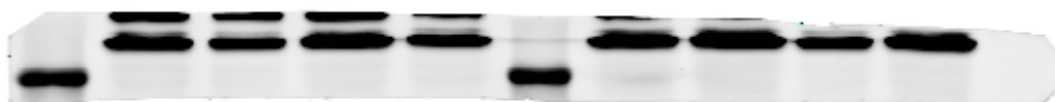

GAPDH

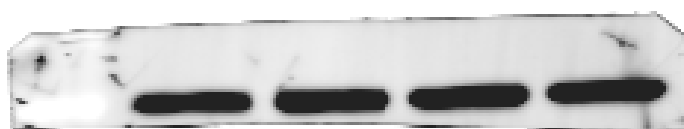

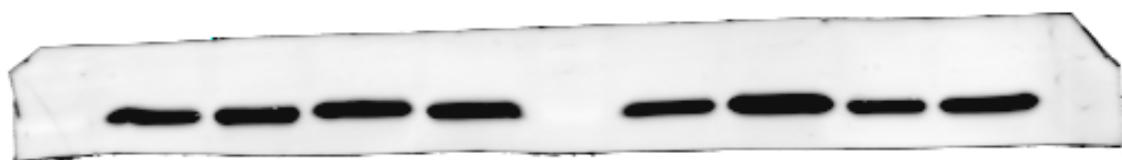

Supplement: Supplementary file 4 — Original Data File [file 41419_2022_4721_MOESM4_ESM.pdf]
