# Supplementary material for: Loss of neurodevelopmental-associated miR-592 impairs neurogenesis and causes social interaction deficits
Source: Cell Death Dis. 2022 Apr 1;13(4):292. doi: 10.1038/s41419-022-04721-z (PMC8976077; doi:10.1038/s41419-022-04721-z)
Supplement: Supplementary file 5 — Supplymentary table [file 41419_2022_4721_MOESM5_ESM.docx]

| Table2. Gene list | | | | |
| --- | --- | --- | --- | --- |
| Predicted targetgene | Autism gene | RNA-seq | TMT-based quantitative proteimic analysis | Intersection |
| GM2007 | ABCA10 | ZBTB20 | TUBB2A | AGAP2 |
| GM11007 | ABCA13 | NNAT | GAPDH | VLDLR |
| PROKR2 | ABCA7 | LCT | ACTG1 | PSMD12 |
| E130309F12RIK | ACE | NRP2 | SPTAN1 | GPD2 |
| PYGO1 | ACHE | NR3C2 | CLTC | PAFAH1B1 |
| DRD5 | ADA | IL16 | ATP1A3 | NRXN1 |
| PAQR9 | ADARB1 | PCDH19 | SPTBN1 | SH3KBP1 |
| ISCA1 | ADCY3 | NR2F2 | DPYSL2 | MTOR |
| ELK4 | ADCY5 | GM2115 | DYNC1H1 | UBE2H |
| TRPM3 | ADK | FAT4 | DNM1 | SCN9A |
| GM3183 | ADNP | CAPN11 | ENO1 | USP15 |
| CLEC4N | ADORA3 | GPR161 | PLEC | ANK3 |
| SKINT3 | ADSL | GPR101 | ATP5F1B | ANKS1B |
| CACNG1 | AFF2 | EPHA6 | HSPA8 | BRINP1 |
| BEST3 | AFF4 | SLIT1 | STXBP1 | STAT1 |
| AK157302 | AGAP1 | NPY2R | ANK2 | CADM2 |
| GM6970 | AGAP2 | ST6GALNAC5 | PKM | PREX1 |
| ONECUT2 | AGBL4 | PPP1R1B | CKB | PLCB1 |
| PDS5B | AGMO | ABCB8 | SLC1A2 | GABRB3 |
| ARHGAP30 | AGO1 | PROX1 | HK1 | HSD11B1 |
| CDH5 | AGTR2 | NOS1 | MAP1A | BIRC6 |
| B230216G23RIK | AHDC1 | THSD4 | ATP5F1A | FAM19A2 |
| TES | AHI1 | DNAH9 | ATP6V1A | SYN3 |
| HTT | AKAP9 | NRP1 | CAMK2A | EXOC6 |
| KCNA2 | ALDH1A3 | TRPM3 | PPP3CA | NRCAM |
| KCNJ3 | ALDH5A1 | NTNG1 | MAP2 | RAB2A |
| TBC1D13 | AMPD1 | CAMK2N1 | AP2B1 | ELAVL3 |
| GM14305 | AMT | SLC44A5 | ATP1A1 | CSNK1D |
| ANGPTL2 | ANK2 | NR4A3 | HSP90AB1 | OGT |
| AGAP2 | ANK3 | PCSK2 | NSF | SYNCRIP |
| GNGT2 | ANKRD11 | CNTNAP5C | ATP2B2 | RFX3 |
| GM10030 | ANXA1 | CACHD1 | MDH2 | NEFL |
| PDE4D | AP1S2 | NKAIN3 | VDAC1 | NRXN3 |
| BLVRB | APBA2 | SIPA1L3 | ACO2 | ACTN4 |
| WBP2 | APBB1 | FAM19A2 | GNB1 | CADM1 |
| ZFP266 | APC | KCTD12 | ALDOA | GIGYF2 |
| GM20826 | APH1A | VXN | YWHAE | SLC1A2 |
| NOD2 | ARHGAP15 | AGT | TUBB4B | PACS2 |
| IL1RAP | ARHGAP24 | IQGAP2 | BSN | SUCLG2 |
| TBL1X | ARHGAP32 | DOC2B | SYN1 | CAMK4 |
| PNRC1 | ARHGAP33 | NAV1 | ENO2 | ANK2 |
| OLFR613 | ARHGAP5 | TNFAIP8L3 | ATP6V1B2 | DPP10 |
| JAM2 | ARHGEF10 | SGK1 | ATP1A2 | NLGN3 |
| VLDLR | ARHGEF9 | MBP | ACTB | CACNA2D1 |
| 1500009L16RIK | ARID1B | 4833420G17RIK | PGK1 | SNAP25 |
| ERG | ARNT2 | CALB2 | HSPD1 | GRM5 |
| GM14288 | ARX | DOCK10 | CALM3 | UNC13A |
| PSMD12 | ABAT | KL | AP2A1 | FMR1 |
| TAC4 | ACTN4 | GRIA1 | CNP | SMARCC2 |
| TRIML2 | ACY1 | TMEM255A | PLP1 | NTRK3 |
| GPD2 | ADAMTS18 | SCN3B | MYH10 | DIP2A |
| WDR5B | ADORA2A | CPNE6 | CNTN1 | GRIK3 |
| GM9821 | ADRB2 | RAVER2 | NCAM1 | DOCK4 |
| ZSCAN22 | ALG6 | HTR2A | DMXL2 | CDH11 |
| 4930447A16RIK | ALOX5AP | ARPP19 | PCLO | HERC2 |
| K230010J24RIK | ANKS1B | PTGDS | MAP1B | IL1RAPL1 |
| UTY | ARHGAP11B | SLC13A4 | SLC25A4 | EIF4E |
| GM14434 | ASAP2 | HAP1 | PPIA | DNAH17 |
| PRL7C1 | ASH1L | PRLR | YWHAZ | SHANK1 |
| SGK2 | ASMT | GABRA5 | PFKM | NTRK2 |
| GM14419 | ASPM | EPHB6 | CRMP1 | BRAF |
| GM4724 | ASTN2 | RYR3 | HIST1H2BH | SRGAP3 |
| F830045P16RIK | AMBRA1 | RELN | SYNGAP1 | SMC3 |
| DDX20 | APP | MARCKSL1 | ACTN1 | GRM7 |
| XK | AR | TSPAN18 | GLUL | DLG1 |
| GM21244 | ASS1 | RASD2 | PYGB | GRIN2B |
| H2-Q4 | ASXL3 | FIBCD1 | SYT1 | LRRC7 |
| NMB | ATG7 | ACVR1C | GNAO1 | KIF21B |
| SGCG | ATP10A | UNC5C | FASN | UBE3B |
| HNRNPF | ATP1A1 | CAMK2D | GOT1 | GABRB1 |
| 2210418O10RIK | ATP1A3 | 9930022D16RIK | GPI | PCDH19 |
| GM6710 | ATP2B2 | RGS4 | UBA1 | INPP1 |
| GM14296 | ATP6V0A2 | TRHR | MAP6 | CELF4 |
| GM1070 | ATP8A1 | DPYD | MYO5A | HOMER1 |
| BC021891 | ATRNL1 | KMT2A | 7-Sep | CNTNAP2 |
| HIST1H2BR | ATRX | TMEM72 | MACF1 | TANC2 |
| SNRK | ATXN7 | PPFIA2 | MBP | GDA |
| GM4245 | AUTS2 | SYT6 | NEFM | CNR1 |
| TECPR2 | AVP | SLC6A20A | VCP | RAB39B |
| CLDN22 | AVPR1A | RFX3 | CKMT1 | MECP2 |
| LY6G6C | AVPR1B | SEMA5A | BIN1 | IRF2BPL |
| ICAM1 | AZGP1 | CBLN2 | ATP2A2 | TOMM20 |
| TACR2 | BAIAP2 | DSP | GOT2 | GRIN2A |
| GM13889 | BAZ2B | TENM1 | TPI1 | CNTN4 |
| TRIP13 | BBS4 | GALNT17 | SNAP91 | SLC27A4 |
| SLC5A7 | BCKDK | CPNE2 | NDUFS1 | SMAD4 |
| MYO10 | BCL11A | DRD2 | ATP6V1E1 | MDGA2 |
| HOXA5 | BCL2 | MOBP | SYN2 | EXOC6B |
| P2RY10 | BDNF | UST | UQCRC2 | RPS6KA2 |
| MAP3K6 | BIRC6 | ZFP618 | CS | CSMD1 |
| GM14403 | BRAF | ARHGEF28 | GLUD1 | PTPRT |
| FAM150A | BRCA2 | HTR4 | EEF2 | SCN1A |
| PSD2 | BRD4 | TGFB2 | IDH3A | OCRL |
| GM17093 | BRINP1 | NGEF | ATP6V0A1 | MAPK1 |
| LRRC4C | BST1 | DOCK4 | TNR | MARK1 |
| UBA1Y | BTAF1 | SPARC | LRP1 | SYNJ1 |
| CENPL | C12orf57 | OSBPL1A | ATP1B1 | CLASP1 |
| MTRF1L | C15orf62 | MTSS1L | MDH1 | PTEN |
| PLCB4 | C3orf58 | PCDH8 | SNAP25 | CAPRIN1 |
| RGS13 | C4B | IFIT1 | GPD2 | ARHGAP5 |
| BC003965 | CA6 | ADORA2A | TLN2 | FOXP1 |
| PPIH | CACNA1A | NHSL1 | PFKP | NAA15 |
| GM11568 | CACNA1C | PTPRZ1 | HSP90AA1 | NIPA1 |
| CMTM5 | BICDL1 | AHCYL2 | HSPA9 | TRIM33 |
| RIMKLA | CACNA1D | ANO3 | IMMT | ELP4 |
| TFG | CACNA1E | SHISA6 | SRCIN1 | PLXNA4 |
| RASL12 | CACNA1F | KSR1 | PLCB1 | CACNA1B |
| SAMD4 | CACNA1G | NDST4 | NAPB | CUL3 |
| H1FOO | CACNA1H | CRLF1 | GDI1 | NOS1 |
| ILF3 | CACNA1I | COL6A1 | HSPA4 | EPHB2 |
| GM9938 | CACNA2D3 | LSAMP | SLC25A12 | SBF1 |
| PDE4D | CACNB2 | EIF2S3Y | VDAC2 | CHD7 |
| PPFIBP1 | CADM1 | MYO5B | ALDOC | RIMS3 |
| PRKAB2 | CADM2 | OGN | LDHB | DEPDC5 |
| ANGPTL7 | CADPS2 | GNAL | CAND1 | PRICKLE1 |
| LCA5L | CAMK2A | MAML2 | SYNJ1 | MPP6 |
| PRCP | CAMK2B | DCN | TUBB3 | APP |
| TSC22D1 | CAMK4 | HPCAL1 | LDHA | PTBP2 |
| PAPLN | CAMSAP2 | ARHGAP6 | GPM6A | MYO5A |
| SSH2 | CAMTA1 | EPHA5 | SLC25A3 | PIK3R2 |
| PPM1A | CAPN12 | PLA2G2F | RYR2 | TSN |
| G430049J08RIK | CAPRIN1 | FGFR1 | DLD | ASTN2 |
| MYO3B | CARD11 | CUX2 | INA | EEF1A2 |
| OSM | CASC4 | VGLL3 | SH3GL2 | SOD1 |
| 4930556J24RIK | CASK | RGS9 | LRPPRC | DLGAP2 |
| CES1G | CBLN1 | MEF2C | WDR7 | HCN1 |
| ZC3H7A | CC2D1A | IPCEF1 | PGAM1 | SCP2 |
| RD3 | CCDC88C | SLC9A2 | OXCT1 | DNM1L |
| MRPL3 | CCDC91 | ARHGAP25 | ATP2B1 | SMARCA4 |
| NKIRAS2 | CCT4 | CPLX3 | CAMK2B | DPP6 |
| ADAM25 | CD276 | HECTD2 | CADPS | UBA6 |
| GM11110 | CD38 | PKP2 | SLC12A5 | ATP8A1 |
| CSNK2A2 | CD44 | PIP5K1B | OPA1 |  |
| 4932411N23RIK | CD99L2 | AW551984 | AP2M1 |  |
| CPXCR1 | CDC42BPB | SIPA1L2 | EPB41L3 |  |
| TTLL7 | CDH10 | PDP1 | YWHAH |  |
| SUV420H1 | CDH11 | CRTAC1 | DNM1L |  |
| SPRR2A1 | CDH22 | DOCK9 | SDHA |  |
| FLT4 | CDH8 | PDE1B | ALB |  |
| CRY2 | BCAS1 | ADCY8 | ITPR1 |  |
| INTU | BIN1 | LAMP5 | HSPH1 |  |
| SH3YL1 | CACNA1B | ZFP131 | PRDX5 |  |
| GM14137 | CACNA2D1 | CACNA1E | OGDH |  |
| CDV3 | CBS | JUN | AMPH |  |
| GNA12 | CCNG1 | EPHA7 | 5-Sep |  |
| PAFAH1B1 | CCNK | PCDH20 | ANK3 |  |
| ZBTB14 | CDH13 | RASGRF1 | DCTN1 |  |
| MOG | CDH9 | ARHGAP36 | AHCYL1 |  |
| FAM47E | CDK13 | COL25A1 | DLAT |  |
| ZFP598 | CDKL5 | RCAN2 | STX1B |  |
| CLVS1 | CDKN1B | FGD4 | VSNL1 |  |
| PARD3B | CECR2 | MEDAG | CYFIP2 |  |
| CLEC2L | CELF4 | KLF3 | PPP2R1A | |
| PHGR1 | CELF6 | OVOL2 | ACLY |  |
| CLCC1 | CEP135 | RREB1 | HBB-B1 |  |
| ZFP352 | CEP290 | KCNG2 | NEFL |  |
| GM14431 | CEP41 | NRGN | SPTB |  |
| CNTNAP5C | CGNL1 | ST18 | VDAC3 |  |
| GM14412 | CHD1 | TPPP3 | DLG4 |  |
| ANKMY2 | CHD2 | SH3RF2 | NFASC |  |
| 4933402D24RIK | CHD5 | KCNA1 | TUBB4A |  |
| CRABP2 | CHD7 | DSCAML1 | GNB2 |  |
| ZFP874A | CHD8 | GALNT15 | STIP1 |  |
| NRXN1 | CHKB | ENAH | PACSIN1 |  |
| D15ERTD621E | CHMP1A | GLIS3 | BASP1 |  |
| 4632428N05RIK | CHRM3 | FRZB | NDRG2 |  |
| DOS | CHRNA7 | KCTD4 | CCT7 |  |
| TLR2 | CHRNB3 | TIAM1 | ACSL6 |  |
| CALD1 | CHST5 | TRPS1 | PI4KA |  |
| LRRC59 | CIB2 | HOMER3 | NCKAP1 |  |
| 4922502D21RIK | CIC | TM6SF2 | RAB3A |  |
| VSNL1 | CLASP1 | SLIT3 | HSPA5 |  |
| GPSM3 | CLN8 | DLG5 | PDHA1 |  |
| NEUROD2 | CLSTN2 | VPS13C | FH |  |
| GM13247 | CLSTN3 | TRPC4 | CLASP2 |  |
| GNS | CLTCL1 | RNF213 | SHANK1 |  |
| 5730507C01RIK | CMIP | ADAMTS9 | MTCO2 |  |
| MED19 | CNGB3 | FGF13 | GRIA2 |  |
| NPR2 | CNKSR2 | MDGA1 | CCT3 |  |
| GM21379 | CNOT3 | KIF26B | HSPA12A | |
| GM14410 | CNR1 | ARHGAP12 | AP2A2 |  |
| NR3C2 | CNR2 | GPR158 | CFL1 |  |
| PDZD9 | CNTN4 | FSCN1 | KIF5C |  |
| QRFP | CNTN5 | PRKCA | PRKCB |  |
| SUMF2 | CNTN6 | ITPR1 | OXR1 |  |
| TMED5 | CNTNAP2 | SLFN5 | PRDX6 |  |
| GM14322 | CNTNAP4 | GM11549 | HNRNPA2B1 | |
| CDH19 | CNTNAP5 | CRYM | BAIAP2 |  |
| RRBP1 | COL28A1 | SLC4A7 | UQCRC1 |  |
| GM4631 | CPT2 | FAU | MYO18A |  |
| GM14418 | CREBBP | BVES | NCL |  |
| ALDH1L2 | CHD3 | PHACTR2 | GSTM1 |  |
| SH3RF2 | CNTN3 | CPNE9 | ANXA6 |  |
| A1BG | CNTNAP3 | SEMA6D | EEF1A2 |  |
| CD8A | CRHR2 | PRDM5 | CCT2 |  |
| TM6SF1 | CSMD1 | ITGA4 | CTNNA2 |  |
| RQCD1 | CSNK1D | HOMER2 | ATP6V1H | |
| SH3KBP1 | CSNK1E | BUB1B | MYH9 |  |
| 9630041A04RIK | CTCF | DISP2 | CYC1 |  |
| CMPK2 | CTNNA3 | AEBP1 | GDA |  |
| CSF2RA | CTNNB1 | AK5 | CCT8 |  |
| SMPD4 | CTNND2 | CD4 | ABAT |  |
| CDX4 | CTTNBP2 | PLXNA4 | HNRNPU | |
| WSB1 | CUL3 | SRRM2 | UCHL1 |  |
| FZD6 | CPEB4 | GM19410 | FSCN1 |  |
| LPAR5 | CTNNA2 | SLC4A5 | TOMM70 | |
| POLA2 | CUL7 | SMOC1 | PDHB |  |
| GM14308 | CUX1 | SYTL2 | NBEA |  |
| GM16513 | CUX2 | SLC39A10 | EPB41L1 |  |
| PRELID2 | CX3CR1 | FOSL2 | GLS |  |
| GM14399 | CXCR3 | NEU2 | HNRNPK | |
| ZFP810 | CYFIP1 | GM11639 | GPRIN1 |  |
| PGGT1B | CYLC2 | AKAP13 | TUFM |  |
| TVP23A | CYP11B1 | C1QL2 | RAB1B |  |
| CCNDBP1 | CYP27A1 | DNER | ACTR2 |  |
| KPNA3 | DAB1 | IGFBP6 | HADHA |  |
| 4930426L09RIK | DAGLA | NEBL | SLC8A2 |  |
| GM10643 | DAPK1 | BCAS1 | ALDH1L1 | |
| ARHGAP40 | DAPP1 | DOCK11 | NCDN |  |
| SERPINB11 | DCTN5 | F5 | TCP1 |  |
| OLFR157 | DDX3X | AQP4 | RPH3A |  |
| ITGB6 | DDX53 | CHRM5 | PRKCG |  |
| 9530002B09RIK | DEAF1 | NCDN | VCL |  |
| GM6505 | DENR | MT-ND5 | NDUFS2 |  |
| AIPL1 | DEPDC5 | VWC2L | CKAP5 |  |
| CHST1 | DHCR7 | RASGEF1B | NCAM2 |  |
| LHX9 | DHX30 | GABRB1 | PDIA3 |  |
| GM10384 | DIAPH3 | DNAJC2 | HSPA4L |  |
| SLMO2 | DIP2A | NEK10 | PPFIA3 |  |
| ZFP185 | DIP2C | LDHB | ROCK2 |  |
| STK32B | DISC1 | ACVR2A | USP9X |  |
| MBNL1 | DIXDC1 | KRT2 | PC |  |
| ZBTB2 | DLG1 | CALM1 | PPP3R1 |  |
| CSF2RB2 | DLG4 | LRRC4C | YWHAB |  |
| RAB7 | DLGAP1 | ARHGEF6 | PYGM |  |
| SPN | DLGAP2 | ATP1A1 | CALR |  |
| CUTAL | DLX6 | EFHD2 | TKT |  |
| NSA2 | DMD | SCN4B | CCT5 |  |
| EMD | DCX | RTL3 | DPYSL3 |  |
| NFIA | DGKZ | ATF3 | KIF21A |  |
| CD300LB | DMPK | GRIK1 | HBA |  |
| ZFP957 | DMXL2 | THSD7B | STMN1 |  |
| CCDC126 | DNAH10 | PDE7B | USP5 |  |
| CD84 | DNAH17 | CDH7 | ACTC1 |  |
| RBM47 | DNAH3 | MAP1A | DNAJC6 |  |
| JRKL | DNER | GUCY1A1 | NRCAM |  |
| TEX101 | DNM1L | OLFM4 | 11-Sep |  |
| CYP3A13 | DNMT3A | ALDH1A2 | ATP5O |  |
| TFDP1 | DOCK1 | ZDBF2 | DPYSL5 |  |
| ANKRD29 | DOCK10 | FHOD3 | HOMER1 | |
| LAMB3 | DOCK4 | GPR88 | EIF4A2 |  |
| LONRF3 | DOCK8 | CABP7 | MAPT |  |
| LRRC14 | DPP10 | DPP6 | NEFH |  |
| MAGOH | DPP4 | CLDN2 | PRKAR2B | |
| DOCK11 | DPP6 | CBL | HIST1H4A | |
| ASB17 | DCUN1D1 | FREM3 | TUBA4A |  |
| PLXDC2 | DDC | LMO4 | ERC2 |  |
| MAP3K7 | DDX11 | ZCCHC12 | AARS |  |
| MAPK10 | DGKK | DSC3 | RTN3 |  |
| CPSF1 | DLGAP3 | PLEKHB1 | PALM |  |
| CCDC115 | DLX1 | EDNRB | WDR1 |  |
| HIST1H2BJ | DLX2 | IGSF1 | GPD1 |  |
| GM4847 | DNAJC19 | FIBIN | CAP1 |  |
| IGFBP1 | DOLK | FSTL5 | SLC1A3 |  |
| MTOR | DPYD | ARHGEF26 | ACTN4 |  |
| MVK | DPYSL2 | CARMIL3 | CYCS |  |
| 2510009E07RIK | DPYSL3 | COX8A | PFN1 |  |
| ENPP4 | DRD1 | SPEN | MAPK1 |  |
| GBP6 | DRD2 | DPP10 | TPP2 |  |
| RIC8B | DRD3 | PPP1R9A | MATR3 |  |
| PEX26 | DSCAM | EXPH5 | SGIP1 |  |
| OSTM1 | DST | STPG1 | TAGLN3 |  |
| GM21092 | DUSP15 | PTPN5 | PTPRZ1 |  |
| CLN8 | DUSP22 | CLMN | PHB2 |  |
| MPV17L | DVL1 | NTS | GNAI2 |  |
| PLEKHA5 | DVL3 | CPNE5 | CASKIN1 | |
| LANCL3 | DYDC1 | CTBP2 | SYNE1 |  |
| AASDHPPT | DYDC2 | PPFIA4 | SIRT2 |  |
| FAM117A | DYNC1H1 | RGS20 | NDUFV1 |  |
| PPP1R12C | DYRK1A | TMEM132A | RTN4 |  |
| 3110007F17RIK | EBF3 | TOX | PGM1 |  |
| MTF2 | EEF1A2 | SLC29A4 | DCLK1 |  |
| PCDHB10 | EFR3A | MYL4 | AP3B2 |  |
| MCM8 | EGR2 | KCNV1 | PAK1 |  |
| CD274 | EHMT1 | FHL2 | COX5B |  |
| PNPLA1 | EIF3G | RORB | TLN1 |  |
| BC147527 | EIF4E | STMN1 | RAB6B |  |
| SYNPR | EIF4EBP2 | ATP2B1 | YWHAG |  |
| GM10251 | ELAVL2 | INKA2 | CCT4 |  |
| ISM1 | ELAVL3 | SHROOM1 | IQSEC2 |  |
| OLFR310 | ELP4 | RPP25 | ALDH5A1 | |
| PTGDR | EML1 | ACTL10 | OGT |  |
| GM10339 | EN2 | GLP1R | HNRNPL |  |
| CBLN4 | EP300 | PINK1 | ATP5F1C | |
| GM2916 | EP400 | N4BP2 | PDE2A |  |
| ZFHX3 | EPC2 | FNDC1 | NAPG |  |
| GRAMD1C | EPHA6 | HECW2 | ATP6V1C1 | |
| FAM83C | EPHB2 | C530008M17RIK | SUCLA2 |  |
| PLA1A | EPHB6 | GOLM1 | CANX |  |
| CCR4 | EPPK1 | 1500011B03RIK | CAPZA2 |  |
| CALML4 | EPS8 | KMT2C | UNC13A |  |
| MCU | ERBB4 | TANC1 | PLXNA4 |  |
| NPFFR1 | ERG | CACNA1H | DLG2 |  |
| TMEM14A | EMSY | CAR12 | ARPC2 |  |
| TGFB2 | ERBIN | TENM3 | CACNA2D1 | |
| CHODL | ERMN | KCNG1 | GFAP |  |
| CLDN8 | ESR1 | SPP1 | TPM3 |  |
| DHDH | ESR2 | DDN | HTT |  |
| TIFAB | ESRRB | TTR | ATP6V0D1 | |
| STK3 | ETFB | ERMN | ARF1 |  |
| SYNPO2 | EXOC6B | HAPLN2 | TRIO |  |
| SIX6 | EXT1 | 2310022B05RIK | AAK1 |  |
| FGF1 | F13A1 | HRASLS | CAMKV |  |
| GCM2 | FABP3 | TTYH2 | ACAT1 |  |
| TEKT2 | FABP5 | CDH23 | ATP5H |  |
| MFSD7C | FABP7 | LRRTM1 | VPS35 |  |
| VIP | FAM19A2 | VMN1R206 | STX1A |  |
| MDC1 | FAM19A3 | SERTM1 | PRDX3 |  |
| ZFP28 | FAM47A | ITGAD | ALDH2 |  |
| NME8 | FAM92B | DKK3 | LMNA |  |
| HOXC9 | FAN1 | SLC26A4 | NPEPPS |  |
| SCNN1G | FAT1 | RUFY4 | NNT |  |
| PLD1 | FBN1 | HTR1A | SBF1 |  |
| ARHGAP6 | FBXO33 | GRIN3A | CLIP1 |  |
| MAFF | FBXO40 | PPME1 | NRXN1 |  |
| SEPP1 | FCRL6 | ZFP804A | DLST |  |
| CLPX | FEZF2 | SLC7A11 | ADD2 |  |
| GM21885 | FGA | OSBPL5 | UBR4 |  |
| E330009J07RIK | FGD1 | SV2B | DPYSL4 |  |
| DNAJC5G | FGFBP3 | COLEC12 | AP3D1 |  |
| ELAVL4 | FHIT | GMNC | SLC3A2 |  |
| OLFR976 | FLT1 | STARD8 | PRKCE |  |
| FBXW5 | FMR1 | GM14137 | EZR |  |
| PLCXD2 | FOLH1 | PTPRG | IDH3G |  |
| MUC19 | FOXG1 | DMBX1 | CCT6A |  |
| SLC18A2 | FOXP1 | BST2 | PSD3 |  |
| GM3055 | FOXP2 | SLC13A3 | GPHN |  |
| BC030500 | FRK | PTPN14 | SYNPO |  |
| DIO2 | ELOVL2 | IFIT3 | DST |  |
| BACH2 | EXOC3 | NOTCH2 | NTM |  |
| LHX3 | EXOC5 | NFIB | SDHB |  |
| GM5444 | EXOC6 | EPOP | SLC25A11 | |
| CALU | FAM135B | SCRN1 | SLC25A22 | |
| INS2 | FRMPD4 | PDE11A | GRM3 |  |
| METTL7A1 | GABBR2 | GM4951 | ITSN1 |  |
| ZFP781 | GABRA1 | BARHL2 | CA2 |  |
| TMEM26 | GABRA3 | SEMA4A | COX5A |  |
| ZFP839 | GABRA4 | CEP162 | VIM |  |
| LUC7L3 | GABRA5 | FCHO2 | VAMP2 |  |
| 1700034J05RIK | GABRB1 | MME | LRRC7 |  |
| LCE1M | GABRB3 | PHF24 | SV2B |  |
| CCDC69 | GABRQ | QSER1 | RAB3GAP2 | |
| LTA | GAD1 | COBL | DCTN2 |  |
| LEPR | GADD45B | SLC4A4 | PRPF8 |  |
| H2-M2 | GALNT13 | TNXB | ALDH6A1 | |
| CNGA1 | GALNT14 | DACH2 | SUCLG1 |  |
| GNG4 | GAN | IMPG1 | VARS |  |
| GM10935 | GAP43 | RNF182 | PIP5K1C |  |
| RELL1 | GAS2 | SEZ6L | HSP90B1 |  |
| CPM | GATM | ONECUT1 | SOD2 |  |
| MAP7 | GDA | LMO1 | PFKL |  |
| HIST1H2BG | GGNBP2 | KDR | PRDX1 |  |
| ZFP398 | GIGYF1 | TNC | RAB2A |  |
| SH2D1A | FBXO11 | TYRO3 | ACTR1A |  |
| ATP8B1 | FBXO15 | AMIGO2 | RPS3 |  |
| EDNRB | FER | BTBD3 | HPCA |  |
| GM17654 | FGFR2 | CCK | NPTN |  |
| ATP1B4 | GABRG3 | PIK3AP1 | ICAM5 |  |
| CALN1 | GALNT8 | LTBP4 | PRDX2 |  |
| OLFR1029 | GIGYF2 | DCHS1 | BCAN |  |
| RAMP2 | GLIS1 | ZFHX2 | DBNL |  |
| HNF4G | GLO1 | FOXP1 | RTN1 |  |
| ZFP943 | GLRA2 | ATF6 | SFXN3 |  |
| FAM24A | GNA14 | GSG1L | MTHFD1 |  |
| BNIP3L | GNAS | NLK | ARFGAP1 | |
| OLFR701 | GNB1L | CMYA5 | TRIM2 |  |
| PMAIP1 | GPC4 | SCN5A | LGI1 |  |
| GLUL | GPC6 | PRKCB | FLOT1 |  |
| GM14295 | GPHN | TRH | AP1B1 |  |
| ABCF1 | GPR139 | COL23A1 | EPB41L2 |  |
| ZBTB8B | GPR37 | PARP10 | KIF1A |  |
| A830018L16RIK | GPR85 | PAK1 | NCAN |  |
| MTURN | GPX1 | PRKACB | UBE2O |  |
| FAHD2A | GRIA1 | FOXC1 | CDC42BPB | |
| ZFP27 | GRID1 | KIF2A | BDH1 |  |
| RAB37 | GRID2 | ZFP142 | EEA1 |  |
| RNF144B | GRIK2 | PRRX2 | PGM2L1 |  |
| POU4F3 | GRIK4 | INTS7 | PSAT1 |  |
| B230219D22RIK | GRIK5 | TRPC5 | ADD1 |  |
| TMEM128 | GRIN1 | S100B | SKT |  |
| GM13089 | GRIN2A | TEX15 | PTPRS |  |
| SLC6A19 | GRIN2B | HIST2H2BB | CASK |  |
| 4931406C07RIK | GRIP1 | LRRC36 | IDH1 |  |
| EVC2 | GRM4 | TCTE1 | CORO1A |  |
| ASCL3 | GRM5 | WIPF3 | L1CAM |  |
| SPTSSA | GRM7 | LHFPL2 | COX4I1 |  |
| PRDM5 | GRM8 | ATP1A2 | PABPC1 |  |
| TTC29 | GRPR | GPR165 | SLC25A5 |  |
| CEP135 | GSK3B | HTR2C | PPP1CB |  |
| CASP14 | GSTM1 | NEXMIF | ATP8A1 |  |
| DRD1A | GTF2I | ZFP608 | ATP5I |  |
| CLEC1A | GUCY1A2 | KCNE2 | AGAP2 |  |
| RNF141 | H2AFZ | HIPK2 | SLC4A10 |  |
| UBE2H | HCN1 | RPS25 | PSAP |  |
| EGFR | HDAC3 | CD6 | XPO1 |  |
| GKN2 | HDAC4 | SCN7A | SYNCRIP | |
| MMP9 | HDC | RASA1 | IPO5 |  |
| BMP5 | HDLBP | GPRIN3 | VPS13C |  |
| DDO | HECTD4 | SPOCK3 | DNM3 |  |
| SLX4IP | HECW2 | GHSR | EPRS |  |
| COQ5 | HEPACAM | TRNP1 | RAB14 |  |
| ENTPD1 | HERC2 | CHEK2 | NDUFA10 | |
| SPATA45 | HIVEP3 | NR4A2 | EEF1G |  |
| UVRAG | HLA-A | LCAT | SV2A |  |
| NKD2 | HLA-B | GRIN2D | ARPC1A |  |
| HOOK2 | HLA-G | BEND6 | HNRNPH1 | |
| HIST2H2AB | HMGN1 | ZIC4 | CRYM |  |
| MCEMP1 | HNRNPH2 | ASCL1 | CNTNAP1 | |
| TMEM240 | HNRNPU | CAVIN3 | DHX9 |  |
| GPR45 | HOMER1 | ITIH2 | THY1 |  |
| ROCK1 | HOXA1 | CHD3 | GAP43 |  |
| OLFR921 | HOXB1 | SPOCK1 | CALB1 |  |
| TAL2 | HRAS | NECAB2 | PITPNA |  |
| EPHA5 | HS3ST5 | SLC45A4 | KPNB1 |  |
| PPM1B | HSD11B1 | FAM78A | PEBP1 |  |
| ARRDC2 | HTR1B | TEAD1 | SLC17A7 |  |
| TRPM8 | HTR2A | SLC47A1 | SHANK3 |  |
| GPR98 | HTR3A | FBLN1 | DPP10 |  |
| PTGFR | HTR3C | CD34 | SND1 |  |
| FBXO40 | GPD2 | ISM1 | TPPP |  |
| TRAF3 | GRID2IP | CLEC2D | PHYHIPL | |
| GM5627 | GRIK3 | KCNN2 | YARS |  |
| GRIN2C | GRM1 | LRRC10B | ACTR3 |  |
| IRG1 | GSN | SLC7A14 | NDUFA9 |  |
| TCF12 | HCFC1 | SLC35D3 | ADAM22 |  |
| ASAH2 | HDAC6 | DGKG | CORO1C |  |
| TNIP3 | HDAC8 | RNF207 | RASAL1 |  |
| AA986860 | HLA-DRB1 | PDYN | GCN1 |  |
| EIF4E3 | HTR7 | FLRT2 | HIST1H1C | |
| MRGPRX2 | HUWE1 | ETV1 | CBR1 |  |
| ZFP493 | HYDIN | TMEM28 | RAB7A |  |
| CACNA1S | ICA1 | AGO2 | PCBP1 |  |
| NKX2-1 | IFNG | CHST9 | GNG2 |  |
| EFCAB1 | IL17RA | GM1043 | DDB1 |  |
| FAM105A | IL1R2 | NAAA | CNKSR2 |  |
| GM10742 | IL1RAPL1 | GPR6 | SHANK2 |  |
| PAX8 | IL1RAPL2 | STRIP2 | PDHX |  |
| IL12B | ILF2 | TBC1D8 | RPN1 |  |
| TRIM67 | IMMP2L | PYGO1 | ETFA |  |
| UBXN4 | INPP1 | CELF2 | UQCRFS1 | |
| JMJD8 | INTS6 | PRSS23 | RUFY3 |  |
| LIN28B | IQGAP3 | ZFP384 | TMOD2 |  |
| CD200R4 | IQSEC2 | SGTB | BPNT1 |  |
| ARIH1 | IRF2BPL | NID1 | CTNND2 |  |
| EIF2AK2 | ITGB3 | INPP4B | IMPA1 |  |
| 2300009A05RIK | ITGB7 | F2R | GNAZ |  |
| MAB21L2 | ITPR1 | COL1A2 | PSMC3 |  |
| TET3 | JAKMIP1 | F13A1 | GSTP1 |  |
| CD80 | JARID2 | TYMP | RPS2 |  |
| TNFSF18 | JMJD1C | PLCB4 | COX6B1 |  |
| FAM110A | KANK1 | CHGA | RAP2B |  |
| STIL | KAT2B | MAL | RPS4X |  |
| HNRNPDL | KAT6A | MORC4 | PPP2CB |  |
| FGF7 | KATNAL1 | LAMB1 | SORBS2 |  |
| PCDHB3 | KATNAL2 | GDPD2 | ARHGEF2 | |
| KLHL8 | KCNB1 | HMBOX1 | DDX5 |  |
| CCDC92 | KCND2 | CCL27A | HUWE1 |  |
| YAE1D1 | KCND3 | F2RL3 | MADD |  |
| TCF20 | KCNJ10 | CACNB4 | GPR158 |  |
| CNIH1 | KCNJ2 | SH2D5 | WDR37 |  |
| PEX13 | KCNK7 | FBN2 | RAPGEF2 | |
| RSPH4A | KCNMA1 | SLBP | SFPQ |  |
| UCK1 | KCNQ2 | HGF | TF |  |
| IKZF1 | KCNQ3 | KLHL7 | ACSBG1 |  |
| TIA1 | KCNT1 | CABP1 | SCRN1 |  |
| MINPP1 | KCTD13 | PLIN3 | HNRNPM | |
| FHL5 | KDM4B | CDR1 | PDCD6IP | |
| EID2B | KDM5B | NTRK3 | MPP2 |  |
| LHFPL4 | KDM5C | CPNE7 | 3-Sep |  |
| PTPN7 | KDM6A | BCAR3 | TTN |  |
| ZFP429 | KDM6B | CCDC80 | GNAI1 |  |
| MYLK4 | KHDRBS2 | RAP1GAP | P4HB |  |
| CTU1 | KIAA1586 | ARHGAP32 | GNAQ |  |
| CDK8 | KIF13B | ARL4D | HNRNPUL2 | |
| PCDH17 | KIF5C | ADAP1 | GRIN2B |  |
| ANKRD13B | KIRREL3 | KCNJ10 | GDI2 |  |
| ASNS | IFNGR1 | DCLK3 | SORBS1 |  |
| DEFB12 | IL16 | RAPGEF5 | UGGT1 |  |
| GM10176 | IL17A | COL11A1 | 9-Sep |  |
| FANK1 | IL6 | ZFP831 | PIP4K2B |  |
| NOP56 | ITGA4 | TRRAP | HNRNPA3 | |
| LAYN | KCNJ12 | STX1A | ASRGL1 |  |
| ZFYVE28 | KCNJ15 | ADRA2A | SIPA1L1 |  |
| TMEM216 | KDM4C | SPRED1 | LONP1 |  |
| CD59A | KHDRBS3 | NDFIP1 | CAMK2G | |
| EIF5 | KIF14 | UBAP1L | DPP3 |  |
| CNTFR | KIF21B | PARP8 | RTCB |  |
| TSEN15 | KIT | FAM180A | CAP2 |  |
| ERLIN2 | KLC2 | FGF22 | PPA1 |  |
| SCN9A | KLF16 | ANLN | UQCRB |  |
| GM5089 | KMT2A | LPAR4 | MPP6 |  |
| FAM19A3 | KMT2C | P2RY14 | MINK1 |  |
| RBM7 | KMT2E | ST5 | DBN1 |  |
| TMEM132C | KPTN | MTSS1 | DSTN |  |
| A930018M24RIK | KRR1 | KCNJ13 | SNCA |  |
| FKBP10 | KRT26 | SLC22A8 | RPL7A |  |
| E130114P18RIK | LAMA1 | EBF4 | LMNB1 |  |
| GM5096 | LAMB1 | ESM1 | MAP2K1 |  |
| FOXN2 | LAMC3 | EHD4 | WARS |  |
| SHISA3 | KMT5B | TCF4 | CAPZB |  |
| BBIP1 | KMO | LRP1 | RPS27A |  |
| HRH2 | LAT | FAM107B | PHB |  |
| GM5493 | LEO1 | PLCXD3 | RACK1 |  |
| VMN2R88 | LEP | BC005561 | CTNNB1 |  |
| A830092H15RIK | LILRB2 | SPSB1 | PPP1R7 |  |
| PIK3IP1 | LIN7B | PPM1E | IARS2 |  |
| ZFP867 | LMX1B | RNF227 | ACOT7 |  |
| GCA | LMX1B | SERPINH1 | PARK7 |  |
| PLCXD3 | LPL | SUN1 | PPID |  |
| TTC37 | LRBA | SOX9 | NDUFV2 |  |
| RFX1 | LRFN2 | GLDC | EFHD2 |  |
| OLFR1020 | LRFN5 | TMEM150C | PFN2 |  |
| 1700029F12RIK | LRP2 | KBTBD11 | HYOU1 |  |
| INO80E | LRP2BP | GRM3 | PTK2B |  |
| EDNRA | LZTR1 | ANGPT1 | CPNE6 |  |
| SRGAP1 | MACROD2 | ROBO1 | SARS |  |
| EDN2 | MAGEL2 | NUDC | GPM6B |  |
| PVRL3 | MAOA | POU3F2 | AKR1B1 |  |
| CTBP2 | MAP2 | CADM1 | PSMC2 |  |
| UPK3B | MAPK1 | VNN3 | RAB11B |  |
| GCDH | MAPK3 | ZCCHC14 | HNRNPA1 | |
| NIPAL4 | LNPK | WNK1 | IDH2 |  |
| DISP2 | MARK1 | RP9 | TUBB5 |  |
| USP15 | MBD1 | ADAMTS1 | CPE |  |
| DHX15 | MBD3 | IFITM3 | NF1 |  |
| 9030612E09RIK | MBD4 | ITPKB | CTTN |  |
| SAMD12 | MBD5 | HRH3 | CSNK2A1 | |
| SEC24D | MBD6 | ENPP6 | DDX3X |  |
| A330008L17RIK | MBOAT7 | PRSS12 | GIT1 |  |
| GM8180 | MCM4 | TRP53BP1 | FAM120A | |
| THEMIS2 | MCM6 | PTGS2 | DLGAP3 |  |
| SMPD5 | MCPH1 | SLCO3A1 | ATP2B4 |  |
| CCL17 | MDGA2 | TIMM8B | PHF24 |  |
| GM17175 | MECP2 | PIP4K2A | CEP170B |  |
| CCDC181 | MED12 | MAS1 | ANKS1B |  |
| TMEM9 | MED13 | RFC4 | ME3 |  |
| TMED7 | MED13L | ACSL5 | RNH1 |  |
| SEC14L3 | MEF2C | XLR4A | PCCA |  |
| LRRFIP2 | MEGF10 | TBR1 | FAM49B |  |
| FAM163A | MEGF11 | SNAP25 | ATP5L |  |
| GM17026 | MET | NCAM1 | LAP3 |  |
| INPP4B | MFRP | ZBTB22 | UBE2V1 |  |
| GM8138 | MIB1 | IGFBP2 | CNDP2 |  |
| GM8122 | LRPPRC | MTURN | NAPA |  |
| TMEM154 | LRRC1 | SEMA7A | AKR1A1 |  |
| DGKB | LRRC4 | FAT1 | RHOA |  |
| SLC25A53 | LRRC7 | STRBP | RNPS1 |  |
| GM8005 | LZTS2 | 5730522E02RIK | SLC4A4 |  |
| GM17124 | MAOB | DCHS2 | SOD1 |  |
| ACVR1B | MAPK12 | PBX1 | NME1 |  |
| SPG20 | MCC | IKZF2 | MAP4 |  |
| ZFP174 | MEIS2 | CECR2 | RPL6 |  |
| BRSK1 | MKL2 | DCC | PPP3CB |  |
| GM8024 | MOCOS | SLIT2 | RAC1 |  |
| MLH3 | MPP6 | BSPH1 | PAFAH1B1 | |
| TMEM129 | MSANTD2 | BTBD10 | YWHAQ |  |
| ADAMTS9 | MSR1 | PSPN | PLXNA1 |  |
| SNX24 | MTF1 | CLDN11 | GPD1L |  |
| ATMIN | MTHFR | MRPL41 | SEC31A |  |
| GMEB1 | MTOR | D10WSU102E | CNTN2 |  |
| GM9922 | MTR | GPR22 | GLG1 |  |
| SERPINA3N | MUC12 | NDN | SYP |  |
| FOXE1 | MUC4 | LRRN2 | MAOA |  |
| HYAL1 | MYH10 | ADGRG6 | GK |  |
| LDB2 | MYH4 | ARHGAP8 | NRXN3 |  |
| PSMB10 | MYO16 | ERP27 | OTUB1 |  |
| 5330417H12RIK | MYO1A | DCTN6 | EIF4G3 |  |
| ANK3 | MIR137 | HDAC1 | DLG1 |  |
| HRH3 | MAGED1 | HUNK | ATL1 |  |
| FAP | MAL | SPEER4B | PPME1 |  |
| MGAT4A | MAPK8IP2 | KIT | ABLIM1 |  |
| ZFP773 | MC4R | HELZ | IQSEC1 |  |
| D830039M14RIK | MNT | PCK2 | SH3GLB2 | |
| 1700023E05RIK | MSN | KIF5A | HDLBP |  |
| GM15821 | MSNP1AS | SNX5 | DDX1 |  |
| TBCK | MTX2 | TMOD1 | UGP2 |  |
| CCDC32 | MYO1E | SAMD14 | TALDO1 |  |
| ADORA2B | MYO5A | SUB1 | GRK2 |  |
| GM10874 | MYO5C | MMD2 | RIMS1 |  |
| PDE4B | MYO9B | EFS | GLOD4 |  |
| D130043K22RIK | MYOZ1 | SLC24A3 | ATP5F1 |  |
| GPR101 | MYT1L | TOMM40 | ATIC |  |
| AEN | NAA15 | SAP30BP | D10JHU81E | |
| SLC24A4 | NAALADL2 | LGALS3BP | PRPS1 |  |
| LUZP1 | NACC1 | GDE1 | RPL4 |  |
| NECAB1 | NAV2 | SYNDIG1 | ALDH7A1 | |
| AVPR1A | NBEA | WISP1 | COPB1 |  |
| SRSF10 | NCKAP1 | GRN | CUL3 |  |
| GM7980 | NCKAP5 | TDP1 | EHD3 |  |
| CREG2 | NCKAP5L | P2RY2 | AK1 |  |
| GM7970 | NCOR1 | MSX1 | RBMX |  |
| CNTNAP5A | NEFL | HTR1F | CLIP2 |  |
| MAP3K13 | NEO1 | IER5 | DDX39B |  |
| GM3072 | NF1 | ADCYAP1R1 | PRKACA |  |
| TBC1D5 | NFIA | IRS2 | EIF3A |  |
| GM8898 | NFIX | PAPPA | GJA1 |  |
| PDSS1 | NINL | ZAN | ALDH1A1 | |
| MRGPRB1 | NIPA1 | IFRD1 | GRIN1 |  |
| LCN12 | NIPA2 | FAM76A | RAB1A |  |
| GM4723 | NIPBL | CRYBB3 | DLG3 |  |
| ACVR1 | NLGN1 | ZDHHC22 | HP1BP3 |  |
| CDK9 | NLGN2 | MYH10 | PSMD1 |  |
| HIST1H2BQ | NLGN3 | AKNA | NT5DC3 |  |
| GPR6 | NEXMIF | DGKD | ASTN1 |  |
| CSNK1A1 | NLGN4X | TUBB2B | FBXO41 |  |
| SLC7A12 | NOS1AP | SZT2 | MAPRE3 |  |
| PAPOLB | NOS2 | EPHA3 | TECPR1 |  |
| PCGF5 | NR1D1 | TAGLN | LSAMP |  |
| RCBTB1 | NR2F1 | KHDC1C | MYEF2 |  |
| ACE2 | NR3C2 | TMCC1 | WDR47 |  |
| ANKS1B | NR4A2 | PDS5B | PHYHIP |  |
| GM7954 | NRCAM | ADAMTS3 | PACS1 |  |
| RPS29 | NRP2 | GPC3 | PSMD2 |  |
| GM17027 | NRXN1 | PRKAR1B | RRBP1 |  |
| GM8165 | NRXN2 | FRMD4A | NDUFS3 |  |
| XKR8 | NRXN3 | TIMP3 | NDUFA13 | |
| CDH17 | NSD1 | GLCE | MTOR |  |
| CCNE1 | NTNG1 | LAMC3 | NDUFA7 |  |
| GM3327 | NTRK1 | CCR2 | GRIA3 |  |
| TMEM194B | NTRK2 | SNHG11 | RAP1GAP | |
| RAPGEF5 | NTRK3 | REX1BD | KALRN |  |
| NOA1 | NUAK1 | RRAS2 | PDIA4 |  |
| GM14443 | NUP133 | OLFML2B | PSMD3 |  |
| GM8212 | NXPH1 | RSAD1 | RPL3 |  |
| KLHL17 | OCRL | KCNE3 | ACO1 |  |
| TSHZ1 | ODF3L2 | NUDT4 | ACADL |  |
| AF529169 | OFD1 | RPS8 | EIF5A |  |
| H2-T10 | OPHN1 | CNTNAP5A | SIRPA |  |
| ZFP772 | OR1C1 | MB21D2 | ANXA7 |  |
| AA474408 | NSMCE3 | SERPINI1 | HNRNPD | |
| IFNAR1 | NDUFA5 | FYCO1 | PPP5C |  |
| GM16390 | NEGR1 | HNMT | SNRNP200 | |
| EHF | NELL1 | CDH18 | CAMK2D | |
| GM21977 | NFIB | PGF | PITPNM2 | |
| ELAVL1 | NLGN4Y | SOWAHC | NDUFA12 | |
| MAL | NOS1 | ZFHX3 | PCK2 |  |
| ELOVL3 | NOTCH2NL | FAM171A1 | CADM2 |  |
| ZSWIM6 | NPAS2 | PTPN12 | MAOB |  |
| 1700042B14RIK | NR1H2 | KLK6 | COPA |  |
| RBPJ | NRG1 | CSRP1 | MICAL3 |  |
| RLIM | NUDCD2 | PDRG1 | AFG3L2 |  |
| 1700066M21RIK | NXF5 | KCTD1 | CSDE1 |  |
| PCDHB11 | OGT | DPM1 | PLS3 |  |
| KLRC3 | OPRM1 | SRP9 | GARS |  |
| GM16506 | OR2M4 | PCDH1 | RABGGTA | |
| 9130409I23RIK | OR2T10 | CLIC5 | HNRNPC | |
| GM8232 | OR52M1 | REV3L | DPP6 |  |
| ZFP518A | OTUD7A | ESF1 | ATP5J2 |  |
| UVSSA | OTX1 | GBX1 | H3F3C |  |
| RBM6 | OXT | PARM1 | H1F0 |  |
| GM4841 | OXTR | NOV | RPSA |  |
| TMEM218 | P2RX4 | CACNA1C | KIF2A |  |
| GM16223 | P2RX5 | JHY | SSB |  |
| P2RX1 | P4HA2 | OMD | MIF |  |
| RCVRN | PACS1 | AY358078 | ANXA5 |  |
| CYP2R1 | PACS2 | SIGLECF | GAD2 |  |
| CREB3L1 | PAH | SETMAR | AHCY |  |
| NPHS1 | PARD3B | GABARAPL1 | PSMC4 |  |
| CD7 | PAX5 | VMN2R54 | COPS2 |  |
| ZFP53 | PAX6 | OASL2 | KCNAB2 |  |
| LOX | PCCA | PLCB1 | CPLX1 |  |
| PSEN1 | PCCB | PAQR8 | SEC23A |  |
| ZBTB20 | PCDH10 | ARHGAP15 | KHSRP |  |
| CORIN | PCDH11X | SMARCD2 | DIP2B |  |
| 1700039E15RIK | PCDH15 | CYP26B1 | ABR |  |
| D2HGDH | PCDH19 | NRAP | FLOT2 |  |
| SLC4A5 | PCDH8 | HS3ST1 | MAPK10 |  |
| SLC30A1 | PCDH9 | AKAP5 | CEP170 |  |
| 1700003H04RIK | PCDHA1 | BHLHE40 | XPNPEP1 | |
| HOXA6 | PCDHA10 | NT5C3 | SRSF1 |  |
| RBM3 | PCDHA11 | MT-ND4 | PPP1R9B | |
| IGSF3 | PCDHA12 | SEL1L2 | NEGR1 |  |
| 4930597O21RIK | PCDHA13 | 5730409E04RIK | SRGAP3 |  |
| TMBIM1 | PCDHA2 | RERG | CORO2B |  |
| GM8068 | PCDHA3 | IIGP1 | QDPR |  |
| KITL | PCDHA4 | PIK3R1 | ACAA2 |  |
| DOC2G | PCDHA5 | TBC1D8B | CADPS2 |  |
| ABLIM3 | PCDHA6 | TREX2 | THOP1 |  |
| ZFP644 | PATJ | SH2D1B1 | PLCL2 |  |
| GLRA1 | PCDHA7 | BMP3 | BLMH |  |
| C330007P06RIK | PCDHA8 | DDX58 | ITPKA |  |
| 4933403O08RIK | PCDHA9 | MGST3 | DYNC1LI1 | |
| CHN2 | PCDHGA11 | ZIK1 | NCALD |  |
| GM14440 | PDCD1 | ERH | ETFDH |  |
| WASL | PDE4B | PCDHGA6 | NONO |  |
| EXOC4 | PDZD4 | PABPC1 | GAK |  |
| ZFP790 | PECR | MAGEH1 | DDAH1 |  |
| CARF | PER1 | DIO3 | IVD |  |
| COLEC12 | PER2 | NPAS3 | SCAMP5 |  |
| GM7120 | PGLYRP2 | CRIM1 | AUH |  |
| NXNL1 | PHF2 | BRWD3 | 6-Sep |  |
| MOB3A | PHF3 | FAM57A | CYP46A1 | |
| GDAP1 | PHIP | SNRPD3 | AIFM1 |  |
| KLHL5 | PHRF1 | SH3BP4 | HNRNPDL | |
| LRFN5 | PIK3R2 | OSBPL10 | PRRT2 |  |
| POLR3F | PINX1 | ATP6V1H | CAPN2 |  |
| F5 | PITX1 | PIP4K2C | RABEP1 |  |
| P2RY4 | PLCB1 | PNO1 | PITPNM1 | |
| RGS5 | PLCD1 | GAA | CTBP1 |  |
| UGT2B34 | PLN | TRAF3 | RPS16 |  |
| NMNAT2 | PLXNA3 | NT5DC3 | RPL7 |  |
| SURF6 | PLXNA4 | GAS7 | ASS1 |  |
| KDELR2 | PLXNB1 | TDRD5 | MAG |  |
| PANK3 | PNPLA7 | GBP2 | LMNB2 |  |
| BEAN1 | POGZ | AP1S2 | LTA4H |  |
| CENPC1 | POLA2 | CPA4 | CD81 |  |
| SCAMP1 | POMT1 | BC049762 | GNL1 |  |
| SENP6 | POT1 | STON2 | GAD1 |  |
| MLF1 | POU3F2 | CCKBR | SLC12A2 |  |
| TCTEX1D1 | PPM1D | FAM198A | RAB5B |  |
| KLHL1 | PPP1R3F | MROH7 | PSMC1 |  |
| FAM155A | PPP2R1B | CNTNAP4 | RPS3A |  |
| SETD7 | PPP2R5D | STRN | WNK2 |  |
| INSR | PREX1 | RAPGEF4 | KCTD16 |  |
| PRLR | PRICKLE1 | DPYSL3 | ARHGAP1 | |
| DDX47 | PRICKLE2 | ALOXE3 | VCAN |  |
| IFI44 | PRKCB | SCCPDH | MTCH2 |  |
| OXCT1 | PRKD1 | FUT2 | GANAB |  |
| ASAP1 | PRKDC | TMEM35A | NARS |  |
| GM6482 | PRODH | POLE3 | VCPIP1 |  |
| SERPINB10 | PRPF39 | AHNAK | NGEF |  |
| NCBP2 | PRR12 | NME7 | EIF3B |  |
| BMP7 | PRUNE2 | TMEM108 | CADM4 |  |
| GM7951 | PSD3 | WDR6 | SNAP47 |  |
| GM10545 | PSMD10 | BCL6 | ARHGAP35 | |
| NAPG | PSMD12 | PDE3A | ST13 |  |
| PDE3A | PTBP2 | HS3ST2 | UQCRQ |  |
| NUP54 | PRKN | SETBP1 | ETFB |  |
| HS6ST2 | PAFAH1B1 | DOT1L | XPO7 |  |
| TIMM8A2 | PAK2 | PIP4K2B | SACS |  |
| TMEM170B | PCDHAC1 | CDHR1 | PREP |  |
| CPNE4 | PCDHAC2 | ATPAF1 | AK3 |  |
| ABCA8B | PDE1C | MPV17L | AJM1 |  |
| ACAD9 | PDE4A | ALS2CL | NIPSNAP1 | |
| CPD | PEX7 | TMEM186 | GPX4 |  |
| MAPK14 | PHB | PEAK1 | PSMA3 |  |
| FAM46A | PHF8 | GCNT2 | PRKAR2A | |
| VNN3 | PIK3CG | SKIL | RPLP0 |  |
| GRAP2 | PLAUR | GPD2 | AP1G1 |  |
| OSGEPL1 | POMGNT1 | PLXNA1 | GNA13 |  |
| TAOK3 | PON1 | ATP1B2 | TJP1 |  |
| RBMS3 | PPFIA1 | TSPAN5 | TST |  |
| TMIE | PRSS38 | ATP2B2 | PSMC6 |  |
| CYP7A1 | PTCHD1 | EQTN | RPL23A |  |
| 9930111J21RIK2 | PTEN | ELOVL6 | KIAA1468 | |
| ZMYM5 | PLPPR4 | CAMKK2 | EPS15L1 |  |
| KIF5A | PPP1R1B | ITSN1 | ALCAM |  |
| CDC73 | PTGER3 | 1810055G02RIK | PHGDH |  |
| DNAJB11 | PTK7 | SAP130 | MOG |  |
| CXXC4 | PTPN11 | STK31 | NPM1 |  |
| RWDD3 | PTPRB | LOXL1 | PEA15 |  |
| 43712 | PYHIN1 | FAM57B | OMG |  |
| LINGO3 | QRICH1 | CYBRD1 | MYO6 |  |
| ERCC2 | RAB11FIP5 | SPARCL1 | NOMO1 |  |
| MPEG1 | RAB2A | VSIG2 | PPP2R2A | |
| GM8229 | RAB39B | TDO2 | ARHGAP32 | |
| HIST2H2BB | RAB43 | CLDN1 | TRIM9 |  |
| GPR158 | RAC1 | CDNF | SCAI |  |
| SOGA3 | RAD21L1 | ANGPTL4 | 8-Sep |  |
| HHIPL1 | RAI1 | TMEM178 | FERMT2 |  |
| VGLL3 | RANBP17 | GUCY1B1 | PDXK |  |
| PARD3 | RAPGEF4 | PCDHB11 | MAPRE2 |  |
| THEMIS | RB1CC1 | PTPRF | WASF1 |  |
| NTM | RBFOX1 | ANKRD61 | CISD1 |  |
| BRINP1 | RBM27 | PCSK5 | OSBPL1A | |
| RMND1 | RBM8A | RFXAP | HADHB |  |
| MROH1 | RBMS3 | HAPLN1 | PCCB |  |
| ELOVL6 | REEP3 | RXRG | FIS1 |  |
| KLHL34 | RELN | GPRIN2 | NDUFA6 |  |
| FECH | RERE | CRTAM | TARS |  |
| DISC1 | RFWD2 | STK36 | CORO7 |  |
| ZBTB49 | RFX3 | THBS1 | DLGAP1 |  |
| ASB4 | RGS7 | CALM2 | NDUFB10 | |
| NAIP1 | RHEB | PCDHB6 | ACTN2 |  |
| MOCS2 | RIMS1 | NECAB3 | HAPLN1 |  |
| BEND6 | RIMS3 | RERE | TWF2 |  |
| IGF1 | RLIM | LPCAT4 | LETM1 |  |
| LDHB | RNF135 | EPB41L4A | ARFGEF2 | |
| OLFR443-PS1 | RNF38 | FER1L4 | DHX15 |  |
| CHERP | ROBO1 | DTHD1 | PGRMC1 |  |
| A330050F15RIK | ROBO2 | TCP11L1 | KLC2 |  |
| 4732440D04RIK | RORA | ITGA8 | COPS4 |  |
| STAT1 | RPL10 | MRGPRF | ME1 |  |
| SLC35D1 | RPS6KA2 | AVP | H2AFX |  |
| ZSWIM5 | RPS6KA3 | CROCC | FKBP4 |  |
| GM8773 | SAE1 | PACS2 | RAB18 |  |
| FMO6 | SATB2 | EVI5 | ACADVL |  |
| AFMID | SBF1 | LPP | ADCY9 |  |
| CADM2 | SCFD2 | TSC2 | GABRA1 |  |
| WDTC1 | SCN1A | CDK14 | RAP1A |  |
| MOB3C | SCN2A | UBR4 | PDE1B |  |
| CBLN3 | RP11-1407O15.2 | BRSK2 | LRRC47 |  |
| PDK4 | PTGS2 | KIRREL3 | DIRAS2 |  |
| RBM28 | PTPRC | RBP3 | GRB2 |  |
| BC025920 | PTPRT | CDC73 | HRAS |  |
| TSPAN18 | PVALB | VIM | FKBP3 |  |
| KIF6 | PXDN | KCNJ4 | HSPE1 |  |
| PREX1 | RAB19 | PFDN2 | CLTB |  |
| ZFP113 | RAD21 | PENK | PTPN23 |  |
| PLK5 | RASD1 | TMEM232 | RAB35 |  |
| CCDC160 | RASSF5 | HOMER1 | WDFY3 |  |
| FOXRED2 | RHOXF1 | 9330182L06RIK | EIF4B |  |
| IL4RA | RIT2 | KCNQ4 | SAMM50 |  |
| ZFP820 | RNPS1 | STAC | HPCAL4 |  |
| BIRC3 | RPP25 | AGO1 | DLGAP2 |  |
| PI15 | SAMD11 | PSMC3 | ABI1 |  |
| PITHD1 | SASH1 | PTCHD1 | CACNB4 |  |
| ZBTB46 | SCN4A | COQ2 | HNRNPLL | |
| UPRT | SCN5A | PLAGL1 | NPTXR |  |
| GM4791 | SCN7A | IYD | COX6C |  |
| NUTF2-PS1 | SCN8A | GNG4 | CNTNAP2 | |
| D830013O20RIK | SCN9A | PCDHGA11 | GLRX3 |  |
| ACPP | SCP2 | NFASC | OLA1 |  |
| DLX1 | SDC2 | GM3252 | ATG7 |  |
| GLP1R | SDK1 | SATB2 | AGK |  |
| PLCB1 | SEMA5A | GPX2 | PSMA6 |  |
| SCAMP5 | SETBP1 | PHACTR1 | ASAP1 |  |
| LYPD6 | SETD1B | C3AR1 | NDRG3 |  |
| MOBP | SETD2 | SCRG1 | VAPA |  |
| TMEM167B | SETD5 | ISG15 | MECP2 |  |
| GABRQ | SETDB1 | MPL | TPR |  |
| URAH | SETDB2 | PTGFR | EFTUD2 |  |
| RASGEF1A | SEZ6L2 | ZFP239 | RGS7 |  |
| CD24A | SGSH | DAB2 | STRN |  |
| ADAM23 | SGSM3 | POGZ | OLFM1 |  |
| CDCP1 | SH3KBP1 | SRSF5 | ATP1B2 |  |
| TNFRSF12A | SHANK1 | RNF7 | CTSD |  |
| GP9 | SHANK2 | CTTNBP2 | MARCKS | |
| FBXO28 | SHANK3 | NCKAP5 | RAB5C |  |
| CCR9 | SHOX | NCAPH | ACSL1 |  |
| LAMC1 | SIK1 | FUT7 | PURA |  |
| TAL1 | SIN3A | FAM131B | HIST1H1E | |
| GABRB3 | SLC12A5 | FGF11 | RPL5 |  |
| GM17567 | SLC16A3 | NHSL2 | GSTM5 |  |
| NSFL1C | SLC16A7 | OLFR172 | CIT |  |
| HMGA2 | SLC1A1 | SRRM4 | INPP1 |  |
| RBM31Y | SLC1A2 | UBE2G2 | UBE2N |  |
| TFAP2A | SLC22A9 | THBS4 | PSMC5 |  |
| RAB14 | SLC25A24 | RNF39 | RPS11 |  |
| UNC45A | SLC25A39 | ALDH1A1 | GNB5 |  |
| ZMAT1 | SLC27A4 | RMDN3 | SLC8A1 |  |
| SERPINB6B | SLC29A4 | ADAM12 | SLC9A3R1 | |
| HSD11B1 | SLC30A5 | OLFR557 | ADCY5 |  |
| KDELC2 | SLC38A10 | HUWE1 | CSRP1 |  |
| CYS1 | SLC45A1 | PPP1R3C | ARHGAP5 | |
| 2900092C05RIK | SLC4A10 | AQP1 | FABP5 |  |
| HEPHL1 | SLC6A1 | LAPTM4A | GRM5 |  |
| VSX2 | SLC6A3 | 7-Sep | MTHFD1L | |
| FAM98B | SLC6A4 | BTBD8 | STXBP5L | |
| TNFRSF19 | SLC22A15 | MYLK | TOM1L2 |  |
| INA | SLC24A2 | EFNB2 | ACACA |  |
| NIT1 | SLC25A12 | NR6A1 | MYO1D |  |
| TYK2 | SLC25A14 | RASGEF1C | PZP |  |
| RASD1 | SLC25A27 | MYH1 | PTPRD |  |
| ABHD10 | SLC30A3 | NTF3 | SOGA3 |  |
| ERMP1 | SLC33A1 | PHF2 | MYH14 |  |
| ST6GALNAC2 | SLC35A3 | DYNC1LI2 | VAT1L |  |
| EML6 | SLC35B1 | PRR32 | ADGRL3 |  |
| GRPEL1 | SLC6A8 | ANKRD45 | CAMSAP3 | |
| OPRK1 | SLC7A3 | ARHGEF1 | ANKFY1 |  |
| TENM4 | SLC7A5 | CPNE4 | RHOT1 |  |
| UNC5C | SLC7A7 | OLFR267 | ELMO2 |  |
| TRAP1 | SLC9A6 | FYB2 | KBTBD11 | |
| 2310039H08RIK | SLC9A9 | NPHS2 | TXNL1 |  |
| HIST3H2A | SLCO1B3 | MRPL48 | NDUFS8 |  |
| CTSK | SLIT3 | PTGFRN | CLYBL |  |
| SCARB2 | SLITRK5 | PCDH10 | PIP4K2C |  |
| PLS1 | SMAD4 | LRRN1 | DARS |  |
| ADRA1B | SMARCA2 | GZMK | HIBADH |  |
| TBL1XR1 | SMARCA4 | ZFP945 | RPL14 |  |
| WDR46 | SMARCC2 | SYT17 | NECAP1 |  |
| MEST | SMC1A | GRHL1 | RARS |  |
| GM14147 | SMC3 | FER1L5 | DYNLL2 |  |
| GTF2A1 | SMG6 | PDE10A | CUL5 |  |
| OLFR606 | SNAP25 | FBXW7 | ARPC5L |  |
| SGSH | SND1 | FRRS1L | IPO7 |  |
| NOP58 | SERPINE1 | PRRC2C | UPF1 |  |
| GSTZ1 | SLC22A3 | SV2C | RAPGEF4 | |
| OLFR464 | SLC39A11 | A830031A19RIK | CSE1L |  |
| SEC22B | SNRPN | ASIC4 | LANCL2 |  |
| 0610010F05RIK | SNTG2 | MRGPRH | CEND1 |  |
| A930033H14RIK | SNX14 | SGCZ | HIP1R |  |
| HPCAL4 | SNX19 | IDO1 | AFDN |  |
| GABRG1 | SOD1 | RIF1 | H2AFY |  |
| RPA1 | SOX5 | H2-Q4 | SYT7 |  |
| ICOSL | SPARCL1 | YPEL4 | GSK3B |  |
| OLFR128 | SPAST | STUM | SNX1 |  |
| LTB4R1 | SPP2 | GM7694 | PACSIN2 |  |
| PARP16 | SRCAP | CNNM1 | PSMA5 |  |
| BIRC6 | SRD5A2 | PLIN4 | AGRN |  |
| HIST1H2BP | SRGAP3 | GM14025 | FBXL16 |  |
| OLFR1443 | SRRM4 | TFCP2L1 | AKAP5 |  |
| FAM19A2 | SRSF11 | PLXNB1 | FRY |  |
| DCTN4 | SSPO | LRRK2 | PSMB5 |  |
| SERTAD2 | SSRP1 | ANK3 | NMT1 |  |
| NODAL | ST7 | PRRC2A | DYNC1I1 | |
| ARHGAP30 | STAG1 | SRGAP3 | SRC |  |
| SYN3 | STAT1 | 1110032F04RIK | CAMK4 |  |
| STMN2 | STX1A | RARA | FTH1 |  |
| SYT12 | STXBP1 | KCNH7 | GSN |  |
| SPTBN4 | STXBP5 | CCL28 | GRIA1 |  |
| EGFL8 | SUCLG2 | DHCR7 | PLAA |  |
| SSBP4 | SYAP1 | TMEM130 | ADH5 |  |
| 6030419C18RIK | SYN1 | SYT4 | OAT |  |
| GBX2 | SYN2 | B3GAT2 | MAP2K4 |  |
| 4930435E12RIK | SYN3 | DUSP4 | PA2G4 |  |
| ZMYND15 | SYNE1 | NEK6 | RPL9 |  |
| GZF1 | SYNGAP1 | ACTR1B | ATP6V1D | |
| ST8SIA6 | SYNJ1 | TAGLN3 | RALA |  |
| ALB | TAF1 | HECTD4 | RPS12 |  |
| 1700028K03RIK | TAF1C | GSAP | SRSF3 |  |
| TMEM47 | TAF1L | SEC24D | ATP1B3 |  |
| CHP2 | TAF6 | NEFL | GRM2 |  |
| EDRF1 | TANC2 | CLCA3A2 | PPP1R21 |  |
| CYP1B1 | TAOK2 | EIF2AK4 | ARFGEF3 | |
| TBC1D8B | TBC1D23 | TMEM30A | MCCC2 |  |
| GHR | TBC1D31 | RIMS3 | PPM1H |  |
| ZFP661 | TBC1D5 | KLHL5 | VPS13A |  |
| DYX1C1 | TBL1XR1 | ZFP277 | CNRIP1 |  |
| AU040320 | TBR1 | RCAN3 | DNAJC11 | |
| EYA3 | TBX1 | GM11985 | MYL6 |  |
| SERPINA3F | TCF20 | SOGA1 | LASP1 |  |
| PDRG1 | TCF4 | RAB6A | TRIM28 |  |
| TMA16 | TCF7L2 | GOLGA4 | SPR |  |
| FAM213B | TECTA | PNISR | GART |  |
| TRIB1 | TERF2 | ADIPOR2 | EIF4G1 |  |
| SORCS1 | TERT | TIE1 | RPS9 |  |
| GATAD2A | TET2 | HNF1A | MTCH1 |  |
| GJE1 | TGM3 | ARPP21 | CLASP1 |  |
| EPB4.1 | THBS1 | S1PR2 | PSMD11 |  |
| FGF5 | TLK2 | MRPL45 | ENPP6 |  |
| CH25H | TM4SF19 | ITPR2 | ECHS1 |  |
| CXCR6 | TM4SF20 | LAD1 | FARSA |  |
| CARTPT | TMLHE | ITGB1 | VPS26B |  |
| EBAG9 | TERB2 | TSPAN12 | VWA8 |  |
| LIPO2 | TNIP2 | TMEM132E | PGP |  |
| S1PR4 | TNRC6B | PARP3 | PLXNB1 |  |
| MAPKAPK2 | TOP1 | TRPC5OS | CADM1 |  |
| ELOVL2 | TOP3B | ADAMTS20 | GRIPAP1 | |
| ATP1B1 | TPH2 | ACSL3 | MARK1 |  |
| GM10650 | TRAPPC6B | PCLO | PPA2 |  |
| KLHL14 | TRAPPC9 | RASGRF2 | XPO5 |  |
| BC052040 | TRIO | TMED9 | PSIP1 |  |
| SLC35G3 | TRIP12 | EFCAB1 | NDUFA2 |  |
| PCDH11X | ST8SIA2 | PDLIM4 | GMFB |  |
| KCNQ2 | STK39 | TRAIP | COTL1 |  |
| 4930451C15RIK | STYK1 | ARID1B | ACOT13 |  |
| GNAT1 | SYNCRIP | CACNG5 | NDUFAB1 | |
| HMGCS2 | SYT1 | ATP1A3 | ALDH1B1 | |
| KCNB2 | SYT17 | PRR14L | ARMC10 |  |
| TGM6 | SYT3 | PAK7 | RNMT |  |
| ST18 | TBC1D7 | DIP2C | SACM1L |  |
| FND3C2 | TBL1X | CKAP4 | SRR |  |
| KBTBD12 | TDO2 | TUNAR | ADAM23 |  |
| FMOD | TH | MYH3 | ATP6V1G2 | |
| RXFP2 | THAP8 | BMPR1B | STUB1 |  |
| GM6483 | THRA | E2F1 | EIF4H |  |
| YWHAZ | TMEM231 | PYGO2 | GABBR1 |  |
| ARMC4 | TNN | LY6H | PSMD13 |  |
| OLFR558 | TOMM20 | CNIH2 | CELF2 |  |
| MUC6 | TPO | TPM1 | CACNA2D3 | |
| PLBD2 | TRAF7 | SYVN1 | ILF3 |  |
| ERICH1 | TRIM33 | MFAP3 | PSMA7 |  |
| CYP2S1 | TRPC6 | TSNAX | COX7A2 |  |
| TRIAP1 | TRPM1 | CDH24 | SCN1A |  |
| SGIP1 | TSC1 | EPS8 | OSBPL8 |  |
| WNT16 | TSC2 | NOVA2 | SAFB |  |
| RCOR1 | TSHZ3 | LPGAT1 | ANP32A |  |
| FBXO25 | TSN | D2HGDH | MGLL |  |
| ZFP618 | TSPAN17 | ST8SIA4 | HPRT1 |  |
| GGACT | TSPAN7 | PRDX6 | EEF1A1 |  |
| DNAJA1 | TTC25 | SPTB | NTRK2 |  |
| STIM1 | TTI2 | HNRNPU | HSPA1B |  |
| EXOC6 | TTN | SLC47A2 | RPL13A |  |
| CHGB | TUBGCP5 | FZD4 | ADSSL1 |  |
| HSF5 | TYR | FABP7 | ATXN10 |  |
| CCDC82 | UBA6 | MECP2 | SLC6A1 |  |
| OLFR1133 | UBE2H | TLR8 | SLC6A11 |  |
| BMP4 | UBE3A | SYS1 | SLC2A3 |  |
| IP6K3 | UBE3B | TNKS | RAB21 |  |
| GPKOW | UBE3C | ANKFY1 | ECI1 |  |
| TEX13 | UBL7 | EPO | 2-Sep |  |
| RARB | UBN2 | FHAD1 | CFL2 |  |
| TAF8 | UBR5 | KIF16B | DBT |  |
| ESP3 | UBR7 | NCOR2 | RAB10 |  |
| CYP11B1 | UCN3 | CAMK2G | RAB3C |  |
| RAB15 | UNC13A | KRT80 | RAN |  |
| OLFR159 | UNC79 | WASF1 | RPL23 |  |
| TMEM186 | UNC80 | MGL2 | GNAS |  |
| 43525 | UPB1 | C130074G19RIK | GABRB2 |  |
| MS4A4C | UPF2 | CCDC146 | CHL1 |  |
| NPNT | UPF3B | MAP7 | G3BP2 |  |
| F10 | TSPOAP1 | MFAP1B | ANK1 |  |
| LRRC3B | USH2A | DLL4 | SCG2 |  |
| 7530416G11RIK | USP15 | BORA | FAHD2 |  |
| FAR1 | USP45 | AS3MT | RAP1GAP2 | |
| RNF222 | USP7 | NOTCH1 | SERPINB6 | |
| SLC39A10 | USP9Y | CCDC172 | ASNS |  |
| LIPC | VASH1 | PROKR2 | HCFC1 |  |
| GM28048 | VIL1 | GBP7 | CD47 |  |
| KRTAP21-1 | VLDLR | JCAD | PCBP2 |  |
| TRPC1 | VPS13B | LCA5 | NDUFA4 |  |
| 4930430F08RIK | VRK3 | SCN9A | NPTX1 |  |
| ZC3H12D | VSIG4 | RAB27A | EIF4G2 |  |
| NKX1-2 | WAC | OPRK1 | VAT1 |  |
| GM10518 | WDFY3 | STRADB | CRK |  |
| SCARA5 | WDR26 | HDAC9 | KIAA1549 | |
| TYRP1 | WDR93 | BHLHE22 | USP7 |  |
| SYCE2 | WNK3 | KRT73 | NAP1L4 |  |
| GPR22 | WNT1 | CYP2E1 | TRIM46 |  |
| ST3GAL4 | WNT2 | LGALS9 | GABBR2 |  |
| ZFP964 | WWOX | CFAP52 | RIMBP2 |  |
| SIDT1 | UTRN | RGS5 | PPFIA2 |  |
| FAM26D | VDR | FGGY | SRRM2 |  |
| TRPV3 | VIP | SNED1 | FLNA |  |
| KDM4C | WASF1 | ANKRD33B | FAM126B | |
| PIP5K1C | XIRP1 | CIDEA | PDE10A |  |
| ZBTB43 | XPC | KRT7 | ACAT2 |  |
| ZCCHC7 | XPO1 | BICRA | DCTN4 |  |
| SLC38A8 | YTHDC2 | EPB41L4B | TRIM32 |  |
| ZFP108 | YWHAE | MYRIP | VPS50 |  |
| MAGI1 | YY1 | RASGRP1 | LIN7A |  |
| SEC61A2 | ZBTB16 | PDE4D | APPL1 |  |
| KLHL13 | ZBTB20 | ARHGEF10 | IGSF8 |  |
| PARM1 | ZC3H4 | PCDHB10 | GDAP1L1 | |
| EPM2A | ZMYND11 | LZTS1 | FUBP1 |  |
| ANKRD11 | ZNF18 | HMMR | FDPS |  |
| ANO6 | ZNF292 | GFRA2 | SF3B3 |  |
| DUSP9 | ZNF385B | DCLK2 | MPI |  |
| KCTD10 | ZNF462 | HCK | CMAS |  |
| NOC3L | ZNF517 | BACE2 | NAMPT |  |
| PWWP2A | ZNF548 | LEFTY1 | TNPO2 |  |
| RINT1 | ZNF559 | ERBB4 | ARHGDIA | |
| NRADD | ZNF626 | BBS2 | HNRNPA0 | |
| HLCS | ZNF713 | RNPEPL1 | FAM213A | |
| CDK14 | ZNF774 | AGPS | ITPA |  |
| ZFP72 | ZNF8 | PYROXD2 | PGD |  |
| VAPB | ZNF804A | TMCC2 | NDUFA8 |  |
| ZFP760 | ZNF827 | PDZRN3 | PAICS |  |
| ALOX8 | ZSWIM5 | MSRB3 | MECR |  |
| GM16039 | ZSWIM6 | MFRP | TOLLIP |  |
| HSDL2 | ZWILCH | FNDC3B | ACTR10 |  |
| FOXO4 | YEATS2 | LARS2 | ECI2 |  |
| C1QTNF1 | ZNF407 | OLFR1392 | DMTN |  |
| RBMX |  | DMGDH | AP2S1 |  |
| FOSL2 |  | ID3 | DLGAP4 |  |
| GM10735 | | EXT1 | CAD |  |
| HSPA12B | | MAN1A | CAPN5 |  |
| MYCL |  | NAXD | UBE3A |  |
| H2-T22 |  | GNB5 | FAAH |  |
| OLFR1393 | | COX18 | API5 |  |
| XKR4 |  | GM17087 | COPB2 |  |
| ACTR6 |  | CDADC1 | PPT1 |  |
| ZBTB12 |  | H2-T10 | HEXB |  |
| ZFP366 |  | OSGEPL1 | CST3 |  |
| SCML2 |  | SFMBT2 | PCMT1 |  |
| GNPDA2 |  | ATP2B3 | PPIB |  |
| KLHL38 |  | SERPINB6E | 4-Sep |  |
| TMPRSS11F | | HNRNPA0 | DBI |  |
| ZFP874B |  | DLGAP3 | ITGAV |  |
| OLFR912 |  | MYCL | NEDD4 |  |
| D3ERTD751E | | FARP1 | PDXP |  |
| VMN2R84 | | FGL2 | PCP4 |  |
| TSEN2 |  | STARD3 | GNG3 |  |
| TMEM44 |  | PTPRH | EPHA4 |  |
| CHPT1 |  | PTPRS | HSPG2 |  |
| PILRA |  | MPDZ | CAB39 |  |
| WDR37 |  | TRIM67 | KCNMA1 | |
| FAM71F2 | | SYT10 | IQSEC3 |  |
| CKAP5 |  | SNTB2 | EXOC5 |  |
| TRPM4 |  | KDM6B | PUF60 |  |
| FGFR1 |  | MST1 | CDC42BPA | |
| F2R |  | GM10300 | BRSK1 |  |
| FAM124B | | B4GALT4 | HARS |  |
| KLHL31 |  | CALD1 | ABCB7 |  |
| 4930451G09RIK | | TMEM74 | ACTR3B |  |
| OSBPL8 |  | RIPK3 | PKP4 |  |
| ZFP560 |  | EXOSC1 | SLC25A23 | |
| ZFP59 |  | ZHX2 | ECPAS |  |
| MICU2 |  | INHBA | DCLK2 |  |
| WNK2 |  | KLHL2 | EFR3B |  |
| SNX27 |  | RBFOX3 | PICALM |  |
| ALCAM |  | WDR1 | ADGRL1 |  |
| ADAMTSL1 | | MED30 | MFN2 |  |
| SHB |  | TXK | MTDH |  |
| PLEKHH2 | | FBXO4 | BCAS1 |  |
| CSDE1 |  | NPNT | WDR48 |  |
| RNF215 |  | LGI1 | ZC2HC1A | |
| VMN2R85 | | ZFYVE16 | TARSL2 |  |
| HDAC8 |  | ZDHHC17 | DOCK3 |  |
| ADH4 |  | ZFP503 | STXBP5 |  |
| PRPS1L3 |  | PARP4 | GSPT1 |  |
| TIMP2 |  | ZFP36L1 | SCCPDH |  |
| GPR27 |  | ZC3H8 | EIF3C |  |
| MLLT3 |  | FOXP2 | AP3M2 |  |
| ZFP719 |  | A630095N17RIK | ACTR1B |  |
| PODXL |  | PAK6 | ACSF2 |  |
| GALNT16 | | SPRY1 | CHCHD6 |  |
| OLFR658 |  | UBN1 | SFXN1 |  |
| G3BP2 |  | APOPT1 | HAGH |  |
| RNF182 |  | BRD9 | VWA5A |  |
| GABARAPL2 | | HEPHL1 | ABHD12 |  |
| MOB1B |  | RMC1 | DHX30 |  |
| PCDHB8 |  | MTPAP | ARPC5 |  |
| ZFP69 |  | H2-AB1 | SUGT1 |  |
| KCNS1 |  | RAB5C | RPS19 |  |
| GM12355 | | RNF123 | PSMD12 |  |
| MED20 |  | SPN | INPP4A |  |
| FAM126B | | NIM1K | PRMT1 |  |
| ADAP2 |  | RBM47 | ALDH9A1 | |
| DAPP1 |  | MSL3L2 | ARPC3 |  |
| OAS1A |  | BC030867 | PIN1 |  |
| STXBP5L | | FST | TRIM3 |  |
| NEURL1B | | PDE4B | FARSB |  |
| AGBL1 |  | BAG5 | NOS1 |  |
| TRPV5 |  | PEG3 | UBA2 |  |
| CCDC39 |  | GALNT11 | USO1 |  |
| CMTM1 |  | PLCZ1 | TSPAN7 |  |
| SESTD1 |  | IL17D | TANC2 |  |
| CAP2 |  | TIMP2 | TTC7B |  |
| D930020B18RIK | | TAX1BP1 | FARP1 |  |
| ZFP65 |  | PLLP | EXOC7 |  |
| HENMT1 | | TTC39B | PLD3 |  |
| CINP |  | FRAS1 | NUDC |  |
| GM10330 | | CTU1 | TIMM44 |  |
| CLEC4A2 | | SOWAHA | COPS5 |  |
| NSUN7 |  | CACNA1B | GUCY1B1 | |
| A930011G23RIK | | MAST1 | EEF1B |  |
| STARD8 |  | ARAP2 | MTX2 |  |
| CSPP1 |  | TNNI1 | LANCL1 |  |
| 2410004P03RIK | | TIMP4 | CTSB |  |
| FAM69A |  | SEC1 | HSPA2 |  |
| CBFA2T2 | | APBB1 | PTPRA |  |
| PIK3CD |  | CNBP | BSG |  |
| 1700074P13RIK | | VSP35L | PRKCA |  |
| SLC7A7 |  | UFD1 | CBX3 |  |
| AKAP7 |  | CSMD3 | MSN |  |
| APOL10B | | DUSP11 | PRKCD |  |
| D5ERTD577E | | SYNDIG1L | PTPN11 |  |
| NRCAM |  | VPS33A | ACADM |  |
| EIF3A |  | H2-M5 | CRYZ |  |
| SLC24A2 |  | CNTN2 | CRKL |  |
| PATL1 |  | CDKN2AIPNL | RPL13 |  |
| GM8356 |  | GABRR2 | RAD23B |  |
| KCNJ5 |  | LYPD1 | TPM1 |  |
| GALNT11 | | VTN | DOCK4 |  |
| BVES |  | SLAMF1 | RUVBL1 |  |
| LEMD2 |  | ESRRG | EIF3E |  |
| 1700026D08RIK | | GM38394 | RPS13 |  |
| PLN |  | MOS | DNAJA1 |  |
| SERAC1 |  | KLRE1 | RPS10 |  |
| PKIA |  | SULT4A1 | STAM |  |
| PATE2 |  | TBC1D30 | SPARCL1 | |
| PPM1L |  | ROBO3 | VPS45 |  |
| U2SURP |  | CASKIN2 | ARVCF |  |
| SYMPK |  | ARMC3 | G6PDX |  |
| FAM149B | | LRRC74B | GMPS |  |
| 4932438H23RIK | | PRICKLE1 | VPS51 |  |
| ERO1L |  | SORCS1 | SPAG9 |  |
| NUTF2 |  | TMEM136 | ERMN |  |
| RAB2A |  | RPS2 | PPP1R1B | |
| STOML3 |  | TOX2 | PAFAH1B2 | |
| F830016B08RIK | | COL14A1 | SNTA1 |  |
| BHLHE41 | | DGAT1 | PDE1A |  |
| MUL1 |  | S100A7A | KIF5B |  |
| KLRA10 |  | FASN | TFRC |  |
| SLC22A13 | | EOGT | CNTN4 |  |
| RYR3 |  | FAM104A | NLGN2 |  |
| PCSK2 |  | VMP1 | NWD2 |  |
| SMPD3 |  | SERPING1 | CNOT1 |  |
| XPR1 |  | CKB | RPL10 |  |
| GMFB |  | CCDC71L | ERLIN2 |  |
| SMS |  | PTPN18 | SLC6A17 |  |
| STK24 |  | GABARAPL2 | CKAP4 |  |
| NR2C1 |  | SUPV3L1 | DIP2A |  |
| NID1 |  | MZB1 | PGAM5 |  |
| ZFP612 |  | CYS1 | USP47 |  |
| ZFP488 |  | HERC3 | CAMKK2 | |
| GM14214 | | ADGRG1 | EXOG |  |
| GM12830 | | NETO2 | VPS53 |  |
| SIM2 |  | OLFR1418 | BCAS3 |  |
| NKRF |  | GM26566 | KATNAL1 | |
| TMEM71 |  | CLCA4B | PSPC1 |  |
| NKAIN3 |  | TMEM50A | CLPTM1 |  |
| IFT57 |  | BNIP3 | PIK3R4 |  |
| NAV3 |  | SCN2B | EIF4A3 |  |
| GLP2R |  | CCDC113 | NLN |  |
| PRRC2C |  | SLC22A1 | PDK3 |  |
| CLPTM1L | | ADAM11 | 2-Mar |  |
| FGF3 |  | HIST1H4K | KARS |  |
| RAB33B |  | PCDH7 | RPL17 |  |
| UHRF1BP1 | | MAL2 | TRAP1 |  |
| RIOK3 |  | NDNF | CENPV |  |
| ITGA4 |  | WSB2 | LRRC57 |  |
| ANKRD23 | | GJD4 | CUL2 |  |
| HIST1H2BM | | ALDH7A1 | DUSP3 |  |
| FAM184B | | LATS2 | RPN2 |  |
| NT5C |  | ASCC2 | STX12 |  |
| IGSF6 |  | NLRP1B | ARHGEF7 | |
| RNF125 |  | APPL2 | NCKIPSD | |
| ADIPOQ |  | RGS7BP | ADD3 |  |
| TCEA1 |  | SORBS1 | GPC1 |  |
| EIF2S1 |  | WNT7A | NUMB |  |
| FAM83B |  | UACA | PSMA1 |  |
| ELAVL3 |  | ZKSCAN2 | LYPLA2 |  |
| NEBL |  | AK4 | ARL3 |  |
| GNAT3 |  | TCOF1 | EHD1 |  |
| VMN2R87 | | MMS19 | MAGI2 |  |
| ENAM |  | DAPK1 | UHRF1BP1L | |
| KCNQ5 |  | FAAP24 | GNG13 |  |
| PRSS41 |  | ZFP459 | PTEN |  |
| SLC6A2 |  | WNT6 | CUL4B |  |
| HNF4A |  | AFAP1L1 | MAP7D1 |  |
| MRRF |  | SLC31A1 | CLTA |  |
| FAM49A |  | HIST1H1C | MYH11 |  |
| NXPE5 |  | ASB2 | DDOST |  |
| RAP1A |  | PDGFRA | COPS3 |  |
| FAM163B | | RIN1 | GDAP1 |  |
| PLEKHG4 | | ZFP516 | RBBP9 |  |
| FSTL1 |  | MCTP1 | APP |  |
| C130079G13RIK | | CDH6 | FECH |  |
| KCNE1 |  | LMNA | KIF3A |  |
| ADAMTS1 | | SELENOF | CTNND1 |  |
| S100A7A |  | POPDC3 | POR |  |
| AK5 |  | WNT9A | TFAM |  |
| SLC7A11 |  | PDLIM1 | ALDH3A2 | |
| ASCL4 |  | URI1 | CDK5 |  |
| AVPR1B |  | CD22 | HSD17B4 | |
| BTBD11 |  | DPY19L1 | LRPAP1 |  |
| FOXD4 |  | INAFM1 | EEF1D |  |
| CSNK1D |  | HIST1H2BB | PTPA |  |
| AOC1 |  | PPP2R2C | CDC42 |  |
| SLMO1 |  | BTG2 | RPS18 |  |
| CARHSP1 | | GABRD | RPL18A |  |
| BEND4 |  | KCNQ1 | RPS6 |  |
| CDKN1B |  | 9530003J23RIK | MTPN |  |
| FAM101B | | CSPG4 | RPL30 |  |
| PLCE1 |  | TRAK1 | RPL8 |  |
| NLGN1 |  | DPF3 | TPT1 |  |
| GLCCI1 |  | PKD2L1 | HMGB1 |  |
| ZFP74 |  | IFI44 | RASGRF2 | |
| 43528 |  | ASB6 | WBP2 |  |
| SERPINB9 | | IREB2 | RPLP2 |  |
| PRMT5 |  | HELZ2 | NUCB1 |  |
| NWD2 |  | DENND5B | OSBP |  |
| PBRM1 |  | GDF11 | ROGDI |  |
| CDC42EP4 | | 4-Sep | MYL12B |  |
| PIK3R3 |  | ANKRD13D | MAT2A |  |
| SLFN5 |  | RTF2 | DAB2IP |  |
| BHMT |  | CHST8 | SNX27 |  |
| SFXN5 |  | NTSR2 | CDKL5 |  |
| GATSL2 |  | CYBB | SPECC1 |  |
| B3GNT2 |  | TGFB3 | LAMB2 |  |
| ANAPC1 |  | DNAH7A | KTN1 |  |
| GMPS |  | SLC35F6 | PLCG1 |  |
| SLC10A7 |  | SLC6A13 | SH3GL3 |  |
| ZFP78 |  | AMOTL2 | DGKB |  |
| OGT |  | PGGHG | DAGLA |  |
| ACKR4 |  | FLYWCH1 | ACAP2 |  |
| CHRM4 |  | BCL10 | NISCH |  |
| AGTR2 |  | STARD10 | UBAP2L |  |
| CANX |  | NHS | C2CD2L |  |
| A1CF |  | CLSTN2 | FLNB |  |
| SH3RF3 |  | DISP3 | DDX42 |  |
| TMEM8B | | FRMPD3 | ABLIM2 |  |
| D130040H23RIK | | TRIO | NCEH1 |  |
| B3GALT2 | | PRICKLE2 | CAMK1D | |
| C1QTNF7 | | ENOX1 | PEX5L |  |
| AMER1 |  | RNF225 | EMC1 |  |
| LPIN3 |  | BRD4 | UBA3 |  |
| RNF6 |  | HPRT | TCERG1 |  |
| ATCAY |  | NFAT5 | SYN3 |  |
| SPOCK3 |  | TBC1D23 | CPNE5 |  |
| LRP10 |  | MDGA2 | NAXE |  |
| WDR19 |  | PTPRO | KIAA0513 | |
| VWDE |  | WSCD2 | WDR13 |  |
| TPH2 |  | CCDC93 | ASL |  |
| ADRA1A |  | MRC1 | PTBP2 |  |
| ACTR2 |  | RIMKLA | GRHPR |  |
| BCL7C |  | KLHL10 | MPST |  |
| AI464131 |  | GABRE | HGS |  |
| GALNT12 | | STPG3 | SF3B1 |  |
| SPACA6 |  | IDS | GLO1 |  |
| LPAR1 |  | ATP6V1E1 | RTRAF |  |
| VMN2R86 | | PCDH17 | NDUFB7 |  |
| TCSTV1 |  | PRDX5 | CHCHD3 |  |
| TACR3 |  | CTSB | ATP6V1F | |
| POLR3B |  | SNX20 | MOBP |  |
| ZFP119A |  | 3830417A13RIK | SNX5 |  |
| DCLK1 |  | EIF3J2 | GNG12 |  |
| GJD2 |  | SLC25A22 | ACADSB |  |
| CEBPZ |  | DPYSL5 | NEBL |  |
| GM14151 | | FAM71E1 | USP14 |  |
| IPO8 |  | IQSEC3 | TXNRD1 |  |
| ACAD12 |  | ACAD12 | DRG2 |  |
| CD97 |  | CSF3 | PSMB3 |  |
| H6PD |  | SRGAP2 | RUVBL2 |  |
| SYNCRIP | | SLC39A12 | TENM2 |  |
| LSAMP |  | BAIAP3 | PFDN5 |  |
| ZFP46 |  | CPT2 | DCTN3 |  |
| STC1 |  | TGFBR2 | BAG6 |  |
| NR2E1 |  | ARSJ | STRAP |  |
| WFDC8 |  | CHN1 | PLPBP |  |
| ALDOB |  | LMBRD1 | RPS15 |  |
| FAM20B |  | FSIP2 | ATP5J |  |
| HS3ST3B1 | | GM14419 | DIAPH1 |  |
| GNAO1 |  | ZFP800 | PSMB1 |  |
| SDK1 |  | CLIC6 | DDT |  |
| GPR179 |  | SMO | PSMD4 |  |
| DIRAS2 |  | NAIP5 | EXOC4 |  |
| RGS14 |  | ARHGAP21 | ASNA1 |  |
| FCRL6 |  | EMID1 | CACNA1B | |
| RHPN2 |  | RPH3AL | SNX3 |  |
| LRIT2 |  | IL6RA | DYNC1I2 | |
| TCSTV3 |  | TGFBR3 | ZFR |  |
| AKT2 |  | OLFR116 | BIRC6 |  |
| ZUFSP |  | PDIA3 | APOE |  |
| GAREM |  | IBA57 | PTMA |  |
| B3GLCT |  | TRPM1 | SCP2 |  |
| SH3BGRL2 | | RAB15 | RPL12 |  |
| MAGEE2 |  | EID1 | RPL28 |  |
| EEA1 |  | GM9949 | HIST1H1B | |
| ST3GAL1 | | ZSWIM7 | RANGAP1 | |
| ARL14 |  | TFAP2A | CRAT |  |
| FANCG |  | TRIM16 | PPM1A |  |
| PTH2R |  | GALNT18 | PSMA2 |  |
| NRG3 |  | ARHGAP39 | RPL10A |  |
| RAP2A |  | ADARB2 | ERP29 |  |
| LRRC49 |  | MNDAL | TNKS1BP1 | |
| IRS3 |  | C330021F23RIK | ABCE1 |  |
| ZFP738 |  | ATP13A4 | VBP1 |  |
| NPHS2 |  | ANKRD66 | RPS25 |  |
| SPRR2A2 | | NWD1 | PTPRN2 |  |
| TGM3 |  | MYPN | ANXA11 |  |
| APLF |  | MFHAS1 | CLU |  |
| OLFR722 |  | GPATCH4 | TRAPPC9 | |
| CYP2C65 | | RNF185 | CMPK2 |  |
| EIF1AX |  | APOD | PLCL1 |  |
| CEBPA |  | COL6A2 | TBC1D24 | |
| SRSF3 |  | TMEM81 | DDX17 |  |
| 9530003J23RIK | | ECE1 | PFAS |  |
| CLDN1 |  | GM28729 | GAS7 |  |
| GM8909 |  | 1700003F12RIK | PAK3 |  |
| CLIC5 |  | HEXIM2 | ELAVL4 |  |
| RPS6KA6 | | HMGB1 | SH3GL1 |  |
| AMBN |  | GALR1 | HEPACAM | |
| PPP1R2 |  | CIPC | PLPPR4 |  |
| ZFP385C |  | HCN1 | PDPR |  |
| GABRA6 |  | NDFIP2 | NAA15 |  |
| KAZN |  | NKD2 | MOGS |  |
| FREM3 |  | GBP3 | LARS |  |
| METTL16 | | LRRC3B | PALM2 |  |
| REEP6 |  | PPT2 | IARS |  |
| GM4477 |  | MGLL | MAP1S |  |
| TNFSF10 |  | ZFP639 | PREPL |  |
| RFX3 |  | SHISA3 | CSNK1G3 | |
| SLC35A5 |  | AKR1C14 | UBA6 |  |
| MAF |  | IMMP2L | TCEAL5 |  |
| SEMA3A |  | SERBP1 | ACAD9 |  |
| SUPT16 |  | SLC28A3 | PNPT1 |  |
| HOXA7 |  | TMEM30B | ATAT1 |  |
| TMEM18 |  | HPSE | PITRM1 |  |
| RRAGB |  | AR | HIBCH |  |
| ANKMY1 | | RSPH9 | RAB11FIP5 | |
| RSAD1 |  | CBR3 | RNPEP |  |
| IMPG1 |  | PPM1K | IPO9 |  |
| FRMD8 |  | RNF169 | TARDBP |  |
| VANGL1 |  | DOK4 | PDIA6 |  |
| KCNS2 |  | FAM107A | SFXN5 |  |
| TRIM9 |  | BCLAF1 | SEC14L2 |  |
| D3ERTD254E | | 10-Sep | STOML2 |  |
| CCDC153 | | FBXW15 | GGT7 |  |
| FTO |  | MED14 | STK24 |  |
| CYP2B19 | | IPMK | DHRS1 |  |
| TNRC18 |  | BLZF1 | GRPEL1 |  |
| RIT2 |  | SRPK2 | PCYOX1 |  |
| PDZD7 |  | GSTO1 | NDUFB9 |  |
| 1700025G04RIK | | HIST1H1D | TPPP3 |  |
| RASL11B | | CALHM2 | MICU3 |  |
| GDF2 |  | RIPOR2 | NDUFS4 |  |
| 2010107G23RIK | | ITIH3 | PGM3 |  |
| DDX23 |  | NEPRO | NAXD |  |
| WDR72 |  | FUT11 | NSFL1C |  |
| GM8369 |  | ZFP946 | RAB3B |  |
| LYSMD4 |  | PDZD3 | SERPINB1A | |
| CHRDL1 |  | PEBP4 | ACP1 |  |
| NAP1L5 |  | OSBPL3 | OCIAD2 |  |
| ZDHHC25 | | OSBPL6 | BTBD17 |  |
| INPP5B |  | FAM160B2 | PRKAR1A | |
| MIEF1 |  | UAP1L1 | RANBP2 |  |
| FAT3 |  | 9130409I23RIK | FLII |  |
| ZFP26 |  | CLEC18A | PDK2 |  |
| VMN1R45 | | EVI2A | ACSS2 |  |
| OPRL1 |  | ACYP1 | PCSK1N |  |
| GM9747 |  | RASL11A | DNAJA2 |  |
| CD276 |  | ABCB9 | VPS29 |  |
| ACBD5 |  | SLC27A3 | UBQLN2 |  |
| ALOX15 |  | PRSS36 | ESD |  |
| HOXA9 |  | RNF168 | PSMA4 |  |
| FAM120B | | PDGFC | NFS1 |  |
| SLC2A3 |  | GABRA2 | RGS6 |  |
| EIF4A1 |  | KCNJ6 | CAMSAP1 | |
| NEFL |  | TMEM216 | RABGAP1 | |
| FV1 |  | GM21958 | SEC22B |  |
| SPTLC3 |  | RHOU | ATPIF1 |  |
| ENTPD6 |  | METAP1D | PSMD14 |  |
| UBAP1 |  | TBCK | NIPSNAP2 | |
| PTER |  | SMG1 | ATP9A |  |
| EIF4A3 |  | GM10944 | HMOX2 |  |
| GPR35 |  | SPRY4 | HCN2 |  |
| CACHD1 |  | PLCH2 | MPP3 |  |
| HRASLS |  | DCDC2A | LAMC1 |  |
| CBLB |  | CHST4 | SERPINA1A | |
| PCMTD2 |  | B4GAT1 | CDH2 |  |
| TMEM131 | | ZFP386 | GNA11 |  |
| TCERG1L | | HIST1H2BF | GABRG2 |  |
| CLDN15 |  | TMEM8B | PNP |  |
| SPRY4 |  | SARDH | BRAF |  |
| ASB7 |  | ZFPM2 | GNB4 |  |
| PHF11D |  | PPAN | PTK2 |  |
| GBP9 |  | RAB11FIP4 | GRIN2A |  |
| LMO3 |  | ST8SIA3 | RIDA |  |
| WDFY1 |  | IRAK1BP1 | DDX6 |  |
| DNLZ |  | ASS1 | VPS33B |  |
| TMPRSS11D | | FOXD2 | ARHGAP39 | |
| CAMK2N1 | | NYAP1 | ARPC4 |  |
| C5AR2 |  | ZFP383 | NRGN |  |
| SERPINA3G | | STARD13 | RPL26 |  |
| 43720 |  | HES1 | RPS7 |  |
| NRXN3 |  | MAGEE2 | RPS8 |  |
| WDR90 |  | SMAP1 | HNRNPH2 | |
| ARHGEF37 | | ZFP770 | HINT1 |  |
| ACSS1 |  | FAM167A | PSME1 |  |
| ALDH8A1 | | EIF4E2 | PSMB4 |  |
| FAM219A | | USP18 | NME2 |  |
| BYSL |  | BEND5 | MARK2 |  |
| PDZRN3 |  | KIF17 | INF2 |  |
| OLFR536 |  | AMOT | ESYT2 |  |
| RPN2 |  | SLC52A3 | PLXND1 |  |
| GM10615 | | MORN5 | EDC4 |  |
| GABRG3 |  | IER2 | IQGAP2 |  |
| N6AMT1 |  | ZHX3 | ARHGAP44 | |
| I830077J02RIK | | SEC14L4 | RABL6 |  |
| GLRA2 |  | DRD1 | PPP2R5C | |
| USHBP1 |  | OTP | TSG101 |  |
| WNT10A |  | MTCL1 | DDX19A |  |
| IQUB |  | MKNK1 | SNX6 |  |
| RNF152 |  | RNF217 | CACNA2D2 | |
| ARID4B |  | ADAMTS7 | SDK2 |  |
| ZCCHC14 | | GFAP | SLC44A1 |  |
| LRRC28 |  | RGS14 | CSPG5 |  |
| TRP53 |  | ACTG2 | MYCBP2 |  |
| BIVM |  | HCFC1 | RAB3GAP1 | |
| HOXB2 |  | LZTS2 | EPN1 |  |
| MYB |  | CCDC117 | THNSL1 |  |
| ZFP251 |  | GRIN2B | DOCK9 |  |
| HSPA1A |  | GNAS | AHSA1 |  |
| CD47 |  | CSF3R | RCN2 |  |
| STXBP4 |  | CYB5A | SCFD1 |  |
| XKR6 |  | HDGF | HOOK3 |  |
| NOX4 |  | TRIM9 | PTGES2 |  |
| 4632404H12RIK | | RPL3 | NLGN3 |  |
| ACTN4 |  | RGS8 | CORO2A |  |
| SLC16A5 |  | EIF1AD | GNAL |  |
| TADA2B |  | MAST4 | ALDH4A1 | |
| FAM107A | | HIST1H1A | EPN2 |  |
| HMGCLL1 | | INSL5 | PRMT5 |  |
| ADAM9 |  | CLSPN | SCAMP1 |  |
| KCNA1 |  | PAPPA2 | WDR11 |  |
| IRF4 |  | GSDMD | HACD3 |  |
| TRMT44 |  | OASL1 | AGFG1 |  |
| DRAM2 |  | PRKCD | BPHL |  |
| ADH7 |  | BMP4 | CCAR2 |  |
| CBX5 |  | SASH1 | ARL8A |  |
| ABCB7 |  | COL5A1 | EPM2AIP1 | |
| CEACAM18 | | ZDHHC14 | TMEM30A | |
| ALG11 |  | F830016B08RIK | CACNG8 |  |
| PHC3 |  | PCDHB7 | VPS11 |  |
| GM8923 |  | GADD45A | SNX4 |  |
| RCAN3 |  | HERC6 | SRGAP2 |  |
| MPPED2 |  | ARRDC3 | SNCB |  |
| ESP34 |  | NFKBIA | PCYT2 |  |
| NANOS1 |  | TMEM131 | LRRC59 |  |
| GOLPH3 |  | ELP6 | HNRNPAB | |
| KAT6A |  | FAM120C | PSMD6 |  |
| RNF14 |  | FLVCR1 | MAT2B |  |
| PSG26 |  | GM6525 | PPP6C |  |
| CADM1 |  | OCIAD2 | TECR |  |
| SAA4 |  | GAD2 | SERBP1 |  |
| FMN1 |  | WNT10A | SEC13 |  |
| RASGRP3 | | NKX3-1 | DTNA |  |
| STX16 |  | RALY | NDUFB8 |  |
| SPATA3 |  | PLEKHA2 | NOP56 |  |
| ALX1 |  | ACSS3 | PHPT1 |  |
| PDE1A |  | CCDC112 | ALG2 |  |
| GIGYF2 |  | ASCC3 | AMPD2 |  |
| OLFR389 |  | CERKL | NDUFS7 |  |
| YIPF5 |  | PRSS2 | CYB5R3 |  |
| ITIH5 |  | TMC7 | TSC1 |  |
| FZD7 |  | EDIL3 | LACTB |  |
| ZFHX2 |  | CEP112 | CLSTN1 |  |
| RNF220 |  | RGS6 | CARS |  |
| SELE |  | SQSTM1 | UCHL3 |  |
| TCF4 |  | MT-ND4L | VAPB |  |
| ZFP655 |  | BC034090 | COPG1 |  |
| TBX15 |  | GM42372 | AK4 |  |
| GORAB |  | HTR1B | ALDH18A1 | |
| SLC4A4 |  | ABHD12 | ABHD16A | |
| PROX2 |  | NDUFB8 | TCOF1 |  |
| SLA |  | NRIP2 | SRSF2 |  |
| MBTPS2 |  | SMARCA2 | RPL24 |  |
| 4933402N03RIK | | GDAP2 | RPL27 |  |
| NAB2 |  | L3HYPDH | MT3 |  |
| OPALIN |  | FOXP4 | EXOC6B |  |
| LAMP5 |  | DEPTOR | HSD17B10 | |
| GPR115 |  | DDX19A | NUMBL |  |
| A430105I19RIK | | COL8A2 | KPNA3 |  |
| EHMT1 |  | GLRB | SRPK2 |  |
| SERPINA1F | | GALT | CSNK2A2 | |
| 8030462N17RIK | | IQCA | SLK |  |
| TPMT |  | ZDHHC20 | ATXN2 |  |
| POLK |  | CEP85L | KLC1 |  |
| LZTS3 |  | TG | SUB1 |  |
| UBTFL1 |  | ZIC2 | FABP3 |  |
| HOXC13 |  | UCHL3 | ABCB1A |  |
| URAD |  | FIS1 | CRYAB |  |
| SPATA17 | | SH3PXD2B | BCAT1 |  |
| AK4 |  | BRD8 | CTNNA1 |  |
| SFXN2 |  | D8ERTD738E | U2AF2 |  |
| VMN1R51 | | FN1 | NAP1L1 |  |
| VARS2 |  | H2-T23 | EPHX2 |  |
| PGK1 |  | PRIMPOL | HMGCL |  |
| UBXN2B |  | ADAM1A | GSR |  |
| SPTB |  | RAD52 | TMOD1 |  |
| ERI1 |  | LBP | PTPN5 |  |
| PYCARD |  | PBK | ADK |  |
| GAB2 |  | EML6 | STRN4 |  |
| 6430573F11RIK | | CEBPG | EIF5 |  |
| ATXN3 |  | ABLIM2 | DNAJC5 |  |
| SNAPC2 |  | MRPL15 | SNRPD2 |  |
| TMEM255A | | DKKL1 | ABI2 |  |
| LRRC8C |  | CD38 | DYNLRB1 | |
| UBL3 |  | ASB1 | RPL31 |  |
| TRIM35 |  | CCDC47 | TRA2B |  |
| PIF1 |  | BAMBI | KCNA2 |  |
| NR2F2 |  | FADD | LXN |  |
| LRRC34 |  | GPR17 | MPP1 |  |
| CCDC184 | | RNF165 | CTPS1 |  |
| CIITA |  | TMEM52 | ELOC |  |
| LDHD |  | PAM16 | FHL1 |  |
| CSNK1G1 | | TSPAN13 | PAM |  |
| POLN |  | AZIN1 | TOP1 |  |
| TRPD52L3 | | RRP1 | EML1 |  |
| ADCY9 |  | ZFP420 | CALB2 |  |
| ADARB2 |  | USP6NL | SSRP1 |  |
| ZPLD1 |  | NPR3 | VPS8 |  |
| N28178 |  | PLA2G4B | PHACTR1 | |
| CERK |  | LTBP1 | HDHD2 |  |
| ZFP819 |  | FNIP2 | ADGRB1 |  |
| ZAK |  | UOX | NDUFB6 |  |
| BRIP1 |  | CD164L2 | THEM4 |  |
| CLDN18 |  | GSR | PSD |  |
| ING1 |  | 1110059G10RIK | FAM169A | |
| CPNE8 |  | FBXO34 | PSMB6 |  |
| WDR18 |  | SLC7A15 | CLDN11 |  |
| ZFP260 |  | KCNJ8 | CAPRIN1 | |
| TMPRSS6 | | PTPRC | CDC37 |  |
| MRGPRB2 | | IMPG2 | SNRPA |  |
| GLA |  | ADAMTS8 | NDRG1 |  |
| METTL7A2 | | ABRACL | MAPK3 |  |
| PLXNA2 |  | CCS | AHCYL2 |  |
| RAB3IL1 |  | SEMA3E | TPM4 |  |
| SORBS2 |  | LRIG1 | WDR44 |  |
| RNF185 |  | NDUFB2 | TNPO3 |  |
| SERPINA7 | | CKAP5 | PRMT8 |  |
| IQCE |  | KCND3 | GAPVD1 |  |
| OFD1 |  | AJUBA | PPP2R5A | |
| FAM73A |  | FUNDC2 | SMARCC2 | |
| SLC37A3 |  | CDK5 | PIK3C3 |  |
| CHRM2 |  | FAM13C | SORT1 |  |
| SKINT2 |  | PCP4L1 | DMXL1 |  |
| PYDC3 |  | TACR3 | MAGI1 |  |
| FAT4 |  | MEMO1 | TACC1 |  |
| MAST4 |  | SOCS4 | MLEC |  |
| OLFR550 |  | WNT5A | ROBO2 |  |
| HIST4H4 |  | POLQ | MYBBP1A | |
| ETS2 |  | POU6F1 | KIAA1107 | |
| TMEM245 | | CRIP2 | PPM1E |  |
| KIF3B |  | ZC3H12D | CYLD |  |
| MT-ND5 |  | CCDC184 | DGKZ |  |
| 1110002L01RIK | | ZFP536 | SUPV3L1 | |
| 5031439G07RIK | | NDUFB3 | TIPRL |  |
| RAB12 |  | TIGD4 | TBC1D10B | |
| HIST2H4 |  | GAL3ST2B | PRUNE1 |  |
| NOL4L |  | ALX4 | SGTA |  |
| DDX21 |  | TTC16 | GGA3 |  |
| EML3 |  | GM17430 | FAM120C | |
| ZDHHC21 | | RTP2 | SHISA7 |  |
| GM13011 | | GM5134 | WDR26 |  |
| HMX2 |  | NOBOX | VPS52 |  |
| NDST3 |  | OLFR541 | NEDD4L |  |
| NRK |  | GM5796 | OPLAH |  |
| GPR26 |  | 4930447C04RIK | GFM1 |  |
| DNAJB6 |  | KCNQ5 | CC2D1A |  |
| EPHX3 |  | KLK12 | LGI3 |  |
| ALG6 |  | MTX3 | PITPNC1 | |
| FAM49B |  | RTP4 | SH3KBP1 | |
| ANKRD13C | | NOMO1 | PRPSAP2 | |
| LPIN2 |  | TSGA10 | HIP1 |  |
| KLHDC10 | | RETNLG | LGALSL |  |
| SLC1A2 |  | KIR3DL2 | AGAP3 |  |
| SLA2 |  | NPTXR | WASL |  |
| TFCP2L1 | | CLRN1 | TWF1 |  |
| KLHL32 |  | TMEM87B | AK5 |  |
| 5430427O19RIK | | PARVG | ACAA1A |  |
| NEK10 |  | NECAB1 | RIN1 |  |
| ABHD17B | | CLIC3 | KLHL22 |  |
| 4921517D22RIK | | APOO | PRPF19 |  |
| PGAM1 |  | MTRR | EPDR1 |  |
| KRT33A |  | NDUFA13 | MCCC1 |  |
| FAM167A | | RNF144A | CADM3 |  |
| TACR1 |  | GEMIN4 | RAB5A |  |
| VKORC1L1 | | PGR15L | SNX2 |  |
| SLC25A21 | | PLEKHA8 | ELP3 |  |
| IDS |  | TSSK6 | LINGO1 |  |
| REPS2 |  | SETD1B | EXOC2 |  |
| ZFP382 |  | SYNM | SF3A3 |  |
| GM527 |  | ZFP706 | ACAD8 |  |
| PPIP5K1 |  | VTI1A | APMAP |  |
| PHF11C |  | ESCO1 | DCPS |  |
| PHF13 |  | SEMA4B | SLC25A18 | |
| LANCL2 |  | GM14418 | PPP1R12A | |
| WAPAL |  | HIBADH | TPRG1L |  |
| GM9979 |  | ANXA1 | NDEL1 |  |
| FGD4 |  | CEP19 | ISYNA1 |  |
| TXLNB |  | LDHD | RNF14 |  |
| GM12253 | | OLFR220 | SMPD3 |  |
| SRMS |  | LY6A | NOVA1 |  |
| ADORA3 |  | SLC27A2 | FMNL1 |  |
| LGI3 |  | IL6ST | SEMA7A |  |
| URI1 |  | DPYS | CLIC4 |  |
| HIST1H1A | | BMP7 | ACOT9 |  |
| GUCD1 |  | PTPRB | PSMB2 |  |
| GM5916 |  | POLR3F | SAE1 |  |
| MALL |  | SPINT1 | ADAM11 |  |
| TTC23L |  | DYDC1 | AKAP12 |  |
| LAMP3 |  | NCL | SKP1 |  |
| 1700016D06RIK | | KCNK3 | GSTZ1 |  |
| ORC2 |  | PCGF6 | MTMR7 |  |
| CD164 |  | PPP2R1B | SUCLG2 |  |
| KCNK6 |  | NDUFA1 | DNPEP |  |
| LRRC8D |  | OBSCN | NDUFC2 |  |
| ZBTB22 |  | RETNLB | RPL21 |  |
| ESRRG |  | S100PBP | TMSB4X |  |
| EFCC1 |  | WWOX | SYNGR1 |  |
| ANKRD61 | | BRMS1 | UQCR11 |  |
| RHOU |  | TMEM167 | FRRS1L |  |
| KCNV1 |  | ALAS2 | PLXNB2 |  |
| FAM84A |  | ANXA4 | CCDC6 |  |
| PAG1 |  | LYNX1 | NUMA1 |  |
| SLC52A3 |  | TSLP | ECH1 |  |
| SOHLH2 |  | KLF9 | FKBP8 |  |
| MAPKBP1 | | DNAAF1 | C1QBP |  |
| FAM161B | | RNF113A1 | CD200 |  |
| USP32 |  | IL1B | NCK2 |  |
| PACS2 |  | ZNHIT6 | PIP4K2A |  |
| NFIC |  | ROGDI | EIF3D |  |
| C1QL1 |  | DHDH | HSD17B12 | |
| KANSL1L | | VMN1R26 | SORL1 |  |
| ANO3 |  | MAMDC4 | COPS6 |  |
| PKIB |  | ESRRA | CPD |  |
| ZFP748 |  | ABCB7 | PRNP |  |
| CHRNA3 |  | SH2D2A | DMD |  |
| KDM2A |  | GM5678 | PDCD6 |  |
| RAD18 |  | PGPEP1 | PRKAR1B | |
| ATF2 |  | TOR3A | MUT |  |
| NCOA4 |  | CAPG | GSTA4 |  |
| MTHFSD | | ATOH8 | RASGRF1 | |
| PTRF |  | MROH9 | DRG1 |  |
| ME1 |  | GAREM1 | RAB12 |  |
| 4933425L06RIK | | NEUROD2 | FAH |  |
| ADNP2 |  | IGFBP4 | FMR1 |  |
| TAF1D |  | MVB12B | FKBP2 |  |
| PROX1 |  | SAR1B | ADSS |  |
| ATG12 |  | RAPGEFL1 | NDUFS6 |  |
| TMEM144 | | ERC1 | ACYP1 |  |
| MYBL1 |  | RSRP1 | WFS1 |  |
| MIB1 |  | SMAGP | FUS |  |
| CNIH4 |  | COL22A1 | GABARAPL2 | |
| ACN9 |  | PTPN23 | EIF4A1 |  |
| TIGD3 |  | VASH2 | UBE2D3 |  |
| BHLHA15 | | KRT18 | UBE2K |  |
| RNF165 |  | CCDC14 | RHOB |  |
| KRCC1 |  | COMMD8 | SULT4A1 | |
| SPRYD7 |  | TSPAN6 | RPS17 |  |
| ENDOD1 |  | GNAI3 | CSNK2B |  |
| RGS2 |  | LCK | UBE2L3 |  |
| PRKAR1A | | WDR26 | PRKACB |  |
| MARCKS | | PLCXD2 | ELAVL1 |  |
| HIST1H1D | | CD63 | UQCRH |  |
| D16ERTD472E | | PPM1G | GSK3A |  |
| SUCLG2 |  | ARHGAP10 | PRRC2C |  |
| DCUN1D4 | | IGDCC4 | SCRN3 |  |
| GM26992 | | H2-AA | CCDC136 | |
| HSP90B1 |  | ZBTB16 | TENM4 |  |
| COA6 |  | GRIK4 | PDAP1 |  |
| 1300002K09RIK | | ADAMDEC1 | RMDN3 |  |
| PDE6A |  | SFXN2 | ERMP1 |  |
| FBXL21 |  | PQBP1 | FAM81A |  |
| NMUR2 |  | GM15737 | ARCN1 |  |
| GTF2IRD1 | | GM9817 | SLC27A1 |  |
| TIAM1 |  | TPRKB | RBBP4 |  |
| KDM6B |  | FAM189A1 | GNG7 |  |
| TFAP2B |  | ARTN | KSR1 |  |
| H2-OB |  | DNAJC14 | CACNA1E | |
| UBE2QL1 | | DOK3 | HADH |  |
| SSTR4 |  | GM4070 | KIF3B |  |
| AMMECR1 | | ALDH1L1 | MARS |  |
| ACTA2 |  | CREBBP | BRSK2 |  |
| IGSF23 |  | ARHGAP24 | SMARCA2 | |
| ZFP426 |  | SYTL4 | SEC23IP |  |
| GPD1L |  | UBQLN2 | SRI |  |
| PGBD1 |  | OLFR173 | SIK3 |  |
| AI182371 |  | MYLK4 | BCR |  |
| TMEM119 | | AQP3 | MOB4 |  |
| 1500009C09RIK | | GPANK1 | UBFD1 |  |
| RGS1 |  | ACVR2B | LUC7L2 |  |
| COL12A1 | | CAMTA2 | ELP1 |  |
| GPR137C | | PIK3CB | SCRIB |  |
| GORASP2 | | AKR1C13 | FAM98B |  |
| XPNPEP3 | | BACH2 | RNF214 |  |
| TMEM251 | | FBXL16 | ZADH2 |  |
| MBTD1 |  | COL9A3 | RAB39B |  |
| 4930453N24RIK | | CD3EAP | RBFOX3 |  |
| KLHL28 |  | CTSF | CSNK1A1 | |
| CAMK4 |  | SHISAL1 | MRPS27 |  |
| CCDC38 |  | AP1AR | PLCXD3 |  |
| GM4794 |  | CLEC4A2 | ARMC6 |  |
| S100Z |  | GTF3A | NCS1 |  |
| CACNA1I | | EP300 | STRIP1 |  |
| H2AFY2 |  | C87436 | RBM14 |  |
| ZKSCAN1 | | TGIF1 | AIDA |  |
| SCGB1B27 | | IRGQ | CROCC |  |
| ERRFI1 |  | NPC1L1 | SLC4A8 |  |
| ABL2 |  | MOV10 | SLC25A1 |  |
| DNASE2A | | GPR160 | FNBP1L |  |
| GM1045 |  | VOPP1 | EIPR1 |  |
| PRR5L |  | SYN2 | CBR3 |  |
| PGRMC2 |  | SOWAHB | SF3A1 |  |
| TMTC4 |  | PMF1 | EIF3L |  |
| CAMK2D | | TMEM39B | ABHD6 |  |
| C230062I16RIK | | FBXO10 | VPS18 |  |
| AGPAT4 |  | DHRS13 | EXOC1 |  |
| ANK2 |  | SLPI | PURG |  |
| SLC6A6 |  | MFNG | NAE1 |  |
| FITM2 |  | JAK2 | CAMKK1 | |
| MYADM |  | PEG10 | VPS4A |  |
| TGFA |  | ID2 | RBM39 |  |
| SEC14L2 |  | ARHGAP11A | SETD7 |  |
| SRC |  | TENM4 | ACOT11 |  |
| EPN2 |  | SEBOX | IPO4 |  |
| RSC1A1 |  | ANPEP |  |  |
| CDH2 |  | GM8281 | SLC27A4 |  |
| FOXO1 |  | KLF4 | HDAC11 |  |
| HEG1 |  | TTN | EIF3H |  |
| A630007B06RIK | | BMPR2 | BRINP1 |  |
| CCDC157 | | PLXDC2 | ATAD3 |  |
| ZFP955B |  | PTCH1 | ACY1 |  |
| FRMD3 |  | PPARGC1A | PPIF |  |
| MB21D2 |  | RETNLA | MLF2 |  |
| CRTAP |  | ACTA2 | CDS2 |  |
| HIF1AN |  | CD74 | GPS1 |  |
| DPP10 |  | PCDHB19 | NDUFS5 |  |
| C77080 |  | NKX6-3 | ERC1 |  |
| AF067061 | | SCN1B | SRRT |  |
| 4932442L08RIK | | LNP1 | ACSS1 |  |
| NLGN3 |  | RSAD2 | NDUFA5 |  |
| PCDHB5 |  | GPSM2 | DECR1 |  |
| SNX17 |  | PDE9A | NDUFB4 |  |
| ZFP36L3 |  | KLRI1 | NDUFB5 |  |
| 4930544L04RIK | | PRRXL1 | YKT6 |  |
| MFSD2A |  | GRIA4 | LRRC40 |  |
| KCTD16 |  | DSG3 | TPD52L2 | |
| PTPLAD2 | | SLC22A13 | ACSL3 |  |
| GM15800 | | OTUD4 | HINT2 |  |
| TMEM65 |  | IDH1 | EPHX1 |  |
| CCND2 |  | CDH5 | AGPAT3 |  |
| FBXO32 |  | ULK1 | ARMC1 |  |
| IIGP1 |  | PTK7 | CHMP4B |  |
| POLR2M |  | CAR11 | CMPK1 |  |
| ADAMTS17 | | HHATL | GMPR |  |
| SYT6 |  | NUDT8 | MGEA5 |  |
| RXFP1 |  | GOLGA7B | MAPK8IP3 | |
| FBXW7 |  | SLC8A2 | IDE |  |
| HOXC4 |  | JAM2 | NIT2 |  |
| 1810011H11RIK | | CHRNA7 | RILPL1 |  |
| RNASE2A | | CHID1 | TUBA8 |  |
| PIP5K1A |  | CAPSL | GLTP |  |
| EDN3 |  | KCNG3 | NT5C |  |
| F9 |  | PPP1R14A | TOMM40 | |
| APPBP2 |  | FBXW5 | QKI |  |
| TIMP3 |  | GARNL3 | EIF3I |  |
| ZWINT |  | IQCM | ACOX1 |  |
| LRRTM2 |  | CLSTN1 | DGKE |  |
| ATG4B |  | ATP6AP1L | PRODH |  |
| EBF2 |  | UNC45A | MTMR1 |  |
| APOL11A | | CYP4B1 | HNRNPF |  |
| TMPRSS11E | | DDR2 | RAB6A |  |
| NFATC2 |  | FAM234B | AP1M1 |  |
| CCR3 |  | TALDO1 | RPLP1 |  |
| ENTPD7 |  | MRPL23 | ICA1 |  |
| CD86 |  | MGEA5 | RPS5 |  |
| CACNA2D1 | | CASR | CBR4 |  |
| CREBZF |  | HELT | CUL1 |  |
| GIF |  | CNNM4 | USP15 |  |
| TNN |  | COL6A4 | SH3BGRL2 | |
| KCNH1 |  | SOX11 | YTHDF3 |  |
| PYDC4 |  | KCNT1 | TMED10 |  |
| GM21292 | | ORAI2 | GLRX |  |
| RPRD2 |  | PLA2G16 | CORO1B |  |
| FMN1 |  | AGTR1A | RPS15A |  |
| HIST1H2BK | | SBK3 | RIMS2 |  |
| LYSMD3 |  | SIX2 | SYNGR3 |  |
| SHROOM3 | | PLS3 | GOLGA4 |  |
| ZFP617 |  | NAT14 | METTL26 | |
| BC030499 | | CDH13 | TAPT1 |  |
| TMEM59L | | HK2 | MAP1LC3A | |
| GNAL |  | ZFP827 | RPS21 |  |
| LRRC39 |  | KDM3A | ARR3 |  |
| HIPK2 |  | BAIAP2 | MAP7D2 |  |
| 2410127L17RIK | | SWI5 | FMNL2 |  |
| SNAP25 |  | CYP27B1 | CTTNBP2 | |
| IVNS1ABP | | GBGT1 | EMD |  |
| OXGR1 |  | YLPM1 | PRKCSH |  |
| ZFP825 |  | NOX4 | GFRA2 |  |
| RILPL1 |  | A930018P22RIK | AIP |  |
| TBX5 |  | RRAGB | SDCBP |  |
| MRPL50 |  | ADCY6 | NDUFB11 | |
| A3GALT2 | | SLC35C1 | PURB |  |
| TNRC6B |  | SLC25A47 | AKAP7 |  |
| MBP |  | ANKRD6 | GNPDA1 |  |
| B230217C12RIK | | IL13RA1 | MTND5 |  |
| BCAP29 |  | MDN1 | WIPF3 |  |
| CAST |  | INPP5B | NID1 |  |
| NUS1 |  | MAP2 | TXN |  |
| GRM5 |  | LANCL3 | TGM2 |  |
| PTMS |  | PIH1D1 | RDX |  |
| AOX3 |  | MLANA | PVALB |  |
| KCNK2 |  | MAGED1 | RPL18 |  |
| MBOAT1 | | METTL6 | VPS26A |  |
| ATF7 |  | TPSG1 | SYT2 |  |
| IPO9 |  | PANK2 | MTX1 |  |
| CDKL1 |  | TMEM218 | GSS |  |
| A630001G21RIK | | MAT2B | USP10 |  |
| WBSCR27 | | GM32687 | ADPRH |  |
| SOX4 |  | NLGN3 | RAB4A |  |
| A4GNT |  | TUBB2A | IDI1 |  |
| SLC22A12 | | CADM4 | RRAS2 |  |
| UNC13A |  | GJB2 | TIMM13 |  |
| CNOT7 |  | SMIM10L1 | PPP1CA |  |
| ATP2C1 |  | PGR | RPS23 |  |
| T |  | DNAJA2 | SNRPD3 |  |
| SLC35F1 |  | ABCD2 | RPL32 |  |
| GM21786 | | PKD1L2 | PSMB7 |  |
| HTR5A |  | FNBP1L | PLXNA2 |  |
| DLG5 |  | USP51 | STIM1 |  |
| HARBI1 |  | TRAF4 | UROD |  |
| 5830473C10RIK | | WASHC5 | SLC30A3 |  |
| E130208F15RIK | | GM35339 | RSU1 |  |
| BANK1 |  | WDR41 | PEPD |  |
| H2-T24 |  | SERPINA3G | AIFM3 |  |
| CAR12 |  | SLC9A4 | EIF3J1 |  |
| PCDHB7 |  | SELENBP2 | CCDC177 | |
| TNFRSF13C | | DNAH2 | MTCL1 |  |
| LRAT |  | GM14406 | TMEM65 |  |
| CLDN12 |  | PAQR9 | C2CD4CC2CD4 | |
| CHMP7 |  | NXPH3 | CCDC88A | |
| ABCE1 |  | COL4A2 | LMTK3 |  |
| RPTOR |  | CEND1 | CLPB |  |
| DCBLD2 |  | ONECUT2 | KHDRBS1 | |
| CCDC85A | | DEFB1 | GCDH |  |
| TCF7 |  | TMEM206 | STXBP3 |  |
| KCNJ14 |  | VEGFA | RASA3 |  |
| CDH1 |  | NSD3 | MAPRE1 |  |
| LEMD3 |  | ANP32E | TSN |  |
| CAR2 |  | MLLT6 | SNRNP70 | |
| HAPLN1 |  | SUSD6 | SORD |  |
| ZDHHC20 | | HAX1 | CRTC1 |  |
| SLC9A7 |  | PRKCH | ELFN2 |  |
| DNAJB9 |  | SLC1A3 | ARHGAP23 | |
| PXDC1 |  | ANKRD39 | CDC5L |  |
| FMR1 |  | BMP5 | ARHGAP21 | |
| 3110035E14RIK | | NCAM2 | CPSF6 |  |
| KCNJ6 |  | REEP6 | TTYH3 |  |
| GUCY1A2 | | FHIT | DYNC1LI2 | |
| HIVEP3 |  | SERPINA9 | WIPF2 |  |
| UNCX |  | MMP17 | EXOC8 |  |
| FRAS1 |  | NKAIN4 | KCTD12 |  |
| DMXL1 |  | SCPEP1 | GIGYF2 |  |
| ZFP458 |  | PRRT2 | USMG5 |  |
| MAK |  | CRYBG3 | CYFIP1 |  |
| SMARCC1 | | PTPN9 | CPEB3 |  |
| SHE |  | SSPN | FNBP1 |  |
| NAB1 |  | TC2N | SNPH |  |
| HIST1H2AH | | STARD4 | LRRC8A |  |
| MXD4 |  | BCL9 | VAC14 |  |
| ZSCAN18 | | MFSD7A | PRICKLE2 | |
| DPY19L2 | | SUSD1 | RAP2A |  |
| CRHBP |  | KCTD6 | THEM6 |  |
| BRS3 |  | COBLL1 | SVOP |  |
| RUNDC1 |  | ZFP449 | PEF1 |  |
| GM5595 |  | FZD1 | ENOPH1 |  |
| KLF6 |  | ALOX12B | ATCAY |  |
| WISP3 |  | SCRT1 | PSMD5 |  |
| 4933406J08RIK | | CDH19 | CPNE4 |  |
| SMARCC2 | | GCK | QARS |  |
| UBR3 |  | DRAXIN | RALGAPB | |
| PIP4K2B |  | EPHX4 | HSDL1 |  |
| CYP26B1 | | CREB3L1 | CAMSAP2 | |
| GM7932 |  | BRI3 | GCC2 |  |
| B3GAT2 |  | CMTR2 | BAG5 |  |
| ACSF2 |  | LUZP2 | AGO2 |  |
| KCNQ3 |  | H13 | CDK17 |  |
| GM6768 |  | MEIS2 | FN3KRP |  |
| LIMD1 |  | PIGS | HECW1 |  |
| MSANTD4 | | ISLR2 | NUDCD3 |  |
| MROH5 |  | HAUS7 | PLCD1 |  |
| GM16485 | | APOC1 | MCAT |  |
| SCN3A |  | SGK2 | VPS39 |  |
| MEIS2 |  | PNCK | COPS8 |  |
| MCOLN3 | | GTF2F2 | SGTB |  |
| ZFP607 |  | SSH1 | NIT1 |  |
| HAS3 |  | RIMS2 | MAN2C1 |  |
| SNX20 |  | KCNC3 | DGKG |  |
| PDZD11 |  | LMX1A | DIRAS1 |  |
| D17WSU104E | | GPAT3 | RHEB |  |
| TRABD2B | | NPY1R | CRYL1 |  |
| RAB22A |  | BARD1 | TMEM121B | |
| BMPR1A |  | SIK2 | RAB27B |  |
| SCIMP |  | METTL8 | NANP |  |
| 4930539E08RIK | | FOXK1 | MTAP |  |
| GM11541 | | SLC6A7 | BZW1 |  |
| MYH15 |  | SPON1 | PSMD9 |  |
| CEP120 |  | PRX | ILF2 |  |
| LARP1B |  | EXOSC9 | TOMM34 | |
| PAPPA |  | HIST3H2BA | COPS7A |  |
| BBS4 |  | RPRML | BCS1L |  |
| RAB11FIP2 | | KLK10 | MPC2 |  |
| JADE1 |  | RSPO3 | CHORDC1 | |
| PPM1H |  | COPE | NOL3 |  |
| NTN3 |  | JADE2 | VPS33A |  |
| PSMA3 |  | DPH7 | U2AF1 |  |
| NEK9 |  | ZFP58 | SCYL1 |  |
| HEATR5B | | CTNS | DAZAP1 |  |
| SERF1 |  | MYO1E | SH3GLB1 | |
| RREB1 |  | ZFP995 | STX6 |  |
| CHL1 |  | ATAD5 | GDE1 |  |
| SERTM1 |  | LTB | ACSL4 |  |
| 2810006K23RIK | | ZFP982 | DNAJC7 |  |
| BMI1 |  | MAGEL2 | MYO1C |  |
| UBE2O |  | GM884 | TRPV2 |  |
| RC3H1 |  | GRIK2 | ASAH1 |  |
| ELFN1 |  | HMGA1B | TIMM9 |  |
| SFTPD |  | PNKD | EIF2S3X |  |
| BPTF |  | ADCY2 | PDPK1 |  |
| FSBP |  | SHQ1 | OSBPL10 | |
| EU599041 | | ZMYND8 | SMS |  |
| 4933426M11RIK | | PTPN4 | TOP2B |  |
| FAM228A | | ADPRM | MTSS1L |  |
| SPAG9 |  | KCNN4 | NUP93 |  |
| TMEM136 | | MUC6 | BRK1 |  |
| RSPRY1 |  | LRGUK | LRRC4B |  |
| PASK |  | GPR45 | THRAP3 |  |
| NTRK3 |  | MINDY2 | OCIAD1 |  |
| NDNF |  | TSPYL1 | SET |  |
| FERMT2 |  | MAST2 | RPS24 |  |
| ZFP763 |  | GM3629 | DCTN5 |  |
| FADD |  | BATF | AKT1 |  |
| UBASH3B | | NFIA | AGPAT4 |  |
| MILL1 |  | TNFRSF19 | RPL29 |  |
| DRG2 |  | PACSIN1 | NUTF2 |  |
| CNKSR3 |  | 4930548H24RIK | GRIK2 |  |
| IFT22 |  | SGTA | RPL37A |  |
| ADAMTS6 | | I830077J02RIK | NOLC1 |  |
| PPAPDC2 | | IFNAR2 | CLUH |  |
| GALNT13 | | TSHZ3 | HDAC6 |  |
| 5730455P16RIK | | TMEM184B | FKBP1A |  |
| ADAMTS5 | | TJP1 | MICU1 |  |
| DIP2A |  | IL17RD | HARS2 |  |
| DHX8 |  | LPAR5 | SPHKAP |  |
| YARS2 |  | TACC1 | FGA |  |
| GIT2 |  | PLCH1 | UBE4A |  |
| CTNNBIP1 | | GM17018 | YJEFN3 |  |
| CALM1 |  | NSMF | GTPBP1 |  |
| PGM2L1 |  | OTOP2 | GRCC10 |  |
| ARL5B |  | ILF3 | PTPN9 |  |
| TIPARP |  | KCNQ2 | KPNA6 |  |
| FAM177A | | STAC2 | IMPACT |  |
| ORAI2 |  | ACE | ILK |  |
| 9130023H24RIK | | TFB1M | HCN1 |  |
| USO1 |  | LRRC57 | C3 |  |
| BHLHE40 | | AGMO | ARAF |  |
| FAM20A |  | ZMIZ1 | GPX1 |  |
| GM12353 | | GTF2E1 | LGALS1 |  |
| CCRL2 |  | SAMD9L | CAT |  |
| APOBEC3 | | TCF7L2 | SNRPB |  |
| GTF2H5 |  | CASK | VCAM1 |  |
| ZFP160 |  | OGDH | MYO1B |  |
| DAAM1 |  | TWNK | CACNB3 |  |
| PRR11 |  | RPS6KA5 | ABCD3 |  |
| RGS17 |  | NEU3 | RAB8A |  |
| PTP4A1 |  | HYPM | PDCD5 |  |
| TCF7L1 |  | PRKAB1 | TUBGCP3 | |
| APOL11B | | SPATC1 | NPLOC4 |  |
| NRP1 |  | 6030458C11RIK | SBDS |  |
| GM9956 |  | MED12L | CACNA1A | |
| CD28 |  | PIWIL4 | RGS14 |  |
| LIG3 |  | RTN2 | MAP6D1 |  |
| DSE |  | RBMS1 | FAF2 |  |
| CDPF1 |  | OSCP1 | UAP1L1 |  |
| DENND4B | | ACTC1 | SPG7 |  |
| CTLA4 |  | SCD4 | MCU |  |
| LSMEM1 |  | OLFM2 | STRADA |  |
| NEDD4 |  | GPC4 | ANKRD28 | |
| IL9R |  | TAF13 | SPIRE1 |  |
| CS |  | AARD | DDX46 |  |
| FNDC7 |  | NBEA | MAP4K2 |  |
| SLC35F3 |  | SMCO4 | FNTA |  |
| TACC1 |  | MT-ND6 | ATRX |  |
| KDM3B |  | CCDC73 | SEMA4A |  |
| INHBB |  | SLC14A2 | PTP4A1 |  |
| TRPM6 |  | WDR59 | RALY |  |
| BARHL2 |  | MOXD1 | CA4 |  |
| GBP7 |  | PAFAH1B1 | SRM |  |
| PRRG4 |  | PLCE1 | GBA2 |  |
| HEY2 |  | MEGF6 | PREX1 |  |
| MYNN |  | GLT8D2 | NOP58 |  |
| NABP1 |  | PCDHB16 | BRINP2 |  |
| ACER1 |  | GM4922 | EXOC3 |  |
| CCR6 |  | ZDHHC23 | OCRL |  |
| DAPK3 |  | PDCD1 | DNAJC8 |  |
| TRIM2 |  | NR2F6 | MFF |  |
| SPOCK1 |  | SHANK2 | TAOK2 |  |
| CDIP1 |  | PRIM1 | ASAP2 |  |
| VMAC |  | UBE2D3 | SMAP2 |  |
| CACNG3 |  | KCNK4 | EML2 |  |
| TICAM2 |  | SLC6A11 | MON2 |  |
| TKTL2 |  | FAM205C | PGRMC2 |  |
| GRPEL2 |  | VIP | FBXO2 |  |
| MLEC |  | E330009J07RIK | DDHD1 |  |
| RORB |  | GRIPAP1 | KATNB1 |  |
| ZFP229 |  | RHBDF2 | SEPHS1 |  |
| USP51 |  | GM21093 | RGS7BP |  |
| CD160 |  | LAG3 | NDRG4 |  |
| CDS1 |  | IL12RB2 | ETF1 |  |
| TLK1 |  | IRF7 | NAA25 |  |
| ZFP324 |  | MREG | AGAP1 |  |
| KCNJ4 |  | CGN | TBCD |  |
| GM765 |  | MKL1 | TMX4 |  |
| MALT1 |  | LMO3 | ADGRL2 |  |
| MC4R |  | GBP10 | COQ9 |  |
| RP23-180L12.5 | | S100A1 | ARFIP2 |  |
| ZBTB7C |  | FBXO18 | NMRAL1 |  |
| IL7R |  | GLRX5 | RAB15 |  |
| KLRI2 |  | ZCCHC2 | MTA1 |  |
| 9830147E19RIK | | P2RX5 | CCSAP |  |
| QTRTD1 |  | GLB1L3 | STK32C |  |
| ZFP654 |  | BAAT | ASPA |  |
| ZFP92 |  | TAF7L | LUZP1 |  |
| IRAK1 |  | PCDHA3 | WASF3 |  |
| COA5 |  | POLN | CIAPIN1 |  |
| GM12185 | | MSL2 | ITM2C |  |
| AQP4 |  | RPH3A | GLDC |  |
| B3GALNT2 | | OPN4 | CRELD1 |  |
| GABRP |  | AMMECR1L | SLC12A6 |  |
| LEPREL1 | | 1700001L19RIK | ABCF2 |  |
| RBM12 |  | JPH2 | UBXN6 |  |
| RASSF9 |  | PTH1R | CYB5B |  |
| PRRC1 |  | PRR36 | SMC3 |  |
| ACMSD |  | TBC1D9B | IST1 |  |
| MGAM |  | PRSS54 | CACYBP |  |
| ATL2 |  | CRAT | RPL11 |  |
| SYT14 |  | ENTPD6 | TSFM |  |
| TBCCD1 |  | PGP | GET4 |  |
| POU4F2 |  | EPHA1 | TTYH1 |  |
| ANKRD44 | | CEMIP | CLVS1 |  |
| BNC1 |  | FAM110A | ATAD1 |  |
| VGF |  | DACH1 | FAM213B | |
| MMP28 |  | TBKBP1 | CSNK1D |  |
| GCOM1 |  | FAM81A | PMPCA |  |
| GRIK3 |  | NPFFR1 | STRN3 |  |
| PPP1R1A | | DLGAP5 | GUCY1A1 | |
| GDI2 |  | PDE8A | GTF2I |  |
| KCTD12B | | CD163 | FIBP |  |
| C8A |  | KLC1 | NECTIN1 | |
| NHSL2 |  | RPS6KC1 | SART3 |  |
| U2AF1 |  | ZBTB38 | RABGEF1 | |
| DRAXIN |  | RAI1 | SPAST |  |
| ZFP459 |  | NMNAT2 | ATP6AP1 | |
| TDRD1 |  | GM16503 | STK39 |  |
| TNFRSF13B | | RBM28 | CDK14 |  |
| NUP153 |  | SLFN3 | CAPNS1 |  |
| SEC61G |  | PRSS41 | CNBP |  |
| ZFP869 |  | PLA2G7 | CDH10 |  |
| 4930578C19RIK | | RNF13 | WNK1 |  |
| MEX3C |  | SPECC1 | NELL2 |  |
| SLC4A1AP | | TNFRSF26 | RALGAPA1 | |
| DOCK4 |  | TPSB2 | RPS27 |  |
| DHH |  | SPOCD1 | IGSF21 |  |
| XKR7 |  | MGAT4A | FSD1 |  |
| TRIM8 |  | PLPPR4 | ELAC2 |  |
| RAB23 |  | SIN3B | ZFYVE1 |  |
| SRPR |  | NPY | PNPLA8 |  |
| SOX12 |  | D130043K22RIK | BLES03 |  |
| TELO2 |  | AIRE | CHID1 |  |
| CHRNA6 |  | COL1A1 | DHRS7B |  |
| NNT |  | TERB1 | VTA1 |  |
| ABHD5 |  | PIGZ | RPS6KA2 | |
| ACTC1 |  | LYZ2 | RAB11FIP2 | |
| EBP |  | OLFR101 | GMDS |  |
| MTUS2 |  | JPH4 | SMAP1 |  |
| TMEM167 | | GCN1L1 | DHRS4 |  |
| ENC1 |  | PCP4 | SMC1A |  |
| PDE5A |  | TSR3 | ARIH1 |  |
| LPAR2 |  | PAIP1 | CSK |  |
| TTC3 |  | CACNA2D2 | TRAPPC10 | |
| EFCAB11 | | KCNT2 | SEZ6L2 |  |
| RASA3 |  | 1700011M02RIK | APC |  |
| EVI5 |  | GOLT1A | PDK1 |  |
| GNAQ |  | ATXN10 | FAHD1 |  |
| GNG12 |  | GRID2IP | GPR37L1 | |
| PRKAA2 |  | CAPN10 | AK2 |  |
| TTC23 |  | ST8SIA2 | ARSA |  |
| TMPRSS13 | | ADGRB1 | TSC2 |  |
| UBFD1 |  | TNFSF13B | SLC25A20 | |
| MFAP1B |  | PARP14 | CELF4 |  |
| MIS12 |  | TRIM41 | DIAPH2 |  |
| 5830462I19RIK | | SLC39A6 | FKBP5 |  |
| MAPT |  | NAPRT | EPHB3 |  |
| RHOQ |  | GM7276 | MAP3K5 |  |
| STXBP6 |  | 2510002D24RIK | RANBP3 |  |
| PCDHB6 |  | LMOD1 | PPP6R3 |  |
| 9330159F19RIK | | SOX13 | ISCA1 |  |
| FOXP2 |  | SELENOK | ARMT1 |  |
| BNC2 |  | COX7A1 | ATL2 |  |
| SETD1B |  | DNAH14 | TOM1 |  |
| POGK |  | MPP4 | S100B |  |
| RASEF |  | MPO | AQP4 |  |
| LAIR1 |  | PCNX | WIPI2 |  |
| LIN7A |  | COA3 | MTMR2 |  |
| FAM122B | | SYT12 | CSTB |  |
| CELF5 |  | 5530401A14RIK | FGF12 |  |
| PRKCA |  | BAHD1 | TMEM11 |  |
| L2HGDH |  | LAMB2 | UQCR10 |  |
| PIRA2 |  | FBLIM1 | U2SURP |  |
| CDH11 |  | CDH9 | NTRK3 |  |
| LDLR |  | TCTE2 | RBX1 |  |
| TAP1 |  | 1700123L14RIK | PTN |  |
| SP4 |  | CD226 | CPT1A |  |
| SCO1 |  | CCND2 | PCP4L1 |  |
| TUBGCP6 | | CISH | LIMD2 |  |
| TTC33 |  | SESN1 | MIA3 |  |
| 3110021N24RIK | | SERPINB2 | SLC2A1 |  |
| GULP1 |  | CNNM2 | SYT5 |  |
| FAM3C |  | CYP2J8 | DNAH17 |  |
| SHMT1 |  | DHX58 | FAM92B |  |
| KCNRG |  | RAB3C | LZTS3 |  |
| FRMD4B |  | ARPC5 | PPIP5K1 |  |
| ZFP84 |  | TRDMT1 | ARFGEF1 | |
| BRCA1 |  | NKIRAS2 | SLC32A1 |  |
| GM6377 |  | 2900026A02RIK | ANXA3 |  |
| PVR |  | CLK3 | CAVIN1 |  |
| NWD1 |  | B020004J07RIK | TRAPPC3 | |
| HINFP |  | FGF7 | GRK6 |  |
| VPS13B |  | SLC23A3 | SNX12 |  |
| 3110043O21RIK | | KCTD12B | AKAP10 |  |
| CIAO1 |  | RPL13A-PS1 | LIN7C |  |
| LRIG1 |  | FGF17 | SERPINA3K | |
| MRAS |  | PLXNB2 | APRT |  |
| VIPR1 |  | LARP1 | ITGB1 |  |
| VCAN |  | HOOK3 | CHGB |  |
| LRP4 |  | EGFEM1 | SELENBP1 | |
| ZFP953 |  | ROM1 | PCSK2 |  |
| CAMTA1 |  | PTPN21 | CELF1 |  |
| HERC2 |  | MST1R | RANBP1 |  |
| SDC3 |  | ROR1 | RAB23 |  |
| BAIAP2L1 | | TGTP1 | RAB24 |  |
| GIP |  | TTC34 | DNM2 |  |
| GATAD2B | | SLC30A6 | STAT1 |  |
| SPRYD4 |  | PHLDB2 | EPHB2 |  |
| SLFN3 |  | ARHGEF9 | ADSL |  |
| EXOC8 |  | CXCL12 | NUDT2 |  |
| AQP11 |  | HIST1H2AH | CYB5A |  |
| IPCEF1 |  | ANAPC2 | TM9SF2 |  |
| CRB1 |  | ACO2 | BCL2L13 |  |
| CACNG8 |  | RAP1GAP2 | MDGA2 |  |
| TRP53INP2 | | BTG1 | RANBP9 |  |
| NXN |  | MRPS18C | MAP2K6 |  |
| DUSP15 |  | COL4A5 | TIAL1 |  |
| RPS6KB1 | | BLNK | GCLC |  |
| LENG8 |  | HTR5B | F8A1 |  |
| SMAD9 |  | DHRS3 | PDE4D |  |
| ABCA5 |  | RCE1 | JUP |  |
| GIMAP5 |  | AI429214 | ACADS |  |
| PIGN |  | SLITRK6 | PLBD2 |  |
| MS4A1 |  | RHEB | AARSD1 |  |
| SLU7 |  | FAM160A1 | ESYT1 |  |
| LYNX1 |  | CNKSR2 | TARS2 |  |
| BCL11B |  | FOXO6 | AFG1L |  |
| DNASE1L2 | | TNIP2 | CDV3 |  |
| USP44 |  | AGBL4 | JCAD |  |
| UBQLN2 |  | HSPA5 | IPCEF1 |  |
| POU6F1 |  | CDK17 | MERTK |  |
| COL28A1 | | HYKK | ELAVL3 |  |
| SSTR3 |  | RIC3 | PRPF4B |  |
| KCTD2 |  | LRPAP1 | PPP2R5E | |
| HIST1H3H | | C7 | SLC9A1 |  |
| CLN5 |  | 1810037I17RIK | IGBP1 |  |
| KNOP1 |  | INTS11 | PICK1 |  |
| FLI1 |  | SRARP | MAP2K2 |  |
| NAA30 |  | IRF4 | SF1 |  |
| CCDC47 |  | SYNGR1 | GRM7 |  |
| A530099J19RIK | | NF1 | PIGS |  |
| WNT2B |  | FBXL2 | SARM1 |  |
| COL19A1 | | HMG20A | NUP98 |  |
| XIAP |  | RMI2 | ARHGAP26 | |
| FTSJ2 |  | PROSER2 | EIF2S1 |  |
| DIMT1 |  | ADAM8 | SESTD1 |  |
| SOX17 |  | LRRFIP1 | TDRKH |  |
| CCDC66 |  | GFRA1 | FBLL1 |  |
| IL5RA |  | CDK5RAP1 | PANK4 |  |
| MYCBP |  | LIAS | TNC |  |
| D630002J18RIK | | SERPINB8 | NRDC |  |
| SELL |  | EIF1AX | EIF2A |  |
| LPAR4 |  | BCKDHA | SGSM1 |  |
| DONSON | | INA | CPSF7 |  |
| TMOD3 |  | SUSD2 | NUDT9 |  |
| STRN |  | CCDC88B | GPRIN3 |  |
| FMO2 |  | TLE1 | CPNE1 |  |
| REV3L |  | KIF1C | CCZ1 |  |
| SLC2A12 |  | DHX57 |  |  |
| GM2381 |  | HIST1H1E | H2AFY2 |  |
| CORO7 |  | CYTL1 | SNX30 |  |
| RAB6B |  | GM9945 | PARS2 |  |
| ZFP930 |  | ATP8A1 | AKR7A2 |  |
| PDP2 |  | GM10912 | CALCOCO1 | |
| SLC37A2 |  | KRT1 | DIS3L2 |  |
| GBP8 |  | CYP7B1 | PAK2 |  |
| DKK3 |  | TRMT61A | RHOT2 |  |
| MCTP2 |  | GPLD1 | RPTOR |  |
| CEP76 |  | FARP2 | LGI2 |  |
| FCAMR |  | PER1 | APEH |  |
| ABCG2 |  | COP1 | HID1 |  |
| PDE6H |  | RGMB | MIC13 |  |
| NOXRED1 | | CXADR | ARHGEF12 | |
| IQCG |  | UBE2J1 | ARL6IP5 |  |
| A830073O21RIK | | PCCA | TKFC |  |
| RASGRP1 | | UBAP1 | ABHD14B | |
| SLCO4C1 | | FMO1 | IRGQ |  |
| MED14 |  | DLG1 | RAB4B |  |
| CHST4 |  | CYFIP1 | SYT17 |  |
| CLOCK |  | COX7C | SYT12 |  |
| 2900026A02RIK | | CWF19L2 | ALS2 |  |
| FAM120A | | OLFR455 | PYCR2 |  |
| KCNAB1 |  | GRIN2A | BLVRB |  |
| TMEM241 | | THOC3 | PACSIN3 |  |
| KCNA4 |  | MR1 | RRAGC |  |
| PDE1C |  | PHF21B | ME2 |  |
| MEGF9 |  | RNF223 | DNAJA3 |  |
| KSR2 |  | USP38 | ACTL6B |  |
| ZBTB39 |  | OCA2 | ATG3 |  |
| CMIP |  | TRIM37 | TXNDC17 | |
| CMAH |  | ARHGEF18 | SLC25A46 | |
| DHRS9 |  | ATXN1 | ATP6V1G1 | |
| ILDR2 |  | NOCT | FAM136A | |
| ADAM12 |  | PRSS45 | PSMD8 |  |
| PIK3CG |  | OLFR46 | BLVRA |  |
| GPR137B | | FAHD1 | RPL15 |  |
| N4BP2 |  | COL16A1 | ACBD6 |  |
| ZFP936 |  | ZFP365 | PRPSAP1 | |
| PARP6 |  | BBS10 | AKT1S1 |  |
| ONECUT1 | | DSG2 | ERP44 |  |
| TASP1 |  | GOT1 | MMAB |  |
| GALNS |  | ENHO | GBE1 |  |
| TRIM56 |  | RBM33 | TIMM50 |  |
| EARS2 |  | DDX6 | MDP1 |  |
| NYX |  | SEMA3A | ECHDC1 |  |
| AFF1 |  | CWC27 | GSTK1 |  |
| 1110032A03RIK | | FOXP3 | SORCS2 |  |
| GDPGP1 |  | SEMA3C | DAP3 |  |
| BMP3 |  | GTDC1 | WDR61 |  |
| ZFP248 |  | SNAP23 | RDH14 |  |
| HYKK |  | TMEM179 | TRAPPC4 | |
| TDRKH |  | ADRA1B | LZTFL1 |  |
| CDKN2B |  | B4GALT1 | CCDC22 |  |
| 6430548M08RIK | | KCTD8 | FMN2 |  |
| NXPH1 |  | AFDN | CDC42EP4 | |
| RNH1 |  | CACNA1A | HOMER2 | |
| RS1 |  | HIST2H3C2 | COPG2 |  |
| TNC |  | CENPB | DNAJB1 |  |
| PIWIL1 |  | SLC5A12 | PLXNC1 |  |
| AVL9 |  | TUSC3 | MTA2 |  |
| TAB2 |  | ANKRD13C | GIPC1 |  |
| HSPA4L |  | VAMP1 | TJP2 |  |
| FYTTD1 |  | LRRK1 | MTMR9 |  |
| SYNRG |  | ARID5A | KIAA1109 | |
| NPRL3 |  | C2CD5 | RUNDC3A | |
| ITGB3BP | | RNF166 | UBL4A |  |
| PCDHB14 | | MAP3K4 | TIMM10 |  |
| MXRA7 |  | METTL16 | SERPINE2 | |
| PEG3 |  | MMP11 | LIMCH1 |  |
| PANK1 |  | SLC5A3 | PPM1G |  |
| INIP |  | NALCN | GPSM1 |  |
| SLC43A2 |  | WDFY3 | RRAGB |  |
| TARDBP |  | CCDC153 | KIF1BP |  |
| SLC38A6 |  | SLC2A12 | GMPPA |  |
| TTC34 |  | ARFGAP3 | APOO |  |
| NCAM2 |  | RNF187 | CDH13 |  |
| IL1RAPL1 | | SOX5 | SEL1L |  |
| ZFHX4 |  | NOSTRIN | TRAPPC11 | |
| GJC3 |  | SELENOI | ALAD |  |
| GRIA3 |  | EIF2AK3 | EPS15 |  |
| JUN |  | CD55 | RPS20 |  |
| CYYR1 |  | AKTIP | TSC22D1 | |
| MED1 |  | FAAH | ELOB |  |
| H3F3B |  | FAM19A5 | EWSR1 |  |
| HTR5B |  | PDE2A | SCN9A |  |
| FNDC3A |  | GRAMD2 | EHBP1 |  |
| ENY2 |  | TRIM30A | PUM1 |  |
| TMEM178B | | DDX3X | DHX36 |  |
| TWISTNB | | PPP3CA | LPGAT1 |  |
| KLF16 |  | PSMA8 | ITFG1 |  |
| ERBB4 |  | EIF3M | MRPS36 |  |
| FZD2 |  | CACNG3 | GID8 |  |
| 5730596B20RIK | | EPS8L1 | HEBP1 |  |
| ART2B |  | PLP1 | KCND2 |  |
| KBTBD13 | | TAC1 | FUK |  |
| FAM172A | | PCDHB4 | PLEKHA6 | |
| GPR88 |  | GADL1 | DAAM2 |  |
| PARD6G |  | ERCC2 |  |  |
| KLF14 |  | ANKRD52 | DDI2 |  |
| RANBP10 | | TMEM40 | TMEM163 | |
| ORC4 |  | GM4881 | PDLIM5 |  |
| WNT6 |  | INTS10 | GPT |  |
| PTGS1 |  | TRAF2 | TXNRD2 |  |
| 43711 |  | APEX2 | SLC4A1 |  |
| UBE2V2 |  | AW011738 | REXO2 |  |
| IGFBP3 |  | FHL1 | FAM131B | |
| PAN3 |  | LGR5 | SLC44A2 |  |
| POLR1B |  | ZBTB7A | RAE1 |  |
| SEC11A |  | DNAJC21 | FHL2 |  |
| PLEK |  | GLUL | VPS16 |  |
| FAM104A | | PGRMC1 | MAGED1 | |
| MYO9A |  | CD86 | ZZEF1 |  |
| TCEANC |  | ADCY5 | LAMA2 |  |
| EIF4E |  | ZFP692 | FBXO22 |  |
| SLFN8 |  | UBE2L3 | CAPN1 |  |
| CCSER1 |  | VMN2R86 | M6PR |  |
| ITPKB |  | ZBTB40 | CRYZL1 |  |
| GAN |  | FBXO3 | CCDC51 |  |
| SLITRK2 |  | HYDIN | PRPF6 |  |
| PGR15L |  | LRRC8D | AACS |  |
| TRIM71 |  | LYPD6B | TMX2 |  |
| ZEB1 |  | CDH8 | MCTS1 |  |
| SLC16A6 |  | FGF5 | SDR39U1 | |
| SYTL5 |  | CARS2 | MTFR1L |  |
| LZIC |  | L1CAM | USP19 |  |
| TM9SF4 |  | RALGPS2 | CLINT1 |  |
| PPP4R2 |  | NUMA1 | PMPCB |  |
| 1700048O20RIK | | CYP2C23 | ENSA |  |
| 4930524B15RIK | | IRX6 | RIMS3 |  |
| RPL24 |  | RETREG1 | PDXDC1 |  |
| MEF2A |  | RARS | AMIGO1 |  |
| AMOTL1 |  | MTHFSL | PLPP3 |  |
| OLFR1033 | | SGCD | COASY |  |
| ZNFX1 |  | IRS1 | PTGES3 |  |
| NOV |  | PRKD2 | ERI3 |  |
| ZFP442 |  | DNAIC2 | NANS |  |
| RGS7BP |  | PLXNA3 | ACIN1 |  |
| RSL1 |  | KCNJ12 | ZC3H15 |  |
| NDOR1 |  | NPHP1 | SCN2B |  |
| HRNR |  | TCEA2 | DHX29 |  |
| NYAP2 |  | SMYD3 | KIAA1671 | |
| CXCL11 |  | RGS19 | DYNLT3 |  |
| DNAH17 |  | CAR8 | CCDC47 |  |
| SHANK1 |  | SLC22A21 | NID2 |  |
| MOSPD1 |  | VAC14 | ITSN2 |  |
| NEK7 |  | PEX7 | EIF2S2 |  |
| PCYOX1 |  | MYO1B | GLB1 |  |
| RNF130 |  | CLEC4G | F3 |  |
| FER1L6 |  | A430033K04RIK | EFR3A |  |
| BSDC1 |  | ATG12 | NUDT21 |  |
| PALM2 |  | CANT1 | PTPRT |  |
| ZFP386 |  | GINM1 | STUM |  |
| D10BWG1379E | | TMEM164 | EMC7 |  |
| GDF11 |  | 9030624G23RIK | SSBP1 |  |
| RP1 |  | GM14569 | ARSB |  |
| TNFRSF11A | | FBXO30 | NDUFA11 | |
| KIFAP3 |  | MAG | PMM1 |  |
| ZFP462 |  | ALKAL2 | SRP68 |  |
| PTPN14 |  | TRUB1 | PRKAG2 |  |
| D630045J12RIK | | APOL7B | ATP13A1 | |
| UGT2B35 | | LRRIQ4 | SYT11 |  |
| AP1S3 |  | OLFR19 | ATP2A1 |  |
| TAF4A |  | TPCN2 | TBC1D13 | |
| B3GNT9 |  | POLR2A | RGS17 |  |
| GLDN |  | TUBA3B | COA7 |  |
| PLA2G3 |  | CD180 | STX8 |  |
| CYB5R2 |  | DOCK3 | NMT2 |  |
| GPRIN3 |  | NPR1 | SEMA4D |  |
| PTCHD1 |  | ACTG1 | LLGL1 |  |
| FGF12 |  | LMNB2 | MGST3 |  |
| ABCA6 |  | PCDHGB8 | ADRM1 |  |
| LAPTM4B | | UPF3B | FKBP15 |  |
| SH3BP5 |  | RBM42 | SLC6A3 |  |
| BARD1 |  | ZBTB7B | CHAMP1 |  |
| ARHGEF38 | | UBASH3A | VIPAS39 |  |
| SIX4 |  | STAT1 | ABCF1 |  |
| 1700001O22RIK | | AP1S1 | CPLX2 |  |
| FAM217B | | SMYD2 | TMED2 |  |
| FNDC8 |  | PPT1 | TIAM2 |  |
| ACOT4 |  | IFI202B | SLC17A6 |  |
| PAQR8 |  | WISP2 | IL1RAP |  |
| GBX1 |  | VMN2R87 | NT5C3A |  |
| VPS26B |  | BUD23 | HDGFL2 |  |
| LRRC15 |  | NTRK1 | TIMM22 |  |
| 9430015G10RIK | | DARS2 | KCNC2 |  |
| UNC13C |  | ATL1 | NUP160 |  |
| HOOK1 |  | LMTK2 | SIPA1L3 |  |
| PROK1 |  | HARS | PPP2CA |  |
| HOMEZ |  | PLCL2 | MP68 |  |
| STX19 |  | DMKN | ATP5F1E | |
| GNAI1 |  | 2410002F23RIK | EIF4E2 |  |
| ZFP81 |  | ZC3H3 | MTATP8 |  |
| SOX5 |  | OCEL1 | TMSB10 |  |
| VWA5A |  | RAB21 | HIGD1A |  |
| SFN |  | PI4KA | ANKRD63 | |
| KEG1 |  | MAPK11 | PDZD8 |  |
| SLC16A12 | | TTLL3 | MRAS |  |
| GM4951 |  | GABRG1 | BCAT2 |  |
| USP30 |  | APC | DNAJB6 |  |
| HOXD13 |  | TRMT12 | COMT |  |
| SRI |  | NUCKS1 | ANXA2 |  |
| ZNRF3 |  | DGKE | UMPS |  |
| LRFN1 |  | XLR3B | SLC4A3 |  |
| PROZ |  | PCBP2 | MUG1 |  |
| 1700034I23RIK | | REL | MARCKSL1 | |
| ELL |  | PCDHGB2 | GFPT1 |  |
| TMEM158 | | SLC6A3 | PITPNB |  |
| ZBTB4 |  | 8-Sep | DCAF7 |  |
| SV2C |  | DCST2 | UFD1 |  |
| LEMD1 |  | MAP3K1 | ARF5 |  |
| SNUPN |  | RASL10B | ISOC2A |  |
| FGD3 |  | OAS1B | NEO1 |  |
| PTCD3 |  | PTK2 | LYPLA1 |  |
| GM6871 |  | ZFP874A | G3BP1 |  |
| MOK |  | TMEM171 | DTYMK |  |
| GADL1 |  | ICAM4 | APLP1 |  |
| UBE2F |  | SNW1 | KCNB1 |  |
| DOCK5 |  | SLC35F5 | EIF5B |  |
| STOX1 |  | CDKL5 | LMTK2 |  |
| RALGPS2 | | NISCH |  |  |
| INO80D |  | STC2 | PTPRN |  |
| POU3F2 |  | GM16867 | FXR1 |  |
| KCNH7 |  | LXN | TPD52 |  |
| B3GALT5 | | HACD1 | REPS2 |  |
| ALX4 |  | KLF14 | ATP2C1 |  |
| AMPD3 |  | INSIG1 | METAP1 |  |
| UTRN |  | SLC9A6 | FAM177A1 | |
| TENM2 |  | ECH1 | TCP11L1 |  |
| SLC6A5 |  | ASTL | MRPL22 |  |
| POMK |  | RUNX3 | COPS7B |  |
| SH3PXD2B | | MAT1A | STX16 |  |
| BMP10 |  | CTSZ | PITHD1 |  |
| LPP |  | MAP3K13 | NADK2 |  |
| SPOPL |  | LAT2 | AMER2 |  |
| TRIM12C | | ESAM | SLTM |  |
| NUGGC |  | IL20RA | BCLAF1 |  |
| KIF5B |  | ZFP37 | TRHDE |  |
| ARRB1 |  | GTSE1 | APPL2 |  |
| NFAM1 |  | JADE1 | REEP2 |  |
| YPEL2 |  | BMP8B | TXNDC5 |  |
| GPCPD1 |  | AVIL | CAMK1 |  |
| SYNGR4 |  | RPS27RT | CHMP1A |  |
| ATXN1 |  | COQ9 |  |  |
| RABGAP1L | | OVGP1 | THUMPD1 | |
| LHFPL2 |  | DBI | USP11 |  |
| FARP2 |  | OPALIN | TOMM22 | |
| SLC12A6 |  | REN1 | GOLPH3 |  |
| CMC2 |  | ELN | SNU13 |  |
| SAMD3 |  | SCYL2 | ATP5F1D | |
| CDC25B |  | DRAP1 | SYNJ2BP | |
| DESI2 |  | LACTB | DNAJB4 |  |
| ZC3H7B |  | NMBR | JAM3 |  |
| MID2 |  | HIST1H3C | CNN3 |  |
| PHF20 |  | CASC1 | EIF3K |  |
| ADAMTSL5 | | PABPN1L | GNAI3 |  |
| CLCA2 |  | WFDC15B | PYCR3 |  |
| PREX2 |  | 3-Mar | SLC38A3 |  |
| JADE2 |  | BBS7 | PARVA |  |
| CYP51 |  | CACNB3 | NIF3L1 |  |
| SMCR8 |  | NUP214 | SON |  |
| ECSCR |  | CSL | CNPY2 |  |
| CHST11 |  | GM6710 | SLC25A10 | |
| HFE |  | MOG | APIP |  |
| THEM7 |  | FAM117A | FXR2 |  |
| HOMER2 | | FAM118B | EIF3G |  |
| ZIC5 |  | MFAP5 | IK |  |
| GINS3 |  | STK32B | TROVE2 |  |
| VTI1A |  | TOMM20 | CACNG2 |  |
| GREB1 |  | TCAIM | CTSL |  |
| RTP2 |  | ZCCHC3 | USP46 |  |
| PER2 |  | NUDT2 | RPS14 |  |
| RAB38 |  | SHCBP1 | CYTH2 |  |
| TPH1 |  | IDE | TP53BP1 |  |
| SLC35G2 |  | SLC34A2 | APBA2 |  |
| MEGF6 |  | OLFR63 | MARK3 |  |
| APTX |  | TEX2 | CPNE7 |  |
| BC048403 | | PSG29 | SLMAP |  |
| SRPK2 |  | LPIN2 | NAA50 |  |
| VMN1R43 | | SLFN9 | ALDH3B1 | |
| HINT3 |  | POU2F1 |  |  |
| ERICH4 |  | COL3A1 | YARS2 |  |
| PON2 |  | RASGEF1A | GUF1 |  |
| SNX29 |  | DEPP1 | TSR2 |  |
| GABRG2 |  | FHDC1 | RYDEN |  |
| GM11437 | | SGPL1 | LARS2 |  |
| EGR3 |  | TPBGL | GLYR1 |  |
| CCDC141 | | GM42715 | GORASP2 | |
| SERPINB5 | | MLLT3 | NSMF |  |
| CYHR1 |  | MRPL39 | SRPRA |  |
| 4933411K20RIK | | CEP126 | CLPX |  |
| COLEC10 | | GOLIM4 | BCAM |  |
| GAD2 |  | PQLC2 | RBM25 |  |
| CLIC1 |  | KLHL25 | RIC8A |  |
| IGLON5 |  | SIS | SLC30A9 |  |
| SH2D7 |  | ERCC6L2 | MARK4 |  |
| PRPS2 |  | GM10643 | UBXN4 |  |
| PRRXL1 |  | PI15 | STRBP |  |
| AR |  | PHKB | NAA10 |  |
| ZDHHC7 |  | ELOVL2 | SART1 |  |
| NTRK2 |  | CDK5R1 | TPP1 |  |
| KATNAL2 | | FXYD7 | COPZ1 |  |
| HGF |  | POP5 | RHOG |  |
| IL22RA1 |  | PRRG3 | FAM49A |  |
| BRAF |  | POLR2J | UBQLN1 |  |
| ELMOD2 |  | PIEZO1 | MSI2 |  |
| CEBPG |  | SSH2 | AP3M1 |  |
| FANCF |  | DAAM1 | WDR54 |  |
| GCNT1 |  | EPPK1 | PGBD5 |  |
| SIGLECH | | COX4I1 | STT3B |  |
| ADORA1 |  | NAT6 | CARMIL2 | |
| TNFAIP3 |  | SLMAP | KCMF1 |  |
| TRIQK |  | PRPS1L3 | NUP107 |  |
| CYLC1 |  | UNC5D | TSPAN2 |  |
| ATRNL1 |  | PALMD | ETHE1 |  |
| MINOS1 |  | BGN | KIF5A |  |
| ST6GAL2 | | SEC22B | KHDRBS3 | |
| 1700102P08RIK | | PIK3C2G | HECTD3 |  |
| ATG14 |  | AU022252 | ZNF638 |  |
| PTK6 |  | ATMIN | ISCU |  |
| KCNV2 |  | OSBPL2 | GAA |  |
| POC1B |  | NENF | SRSF6 |  |
| KANSL2 |  | PALLD | TRNT1 |  |
| ST8SIA1 |  | CARMIL1 | FGF1 |  |
| SRGAP3 |  | SHISA7 | PCYOX1L | |
| CCDC15 |  | SLC23A4 | ATP6AP2 | |
| DCUN1D1 | | OLFR61 | TENM1 |  |
| SSBP2 |  | ZFP410 | FN3K |  |
| ZFP827 |  | FGFR1OP2 | CIRBP |  |
| AW554918 | | CYP1B1 | NACA |  |
| PROM1 |  | SPINT2 | VPS28 |  |
| CAPN8 |  | AMY1 | SERPINI1 | |
| 2310045N01RIK | | CCDC198 | EPS8 |  |
| TNFRSF10B | | ARHGEF39 | SLC16A1 |  |
| UBE2E3 |  | GRB14 | STIM2 |  |
| LAMTOR3 | | AIG1 | JPH4 |  |
| BATF2 |  | GM9999 | LNPEP |  |
| ITPRIPL1 | | 4930432M17RIK | ABCG2 |  |
| NQO2 |  | PILRB2 | SH3BGRL | |
| LHX6 |  | GM3453 | FGB |  |
| CACNB2 |  | TRIM40 | HMGCS1 |  |
| 5430435G22RIK | | FMO6 | ATP5S |  |
| CAPN2 |  | CALML4 | DNAJB11 | |
| TIAL1 |  | FIGN | CFDP1 |  |
| MAPK1IP1L | | GRIK3 | GMPPB |  |
| CCDC24 |  | TUBGCP5 | HPX |  |
| NMNAT1 | | FBN1 | DTD1 |  |
| MANEA |  | FAM81B | PLCH2 |  |
| ZFP438 |  | ACY1 | CRIP2 |  |
| COA4 |  | LKAAEAR1 | RPL38 |  |
| RRP12 |  | IGBP1B | MYH4 |  |
| GM10447 | | CHRM2 | TNIK |  |
| SLURP1 |  | CAP1 | CACNG3 |  |
| SLC11A1 |  | RNF183 | NSDHL |  |
| DEPTOR |  | SLC5A1 | KRIT1 |  |
| STOX2 |  | FOLR2 | SMCHD1 |  |
| DUSP18 |  | HAUS4 | DGKQ |  |
| THBS2 |  | BTF3L4 | NOP2 |  |
| VMN1R44 | | KLF15 | GNG4 |  |
| CRISPLD2 | | H2-Q1 | MLYCD |  |
| VWC2 |  | BC024139 | UBTF |  |
| 4930523C07RIK | | KCNH8 | KYAT3 |  |
| RFX7 |  | TNNI3 | MVD |  |
| SOWAHA | | TMEM196 | LSM12 |  |
| FIP1L1 |  | PCDHB8 | EMB |  |
| EIF3J1 |  | DOC2A | PPP1R1A | |
| FBXL16 |  | DAPL1 | GATM |  |
| CHRM5 |  | FAM169B | EFL1 |  |
| 2810021J22RIK | | AUH | GDPD1 |  |
| COL4A1 |  | GM7030 | TMEM132A | |
| SNRPN |  | ATP6V1D | RARS2 |  |
| GUCY2F |  | ATRNL1 | TBRG4 |  |
| GM15737 | | MXI1 | CCNY |  |
| PTPRO |  | RTP1 | TRAF3 |  |
| GYPC |  | AP3M2 | RPL35A |  |
| MRGPRD | | PRR12 | ARL8B |  |
| PTPLAD1 | | GM6592 | RAB22A |  |
| PTPN6 |  | NXF7 | ATL3 |  |
| ZFP935 |  | TGIF2 | SUGP2 |  |
| CEP41 |  | GALNT12 | NEDD8 |  |
| B4GALNT2 | | NAGLU | BCAP31 |  |
| 2310005G13RIK | | SLC30A9 | PEX14 |  |
| SMC3 |  | ODF2 | MAP7 |  |
| NCAM1 |  | HABP4 | RPL27A |  |
| MAS1 |  | TUBGCP4 | USP24 |  |
| NCF2 |  | ATP6AP2 | MRPL11 |  |
| RPE |  | COL4A3BP | FTO |  |
| GGT5 |  | TLL2 | SETD3 |  |
| ANXA8 |  | RRP1B | PTGR2 |  |
| LGALS4 |  | CEACAM9 | ABCB8 |  |
| LCORL |  | QPCTL | NYAP2 |  |
| STK17B |  | UQCRH | RUFY2 |  |
| GIMAP4 |  | RIMS4 | SRSF7 |  |
| CERS4 |  | FAM161A | S1PR1 |  |
| PNRC2 |  | EMC3 | MRPS5 |  |
| C2CD2 |  | KANTR | CMTR1 |  |
| SRD5A2 |  | DUSP28 | NECAB2 |  |
| ACLY |  | 0610010K14RIK | GSTO1 |  |
| ONECUT3 | | VMN2R10 | AP3S1 |  |
| GRM7 |  | OLFR1391 | LCP1 |  |
| PARK2 |  | MCOLN2 | LRP1B |  |
| IREB2 |  | GRM6 | KNG1 |  |
| SLC6A20B | | GP9 | SMU1 |  |
| KCNH5 |  | TCF7L1 | MAPK9 |  |
| THBD |  | DSTN | ARL15 |  |
| RAD54B |  | TTC9B | FKBP1B |  |
| MAST2 |  | TET3 | NME3 |  |
| NT5E |  | RFC5 | MYDGF |  |
| KLF7 |  | LHX9 | MAGI3 |  |
| TC2N |  | TATDN3 | SAR1A |  |
| PSMD4 |  | EIF2B3 | HEATR5B | |
| CCDC112 | | JMJD8 | DZANK1 |  |
| ZC3H10 |  | KRT20 | LEMD3 |  |
| EDEM1 |  | GNG7 | GSTT1 |  |
| ADHFE1 |  | TSPAN10 | TEX2 |  |
| 6330403A02RIK | | HERPUD2 | RFTN2 |  |
| CEP131 |  | CHSY3 | NIPSNAP3B | |
| SDC4 |  | HJURP | PHKB |  |
| POLR3E |  | TMEM63B | CYB5R1 |  |
| IDE |  | ORMDL2 | SEC23B |  |
| UBXN2A |  | KLF1 | LIN7B |  |
| FSTL4 |  | SPIDR | DNAJC19 | |
| RAD23B |  | RPS27A | CHD4 |  |
| DENND3 |  | NHP2 | TRP53I11 | |
| TRIM5 |  | NIP7 | HSDL2 |  |
| BC034090 | | TSGA8 |  |  |
| SLC25A44 | | 3830403N18RIK | CCDC58 |  |
| CABP2 |  | DPEP1 | MYADM |  |
| CCDC25 |  | AMPD1 | UQCC1 |  |
| MILR1 |  | SELP | ATOX1 |  |
| EYA1 |  | KRT79 | FYN |  |
| ASL |  | SPPL2B | LY6H |  |
| PSMB9 |  | SLC8A3 | ADGRB3 |  |
| MAPK9 |  | GM128 | FSIP2 |  |
| PLEKHM3 | | PYURF | NCLN |  |
| HAX1 |  | PCDHA5 | DYNLL1 |  |
| PLAGL2 |  | KDM5C | SUPT5H |  |
| GPATCH2 | | ATAD2B | GATD1 |  |
| DLG1 |  | TUSC1 | GMPR2 |  |
| DIAP2 |  | KLF11 | IMPDH2 |  |
| KBTBD11 | | LRRC26 | ZWINT |  |
| PRPF39 |  | IL33 | OXSR1 |  |
| NEU3 |  | ARL5C | DNMT1 |  |
| SLIT1 |  | PSMD12 | RCC1 |  |
| IGFBP5 |  | TMEM41B | UBR1 |  |
| GABRR2 |  | ARHGAP30 | FAM171A2 | |
| PLSCR1 |  | NDUFS1 | RPL36 |  |
| FCRL1 |  | CP | PARP1 |  |
| SYTL3 |  | SEL1L3 | CD9 |  |
| PHF20L1 |  | PRR16 | DNAJA4 |  |
| ERAP1 |  | HDAC4 | CALU |  |
| ZFP759 |  | DTL | DDAH2 |  |
| CHST3 |  | ENC1 | RBM12 |  |
| ALOX5 |  | ESYT3 | CDH11 |  |
| RERGL |  | UVRAG | PPP1R10 |  |
| GNRH1 |  | KCNA4 | DOCK7 |  |
| RAB9B |  | MYL9 | EIF2B5 |  |
| FGF14 |  | DNAAF2 | RIOX1 |  |
| SHISA9 |  | GRM8 | TIAM1 |  |
| SHC4 |  | PRRC2B | ACTBL2 |  |
| EBF1 |  | RIIAD1 | PDS5B |  |
| DICER1 |  | RB1 | MTND1 |  |
| PLEKHH3 | | PRKCE | ENPP5 |  |
| A730020M07RIK | | GABRB3 | PROM1 |  |
| CETN4 |  | ZFP59 | SMAP |  |
| SLC4A8 |  | BEND4 | LNPK |  |
| MED13L |  | PKP1 | FGFR2 |  |
| FAM212B | | BTK | TXNDC12 | |
| NPR3 |  | FAM71D | EMSY |  |
| POFUT1 |  | SARS | SDHD |  |
| ADCY8 |  | IFITM10 | PROS1 |  |
| VRK1 |  | PPFIBP1 | MAP1LC3B | |
| RALGPS1 | | WDR72 | VTI1B |  |
| EIF3C |  | TIMM8A2 | DNAJB5 |  |
| M1AP |  | A1CF | AIMP1 |  |
| SH3GLB1 | | TRIM30C | S100A1 |  |
| MSR1 |  | NFE2L1 | VAMP7 |  |
| TMEM87B | | TTC39D | SLC2A13 |  |
| P2RY2 |  | OTX2 | NDUFAF2 | |
| CHST7 |  | OLFR145 | TMPO |  |
| 1810041L15RIK | | DDX60 | RIPOR1 |  |
| HAS2 |  | ST8SIA6 | LRFN2 |  |
| LYPLAL1 | | SH2B2 | HAPLN4 |  |
| AMER3 |  | SPPL2A | MFN1 |  |
| CD93 |  | FAM227A | SLC7A14 |  |
| MFAP1A |  | PDE6G | MAP2K7 |  |
| PRR18 |  | ACVRL1 | GLCCI1 |  |
| FAM160B2 | | ULK3 | FUOM |  |
| ZFP346 |  | FOXF1 | RTN4IP1 |  |
| WIPF2 |  | ENPP3 | TBCB |  |
| FBXL18 |  | SMAD7 | MRPL12 |  |
| PIK3R5 |  | SLC3A2 | ARMC8 |  |
| ZFP213 |  | FBXO45 | EIF3F |  |
| ZNRF1 |  | NPR2 | EHD4 |  |
| PTGIR |  | GM14862 | LAMTOR2 | |
| TAF2 |  | GRCC10 | APBB1 |  |
| GRIN2B |  | SWAP70 | TSNAX |  |
| XIRP2 |  | RAD23B | STK11 |  |
| COL6A1 |  | IL2RG | KIT |  |
| LRRC7 |  | GM10490 | EIF1 |  |
| PANX2 |  | EXTL2 | ANAPC1 |  |
| HDX |  | PRCC | ENAH |  |
| XPNPEP1 | | GM6657 | ANKMY2 | |
| SLC25A42 | | PGS1 | PPP2R5B | |
| THSD7A |  | TCP11 | ACBD3 |  |
| SERPINA3I | | CLOCK | ACSL5 |  |
| ABCB9 |  | COX14 | VPS25 |  |
| SPNS2 |  | GABRA1 | OXSM |  |
| TMEM63C | | RBM12B1 | GOLGA7B | |
| LHX4 |  | COCH | RTCA |  |
| NUDT12 |  | RHOB | NFU1 |  |
| RAB39 |  | MNAT1 | PIKFYVE | |
| KIF21B |  | PIK3CD | DEK |  |
| ZFP319 |  | IL1RAPL1 | GLRX5 |  |
| HS3ST3A1 | | PACSIN2 | EIF3M |  |
| GLI3 |  | KIF26A | BCAS2 |  |
| TMPPE |  | CBLN1 | FTL1 |  |
| TBC1D16 | | AGK | TAGLN |  |
| MAPK8IP3 | | PLK2 | UNC5A |  |
| KLHL21 |  | ZRANB3 | SELENOM | |
| NUFIP2 |  | FASTK | SEC63 |  |
| TMEM63A | | ZFP871 | HNMT |  |
| LTBR |  | FGF14 | ECSIT |  |
| FAT2 |  | CNTNAP5B | SCG5 |  |
| ITGA8 |  | FAAP100 | NUDT16 |  |
| PRTG |  | ARFRP1 | DNAJB2 |  |
| IL17RD |  | PDLIM5 | GCLM |  |
| TET1 |  | RNF139 | CD82 |  |
| KMT2A |  | SLC50A1 | BCKDHA | |
| INHBA |  | GBP4 | PTCD3 |  |
| LIMS1 |  | MAX | EIF2B4 |  |
| ALKBH5 |  | KBTBD3 | UBQLN4 |  |
| ABLIM1 |  | ENG | WDFY1 |  |
| AKAP13 |  | 1110038F14RIK | TBR1 |  |
| FIGNL2 |  | APAF1 | PPTC7 |  |
| FOXO3 |  | SYNGR3 | CPM |  |
| XYLT1 |  | EFHC2 | MRPS23 |  |
| GLIS3 |  | MKRN1 | PPP1R2 |  |
| ZC3H12C | | OLFR460 | RBM3 |  |
| SLIT3 |  | CAR13 | SLC6A9 |  |
| TOR1AIP2 | | CPLX2 | IL1RAPL1 | |
| 2810474O19RIK | | TDRD6 | SLC4A7 |  |
| KCTD21 |  | BC048403 | ASPSCR1 | |
| PCMTD1 |  | RBMS3 | GGCT |  |
| TMEM175 | | TMEM182 | CNTFR |  |
| PIGW |  | GM2310 | DNAL1 |  |
| MCIDAS |  | ADCK5 | CLMN |  |
| HSPA1B |  | CD84 | MRPL13 |  |
| HIC2 |  | RPL31 | PTRH2 |  |
| ARHGAP31 | | BTBD9 | TOMM20 | |
| SOWAHC | | GNGT2 | PAPSS1 |  |
| CTNNA3 |  | NGLY1 | ELFN1 |  |
| SERPINA3C | | ENPEP | GGA1 |  |
| SMAD5 |  | PRPS1 | MACROD1 | |
| PEX7 |  | MATR3 | MRRF |  |
| UBE3B |  | TRAPPC13 | TOMM40L | |
| GABRB1 |  | KLHDC9 | HSD17B8 | |
| SIMC1 |  | SAXO1 | CNNM1 |  |
| SLC17A2 |  | METTL22 | MARS2 |  |
| H2AFV |  | ATP6V1G2 | COMMD3 | |
| LYVE1 |  | ABHD2 | ADO |  |
| TBC1D1 |  | ALDH6A1 | HINT3 |  |
| CAND1 |  | THOP1 | AKR1E2 |  |
| KRT84 |  | VTI1B | DHODH |  |
| TTF1 |  | ZDHHC12 | S100A13 |  |
| PRPF19 |  | FAM50A | DENR |  |
| SLX1B |  | CUL5 | LMBRD2 |  |
| SMAD3 |  | 9130213A22RIK | SPON1 |  |
| CELF2 |  | TFB2M | ALYREF |  |
| ZFP568 |  | ZFP128 | CFAP36 |  |
| NUP155 |  | CYP2G1 | TIMM8B |  |
| LYZ2 |  | VMN1R55 | CROT |  |
| ALG10B |  | OLFR533 | SNW1 |  |
| AFAP1 |  | VAMP2 | VLDLR |  |
| UGT2A1 |  | GPR35 | ADPRHL2 | |
| FBXL6 |  | SHB | CTPS2 |  |
| KCTD1 |  | TMX2 | CDK18 |  |
| KRR1 |  | KLHDC8B | SNRPE |  |
| POT1B |  | GTF2F1 | SLC30A1 |  |
| TOX4 |  | CMBL | OLFM2 |  |
| GPR4 |  | TPBG | DENND1A | |
| PCDH19 |  | SNRPA | SLC1A4 |  |
| LNPEP |  | TTC13 | RGS20 |  |
| TMEM88 |  | MALT1 | SARDH |  |
| UBN2 |  | GM12394 | SPOCK2 |  |
| HSPB7 |  | BMP15 | TACO1 |  |
| DARS2 |  | PLG | ARF6 |  |
| SUV39H2 | | GRPEL1 | TIMM29 |  |
| CES2B |  | JAG1 | MRPS22 |  |
| CD2AP |  | CSMD1 | EXOC6 |  |
| ANO5 |  | TSNAXIP1 | CLIC1 |  |
| SENP8 |  | CFL2 | VTI1A |  |
| ZFP516 |  | BEAN1 | VPS37B |  |
| TMEM220 | | KCNJ2 | TH |  |
| ZFP704 |  | UBASH3B | ACP6 |  |
| PKN2 |  | SCARF1 | CNST |  |
| SLC22A15 | | PRSS8 | APEX1 |  |
| COG3 |  | A4GNT | FAM3C |  |
| CCR1 |  | KIF5C | SARNP |  |
| TRIM58 |  | CENPP | GUK1 |  |
| PDE10A |  | CARD6 | SRPRB |  |
| TMEM74 |  | PPP1R26 | TCEA1 |  |
| CDH12 |  | POMP | PLXNA3 |  |
| EYA4 |  | LUC7L | PSME3 |  |
| FAM206A | | TUBA8 | MRPS30 |  |
| PRDX6B |  | SLC9A9 | OSBPL6 |  |
| MEGF11 |  | SLC39A8 | CCDC85A | |
| FRMD7 |  | FAM43B | HTATSF1 | |
| SIAE |  | GNL2 | RPL22 |  |
| OLFR619 |  | RASA4 | AGFG2 |  |
| MBOAT2 | | DCT | COMMD9 | |
| C030039L03RIK | | SOD1 | CLSTN3 |  |
| INPP1 |  | NUP210L | PLS1 |  |
| ILDR1 |  | HECW1 | LRSAM1 |  |
| SNX16 |  | BLCAP | TAX1BP1 | |
| IRF7 |  | P2RX4 | ERH |  |
| ATL3 |  | PLEKHG2 | CUL4A |  |
| HAUS2 |  | RRM2 | THSD7A |  |
| BRCC3 |  | KCNB2 | NAA35 |  |
| PIAS1 |  | LRRC8B | SCYL2 |  |
| CYP4F14 |  | 8030423J24RIK | SMAD2 |  |
| LIPO1 |  | UBA7 | STK38L |  |
| NDUFAB1 | | NAIP6 | UAP1 |  |
| CEP162 |  | NDRG4 | UBE2Z |  |
| RPS26-PS1 | | GPD1 | LYSMD2 |  |
| CELF4 |  | ALS2 | OSBP2 |  |
| NUDT21 |  | GRM5 | PAM16 |  |
| FAXC |  | 1600014C23RIK | TIMM8A1 | |
| ABI2 |  | ALDH1A7 | SLIRP |  |
| AGO3 |  | CHRM4 | CHERP |  |
| CD59B |  | RNF219 | PFDN2 |  |
| TNR |  | PHYHIP | SNRPD1 |  |
| TRPS1 |  | CDH16 | HPCAL1 |  |
| SGCB |  | OLFR1348 | LAMP1 |  |
| MIB2 |  | GM12830 | RAB9A |  |
| ACNAT2 |  | MAST3 | DCAF8 |  |
| GM10638 | | CD209B | AKAP1 |  |
| NUDT5 |  | ARID1A | OTUD6B |  |
| SEMA5A |  | DAB1 | SEC62 |  |
| RAB3C |  | EXTL3 | SCG3 |  |
| TRPV1 |  | NECTIN3 | PLCB3 |  |
| SLC45A3 |  | AC170998.1 | ARRB1 |  |
| F2RL3 |  | CMAS | UROS |  |
| D19BWG1357E | | BCDIN3D | INPP5J |  |
| PBXIP1 |  | SMIM19 | NKIRAS1 | |
| DGKQ |  | CLN6 | ANPEP |  |
| KLHL29 |  | OLFR414 | BCKDHB | |
| GPR171 |  | SPDEF | PADI2 |  |
| WRB |  | SNRPG | LMAN2 |  |
| ZFP169 |  | MED9 | TAOK1 |  |
| SLC41A2 |  | CALHM6 | DSCR3 |  |
| PECAM1 |  | 4930519P11RIK | PPM1F |  |
| AGPAT3 |  | MAEL | MRPS35 |  |
| PHLPP2 |  | 4930430F08RIK | POMGNT2 | |
| TTLL4 |  | 0610040J01RIK | TRAPPC6B | |
| MED12L |  | GCSH | RDH13 |  |
| KDM5A |  | NRTN | ATG4B |  |
| SPHKAP |  | DTX3L | NUP54 |  |
| NIN |  | SERINC1 | GSDME |  |
| DCDC2A |  | FAM185A | ADCY6 |  |
| MFAP3L |  | USO1 | PTMS |  |
| GM3629 |  | CAP2 | VEZT |  |
| CRIPT |  | BARX2 | SARS2 |  |
| IGF2BP2 |  | CNN3 | AVL9 |  |
| FERMT1 |  | RAB11FIP1 | SSR1 |  |
| FUT9 |  | PRELP | CUTA |  |
| HAUS6 |  | ASCC1 | PPP2R2C | |
| ANKS6 |  | CCDC85B | CCAR1 |  |
| BCLAF1 |  | CXCL16 | PAIP1 |  |
| NDUFAF7 | | KIF20A | IQCB1 |  |
| SCG3 |  | RNF146 | ARL1 |  |
| HRCT1 |  | MX2 | MOCS3 |  |
| HPGDS |  | GPHB5 | ANLN |  |
| IKBKG |  | BC107364 | CDH6 |  |
| TMEM126A | | EFNA1 | SDHC |  |
| GALNT4 |  | MAP2K2 | TTBK1 |  |
| HOMER1 | | MAEA | EMC8 |  |
| NIPAL2 |  | 9330161L09RIK | EML4 |  |
| BROX |  | CXCL10 | GRM8 |  |
| SLC11A2 |  | HSD17B7 | UBE2Q1 |  |
| CNTNAP2 | | BEST1 | PGLS |  |
| APOL6 |  | PEX19 | CAPZA1 |  |
| TYW3 |  | SIK3 | CDC23 |  |
| SPECC1 |  | STAB1 | DKC1 |  |
| SLC25A43 | | GMDS | HMGN2 |  |
| PPP1R9A | | TRIM12C | ARHGEF9 | |
| CLDN19 |  | TMEM145 | SLITRK1 |  |
| TIRAP |  | CHRD | CHM |  |
| ATP13A5 | | RAPGEF6 | FAM129B | |
| DENND6B | | EFCAB5 | MBLAC2 |  |
| CHRNA1 |  | MON2 | UBE2M |  |
| B230359F08RIK | | 1700029I15RIK | PTPRJ |  |
| BAAT |  | TAB2 | CHGA |  |
| SMYD3 |  | WDR17 | SLC25A51 | |
| KPNA4 |  | NID2 | HSD17B11 | |
| BTN1A1 |  | EPOR | COMMD1 | |
| BCHE |  | ENSA | CHRM1 |  |
| TANC2 |  | RNF32 | FAM234A | |
| SMAD2 |  | DES | DMWD |  |
| SLC26A3 |  | GM20075 | STX7 |  |
| GDA |  | TSHB | SURF4 |  |
| 3110052M02RIK | | SEMA4F | NDUFV3 |  |
| DTX4 |  | MUC15 | PREX2 |  |
| DDX19B |  | CBR1 | OSBPL9 |  |
| TUBA8 |  | EFCAB8 | FAM45A |  |
| POU2F1 |  | CC2D1B | GRIP1 |  |
| ZFP507 |  | PCDHA2 | ATXN2L |  |
| TMEM151B | | TRAPPC2 | IQGAP1 |  |
| SH2D4B |  | HHAT | UBE2F |  |
| SH3TC2 |  | WDR43 | CBL |  |
| NRG4 |  | PDZRN4 | FAM171B | |
| LURAP1 |  | GPRIN1 | HAGHL |  |
| HOOK3 |  | TAF11 | FAM114A2 | |
| EIF6 |  | CDKL3 | PPP1CC |  |
| TRAF5 |  | RABEP1 | WDR6 |  |
| NDST1 |  | CNTN4 | PIK3R2 |  |
| PON3 |  | RCVRN | HDAC2 |  |
| HOGA1 |  | ZRSR2 | MRPL46 |  |
| GABPB2 |  | ZMYM1 | SH3BGRL3 | |
| PRPF4B |  | ID4 | REPS1 |  |
| CD180 |  | TTC41 | IGF2R |  |
| DRG1 |  | TSTD3 | GOLGA2 |  |
| KRBA1 |  | PIF1 | TTC9 |  |
| MAP4K5 |  | RSG1 | HIST3H2BB | |
| HVCN1 |  | HIST1H2BP | EFNB1 |  |
| HN1L |  | FKBP10 | RMDN1 |  |
| 2510049J12RIK | | MAK16 | TMX3 |  |
| TCERG1 |  | SHANK1 | UFC1 |  |
| BC106179 | | MESD | KIFAP3 |  |
| PCYT1B |  | SPATA9 | NCSTN |  |
| CNR1 |  | NXN | ATG2B |  |
| SNED1 |  | HYAL1 | GNG5 |  |
| SEMA3C |  | GM13420 | HMGB3 |  |
| TMEM212 | | MVP | PI4K2A |  |
| ARHGEF17 | | A2M | POGLUT1 | |
| FLRT1 |  | USH2A | RHOF |  |
| ZFP300 |  | NME9 | NT5E |  |
| BC026585 | | SORD | SYNPR |  |
| RC3H2 |  | DERA | GNS |  |
| RAB39B |  | HSPBAP1 | ALPL |  |
| DLG2 |  | FNDC10 | KCNIP4 |  |
| SLC22A2 |  | PIGW | OSGEP |  |
| TMEM80 |  | BMP6 | PTPMT1 |  |
| AGO1 |  | TRIOBP | MBOAT7 | |
| 9230109A22RIK | | AI593442 | PLEKHG5 | |
| KLRB1F |  | HMOX2 | RPS29 |  |
| 9630033F20RIK | | NDUFV3 | TM9SF3 |  |
| CUL5 |  | SVEP1 | MPDZ |  |
| FBXO47 |  | EGR4 | PIP5K1A |  |
| WNT5A |  | UBTD2 | PPP1R12B | |
| DDIT4L |  | INCA1 | CNTN3 |  |
| TXNRD1 |  | STRAP | HDHD3 |  |
| TARM1 |  | RAB18 | TTC1 |  |
| PMS2 |  | DTNB | CYP51A1 | |
| VTCN1 |  | FAM241A | TUSC3 |  |
| MECP2 |  | VSNL1 | NDUFAF3 | |
| CREM |  | GM38393 | AP3S2 |  |
| MEMO1 |  | AFTPH | KSR2 |  |
| ZFP873 |  | ZFP661 | CNR1 |  |
| CBFA2T3 | | LMF1 | CNPY3 |  |
| 4930415F15RIK | | DYNLT3 | JPT1 |  |
| ZBTB9 |  | CDC34B | AGPAT5 |  |
| WSB2 |  | COL9A2 | SCN1B |  |
| 1190007I07RIK | | FBXO33 | HECTD1 |  |
| RASGRP4 | | GM14412 | MANF |  |
| NEU1 |  | 1700021F05RIK | XRN1 |  |
| GTPBP3 |  | ZFP619 | ELP2 |  |
| CD300A |  | RET | SBNO2 |  |
| WIPF3 |  | GM5136 | IREB2 |  |
| HIST1H2BB | | TLE3 | MPC1 |  |
| SHISA7 |  | AGL | CHCHD4 |  |
| ARL14EP | | 5430403G16RIK | NSUN2 |  |
| CD46 |  | LRRC47 | TBC1D23 | |
| ARHGAP24 | | FSTL1 | AIMP2 |  |
| COMMD1 | | HIST1H3D | HMGA1 |  |
| IFI203 |  | UBE2D1 | MRPL4 |  |
| VEZT |  | RSPH3B | RBM42 |  |
| RAB11A |  | UBXN6 | NDUFA1 |  |
| NADSYN1 | | FBXO31 | RABGAP1L | |
| RFX6 |  | HPF1 | PFKFB2 |  |
| KCNJ2 |  | RDH11 | FAU |  |
| IRF2BPL |  | 2310034G01RIK | MTIF2 |  |
| TOMM20 | | VRK2 | SLC7A8 |  |
| TRIM30D | | 4931414P19RIK | CEP290 |  |
| CCL28 |  | GREM2 | MGRN1 |  |
| ZFAND5 |  | ARG2 | KRAS |  |
| OTUD6B |  | PAG1 | PPP6R2 |  |
| CLCN5 |  | MAP3K6 | TTC9B |  |
| GPR1 |  | CCDC69 | FBXO6 |  |
| ZFP109 |  | DCLK1 | MED14 |  |
| NCAPG2 |  | DENR | SH2B1 |  |
| ALKBH8 |  | CHAC1 | BMPR2 |  |
| SUMO2 |  | SLC7A7 | MAGOH |  |
| SMAP2 |  | UHRF1BP1L | WASHC2 |  |
| LIMD2 |  | BROX | FAF1 |  |
| ETS1 |  | MINDY4 | LRRTM4 |  |
| TNFAIP8L1 | | TEX19.2 | LSM4 |  |
| 9930021J03RIK | | RGS21 | MBNL2 |  |
| SPATA25 | | GM7247 | DOCK2 |  |
| TFEC |  | CPVL | UBLCP1 |  |
| ACSM2 |  | OLFR993 | S100A16 |  |
| URGCP |  | FAM170A | CHMP2B |  |
| NUP160 |  | CD19 | DPP9 |  |
| ROBO4 |  | RRAGD | EIF1A |  |
| LRRC9 |  | DHX32 | HIST1H1D | |
| AMER2 |  | CHM | TUBB2B |  |
| DDX51 |  | PPIB | DYTN |  |
| TTLL1 |  | USP21 | PHLDA3 |  |
| SLC26A10 | | 0610010F05RIK | PTBP1 |  |
| AU022252 | | HHIPL1 | TUBG1 |  |
| FCHO1 |  | VIT | HECW2 |  |
| GRIN2A |  | AAK1 | BROX |  |
| CYP7B1 |  | MTPN | TIMM10B | |
| 2610002M06RIK | | NAA11 | SRP54 |  |
| CYP4V3 |  | IRGM2 | MPRIP |  |
| AKR1E1 |  | IRF2BP2 | C2CD5 |  |
| SGCD |  | EPM2A | ALG1 |  |
| RUFY2 |  | ANKLE1 | CPT2 |  |
| SSX2IP |  | VIL1 | GNPDA2 |  |
| CBX6 |  | B3GAT3 | UBE4B |  |
| ZSWIM4 |  | SETD3 | UCHL5 |  |
| SATB2 |  | 4930595D18RIK | CCDC127 | |
| PLEKHM2 | | NAF1 | FAM160B1 | |
| PXDN |  | MYCBP2 | SNF8 |  |
| TBC1D9B | | MIA3 | TMEM43 |  |
| SOS2 |  | TIFA | RAB33B |  |
| VAT1L |  | DAGLB | ENTPD2 |  |
| CNTN4 |  | EPHA2 | CHMP3 |  |
| CD209B |  | LDLRAD3 | APOA1 |  |
| VASP |  | CR2 | MACROD2 | |
| KCNJ8 |  | DPH3 | PNKD |  |
| ZFP882 |  | FRMD4B | PFDN6 |  |
| MAP3K9 |  | TCEAL1 | SCRG1 |  |
| MMD2 |  | COX8B | TMED4 |  |
| HOXB9 |  | TVP23B | CMBL |  |
| CYP2J13 |  | SRCAP | MCEE |  |
| REST |  | 4930579G24RIK | SF3B6 |  |
| SPATA6 |  | TMEM192 | CPNE2 |  |
| SLC7A2 |  | CCND3 | PRKAA2 |  |
| IL15 |  | USF1 | STXBP6 |  |
| MAN1A2 |  | HOMEZ | DHRS7 |  |
| SAP30L |  | RHOBTB3 | HDGF |  |
| ZWILCH |  | RHOJ | DOC2A |  |
| WARS2 |  | PDE4DIP | PFDN1 |  |
| MIER3 |  | OCM | SLC7A11 |  |
| ACR |  | BATF3 | NDUFAF4 | |
| SLC27A4 |  | SLC6A12 | PXN |  |
| ABT1 |  | H2-M10.2 | NPEPL1 |  |
| ANKS4B |  | RGN | CHTOP |  |
| FIGN |  | TMEM183A | CP |  |
| PDIK1L |  | CCDC85A | RAP1B |  |
| RDH14 |  | INMT | RPS6KA5 | |
| PDE7A |  | SNRPF | ATG5 |  |
| COL4A3BP | | KLC2 | VAMP1 |  |
| CCDC114 | | SUMO3 | LSM8 |  |
| RNF217 |  | KRT222 | GFER |  |
| PSMB5 |  | MAPK6 | ACYP2 |  |
| SNX21 |  | ZFP97 | HEXA |  |
| NDRG2 |  | EXTL1 | ENDOD1 |  |
| SMAD4 |  | SYT7 | TGFBRAP1 | |
| TROVE2 |  | GABPA | MINDY2 |  |
| ZFP40 |  | DNHD1 | RAP2C |  |
| CCDC129 | | ACOT10 | HSPB1 |  |
| SELPLG |  | CCDC190 | PRRC1 |  |
| PPP2R2A | | ZFP101 | TRIAP1 |  |
| TLL1 |  | UQCC1 | TMCO1 |  |
| PDE4C |  | GM14410 | TBCE |  |
| HIST1H2BE | | NDUFV1 | TBCC |  |
| CNIH3 |  | KDM5D | BANF1 |  |
| ZFP13 |  | FBXO41 | RAB33A |  |
| DBNDD2 |  | TSR2 | ADCY1 |  |
| PPFIA2 |  | FILIP1 | HMBS |  |
| FAR2 |  | SRGAP1 | RPS28 |  |
| MPG |  | RAB33A | MVB12B |  |
| TAS2R135 | | RGS9BP | MRS2 |  |
| RMDN1 |  | BSN | MAP9 |  |
| CDYL2 |  | GSTA4 | EXD2 |  |
| FLRT2 |  | SNAI3 | HIST1H1A | |
| 1700013F07RIK | | ZFP462 | PPP1R16A | |
| PRIMA1 |  | ADAMTS4 | HERC4 |  |
| APOO |  | ASIC1 | PRPF40B | |
| 2610028H24RIK | | SLC9A7 | RPRD2 |  |
| LRRC10 |  | RYR2 | RMND5A | |
| AKR1C14 | | TPM4 | ELMO1 |  |
| CNGA3 |  | USP46 | NDUFAF1 | |
| ZFP786 |  | CYP2W1 | SYT3 |  |
| PTCHD4 |  | SPATA20 | MRPS26 |  |
| RRP7A |  | CELF3 | ZNFX1 |  |
| LECT2 |  | KATNB1 | MRPS15 |  |
| ZFP420 |  | SLC4A11 | CPPED1 |  |
| RSF1 |  | SP100 | ARAP2 |  |
| SNRNP35 | | RAB19 | AAMDC |  |
| BC035044 | | CCDC105 | UBE3C |  |
| ARRDC3 |  | TREX1 | ABHD10 |  |
| TUSC2 |  | EFR3B | RANBP6 |  |
| ASPA |  | TSC22D3 | CAMK2N1 | |
| SLFN9 |  | RRP36 | SMAD4 |  |
| SMCHD1 |  | SFXN3 | GALK2 |  |
| TRIP4 |  | RAB25 | NEK7 |  |
| KIF3C |  | SLC18A1 | SPATS2L | |
| D430041D05RIK | | EIF2S2 | MTFP1 |  |
| PPP1R3A | | FAM171A2 | AKT3 |  |
| MDGA2 |  | FKBP8 | ZNF428 |  |
| RTKN2 |  | ICE2 | WASHC3 |  |
| ZFP664 |  | HIST1H3H | APBA1 |  |
| RBPJL |  | CAR1 | SHC3 |  |
| LIPA |  | VPS72 | PTPN1 |  |
| SSFA2 |  | ZSWIM5 | SLC25A42 | |
| ENTPD3 |  | HIC1 | ZFAND2B | |
| ENO1 |  | NUP210 | SARAF |  |
| ALKBH1 |  | STAMBP | LPCAT4 |  |
| 1700028P14RIK | | PDZD9 | CDC73 |  |
| FBXL22 |  | TOMM70A | TPMT |  |
| ABHD6 |  | KCNK15 | YME1L1 |  |
| PHEX |  | FBXL6 | COX7C |  |
| NEUROD4 | | YTHDC2 | BCL2L2 |  |
| FOXN3 |  | ACVR1 | MRPL10 |  |
| EXOC6B |  | CSNK1G2 | OPA3 |  |
| TOX2 |  | PMFBP1 | KCNH1 |  |
| IKBKB |  | AFF4 | UBXN7 |  |
| WNK3 |  | ARMCX1 | TM9SF4 |  |
| SLC44A1 |  | EHMT1 | RAB9B |  |
| GM6086 |  | TBX2 | MAL2 |  |
| PNMA3 |  | GAR1 | PISD |  |
| MCC |  | USP43 | SH2D5 |  |
| FAM13A |  | ETV5 | FDX2 |  |
| DTX1 |  | ZBTB33 | TOMM6 |  |
| MAPK4 |  | AFF1 | XPOT |  |
| BZW1 |  | PCDHA11 | SMNDC1 |  |
| PLCH1 |  | SLC25A18 | RPRD1A |  |
| ZBTB10 |  | VAMP9 | PRPS2 |  |
| PDPK1 |  | SART1 | ERGIC1 |  |
| RPS6KA2 | | UPF1 | LAMTOR1 | |
| TBX22 |  | GM136 | SMARCE1 | |
| WNK1 |  | GGT6 | HERC2 |  |
| CREBRF |  | IZUMO1R | CBARP |  |
| HECW1 |  | RBM41 | RMC1 |  |
| KBTBD7 |  | DMPK | ARPP19 |  |
| WSCD2 |  | TWF2 | P33MONOX | |
| SLC2A10 |  | LRRC55 | ARC |  |
| TPM3 |  | EI24 | VPS37C |  |
| FAM132B | | EMP2 | PEX5 |  |
| GM13251 | | SLC37A3 | KPNA1 |  |
| SPTY2D1 | | PODXL2 | ACSF3 |  |
| PEG10 |  | GRIK5 | DKK3 |  |
| SACS |  | NANOS2 | FLAD1 |  |
| ZFP408 |  | RAMP2 | SNX17 |  |
| FRMPD4 |  | ARHGEF5 | APLP2 |  |
| DNAJC6 |  | PCDHGB7 | TIGAR |  |
| B3GAT1 |  | CLCA3B | NTNG1 |  |
| NFATC4 |  | TMEM114 | CCDC92 |  |
| PDZD2 |  | KIF21B | ARL2 |  |
| CTNS |  | KRCC1 | CPNE8 |  |
| EPAS1 |  | CCDC22 | LETMD1 |  |
| ADAMTS12 | | MTMR1 |  |  |
| CSMD1 |  | FHL5 | CARM1 |  |
| FAM178A | | GPN3 | ARMCX3 | |
| TPPP |  | FGF12 | MAP4K3 |  |
| GM10521 | | GRAMD3 | MCTP1 |  |
| NHSL1 |  | GPC6 | FIP1L1 |  |
| SLITRK4 |  | GLIPR2 | TRAPPC5 | |
| SUGP2 |  | FAM241B | SIN3A |  |
| FAM189A1 | | GXYLT2 | FOLH1 |  |
| GCNT4 |  | MRAS | IMPAD1 |  |
| WDR76 |  | UBE2CBP | ARHGEF10L | |
| COL26A1 | | CDKN1B | ANP32E |  |
| USP29 |  | RCOR3 | NCK1 |  |
| DDX58 |  | ATP8B1 | NT5M |  |
| PAK3 |  | NOTCH3 | MRPS34 |  |
| OLFR60 |  | ATP12A | DDC |  |
| LRCH3 |  | TRIM6 | DCXR |  |
| HTR1A |  | RAG2 | ABCB6 |  |
| BICD2 |  | OMG | ATPAF1 |  |
| ADAMTSL3 | | GM10269 | PPOX |  |
| SPOCK2 |  | EIF3I | DHCR7 |  |
| TET2 |  | SLTM | CHMP6 |  |
| MYOM3 |  | RITA1 | SERPINA1B | |
| FAM196A | | GDF7 | VKORC1L1 | |
| FUT4 |  | MECR | FLRT2 |  |
| DDI2 |  | SPATA16 | EIF2B1 |  |
| CN725425 | | CYLD | CTNNBL1 | |
| CPLX2 |  | STK3 | UBE2R2 |  |
| PRDM15 |  | LRRC23 | PPP1R3F |  |
| TUB |  | STK33 | IBA57 |  |
| PRDM4 |  | MDH1 | CHAT |  |
| USP9X |  | DCPP3 | TBC1D10A | |
| LRRC4 |  | TNS3 | UCK1 |  |
| PTPRT |  | CLDN20 | SSR4 |  |
| GFI1 |  | CEP83 | MAP3K7 |  |
| RGS16 |  | TMEM200A | APOOL |  |
| CSRNP3 |  | LIMA1 | HDHD5 |  |
| DCX |  | GSG1 | SMPD4 |  |
| EIF5A2 |  | COQ8B | NRBP2 |  |
| HTR1B |  | LMAN2L | SCAMP3 |  |
| MUC15 |  | TMEM132D | CRTAC1 |  |
| HUNK |  | ARSG | ANKRD34B | |
| LMX1B |  | PKD1L1 | HSPA12B | |
| GDF7 |  | BEST3 | EPHA5 |  |
| ADAMTS15 | | PAK3 | GRID1 |  |
| UNC80 |  | ARL6IP1 | PITPNM3 | |
| ADAMTS4 | | ZDHHC24 | PDE12 |  |
| APOBR |  | GPR63 | TTC39C |  |
| A530054K11RIK | | PROKR1 | PTP4A2 |  |
| GPR152 |  | MOSMO | CELSR2 |  |
| SSTR2 |  | XYLT1 | WBP11 |  |
| ABCG4 |  | D830030K20RIK | AKT2 |  |
| HS6ST3 |  | MDH2 | C1QA |  |
| MAP3K1 |  | TMEFF2 | PEX11B |  |
| KLK7 |  | LAT | BCAR1 |  |
| SCN1A |  | TAOK2 | PPP1R14A | |
| FAM199X | | SEMA6A | MEF2D |  |
| CACNA2D2 | | VIPR2 | USP8 |  |
| MAP9 |  | B4GALNT1 | ANAPC5 |  |
| OCRL |  | DHH | BABAM2 |  |
| SIPA1L3 |  | LGALS8 | MLST8 |  |
| 6030445D17RIK | | ACADVL | FSD1L |  |
| MTF1 |  | CNTLN | BPGM |  |
| FBXL7 |  | UNC5A | CDC16 |  |
| SLC1A7 |  | PTPRU | GPT2 |  |
| MAPK1 |  | PCED1A | SLC25A24 | |
| CD200 |  | RMND1 | MT1 |  |
| JPH4 |  | EPDR1 | CPT1C |  |
| MCF2L |  | MBNL1 | ADGRB2 |  |
| HTR2C |  | ACER3 | CACNB1 |  |
| NKX6-3 |  | GAS2L2 | CTIF |  |
| IKZF4 |  | KDELC1 | RTF1 |  |
| ARHGAP26 | | TMEM123 | ZRANB2 |  |
| SNTB1 |  | GM7233 | COQ6 |  |
| SET |  | GM867 | ZW10 |  |
| GRIN3A |  | CSF1R | ANKRD13D | |
| MS4A4B |  | BCL2L12 | ILDR2 |  |
| PIKFYVE | | MOB3B | CLPP |  |
| MMP11 |  | TUBG2 | PRRT3 |  |
| CDH6 |  | GPAA1 | EPHA7 |  |
| ALK |  | C2CD3 | ISLR2 |  |
| PCDHGA12 | | HEATR5A | RPL36A |  |
| IKZF2 |  | CACNA1F | PDP1 |  |
| CHDH |  | MMP16 | RBMS1 |  |
| TAOK1 |  | NKX2-2 | CHN1 |  |
| ECE1 |  | NDEL1 | GNG10 |  |
| ITGA1 |  | BANP | TMEM201 | |
| LDLRAD1 | | DVL3 | DENND4B | |
| DEPDC1B | | SSR2 | COMMD6 | |
| ACCS |  | TMEM199 | PSPH |  |
| ATXN7 |  | CRHR2 | GOLGA3 |  |
| RAPGEF6 | | SPATA32 | ABCB9 |  |
| MARF1 |  | LOXL2 | RPL35 |  |
| LATS1 |  | FCGR3 | WDR5 |  |
| ZFP148 |  | DUSP14 | TTR |  |
| DLC1 |  | ATP5G3 | PLRG1 |  |
| SLC2A1 |  | GM42517 | SLC12A9 |  |
| STAR |  | ADAM9 | THOC2 |  |
| SCN3B |  | CCPG1OS | PMM2 |  |
| GM10221 | | FAM169A | AKTIP |  |
| MAST3 |  | 1700008O03RIK | PIGU |  |
| POLDIP3 | | CTLA2A | CSAD |  |
| ZBTB16 |  | EEF1A1 | LAMP2 |  |
| TNPO1 |  | SLC2A8 | XPNPEP3 | |
| CCR5 |  | APEX1 | NUBPL |  |
| ARID3A |  | GRHL2 | WDR91 |  |
| PSIP1 |  | GSE1 | NTMT1 |  |
| 9430020K01RIK | | YWHAH | CCDC93 |  |
| KIF1A |  | ISG20 | CTH |  |
| STAT3 |  | NSMCE1 | NRAS |  |
| NT5C1A |  | SLC25A45 | MRPS9 |  |
| BCL2 |  | OAS1A | BABAM1 |  |
| APBA1 |  | TGM3 | SNX32 |  |
| BC067074 | | ISCA2 | ENY2 |  |
| LATS2 |  | PITPNM2 | RALBP1 |  |
| TGFBR3 |  | VPS26A | TMCC1 |  |
| KIRREL |  | SUN2 | PGAM2 |  |
| GLTSCR1L | | CBS | KAZN |  |
| OLFML2A | | TENM2 | KCNA1 |  |
| CST8 |  | PRDM8 | GYG1 |  |
| MAP4 |  | NUDT17 | UBE2H |  |
| TRIM65 |  | SUPT7L | REEP5 |  |
| MAN2A2 |  | GM3264 | CNOT9 |  |
| NHS |  | RBM11 | SLC22A23 | |
| DYRK1A |  | GM21981 | TTLL12 |  |
| TMEM125 | | RIOX2 | ABCA8B |  |
| RIC3 |  | MEI4 | PCNX1 |  |
| TSPAN4 |  | S100A10 | YLPM1 |  |
| RICTOR |  | INSM2 | ANGEL2 |  |
| SKIL |  | KCNAB3 | BOLA2 |  |
| PTPN4 |  | GPC5 | BAG4 |  |
| 1700052N19RIK | | PNMAL2 | MIEN1 |  |
| ADO |  | DCAF8 | TMEM160 | |
| PARP8 |  | NUMB | ACOX3 |  |
| CDK6 |  | ZFP771 | RNF141 |  |
| SMIM14 |  | PPP3CC | KIF21B |  |
| MARK1 |  | DYRK4 | TMEM33 |  |
| ITGA2 |  | SMARCA4 | MRPL37 |  |
| SOX11 |  | GALR3 | CHMP2A |  |
| BC094916 | | PLGRKT | CASTOR2 | |
| CHM |  | GATAD1 | TMEM109 | |
| GFOD1 |  | CAB39 | CISD2 |  |
| TRAK2 |  | COPA | STX4 |  |
| SYNJ1 |  | IFI203 | RELL2 |  |
| TLE4 |  | COL20A1 | TFIP11 |  |
| POU2F1 |  | DHRS11 | DDHD2 |  |
| GFRA1 |  | PALD1 | COPE |  |
| ALOX5AP | | 1110004F10RIK | DPY30 |  |
| BDKRB2 |  | WNK2 | CDK11B |  |
| ARID5B |  | CENPK | FAIM |  |
| HNRNPA3 | | PALB2 | ARHGAP25 | |
| FAM198B | | UBXN1 | CLCN6 |  |
| RAB42 |  | FRAT2 | SORCS1 |  |
| GJB4 |  | ZFP334 | TINAGL1 | |
| CD40 |  | DLX1 | YTHDC1 |  |
| DENND6A | | GPN1 | TIMM17A | |
| ZSCAN26 | | LONRF2 | GFOD1 |  |
| NKX2-5 |  | ARSK | PABPN1 |  |
| BBOX1 |  | GM14440 | WASHC4 |  |
| TNFAIP1 |  | CRYZ | SMARCA4 | |
| RAPGEF3 | | KLHL31 | LRRFIP2 |  |
| CCDC68 |  | PPDPF | WDR82 |  |
| KLF13 |  | RNF220 | SNRPA1 |  |
| VPS33A |  | METRN | SNRPG |  |
| CCDC122 | | RASD1 | PHF5A |  |
| TMEM132B | | 4933408B17RIK | RPL19 |  |
| GAS2L3 |  | RHPN2 | TBCEL |  |
| ZHX3 |  | VPS9D1 | EPG5 |  |
| ARFGAP1 | | ATAD3A | CPOX |  |
| PURA |  | DUSP3 | SYPL1 |  |
| SPNS3 |  | CRYL1 | PSMD7 |  |
| LPGAT1 |  | DNAJB13 | NAGK |  |
| DSEL |  | WASF2 | MRPL3 |  |
| AAK1 |  | HLF | HABP4 |  |
| HAO1 |  | NUBP1 | KIAA0100 | |
| NME2 |  | MYO7A | UBR2 |  |
| RAPH1 |  | TMEM132B | ACAN |  |
| ICA1L |  | GRP | AKR1B8 |  |
| PEX5L |  | ZER1 | MIEF1 |  |
| GIMAP3 |  | CHIL5 | PRELP |  |
| RASAL2 |  | PRKDC | NPC2 |  |
| FSD2 |  | UCK1 | CLCN3 |  |
| TRIM25 |  | EP400 | PTPRG |  |
| C130074G19RIK | | IFT81 | SYAP1 |  |
| CPNE3 |  | ASGR1 | TBCA |  |
| DYRK2 |  | GSTCD | PRUNE2 |  |
| PCDHB21 | | PDE1C | KCTD13 |  |
| ITCH |  | RIPOR3 | SPRYD7 |  |
| UFL1 |  | ATP4A | PRAF2 |  |
| STRBP |  | RBSN | ADCK1 |  |
| CREB1 |  | CTTNBP2NL | IRF2BPL |  |
| GLTSCR2 | | SLITRK4 | COMMD8 | |
| OXSR1 |  | NTMT1 | LPIN2 |  |
| FMNL2 |  | ALG9 | NRBP1 |  |
| GABPA |  | BLM | IRF3 |  |
| F930015N05RIK | | LEP | RDH11 |  |
| CYTIP |  | CD44 | LARP1 |  |
| STAT5A |  | TMEM189 | SYNRG |  |
| BC003331 | | NUSAP1 | CLCC1 |  |
| CHIC2 |  | IZUMO4 |  |  |
| ZSCAN29 | | DBH | RBSN |  |
| HMMR |  | TOMM7 | DPP7 |  |
| CIR1 |  | PAMR1 | TBC1D9B | |
| GMCL1 |  | CPEB2 | PRKG2 |  |
| GLG1 |  | TRPC7 | PAFAH1B3 | |
| TNRC6C |  | SECISBP2L | LYST |  |
| HR |  | ZFP423 | SELENOF | |
| EHD2 |  | POLR2E | FBL |  |
| TMTC1 |  | AMN | NUP214 |  |
| MAML2 |  | DYDC2 | EPHB6 |  |
| XCR1 |  | SLC16A13 | AMT |  |
| RORA |  | GIT1 | ABCA3 |  |
| MS4A4D |  | RPL19 | DHDH |  |
| UBE2J1 |  | PRNP | DGLUCY | |
| ATP11C |  | RAB40C | NUDT5 |  |
| 6430571L13RIK | | SNAPC5 | KRT10 |  |
| CHIC1 |  | SEMA3B | NRP1 |  |
| MAPK8 |  | XPOT | BORCS8 |  |
| LRRC8B |  | KBTBD13 | P2RY12 |  |
| ATP7A |  | KCNK1 | KIAA1522 | |
| TAZ |  | CNIH4 | EMC10 |  |
| GEMIN4 |  | HBB-BS | BAZ1B |  |
| PGAP1 |  | VPS37C | WASHC5 |  |
| MAPRE2 |  | KNDC1 | GGT1 |  |
| LRP2 |  | BCL2L2 | VPS41 |  |
| SAMD5 |  | MYT1 | PRKAB2 |  |
| ABRA |  | CAVIN1 | SMARCA5 | |
| CLASP1 |  | SPPL2C | CNTNAP5A | |
| GM53 |  | AK2 | UBE3B |  |
| CETN2 |  | KLHL32 | PHKA1 |  |
| GTF2A2 |  | MGP | KCNJ10 |  |
| MFAP3 |  | DSE | RPL34 |  |
| MUC20 |  | HUS1B | RAB30 |  |
| ST3GAL6 | | PTRH1 | PALMD |  |
| CAPN5 |  | LMAN2 | PTPRE |  |
| SLC16A7 |  | MRPL47 | AFTPH |  |
| TMEM126B | | ELMSAN1 | WARS2 |  |
| JAM3 |  | CCR10 | PIGT |  |
| PEX10 |  | RGS22 | GCAT |  |
| AP1G1 |  | BHMT2 | CEP131 |  |
| EMC3 |  | ATAT1 | STAM2 |  |
| PTEN |  | KCNK2 | VPS4B |  |
| ATP6V1D | | FAM186B | CDK16 |  |
| TCP11L1 |  | CSK | R3HDM2 |  |
| ATRN |  | NLRC5 | COQ3 |  |
| RASGRF2 | | IRS4 | RALYL |  |
| DNAJC5 |  | EIF1 | ZNF706 |  |
| TRAF6 |  | NOP14 | CTSF |  |
| 9030624J02RIK | | PCMTD1 | GAS2L1 |  |
| H2-DMB1 | | LPAR1 | FAIM2 |  |
| SNX13 |  | ZFAND5 | NUP62 |  |
| RIPK1 |  | PLSCR1 | BLOC1S2 | |
| LURAP1L | | 1700123O20RIK | CARMIL1 | |
| ITGBL1 |  | P2RX3 | CTSA |  |
| PDXDC1 |  | EMC6 | MTND4 |  |
| VCPIP1 |  | TFEB | TRAPPC12 | |
| GPC6 |  | MYOF | LYRM4 |  |
| EDC3 |  | ASNS | MFSD6 |  |
| REEP3 |  | XRN2 | TMEM35A | |
| FAM222B | | MED31 | WASHC1 |  |
| EIF5B |  | KSR2 | ERO1A |  |
| AZI2 |  | COA7 | STK4 |  |
| HIST1H2AD | | GRAMD1C | DTX3 |  |
| CCDC171 | | SH3RF3 | MCAM |  |
| EPHA7 |  | KISS1R | CLEC2L |  |
| MAP3K19 | | ISYNA1 | MYLK |  |
| USP31 |  | OTUD7B | PRICKLE1 | |
| MILL2 |  | FAM129B | TOR1AIP1 | |
| COL13A1 | | RPAIN | DPP8 |  |
| MAP7D1 |  | EXOC6B | PAK5 |  |
| ATP6V1B1 | | PSAPL1 | SLC25A25 | |
| GM20390 | | ZFP948 | PCDHGA4 | |
| BICD1 |  | CTSS | LMAN2L |  |
| NOL3 |  | UBE2V1 | CDC27 |  |
| RRAGD |  | TMED1 | ARGLU1 |  |
| SLC22A23 | | HTR3A | NAPEPLD | |
| TMEM56 |  | HIST3H2A | NUDCD2 |  |
| ELF1 |  | LRRC69 | WWOX |  |
| CAPRIN1 | | FAM196B | CDIPT |  |
| FNBP1 |  | SPATA3 | PDE4A |  |
| THNSL1 |  | NAE1 | CCDC124 | |
| ZBTB1 |  | 1810010H24RIK | NRP2 |  |
| TMEM127 | | PRKAG1 | SHISA6 |  |
| NFIX |  | DONSON | ABCA2 |  |
| PRSS23 |  | SLITRK1 | IRF2BP1 |  |
| FZD3 |  | FTH1 | TTPAL |  |
| SLIT2 |  | CAR10 | MRPL16 |  |
| POGLUT1 | | ZFP35 | MAEA |  |
| PHACTR1 | | TMPRSS4 | PANK1 |  |
| AI429214 |  | PALM2 | SELENOW | |
| CLMP |  | ITGA5 | MT-CYB |  |
| LARS2 |  | LRRC9 | ACBD5 |  |
| SERINC5 |  | ZFP811 | NELFB |  |
| USP13 |  | OLFR554 | UBL3 |  |
| F8 |  | MT-ND2 | CAMLG |  |
| ITPR2 |  | CDK19 | NDUFB1 |  |
| KY |  | TXN2 | MIPEP |  |
| S100B |  | CSDE1 | UBR3 |  |
| E2F3 |  | PCDHGA4 | ARFGAP2 | |
| SASS6 |  | CDKL4 | YTHDC2 |  |
| PMEPA1 |  | CD8A | NDUFA3 |  |
| CD109 |  | PLEKHH2 | AP3B1 |  |
| METTL10 | | POLDIP3 | PCNA |  |
| ETV1 |  | ZFP36L2 |  |  |
| CIT |  | EIF2AK1 | PTCD1 |  |
| NOVA1 |  | SLC38A7 | RPL37 |  |
| PPIG |  | TMEM267 | SMCR8 |  |
| IFT52 |  | SBSN | LRBA |  |
| DNAJC9 |  | GSTA3 | EHD2 |  |
| NAALAD2 | | STX6 | NUP155 |  |
| KCNJ16 |  | BC035044 | KBTBD3 |  |
| ZFP758 |  | OTUD6B | BTF3 |  |
| SGMS2 |  | KHDC4 | CUTC |  |
| CLEC4E |  | MROH4 |  |  |
| YTHDF3 |  | RAB11FIP3 | RAC3 |  |
| UBALD2 |  | CNGA4 | LSM5 |  |
| PIGM |  | PTGIS | RPS26 |  |
| TNS1 |  | POLB | MYH1 |  |
| LSM1 |  | NAA15 | MPDU1 |  |
| DSC1 |  | MLIP | SLC8A3 |  |
| RNF139 |  | TUBA1B | MCF2L |  |
| PTCHD2 |  | PUDP | PTGDS |  |
| GM11444 | | OLFR1312 | OLFR148 |  |
| TTBK1 |  | C130026I21RIK | RP2 |  |
| NVL |  | CLCNKB | MTPAP |  |
| FCGR2B |  | CCL26 | ATP5MC2 | |
| IKZF3 |  | GAST |  |  |
| CXCL15 |  | TMPRSS12 | CHP1 |  |
| ACP2 |  | CD300LD4 | AP1S1 |  |
| MYL6 |  | ZFP708 | VAMP3 |  |
| SMIM19 |  | MRGPRB5 | GABRB3 |  |
| FRK |  | GTSF1L | SERPINA1D | |
| NSF |  | SIGLECG | TAMM41 | |
| E430018J23RIK | | P4HTM | FAM160A2 | |
| DNAL1 |  | NXPE4 | PRKAA1 |  |
| ALPK1 |  | NWD2 | TGFB1I1 |  |
| GPR146 |  | DYRK1B | SNRPC |  |
| CAMSAP1 | | FAM221A | SPAG7 |  |
| VPS37A |  | GOLGA5 | GSTM7 |  |
| PPP1R36 |  | NREP | OSCP1 |  |
| HAPLN3 |  | CTTN | ARFRP1 |  |
| PEG12 |  | MRPL10 | THTPA |  |
| HSP90AA1 | | NIPSNAP2 | EXOSC4 |  |
| LRIG2 |  | POLG2 | POLR2H |  |
| COCH |  | MUC5B | EMC2 |  |
| BTC |  | CPT1C | LIPT2 |  |
| C77370 |  | RESF1 | LMAN1 |  |
| STON1 |  | CATSPER3 | PTP4A3 |  |
| ARV1 |  | SPACA3 | BOLA1 |  |
| CDK19 |  | TUSC5 | SLC25A19 | |
| FER |  | ECM2 | TLNRD1 |  |
| AP5Z1 |  | AP4S1 | NUDT3 |  |
| PHACTR2 | | PDE4A | SCN8A |  |
| MASTL |  | CHRDL1 | PGM2 |  |
| MTNR1A | | ANK2 | SMAD3 |  |
| TRIT1 |  | CUX1 | DHRS11 |  |
| TTBK2 |  | DCUN1D3 | DDRGK1 |  |
| TOMM70A | | TRIM46 | LCHN |  |
| HDHD2 |  | STX19 | STMN3 |  |
| PTPRD |  | FLT1 | EDC3 |  |
| SUPT4A |  | RUNX2 | SDF2L1 |  |
| ARHGAP5 | | RAP1GDS1 | IFT22 |  |
| FOXP1 |  | ZFP991 | BAG3 |  |
| UQCRH |  | CASP7 | CHMP5 |  |
| GRASP |  | SUGT1 | RLBP1 |  |
| LACTB2 |  | UBTF | NLRX1 |  |
| PPIL6 |  | GALNT13 | L2HGDH |  |
| MAP1A |  | SMURF1 | IPO11 |  |
| METTL21E | | MYCBP | USE1 |  |
| TPD52L2 | | NECTIN1 | RASGRP2 | |
| DZANK1 |  | POLD3 | GRN |  |
| SPRYD3 |  | HSPA12A | SMPD1 |  |
| RGS4 |  | PNPLA3 | GABRD |  |
| CNOT6 |  | PCDHGA12 | IGLON5 |  |
| RECQL |  | IGFBP3 |  |  |
| SLC5A4B | | LPIN3 | YES1 |  |
| ARHGEF12 | | RYBP | PSMD10 |  |
| FAM60A |  | ZFP941 | TMEM106B | |
| RPS6KA3 | | PKP4 | ELMOD1 |  |
| MROH6 |  | LAGE3 | STK25 |  |
| ZFP236 |  | CDSN | SHOC2 |  |
| SLC26A9 |  | MOCS2 | AIG1 |  |
| TTC14 |  | 7-Mar | PYGL |  |
| C1QTNF3 | | GM5737 | UBP1 |  |
| ALS2 |  | NEK1 | CSDC2 |  |
| ZFP597 |  | DNAJC12 | HDDC2 |  |
| SERPINA3M | | HDAC2 | PIK3CA |  |
| CBR4 |  | SAMD3 | MPV17 |  |
| CHSY3 |  | TM7SF2 | MRPL39 |  |
| CUTC |  | UBR2 | DESI1 |  |
| SCML4 |  | KCNMB4 | NPDC1 |  |
| CYB561D1 | | SNX17 | ZNF512 |  |
| SCAI |  | GFPT1 | PSMG1 |  |
| CCBL2 |  | BCL2L13 | MRPS28 |  |
| ANKRD9 |  | GM10309 | UVRAG |  |
| TTC39B |  | TRPM4 | ADAM10 |  |
| MYF6 |  | MRC2 | PGAP1 |  |
| TMEM263 | | PEX11B | RBFOX1 |  |
| TMEM5 |  | POLR1D | AHSG |  |
| CSGALNACT2 | | SSR3 | COQ5 |  |
| ARHGAP4 | | WLS | CLNS1A |  |
| NAA15 |  | ITGA2 | CRBN |  |
| MTIF3 |  | CFH | TSTD3 |  |
| MBD5 |  | PDAP1 | LINGO2 |  |
| LSG1 |  | SLC52A2 | CNTNAP4 | |
| SRRM4 |  | KRT8 | HBS1L |  |
| ENAH |  | SNTB1 | SLC6A4 |  |
| CHRNA7 |  | PLEKHF2 | NT5C2 |  |
| ASXL2 |  | CSPP1 | GCSH |  |
| SMARCAD1 | | CDK15 | IKBKG |  |
| 1700016K19RIK | | SERPINB5 | TNFRSF21 | |
| CCNY |  | GSTM2 | ABLIM3 |  |
| VPS4B |  | AHI1 | MTMR6 |  |
| LEAP2 |  | CABYR | AGPAT1 |  |
| CTDSP1 |  | RANBP3L | AMACR |  |
| GOLGA7 |  | SLC30A7 | FAM103A1 | |
| NMRAL1 |  | CCDC134 | ZYX |  |
| ZC3HAV1L | | RALGAPA2 | CHMP7 |  |
| CALB1 |  | 1700123K08RIK | CSNK1E |  |
| TMEM86B | | CCT3 | ABRAXAS2 | |
| DPT |  | CFAP298 | RAB2B |  |
| MAU2 |  | USP12 | LSM14B |  |
| OSBPL3 |  | HIST1H3A | MRI1 |  |
| OCSTAMP | | PREB | GRIA4 |  |
| WBSCR17 | | BCL2L10 | HSD11B1 | |
| TBL2 |  | SCGN | GAMT |  |
| PGM5 |  | TNK1 | KIFC2 |  |
| ZFP445 |  | 4930467E23RIK | SCARB2 |  |
| BAMBI |  | 2310003L06RIK | BAG1 |  |
| RASSF2 |  | TTC39C | NUDCD1 |  |
| ACSM5 |  | ZBTB32 | PELO |  |
| B9D1 |  | ZYX | PCBD2 |  |
| MIPOL1 |  | EIF4EBP2 | HTATIP2 | |
| OTC |  | HNRNPL | PRRT1 |  |
| STK32A |  | ITPRIPL2 | TNPO1 |  |
| FMO5 |  | RNF11 | MAPK8 |  |
| NIPA1 |  | GEM | CNNM3 |  |
| GCFC2 |  | ZFP367 | SNX9 |  |
| 1700019N19RIK | | ZFP236 | PPP1R16B | |
| MYOCD |  | UBR1 | BECN1 |  |
| ZFP599 |  | TRIM80 | DCUN1D3 | |
| TBP |  | FAM96B | PPIH |  |
| FAM160A2 | | EXOG | SQOR |  |
| KCNN3 |  | BCCIP | RALB |  |
| YIPF6 |  | PSEN2 | AGTPBP1 | |
| HDDC2 |  | TEX14 | PLPPR3 |  |
| CLMN |  | NEUROD6 | MRPS7 |  |
| TMEM106B | | TEX38 | GABRB1 |  |
| DCAF10 |  | COPB2 | SLC25A31 | |
| CUL4A |  | VLDLR | SCO1 |  |
| SLC22A6 |  | KIZ | LDB1 |  |
| MPDZ |  | IMPDH1 | UBA5 |  |
| FAAH |  | SLC25A38 | FCHO2 |  |
| CHMP4C |  | AQP12 | PTPN12 |  |
| LRRC57 |  | F2RL1 | UQCC2 |  |
| CLCA1 |  | SIRT2 | RPIA |  |
| SENP1 |  | FUCA1 | COL1A2 |  |
| ZFP709 |  | 1500009C09RIK | KCNJ9 |  |
| MRPS30 |  | CCDC39 | STAU1 |  |
| ARPP19 |  | OLAH | CES1C |  |
| F11R |  | SERPINA1C | GRIK5 |  |
| FAM227A | | MGARP | TAGLN2 |  |
| CLEC12A | | TMEM235 | GATB |  |
| AGMO |  | PHF1 | DAG1 |  |
| CECR2 |  | CHL1 | ZC3HC1 |  |
| CASR |  | MED10 | RETREG3 | |
| TRIM33 |  | BTBD18 | CETN2 |  |
| PTPRQ |  | GM765 | STOM |  |
| ZNHIT6 |  | BDNF | REEP1 |  |
| SLITRK5 |  | LHX1 | SNAP23 |  |
| MMP19 |  | F11 | SFR1 |  |
| EVI5L |  | 6720489N17RIK | ZMPSTE24 | |
| RNMT |  | TBX18 | IGH-3 |  |
| METTL6 |  | COMMD4 | NUP210 |  |
| TTC9 |  | MRPS15 | LMCD1 |  |
| IFIH1 |  | VAV2 |  |  |
| AADAT |  | VWC2 | PCDH19 |  |
| PES1 |  | ATP6V0B | UBAC2 |  |
| DHX36 |  | CTRL | ANO10 |  |
| RPS6KC1 | | LRRC49 | NUFIP2 |  |
| BTG1 |  | THBS3 | ILVBL |  |
| DDR2 |  | HTR2B | TATDN1 |  |
| WDR20 |  | BTF3 | FDXR |  |
| CDC42EP2 | | HIST1H4D | LARP7 |  |
| GM15440 | | GNAI2 | CASP3 |  |
| CMTM4 |  | WDR45 | NHLRC2 |  |
| ELP4 |  | E130309D02RIK | STX5 |  |
| 2810459M11RIK | | CCDC40 | NAT14 |  |
| FANCM |  | AI597479 | ARPC1B |  |
| RSRC2 |  | PRKG2 | CACNA1C | |
| TSC22D2 | | NFATC2 | ATP8A2 |  |
| LPHN3 |  | RASL2-9 | ABCF3 |  |
| ANKRD17 | | NUBPL | HLCS |  |
| AQP2 |  | ZFP764 | PPM1K |  |
| MRPL11 |  | RPL30 | SPEG |  |
| AI846148 |  | HIF1AN | CSTF2 |  |
| SOAT1 |  | AK6 | RABAC1 |  |
| GCC1 |  | ZNRF3 | PRKRA |  |
| DNAJB4 |  | LANCL1 | CHCHD2 |  |
| FASTKD1 | | ATXN1L | ISCA2 |  |
| RIT1 |  | SAAL1 | SQSTM1 |  |
| RPS12 |  | NANP | COQ8A |  |
| 5330417C22RIK | | KERA | EEPD1 |  |
| MAMDC2 | | IFI213 | COL6A1 |  |
| DHFR |  | 1810030O07RIK | WDR12 |  |
| BNIP2 |  | MPZL2 | FABP7 |  |
| RBM12B1 | | HAUS2 | WRNIP1 |  |
| AU021092 | | FAM184B | RIMS4 |  |
| TIMM17A | | PATJ | DENND5B | |
| KLF4 |  | GSTZ1 | GPRC5B |  |
| PLXNA4 |  | ALDH5A1 | TRAPPC2L | |
| FAM3A |  | APBA3 | MCRIP1 |  |
| EMC4 |  | NOTCH4 | LAMA5 |  |
| CYP2J9 |  | GMPR | SNX16 |  |
| PGM3 |  | TRIB2 | ALG5 |  |
| SUCNR1 |  | SLC25A10 | KCNAB1 |  |
| E030019B06RIK | | SERPINF1 | TXN2 |  |
| NAF1 |  | RABGAP1L | RPRD1B |  |
| GSTM7 |  | CPSF7 | TMED9 |  |
| UFM1 |  | DDX59 | MRPL41 |  |
| KRTAP6-5 | | KCNIP3 | ZNF207 |  |
| DNAJC1 |  | APOBEC1 | COMMD10 | |
| PMF1 |  | BAX | NLGN4L |  |
| NMT1 |  | MTMR12 | PHOSPHO1 | |
| ZFP207 |  | XCR1 | ASTN2 |  |
| DNAJC10 | | 4921504E06RIK | PLPPR2 |  |
| FBXL20 |  | PEBP1 | MSTO1 |  |
| TTPA |  | A730046J19RIK | CRYZL2 |  |
| UXS1 |  | RELL2 | TMEM200A | |
| A330021E22RIK | | MAB21L1 | ABL2 |  |
| PELI2 |  | PLEKHA4 | ITCH |  |
| PTPN21 |  | ACTA1 | SYMPK |  |
| LTC4S |  | HSF1 | PPP2R2D | |
| RTCA |  | ZIC3 | KIRREL3 | |
| APPL1 |  | STK4 | PNN |  |
| CACNA1B | | RDH5 | POMP |  |
| ELOVL7 |  | INIP | OSBPL3 |  |
| ZFP1 |  | WDR61 | RBM10 |  |
| CHID1 |  | RPL11 | PEAK1 |  |
| TMOD2 |  | TCP1 | CHMP1B1 | |
| COQ4 |  | PIGN | BCAP29 |  |
| RRP1B |  | SOX3 | FMC1 |  |
| CTNND1 |  | STX5A | RBMXL1 |  |
| PREB |  | SLC17A8 | TAB1 |  |
| MRPL39 |  | PLEKHG4 | LAMP5 |  |
| RBM17 |  | TBC1D5 | MTR |  |
| CTNND1 |  | PKN1 | ASPHD2 |  |
| ZFP866 |  | ZMAT3 | SEMA4B |  |
| ATP6V0E2 | | SLAIN1 | EMC3 |  |
| UBE2S |  | JAZF1 | GRM1 |  |
| RBMS2 |  | OLFR1426 | FYTTD1 |  |
| PLEKHF1 | | TRPV5 | MRPL23 |  |
| SEMA3E |  | TMEM71 | ASIC1 |  |
| VSIG10L |  | CAB39L | ANKRD46 | |
| EEF2K |  | PTPRE | BUB3 |  |
| FBXO4 |  | TNFAIP1 | PLEKHH2 | |
| CML2 |  | BBX | F11R |  |
| 3830408C21RIK | | TRPT1 | PPM1B |  |
| LAMC3 |  | TES | TBPL1 |  |
| LNP |  | OCRL | EML6 |  |
| DHX40 |  | TRMT9B | CR1L |  |
| IRF2BP2 |  | INPP5J | PPIP5K2 |  |
| KPNA6 |  | ZFP729B | SNX8 |  |
| NKD1 |  | THOC6 | SPRED1 |  |
| SERPINI1 | | S1PR5 | DCTN6 |  |
| GM16314 | | NAT8F6 | UBE2E1 |  |
| QSOX2 |  | CADPS2 | NUCKS1 |  |
| STEAP2 |  | BC029722 | RBM26 |  |
| CLEC14A | | LYL1 | UBL7 |  |
| AY358078 | | STOM | DAPK1 |  |
| PDE3B |  | PIP4P1 | MRPL28 |  |
| 1810030O07RIK | | TIRAP | SNRPF |  |
| CUL3 |  | ANGPT2 | EFNB2 |  |
| RGP1 |  | TRNAU1AP | PPP4R2 |  |
| FAM174A | | EYA1 | SERPINB8 | |
| MBNL3 |  | KMT2B | VPS37A |  |
| G2E3 |  | CNST | CPQ |  |
| SERPINC1 | | DNAJB4 | EXOSC7 |  |
| IKBKAP |  | KATNAL1 | CYGB |  |
| GPRASP2 | | FXN | UFL1 |  |
| TMC2 |  | TUBB4B | GPR162 |  |
| ANXA13 |  | HIST1H2BC | SLC22A17 | |
| RAB2B |  | PSMD14 | SRSF10 |  |
| THOC1 |  | CCDC103 | CCDC91 |  |
| AHCY |  | ENOPH1 | TUSC2 |  |
| CNNM2 |  | UBAP2L | NAV1 |  |
| POU2F2 |  | NRG4 | OSTC |  |
| NUP35 |  | ITSN2 | CPEB4 |  |
| UGGT2 |  | RASSF2 | HDGFL3 |  |
| NDUFA12 | | ACTR1A | TSPYL4 |  |
| CDK13 |  | PRKCZ | ATG4C |  |
| ZSCAN25 | | UXS1 | HDAC5 |  |
| LY96 |  | WEE1 | TMEM63B | |
| METTL21A | | TLCD1 | QTRT1 |  |
| MKLN1 |  | CCT7 | WDR77 |  |
| OCIAD1 |  | B3GNT7 | SLC39A12 | |
| GPR89 |  | EPHB3 | CNOT4 |  |
| PITPNA |  | UBA52 | CSMD1 |  |
| PACRGL |  | EDARADD | ZMIZ2 |  |
| PYROXD2 | | LHX4 | SUOX |  |
| PLLP |  | XRCC2 | ADCYAP1R1 | |
| 2700062C07RIK | | TMEM243 | AS3MT |  |
| PFKFB4 |  | GM21985 | KCNH7 |  |
| AZGP1 |  | SLCO1A5 | RPA3 |  |
| ULK4 |  | OLFR283 | ADCY8 |  |
| OSCP1 |  | GM43720 | MAN2A2 |  |
| CCDC13 |  | KLRI2 | NIPA1 |  |
| CSRP2 |  | GM11037 | CDIP1 |  |
| WIZ |  | GM49380 | EARS2 |  |
| CRK |  | KLK1B5 | ZDHHC17 | |
| PDE11A |  | OLFR57 | CXADR |  |
| VIL1 |  | TNNT3 | CTBP2 |  |
| PSMA2 |  | KRT75 | SDC4 |  |
| IARS |  | TLR5 | USP4 |  |
| ECHDC3 |  | VSTM2L | PDZD11 |  |
| CCDC137 | | GIGYF1 | SLC26A6 |  |
| YME1L1 |  | MCM2 | OGFR |  |
| WNT4 |  | ACBD5 | SHISA4 |  |
| ZFP286 |  | TSPAN7 | TBC1D22A | |
| LRRC14B | | NFYC | LRRK2 |  |
| RAB3GAP2 | | ZFP984 | SLC39A10 | |
| NEMF |  | SLC7A2 | TIA1 |  |
| NOL7 |  | MYO10 | CPEB2 |  |
| GALNTL6 | | ZDHHC21 | ASCC3 |  |
| POU2F3 |  | HEATR1 | UBE2G1 |  |
| CNTF |  | FAM53C | SMARCB1 | |
| PLXDC1 |  | BC031181 | JPH3 |  |
| JOSD2 |  | HIST2H2BE | TTC38 |  |
| FABP12 |  | ZIM1 | TTC3 |  |
| FBXW17 |  | TNFRSF8 | THG1L |  |
| SOD3 |  | DNAJA1 | LSM7 |  |
| METAP2 |  | GM13547 | PIR |  |
| ISOC2A |  | GM5819 | SLIT2 |  |
| LEKR1 |  | MRPL49 | GEMIN5 |  |
| ZFP318 |  | SDR9C7 | TMEM9B | |
| SIRPB1C |  | PRDM11 | ARID1A |  |
| LMTK3 |  | FAM3A | RAB26 |  |
| CLDN16 |  | STRIP1 | POLDIP2 | |
| NOS1 |  | KIF19A | PSMG2 |  |
| ARHGAP11A | | HIST1H4N | ADCY2 |  |
| ARFGAP2 | | CELSR1 | POLR2E |  |
| MOB4 |  | RASA2 | RER1 |  |
| ADRB3 |  | GM26992 | ZC3H4 |  |
| CASP16 |  | FMOD | LPP |  |
| ERCC6L |  | PSAT1 | CBX1 |  |
| TACO1 |  | CHMP7 | NDUFB2 |  |
| ARL4A |  | TNFRSF25 | PDS5A |  |
| FOXA1 |  | NRG3 | PBXIP1 |  |
| PLSCR3 |  | CIAO1 | PTRHD1 |  |
| LRTM1 |  | LY6E | TGOLN1 |  |
| RAD50 |  | PHF19 | MAP3K4 |  |
| PRPF38A | | PAFAH1B2 | DPF1 |  |
| UBR2 |  | SIGLEC1 | VPS36 |  |
| BC024978 | | CASP1 | CYTH1 |  |
| VPS13D |  | CDC42SE2 | MINDY3 |  |
| LRRC58 |  | ZC3HAV1 | RMND1 |  |
| INHA |  | CLDN14 | MTREX |  |
| C87436 |  | PRSS37 | DOPEY2 |  |
| ALG9 |  | NME3 | KLC4 |  |
| FIBIN |  | 1500011K16RIK | ATG16L1 | |
| POF1B |  | RAB30 | NUDT16L1 | |
| LRCH2 |  | CELF4 | ZNF326 |  |
| CYP2D37-PS | | CREB3L4 | ATG2A |  |
| SRGN |  | GFER | MMP17 |  |
| JMJD4 |  | ARNTL | PON2 |  |
| RAB11B |  | CLCA4A | PCYT1A |  |
| TAF10 |  | SGCA | EIF4E |  |
| ST3GAL2 | | GVIN1 | NUCB2 |  |
| DAGLB |  | PDE6B | VANGL2 |  |
| B3GALT6 | | CTSD | TRAPPC13 | |
| PCDHB22 | | PGM3 | C1QC |  |
| ABCC1 |  | CCDC3 | FASTKD2 | |
| CDA |  | PEX2 | SAMD4B |  |
| GPR116 |  | RTL4 | KAT8 |  |
| CALCOCO1 | | IK | GALNT16 | |
| HK2 |  | SLC19A1 | TRIM72 |  |
| CDC37 |  | NAPEPLD | DNAJB12 | |
| KIDINS220 | | COX7B2 | CDC34 |  |
| FBXL2 |  | ETS2 | CHRNA4 |  |
| PLIN3 |  | 2810004N23RIK | MLC1 |  |
| IDNK |  | LY6C1 | MRPL49 |  |
| FAM26E |  | LRP5 | RHOQ |  |
| MSANTD1 | | GM17660 | NCOA7 |  |
| PNPLA8 |  | GM12689 | CTAGE5 |  |
| SLC25A32 | | MOV10L1 | PXMP4 |  |
| NRG2 |  | GM10837 | EFNB3 |  |
| EFCAB9 |  | S100A3 | NEMF |  |
| ERBB2 |  | GPM6A | OXNAD1 |  |
| RGL3 |  | UGGT2 | MRPL1 |  |
| GINS1 |  | URAH | TM7SF2 |  |
| ITIH1 |  | FXR2 | PLEKHB1 | |
| ARID5A |  | BMPR1A | HSCB |  |
| GM15557 | | SLC22A23 | KCND3 |  |
| MBD2 |  | CPLANE1 | SEC11C |  |
| SLCO2A1 | | AKAP7 | LIG3 |  |
| 4932431P20RIK | | KLHL23 | NUP43 |  |
| CENPU |  | RIMKLB | JMY |  |
| EAPP |  | FANCG | CERS1 |  |
| AKIRIN2 |  | BZW1 | MAST1 |  |
| MAVS |  | DSC2 | TCEAL3 |  |
| RPS6KB2 | | MBIP | CMTM5 |  |
| LRRC66 |  | SCAMP1 | DNAJC3 |  |
| ZFP595 |  | BAG4 | LRRTM3 |  |
| LMF1 |  | ULK2 | SIRT3 |  |
| NUDCD1 |  | NPPC | MAST3 |  |
| CRP |  | PTMS | DCN |  |
| WDR86 |  | TRPV6 | FAM210A | |
| PLA2G2C | | HTATIP2 | ARHGEF38 | |
| EPHB2 |  | RPL6 | PLD2 |  |
| TMEM52B | | EYA4 | STAMBP |  |
| TMEM120A | | SPATA13 | PACS2 |  |
| MYL1 |  | BEX1 | NUBP1 |  |
| CD34 |  | CAML | GSTT3 |  |
| 6330419J24RIK | | SVOP | FAM162A | |
| UBALD1 |  | 2210016F16RIK | MTND2 |  |
| CLCNKB |  | DLEU7 | ICA1L |  |
| C030046E11RIK | | B9D1 | CDK5RAP3 | |
| TOX |  | CPNE8 | MRPL53 |  |
| SBF1 |  | PARP9 | PKN1 |  |
| LRRN3 |  | PKD2 | TOMM5 |  |
| FAM210A | | DCDC2C | THUMPD3 | |
| ZFP189 |  | PLD1 | RTTN |  |
| DUSP1 |  | MTHFD1 | CHKB |  |
| MRPL19 |  | ZBTB45 | GOSR1 |  |
| ZFP963 |  | MCAM | CLVS2 |  |
| AGPAT5 |  | PCOLCE2 | EVL |  |
| TRDMT1 | | CALCR | AJAP1 |  |
| COL5A1 |  | FNDC4 | AOC3 |  |
| CASC5 |  | IL10RA | H2AFZ |  |
| EDEM2 |  | VPS41 | EVI2A |  |
| C3AR1 |  | HDAC11 | IMPDH1 |  |
| XPO6 |  | CDKN1C | DYNLT1 |  |
| DUSP12 |  | LAPTM4B | PCBP3 |  |
| RNF13 |  | ATP5L | FOXP1 |  |
| RUNX3 |  | CNP | UFM1 |  |
| PLEKHG2 | | ASB3 | ATP6V0C | |
| COQ6 |  | ZFP54 | HIST1H3A | |
| OSGEP |  | SIAH3 | NUDT18 |  |
| TRIM27 |  | PLEKHO2 | OSTF1 |  |
| HELZ |  | BC005537 | BCL2L1 |  |
| RFTN2 |  | CCM2 | LARP4B |  |
| UBE2D2A | | PPM1L | MRPS10 |  |
| OLFR433 |  | FUBP3 | COMTD1 | |
| IL17D |  | UBA3 | FBXO44 |  |
| PRSS8 |  | COL4A1 | PODXL2 |  |
| DYRK1B |  | RNASEH1 | COG3 |  |
| SYNE4 |  | EIF4A1 | LZIC |  |
| KIF2A |  | GBF1 | VWA1 |  |
| SOS1 |  | LRIG3 | FAM20B |  |
| SEL1L |  | MBD6 | GHITM |  |
| MBL1 |  | GM3739 | CSTF3 |  |
| SLC25A51 | | KCNH5 | NHP2 |  |
| TRMT2A |  | OLFR77 | FXYD6 |  |
| MRPS26 |  | GM10053 | |  |
| NOTUM |  | B4GALT6 | HACD2 |  |
| 3632451O06RIK | | CDKL1 | RABL3 |  |
| ACBD3 |  | WFDC3 |  |  |
| CCDC125 | | SYNJ1 | HM13 |  |
| MPLKIP |  | IKBIP | FUNDC1 |  |
| PLIN2 |  | HIPK4 | SSR3 |  |
| NPAT |  | SMG9 | TMEM47 |  |
| CHD7 |  | TCN2 | SPPL2B |  |
| FBXO7 |  | TAP2 | HIST1H3B | |
| PLEKHO1 | | KANSL3 | EEFSEC |  |
| RAB20 |  | TNFRSF22 | SEC24A |  |
| ISY1 |  | RNF208 | RPS27L |  |
| FAM35A |  | ABL1 |  |  |
| ZFP944 |  | AK3 | HIST2H2AA1 | |
| PABPC4L | | LSP1 | PBDC1 |  |
| FBXL17 |  | SLK | ANKRD17 | |
| SGCA |  | ACP2 | NCKAP1L | |
| MS4A8A |  | TPI1 | SERPINH1 | |
| ALAD |  | PDZPH1 | ZNHIT6 |  |
| CYLD |  | GM4737 | PLCH1 |  |
| PRR16 |  | RAB8B | SDHAF1 |  |
| POC1A |  | S100A6 | PDLIM1 |  |
| LRRC75A | | BNIP2 | PRDX4 |  |
| IL12RB1 |  | PPIL1 | HMGN1 |  |
| DNAH8 |  | FRMD3 | CHD7 |  |
| SLC39A5 |  | LTK | TMA7 |  |
| CD36 |  | CMIP | SPOCK1 |  |
| MROH2A | | PAOX | MID1IP1 |  |
| TRIM7 |  | USF3 | PPM1J |  |
| HOXA3 |  | MTERF4 | ZNF365 |  |
| DMRT2 |  | SOX12 | FTSJ3 |  |
| KLHL23 |  | A730061H03RIK | ARF2 |  |
| TRMT13 |  | ANKHD1 | OSBPL11 | |
| RIMS3 |  | DAP3 | MRPS17 |  |
| GM14326 | | PARPBP | TPM2 |  |
| CFL2 |  | STK35 | PLXDC2 |  |
| BCL6 |  | WDR89 | RXRB |  |
| 3830406C13RIK | | COL13A1 | BIN2 |  |
| NDNL2 |  | GM2000 | GARNL3 |  |
| SFTPA1 |  | POU5F2 | ADGRG1 |  |
| SHISA6 |  | BIN3 | RBP1 |  |
| GCGR |  | GPRASP2 | ZDHHC14 | |
| TMEM199 | | TRMT10C | TLR13 |  |
| CAR5B |  | RDH14 | SERAC1 |  |
| TMEM60 |  | TMEM121B | ASXL3 |  |
| HIST1H4J | | SELENOT | CCDC66 |  |
| EXOC7 |  | RPP21 | SCFD2 |  |
| NDUFB5 |  | B9D2 | POFUT2 |  |
| IL23R |  | XPO4 | RAB8B |  |
| EDARADD | | EMILIN2 | CISD3 |  |
| R3HDM2 |  | ACSF2 | COX7B |  |
| NAPA |  | SOX15 | RBM44 |  |
| OASL2 |  | ERBIN | SZT2 |  |
| 9430016H08RIK | | KCNU1 | CEP152 |  |
| CDK7 |  | NHLH2 | RSRC2 |  |
| MAMSTR | | RPAP1 | GRIK3 |  |
| RHOBTB2 | | ZDHHC5 | POLR2J |  |
| POLR3C |  | HCFC1R1 | SRSF5 |  |
| SLC15A5 |  | SPTA1 | FXN |  |
| UAP1 |  | MSX2 | DAPK3 |  |
| MKX |  | CENPT | AKAP2 |  |
| ZEB2 |  | TMEM68 | SAP18 |  |
| POPDC3 |  | ZKSCAN4 | NINJ1 |  |
| ABCF3 |  | ADNP | NPTX2 |  |
| ORAI1 |  | SLC10A7 | CTSS |  |
| ANK1 |  | 3110082I17RIK | ADAM15 |  |
| 2410131K14RIK | | TMC8 | ITM2B |  |
| MMP16 |  | LMBRD2 | ITGAM |  |
| TERF2IP |  | RAB3D | S100A4 |  |
| MAT2A |  | UPF3A | LYNX1 |  |
| TNFSF13B | | SNCAIP | RPS6KA1 | |
| SLC51A |  | PLTP | GABRA2 |  |
| AGTRAP |  | EIF4B | GABRA3 |  |
| RASSF4 |  | SCNN1G | FLT1 |  |
| SHROOM1 | | ASAH1 | TTL |  |
| 43526 |  | GAL3ST1 | MSH2 |  |
| SAP18 |  | KLF10 | SULT1A1 | |
| A430033K04RIK | | COL18A1 | RAD23A |  |
| CCDC71L | | SEC62 | NUB1 |  |
| UBE2T |  | MIEF1 | TYRO3 |  |
| HSD17B6 | | PDE6D | PCBP4 |  |
| PRSS12 |  | CAR4 | MRPS21 |  |
| GBP5 |  | UAP1 | MRPS6 |  |
| GNPTG |  | FBXL12 | B3GAT3 |  |
| DDX25 |  | NOP58 | GMEB2 |  |
| EIF3E |  | ZFX | DEPDC5 |  |
| BDH1 |  | ITPR3 | KCNJ3 |  |
| HAO2 |  | PTK2B | SMAD1 |  |
| SBK2 |  | ARHGEF10L | BID |  |
| PIGV |  | ZNFX1 | NXN |  |
| CCDC18 |  | LONRF3 | DAB2 |  |
| DEPDC5 |  | FAM193B | CREB1 |  |
| CAPZB |  | MRM2 | SLC7A1 |  |
| SLC30A5 |  | CCDC30 | DACT3 |  |
| CTTNBP2NL | | ANKRA2 | TANC1 |  |
| CNOT11 |  | ZFP26 | CPNE9 |  |
| VTA1 |  | TTPAL | PNPLA6 |  |
| 1700003E16RIK | | EZR | PIP4P1 |  |
| EPC2 |  | PLPPR5 | PAG1 |  |
| LAS1L |  | KRT12 | CTU2 |  |
| BRD8 |  | SRP68 | GAREM1 |  |
| ST13 |  | VMN1R90 | LYRM9 |  |
| DNAJB14 | | KCNIP1 | SVIP |  |
| LTBP1 |  | NUDT21 | BBS1 |  |
| LOH12CR1 | | RASSF3 | SNX25 |  |
| CHCHD5 |  | GM21698 | WDR18 |  |
| ALKBH3 |  | LTC4S | TSHZ1 |  |
| FOXK2 |  | UMAD1 | UNKL |  |
| PRICKLE1 | | FBXO43 | WDR81 |  |
| BCKDK |  | NRG2 | WDR34 |  |
| NOSTRIN | | HNRNPAB | DPF2 |  |
| ATP1A2 |  | FCRL6 | COX7A2L | |
| TTC21B |  | KCNH1 | PDCD4 |  |
| SHOC2 |  | TLL1 | ITGA3 |  |
| ZFP574 |  | KLHL8 | RAB3IP |  |
| CEP72 |  | DNAJC8 | KRT73 |  |
| MANBAL | | CDH3 | ZC3H11A | |
| CPXM2 |  | SCUBE1 | PPP4R3A | |
| AA415398 | | PYCARD | NUP188 |  |
| MYO5B |  | SERPINA3C | VMA21 |  |
| YAF2 |  | CFHR2 | SUGCT |  |
| RAB29 |  | LIPN | FAM19A2 | |
| 9130019O22RIK | | VMN1R34 | PRRC2B |  |
| KTN1 |  | OLFR685 | DNPH1 |  |
| TSGA10 |  | GM364 | RIC8B |  |
| MRPL48 |  | TNP1 | TTC9C |  |
| PTRH2 |  | GM15448 | NECAB1 |  |
| PFKL |  | HOXD1 | COA6 |  |
| CEBPZOS | | CGRRF1 | GOPC |  |
| ASB3 |  | RPLP0 | ELOVL5 |  |
| NHP2L1 |  | PLPPR2 | SCN3B |  |
| ESD |  | FAM216A | B3GLCT |  |
| ZFP933 |  | PPP1R3F | ITIH5 |  |
| DDX4 |  | A230050P20RIK | SMARCAL1 | |
| MESDC1 |  | LGALS3 | TSPAN9 |  |
| BST1 |  | CCNY | DOPEY1 |  |
| 4930529M08RIK | | B430218F22RIK | LYPD1 |  |
| CYP2D10 | | CATSPERB | TMEM87A | |
| HEATR2 |  | ADIPOQ | ITPK1 |  |
| MTMR12 | | SLAMF8 | WDR59 |  |
| ZFP276 |  | XXYLT1 | SH3RF3 |  |
| GPC4 |  | NAA16 | MMAA |  |
| ZKSCAN4 | | TEKT1 | STRIP2 |  |
| RPGRIP1L | | COG1 | YOD1 |  |
| STAM |  | LZTS3 | TTC19 |  |
| LTV1 |  | CCR3 | BOLA3 |  |
| TSPAN1 |  | CDC42EP5 | MTURN |  |
| SNF8 |  | MORF4L1 | RGS12 |  |
| MBL2 |  | TRIM29 | SLC24A4 |  |
| RAB8A |  | VPS11 | DNER |  |
| SLC4A3 |  | EEFSEC | KCTD15 |  |
| D1ERTD622E | | E230025N22RIK | CNOT3 |  |
| MAP2K3 |  | FRS2 | PPP1R11 |  |
| MYG1 |  | WDTC1 | MYRIP |  |
| NEIL2 |  | CC2D2A | OPTN |  |
| SNX7 |  | NFYB | SLC36A1 |  |
| SRL |  | GM4969 | ABHD11 |  |
| UTP23 |  | KLHL35 |  |  |
| CTSC |  | TTF1 | HS2ST1 |  |
| MTHFSL |  | CACNA1S | MED25 |  |
| SRPX |  | STAT4 | GPN1 |  |
| ACSM1 |  | NELL2 | ADPGK |  |
| POLD4 |  | OLFR456 | RPA1 |  |
| CRISPLD1 | | ASAH2 | IMPA2 |  |
| ETNPPL |  | HDGFL3 | CAMK1G | |
| DIRC2 |  | KIF21A | TDRD3 |  |
| NDUFS4 |  | POLR1E | GORASP1 | |
| TCEANC2 | | GM13889 | ERLIN1 |  |
| OXR1 |  | KDF1 | UCKL1 |  |
| GPR108 |  | MIA2 | RNF185 |  |
| PRPF31 |  | GNG8 | GLRX2 |  |
| BECN1 |  | CCDC89 | XPO6 |  |
| BTLA |  | ALDH1L2 | ELP5 |  |
| PRR15 |  | EAF2 | NENF |  |
| TGFB1 |  | GLTP | SAR1B |  |
| PHYKPL |  | PTP4A1 | RGS10 |  |
| PDGFC |  | 9030612E09RIK | MRPL57 |  |
| SMIM5 |  | MCOLN1 | GOLT1B |  |
| SORCS2 |  | ATP6V1C2 | BBS2 |  |
| ACOT12 |  | SNAPIN | RBM8A |  |
| LGALS3BP | | CELSR2 | GTPBP8 |  |
| ZFP846 |  | SPIRE1 | MMS19 |  |
| TRIM62 |  | TBC1D1 | LAMTOR5 | |
| NAMPT |  | LAMC1 | PLGRKT |  |
| SNX33 |  | ECT2 | POLR3C |  |
| TCP11L2 |  | UNC13A | PPIL3 |  |
| D17WSU92E | | NAV2 | NOSIP |  |
| AREL1 |  | SPTLC1 | TRIR |  |
| DSTN |  | MAPK1IP1L | COX20 |  |
| USH1C |  | ATG14 | POLR2D |  |
| ZFP281 |  | SMN1 | SLC48A1 |  |
| NXPE3 |  | PIP5K1C | TMEM126A | |
| ACTR10 |  | GAPDHS | DDA1 |  |
| FBRS |  | SKA2 | PELP1 |  |
| TNIP2 |  | RPA2 | ABHD5 |  |
| 1110004E09RIK | | FAM114A2 | POLD3 |  |
| ASRGL1 |  | CPLX1 | CHRM3 |  |
| DOK2 |  | ARL1 | CLDN12 |  |
| EXTL1 |  | UCP2 | RCAN2 |  |
| COX20 |  | ARHGEF15 | GLMP |  |
| WDR12 |  | MGRN1 | NQO2 |  |
| LENEP |  | FBXL7 | SPHK2 |  |
| TRUB2 |  | ADAM15 | NRIP3 |  |
| DUSP19 |  | HAO2 | AP4M1 |  |
| 43530 |  | CLASP2 | ELOVL1 |  |
| TLR8 |  | ABCC6 | NPHP1 |  |
| MCCC1 |  | GUCY1A2 | PNCK |  |
| ANKRD13A | | GSS | CA5B |  |
| PLEC |  | NPEPPS | TIMM23 |  |
| TRP53BP1 | | MMS22L | SLC25A15 | |
| MTERFD2 | | EIF3J1 | SNCG |  |
| TXNL4A |  | POLM | TPBG |  |
| KNSTRN |  | RPN2 | TIMM17B | |
| DBF4 |  | WDFY2 | MOCS2 |  |
| VAMP5 |  | PGRMC2 | SNAPIN |  |
| ZDHHC3 |  | NBN | MTM1 |  |
| CFH |  | MRPL27 | SLC7A4 |  |
| CCDC58 |  | AU021092 | STARD5 |  |
| SNX4 |  | TSPAN1 | MAST4 |  |
| TRDN |  | U2AF1 | EVI5 |  |
| MPP6 |  | PPIL4 | KYAT1 |  |
| GUCY2G |  | DSCC1 | FN1 |  |
| AP2B1 |  | GPR143 | YBX1 |  |
| KIF18A |  | GYPA | CD34 |  |
| DCLRE1B | | KEL | MSL3 |  |
| SIKE1 |  | DPEP2NB | DAB1 |  |
| ARHGAP1 | | NEIL3 | SSTR2 |  |
| GM14327 | | HEMGN | LHPP |  |
| ASIC5 |  | GM8122 | SPRY2 |  |
| SFTPB |  | OLFR1318 | TRIM33 |  |
| ADAM15 |  | OLFR1362 | VKORC1 |  |
| ZFP955A |  | UPK3A | EXTL2 |  |
| NAGS |  | FCNB | FEZ1 |  |
| NOSIP |  | DEFB47 | MRPS11 |  |
| TMEM261 | | GM648 | SLC20A2 |  |
| HMGB2 |  | CAR6 | H2-Q10 |  |
| SIAH1A |  | SSBP3 | CAMTA2 |  |
| RALB |  | STK38 |  |  |
| CCL25 |  | KANSL1L | GPR89A |  |
| RBM12B2 | | WIPF2 | MTA3 |  |
| TMEM27 |  | WEE2 | CBFB |  |
| GLOD4 |  | GNPAT | DHX57 |  |
| LZTR1 |  | PDE8B | SLC5A6 |  |
| MTPAP |  | SALL3 | TTC17 |  |
| MPZL2 |  | KLHL14 | PLIN3 |  |
| SLC5A8 |  | DCPS | DPY19L1 | |
| IKBKE |  | COL4A3 | ECHDC2 |  |
| SNX25 |  | TIMM21 | PEX6 |  |
| APP |  | YEATS2 | KIAA1211 | |
| ATP1A4 |  | CIRBP | YIPF5 |  |
| GTF3C6 |  | YJEFN3 | KRT6A |  |
| DUSP23 |  | MAP3K2 | SLC22A6 |  |
| ATP8A2 |  | EWSR1 | DGUOK |  |
| IGSF10 |  | MOB3A | TSTA3 |  |
| TRMU |  | GM45623 | SLC25A40 | |
| ATP9A |  | 1700017B05RIK | PPP1R3G | |
| PODNL1 |  | RPL37 | HOPX |  |
| CCDC80 |  | AARSD1 | KIF3C |  |
| UCP2 |  | POLA2 | PIK3CD |  |
| RHOH |  | GM3636 | MTCO1 |  |
| COL1A2 |  | AMH | MSH6 |  |
| PAFAH1B3 | | TCEAL8 | SEPHS2 |  |
| MDH2 |  | DNAJC5B | NR4A2 |  |
| BC021785 | | C030006K11RIK | DNAJB14 | |
| TSKU |  | SLC35A2 | SCN4B |  |
| DFFA |  | CALB1 | NRN1 |  |
| TTC39A |  | HTT | STAU2 |  |
| GABBR1 |  | DAO | CORO6 |  |
| MIOS |  | DOPEY1 | GFM2 |  |
| CDC14B |  | RASA3 | CUEDC2 |  |
| FMO4 |  | DUSP15 | DCP1A |  |
| SPC25 |  | NAALADL2 | SEZ6L |  |
| MTUS1 |  | CYTH4 | LRTM2 |  |
| GM9887 |  | GNB1 | CPSF4 |  |
| SLC30A9 |  | SLC4A8 | SLC14A1 |  |
| BRCA2 |  | D17H6S53E | RNASEK |  |
| ZFP68 |  | CAMKK1 | TSC22D3 | |
| HIST1H4I | | GM8186 | CCNF |  |
| ZFP619 |  | RAC3 | PLA2G15 | |
| TGM2 |  | TRP53BP2 | MEF2C |  |
| BLOC1S5 | | USP36 | LPCAT1 |  |
| TSPAN14 | | SPRTN | ALG11 |  |
| SERBP1 |  | LYRM9 | CPLX3 |  |
| PPP1R14A | | KBTBD2 | KCNC3 |  |
| ATG2B |  | LRRIQ1 | MRPL45 |  |
| CASP12 |  | PNP2 | MKRN2 |  |
| ZW10 |  | PABPC4L | BLOC1S1 | |
| PTBP2 |  | PHYH | CCT6B |  |
| GIN1 |  | ZIC1 | RAB28 |  |
| 1700007B14RIK | | RBMX | NCOA5 |  |
| UBE2B |  | DAPP1 | GPC5 |  |
| ARL6IP6 |  | GM9938 | YIPF4 |  |
| TMEM41B | | VAMP4 | FADS2 |  |
| RBM33 |  | TTYH3 | SMG6 |  |
| LYRM9 |  | TPRGL | GALNS |  |
| IRAK3 |  | 1700066M21RIK | CTSH |  |
| CPN1 |  | PCDHA6 | MXRA7 |  |
| 43533 |  | ENDOU | GPAM |  |
| MESDC2 |  | TRIM14 | CPTP |  |
| TIMM22 |  | NBEAL1 | SYNM |  |
| KLHDC2 |  | MPP1 | EIF6 |  |
| POLR3G |  | BNIP3L | MED11 |  |
| HIP1R |  | ARID4A | PTPN13 |  |
| BC025446 | | EPN3 | COPS9 |  |
| S1PR2 |  | LOR | KRT2 |  |
| MMP17 |  | PDGFD | SPCS1 |  |
| ACYP2 |  | TCHH | RNF7 |  |
| NSUN6 |  | GUSB | NTNG2 |  |
| FBXO36 |  | XPNPEP3 | ATP6V1G3 | |
| SH3TC1 |  | CYHR1 | SERPINF2 | |
| MRPL17 |  | MRPS24 | NCOR1 |  |
| ANKRD45 | | XPO1 | SYVN1 |  |
| FYN |  | GM973 | TBK1 |  |
| MYO5A |  | PRR15 | ZNF330 |  |
| COX16 |  | HELLS | LRRN1 |  |
| MYO6 |  | HMCES | TRMT112 | |
| EIF2S3Y |  | BRD7 | LAMTOR3 | |
| TXNDC9 |  | TRIM34A | GALK1 |  |
| HSF2 |  | KDM2B | TAF7 |  |
| NCKIPSD | | SNX29 | SLC29A2 |  |
| ATF6B |  | PCYOX1 | PCIF1 |  |
| TRIM26 |  | SLC13A5 | PGM5 |  |
| CYP2B9 |  | GLS2 | KCNAB3 |  |
| TSPAN11 | | FAT3 | SNRK |  |
| ST14 |  | CNN2 | EMC6 |  |
| CANT1 |  | NCF4 | OPALIN |  |
| ANLN |  | SCUBE2 | DENND6B | |
| KLHL20 |  | ABCA4 | SCOC |  |
| ERP44 |  | MTMR6 | ARL2BP |  |
| PINK1 |  | ARNTL2 | MOB2 |  |
| CALCRL |  | CCR5 | LRFN1 |  |
| GRB14 |  | RBBP5 | DUSP14 |  |
| ZFML |  | 6430628N08RIK | RNF123 |  |
| CEP250 |  | CNGA3 | PTPRR |  |
| TAPT1 |  | LPCAT3 | DOLPP1 |  |
| WEE1 |  | ACTR3B | RUSC1 |  |
| AEBP2 |  | ABCC9 | SMIM8 |  |
| KCNQ1 |  | BOD1 | LBH |  |
| PPP1R12A | | PIK3CA | UFSP1 |  |
| RPAP3 |  | ZFP760 | PEX19 |  |
| SNAP47 |  | CHTOP | INPP5K |  |
| JAK1 |  | CDH26 | PTGFRN |  |
| 1700011H14RIK | | OLFR1385 | NLK |  |
| IL18R1 |  | CYP2C55 | MAIP1 |  |
| SF3B5 |  | TESCL | HDAC4 |  |
| CYP4F15 |  | MOCS3 | PPARG |  |
| THAP3 |  | MRPS33 | EEF1AKMT1 | |
| TTLL12 |  | VMN1R188 | ZNRF1 |  |
| PSMG4 |  | BICDL2 | STK33 |  |
| CDCA7L |  | FERMT3 | APOD |  |
| NEXN |  | ATP13A5 | DEGS1 |  |
| F11 |  | CDKN2AIP | ENPEP |  |
| FAM169A | | AGTRAP | CNIH4 |  |
| CYP4A12A | | AFF2 | TMX1 |  |
| KTI12 |  | RBX1 | TMED8 |  |
| TRMT5 |  | ADGRV1 | POT1 |  |
| MAPKAPK5 | | TAS2R108 | PCBD1 |  |
| EID1 |  | SP9 | XDH |  |
| DNAJC15 | | ZFP715 | GAR1 |  |
| MPI |  | SLC25A23 | PPP4R4 |  |
| STAU1 |  | DBP | DLG5 |  |
| TRP53RK | | NIPBL | EVA1A |  |
| TRADD |  | OPTN | GAB2 |  |
| XPO7 |  | MINDY3 | RAB27A |  |
| SCNN1B |  | SYT9 | CLCN4 |  |
| APOPT1 |  | A4GALT | PSMF1 |  |
| MARS2 |  | TPRN | FXYD7 |  |
| CNTN2 |  | CCDC7B | CSMD3 |  |
| CLCN6 |  | NCALD | G0S2 |  |
| AKR1B10 | | COMMD6 | OGFOD1 |  |
| SWT1 |  | EIF6 | ULK3 |  |
| GPR149 |  | GPALPP1 | KCNJ11 |  |
| GNG3 |  | PRIM2 | MRPL51 |  |
| SF3A1 |  | TLR6 | RABGGTB | |
| MBIP |  | CDPF1 | ZYG11B |  |
| HNRNPUL2 | | DSN1 | MINPP1 |  |
| EPHX1 |  | APLP2 | CYTH3 |  |
| PRX |  | MC4R | NAB2 |  |
| GCLM |  | ARMC7 | MUL1 |  |
| KCNG2 |  | TECPR2 | SLC35A2 |  |
| ACOT1 |  | TRIM33 | COA3 |  |
| FXN |  | TNFRSF9 | MAPK8IP2 | |
| MFSD8 |  | WNT7B | FHIT |  |
| NAPEPLD | | ZFP68 | MFGE8 |  |
| SKP1A |  | DNAJB6 | NUP58 |  |
| DARS |  | FKBP2 | OXLD1 |  |
| NUDT19 |  | TNPO1 | ZDHHC8 |  |
| NAT1 |  | DAZL | INTS5 |  |
| 2610528J11RIK | | GAS2L3 | CRHBP |  |
| MALSU1 |  | NEUROD1 | AASDHPPT | |
| NME1 |  | PANX2 | SRRM1 |  |
| BC089597 | | MRPL40 | NTSR2 |  |
| TIMM21 |  | NSUN7 | GALNT2 |  |
| PIK3R6 |  | CD160 | MRPL21 |  |
| PIK3R2 |  | EDN3 | PTK7 |  |
| TSPAN13 | | VPS39 | ANKRD27 | |
| EIF2B3 |  | FAM193A | KCTD10 |  |
| EIF2B5 |  | DOPEY2 | MKLN1 |  |
| NUFIP1 |  | HSD17B10 | HCK |  |
| PDDC1 |  | MBLAC1 | BOD1L |  |
| MYOZ3 |  | SCP2D1 | PGS1 |  |
| CYP4F39 |  | 4933406M09RIK | FERMT3 |  |
| TCEB3 |  | GM49320 | ZNF385A |  |
| KLK13 |  | ZFP940 | CEPT1 |  |
| SDCCAG8 | | TREH | ARSG |  |
| ICT1 |  | RSPRY1 | SHPK |  |
| AKAP6 |  | NUDT7 | LRRC8B |  |
| OPTN |  | HYAL2 | RHPN2 |  |
| SLC52A2 |  | OST4 | LRRC20 |  |
| IFI35 |  | XPC | SDSL |  |
| APCS |  | SLC25A14 | CYP1A2 |  |
| SLC8A1 |  | RNPEP | CR2 |  |
| ANAPC10 | | WWP2 | INTS1 |  |
| RAI1 |  | KLF12 | ASH2L |  |
| FAM83H |  | ZFP280C | NXF1 |  |
| RPS15A |  | STEAP2 | KANK3 |  |
| CCDC14 |  | CXXC5 | SLC38A7 |  |
| CPN2 |  | B630019K06RIK | CNPY4 |  |
| SCEL |  | CTR9 | TRAPPC2 | |
| SUGT1 |  | TGM2 | METTL13 | |
| S1PR1 |  | TRIQK | SHD |  |
| ANKHD1 |  | MYO18B | C1QTNF4 | |
| FILIP1L |  | TMTC4 | WDR4 |  |
| CGGBP1 |  | NCK1 | ELP4 |  |
| POLR3K |  | COX7A2L | MEGF9 |  |
| FTCD |  | TMEM205 | TACC2 |  |
| 2610001J05RIK | | PTPRN | P4HA1 |  |
| AGK |  | HSPA8 | SLC25A14 | |
| TSN |  | GM32717 | |  |
| PRDX2 |  | KCNE1 | ABHD17A | |
| ASCC3 |  | KRTAP2-4 | CD55 |  |
| GM14306 | | NPW | GLMN |  |
| RPL21 |  | 14-Sep | ANO3 |  |
| GGT7 |  | CEACAM15 | ELAC1 |  |
| STAB2 |  | OLFR624 |  |  |
| RNF40 |  | 1700013H16RIK | EFEMP2 |  |
| ZMIZ2 |  | OLFR329-PS | RABEPK |  |
| MRPL57 |  | TRIM10 | ITGA1 |  |
| GRAMD3 | | ATL3 | APOC1 |  |
| DERL2 |  | SPAG9 | B3GALT6 | |
| EIF3F |  | GABRA3 | RFX3 |  |
| ZDHHC2 |  | FGFR2 | CDS1 |  |
| ABCC5 |  | RPRM | ITGA6 |  |
| C2CD2L |  | CNOT9 | GBA |  |
| SAP30BP | | SAMD11 |  |  |
| RFNG |  | H2-OB |  |  |
| CRCP |  | AQP11 |  |  |
| BAG5 |  | HAT1 | SATB1 |  |
| TRAPPC2L | | AASDHPPT | TYW5 |  |
| MTMR7 |  | TFAP2C | LRRTM2 |  |
| NDUFB11 | | AP3B1 | NQO1 |  |
| TAGLN2 |  | ZC2HC1A | SH3BP1 |  |
| PECR |  | MOB1B | TOMM7 |  |
| IKBIP |  | SIGLECE | MSANTD4 | |
| RNF41 |  | TEKT3 | CEACAM20 | |
| SERPINF1 | | GM3488 | BET1 |  |
| NUPR1L |  | TRIM25 | DEPTOR |  |
| IL15RA |  | ZDHHC1 | SCPEP1 |  |
| ERICH5 |  | LYRM2 | VASP |  |
| FXR1 |  | RFTN1 | USP39 |  |
| SKIDA1 |  | RTN4 | MAVS |  |
| ACADSB |  | DDX54 | SUN2 |  |
| ITPKC |  | GPD1L | TSPAN5 |  |
| TSC22D4 | | DNAJA3 | ARL5A |  |
| TMEM161A | | KREMEN2 | TMEM223 | |
| DOCK1 |  | USH1C | MRPL55 |  |
| PHF8 |  | GGH | HASPIN |  |
| GPR39 |  | GALNT3 | CLEC16A | |
| ZFP639 |  | OS9 | ASPH |  |
| LAMP2 |  | FAM173B | BZW2 |  |
| STOML1 |  | FAM19A3 | POLR2C |  |
| DNAJC22 | | TRAF6 | RGS4 |  |
| GM10762 | | EIF5B | ODF2 |  |
| TTLL6 |  | BRK1 | TERF2 |  |
| RNF146 |  | IL1R1 | SCAMP4 |  |
| RPS19 |  | FV1 | NT5C3B |  |
| SEC62 |  | SLCO1C1 | FAM126A | |
| RBP1 |  | VSIR |  |  |
| F2 |  | TMEM240 | STAMBPL1 | |
| DNAAF2 |  | RNF215 | CDH8 |  |
| DMAP1 |  | CENPQ | SH3PXD2A | |
| GPBP1 |  | ZFP444 | ETNK1 |  |
| LCAT |  | RSF1 | BSCL2 |  |
| HGSNAT |  | MS4A6B | NDUFAF5 | |
| RABL3 |  | AIFM1 | PTGS1 |  |
| POLE |  | EIF2B1 | SEC61A1 |  |
| COL14A1 | | GM10801 | TXNL4A |  |
| MRPS25 |  | HPGDS | MYT1L |  |
| KCNK3 |  | LGI3 | YY1 |  |
| NARF |  | ZFP790 | XKR4 |  |
| MDP1 |  | GM9774 | OVGP1 |  |
| E030030I06RIK | | DCP2 | NYAP1 |  |
| P4HA2 |  | NUDT1 | CCDC90B | |
| 0610040J01RIK | | OLFR1033 | ALKBH6 |  |
| CLPTM1 |  | CHCHD5 | ABHD4 |  |
| C1QL2 |  | SLC39A1 | RNF167 |  |
| LSM4 |  | PEX5 | BCL7B |  |
| SLC16A3 |  | PTS | CSTF1 |  |
| SH3D19 |  | FAM220A | RAD54L2 | |
| ROBO1 |  | TBATA | GTF2A1 |  |
| REM1 |  | CBX3 | ATP23 |  |
| ASTN2 |  | FOXO1 | RPL22L1 |  |
| MACC1 |  | ATP6V1B1 | LIPA |  |
| SART3 |  | ESRRB | KCTD3 |  |
| NPY2R |  | HDC | UCMA |  |
| HCAR1 |  | GM19426 | TMEM209 | |
| UQCRFS1 | | DMTN |  |  |
| FAM193A | | FAM98A |  |  |
| AMY2A3 |  | FAT2 |  |  |
| RTTN |  | ELOA |  |  |
| MUP21 |  | GDNF |  |  |
| KCNK10 |  | TCF20 |  |  |
| DDRGK1 |  | SPHK2 |  |  |
| SDPR |  | RNF19A |  |  |
| ANGPTL3 | | GALM |  |  |
| GM2026 |  | PI4K2A |  |  |
| BOLA2 |  | PNP |  |  |
| FAM161A | | SLC30A3 |  |  |
| CES3A |  | OLFR113 |  |  |
| ACAT1 |  | SRP54C |  |  |
| CNPY2 |  | RAB42 |  |  |
| FAHD1 |  | MRPL2 |  |  |
| TRAFD1 |  | AFAP1L2 | |  |
| PDZD8 |  | ACOT4 |  |  |
| AMY2A4 |  | RBM43 |  |  |
| ELOF1 |  | PROSER3 | |  |
| KRT7 |  | SDHA |  |  |
| DTNBP1 |  | CDC34 |  |  |
| FAM114A1 | | VPS13D |  |  |
| PCK2 |  | ACE2 |  |  |
| NEK6 |  | IL10RB |  |  |
| N4BP3 |  | AMPD2 |  |  |
| ACOX3 |  | GDPD5 |  |  |
| AMY2A2 |  | LIMS2 |  |  |
| TEN1 |  | BARHL1 |  |  |
| ZFP292 |  | ITGB5 |  |  |
| ARPC3 |  | EIF2D |  |  |
| IFT88 |  | MFAP4 |  |  |
| RHNO1 |  | DUOX1 |  |  |
| SCRG1 |  | GM5741 |  |  |
| MFAP5 |  | TMEM140 | |  |
| CNPPD1 |  | HTR1D |  |  |
| TMEM45B | | CCBE1 |  |  |
| EMC10 |  | HIST1H2AE | |  |
| GM14420 | | XLR4B |  |  |
| MTMR2 |  | RS1 |  |  |
| MT1 |  | HASPIN |  |  |
| FBXO3 |  | SERPIND1 | |  |
| AFM |  | DNAJC5 |  |  |
| BORA |  | RASAL2 |  |  |
| SLC22A22 | | IP6K1 |  |  |
| CLEC4D |  | GLI3 |  |  |
| LY6A |  | TWSG1 |  |  |
| IZUMO4 |  | ABCG1 |  |  |
| DPPA1 |  | FAM83G |  |  |
| TMEM9B | | CCDC88C | |  |
| LHX5 |  | PTPN1 |  |  |
| MYH11 |  | ZFP873 |  |  |
| TOP1MT |  | EIF4H |  |  |
| NAPSA |  | PCDHGA3 | |  |
| ANKRD40 | | TPD52L2 | |  |
| EXOSC10 | | IFIT3B |  |  |
| FKBP9 |  | LCP1 |  |  |
| UNC5CL |  | MLF2 |  |  |
| EEF1A2 |  | GRSF1 |  |  |
| AP1G2 |  | CARF |  |  |
| HMGCR |  | D430019H16RIK | |  |
| MAP4K4 |  | P3H2 |  |  |
| PIGC |  | RHBDD3 |  |  |
| CDO1 |  | GM42688 | |  |
| CCL6 |  | RBM15B |  |  |
| CABLES1 | | PHF7 |  |  |
| ARHGEF16 | | REM1 |  |  |
| NDUFA11 | | ARHGAP19 | |  |
| SPR |  | BCL7A |  |  |
| COX17 |  | EAF1 |  |  |
| CENPA |  | SLC16A3 |  |  |
| 2210016F16RIK | | MTMR11 | |  |
| KXD1 |  | NDUFAF3 | |  |
| RARS2 |  | GTF3C5 |  |  |
| CES1D |  | RFX1 |  |  |
| UBR5 |  | NUP88 |  |  |
| PLA2G16 | | PLIN2 |  |  |
| CASQ2 |  | NHEJ1 |  |  |
| TCEB1 |  | LINGO1 |  |  |
| LPIN1 |  | PLEKHJ1 | |  |
| RBM4 |  | CATSPERE2 | |  |
| SLCO2B1 | | FCER2A |  |  |
| COX7C |  | RALBP1 |  |  |
| PPIF |  | NKD1 |  |  |
| COMTD1 | | NPM3 |  |  |
| TCEA3 |  | CTSL |  |  |
| SIX5 |  | MAOA |  |  |
| TMEM45A | | PLAGL2 |  |  |
| HACE1 |  | GRK2 |  |  |
| OLFML1 |  | SESN3 |  |  |
| TNFAIP8L3 | | HNRNPDL | |  |
| ZFP474 |  | PFDN6 |  |  |
| AASS |  | GIF |  |  |
| SLC25A10 | | RMND5B | |  |
| SREK1IP1 | | GM6685 |  |  |
| SLC25A25 | | SNX7 |  |  |
| NISCH |  | RGCC |  |  |
| SLC7A13 |  | RAPH1 |  |  |
| ZFP444 |  | RALB |  |  |
| AGL |  | CHFR |  |  |
| POLR3A |  | LAMA2 |  |  |
| NFE2L1 |  | PDCD10 |  |  |
| FAM192A | | MUCL3 |  |  |
| SEPHS1 |  | HIVEP3 |  |  |
| PHOSPHO2 | | PDIA4 |  |  |
| AP2M1 |  | PFKL |  |  |
| NTF5 |  | ATG2B |  |  |
| 43527 |  | CRTC2 |  |  |
| UGT2A3 |  | UBR5 |  |  |
| TEP1 |  | NOP10 |  |  |
| OLR1 |  | ACSL1 |  |  |
| STAU2 |  | MPC1 |  |  |
| LUZP2 |  | CLK1 |  |  |
| N4BP2L2 | | CHMP3 |  |  |
| SENP2 |  | ITGB1BP1 | |  |
| MTAP |  | HSCB |  |  |
| NGLY1 |  | MTA2 |  |  |
| NARS2 |  | TSSC4 |  |  |
| CEP170B |  | CCDC120 | |  |
| IMP3 |  | PDHA1 |  |  |
| B430305J03RIK | | GALK2 |  |  |
| GTF2B |  | CFAP97 |  |  |
| SAPCD2 |  | SAMD10 |  |  |
| FUCA2 |  | ATXN7L3 | |  |
| MRC1 |  | CAPRIN1 | |  |
| MICAL3 |  | OLFR689 |  |  |
| TDP1 |  | ATP1B3 |  |  |
| APEX1 |  | ARHGEF40 | |  |
| FGB |  | LRRIQ3 |  |  |
| C1RL |  | CREB3 |  |  |
| DIS3 |  | ANKRD37 | |  |
| TTC19 |  | RAX |  |  |
| CYCS |  | ESRP1 |  |  |
| NLN |  | ABRAXAS1 | |  |
| TACC2 |  | TRMT112 | |  |
| KCTD3 |  | UBE2H |  |  |
| RBFA |  | ASTE1 |  |  |
| PSMD5 |  | NANS |  |  |
| GJB1 |  | AKAP6 |  |  |
| PRKCDBP | | COL9A1 |  |  |
| PM20D1 |  | H2-OA |  |  |
| SLC35E1 |  | CYP51 |  |  |
| FTSJ1 |  | GLP2R |  |  |
| TNFAIP8 |  | CHURC1 |  |  |
| AW209491 | | DGUOK |  |  |
| CCDC134 | | SNX18 |  |  |
| AGT |  | KCNJ9 |  |  |
| PPP1R35 |  | GM10271 | |  |
| KMT2E |  | HAGH |  |  |
| TMEM176B | | IVNS1ABP | |  |
| DUT |  | MAPRE1 |  |  |
| AI462493 |  | LYAR |  |  |
| ADAT1 |  | ATF4 |  |  |
| RNF208 |  | FAM171B | |  |
| ZFP467 |  | SLC6A17 |  |  |
| SSR1 |  | LRRTM3 |  |  |
| GCC2 |  | EFNB1 |  |  |
| KCNT2 |  | SLC3A1 |  |  |
| ILKAP |  | HSD17B1 | |  |
| RPUSD2 |  | C730034F03RIK | |  |
| CCR8 |  | SLC41A2 |  |  |
| SLAMF7 |  | GPM6B |  |  |
| 1700047I17RIK2 | | BAIAP2L1 | |  |
| UTP3 |  | GM826 |  |  |
| ENO3 |  | SC5D |  |  |
| PCX |  | PGAP2 |  |  |
| HIST1H4M | | BBS1 |  |  |
| SLC38A4 |  | SST |  |  |
| FLAD1 |  | EIF4A2 |  |  |
| SORT1 |  | PTPN7 |  |  |
| F13A1 |  | SCAF1 |  |  |
| UBAC1 |  | OLFR1423 | |  |
| BOLA3 |  | PLCG2 |  |  |
| SSB |  | CCDC166 | |  |
| ATP8B2 |  | ABCB1A |  |  |
| RPF2 |  | R3HCC1 |  |  |
| FGA |  | POLR3G |  |  |
| PSMB4 |  | ZFP354B |  |  |
| SYT15 |  | VSX2 |  |  |
| MIPEP |  | LEF1 |  |  |
| TIMM23 |  | PRPS1L1 |  |  |
| RELA |  | CKMT1 |  |  |
| NT5C3 |  | FNDC9 |  |  |
| TMEM192 | | SLC25A54 | |  |
| SAFB |  | SLAMF7 |  |  |
| SAV1 |  | RPL15 |  |  |
| TSSK6 |  | CCDC9B |  |  |
| TRPC5 |  | 3110070M22RIK | |  |
| HDHD3 |  | SKOR1 |  |  |
| CISD1 |  | RAI2 |  |  |
| AGR3 |  | PKHD1L1 | |  |
| MAP3K12 | | ASCL2 |  |  |
| EMC6 |  | STAT6 |  |  |
| KDM1B |  | ARPIN |  |  |
| SLC3A1 |  | SPTBN1 |  |  |
| DCUN1D5 | | COG5 |  |  |
| RHOD |  | TIMM22 |  |  |
| ZFP830 |  | EIF3K |  |  |
| HYPK |  | ST3GAL6 | |  |
| CDCA4 |  | DAG1 |  |  |
| UBE2J2 |  | 10-Mar |  |  |
| HERC4 |  | ENTPD1 |  |  |
| NOD1 |  | BCL2L15 |  |  |
| FBXW2 |  | CNTNAP3 | |  |
| DLD |  | NGF |  |  |
| F13B |  | TRMT13 |  |  |
| PIK3AP1 |  | TELO2 |  |  |
| DGKI |  | YDJC |  |  |
| GPR161 |  | PRL8A1 |  |  |
| POLR1E |  | GM8909 |  |  |
| CIDEC |  | SLC15A1 |  |  |
| ANKRD42 | | CTSE |  |  |
| MAN2B2 |  | RDH7 |  |  |
| TMED9 |  | SSXB10 |  |  |
| PLCD4 |  | OLFR32 |  |  |
| ATP5O |  | VMN1R237 | |  |
| NAP1L4 |  | MCOLN3 | |  |
| SLIRP |  | GRXCR1 |  |  |
| NCL |  | ACNAT2 |  |  |
| CEP57 |  | 4930519G04RIK | |  |
| SLC25A45 | | GM13283 | |  |
| PCID2 |  | ZFAT |  |  |
| SULT1C2 | | CRLS1 |  |  |
| ZMAT2 |  | SLITRK3 |  |  |
| PREP |  | STX7 |  |  |
| TBC1D10A | | RNF17 |  |  |
| LSM6 |  | MYH14 |  |  |
| SDCCAG3 | | TMEM229B | |  |
| PRR12 |  | KBTBD6 |  |  |
| PPP2R3A | | ZFP456 |  |  |
| UMPS |  | WBP1L |  |  |
| EMB |  | SMARCA1 | |  |
| ADCK2 |  | CAR5B |  |  |
| PDHX |  | TUBA3A |  |  |
| SOD1 |  | SMTN |  |  |
| YWHAQ |  | DXO |  |  |
| TRPC4 |  | NES |  |  |
| H2-Q10 |  | MTFR1 |  |  |
| GJA8 |  | JDP2 |  |  |
| UACA |  | PPP1R14B | |  |
| CHTOP |  | GPSM1 |  |  |
| ESYT2 |  | CD2 |  |  |
| OCLN |  | SERPINA1D | |  |
| EWSR1 |  | LRRC37A | |  |
| CYP2B13 | | CHEK1 |  |  |
| CEP170 |  | ARFGEF1 | |  |
| ZBTB25 |  | DHX33 |  |  |
| BCAT2 |  | TCERG1L | |  |
| NUDCD3 |  | CCL7 |  |  |
| FBP1 |  | CLEC14A | |  |
| SYT10 |  | TAF8 |  |  |
| ITIH4 |  | E2F8 |  |  |
| TUBB3 |  | PMAIP1 |  |  |
| RPLP2 |  | SEC14L2 |  |  |
| FAH |  | SLC39A9 |  |  |
| TTC30B |  | WASL |  |  |
| IFNAR2 |  | DCAF10 |  |  |
| ADSSL1 |  | SYN3 |  |  |
| AP5B1 |  | CPT1A |  |  |
| RBCK1 |  | SRSF10 |  |  |
| BRAP |  | OSBP2 |  |  |
| PNPLA6 |  | FKBP14 |  |  |
| TINAGL1 | | D630045J12RIK | |  |
| DEB1 |  | ADGRL4 |  |  |
| TLN1 |  | KCNK10 |  |  |
| UPB1 |  | CCDC12 |  |  |
| PWP1 |  | RTL6 |  |  |
| CETN3 |  | PSMD2 |  |  |
| FAM179A | | TRP53INP1 | |  |
| POLR1A |  | CEPT1 |  |  |
| PPARG |  | GABRG3 |  |  |
| FAM135B | | FMNL2 |  |  |
| OS9 |  | IL21R |  |  |
| AKAP5 |  | CRB1 |  |  |
| SCN7A |  | DPAGT1 |  |  |
| PSME2B |  | CD300C2 |  |  |
| RSU1 |  | RIC1 |  |  |
| ANXA3 |  | JAG2 |  |  |
| KRTCAP2 | | ZFP91 |  |  |
| HAVCR2 |  | IPPK |  |  |
| CHD1 |  | MMD |  |  |
| MYH1 |  | UFL1 |  |  |
| CDKN3 |  | PCDHGC3 | |  |
| ECI1 |  | STOX2 |  |  |
| MRC2 |  | TEC |  |  |
| LEO1 |  | GLRX2 |  |  |
| GSTCD |  | IGF2R |  |  |
| TUFT1 |  | SH3YL1 |  |  |
| USP18 |  | ALKBH3 |  |  |
| CYP2C40 | | FGF10 |  |  |
| PPWD1 |  | PIWIL2 |  |  |
| DLGAP2 |  | RNASE10 | |  |
| ZFP804A |  | KLHL18 |  |  |
| TMEM165 | | SS18L1 |  |  |
| SLC13A1 |  | MACO1 |  |  |
| PPP2R2C | | THAP7 |  |  |
| CES3B |  | WDR93 |  |  |
| ADPRHL1 | | RAB3IL1 |  |  |
| HCN1 |  | 1-Mar |  |  |
| CYP2J5 |  | HNRNPUL1 | |  |
| THAP4 |  | FAM49A |  |  |
| ARMC1 |  | ANKRD26 | |  |
| CYP4A14 | | UEVLD |  |  |
| PGAM5 |  | ZFYVE9 |  |  |
| MTERFD3 | | CLVS2 |  |  |
| PIH1D1 |  | NUFIP2 |  |  |
| PEPD |  | KCTD13 |  |  |
| ATXN10 |  | HKDC1 |  |  |
| GYK |  | MAP3K10 | |  |
| METTL21C | | RTN4IP1 |  |  |
| SMEK2 |  | PXN |  |  |
| MSRB1 |  | TMEM260 | |  |
| APIP |  | PNN |  |  |
| AVEN |  | UBL7 |  |  |
| TRPM7 |  | ABHD11 |  |  |
| GM20498 | | GLYR1 |  |  |
| TIMM8B |  | TACSTD2 | |  |
| SPINT2 |  | PRKAR1A | |  |
| TUSC3 |  | CNPY3 |  |  |
| CNOT10 |  | TMEM115 | |  |
| RND3 |  | GNB3 |  |  |
| CNOT8 |  | SBF1 |  |  |
| PTPRA |  | KAT7 |  |  |
| UROC1 |  | PHF21A |  |  |
| YIPF7 |  | VGF |  |  |
| EIF4EBP1 | | MARVELD2 | |  |
| LGI1 |  | PRPH |  |  |
| SCP2 |  | TMCC3 |  |  |
| PPP2R5A | | CRISPLD2 | |  |
| HS2ST1 |  | RHBDD2 |  |  |
| LBH |  | EHF |  |  |
| COG2 |  | TCIRG1 |  |  |
| AK1 |  | ATP2A1 |  |  |
| COL3A1 |  | CPM |  |  |
| MDM4 |  | CUEDC2 |  |  |
| KMT2B |  | HSPB9 |  |  |
| AMFR |  | SYT16 |  |  |
| KBTBD3 |  | ASNSD1 |  |  |
| MOB2 |  | CHAMP1 |  |  |
| SERPINA3K | | ZFP318 |  |  |
| PSMB1 |  | SH3RF1 |  |  |
| CBLN2 |  | ZMAT2 |  |  |
| FMO3 |  | APMAP |  |  |
| UGT2B36 | | LGMN |  |  |
| UGGT1 |  | RAD21L |  |  |
| SERGEF |  | TMED5 |  |  |
| DNMT3L | | ANGPTL2 | |  |
| VMP1 |  | CX3CL1 |  |  |
| TWIST1 |  | GM11099 | |  |
| HECTD1 |  | UPP2 |  |  |
| SCAF8 |  | STARD5 |  |  |
| LLGL2 |  | KRT9 |  |  |
| NMD3 |  | BCL2L11 |  |  |
| RAB18 |  | ZEB2 |  |  |
| ATAD1 |  | ZFP354C |  |  |
| EPHB4 |  | PKNOX2 |  |  |
| PSD4 |  | CNR2 |  |  |
| SLC22A1 |  | CDR2 |  |  |
| CAPNS1 |  | CYB5RL |  |  |
| POLR1D |  | GPR182 |  |  |
| RPL26 |  | SPHK1 |  |  |
| GPM6A |  | PCDH9 |  |  |
| DSCAM |  | SNCB |  |  |
| VASN |  | CD93 |  |  |
| FNTA |  | GALR2 |  |  |
| NSMF |  | PPP4R3A | |  |
| MICU3 |  | VMN2R85 | |  |
| VIM |  | BCAP29 |  |  |
| 1200014J11RIK | | CABIN1 |  |  |
| PAWR |  | ERCC8 |  |  |
| CDH18 |  | MT-ATP8 | |  |
| ADRA1D |  | MUP5 |  |  |
| UBTD2 |  | PRORSD1 | |  |
| SPECC1L | | PSMB10 |  |  |
| PLET1 |  | LMCD1 |  |  |
| FDFT1 |  | ANXA9 |  |  |
| KDELR1 |  | DCTD |  |  |
| BTG2 |  | IL2RA |  |  |
| ACOT13 |  | HEXDC |  |  |
| COMMD2 | | GTPBP2 |  |  |
| CD8B1 |  | CDC20B |  |  |
| NDUFB9 |  | TAZ |  |  |
| ATP10A |  | MSANTD4 | |  |
| DNM1L |  | DDIAS |  |  |
| PPP4R1 |  | H2-KE6 |  |  |
| MRPL51 |  | NSG1 |  |  |
| KATNA1 |  | EPB41L2 |  |  |
| SMARCAL1 | | DGKH |  |  |
| TFR2 |  | RORC |  |  |
| SECISBP2L | | LHFPL5 |  |  |
| CRAT |  | CAMK2A | |  |
| SDR9C7 |  | SMTNL2 |  |  |
| ADIG |  | ADAMTS14 | |  |
| SEPW1 |  | FPGS |  |  |
| CCT5 |  | IL1RAP |  |  |
| KBTBD2 |  | PWP1 |  |  |
| GM14391 | | MCM6 |  |  |
| CD99L2 |  | BAZ2A |  |  |
| WWP2 |  | PDCL3 |  |  |
| TUBB2B |  | RASL11B | |  |
| SGK3 |  | IGFBP7 |  |  |
| BCKDHB | | RSU1 |  |  |
| UGP2 |  | MORF4L2 | |  |
| CDK5RAP3 | | MRPS16 |  |  |
| DMGDH |  | IL17C |  |  |
| KDM4B |  | TM2D1 |  |  |
| FBXO38 |  | GNG13 |  |  |
| WDR75 |  | VANGL2 |  |  |
| GPX7 |  | 5730480H06RIK | |  |
| FOPNL |  | 2810408A11RIK | |  |
| HEATR3 |  | GM11084 | |  |
| RBBP4 |  | SORCS2 |  |  |
| IFITM2 |  | MAP9 |  |  |
| COLQ |  | COX19 |  |  |
| UBC |  | ELP4 |  |  |
| PLCXD1 |  | KMT2D |  |  |
| MOAP1 |  | CAPN15 |  |  |
| QSOX1 |  | TMEM248 | |  |
| CCDC154 | | SMARCB1 | |  |
| UNC45B |  | SLC10A1 |  |  |
| DGKG |  | ACP4 |  |  |
| UAP1L1 |  | CEP295 |  |  |
| AAR2 |  | DYNLT1F | |  |
| LMAN2 |  | NDUFA12 | |  |
| DPPA2 |  | ELOVL5 |  |  |
| TMCC3 |  | SDC3 |  |  |
| RIF1 |  | GAD1 |  |  |
| NDUFA4 |  | LDLRAD4 | |  |
| SDC1 |  | TNK2 |  |  |
| SLC38A10 | | USP33 |  |  |
| SLC39A14 | | ACP7 |  |  |
| DCP1B |  | NBDY |  |  |
| 2900011O08RIK | | BDKRB2 |  |  |
| GM20604 | | VAPB |  |  |
| ZAP70 |  | DLX2 |  |  |
| LILRA6 |  | LIMK2 |  |  |
| CBR2 |  | ESPNL |  |  |
| SQRDL |  | NABP2 |  |  |
| S100G |  | DACT1 |  |  |
| RTN4RL1 | | OPRD1 |  |  |
| MTFR1 |  | VMN2R29 | |  |
| RALGAPB | | ELAVL4 |  |  |
| PRR13 |  | SLC12A1 |  |  |
| GRK6 |  | FMO2 |  |  |
| BPNT1 |  | GM13097 | |  |
| IL6ST |  | CD14 |  |  |
| MN1 |  | CRH |  |  |
| DPH7 |  | STON1 |  |  |
| C1QB |  | EPB41L3 |  |  |
| PZP |  | SYNGAP1 | |  |
| SLAIN2 |  | SIK1 |  |  |
| MLLT4 |  | MFSD11 |  |  |
| AMZ2 |  | ITCH |  |  |
| HIVEP1 |  | KLF13 |  |  |
| TOR1B |  | LDLRAD1 | |  |
| EMC2 |  | BPIFB5 |  |  |
| SLC8A2 |  | DACT2 |  |  |
| 3110001I22RIK | | PSMA6 |  |  |
| SLCO1A1 | | ADGRF5 |  |  |
| NPTN |  | PWP2 |  |  |
| PAM |  | HOPX |  |  |
| AXL |  | SNX1 |  |  |
| TMEM206 | | RGL3 |  |  |
| UGDH |  | MYC |  |  |
| UBA5 |  | PHF11B |  |  |
| CRYZ |  | LARP6 |  |  |
| SMARCA4 | | TATDN2 |  |  |
| ASUN |  | PRR29 |  |  |
| PRR3 |  | ZBTB2 |  |  |
| NACC1 |  | APOB |  |  |
| CLTC |  | G6PDX |  |  |
| TBC1D19 | | ADPGK |  |  |
| OGDH |  | EEF1G |  |  |
| SUPV3L1 | | GAS8 |  |  |
| PCM1 |  | SAE1 |  |  |
| CUL1 |  | CTIF |  |  |
| LYRM2 |  | DNAJB9 |  |  |
| EHD1 |  | FOXD1 |  |  |
| WDR1 |  | GLS |  |  |
| ASTE1 |  | PHEX |  |  |
| USP39 |  | CUL4A |  |  |
| DPP6 |  | TNFSF13 |  |  |
| RPS3 |  | TAF4B |  |  |
| KLF2 |  | FBF1 |  |  |
| ZFP14 |  | AKAIN1 |  |  |
| MRPL14 |  | ZFP513 |  |  |
| ACACA |  | DHCR24 |  |  |
| PARG |  | POP1 |  |  |
| ING2 |  | GPSM3 |  |  |
| GRB7 |  | BLMH |  |  |
| ZYG11B |  | ZFP786 |  |  |
| TMEM184B | | DDX21 |  |  |
| ZMYND11 | | CLCC1 |  |  |
| SLC16A4 |  | LRRC40 |  |  |
| FKRP |  | FANCI |  |  |
| LPHN2 |  | PDE7A |  |  |
| UBA6 |  | CALCOCO1 | |  |
| NEDD8 |  | TYW5 |  |  |
| SLC25A4 |  | PIEZO2 |  |  |
| ATP8A1 |  | KIF9 |  |  |
| VBP1 |  | PCM1 |  |  |
| ANKRD49 | | FARSA |  |  |
| PRSS16 |  | ATCAY |  |  |
| ACOX1 |  | TCEAL3 |  |  |
| OGN |  | PBXIP1 |  |  |
| ACTB |  | KCNG4 |  |  |
| SLC25A15 | | FLAD1 |  |  |
| PHYH |  | SLC13A1 |  |  |
| SPARC |  | LIN28B |  |  |
| ARFRP1 |  | SGCG |  |  |
| 43715 |  | GM10600 | |  |
| SLITRK3 |  | DISC1 |  |  |
| NRG1 |  | ZFAND6 |  |  |
| FXYD6 |  | NKAP |  |  |
| EPHA6 |  | TMEM82 |  |  |
| SAAL1 |  | CIB2 |  |  |
| COX6C |  | GNAT1 |  |  |
| SKAP1 |  | ACYP2 |  |  |
| RPL37A |  | OLFR654 |  |  |
| IFFO2 |  | 8-Mar |  |  |
| D6WSU163E | | RTCB |  |  |
| MMP20 |  | EPS15L1 |  |  |
| THSD7B |  | NUDCD2 |  |  |
|  |  | MYOM1 |  |  |
|  |  | TRP53I11 | |  |
|  |  | IFI35 |  |  |
|  |  | TNFAIP8L2 | |  |
|  |  | VCL |  |  |
|  |  | GM13199 | |  |
|  |  | CPEB4 |  |  |
|  |  | PKIA |  |  |
|  |  | SERPINB1B | |  |
|  |  | SERF2 |  |  |
|  |  | GIPR |  |  |
|  |  | HIST1H2AD | |  |
|  |  | PSMG3 |  |  |
|  |  | CHMP2B |  |  |
|  |  | ALS2CR12 | |  |
|  |  | IER3 |  |  |
|  |  | FAIM |  |  |
|  |  | PRKCQ |  |  |
|  |  | LRRC18 |  |  |
|  |  | TBL1XR1 | |  |
|  |  | SLC25A20 | |  |
|  |  | SALL4 |  |  |
|  |  | B3GALT4 | |  |
|  |  | SULT6B2 | |  |
|  |  | CCDC28B | |  |
|  |  | HES7 |  |  |
|  |  | ZFP951 |  |  |
|  |  | GM10277 | |  |
|  |  | SCARF2 |  |  |
|  |  | NSMCE3 |  |  |
|  |  | MTRF1L |  |  |
|  |  | LBH |  |  |
|  |  | MSL1 |  |  |
|  |  | DPH5 |  |  |
|  |  | TTC30A2 | |  |
|  |  | NAP1L2 |  |  |
|  |  | CEBPB |  |  |
|  |  | 2-Mar |  |  |
|  |  | PLXDC1 |  |  |
|  |  | SAMD12 |  |  |
|  |  | NCKAP5L | |  |
|  |  | ZFP943 |  |  |
|  |  | CATSPERG2 | |  |
|  |  | ABI1 |  |  |
|  |  | FOS |  |  |
|  |  | STK40 |  |  |
|  |  | A930017K11RIK | |  |
|  |  | CASTOR2 | |  |
|  |  | SYCE1 |  |  |
|  |  | PSAP |  |  |
|  |  | RBM12B2 | |  |
|  |  | WASF3 |  |  |
|  |  | FRYL |  |  |
|  |  | ATP11C |  |  |
|  |  | NUDT13 |  |  |
|  |  | CHD1L |  |  |
|  |  | VDAC1 |  |  |
|  |  | MAN2C1 |  |  |
|  |  | YTHDC1 |  |  |
|  |  | PJA1 |  |  |
|  |  | SIGMAR1 | |  |
|  |  | PIAS3 |  |  |
|  |  | UBL5 |  |  |
|  |  | KCNC2 |  |  |
|  |  | ZFP1 |  |  |
|  |  | 4930550C14RIK | |  |
|  |  | THEM7 |  |  |
|  |  | CRX |  |  |
|  |  | FGR |  |  |
|  |  | SMCO2 |  |  |
|  |  | FEZF1 |  |  |
|  |  | ANG2 |  |  |
|  |  | OLFR570 |  |  |
|  |  | 2310007B03RIK | |  |
|  |  | UPK3B |  |  |
|  |  | OLFR95 |  |  |
|  |  | SMOK2B |  |  |
|  |  | TMEM217 | |  |
|  |  | WFDC2 |  |  |
|  |  | ADAM2 |  |  |
|  |  | SHLD2 |  |  |
|  |  | ERBB3 |  |  |
|  |  | ZBTB8OS | |  |
|  |  | D1ERTD622E | |  |
|  |  | TCAP |  |  |
|  |  | GNG2 |  |  |
|  |  | ALPK2 |  |  |
|  |  | FJX1 |  |  |
|  |  | MRPL37 |  |  |
|  |  | ECEL1 |  |  |
|  |  | STYXL1 |  |  |
|  |  | PITX1 |  |  |
|  |  | ASCL5 |  |  |
|  |  | PIGP |  |  |
|  |  | TMEM231 | |  |
|  |  | OLFM3 |  |  |
|  |  | FCGR2B |  |  |
|  |  | FKTN |  |  |
|  |  | ABCA9 |  |  |
|  |  | FUT9 |  |  |
|  |  | RCAN1 |  |  |
|  |  | CASKIN1 | |  |
|  |  | CHIA1 |  |  |
|  |  | OLFR114 |  |  |
|  |  | NR1I2 |  |  |
|  |  | HILPDA |  |  |
|  |  | TOX3 |  |  |
|  |  | AMELX |  |  |
|  |  | MCM5 |  |  |
|  |  | ACAA1B |  |  |
|  |  | ACTN2 |  |  |
|  |  | RPE65 |  |  |
|  |  | GM21680 | |  |
|  |  | PCNT |  |  |
|  |  | RBM17 |  |  |
|  |  | SLC2A9 |  |  |
|  |  | SRSF3 |  |  |
|  |  | GM14399 | |  |
|  |  | AQP5 |  |  |
|  |  | ST3GAL1 | |  |
|  |  | ACOX3 |  |  |
|  |  | FAF1 |  |  |
|  |  | CARHSP1 | |  |
|  |  | GALE |  |  |
|  |  | ZFP474 |  |  |
|  |  | MRM1 |  |  |
|  |  | PTPRA |  |  |
|  |  | CNIH3 |  |  |
|  |  | GPAT2 |  |  |
|  |  | MYH15 |  |  |
|  |  | ENPP1 |  |  |
|  |  | WNT4 |  |  |
|  |  | LRCH2 |  |  |
|  |  | VPREB3 |  |  |
|  |  | CLCN1 |  |  |
|  |  | BRINP1 |  |  |
|  |  | E2F7 |  |  |
|  |  | CPOX |  |  |
|  |  | CCHCR1 |  |  |
|  |  | CCND1 |  |  |
|  |  | RAB5A |  |  |
|  |  | DEPDC1B | |  |
|  |  | BHLHE41 | |  |
|  |  | PPIL3 |  |  |
|  |  | ZFP704 |  |  |
|  |  | KLK11 |  |  |
|  |  | AGTR2 |  |  |
|  |  | COLQ |  |  |
|  |  | FOXF2 |  |  |
|  |  | SEC11A |  |  |
|  |  | ICE1 |  |  |
|  |  | SYNJ2BP | |  |
|  |  | PTX3 |  |  |
|  |  | ADORA2B | |  |
|  |  | MACROD2 | |  |
|  |  | HIST1H4B | |  |
|  |  | SEC24A |  |  |
|  |  | 4930562C15RIK | |  |
|  |  | SDHC |  |  |
|  |  | SIAH2 |  |  |
|  |  | TMUB1 |  |  |
|  |  | TMEM210 | |  |
|  |  | HNRNPA3 | |  |
|  |  | CER1 |  |  |
|  |  | PLEKHG1 | |  |
|  |  | SLC6A20B | |  |
|  |  | NTN1 |  |  |
|  |  | 2010300C02RIK | |  |
|  |  | RCC1 |  |  |
|  |  | MROH6 |  |  |
|  |  | MT-CYTB | |  |
|  |  | FUT8 |  |  |
|  |  | USPL1 |  |  |
|  |  | DTX1 |  |  |
|  |  | ACACA |  |  |
|  |  | ADAMTSL1 | |  |
|  |  | DNAJB1 |  |  |
|  |  | TFAP2D |  |  |
|  |  | SLCO2A1 | |  |
|  |  | COX10 |  |  |
|  |  | SLC26A1 |  |  |
|  |  | ANAPC5 |  |  |
|  |  | GM17720 | |  |
|  |  | CNNM3 |  |  |
|  |  | UFM1 |  |  |
|  |  | ACKR2 |  |  |
|  |  | SFPQ |  |  |
|  |  | CCNJL |  |  |
|  |  | JPT2 |  |  |
|  |  | GPX4 |  |  |
|  |  | SGK3 |  |  |
|  |  | FSTL3 |  |  |
|  |  | RPL28 |  |  |
|  |  | TSEN54 |  |  |
|  |  | PRMT9 |  |  |
|  |  | TIA1 |  |  |
|  |  | PEX26 |  |  |
|  |  | LRRTM4 |  |  |
|  |  | GEMIN7 |  |  |
|  |  | SMYD5 |  |  |
|  |  | HSPB8 |  |  |
|  |  | EMC10 |  |  |
|  |  | PRKAR2A | |  |
|  |  | ST7 |  |  |
|  |  | GM17655 | |  |
|  |  | NOG |  |  |
|  |  | GTPBP4 |  |  |
|  |  | TBC1D19 | |  |
|  |  | CDK5RAP2 | |  |
|  |  | CD53 |  |  |
|  |  | GAS1 |  |  |
|  |  | GRID2 |  |  |
|  |  | FADS6 |  |  |
|  |  | PFKM |  |  |
|  |  | TMEM219 | |  |
|  |  | SYNPR |  |  |
|  |  | SNRPE |  |  |
|  |  | GPRASP1 | |  |
|  |  | SLC35E4 |  |  |
|  |  | NUTF2 |  |  |
|  |  | EFCAB12 | |  |
|  |  | BGLAP3 |  |  |
|  |  | MTHFD2L | |  |
|  |  | RPS28 |  |  |
|  |  | COX5A |  |  |
|  |  | DLGAP1 |  |  |
|  |  | HDGFL2 |  |  |
|  |  | MAN2A1 |  |  |
|  |  | COMMD1 | |  |
|  |  | LSM11 |  |  |
|  |  | CELF6 |  |  |
|  |  | USP28 |  |  |
|  |  | NFKBIE |  |  |
|  |  | EGF |  |  |
|  |  | TRP53TG5 | |  |
|  |  | PLCD1 |  |  |
|  |  | SPATA46 | |  |
|  |  | PRAM1 |  |  |
|  |  | PGBD1 |  |  |
|  |  | SGO2A |  |  |
|  |  | ARL4A |  |  |
|  |  | TCF7 |  |  |
|  |  | ADRB3 |  |  |
|  |  | AP5B1 |  |  |
|  |  | TRP53INP2 | |  |
|  |  | AAMDC |  |  |
|  |  | TERF2IP |  |  |
|  |  | POLE2 |  |  |
|  |  | TNS1 |  |  |
|  |  | PFKP |  |  |
|  |  | FAM102A | |  |
|  |  | GBP5 |  |  |
|  |  | ARPC5L |  |  |
|  |  | SZRD1 |  |  |
|  |  | TNFSF18 |  |  |
|  |  | ADGRD1 |  |  |
|  |  | CSNK1D |  |  |
|  |  | IFITM1 |  |  |
|  |  | EVC2 |  |  |
|  |  | HYAL5 |  |  |
|  |  | GM10447 | |  |
|  |  | SLC10A6 |  |  |
|  |  | CATSPER1 | |  |
|  |  | ZFP638 |  |  |
|  |  | ZFP473 |  |  |
|  |  | SMIM20 |  |  |
|  |  | PIGB |  |  |
|  |  | TTLL4 |  |  |
|  |  | P2RY4 |  |  |
|  |  | MYMK |  |  |
|  |  | DYNLT1A | |  |
|  |  | TCF19 |  |  |
|  |  | GUF1 |  |  |
|  |  | SCARA3 |  |  |
|  |  | HNRNPH2 | |  |
|  |  | JMJD7 |  |  |
|  |  | LRRC8E |  |  |
|  |  | RAB9B |  |  |
|  |  | SAMD8 |  |  |
|  |  | PRADC1 |  |  |
|  |  | ZFP974 |  |  |
|  |  | FRY |  |  |
|  |  | RNH1 |  |  |
|  |  | IDH3A |  |  |
|  |  | DARS |  |  |
|  |  | PDS5A |  |  |
|  |  | SLC17A6 |  |  |
|  |  | SPEF2 |  |  |
|  |  | PIK3R4 |  |  |
|  |  | NDRG1 |  |  |
|  |  | CST3 |  |  |
|  |  | TYROBP |  |  |
|  |  | MYCN |  |  |
|  |  | LRRC66 |  |  |
|  |  | PHF5A |  |  |
|  |  | PHF11D |  |  |
|  |  | SLC26A8 |  |  |
|  |  | AKR1A1 |  |  |
|  |  | RARB |  |  |
|  |  | PLEKHN1 | |  |
|  |  | SLC37A4 |  |  |
|  |  | THRB |  |  |
|  |  | LMAN1L |  |  |
|  |  | RPF1 |  |  |
|  |  | ZFP292 |  |  |
|  |  | COL2A1 |  |  |
|  |  | TRIM32 |  |  |
|  |  | MROH5 |  |  |
|  |  | SOX7 |  |  |
|  |  | ZFP551 |  |  |
|  |  | 4933434E20RIK | |  |
|  |  | HECTD1 |  |  |
|  |  | CACUL1 |  |  |
|  |  | ERCC5 |  |  |
|  |  | TENT5A |  |  |
|  |  | BMI1 |  |  |
|  |  | SYNPO2L | |  |
|  |  | NAB2 |  |  |
|  |  | D730048I06RIK | |  |
|  |  | 2210010C04RIK | |  |
|  |  | 1700001C19RIK | |  |
|  |  | GM28035 | |  |
|  |  | GM42417 | |  |
|  |  | PSG17 |  |  |
|  |  | 2610028H24RIK | |  |
|  |  | GM45140 | |  |
|  |  | HABP2 |  |  |
|  |  | AOC1 |  |  |
|  |  | FFAR2 |  |  |
|  |  | MS4A4B |  |  |
|  |  | SOHLH1 |  |  |
|  |  | 4930567H17RIK | |  |
|  |  | SLC6A14 |  |  |
|  |  | POM121L2 | |  |
|  |  | PLA2G2E | |  |
|  |  | OLFR56 |  |  |
|  |  | MFSD4B3 | |  |
|  |  | MRGPRA1 | |  |
|  |  | 1700016D06RIK | |  |
|  |  | SPATA31 | |  |
|  |  | ATP6V1G3 | |  |
|  |  | NR3C1 |  |  |
|  |  | BCL3 |  |  |
|  |  | SEM1 |  |  |
|  |  | PCYOX1L | |  |
|  |  | PIM3 |  |  |
|  |  | SAT2 |  |  |
|  |  | GUCY2G |  |  |
|  |  | DPP9 |  |  |
|  |  | HEXIM1 |  |  |
|  |  | SEC23IP |  |  |
|  |  | GM6563 |  |  |
|  |  | SERTAD2 | |  |
|  |  | FAAP20 |  |  |
|  |  | VSX1 |  |  |
|  |  | CAAP1 |  |  |
|  |  | CCDC114 | |  |
|  |  | HTR5A |  |  |
|  |  | PAPOLB |  |  |
|  |  | SSNA1 |  |  |
|  |  | TNFRSF11B | |  |
|  |  | ERMAP |  |  |
|  |  | KCNN3 |  |  |
|  |  | ABCA1 |  |  |
|  |  | AKAP9 |  |  |
|  |  | CMSS1 |  |  |
|  |  | LRP12 |  |  |
|  |  | ARXES2 |  |  |
|  |  | MTX2 |  |  |
|  |  | PDIA2 |  |  |
|  |  | ZFP703 |  |  |
|  |  | CAMK2B | |  |
|  |  | ARHGEF7 | |  |
|  |  | RUNDC1 |  |  |
|  |  | GANC |  |  |
|  |  | ANKRD29 | |  |
|  |  | NR5A2 |  |  |
|  |  | DPPA1 |  |  |
|  |  | CD300LD | |  |
|  |  | GM42906 | |  |
|  |  | ZFP212 |  |  |
|  |  | THRA |  |  |
|  |  | TMEM221 | |  |
|  |  | GRXCR2 |  |  |
|  |  | NT5C |  |  |
|  |  | UBAP2 |  |  |
|  |  | NAT10 |  |  |
|  |  | TXNL4B |  |  |
|  |  | AKIRIN2 |  |  |
|  |  | SDAD1 |  |  |
|  |  | MFAP1A |  |  |
|  |  | CTXN1 |  |  |
|  |  | CDC37L1 | |  |
|  |  | ZFP51 |  |  |
|  |  | IGF1R |  |  |
|  |  | AP3S1 |  |  |
|  |  | FOXJ3 |  |  |
|  |  | DIS3 |  |  |
|  |  | PCBP4 |  |  |
|  |  | THEM6 |  |  |
|  |  | XPNPEP2 | |  |
|  |  | TRIR |  |  |
|  |  | EIF3G |  |  |
|  |  | MPPE1 |  |  |
|  |  | PPP2R3C | |  |
|  |  | TXN1 |  |  |
|  |  | SMAD9 |  |  |
|  |  | DVL1 |  |  |
|  |  | SLC25A11 | |  |
|  |  | RPS23 |  |  |
|  |  | RNASE6 |  |  |
|  |  | PCDHA1 |  |  |
|  |  | CRACR2A | |  |
|  |  | SYT5 |  |  |
|  |  | SLC12A7 |  |  |
|  |  | MROH3 |  |  |
|  |  | GM11627 | |  |
|  |  | SCTR |  |  |
|  |  | IQCH |  |  |
|  |  | GSTM6 |  |  |
|  |  | ATP2B4 |  |  |
|  |  | EMC1 |  |  |
|  |  | HCAR1 |  |  |
|  |  | MET |  |  |
|  |  | MAP10 |  |  |
|  |  | HIP1 |  |  |
|  |  | TM9SF3 |  |  |
|  |  | SYK |  |  |
|  |  | SEC63 |  |  |
|  |  | CATSPERD | |  |
|  |  | SLC35B2 |  |  |
|  |  | PNPLA1 |  |  |
|  |  | UBXN8 |  |  |
|  |  | CXCL1 |  |  |
|  |  | STAM2 |  |  |
|  |  | CCSAP |  |  |
|  |  | KNL1 |  |  |
|  |  | RBM14 |  |  |
|  |  | PIP5KL1 |  |  |
|  |  | TMEM86A | |  |
|  |  | VPS8 |  |  |
|  |  | ARHGAP18 | |  |
|  |  | GAN |  |  |
|  |  | PTAFR |  |  |
|  |  | SNTG2 |  |  |
|  |  | SENP1 |  |  |
|  |  | 11-Sep |  |  |
|  |  | GPR50 |  |  |
|  |  | TNKS1BP1 | |  |
|  |  | STX16 |  |  |
|  |  | ZFP560 |  |  |
|  |  | GPR137B | |  |
|  |  | AZIN2 |  |  |
|  |  | FAM210B | |  |
|  |  | CPS1 |  |  |
|  |  | FBXL18 |  |  |
|  |  | MPZL1 |  |  |
|  |  | HERC2 |  |  |
|  |  | GBP8 |  |  |
|  |  | CES1A |  |  |
|  |  | CTNNBIP1 | |  |
|  |  | NEUROG1 | |  |
|  |  | IL18RAP |  |  |
|  |  | KLK1 |  |  |
|  |  | TDH |  |  |
|  |  | MKX |  |  |
|  |  | CYP46A1 | |  |
|  |  | CDO1 |  |  |
|  |  | NLRP6 |  |  |
|  |  | CRTAP |  |  |
|  |  | PRPSAP2 | |  |
|  |  | GTPBP3 |  |  |
|  |  | NCOA2 |  |  |
|  |  | MRPS35 |  |  |
|  |  | TWISTNB | |  |
|  |  | DNM3 |  |  |
|  |  | ITGA10 |  |  |
|  |  | UQCRC2 |  |  |
|  |  | ADCY7 |  |  |
|  |  | GINS1 |  |  |
|  |  | FAM166B | |  |
|  |  | CNIH1 |  |  |
|  |  | DAP |  |  |
|  |  | JSRP1 |  |  |
|  |  | PCDH15 |  |  |
|  |  | ADSL |  |  |
|  |  | P2RX2 |  |  |
|  |  | PARP11 |  |  |
|  |  | RPL13A |  |  |
|  |  | ATP2C2 |  |  |
|  |  | DNALI1 |  |  |
|  |  | RANGAP1 | |  |
|  |  | ADSSL1 |  |  |
|  |  | RNF130 |  |  |
|  |  | ANXA3 |  |  |
|  |  | ARPC1B |  |  |
|  |  | FZR1 |  |  |
|  |  | MT-CO1 |  |  |
|  |  | TMEM88B | |  |
|  |  | TGFA |  |  |
|  |  | BNC1 |  |  |
|  |  | KLHL3 |  |  |
|  |  | MLC1 |  |  |
|  |  | SNX12 |  |  |
|  |  | POLR2H |  |  |
|  |  | 6430548M08RIK | |  |
|  |  | ATG3 |  |  |
|  |  | GON4L |  |  |
|  |  | EGFL8 |  |  |
|  |  | RIMBP2 |  |  |
|  |  | H2-Q6 |  |  |
|  |  | SMIM5 |  |  |
|  |  | TSACC |  |  |
|  |  | HRCT1 |  |  |
|  |  | PTPRR |  |  |
|  |  | DAZAP2 |  |  |
|  |  | ASPH |  |  |
|  |  | SKAP2 |  |  |
|  |  | TYSND1 |  |  |
|  |  | SPATS2 |  |  |
|  |  | CCNL2 |  |  |
|  |  | CYP3A13 | |  |
|  |  | AK157302 | |  |
|  |  | PCDH11X | |  |
|  |  | RBM38 |  |  |
|  |  | SUPT6 |  |  |
|  |  | PET100 |  |  |
|  |  | CRYZL1 |  |  |
|  |  | BC037034 | |  |
|  |  | YIF1A |  |  |
|  |  | PRRT3 |  |  |
|  |  | USP14 |  |  |
|  |  | ROBO4 |  |  |
|  |  | APOL9B |  |  |
|  |  | A730015C16RIK | |  |
|  |  | ZBTB8A |  |  |
|  |  | OTOGL |  |  |
|  |  | PPARG |  |  |
|  |  | SP110 |  |  |
|  |  | PCDHB12 | |  |
|  |  | GRHPR |  |  |
|  |  | 4930455H04RIK | |  |
|  |  | SH3GL1 |  |  |
|  |  | SBF2 |  |  |
|  |  | MBD3 |  |  |
|  |  | D6ERTD527E | |  |
|  |  | SLC22A29 | |  |
|  |  | SLC6A21 |  |  |
|  |  | 1700003E16RIK | |  |
|  |  | FLT3L |  |  |
|  |  | EDC4 |  |  |
|  |  | TOR1AIP1 | |  |
|  |  | 1110059E24RIK | |  |
|  |  | 4932438A13RIK | |  |
|  |  | CTDSP2 |  |  |
|  |  | DMAC2 |  |  |
|  |  | BPIFB9A |  |  |
|  |  | FAM213A | |  |
|  |  | CLEC7A |  |  |
|  |  | GM10320 | |  |
|  |  | OLFR692 |  |  |
|  |  | AI606181 |  |  |
|  |  | ZFP609 |  |  |
|  |  | VIPR1 |  |  |
|  |  | NNT |  |  |
|  |  | TRIM63 |  |  |
|  |  | HSF3 |  |  |
|  |  | CCNG2 |  |  |
|  |  | NDUFA6 |  |  |
|  |  | PLAA |  |  |
|  |  | TLR12 |  |  |
|  |  | SIGIRR |  |  |
|  |  | SYNE4 |  |  |
|  |  | LY6G6D |  |  |
|  |  | EXOSC5 |  |  |
|  |  | GPR31B |  |  |
|  |  | TAGLN2 |  |  |
|  |  | GM12258 | |  |
|  |  | CCDC63 |  |  |
|  |  | CRIP3 |  |  |
|  |  | PPP1R3A | |  |
|  |  | RIPK4 |  |  |
|  |  | TNRC6C |  |  |
|  |  | MAGEE1 |  |  |
|  |  | GPR3 |  |  |
|  |  | STAR |  |  |
|  |  | OXSR1 |  |  |
|  |  | USP8 |  |  |
|  |  | GP1BA |  |  |
|  |  | GM3045 |  |  |
|  |  | AQP2 |  |  |
|  |  | GM3002 |  |  |
|  |  | IL31RA |  |  |
|  |  | TMEM9 |  |  |
|  |  | FBXW8 |  |  |
|  |  | CLCNKA |  |  |
|  |  | CERS3 |  |  |
|  |  | TDRD9 |  |  |
|  |  | BCAN |  |  |
|  |  | DCAF12L2 | |  |
|  |  | MRPL35 |  |  |
|  |  | OCIAD1 |  |  |
|  |  | SERP2 |  |  |
|  |  | CNOT6 |  |  |
|  |  | ACADL |  |  |
|  |  | RPA3 |  |  |
|  |  | GM28551 | |  |
|  |  | OLFR1417 | |  |
|  |  | PCDHGA7 | |  |
|  |  | CADM2 |  |  |
|  |  | UVSSA |  |  |
|  |  | RPL18 |  |  |
|  |  | PHF20 |  |  |
|  |  | NEDD9 |  |  |
|  |  | GINS4 |  |  |
|  |  | B4GALT2 | |  |
|  |  | AP3M1 |  |  |
|  |  | BC035947 | |  |
|  |  | PGM2L1 |  |  |
|  |  | NRIP1 |  |  |
|  |  | SPTLC2 |  |  |
|  |  | P4HA1 |  |  |
|  |  | GM17651 | |  |
|  |  | IPO7 |  |  |
|  |  | PPHLN1 |  |  |
|  |  | EMILIN1 |  |  |
|  |  | ANKRD40 | |  |
|  |  | OGFOD3 |  |  |
|  |  | STK26 |  |  |
|  |  | NOL11 |  |  |
|  |  | DRD5 |  |  |
|  |  | SEC22C |  |  |
|  |  | VMN1R24 | |  |
|  |  | CGNL1 |  |  |
|  |  | SURF2 |  |  |
|  |  | PSMD4 |  |  |
|  |  | GM17606 | |  |
|  |  | MSANTD1 | |  |
|  |  | GDF5 |  |  |
|  |  | XPA |  |  |
|  |  | PPEF1 |  |  |
|  |  | BAG2 |  |  |
|  |  | FZD2 |  |  |
|  |  | CBLB |  |  |
|  |  | PCOLCE |  |  |
|  |  | FANCB |  |  |
|  |  | UBE2O |  |  |
|  |  | PKIB |  |  |
|  |  | AVPR1B |  |  |
|  |  | MTNR1B | |  |
|  |  | ENTPD8 |  |  |
|  |  | PRSS47 |  |  |
|  |  | OLFR1135 | |  |
|  |  | OLFR1137 | |  |
|  |  | OLFR683 |  |  |
|  |  | APOF |  |  |
|  |  | 1700080E11RIK | |  |
|  |  | DEFB37 |  |  |
|  |  | SMGC |  |  |
|  |  | HRNR |  |  |
|  |  | NKX6-2 |  |  |
|  |  | LAMA1 |  |  |
|  |  | HNRNPC | |  |
|  |  | ACOT7 |  |  |
|  |  | R3HCC1L | |  |
|  |  | FRMD5 |  |  |
|  |  | NUP62CL | |  |
|  |  | COMMD10 | |  |
|  |  | ISCA1 |  |  |
|  |  | ZFP113 |  |  |
|  |  | PSD4 |  |  |
|  |  | SLA2 |  |  |
|  |  | PCDHGA10 | |  |
|  |  | SPON2 |  |  |
|  |  | N4BP3 |  |  |
|  |  | LRRC75A | |  |
|  |  | PLXNB3 |  |  |
|  |  | SLA |  |  |
|  |  | PRAG1 |  |  |
|  |  | LY9 |  |  |
|  |  | CNTNAP2 | |  |
|  |  | SF3B5 |  |  |
|  |  | SYT1 |  |  |
|  |  | TNNI2 |  |  |
|  |  | CD96 |  |  |
|  |  | BICC1 |  |  |
|  |  | SHOX2 |  |  |
|  |  | VAX2 |  |  |
|  |  | BC030500 | |  |
|  |  | ABI3BP |  |  |
|  |  | MT-ND3 |  |  |
|  |  | MED13L |  |  |
|  |  | LRSAM1 |  |  |
|  |  | ITGAX |  |  |
|  |  | ANO5 |  |  |
|  |  | CCDC9 |  |  |
|  |  | CCDC151 | |  |
|  |  | UBE2D2B | |  |
|  |  | FGF9 |  |  |
|  |  | EIF4EBP3 | |  |
|  |  | DLX5 |  |  |
|  |  | ZFP13 |  |  |
|  |  | MTMR4 |  |  |
|  |  | AGAP3 |  |  |
|  |  | PPARA |  |  |
|  |  | IL20RB |  |  |
|  |  | INTS4 |  |  |
|  |  | MAPK8IP1 | |  |
|  |  | GMPPB |  |  |
|  |  | GLIS2 |  |  |
|  |  | CACNA1I | |  |
|  |  | XNDC1 |  |  |
|  |  | FOXRED2 | |  |
|  |  | TRIM72 |  |  |
|  |  | HEG1 |  |  |
|  |  | CBLN3 |  |  |
|  |  | B3GNT6 |  |  |
|  |  | ABCA13 |  |  |
|  |  | CXCL17 |  |  |
|  |  | DCBLD1 |  |  |
|  |  | CYB5R4 |  |  |
|  |  | PLEKHB2 | |  |
|  |  | PPP2R2D | |  |
|  |  | CDC123 |  |  |
|  |  | CCNH |  |  |
|  |  | CYP2J12 |  |  |
|  |  | ANGEL1 |  |  |
|  |  | TAS2R143 | |  |
|  |  | MYCBPAP | |  |
|  |  | CHRNB1 |  |  |
|  |  | ZDHHC16 | |  |
|  |  | NCAPG |  |  |
|  |  | FBXO36 |  |  |
|  |  | MYH6 |  |  |
|  |  | OLFR112 |  |  |
|  |  | OSBPL7 |  |  |
|  |  | CYP2B10 | |  |
|  |  | EIF4E3 |  |  |
|  |  | SLC41A3 |  |  |
|  |  | DFFA |  |  |
|  |  | DYM |  |  |
|  |  | KCNB1 |  |  |
|  |  | CAMSAP2 | |  |
|  |  | ARHGEF3 | |  |
|  |  | LIMK1 |  |  |
|  |  | STX4A |  |  |
|  |  | RAD54L2 | |  |
|  |  | MRPL18 |  |  |
|  |  | TEX9 |  |  |
|  |  | ALDH1B1 | |  |
|  |  | IRF2 |  |  |
|  |  | CD37 |  |  |
|  |  | ABCA5 |  |  |
|  |  | PLPP3 |  |  |
|  |  | KRTAP28-10 | |  |
|  |  | OLFR311 |  |  |
|  |  | BCL2A1D | |  |
|  |  | RCCD1 |  |  |
|  |  | TMEM143 | |  |
|  |  | D5ERTD579E | |  |
|  |  | NINJ2 |  |  |
|  |  | PFN2 |  |  |
|  |  | MAPK9 |  |  |
|  |  | AP2B1 |  |  |
|  |  | CRMP1 |  |  |
|  |  | BC067074 | |  |
|  |  | FATE1 |  |  |
|  |  | FAM208B | |  |
|  |  | HOOK1 |  |  |
|  |  | BCL7C |  |  |
|  |  | ABHD17C | |  |
|  |  | DUS4L |  |  |
|  |  | UFSP2 |  |  |
|  |  | TBXAS1 |  |  |
|  |  | E2F5 |  |  |
|  |  | CCDC148 | |  |
|  |  | CCL22 |  |  |
|  |  | ATP2A2 |  |  |
|  |  | C530025M09RIK | |  |
|  |  | UBE3A |  |  |
|  |  | OLFR1438-PS1 | |  |
|  |  | RFPL4 |  |  |
|  |  | KLKB1 |  |  |
|  |  | FOXI1 |  |  |
|  |  | OLFR15 |  |  |
|  |  | TFIP11 |  |  |
|  |  | LETM1 |  |  |
|  |  | SF3B1 |  |  |
|  |  | CLDND2 |  |  |
|  |  | ANK1 |  |  |
|  |  | ANK |  |  |
|  |  | MAFG |  |  |
|  |  | PLA2G10 | |  |
|  |  | P4HA3 |  |  |
|  |  | ZFP455 |  |  |
|  |  | MRPS17 |  |  |
|  |  | SNX22 |  |  |
|  |  | PPP4C |  |  |
|  |  | TPH2 |  |  |
|  |  | ETFDH |  |  |
|  |  | FBRS |  |  |
|  |  | SFSWAP |  |  |
|  |  | GIN1 |  |  |
|  |  | PQLC1 |  |  |
|  |  | CFAP157 |  |  |
|  |  | FCHSD1 |  |  |
|  |  | NT5DC1 |  |  |
|  |  | APOL7A |  |  |
|  |  | SIPA1L1 |  |  |
|  |  | ATP5O |  |  |
|  |  | RGS16 |  |  |
|  |  | CBX8 |  |  |
|  |  | TSHZ2 |  |  |
|  |  | RASIP1 |  |  |
|  |  | CCDC32 |  |  |
|  |  | WISP3 |  |  |
|  |  | IRF3 |  |  |
|  |  | FLRT1 |  |  |
|  |  | MACF1 |  |  |
|  |  | CAR3 |  |  |
|  |  | PTCHD4 |  |  |
|  |  | MRPL3 |  |  |
|  |  | ATF7IP |  |  |
|  |  | HLX |  |  |
|  |  | TMC2 |  |  |
|  |  | TMEM202 | |  |
|  |  | GRK6 |  |  |
|  |  | ARPC3 |  |  |
|  |  | VEZT |  |  |
|  |  | MYLPF |  |  |
|  |  | TOGARAM2 | |  |
|  |  | LCN2 |  |  |
|  |  | ZFP202 |  |  |
|  |  | ATP8B4 |  |  |
|  |  | C130050O18RIK | |  |
|  |  | UBA2 |  |  |
|  |  | NOL4 |  |  |
|  |  | CLVS1 |  |  |
|  |  | VPS37D |  |  |
|  |  | METTL15 | |  |
|  |  | FKBP1B |  |  |
|  |  | ZFP598 |  |  |
|  |  | PRF1 |  |  |
|  |  | TEX50 |  |  |
|  |  | 2210418O10RIK | |  |
|  |  | PKP3 |  |  |
|  |  | SLC12A3 |  |  |
|  |  | SNAP47 |  |  |
|  |  | MBTD1 |  |  |
|  |  | KCNMA1 | |  |
|  |  | RNF111 |  |  |
|  |  | RPS17 |  |  |
|  |  | COMMD3 | |  |
|  |  | 4933411K16RIK | |  |
|  |  | HRH1 |  |  |
|  |  | MTMR7 |  |  |
|  |  | CAMSAP3 | |  |
|  |  | ALDH8A1 | |  |
|  |  | APBB1IP |  |  |
|  |  | DUXBL1 |  |  |
|  |  | NIPAL2 |  |  |
|  |  | OLFR679 |  |  |
|  |  | OLFML3 |  |  |
|  |  | NUTM1 |  |  |
|  |  | INTS6L |  |  |
|  |  | GABRB2 |  |  |
|  |  | UBQLN1 |  |  |
|  |  | KCNC4 |  |  |
|  |  | RPL27A |  |  |
|  |  | AGPAT2 |  |  |
|  |  | ZFP286 |  |  |
|  |  | GM17732 | |  |
|  |  | NPC1 |  |  |
|  |  | KAT6B |  |  |
|  |  | UCK2 |  |  |
|  |  | MATN4 |  |  |
|  |  | RAB29 |  |  |
|  |  | 9530053A07RIK | |  |
|  |  | CHPF |  |  |
|  |  | CORO1C |  |  |
|  |  | ZBTB1 |  |  |
|  |  | WDR77 |  |  |
|  |  | WNT1 |  |  |
|  |  | GRID1 |  |  |
|  |  | OLFR1029 | |  |
|  |  | PJVK |  |  |
|  |  | CAMK4 |  |  |
|  |  | TECPR1 |  |  |
|  |  | MYLIP |  |  |
|  |  | CDH10 |  |  |
|  |  | MRPS9 |  |  |
|  |  | TMEM263 | |  |
|  |  | TECTB |  |  |
|  |  | UBL4A |  |  |
|  |  | GM44502 | |  |
|  |  | ODC1 |  |  |
|  |  | MCM9 |  |  |
|  |  | FIBP |  |  |
|  |  | HTRA1 |  |  |
|  |  | ANKRD31 | |  |
|  |  | LRRC73 |  |  |
|  |  | CAMTA1 |  |  |
|  |  | AGO3 |  |  |
|  |  | FTL1 |  |  |
|  |  | HEPACAM2 | |  |
|  |  | SLC11A1 |  |  |
|  |  | PDCD4 |  |  |
|  |  | ADGRA3 |  |  |
|  |  | GRIN3B |  |  |
|  |  | ADAL |  |  |
|  |  | USP34 |  |  |
|  |  | GPBP1 |  |  |
|  |  | MRPL17 |  |  |
|  |  | DBN1 |  |  |
|  |  | MTHFD1L | |  |
|  |  | SENP7 |  |  |
|  |  | UBQLN4 |  |  |
|  |  | MOSPD1 |  |  |
|  |  | KCNMB1 | |  |
|  |  | TM7SF3 |  |  |
|  |  | 1700020L24RIK | |  |
|  |  | 0610012G03RIK | |  |
|  |  | C1RA |  |  |
|  |  | PIGL |  |  |
|  |  | MFSD6 |  |  |
|  |  | GLTPD2 |  |  |
|  |  | PRM1 |  |  |
|  |  | RNASEK |  |  |
|  |  | GJA1 |  |  |
|  |  | GPR157 |  |  |
|  |  | LINGO4 |  |  |
|  |  | SIM2 |  |  |
|  |  | EIF5 |  |  |
|  |  | FOXC2 |  |  |
|  |  | CLUH |  |  |
|  |  | PPP3R2 |  |  |
|  |  | PROX2 |  |  |
|  |  | NUP50 |  |  |
|  |  | PHOSPHO2 | |  |
|  |  | CYB5R2 |  |  |
|  |  | FAM43A |  |  |
|  |  | STX1B |  |  |
|  |  | PTPDC1 |  |  |
|  |  | THRAP3 |  |  |
|  |  | ANKUB1 |  |  |
|  |  | SCML2 |  |  |
|  |  | SLC17A9 |  |  |
|  |  | CNBD2 |  |  |
|  |  | MYH7B |  |  |
|  |  | AADAT |  |  |
|  |  | UNC79 |  |  |
|  |  | NTPCR |  |  |
|  |  | TNFRSF21 | |  |
|  |  | CCDC189 | |  |
|  |  | SRGN |  |  |
|  |  | DDRGK1 |  |  |
|  |  | E2F6 |  |  |
|  |  | ZFP282 |  |  |
|  |  | DHRS7 |  |  |
|  |  | SFTPC |  |  |
|  |  | PPRC1 |  |  |
|  |  | LEPROTL1 | |  |
|  |  | GINS3 |  |  |
|  |  | TFDP2 |  |  |
|  |  | MRPS36 |  |  |
|  |  | TACC3 |  |  |
|  |  | PLEKHA6 | |  |
|  |  | LMLN |  |  |
|  |  | RPS6KA4 | |  |
|  |  | LIPO3 |  |  |
|  |  | AKR1B10 | |  |
|  |  | ARHGAP28 | |  |
|  |  | YIPF2 |  |  |
|  |  | CREG2 |  |  |
|  |  | TTC38 |  |  |
|  |  | DDX28 |  |  |
|  |  | PUM3 |  |  |
|  |  | ENPP5 |  |  |
|  |  | CROCC2 |  |  |
|  |  | ARXES1 |  |  |
|  |  | ASB13 |  |  |
|  |  | GM45844 | |  |
|  |  | SNRNP27 | |  |
|  |  | SUV39H1 | |  |
|  |  | NPRL2 |  |  |
|  |  | AP5Z1 |  |  |
|  |  | AF529169 | |  |
|  |  | TMEFF1 |  |  |
|  |  | RBM18 |  |  |
|  |  | 1700102P08RIK | |  |
|  |  | DENND6A | |  |
|  |  | SERPINE3 | |  |
|  |  | INSL6 |  |  |
|  |  | CEP192 |  |  |
|  |  | KCTD21 |  |  |
|  |  | TRMT2B |  |  |
|  |  | LAYN |  |  |
|  |  | SOSTDC1 | |  |
|  |  | LDAH |  |  |
|  |  | ZFP651 |  |  |
|  |  | TCF3 |  |  |
|  |  | HSPD1 |  |  |
|  |  | CAPN12 |  |  |
|  |  | CCNL1 |  |  |
|  |  | GRB10 |  |  |
|  |  | GSDMC4 |  |  |
|  |  | UQCRFS1 | |  |
|  |  | ARL5B |  |  |
|  |  | MRPS22 |  |  |
|  |  | ZFC3H1 |  |  |
|  |  | PATE4 |  |  |
|  |  | GM3005 |  |  |
|  |  | CLEC1B |  |  |
|  |  | ZSCAN10 | |  |
|  |  | CD209C |  |  |
|  |  | SLC17A3 |  |  |
|  |  | ARRDC5 |  |  |
|  |  | MS4A4D |  |  |
|  |  | ALOX12E | |  |
|  |  | SPDYE4B | |  |
|  |  | SH2D1B2 | |  |
|  |  | VMN1R232 | |  |
|  |  | VMN1R200 | |  |
|  |  | LRIT1 |  |  |
|  |  | PLAC8 |  |  |
|  |  | GM3500 |  |  |
|  |  | TRIM50 |  |  |
|  |  | SLC25A28 | |  |
|  |  | H3F3A |  |  |
|  |  | GNA14 |  |  |
|  |  | FOXN3 |  |  |
|  |  | GM5148 |  |  |
|  |  | INPP4A |  |  |
|  |  | NCAPG2 |  |  |
|  |  | PPP1R3B | |  |
|  |  | MAP4K5 |  |  |
|  |  | GM9767 |  |  |
|  |  | OLFR45 |  |  |
|  |  | FRMD6 |  |  |
|  |  | SNN |  |  |
|  |  | H2-T24 |  |  |
|  |  | ARL11 |  |  |
|  |  | GDAP1 |  |  |
|  |  | AI182371 |  |  |
|  |  | BRIX1 |  |  |
|  |  | BCL11B |  |  |
|  |  | ZBBX |  |  |
|  |  | ADGRF3 |  |  |
|  |  | KLHDC1 |  |  |
|  |  | UBN2 |  |  |
|  |  | FICD |  |  |
|  |  | CEP250 |  |  |
|  |  | FAM50B |  |  |
|  |  | CLEC3A |  |  |
|  |  | LDHC |  |  |
|  |  | CCR1L1 |  |  |
|  |  | OLFR857 |  |  |
|  |  | KRTDAP |  |  |
|  |  | SAA1 |  |  |
|  |  | GM33543 | |  |
|  |  | GM45521 | |  |
|  |  | OLFR1347 | |  |
|  |  | CLDN4 |  |  |
|  |  | PRKCG |  |  |
|  |  | POU5F1 |  |  |
|  |  | CD28 |  |  |
|  |  | PIGR |  |  |
|  |  | SEC24B |  |  |
|  |  | EPSTI1 |  |  |
|  |  | OLFR552 |  |  |
|  |  | A030005L19RIK | |  |
|  |  | CORO1A |  |  |
|  |  | SH3D19 |  |  |
|  |  | CAPRIN2 | |  |
|  |  | C4B |  |  |
|  |  | ALG13 |  |  |
|  |  | BRPF3 |  |  |
|  |  | KCTD20 |  |  |
|  |  | RPSA |  |  |
|  |  | EPS8L3 |  |  |
|  |  | SYF2 |  |  |
|  |  | SBDS |  |  |
|  |  | GM609 |  |  |
|  |  | SLC43A2 |  |  |
|  |  | ALG5 |  |  |
|  |  | CSNK2A1 | |  |
|  |  | UGDH |  |  |
|  |  | IGSF6 |  |  |
|  |  | MLST8 |  |  |
|  |  | METTL11B | |  |
|  |  | PCBP1 |  |  |
|  |  | DCAF12 |  |  |
|  |  | PYCR1 |  |  |
|  |  | ERP44 |  |  |
|  |  | FDXR |  |  |
|  |  | CNOT7 |  |  |
|  |  | PLSCR2 |  |  |
|  |  | SLC12A9 |  |  |
|  |  | GYG |  |  |
|  |  | MYZAP |  |  |
|  |  | C1QTNF1 | |  |
|  |  | BMP2 |  |  |
|  |  | AP2A1 |  |  |
|  |  | SGCE |  |  |
|  |  | PDIK1L |  |  |
|  |  | NSMAF |  |  |
|  |  | SPEG |  |  |
|  |  | GSX2 |  |  |
|  |  | MYBPC2 |  |  |
|  |  | MROH8 |  |  |
|  |  | NUP205 |  |  |
|  |  | SSX9 |  |  |
|  |  | NCEH1 |  |  |
|  |  | TMEM203 | |  |
|  |  | FBXW2 |  |  |
|  |  | RAB8A |  |  |
|  |  | LPAR2 |  |  |
|  |  | POLR2F |  |  |
|  |  | WDR44 |  |  |
|  |  | LTBR |  |  |
|  |  | RAB34 |  |  |
|  |  | KLB |  |  |
|  |  | SLX1B |  |  |
|  |  | MFSD3 |  |  |
|  |  | PSD |  |  |
|  |  | ZFP626 |  |  |
|  |  | ZFP868 |  |  |
|  |  | KATNBL1 | |  |
|  |  | SDCCAG8 | |  |
|  |  | KTI12 |  |  |
|  |  | MAP4K4 |  |  |
|  |  | ANAPC15-PS | |  |
|  |  | HSD3B7 |  |  |
|  |  | ENO2 |  |  |
|  |  | ZSCAN22 | |  |
|  |  | IMPAD1 |  |  |
|  |  | C2CD4D |  |  |
|  |  | GRIA2 |  |  |
|  |  | IMMP1L |  |  |
|  |  | ZFP507 |  |  |
|  |  | RNF121 |  |  |
|  |  | ILF2 |  |  |
|  |  | H2-EB1 |  |  |
|  |  | PPP2CB |  |  |
|  |  | RETREG2 | |  |
|  |  | H2-Q2 |  |  |
|  |  | UCMA |  |  |
|  |  | SLC39A5 |  |  |
|  |  | MAMDC2 | |  |
|  |  | H2AFZ |  |  |
|  |  | LRRC46 |  |  |
|  |  | LIPT2 |  |  |
|  |  | ZMYM3 |  |  |
|  |  | DEK |  |  |
|  |  | COPB1 |  |  |
|  |  | WDR33 |  |  |
|  |  | USP27X |  |  |
|  |  | TEX12 |  |  |
|  |  | BABAM2 |  |  |
|  |  | MEX3D |  |  |
|  |  | TTC23 |  |  |
|  |  | UBXN2A |  |  |
|  |  | TTC6 |  |  |
|  |  | TMF1 |  |  |
|  |  | SHMT2 |  |  |
|  |  | EPAS1 |  |  |
|  |  | CCDC33 |  |  |
|  |  | FBXL5 |  |  |
|  |  | ZFP930 |  |  |
|  |  | ZSWIM8 |  |  |
|  |  | UBR7 |  |  |
|  |  | ZFP740 |  |  |
|  |  | CDC45 |  |  |
|  |  | SEMA6C |  |  |
|  |  | 1700018G05RIK | |  |
|  |  | A930033H14RIK | |  |
|  |  | SP2 |  |  |
|  |  | ZFP688 |  |  |
|  |  | SH3BGR |  |  |
|  |  | MIIP |  |  |
|  |  | NDUFAF8 | |  |
|  |  | BBOF1 |  |  |
|  |  | UNC93B1 | |  |
|  |  | POLR1A |  |  |
|  |  | RBFOX1 |  |  |
|  |  | STARD9 |  |  |
|  |  | ATAD1 |  |  |
|  |  | CD40 |  |  |
|  |  | MLF1 |  |  |
|  |  | PPM1A |  |  |
|  |  | TRIM65 |  |  |
|  |  | CEBPZOS | |  |
|  |  | DOCK1 |  |  |
|  |  | FAM124B | |  |
|  |  | NIPAL4 |  |  |
|  |  | USP45 |  |  |
|  |  | ABCC5 |  |  |
|  |  | T |  |  |
|  |  | SPRN |  |  |
|  |  | CDC16 |  |  |
|  |  | DNAH10 |  |  |
|  |  | VMN2R84 | |  |
|  |  | HFE |  |  |
|  |  | DENND1C | |  |
|  |  | PTCRA |  |  |
|  |  | GRIFIN |  |  |
|  |  | ADAD2 |  |  |
|  |  | FASTKD1 | |  |
|  |  | RAB2B |  |  |
|  |  | F830045P16RIK | |  |
|  |  | K230010J24RIK | |  |
|  |  | SPATA6 |  |  |
|  |  | HEYL |  |  |
|  |  | MMP15 |  |  |
|  |  | TMEM26 |  |  |
|  |  | DRD4 |  |  |
|  |  | PLA2G15 | |  |
|  |  | NRAS |  |  |
|  |  | POU6F2 |  |  |
|  |  | ZFP830 |  |  |
|  |  | CRELD2 |  |  |
|  |  | STK24 |  |  |
|  |  | EBAG9 |  |  |
|  |  | DCUN1D2 | |  |
|  |  | 6030445D17RIK | |  |
|  |  | TDP2 |  |  |
|  |  | SMCR8 |  |  |
|  |  | HTR7 |  |  |
|  |  | HS6ST1 |  |  |
|  |  | NDE1 |  |  |
|  |  | PPP1R32 |  |  |
|  |  | KCNS3 |  |  |
|  |  | GTF2H4 |  |  |
|  |  | RAD1 |  |  |
|  |  | CD33 |  |  |
|  |  | MED4 |  |  |
|  |  | LAMTOR1 | |  |
|  |  | ASTN1 |  |  |
|  |  | WDR81 |  |  |
|  |  | NCAPD2 |  |  |
|  |  | PSMC6 |  |  |
|  |  | PATE3 |  |  |
|  |  | ITGB1BP2 | |  |
|  |  | GCLM |  |  |
|  |  | PLEK |  |  |
|  |  | BRINP2 |  |  |
|  |  | AARS |  |  |
|  |  | MAFK |  |  |
|  |  | NUDT3 |  |  |
|  |  | CLCN4 |  |  |
|  |  | ZFP647 |  |  |
|  |  | NKIRAS1 | |  |
|  |  | PLXNC1 |  |  |
|  |  | ZFP960 |  |  |
|  |  | GRK3 |  |  |
|  |  | ACTR8 |  |  |
|  |  | TIMMDC1 | |  |
|  |  | CHRNA4 |  |  |
|  |  | HNRNPR | |  |
|  |  | GSTM4 |  |  |
|  |  | HNRNPH3 | |  |
|  |  | MAPK8IP2 | |  |
|  |  | MCFD2 |  |  |
|  |  | TMX1 |  |  |
|  |  | BAP1 |  |  |
|  |  | PUS1 |  |  |
|  |  | HTATSF1 | |  |
|  |  | NRBP2 |  |  |
|  |  | TBC1D22A | |  |
|  |  | RHOQ |  |  |
|  |  | CTNNA2 |  |  |
|  |  | DYNC1H1 | |  |
|  |  | AKR1B8 |  |  |
|  |  | AK1 |  |  |
|  |  | CYB5B |  |  |
|  |  | NEK4 |  |  |
|  |  | SRPRB |  |  |
|  |  | RAB43 |  |  |
|  |  | YIPF1 |  |  |
|  |  | TCERG1 |  |  |
|  |  | CDH15 |  |  |
|  |  | GAL3ST3 | |  |
|  |  | GM9493 |  |  |
|  |  | TRPC3 |  |  |
|  |  | EMP1 |  |  |
|  |  | SLC26A7 |  |  |
|  |  | ERGIC1 |  |  |
|  |  | IGF2BP3 |  |  |
|  |  | RPL36A |  |  |
|  |  | NRIP3 |  |  |
|  |  | CSMD2 |  |  |
|  |  | CRLF2 |  |  |
|  |  | MS4A4A |  |  |
|  |  | GPR155 |  |  |
|  |  | PMEL |  |  |
|  |  | ART4 |  |  |
|  |  | TRIM12A | |  |
|  |  | CLBA1 |  |  |
|  |  | SLC45A1 |  |  |
|  |  | ST6GALNAC4 | |  |
|  |  | NPAS1 |  |  |
|  |  | CCDC129 | |  |
|  |  | PLEKHA3 | |  |
|  |  | PPM1F |  |  |
|  |  | GLRA2 |  |  |
|  |  | ITGB2 |  |  |
|  |  | SERPINE1 | |  |
|  |  | USP40 |  |  |
|  |  | ZFP994 |  |  |
|  |  | CCDC51 |  |  |
|  |  | SEC61A2 |  |  |
|  |  | CHRDL2 |  |  |
|  |  | ANKMY1 | |  |
|  |  | ATG13 |  |  |
|  |  | RAB3IP |  |  |
|  |  | PACRG |  |  |
|  |  | SLC16A8 |  |  |
|  |  | GM15013 | |  |
|  |  | RCN1 |  |  |
|  |  | 5930422O12RIK | |  |
|  |  | KIF18B |  |  |
|  |  | ZMIZ2 |  |  |
|  |  | IL15 |  |  |
|  |  | EPN1 |  |  |
|  |  | ZFP85 |  |  |
|  |  | DCX |  |  |
|  |  | BSCL2 |  |  |
|  |  | CDYL |  |  |
|  |  | MDP1 |  |  |
|  |  | AOAH |  |  |
|  |  | 1700001P01RIK | |  |
|  |  | THBD |  |  |
|  |  | SPINK8 |  |  |
|  |  | PPP3R1 |  |  |
|  |  | KDM1A |  |  |
|  |  | F12 |  |  |
|  |  | D3ERTD751E | |  |
|  |  | ANXA11 |  |  |
|  |  | IWS1 |  |  |
|  |  | FOXO3 |  |  |
|  |  | TCTN3 |  |  |
|  |  | SRP54B |  |  |
|  |  | VWA3B |  |  |
|  |  | NDUFB5 |  |  |
|  |  | INTS9 |  |  |
|  |  | CD200R1 |  |  |
|  |  | HSPA2 |  |  |
|  |  | NEDD4L |  |  |
|  |  | ACOXL |  |  |
|  |  | GON7 |  |  |
|  |  | MTHFD2 |  |  |
|  |  | TGFB1 |  |  |
|  |  | GUCY2E |  |  |
|  |  | DUSP13 |  |  |
|  |  | PSMA7 |  |  |
|  |  | SRRM1 |  |  |
|  |  | ATP5S |  |  |
|  |  | SRXN1 |  |  |
|  |  | CIC |  |  |
|  |  | MFSD4A |  |  |
|  |  | ALDH4A1 | |  |
|  |  | ANKRD55 | |  |
|  |  | FAM155A | |  |
|  |  | IARS2 |  |  |
|  |  | RFNG |  |  |
|  |  | RUBCNL |  |  |
|  |  | DEPDC5 |  |  |
|  |  | NTM |  |  |
|  |  | STAB2 |  |  |
|  |  | GPX1 |  |  |
|  |  | GEMIN2 |  |  |
|  |  | PRDM4 |  |  |
|  |  | ZSWIM9 |  |  |
|  |  | MAFB |  |  |
|  |  | CD248 |  |  |
|  |  | MOGAT1 | |  |
|  |  | OLFR523 |  |  |
|  |  | DNASE1L3 | |  |
|  |  | ABCF2 |  |  |
|  |  | CAMK1G | |  |
|  |  | ZC2HC1C | |  |
|  |  | RPL38 |  |  |
|  |  | TRP53RKB | |  |
|  |  | ALG11 |  |  |
|  |  | ACHE |  |  |
|  |  | UBE2E1 |  |  |
|  |  | MOSPD2 |  |  |
|  |  | HSD3B1 |  |  |
|  |  | GPR151 |  |  |
|  |  | ARG1 |  |  |
|  |  | PPM1D |  |  |
|  |  | LARP4 |  |  |
|  |  | AVL9 |  |  |
|  |  | MTUS1 |  |  |
|  |  | MDM2 |  |  |
|  |  | KPNA3 |  |  |
|  |  | GNG11 |  |  |
|  |  | RPL23 |  |  |
|  |  | TMEM11 |  |  |
|  |  | KIF1BP |  |  |
|  |  | GM11492 | |  |
|  |  | AKR1E1 |  |  |
|  |  | GM17324 | |  |
|  |  | CDC42BPB | |  |
|  |  | PLPPR3 |  |  |
|  |  | KXD1 |  |  |
|  |  | SLC12A4 |  |  |
|  |  | DET1 |  |  |
|  |  | PLBD1 |  |  |
|  |  | KCNS1 |  |  |
|  |  | TLN1 |  |  |
|  |  | IL18 |  |  |
|  |  | DLX6 |  |  |
|  |  | IQGAP1 |  |  |
|  |  | BRI3BP |  |  |
|  |  | NRSN1 |  |  |
|  |  | ARL8A |  |  |
|  |  | CTH |  |  |
|  |  | AACS |  |  |
|  |  | TMEM63A | |  |
|  |  | RAD50 |  |  |
|  |  | KCNK18 |  |  |
|  |  | MYOM2 |  |  |
|  |  | CCDC155 | |  |
|  |  | SH3BP5 |  |  |
|  |  | LRRC61 |  |  |
|  |  | TBC1D10B | |  |
|  |  | BRAF |  |  |
|  |  | EMSY |  |  |
|  |  | MLX |  |  |
|  |  | SH3GLB2 | |  |
|  |  | TCEAL6 |  |  |
|  |  | MMP9 |  |  |
|  |  | UBE2M |  |  |
|  |  | KDM7A |  |  |
|  |  | CCDC43 |  |  |
|  |  | KANSL1 |  |  |
|  |  | LONP1 |  |  |
|  |  | RBM4 |  |  |
|  |  | ARL5A |  |  |
|  |  | PPID |  |  |
|  |  | BNIPL |  |  |
|  |  | WNT5B |  |  |
|  |  | RSBN1 |  |  |
|  |  | ZFP69 |  |  |
|  |  | SLC18A2 |  |  |
|  |  | CNMD |  |  |
|  |  | TRDN |  |  |
|  |  | CRISPLD1 | |  |
|  |  | FOSB |  |  |
|  |  | DCAF7 |  |  |
|  |  | ANKZF1 |  |  |
|  |  | SERINC5 |  |  |
|  |  | 2310011J03RIK | |  |
|  |  | RASL10A | |  |
|  |  | COQ6 |  |  |
|  |  | LYPLA1 |  |  |
|  |  | GM5751 |  |  |
|  |  | LUC7L2 |  |  |
|  |  | THBS2 |  |  |
|  |  | ZDHHC9 |  |  |
|  |  | ATRAID |  |  |
|  |  | TAX1BP3 | |  |
|  |  | MUSTN1 |  |  |
|  |  | FOLR1 |  |  |
|  |  | CXCR5 |  |  |
|  |  | ERLEC1 |  |  |
|  |  | SMCO3 |  |  |
|  |  | SULF1 |  |  |
|  |  | KIF27 |  |  |
|  |  | AAED1 |  |  |
|  |  | PAQR7 |  |  |
|  |  | ANAPC13 | |  |
|  |  | YPEL3 |  |  |
|  |  | UBFD1 |  |  |
|  |  | DDX11 |  |  |
|  |  | PPP1R18 |  |  |
|  |  | NAPG |  |  |
|  |  | FCER1G |  |  |
|  |  | NCKAP1L | |  |
|  |  | A3GALT2 | |  |
|  |  | SLC1A1 |  |  |
|  |  | DGLUCY | |  |
|  |  | RGS13 |  |  |
|  |  | ACTR6 |  |  |
|  |  | DDX47 |  |  |
|  |  | ATF7 |  |  |
|  |  | PAFAH2 |  |  |
|  |  | SUMO2 |  |  |
|  |  | PDP2 |  |  |
|  |  | NCF1 |  |  |
|  |  | FANCM |  |  |
|  |  | MINPP1 |  |  |
|  |  | VPS4B |  |  |
|  |  | FADS3 |  |  |
|  |  | SP4 |  |  |
|  |  | ACCSL |  |  |
|  |  | AMPH |  |  |
|  |  | C1QBP |  |  |
|  |  | PSMC3IP | |  |
|  |  | A730009L09RIK | |  |
|  |  | FZD7 |  |  |
|  |  | MGMT |  |  |
|  |  | ANO7 |  |  |
|  |  | MAB21L3 | |  |
|  |  | POLR2C |  |  |
|  |  | CCDC92B | |  |
|  |  | PUM2 |  |  |
|  |  | LRP1B |  |  |
|  |  | POLR3A |  |  |
|  |  | VSIG8 |  |  |
|  |  | PRDM6 |  |  |
|  |  | PREX2 |  |  |
|  |  | VASP |  |  |
|  |  | DMRTB1 | |  |
|  |  | BDKRB1 |  |  |
|  |  | SCIMP |  |  |
|  |  | PSMB11 |  |  |
|  |  | OLFR457 |  |  |
|  |  | NCK2 |  |  |
|  |  | GM42957 | |  |
|  |  | TOP1 |  |  |
|  |  | DTD2 |  |  |
|  |  | PDK2 |  |  |
|  |  | DYNC1I1 | |  |
|  |  | SNUPN |  |  |
|  |  | DOK1 |  |  |
|  |  | ZFP114 |  |  |
|  |  | A2ML1 |  |  |
|  |  | ACER2 |  |  |
|  |  | DNASE1 |  |  |
|  |  | RAB39B |  |  |
|  |  | NYNRIN |  |  |
|  |  | CASC4 |  |  |
|  |  | FGF1 |  |  |
|  |  | TRIP10 |  |  |
|  |  | SLC15A2 |  |  |
|  |  | DGKZ |  |  |
|  |  | UBP1 |  |  |
|  |  | CHPF2 |  |  |
|  |  | BTG3 |  |  |
|  |  | CYP2D26 | |  |
|  |  | STOML1 |  |  |
|  |  | PCDHB22 | |  |
|  |  | TEX264 |  |  |
|  |  | NDUFB10 | |  |
|  |  | LPCAT2 |  |  |
|  |  | ADAMTS19 | |  |
|  |  | NRL |  |  |
|  |  | YKT6 |  |  |
|  |  | REXO5 |  |  |
|  |  | SNX27 |  |  |
|  |  | ZDHHC18 | |  |
|  |  | CCER1 |  |  |
|  |  | SHISA8 |  |  |
|  |  | HDAC10 |  |  |
|  |  | HMGCS1 |  |  |
|  |  | TPD52 |  |  |
|  |  | HAS3 |  |  |
|  |  | EXOC8 |  |  |
|  |  | GSTM7 |  |  |
|  |  | H2-DMB2 | |  |
|  |  | HSD17B14 | |  |
|  |  | GM20379 | |  |
|  |  | ARMC4 |  |  |
|  |  | AAAS |  |  |
|  |  | PANK1 |  |  |
|  |  | PAQR3 |  |  |
|  |  | GM3604 |  |  |
|  |  | RSPH10B | |  |
|  |  | CREBL2 |  |  |
|  |  | SYNCRIP | |  |
|  |  | NUAK1 |  |  |
|  |  | HEPACAM | |  |
|  |  | TBX6 |  |  |
|  |  | EIF3F |  |  |
|  |  | SOX6 |  |  |
|  |  | TMEM191C | |  |
|  |  | ACP1 |  |  |
|  |  | N4BP1 |  |  |
|  |  | YBX3 |  |  |
|  |  | DHRS7B |  |  |
|  |  | ELAVL2 |  |  |
|  |  | MTO1 |  |  |
|  |  | EPB41 |  |  |
|  |  | PLIN1 |  |  |
|  |  | PSCA |  |  |
|  |  | GM7271 |  |  |
|  |  | SMCO1 |  |  |
|  |  | TAS2R113 | |  |
|  |  | TMEM211 | |  |
|  |  | EPHB2 |  |  |
|  |  | MED28 |  |  |
|  |  | PDE6H |  |  |
|  |  | NADK |  |  |
|  |  | TMEM273 | |  |
|  |  | HDAC8 |  |  |
|  |  | ZFP850 |  |  |
|  |  | ARL8B |  |  |
|  |  | HSPA12B | |  |
|  |  | SNTN |  |  |
|  |  | VPS36 |  |  |
|  |  | TSPAN31 | |  |
|  |  | ARGLU1 |  |  |
|  |  | PLCB2 |  |  |
|  |  | TSPAN33 | |  |
|  |  | AU041133 | |  |
|  |  | GLO1 |  |  |
|  |  | COQ4 |  |  |
|  |  | TMC3 |  |  |
|  |  | GLT1D1 |  |  |
|  |  | DENND3 |  |  |
|  |  | ALK |  |  |
|  |  | IL22RA1 |  |  |
|  |  | FGF16 |  |  |
|  |  | DYNLL2 |  |  |
|  |  | SIL1 |  |  |
|  |  | DSCAM |  |  |
|  |  | LSM6 |  |  |
|  |  | GPC1 |  |  |
|  |  | IGF2 |  |  |
|  |  | RPF2 |  |  |
|  |  | MTERF1B | |  |
|  |  | UNK |  |  |
|  |  | NDUFA7 |  |  |
|  |  | CALN1 |  |  |
|  |  | TRIM3 |  |  |
|  |  | CFAP65 |  |  |
|  |  | GM17622 | |  |
|  |  | NHLH1 |  |  |
|  |  | ZFP87 |  |  |
|  |  | SPAG6 |  |  |
|  |  | GNAQ |  |  |
|  |  | FAM173A | |  |
|  |  | IFT74 |  |  |
|  |  | MED18 |  |  |
|  |  | PDHX |  |  |
|  |  | ARMCX3 | |  |
|  |  | LRRC8C |  |  |
|  |  | LSM2 |  |  |
|  |  | BCAS2 |  |  |
|  |  | U2AF2 |  |  |
|  |  | RTL9 |  |  |
|  |  | PCSK1 |  |  |
|  |  | PYCR2 |  |  |
|  |  | SMARCC2 | |  |
|  |  | EARS2 |  |  |
|  |  | SSBP4 |  |  |
|  |  | CACNA2D1 | |  |
|  |  | MMP28 |  |  |
|  |  | EFCAB9 |  |  |
|  |  | RFC1 |  |  |
|  |  | SMARCAL1 | |  |
|  |  | MYO1H |  |  |
|  |  | PTN |  |  |
|  |  | INSM1 |  |  |
|  |  | SUCLA2 |  |  |
|  |  | LRRC28 |  |  |
|  |  | EIF3C |  |  |
|  |  | TMEM238 | |  |
|  |  | PSTPIP1 |  |  |
|  |  | NLRX1 |  |  |
|  |  | BRAT1 |  |  |
|  |  | ALAS1 |  |  |
|  |  | TSEN15 |  |  |
|  |  | 9530077C05RIK | |  |
|  |  | ECSCR |  |  |
|  |  | PKM |  |  |
|  |  | CACNA1D | |  |
|  |  | ARHGEF4 | |  |
|  |  | HIST1H3B | |  |
|  |  | STUB1 |  |  |
|  |  | DHODH |  |  |
|  |  | DHX34 |  |  |
|  |  | CPSF3 |  |  |
|  |  | SHLD1 |  |  |
|  |  | EPRS |  |  |
|  |  | NDC80 |  |  |
|  |  | MYO9A |  |  |
|  |  | CFP |  |  |
|  |  | TMBIM7 |  |  |
|  |  | PRDX4 |  |  |
|  |  | PALM |  |  |
|  |  | CEP152 |  |  |
|  |  | SLC39A11 | |  |
|  |  | ARMCX4 | |  |
|  |  | CDC25B |  |  |
|  |  | TNFRSF23 | |  |
|  |  | NDUFA4L2 | |  |
|  |  | 2410015M20RIK | |  |
|  |  | ATP7B |  |  |
|  |  | ABHD8 |  |  |
|  |  | FLOT2 |  |  |
|  |  | CCDC88A | |  |
|  |  | PATL2 |  |  |
|  |  | MATN1 |  |  |
|  |  | KHDC3 |  |  |
|  |  | OLFR656 |  |  |
|  |  | GM3285 |  |  |
|  |  | ADAP2 |  |  |
|  |  | MYO1C |  |  |
|  |  | IGSF21 |  |  |
|  |  | LIPT1 |  |  |
|  |  | AURKA |  |  |
|  |  | RPL22 |  |  |
|  |  | ZFP879 |  |  |
|  |  | SARM1 |  |  |
|  |  | PRSS35 |  |  |
|  |  | C1QTNF12 | |  |
|  |  | IGSF8 |  |  |
|  |  | NUAK2 |  |  |
|  |  | VSIG10L |  |  |
|  |  | CHML |  |  |
|  |  | FAM96A |  |  |
|  |  | RNF152 |  |  |
|  |  | SP3 |  |  |
|  |  | TARSL2 |  |  |
|  |  | TMEM67 |  |  |
|  |  | MAPKBP1 | |  |
|  |  | HIST1H2AN | |  |
|  |  | NLN |  |  |
|  |  | ERICH6 |  |  |
|  |  | SUPT16 |  |  |
|  |  | GM36368 | |  |
|  |  | ZMAT1 |  |  |
|  |  | IFNGR2 |  |  |
|  |  | GOT2 |  |  |
|  |  | OVOL3 |  |  |
|  |  | TMEM253 | |  |
|  |  | SAMD15 |  |  |
|  |  | PHYHD1 |  |  |
|  |  | LIPG |  |  |
|  |  | AK7 |  |  |
|  |  | ADGRF4 |  |  |
|  |  | VAV3 |  |  |
|  |  | DZIP3 |  |  |
|  |  | HIST1H2BJ | |  |
|  |  | AMACR |  |  |
|  |  | CDS1 |  |  |
|  |  | KHSRP |  |  |
|  |  | CTDSPL |  |  |
|  |  | AHCYL1 |  |  |
|  |  | CHADL |  |  |
|  |  | PPP6R3 |  |  |
|  |  | EML1 |  |  |
|  |  | TDRKH |  |  |
|  |  | TIMM23 |  |  |
|  |  | SHC4 |  |  |
|  |  | NDOR1 |  |  |
|  |  | TOR2A |  |  |
|  |  | CYP27A1 | |  |
|  |  | STPG4 |  |  |
|  |  | EIF4ENIF1 | |  |
|  |  | GM17669 | |  |
|  |  | CPQ |  |  |
|  |  | DUT |  |  |
|  |  | RAD17 |  |  |
|  |  | TMEM158 | |  |
|  |  | FLCN |  |  |
|  |  | LY75 |  |  |
|  |  | PPP1R42 |  |  |
|  |  | PARP2 |  |  |
|  |  | PLAUR |  |  |
|  |  | PRRX1 |  |  |
|  |  | PRDM16 |  |  |
|  |  | ZFP788 |  |  |
|  |  | DTNA |  |  |
|  |  | TAF1D |  |  |
|  |  | PLCD4 |  |  |
|  |  | ZFP949 |  |  |
|  |  | ANGPTL3 | |  |
|  |  | GM43064 | |  |
|  |  | PI4KB |  |  |
|  |  | CENPH |  |  |
|  |  | KCND2 |  |  |
|  |  | ARSA |  |  |
|  |  | AIFM3 |  |  |
|  |  | YOD1 |  |  |
|  |  | FAM219B | |  |
|  |  | TBCA |  |  |
|  |  | HSPB1 |  |  |
|  |  | 9330159F19RIK | |  |
|  |  | TBC1D9 |  |  |
|  |  | GM21954 | |  |
|  |  | CACNA2D4 | |  |
|  |  | ZFP672 |  |  |
|  |  | BAHCC1 |  |  |
|  |  | GRIP2 |  |  |
|  |  | BCL2A1A | |  |
|  |  | GM4553 |  |  |
|  |  | PHC1 |  |  |
|  |  | CEP41 |  |  |
|  |  | 1110012L19RIK | |  |
|  |  | PAPOLG |  |  |
|  |  | TACO1 |  |  |
|  |  | E2F4 |  |  |
|  |  | ALDOART1 | |  |
|  |  | ACOX1 |  |  |
|  |  | SULT1A1 | |  |
|  |  | TNFRSF11A | |  |
|  |  | ANTXR1 |  |  |
|  |  | GM7347 |  |  |
|  |  | PPM1H |  |  |
|  |  | ZFP119B |  |  |
|  |  | COX7B |  |  |
|  |  | GM12355 | |  |
|  |  | PCDH12 |  |  |
|  |  | TSKU |  |  |
|  |  | ACSL4 |  |  |
|  |  | MFSD4B2 | |  |
|  |  | ADD3 |  |  |
|  |  | ZFP110 |  |  |
|  |  | BMT2 |  |  |
|  |  | SPTAN1 |  |  |
|  |  | MIGA1 |  |  |
|  |  | NEFH |  |  |
|  |  | ABTB2 |  |  |
|  |  | SPAST |  |  |
|  |  | CKS1B |  |  |
|  |  | GM19935 | |  |
|  |  | MROH1 |  |  |
|  |  | ADGRG3 |  |  |
|  |  | KEAP1 |  |  |
|  |  | ZXDC |  |  |
|  |  | SERAC1 |  |  |
|  |  | HSDL2 |  |  |
|  |  | E130308A19RIK | |  |
|  |  | CD101 |  |  |
|  |  | INTS6 |  |  |
|  |  | MICAL3 |  |  |
|  |  | PIK3C2B |  |  |
|  |  | TARBP2 |  |  |
|  |  | CLHC1 |  |  |
|  |  | AI854703 |  |  |
|  |  | NET1 |  |  |
|  |  | CCDC187 | |  |
|  |  | GM10282 | |  |
|  |  | ACTRT3 |  |  |
|  |  | WDR5B |  |  |
|  |  | MCAT |  |  |
|  |  | RRAGC |  |  |
|  |  | TOP1MT |  |  |
|  |  | FEN1 |  |  |
|  |  | DMAC1 |  |  |
|  |  | FSIP1 |  |  |
|  |  | 1700088E04RIK | |  |
|  |  | SCYL1 |  |  |
|  |  | AGA |  |  |
|  |  | ARHGEF38 | |  |
|  |  | ZCCHC13 | |  |
|  |  | ZFP599 |  |  |
|  |  | RHOD |  |  |
|  |  | SPAAR |  |  |
|  |  | RXRA |  |  |
|  |  | PRSS48 |  |  |
|  |  | RAB37 |  |  |
|  |  | FRA10AC1 | |  |
|  |  | SEMA4G |  |  |
|  |  | USP7 |  |  |
|  |  | PPP1R27 |  |  |
|  |  | PABPC1L | |  |
|  |  | POLR2I |  |  |
|  |  | CCDC171 | |  |
|  |  | RBPJL |  |  |
|  |  | PHLPP1 |  |  |
|  |  | MZF1 |  |  |
|  |  | ODR4 |  |  |
|  |  | BOLA1 |  |  |
|  |  | APBA1 |  |  |
|  |  | SSU72 |  |  |
|  |  | KIRREL |  |  |
|  |  | HRH2 |  |  |
|  |  | ATP23 |  |  |
|  |  | PRDM15 |  |  |
|  |  | GFOD1 |  |  |
|  |  | SHANK3 |  |  |
|  |  | ABCB1B |  |  |
|  |  | SPATA7 |  |  |
|  |  | GABBR1 |  |  |
|  |  | GM28710 | |  |
|  |  | RPL39 |  |  |
|  |  | MFSD4B4 | |  |
|  |  | MAGED2 | |  |
|  |  | DDX50 |  |  |
|  |  | PTGR1 |  |  |
|  |  | CDCA3 |  |  |
|  |  | POU3F4 |  |  |
|  |  | SSTR2 |  |  |
|  |  | TUBGCP2 | |  |
|  |  | CYYR1 |  |  |
|  |  | VEGFC |  |  |
|  |  | CASC3 |  |  |
|  |  | CD59B |  |  |
|  |  | CNEP1R1 | |  |
|  |  | RGSL1 |  |  |
|  |  | CEP72 |  |  |
|  |  | SYNA |  |  |
|  |  | RPN1 |  |  |
|  |  | DLL1 |  |  |
|  |  | TIAL1 |  |  |
|  |  | FAM3C |  |  |
|  |  | NEDD1 |  |  |
|  |  | LRP6 |  |  |
|  |  | PLA2G4A | |  |
|  |  | QPCT |  |  |
|  |  | ZFR |  |  |
|  |  | GFY |  |  |
|  |  | TSPAN9 |  |  |
|  |  | CWH43 |  |  |
|  |  | ABRAXAS2 | |  |
|  |  | LRRN3 |  |  |
|  |  | NRDE2 |  |  |
|  |  | IPO13 |  |  |
|  |  | RPS6KA3 | |  |
|  |  | CLDN15 |  |  |
|  |  | ZFP229 |  |  |
|  |  | SPTSSA |  |  |
|  |  | DCBLD2 |  |  |
|  |  | TAF6L |  |  |
|  |  | KLHL28 |  |  |
|  |  | REEP5 |  |  |
|  |  | KCTD15 |  |  |
|  |  | GM45861 | |  |
|  |  | TEDDM2 | |  |
|  |  | PYGB |  |  |
|  |  | TWIST1 |  |  |
|  |  | RPAP3 |  |  |
|  |  | PGGT1B |  |  |
|  |  | JMY |  |  |
|  |  | OSTM1 |  |  |
|  |  | DRC3 |  |  |
|  |  | NMNAT3 | |  |
|  |  | SLC38A6 |  |  |
|  |  | PTK6 |  |  |
|  |  | RAPGEF3 | |  |
|  |  | CLIC1 |  |  |
|  |  | CCDC78 |  |  |
|  |  | NEMF |  |  |
|  |  | VWA7 |  |  |
|  |  | DEDD2 |  |  |
|  |  | GFM2 |  |  |
|  |  | SON |  |  |
|  |  | PPP1R16A | |  |
|  |  | PLPP4 |  |  |
|  |  | RALGAPB | |  |
|  |  | RASSF10 |  |  |
|  |  | SPRED2 |  |  |
|  |  | HIRIP3 |  |  |
|  |  | TMEM59L | |  |
|  |  | GIMAP1 |  |  |
|  |  | NEK5 |  |  |
|  |  | SRPK1 |  |  |
|  |  | D6WSU163E | |  |
|  |  | ART1 |  |  |
|  |  | SOAT2 |  |  |
|  |  | S100A11 |  |  |
|  |  | GM8994 |  |  |
|  |  | SCIN |  |  |
|  |  | SLC25A48 | |  |
|  |  | COQ8A |  |  |
|  |  | CLCN5 |  |  |
|  |  | FREM1 |  |  |
|  |  | HID1 |  |  |
|  |  | CFAP44 |  |  |
|  |  | OAS2 |  |  |
|  |  | MYO7B |  |  |
|  |  | EFR3A |  |  |
|  |  | GNB4 |  |  |
|  |  | ADRA1D |  |  |
|  |  | CDH12 |  |  |
|  |  | FAM111A | |  |
|  |  | INO80D |  |  |
|  |  | PSMD6 |  |  |
|  |  | VAT1 |  |  |
|  |  | PARS2 |  |  |
|  |  | FUS |  |  |
|  |  | PTGES |  |  |
|  |  | FAM13A |  |  |
|  |  | GREB1 |  |  |
|  |  | YWHAB |  |  |
|  |  | PSMB3 |  |  |
|  |  | NRCAM |  |  |
|  |  | FAM189A2 | |  |
|  |  | SRD5A3 |  |  |
|  |  | ZSCAN18 | |  |
|  |  | ZFP64 |  |  |
|  |  | APOLD1 |  |  |
|  |  | SLC25A5 |  |  |
|  |  | MAP4K1 |  |  |
|  |  | GRRP1 |  |  |
|  |  | BSPRY |  |  |
|  |  | IPO5 |  |  |
|  |  | HLCS |  |  |
|  |  | GGT1 |  |  |
|  |  | LNX2 |  |  |
|  |  | ILVBL |  |  |
|  |  | MTHFSD | |  |
|  |  | TOP2A |  |  |
|  |  | FREM2 |  |  |
|  |  | CNPPD1 |  |  |
|  |  | ALYREF |  |  |
|  |  | PAPOLA |  |  |
|  |  | AFMID |  |  |
|  |  | TMEM14A | |  |
|  |  | UBALD1 |  |  |
|  |  | LRRFIP2 |  |  |
|  |  | SH3PXD2A | |  |
|  |  | TIMM9 |  |  |
|  |  | ACAT1 |  |  |
|  |  | SPINK10 |  |  |
|  |  | CEBPD |  |  |
|  |  | ZFP397 |  |  |
|  |  | BFAR |  |  |
|  |  | TMEM65 |  |  |
|  |  | CCDC183 | |  |
|  |  | GGCT |  |  |
|  |  | SOX1 |  |  |
|  |  | NARF |  |  |
|  |  | ITM2C |  |  |
|  |  | CCDC122 | |  |
|  |  | DLST |  |  |
|  |  | PPP6R2 |  |  |
|  |  | GM11042 | |  |
|  |  | IRAK3 |  |  |
|  |  | YARS |  |  |
|  |  | CCL9 |  |  |
|  |  | SMIM12 |  |  |
|  |  | GM6899 |  |  |
|  |  | ISX |  |  |
|  |  | 4833427G06RIK | |  |
|  |  | 1810046K07RIK | |  |
|  |  | TLDC2 |  |  |
|  |  | GM3417 |  |  |
|  |  | UGT1A2 |  |  |
|  |  | UTP23 |  |  |
|  |  | NSUN5 |  |  |
|  |  | GTF3C1 |  |  |
|  |  | TBL1X |  |  |
|  |  | NARFL |  |  |
|  |  | RORA |  |  |
|  |  | SH3BGRL2 | |  |
|  |  | TOB2 |  |  |
|  |  | FAM49B |  |  |
|  |  | ABHD4 |  |  |
|  |  | HIF3A |  |  |
|  |  | FUCA2 |  |  |
|  |  | ATP6V1B2 | |  |
|  |  | RPL13 |  |  |
|  |  | DLAT |  |  |
|  |  | LYSMD4 |  |  |
|  |  | IGSF10 |  |  |
|  |  | ANAPC15 | |  |
|  |  | EPP13 |  |  |
|  |  | MYOCOS | |  |
|  |  | GM21798 | |  |
|  |  | MEGF11 |  |  |
|  |  | 4933416C03RIK | |  |
|  |  | UTP15 |  |  |
|  |  | MAPKAPK5 | |  |
|  |  | ST8SIA5 |  |  |
|  |  | MPP3 |  |  |
|  |  | STXBP1 |  |  |
|  |  | GM21671 | |  |
|  |  | PAK1IP1 |  |  |
|  |  | MED26 |  |  |
|  |  | NUP85 |  |  |
|  |  | PIAS1 |  |  |
|  |  | ASXL3 |  |  |
|  |  | HELB |  |  |
|  |  | LST1 |  |  |
|  |  | KLHL40 |  |  |
|  |  | DGKB |  |  |
|  |  | PPP1R21 |  |  |
|  |  | CEP170 |  |  |
|  |  | BTLA |  |  |
|  |  | D330045A20RIK | |  |
|  |  | MFSD1 |  |  |
|  |  | MAF |  |  |
|  |  | ZFP81 |  |  |
|  |  | GM16485 | |  |
|  |  | CES2G |  |  |
|  |  | ASB4 |  |  |
|  |  | 4930507D05RIK | |  |
|  |  | MAPK1 |  |  |
|  |  | SCAMP4 |  |  |
|  |  | GM20604 | |  |
|  |  | FNTA |  |  |
|  |  | NMT2 |  |  |
|  |  | BTBD17 |  |  |
|  |  | ISOC1 |  |  |
|  |  | CCT4 |  |  |
|  |  | MOB2 |  |  |
|  |  | KIF4 |  |  |
|  |  | NPAS2 |  |  |
|  |  | TRP53RKA | |  |
|  |  | SETD4 |  |  |
|  |  | DZIP1L |  |  |
|  |  | PROZ |  |  |
|  |  | KLHL11 |  |  |
|  |  | STAU2 |  |  |
|  |  | BASP1 |  |  |
|  |  | GM14326 | |  |
|  |  | PODN |  |  |
|  |  | ARSB |  |  |
|  |  | GCNT7 |  |  |
|  |  | SLC26A2 |  |  |
|  |  | HIST1H4H | |  |
|  |  | METTL27 | |  |
|  |  | IQSEC1 |  |  |
|  |  | SCAF4 |  |  |
|  |  | SCFD1 |  |  |
|  |  | TMC1 |  |  |
|  |  | 1700034J05RIK | |  |
|  |  | ABCA14 |  |  |
|  |  | MTNR1A | |  |
|  |  | OLFR153 |  |  |
|  |  | OLFR191 |  |  |
|  |  | H2-M1 |  |  |
|  |  | RAB17 |  |  |
|  |  | 4933415F23RIK | |  |
|  |  | UCN3 |  |  |
|  |  | 1700001F09RIK | |  |
|  |  | OLFR1459 | |  |
|  |  | CCKAR |  |  |
|  |  | TAT |  |  |
|  |  | TPRG |  |  |
|  |  | FBXW26 |  |  |
|  |  | CD79B |  |  |
|  |  | CELA2A |  |  |
|  |  | SPATA22 | |  |
|  |  | 1600015I10RIK | |  |
|  |  | GJB6 |  |  |
|  |  | WAPL |  |  |
|  |  | ZZEF1 |  |  |
|  |  | SF3B3 |  |  |
|  |  | LIPA |  |  |
|  |  | MUM1L1 | |  |
|  |  | ZFP804B |  |  |
|  |  | ABI2 |  |  |
|  |  | UGGT1 |  |  |
|  |  | ZPR1 |  |  |
|  |  | HS3ST3A1 | |  |
|  |  | DDX5 |  |  |
|  |  | FAP |  |  |
|  |  | LSM14A |  |  |
|  |  | KCNH3 |  |  |
|  |  | TNFAIP3 |  |  |
|  |  | KRT26 |  |  |
|  |  | PTEN |  |  |
|  |  | GIMAP8 |  |  |
|  |  | SPSB3 |  |  |
|  |  | ANO1 |  |  |
|  |  | IL13 |  |  |
|  |  | RBM44 |  |  |
|  |  | SNAPC4 |  |  |
|  |  | ARL6IP6 |  |  |
|  |  | TDRP |  |  |
|  |  | SLC10A4 |  |  |
|  |  | KIFAP3 |  |  |
|  |  | KYNU |  |  |
|  |  | EXOSC2 |  |  |
|  |  | RIPPLY3 |  |  |
|  |  | ZFP46 |  |  |
|  |  | CCZ1 |  |  |
|  |  | DICER1 |  |  |
|  |  | LEFTY2 |  |  |
|  |  | GM5127 |  |  |
|  |  | ZFP709 |  |  |
|  |  | FAM196A | |  |
|  |  | JUP |  |  |
|  |  | CCNB1 |  |  |
|  |  | 9-Mar |  |  |
|  |  | CDR2L |  |  |
|  |  | ANKRD44 | |  |
|  |  | MGST1 |  |  |
|  |  | JOSD1 |  |  |
|  |  | SLITRK2 |  |  |
|  |  | BOLA3 |  |  |
|  |  | 1700007K13RIK | |  |
|  |  | USP2 |  |  |
|  |  | TCF24 |  |  |
|  |  | SSRP1 |  |  |
|  |  | ODF3L1 |  |  |
|  |  | INTS1 |  |  |
|  |  | PPP1R12B | |  |
|  |  | SYTL1 |  |  |
|  |  | RDH13 |  |  |
|  |  | SPTBN4 |  |  |
|  |  | CD2BP2 |  |  |
|  |  | GPR171 |  |  |
|  |  | ACTB |  |  |
|  |  | SARNP |  |  |
|  |  | SPNS2 |  |  |
|  |  | SLC11A2 |  |  |
|  |  | CHAF1B |  |  |
|  |  | GJB1 |  |  |
|  |  | RBMX2 |  |  |
|  |  | ALDH16A1 | |  |
|  |  | PTCD2 |  |  |
|  |  | MTIF3 |  |  |
|  |  | TCP11X2 | |  |
|  |  | GAL3ST2 | |  |
|  |  | MIXL1 |  |  |
|  |  | LRRC52 |  |  |
|  |  | IFITM6 |  |  |
|  |  | OLFR1510 | |  |
|  |  | HIST1H2AP | |  |
|  |  | CALHM1 |  |  |
|  |  | OLFR466 |  |  |
|  |  | KLRA8 |  |  |
|  |  | GUCA2B |  |  |
|  |  | GM8050 |  |  |
|  |  | USP26 |  |  |
|  |  | OLFR651 |  |  |
|  |  | OLFR1351 | |  |
|  |  | SLC22A22 | |  |
|  |  | TNF |  |  |
|  |  | CYP2D11 | |  |
|  |  | CLDN34D | |  |
|  |  | MMP27 |  |  |
|  |  | WAP |  |  |
|  |  | NPY4R |  |  |
|  |  | OLFR16 |  |  |
|  |  | UGT1A1 |  |  |
|  |  | CLEC12B | |  |
|  |  | SUMF2 |  |  |
|  |  | FBXO27 |  |  |
|  |  | VMN1R28 | |  |
|  |  | FBXO24 |  |  |
|  |  | HNRNPH1 | |  |
|  |  | PICK1 |  |  |
|  |  | ABCA7 |  |  |
|  |  | ANXA6 |  |  |
|  |  | RTL5 |  |  |
|  |  | HEPH |  |  |
|  |  | NKAPL |  |  |
|  |  | CNN1 |  |  |
|  |  | UBL3 |  |  |
|  |  | MFSD13B | |  |
|  |  | B3GNTL1 | |  |
|  |  | ADORA1 |  |  |
|  |  | ZFP592 |  |  |
|  |  | MANBAL | |  |
|  |  | CANX |  |  |
|  |  | MAN2B2 |  |  |
|  |  | SH3GL3 |  |  |
|  |  | WNT9B |  |  |
|  |  | IGFN1 |  |  |
|  |  | P2RY6 |  |  |
|  |  | ELMOD3 |  |  |
|  |  | ZBTB5 |  |  |
|  |  | NEURL1A | |  |
|  |  | PSMF1 |  |  |
|  |  | ST8SIA1 |  |  |
|  |  | GM10382 | |  |
|  |  | RAB14 |  |  |
|  |  | ANKRD12 | |  |
|  |  | ZMPSTE24 | |  |
|  |  | EIF1A |  |  |
|  |  | EFNA2 |  |  |
|  |  | DUSP23 |  |  |
|  |  | ANXA5 |  |  |
|  |  | CEACAM3 | |  |
|  |  | ATF7IP2 |  |  |
|  |  | SLC30A10 | |  |
|  |  | ALOX12 |  |  |
|  |  | PGBD5 |  |  |
|  |  | NDUFAF1 | |  |
|  |  | TMOD4 |  |  |
|  |  | KLRD1 |  |  |
|  |  | PNLIPRP2 | |  |
|  |  | RHBDF1 |  |  |
|  |  | CEP57L1 |  |  |
|  |  | SLAIN2 |  |  |
|  |  | 1810022K09RIK | |  |
|  |  | ASPN |  |  |
|  |  | NIPA2 |  |  |
|  |  | GATB |  |  |
|  |  | BTBD16 |  |  |
|  |  | RAMP3 |  |  |
|  |  | RTRAF |  |  |
|  |  | MED12 |  |  |
|  |  | BMP1 |  |  |
|  |  | DCAF11 |  |  |
|  |  | RPTOR |  |  |
|  |  | CFAP46 |  |  |
|  |  | HSPA9 |  |  |
|  |  | PSMD13 |  |  |
|  |  | LYRM7 |  |  |
|  |  | TUSC2 |  |  |
|  |  | FRMD8 |  |  |
|  |  | SOST |  |  |
|  |  | SCAF11 |  |  |
|  |  | ABR |  |  |
|  |  | TM4SF1 |  |  |
|  |  | ZBTB18 |  |  |
|  |  | GM47841 | |  |
|  |  | GM1110 |  |  |
|  |  | ANKEF1 |  |  |
|  |  | METTL14 | |  |
|  |  | SMOC2 |  |  |
|  |  | SSFA2 |  |  |
|  |  | RBM22 |  |  |
|  |  | GINS2 |  |  |
|  |  | ESYT2 |  |  |
|  |  | MARK4 |  |  |
|  |  | CUL4B |  |  |
|  |  | ATG9A |  |  |
|  |  | PAPSS2 |  |  |
|  |  | 4931428F04RIK | |  |
|  |  | WSCD1 |  |  |
|  |  | ADGRA2 |  |  |
|  |  | B4GALT5 | |  |
|  |  | C1GALT1 | |  |
|  |  | SNX11 |  |  |
|  |  | ARRB1 |  |  |
|  |  | TMED8 |  |  |
|  |  | RAB28 |  |  |
|  |  | SSBP1 |  |  |
|  |  | 4931440F15RIK | |  |
|  |  | PASK |  |  |
|  |  | TOM1L2 |  |  |
|  |  | EIF2B2 |  |  |
|  |  | GRTP1 |  |  |
|  |  | PIM2 |  |  |
|  |  | THUMPD1 | |  |
|  |  | WNT2B |  |  |
|  |  | UROS |  |  |
|  |  | ARL13A |  |  |
|  |  | TIPIN |  |  |
|  |  | DDX1 |  |  |
|  |  | F8A |  |  |
|  |  | AEN |  |  |
|  |  | KLF5 |  |  |
|  |  | SHISA4 |  |  |
|  |  | ST7L |  |  |
|  |  | TAF10 |  |  |
|  |  | NUP188 |  |  |
|  |  | PLCG1 |  |  |
|  |  | COQ3 |  |  |
|  |  | NUDT5 |  |  |
|  |  | EMC2 |  |  |
|  |  | PHF14 |  |  |
|  |  | KIF22 |  |  |
|  |  | PRPH2 |  |  |
|  |  | KCNAB1 |  |  |
|  |  | COASY |  |  |
|  |  | GMEB2 |  |  |
|  |  | BBOX1 |  |  |
|  |  | 1110008P14RIK | |  |
|  |  | MRPS11 |  |  |
|  |  | HDDC3 |  |  |
|  |  | ZFP296 |  |  |
|  |  | AGRN |  |  |
|  |  | SPOCK2 |  |  |
|  |  | D630036H23RIK | |  |
|  |  | CT030732.1 | |  |
|  |  | TMEM79 |  |  |
|  |  | CELA1 |  |  |
|  |  | SSSCA1 |  |  |
|  |  | GM21969 | |  |
|  |  | HACL1 |  |  |
|  |  | NME4 |  |  |
|  |  | PSMD3 |  |  |
|  |  | LRRC8A |  |  |
|  |  | RIC8B |  |  |
|  |  | GPR146 |  |  |
|  |  | RFLNB |  |  |
|  |  | POU2F3 |  |  |
|  |  | EXOC5 |  |  |
|  |  | JTB |  |  |
|  |  | SPAG8 |  |  |
|  |  | TMEM35B | |  |
|  |  | RAB40B |  |  |
|  |  | NABP1 |  |  |
|  |  | IFITM2 |  |  |
|  |  | PCYT1A |  |  |
|  |  | FBXO22 |  |  |
|  |  | TSPAN17 | |  |
|  |  | ALG14 |  |  |
|  |  | CHCHD2 |  |  |
|  |  | ZFP457 |  |  |
|  |  | CAMP |  |  |
|  |  | ERLIN1 |  |  |
|  |  | LAIR1 |  |  |
|  |  | GM49322 | |  |
|  |  | ADCY1 |  |  |
|  |  | SCLY |  |  |
|  |  | D7ERTD443E | |  |
|  |  | NDUFAF2 | |  |
|  |  | ZAR1L |  |  |
|  |  | IFI27L2B |  |  |
|  |  | OTOR |  |  |
|  |  | OLFR25 |  |  |
|  |  | GM4792 |  |  |
|  |  | BLOC1S4 | |  |
|  |  | MSLN |  |  |
|  |  | TMEM86B | |  |
|  |  | APOL10B | |  |
|  |  | PLEKHG6 | |  |
|  |  | DNMT3C | |  |
|  |  | PLOD3 |  |  |
|  |  | RILPL1 |  |  |
|  |  | TCF12 |  |  |
|  |  | PTPRQ |  |  |
|  |  | GK |  |  |
|  |  | POSTN |  |  |
|  |  | ATOH7 |  |  |
|  |  | CHD5 |  |  |
|  |  | 2-Sep |  |  |
|  |  | RARRES1 | |  |
|  |  | ARX |  |  |
|  |  | RPS14 |  |  |
|  |  | TTC19 |  |  |
|  |  | TMEM200B | |  |
|  |  | ACD |  |  |
|  |  | PCSK7 |  |  |
|  |  | GRAMD1A | |  |
|  |  | SLX4IP |  |  |
|  |  | PRMT7 |  |  |
|  |  | TMEM88 |  |  |
|  |  | GM6741 |  |  |
|  |  | ZFP748 |  |  |
|  |  | PSMC4 |  |  |
|  |  | 1700028J19RIK | |  |
|  |  | SKIV2L |  |  |
|  |  | PDLIM2 |  |  |
|  |  | MTERF2 |  |  |
|  |  | SPCS3 |  |  |
|  |  | MAK |  |  |
|  |  | TOMM34 | |  |
|  |  | SNF8 |  |  |
|  |  | MUC1 |  |  |
|  |  | GM48552 | |  |
|  |  | HAPLN3 |  |  |
|  |  | RACGAP1 | |  |
|  |  | TRPM2 |  |  |
|  |  | NCOA1 |  |  |
|  |  | SULF2 |  |  |
|  |  | FBXO2 |  |  |
|  |  | PRPF38B | |  |
|  |  | 9230113P08RIK | |  |
|  |  | CAPN6 |  |  |
|  |  | CARTPT |  |  |
|  |  | ST3GAL5 | |  |
|  |  | FXYD3 |  |  |
|  |  | SLC20A1 |  |  |
|  |  | PPIL6 |  |  |
|  |  | PRRG4 |  |  |
|  |  | DCTN3 |  |  |
|  |  | KCNA3 |  |  |
|  |  | RUVBL2 |  |  |
|  |  | POPDC2 |  |  |
|  |  | SLC25A4 |  |  |
|  |  | RPGR |  |  |
|  |  | OLFR1393 | |  |
|  |  | GPER1 |  |  |
|  |  | TMEM63C | |  |
|  |  | SCN3A |  |  |
|  |  | MED20 |  |  |
|  |  | AGO4 |  |  |
|  |  | USP54 |  |  |
|  |  | TFRC |  |  |
|  |  | SPAG17 |  |  |
|  |  | COL6A3 |  |  |
|  |  | CAMK1 |  |  |
|  |  | MTFR1L |  |  |
|  |  | USP11 |  |  |
|  |  | PAKAP |  |  |
|  |  | TMPRSS11A | |  |
|  |  | IGF2BP1 |  |  |
|  |  | GM17374 | |  |
|  |  | ME3 |  |  |
|  |  | CORO6 |  |  |
|  |  | MRPS5 |  |  |
|  |  | CHIL1 |  |  |
|  |  | RAP2C |  |  |
|  |  | POLR1C |  |  |
|  |  | MARS |  |  |
|  |  | LRRC31 |  |  |
|  |  | TMEM45A2 | |  |
|  |  | ASB17 |  |  |
|  |  | WBP2 |  |  |
|  |  | FEZF2 |  |  |
|  |  | SHH |  |  |
|  |  | IKBKB |  |  |
|  |  | ATP10B |  |  |
|  |  | TEP1 |  |  |
|  |  | LETM2 |  |  |
|  |  | OLFR55 |  |  |
|  |  | CREB3L2 | |  |
|  |  | ADM |  |  |
|  |  | H1F0 |  |  |
|  |  | CENPX |  |  |
|  |  | COG8 |  |  |
|  |  | IRF2BPL |  |  |
|  |  | ITPKA |  |  |
|  |  | FA2H |  |  |
|  |  | RBM25 |  |  |
|  |  | CSTA1 |  |  |
|  |  | PPM1B |  |  |
|  |  | DDO |  |  |
|  |  | NPFFR2 |  |  |
|  |  | BMF |  |  |
|  |  | MPPED2 |  |  |
|  |  | PMEPA1 |  |  |
|  |  | WHRN |  |  |
|  |  | PPP2R1A | |  |
|  |  | ZFP160 |  |  |
|  |  | CYB5R3 |  |  |
|  |  | AKR1B3 |  |  |
|  |  | RND3 |  |  |
|  |  | SLC25A16 | |  |
|  |  | PIGQ |  |  |
|  |  | FAM163A | |  |
|  |  | SCNN1B |  |  |
|  |  | HHLA1 |  |  |
|  |  | PJA2 |  |  |
|  |  | NARS2 |  |  |
|  |  | HSF2BP |  |  |
|  |  | TMEM126B | |  |
|  |  | SIRT5 |  |  |
|  |  | GNPNAT1 | |  |
|  |  | PHLDB3 |  |  |
|  |  | NUGGC |  |  |
|  |  | LYN |  |  |
|  |  | HRC |  |  |
|  |  | TSTD2 |  |  |
|  |  | MOGS |  |  |
|  |  | ZFP251 |  |  |
|  |  | EIF4EBP1 | |  |
|  |  | ZFP763 |  |  |
|  |  | SFXN1 |  |  |
|  |  | OPN1SW |  |  |
|  |  | ATP6V0D1 | |  |
|  |  | FBXW4 |  |  |
|  |  | ZFP532 |  |  |
|  |  | SERTAD4 | |  |
|  |  | SRRM3 |  |  |
|  |  | EXOC3L |  |  |
|  |  | DYSF |  |  |
|  |  | VPS18 |  |  |
|  |  | GM10644 | |  |
|  |  | HIGD1A |  |  |
|  |  | BPTF |  |  |
|  |  | LAO1 |  |  |
|  |  | 4931409K22RIK | |  |
|  |  | SKINT11 |  |  |
|  |  | KAZALD1 | |  |
|  |  | ZFP275 |  |  |
|  |  | XDH |  |  |
|  |  | YBEY |  |  |
|  |  | SINHCAF | |  |
|  |  | SETD1A |  |  |
|  |  | GABPB1 |  |  |
|  |  | LRMP |  |  |
|  |  | GM1979 |  |  |
|  |  | TMEM236 | |  |
|  |  | KCNJ16 |  |  |
|  |  | GET4 |  |  |
|  |  | PPP2R2B | |  |
|  |  | SS18L2 |  |  |
|  |  | GPR150 |  |  |
|  |  | ATP13A3 | |  |
|  |  | SRD5A1 |  |  |
|  |  | SPAG4 |  |  |
|  |  | COL12A1 | |  |
|  |  | LYPLA2 |  |  |
|  |  | CREG1 |  |  |
|  |  | YBX1 |  |  |
|  |  | CHST14 |  |  |
|  |  | TPCN1 |  |  |
|  |  | ZBTB3 |  |  |
|  |  | ACTR10 |  |  |
|  |  | DCAKD |  |  |
|  |  | FGFBP3 |  |  |
|  |  | GPR18 |  |  |
|  |  | FGD5 |  |  |
|  |  | ASB11 |  |  |
|  |  | BAZ2B |  |  |
|  |  | MICAL1 |  |  |
|  |  | SYCP2 |  |  |
|  |  | BAZ1B |  |  |
|  |  | MVB12A |  |  |
|  |  | CALU |  |  |
|  |  | FPGT |  |  |
|  |  | GM10217 | |  |
|  |  | CENPA |  |  |
|  |  | ABTB1 |  |  |
|  |  | AANAT |  |  |
|  |  | RARG |  |  |
|  |  | TMEM230 | |  |
|  |  | REEP2 |  |  |
|  |  | ARHGEF19 | |  |
|  |  | ZFP185 |  |  |
|  |  | GM6096 |  |  |
|  |  | MKRN2 |  |  |
|  |  | ARID4B |  |  |
|  |  | PMM1 |  |  |
|  |  | PAIP2B |  |  |
|  |  | TNFRSF10B | |  |
|  |  | SRR |  |  |
|  |  | ABLIM3 |  |  |
|  |  | ATXN3 |  |  |
|  |  | SV2A |  |  |
|  |  | MTMR2 |  |  |
|  |  | ZFP143 |  |  |
|  |  | UNC80 |  |  |
|  |  | CENPS |  |  |
|  |  | MAP6D1 |  |  |
|  |  | NDUFAF5 | |  |
|  |  | CALM3 |  |  |
|  |  | DMP1 |  |  |
|  |  | SSR4 |  |  |
|  |  | GM5591 |  |  |
|  |  | CIB4 |  |  |
|  |  | GM21905 | |  |
|  |  | 8030474K03RIK | |  |
|  |  | OLFR262 |  |  |
|  |  | TCHHL1 |  |  |
|  |  | BPIFA3 |  |  |
|  |  | PRH1 |  |  |
|  |  | CNBD1 |  |  |
|  |  | MIA |  |  |
|  |  | GM8334 |  |  |
|  |  | CALHM4 |  |  |
|  |  | DPEP2 |  |  |
|  |  | OLFR435 |  |  |
|  |  | OLFR513 |  |  |
|  |  | KLK4 |  |  |
|  |  | OLFR1432 | |  |
|  |  | OLFR657 |  |  |
|  |  | OLFR1307 | |  |
|  |  | 1700047I17RIK2 | |  |
|  |  | KRT5 |  |  |
|  |  | GM10181 | |  |
|  |  | R3HDML | |  |
|  |  | MYL7 |  |  |
|  |  | CRISP2 |  |  |
|  |  | OLFR682-PS1 | |  |
|  |  | STK32C |  |  |
|  |  | TM4SF4 |  |  |
|  |  | HOXB3 |  |  |
|  |  | NXNL1 |  |  |
|  |  | 4930516K23RIK | |  |
|  |  | AKR1C19 | |  |
|  |  | SKINT5 |  |  |
|  |  | KRT6A |  |  |
|  |  | FAM110C | |  |
|  |  | BTNL10 |  |  |
|  |  | GM815 |  |  |
|  |  | GM3550 |  |  |
|  |  | GM3327 |  |  |
|  |  | OBOX2 |  |  |
|  |  | SLC30A4 |  |  |
|  |  | BC030870 | |  |
|  |  | VCAM1 |  |  |
|  |  | ANGPTL1 | |  |
|  |  | PLSCR4 |  |  |
|  |  | DNM2 |  |  |
|  |  | SKP1A |  |  |
|  |  | COL27A1 | |  |
|  |  | SPRYD4 |  |  |
|  |  | POLR3GL | |  |
|  |  | SFXN4 |  |  |
|  |  | SELL |  |  |
|  |  | KCTD18 |  |  |
|  |  | NUMBL |  |  |
|  |  | CSTL1 |  |  |
|  |  | DIRAS2 |  |  |
|  |  | HPDL |  |  |
|  |  | MBLAC2 |  |  |
|  |  | EIF4G3 |  |  |
|  |  | ALPK1 |  |  |
|  |  | MYL6 |  |  |
|  |  | CEP104 |  |  |
|  |  | MYBPH |  |  |
|  |  | ZFP407 |  |  |
|  |  | OLFR102 |  |  |
|  |  | ZPBP2 |  |  |
|  |  | GCGR |  |  |
|  |  | G430049J08RIK | |  |
|  |  | RALGPS1 | |  |
|  |  | KCNA2 |  |  |
|  |  | SMARCA5 | |  |
|  |  | CCDC71 |  |  |
|  |  | SPCS1 |  |  |
|  |  | FASTKD2 | |  |
|  |  | SLC35A5 |  |  |
|  |  | FAM122A | |  |
|  |  | HGD |  |  |
|  |  | CST10 |  |  |
|  |  | TRIM43B | |  |
|  |  | TM4SF5 |  |  |
|  |  | CD300LG | |  |
|  |  | FAM187A | |  |
|  |  | PLEKHS1 | |  |
|  |  | E030018B13RIK | |  |
|  |  | CUL9 |  |  |
|  |  | AGPAT4 |  |  |
|  |  | CEP57 |  |  |
|  |  | NTN5 |  |  |
|  |  | NUPL1 |  |  |
|  |  | CTU2 |  |  |
|  |  | TNS2 |  |  |
|  |  | ACPP |  |  |
|  |  | 1110065P20RIK | |  |
|  |  | GM9913 |  |  |
|  |  | LPAR3 |  |  |
|  |  | OLFR18 |  |  |
|  |  | ZFP653 |  |  |
|  |  | ZFP750 |  |  |
|  |  | PDK1 |  |  |
|  |  | PTGDR2 |  |  |
|  |  | IL2RB |  |  |
|  |  | ZFP820 |  |  |
|  |  | ZFP641 |  |  |
|  |  | CCDC65 |  |  |
|  |  | LMAN1 |  |  |
|  |  | SEZ6L2 |  |  |
|  |  | GM2A |  |  |
|  |  | EFNA3 |  |  |
|  |  | NPTN |  |  |
|  |  | FBXO40 |  |  |
|  |  | NUP93 |  |  |
|  |  | MGAT4C | |  |
|  |  | GLRA3 |  |  |
|  |  | NAPA |  |  |
|  |  | SLC7A3 |  |  |
|  |  | GM5464 |  |  |
|  |  | RMI1 |  |  |
|  |  | ABCA15 |  |  |
|  |  | GM43738 | |  |
|  |  | PIN1RT1 |  |  |
|  |  | GM11213 | |  |
|  |  | UBALD2 |  |  |
|  |  | C5AR1 |  |  |
|  |  | P3H3 |  |  |
|  |  | ANKS4B |  |  |
|  |  | TPPP |  |  |
|  |  | LAMA3 |  |  |
|  |  | PTPN6 |  |  |
|  |  | COG4 |  |  |
|  |  | DOLPP1 |  |  |
|  |  | C5AR2 |  |  |
|  |  | ZKSCAN16 | |  |
|  |  | IFI209 |  |  |
|  |  | GM9837 |  |  |
|  |  | CAPN1 |  |  |
|  |  | CSF2RA |  |  |
|  |  | SPIN2C |  |  |
|  |  | CAPNS1 |  |  |
|  |  | TAF9 |  |  |
|  |  | MPHOSPH9 | |  |
|  |  | TRMT1L |  |  |
|  |  | 9930021J03RIK | |  |
|  |  | NME5 |  |  |
|  |  | PRSS53 |  |  |
|  |  | SFRP1 |  |  |
|  |  | DCST1 |  |  |
|  |  | RFX2 |  |  |
|  |  | OLFR558 |  |  |
|  |  | SLC35G3 |  |  |
|  |  | SPATA21 | |  |
|  |  | MEX3A |  |  |
|  |  | ITGBL1 |  |  |
|  |  | KDM1B |  |  |
|  |  | PSMB5 |  |  |
|  |  | SPATA31D1A | |  |
|  |  | SLC1A7 |  |  |
|  |  | KRTCAP3 | |  |
|  |  | SMARCD1 | |  |
|  |  | SLC35A4 |  |  |
|  |  | RIOK2 |  |  |
|  |  | EFL1 |  |  |
|  |  | GPR25 |  |  |
|  |  | S100A16 |  |  |
|  |  | TRIP13 |  |  |
|  |  | NAT8F7 |  |  |
|  |  | KREMEN1 | |  |
|  |  | RPL34 |  |  |
|  |  | OAF |  |  |
|  |  | ABT1 |  |  |
|  |  | MRPL55 |  |  |
|  |  | ZBTB17 |  |  |
|  |  | BIN1 |  |  |
|  |  | CGREF1 |  |  |
|  |  | MRRF |  |  |
|  |  | COL7A1 |  |  |
|  |  | ABCD1 |  |  |
|  |  | ZFP865 |  |  |
|  |  | CLDN10 |  |  |
|  |  | PRMT2 |  |  |
|  |  | VAX1 |  |  |
|  |  | DNPH1 |  |  |
|  |  | RPS24 |  |  |
|  |  | NIF3L1 |  |  |
|  |  | DSG1C |  |  |
|  |  | SRFBP1 |  |  |
|  |  | DMRT3 |  |  |
|  |  | SYNE3 |  |  |
|  |  | NCF2 |  |  |
|  |  | CCDC57 |  |  |
|  |  | VPS45 |  |  |
|  |  | COPZ2 |  |  |
|  |  | ARMH1 |  |  |
|  |  | SOD2 |  |  |
|  |  | TRIM62 |  |  |
|  |  | PKMYT1 | |  |
|  |  | C8A |  |  |
|  |  | RABGGTA | |  |
|  |  | GRIA3 |  |  |
|  |  | TRAF3IP1 | |  |
|  |  | MTMR10 | |  |
|  |  | CTSC |  |  |
|  |  | PFDN5 |  |  |
|  |  | RNASET2B | |  |
|  |  | TKFC |  |  |
|  |  | 2310039H08RIK | |  |
|  |  | MCU |  |  |
|  |  | OXCT1 |  |  |
|  |  | DNAJC6 |  |  |
|  |  | SRA1 |  |  |
|  |  | SCX |  |  |
|  |  | RFC2 |  |  |
|  |  | B3GNT4 |  |  |
|  |  | GM5617 |  |  |
|  |  | MACC1 |  |  |
|  |  | ZMYND12 | |  |
|  |  | CROT |  |  |
|  |  | IBSP |  |  |
|  |  | GM3625 |  |  |
|  |  | VCPKMT | |  |
|  |  | ENO1B |  |  |
|  |  | MAPK8 |  |  |
|  |  | CDC20 |  |  |
|  |  | DAND5 |  |  |
|  |  | FZD3 |  |  |
|  |  | PIK3CG |  |  |
|  |  | AMER3 |  |  |
|  |  | ZFP729A |  |  |
|  |  | TGM6 |  |  |
|  |  | NOA1 |  |  |
|  |  | VGLL4 |  |  |
|  |  | MIER2 |  |  |
|  |  | ELK4 |  |  |
|  |  | TAGAP |  |  |
|  |  | WNT10B |  |  |
|  |  | TMEM173 | |  |
|  |  | ZFP385C |  |  |
|  |  | HEY2 |  |  |
|  |  | LRRC17 |  |  |
|  |  | AGR3 |  |  |
|  |  | RNF20 |  |  |
|  |  | TEX45 |  |  |
|  |  | RHEBL1 |  |  |
|  |  | SLC1A2 |  |  |
|  |  | PTX4 |  |  |
|  |  | SLC4A10 |  |  |
|  |  | TMEM18 |  |  |
|  |  | PRKAR2B | |  |
|  |  | TMEM178B | |  |
|  |  | CACTIN |  |  |
|  |  | 2310057M21RIK | |  |
|  |  | TTC25 |  |  |
|  |  | LMNB1 |  |  |
|  |  | HSD17B11 | |  |
|  |  | MEIG1 |  |  |
|  |  | FRMPD4 |  |  |
|  |  | KLC4 |  |  |
|  |  | VSIG10 |  |  |
|  |  | PITPNM3 | |  |
|  |  | PM20D2 |  |  |
|  |  | UBTD1 |  |  |
|  |  | COA4 |  |  |
|  |  | PPFIA3 |  |  |
|  |  | 3300002I08RIK | |  |
|  |  | VMN2R110 | |  |
|  |  | CYP2B19 | |  |
|  |  | TNFRSF1A | |  |
|  |  | DROSHA |  |  |
|  |  | TMEM176A | |  |
|  |  | ZMAT4 |  |  |
|  |  | RPL12 |  |  |
|  |  | NUDCD1 |  |  |
|  |  | MYPOP |  |  |
|  |  | CTDSPL2 | |  |
|  |  | SSTR5 |  |  |
|  |  | MBD3L2 |  |  |
|  |  | 5830473C10RIK | |  |
|  |  | HTR6 |  |  |
|  |  | AUP1 |  |  |
|  |  | CRB2 |  |  |
|  |  | GMCL1 |  |  |
|  |  | TRP53I13 | |  |
|  |  | RBAK |  |  |
|  |  | SLC24A5 |  |  |
|  |  | KCNC1 |  |  |
|  |  | NUDT15 |  |  |
|  |  | WDR62 |  |  |
|  |  | KAT6A |  |  |
|  |  | TMEM198 | |  |
|  |  | SIRT1 |  |  |
|  |  | TARM1 |  |  |
|  |  | H2-M10.1 | |  |
|  |  | ACP5 |  |  |
|  |  | SLFN2 |  |  |
|  |  | ALOX15 |  |  |
|  |  | SMARCC1 | |  |
|  |  | 5-Sep |  |  |
|  |  | GRAMD4 | |  |
|  |  | SEMA5B |  |  |
|  |  | CIART |  |  |
|  |  | ZFP970 |  |  |
|  |  | CNRIP1 |  |  |
|  |  | SCP2 |  |  |
|  |  | RCHY1 |  |  |
|  |  | FBXL8 |  |  |
|  |  | SERPINA1B | |  |
|  |  | SLURP2 |  |  |
|  |  | BRS3 |  |  |
|  |  | RPUSD1 |  |  |
|  |  | CCNB2 |  |  |
|  |  | TOE1 |  |  |
|  |  | GLOD4 |  |  |
|  |  | AGBL2 |  |  |
|  |  | MYO16 |  |  |
|  |  | RAP2A |  |  |
|  |  | CCDC154 | |  |
|  |  | CD79A |  |  |
|  |  | CHCHD6 |  |  |
|  |  | HNRNPM | |  |
|  |  | CFAP69 |  |  |
|  |  | NFX1 |  |  |
|  |  | TTLL10 |  |  |
|  |  | ELP3 |  |  |
|  |  | TMEM201 | |  |
|  |  | MBD2 |  |  |
|  |  | TMEM234 | |  |
|  |  | DENND2A | |  |
|  |  | ICA1L |  |  |
|  |  | MAP1LC3A | |  |
|  |  | ARHGAP29 | |  |
|  |  | KLHL12 |  |  |
|  |  | ZFP955B |  |  |
|  |  | NR1D2 |  |  |
|  |  | THAP1 |  |  |
|  |  | ANKRD7 |  |  |
|  |  | OLFR873 |  |  |
|  |  | OLFR417 |  |  |
|  |  | GM8104 |  |  |
|  |  | SLC51B |  |  |
|  |  | SMU1 |  |  |
|  |  | FBXW17 |  |  |
|  |  | MRPS34 |  |  |
|  |  | RPL36 |  |  |
|  |  | ZFP120 |  |  |
|  |  | CYGB |  |  |
|  |  | XIAP |  |  |
|  |  | NPM2 |  |  |
|  |  | MYL6B |  |  |
|  |  | SEMA4C |  |  |
|  |  | FAM163B | |  |
|  |  | GCG |  |  |
|  |  | ZFP973 |  |  |
|  |  | MC2R |  |  |
|  |  | ALKBH8 |  |  |
|  |  | MNS1 |  |  |
|  |  | BUB1 |  |  |
|  |  | FAM92B |  |  |
|  |  | TMEM64 |  |  |
|  |  | GM20219 | |  |
|  |  | ERFE |  |  |
|  |  | TNMD |  |  |
|  |  | HYI |  |  |
|  |  | PTCD3 |  |  |
|  |  | MRPS26 |  |  |
|  |  | DDIT4 |  |  |
|  |  | MYBPC3 |  |  |
|  |  | PPP2R5E | |  |
|  |  | METTL21E | |  |
|  |  | TMEM151A | |  |
|  |  | MCEE |  |  |
|  |  | SPACA9 |  |  |
|  |  | TMSB10 |  |  |
|  |  | ATP11A |  |  |
|  |  | LYPD6 |  |  |
|  |  | HAO1 |  |  |
|  |  | GLE1 |  |  |
|  |  | HNRNPA1 | |  |
|  |  | PSME2 |  |  |
|  |  | BCL7B |  |  |
|  |  | FHL4 |  |  |
|  |  | ICAM2 |  |  |
|  |  | MDM4 |  |  |
|  |  | DCTN5 |  |  |
|  |  | DEXI |  |  |
|  |  | PRSS56 |  |  |
|  |  | ASB7 |  |  |
|  |  | KLHL33 |  |  |
|  |  | NFKB1 |  |  |
|  |  | AK8 |  |  |
|  |  | NEDD4 |  |  |
|  |  | ATP9B |  |  |
|  |  | DLL3 |  |  |
|  |  | ZFP579 |  |  |
|  |  | IKBKG |  |  |
|  |  | BMS1 |  |  |
|  |  | SCEL |  |  |
|  |  | PRDM9 |  |  |
|  |  | OIP5 |  |  |
|  |  | TOPORS |  |  |
|  |  | ASXL1 |  |  |
|  |  | SASH3 |  |  |
|  |  | IQSEC2 |  |  |
|  |  | SETD5 |  |  |
|  |  | MYOC |  |  |
|  |  | 1700086D15RIK | |  |
|  |  | CWC25 |  |  |
|  |  | TBCD |  |  |
|  |  | RANBP3 |  |  |
|  |  | FGF15 |  |  |
|  |  | SLC43A1 |  |  |
|  |  | GJC1 |  |  |
|  |  | TCP11L2 |  |  |
|  |  | DTX4 |  |  |
|  |  | AMFR |  |  |
|  |  | CYTH3 |  |  |
|  |  | KIF5B |  |  |
|  |  | ACKR4 |  |  |
|  |  | FBXO11 |  |  |
|  |  | TRMT10A | |  |
|  |  | RPL3L |  |  |
|  |  | GDPD1 |  |  |
|  |  | NFS1 |  |  |
|  |  | CD68 |  |  |
|  |  | PPP1R14C | |  |
|  |  | FEM1C |  |  |
|  |  | USP24 |  |  |
|  |  | MSL3 |  |  |
|  |  | MMP19 |  |  |
|  |  | ERMP1 |  |  |
|  |  | EFNA5 |  |  |
|  |  | DNAH17 |  |  |
|  |  | PLA2G4C | |  |
|  |  | ZFP109 |  |  |
|  |  | ANKRD34B | |  |
|  |  | RFK |  |  |
|  |  | NIPSNAP3B | |  |
|  |  | GSTA2 |  |  |
|  |  | 6030498E09RIK | |  |
|  |  | ISL2 |  |  |
|  |  | ADGRG2 |  |  |
|  |  | ACSS1 |  |  |
|  |  | CWC22 |  |  |
|  |  | WDR3 |  |  |
|  |  | GADD45GIP1 | |  |
|  |  | SMARCAD1 | |  |
|  |  | TRMT11 |  |  |
|  |  | ZFP597 |  |  |
|  |  | SLC45A3 |  |  |
|  |  | RTN4RL1 | |  |
|  |  | ZRANB1 |  |  |
|  |  | FKBP3 |  |  |
|  |  | PPA2 |  |  |
|  |  | ASPG |  |  |
|  |  | AURKB |  |  |
|  |  | TMSB15B1 | |  |
|  |  | WIZ |  |  |
|  |  | WDR54 |  |  |
|  |  | MAVS |  |  |
|  |  | WDR36 |  |  |
|  |  | PLEKHF1 | |  |
|  |  | TNFRSF13B | |  |
|  |  | VANGL1 |  |  |
|  |  | SCN8A |  |  |
|  |  | NXPH4 |  |  |
|  |  | YIF1B |  |  |
|  |  | SLC16A2 |  |  |
|  |  | NAV3 |  |  |
|  |  | PCDHGB5 | |  |
|  |  | REPS2 |  |  |
|  |  | DYRK2 |  |  |
|  |  | SIM1 |  |  |
|  |  | AMIGO3 |  |  |
|  |  | PPP1R11 |  |  |
|  |  | CLTB |  |  |
|  |  | GM3278 |  |  |
|  |  | ASXL2 |  |  |
|  |  | UNC5B |  |  |
|  |  | NT5C1A |  |  |
|  |  | SSB |  |  |
|  |  | PRSS57 |  |  |
|  |  | DNAJC11 | |  |
|  |  | RUNDC3A | |  |
|  |  | FAM219A | |  |
|  |  | NXT1 |  |  |
|  |  | DVL2 |  |  |
|  |  | OGDHL |  |  |
|  |  | SLC35D1 |  |  |
|  |  | CTPS2 |  |  |
|  |  | QTRT2 |  |  |
|  |  | MLYCD |  |  |
|  |  | PTCD1 |  |  |
|  |  | TRANK1 |  |  |
|  |  | 2610528J11RIK | |  |
|  |  | CBR4 |  |  |
|  |  | CPPED1 |  |  |
|  |  | FAM83F |  |  |
|  |  | ITIH4 |  |  |
|  |  | GM10334 | |  |
|  |  | PTH2R |  |  |
|  |  | RAPGEF2 | |  |
|  |  | ELAVL1 |  |  |
|  |  | TVP23A |  |  |
|  |  | BTBD2 |  |  |
|  |  | BBS12 |  |  |
|  |  | WDR11 |  |  |
|  |  | CXCL14 |  |  |
|  |  | RALGDS |  |  |
|  |  | NUDT16 |  |  |
|  |  | IFFO1 |  |  |
|  |  | TM6SF1 |  |  |
|  |  | IFI207 |  |  |
|  |  | CLIP2 |  |  |
|  |  | ZFP524 |  |  |
|  |  | GTF3C4 |  |  |
|  |  | LSM4 |  |  |
|  |  | SLC22A2 |  |  |
|  |  | NANOS1 |  |  |
|  |  | PHRF1 |  |  |
|  |  | ELMOD1 |  |  |
|  |  | GM21028 | |  |
|  |  | ELF4 |  |  |
|  |  | SNRPB2 |  |  |
|  |  | EIF3D |  |  |
|  |  | DCAF15 |  |  |
|  |  | TAMM41 | |  |
|  |  | PLPP1 |  |  |
|  |  | MSANTD3 | |  |
|  |  | PARN |  |  |
|  |  | GLRA4 |  |  |
|  |  | MARK2 |  |  |
|  |  | ARHGEF25 | |  |
|  |  | MFF |  |  |
|  |  | ATRX |  |  |
|  |  | PSMA3 |  |  |
|  |  | EPYC |  |  |
|  |  | SLC25A27 | |  |
|  |  | PRRG1 |  |  |
|  |  | OLFR53 |  |  |
|  |  | ARHGAP26 | |  |
|  |  | ARHGAP17 | |  |
|  |  | ORAOV1 |  |  |
|  |  | CERS6 |  |  |
|  |  | MCM4 |  |  |
|  |  | ARMCX5 | |  |
|  |  | BABAM1 |  |  |
|  |  | CLASP1 |  |  |
|  |  | RBM19 |  |  |
|  |  | EPB41L1 |  |  |
|  |  | MAGEB3 |  |  |
|  |  | PCBP3 |  |  |
|  |  | GBP6 |  |  |
|  |  | COPS7A |  |  |
|  |  | IFI27L2A |  |  |
|  |  | NR2C2 |  |  |
|  |  | CCDC85C | |  |
|  |  | GM10778 | |  |
|  |  | PRSS55 |  |  |
|  |  | NME1 |  |  |
|  |  | TTLL13 |  |  |
|  |  | ADAM28 |  |  |
|  |  | RPRD2 |  |  |
|  |  | DUSP10 |  |  |
|  |  | DNAJC16 | |  |
|  |  | AW554918 | |  |
|  |  | ZFP664 |  |  |
|  |  | CNGA2 |  |  |
|  |  | CCDC82 |  |  |
|  |  | CMKLR1 |  |  |
|  |  | NOC3L |  |  |
|  |  | SOX10 |  |  |
|  |  | RPS18 |  |  |
|  |  | TMEM266 | |  |
|  |  | GM17409 | |  |
|  |  | PRR22 |  |  |
|  |  | CLN3 |  |  |
|  |  | DENND1A | |  |
|  |  | KMT5C |  |  |
|  |  | CCNA1 |  |  |
|  |  | AMER1 |  |  |
|  |  | VBP1 |  |  |
|  |  | XAF1 |  |  |
|  |  | AMIGO1 |  |  |
|  |  | ZFP961 |  |  |
|  |  | GZF1 |  |  |
|  |  | CHORDC1 | |  |
|  |  | ZFP738 |  |  |
|  |  | TMEM29 |  |  |
|  |  | DPM3 |  |  |
|  |  | GM14409 | |  |
|  |  | MEIS3 |  |  |
|  |  | CCDC28A | |  |
|  |  | EDEM3 |  |  |
|  |  | ORM2 |  |  |
|  |  | ZBTB49 |  |  |
|  |  | ORMDL3 | |  |
|  |  | MTG2 |  |  |
|  |  | IL1F5 |  |  |
|  |  | ZFP966 |  |  |
|  |  | GKN1 |  |  |
|  |  | RPL22L1 |  |  |
|  |  | RCBTB2 |  |  |
|  |  | PDCD7 |  |  |
|  |  | YPEL1 |  |  |
|  |  | MYRF |  |  |
|  |  | TRPC1 |  |  |
|  |  | TIMM8A1 | |  |
|  |  | IP6K3 |  |  |
|  |  | SMC2 |  |  |
|  |  | HMGN2 |  |  |
|  |  | ZRANB2 |  |  |
|  |  | MRGBP |  |  |
|  |  | KAT2A |  |  |
|  |  | HEATR3 |  |  |
|  |  | CLDN5 |  |  |
|  |  | MCUB |  |  |
|  |  | PIAS4 |  |  |
|  |  | CREBRF |  |  |
|  |  | OVOL1 |  |  |
|  |  | GM12353 | |  |
|  |  | HOOK2 |  |  |
|  |  | CNTF |  |  |
|  |  | NKX2-1 |  |  |
|  |  | SPSB4 |  |  |
|  |  | UPRT |  |  |
|  |  | SULT2B1 | |  |
|  |  | TTL |  |  |
|  |  | SLCO5A1 | |  |
|  |  | TOR1AIP2 | |  |
|  |  | OSCAR |  |  |
|  |  | FCRL1 |  |  |
|  |  | CFAP73 |  |  |
|  |  | SEL1L |  |  |
|  |  | SMIM6 |  |  |
|  |  | KLF7 |  |  |
|  |  | EPS15 |  |  |
|  |  | PNMAL1 |  |  |
|  |  | ANKRD17 | |  |
|  |  | AMDHD2 | |  |
|  |  | REXO1 |  |  |
|  |  | GDI2 |  |  |
|  |  | ZFP14 |  |  |
|  |  | TNFRSF18 | |  |
|  |  | PCDHB13 | |  |
|  |  | FILIP1L |  |  |
|  |  | GM4491 |  |  |
|  |  | EIF2AK2 |  |  |
|  |  | SNRPD1 |  |  |
|  |  | 1520401A03RIK | |  |
|  |  | ANKRD34C | |  |
|  |  | MRGPRE | |  |
|  |  | CD302 |  |  |
|  |  | 5031410I06RIK | |  |
|  |  | CLEC3B |  |  |
|  |  | GM11733 | |  |
|  |  | IGSF9B |  |  |
|  |  | TEX10 |  |  |
|  |  | GHITM |  |  |
|  |  | TOM1L1 |  |  |
|  |  | BTBD11 |  |  |
|  |  | WIPI2 |  |  |
|  |  | CENPM |  |  |
|  |  | SPATA31D1D | |  |
|  |  | DBIL5 |  |  |
|  |  | SAP18B |  |  |
|  |  | PPP4R3B | |  |
|  |  | DNAJB11 | |  |
|  |  | 9130023H24RIK | |  |
|  |  | RNF113A2 | |  |
|  |  | TRAPPC6A | |  |
|  |  | RPUSD2 |  |  |
|  |  | PRUNE1 |  |  |
|  |  | DNAJC7 |  |  |
|  |  | ADCK2 |  |  |
|  |  | HCCS |  |  |
|  |  | RAPGEF1 | |  |
|  |  | CAVIN2 |  |  |
|  |  | CBFA2T2 | |  |
|  |  | CMTM7 |  |  |
|  |  | FAM168A | |  |
|  |  | PPP1R3D | |  |
|  |  | RPL18A |  |  |
|  |  | FAM83E |  |  |
|  |  | RNF122 |  |  |
|  |  | OPTC |  |  |
|  |  | TSC1 |  |  |
|  |  | JKAMP |  |  |
|  |  | ADH1 |  |  |
|  |  | OGFR |  |  |
|  |  | GLRA1 |  |  |
|  |  | ISG20L2 |  |  |
|  |  | PPP1R13B | |  |
|  |  | GM21887 | |  |
|  |  | CXCL11 |  |  |
|  |  | ADGRG5 |  |  |
|  |  | GABRR1 |  |  |
|  |  | ARIH1 |  |  |
|  |  | ARHGAP20 | |  |
|  |  | SEC14L5 |  |  |
|  |  | ZFAND1 |  |  |
|  |  | PILRA |  |  |
|  |  | TXLNB |  |  |
|  |  | PISD |  |  |
|  |  | CYP39A1 | |  |
|  |  | SLC25A3 |  |  |
|  |  | B2M |  |  |
|  |  | AP1S3 |  |  |
|  |  | CRY2 |  |  |
|  |  | RADIL |  |  |
|  |  | CLU |  |  |
|  |  | GJA4 |  |  |
|  |  | TMEM59 |  |  |
|  |  | SCYL3 |  |  |
|  |  | DHPS |  |  |
|  |  | PYCRL |  |  |
|  |  | GM7102 |  |  |
|  |  | PNPLA8 |  |  |
|  |  | CPA2 |  |  |
|  |  | BDH2 |  |  |
|  |  | LAMA4 |  |  |
|  |  | GM10184 | |  |
|  |  | SNAPC1 |  |  |
|  |  | NDRG2 |  |  |
|  |  | ARR3 |  |  |
|  |  | RIC8A |  |  |
|  |  | GM10638 | |  |
|  |  | FOXS1 |  |  |
|  |  | TNNI3K |  |  |
|  |  | TNFSF9 |  |  |
|  |  | TGFBRAP1 | |  |
|  |  | B4GALT3 | |  |
|  |  | ZCCHC8 |  |  |
|  |  | PSME4 |  |  |
|  |  | ADGRA1 |  |  |
|  |  | AKAP2 |  |  |
|  |  | CACNG2 |  |  |
|  |  | ITK |  |  |
|  |  | FCHSD2 |  |  |
|  |  | DUSP16 |  |  |
|  |  | MICALL1 | |  |
|  |  | GALNT14 | |  |
|  |  | DCDC2B |  |  |
|  |  | NDUFC1 |  |  |
|  |  | EFTUD2 |  |  |
|  |  | NPAS4 |  |  |
|  |  | APOH |  |  |
|  |  | ASB8 |  |  |
|  |  | COLGALT1 | |  |
|  |  | GM26558 | |  |
|  |  | D930020B18RIK | |  |
|  |  | ANKIB1 |  |  |
|  |  | SERPINB6A | |  |
|  |  | AKT3 |  |  |
|  |  | FGD2 |  |  |
|  |  | POLR3E |  |  |
|  |  | NCMAP |  |  |
|  |  | INO80E |  |  |
|  |  | SH3TC1 |  |  |
|  |  | HSPA13 |  |  |
|  |  | AP5M1 |  |  |
|  |  | WDSUB1 | |  |
|  |  | GALNT1 |  |  |
|  |  | TEAD4 |  |  |
|  |  | MRPL19 |  |  |
|  |  | TNKS2 |  |  |
|  |  | FGFR4 |  |  |
|  |  | IRX3 |  |  |
|  |  | SLC22A7 |  |  |
|  |  | RAB3GAP2 | |  |
|  |  | ZFP362 |  |  |
|  |  | MAD2L1BP | |  |
|  |  | ZFP808 |  |  |
|  |  | NARS |  |  |
|  |  | CYP4F13 |  |  |
|  |  | ACKR1 |  |  |
|  |  | SPRYD3 |  |  |
|  |  | LRFN3 |  |  |
|  |  | TCEAL7 |  |  |
|  |  | MYL1 |  |  |
|  |  | HSPBP1 |  |  |
|  |  | TRIT1 |  |  |
|  |  | 6430571L13RIK | |  |
|  |  | HSD17B4 | |  |
|  |  | 2010106E10RIK | |  |
|  |  | KRTAP4-1 | |  |
|  |  | GM21854 | |  |
|  |  | OLFR285 |  |  |
|  |  | FCER1A |  |  |
|  |  | FGB |  |  |
|  |  | GM17677 | |  |
|  |  | NKX1-1 |  |  |
|  |  | VMN1R9 |  |  |
|  |  | SLCO6D1 | |  |
|  |  | GM17402 | |  |
|  |  | PTH2 |  |  |
|  |  | ILK |  |  |
|  |  | OLFR735 |  |  |
|  |  | GM11939 | |  |
|  |  | CST11 |  |  |
|  |  | OLFR175-PS1 | |  |
|  |  | KCNJ15 |  |  |
|  |  | 4930556J24RIK | |  |
|  |  | ATAD2 |  |  |
|  |  | OLFR979 |  |  |
|  |  | PRSS32 |  |  |
|  |  | FERD3L |  |  |
|  |  | VMN2R58 | |  |
|  |  | OLFR315 |  |  |
|  |  | OLFR330 |  |  |
|  |  | OLFR370 |  |  |
|  |  | GM45783 | |  |
|  |  | OLFR578 |  |  |
|  |  | GM8024 |  |  |
|  |  | OLFR678 |  |  |
|  |  | OLFR1330 | |  |
|  |  | OLFR1390 | |  |
|  |  | MFSD6L |  |  |
|  |  | MUG2 |  |  |
|  |  | USP9Y |  |  |
|  |  | CRYGS |  |  |
|  |  | PITX3 |  |  |
|  |  | GM2956 |  |  |
|  |  | GM10128 | |  |
|  |  | FCMR |  |  |
|  |  | MYF6 |  |  |
|  |  | SULT3A2 | |  |
|  |  | 2310057J18RIK | |  |
|  |  | GSDMC |  |  |
|  |  | HSPA4L |  |  |
|  |  | OLFR59 |  |  |
|  |  | OLFR11 |  |  |
|  |  | GJB5 |  |  |
|  |  | CTCFL |  |  |
|  |  | HOXB4 |  |  |
|  |  | SKINT3 |  |  |
|  |  | GM2381 |  |  |
|  |  | PFN5 |  |  |
|  |  | OLFR680-PS1 | |  |
|  |  | 4933402J07RIK | |  |
|  |  | GM3404 |  |  |
|  |  | GM4763 |  |  |
|  |  | CD70 |  |  |
|  |  | TRIM31 |  |  |
|  |  | CCL4 |  |  |
|  |  | SPINK5 |  |  |
|  |  | GDF15 |  |  |
|  |  | LRCOL1 |  |  |
|  |  | 1700092M07RIK | |  |
|  |  | MYRFL |  |  |
|  |  | THA1 |  |  |
|  |  | ITGA1 |  |  |
|  |  | SYTL5 |  |  |
|  |  | BIK |  |  |
|  |  | GM28557 | |  |
|  |  | OLFR859 |  |  |
|  |  | ADGRB3 |  |  |
|  |  | LAMP1 |  |  |
|  |  | CPSF1 |  |  |
|  |  | AMER2 |  |  |
|  |  | CHST10 |  |  |
|  |  | CSRNP3 |  |  |
|  |  | GM49373 | |  |
|  |  | PAXIP1 |  |  |
|  |  | BEX3 |  |  |
|  |  | RSPH6A |  |  |
|  |  | HSPA14 |  |  |
|  |  | PLIN5 |  |  |
|  |  | TRMO |  |  |
|  |  | BCR |  |  |
|  |  | ZNHIT2 |  |  |
|  |  | ROCK2 |  |  |
|  |  | NOL4L |  |  |
|  |  | PRR11 |  |  |
|  |  | ZFP94 |  |  |
|  |  | IMPA1 |  |  |
|  |  | SELENBP1 | |  |
|  |  | FAM102B | |  |
|  |  | PITRM1 |  |  |
|  |  | SEPHS1 |  |  |
|  |  | ABCC12 |  |  |
|  |  | WDR83OS | |  |
|  |  | SETD7 |  |  |
|  |  | SWT1 |  |  |
|  |  | FERMT1 |  |  |
|  |  | WASHC1 |  |  |
|  |  | MEGF8 |  |  |
|  |  | CCDC107 | |  |
|  |  | SLC12A5 |  |  |
|  |  | NOL12 |  |  |
|  |  | ADAM3 |  |  |
|  |  | CASTOR1 | |  |
|  |  | ZUP1 |  |  |
|  |  | KCNAB2 |  |  |
|  |  | CLDN7 |  |  |
|  |  | NT5C2 |  |  |
|  |  | CYP2J6 |  |  |
|  |  | LIN7A |  |  |
|  |  | MECOM |  |  |
|  |  | PDXP |  |  |
|  |  | 1700019N19RIK | |  |
|  |  | FAM84A |  |  |
|  |  | DIAPH1 |  |  |
|  |  | TAS1R3 |  |  |
|  |  | SPACA5 |  |  |
|  |  | PIGC |  |  |
|  |  | FAM92A |  |  |
|  |  | SMIM3 |  |  |
|  |  | JPH3 |  |  |
|  |  | SOX30 |  |  |
|  |  | RASGRP4 | |  |
|  |  | TBX3 |  |  |
|  |  | SLC19A2 |  |  |
|  |  | REEP4 |  |  |
|  |  | CENPU |  |  |
|  |  | ATG9B |  |  |
|  |  | RHO |  |  |
|  |  | 4930432K21RIK | |  |
|  |  | FRG2F1 |  |  |
|  |  | NAT8F3 |  |  |
|  |  | BIVM |  |  |
|  |  | DECR1 |  |  |
|  |  | FBP1 |  |  |
|  |  | RBM45 |  |  |
|  |  | PDLIM7 |  |  |
|  |  | CN725425 | |  |
|  |  | ATP10A |  |  |
|  |  | CAPZA2 |  |  |
|  |  | ARHGAP22 | |  |
|  |  | UHRF1BP1 | |  |
|  |  | DPP8 |  |  |
|  |  | METTL25 | |  |
|  |  | ZFP710 |  |  |
|  |  | CALR4 |  |  |
|  |  | CDCA7L |  |  |
|  |  | RAB24 |  |  |
|  |  | SREK1IP1 | |  |
|  |  | ZFP189 |  |  |
|  |  | SP140 |  |  |
|  |  | ITGA7 |  |  |
|  |  | EHD3 |  |  |
|  |  | HS2ST1 |  |  |
|  |  | RAB6B |  |  |
|  |  | RASAL3 |  |  |
|  |  | D630023F18RIK | |  |
|  |  | RAB36 |  |  |
|  |  | TSPAN15 | |  |
|  |  | ABCG3 |  |  |
|  |  | PROCA1 |  |  |
|  |  | GM6588 |  |  |
|  |  | PRKN |  |  |
|  |  | CWC15 |  |  |
|  |  | B3GAT1 |  |  |
|  |  | RGMA |  |  |
|  |  | 4933425L06RIK | |  |
|  |  | NEK8 |  |  |
|  |  | THSD7A |  |  |
|  |  | SELENOH | |  |
|  |  | TBXA2R |  |  |
|  |  | RPLP1 |  |  |
|  |  | SMG6 |  |  |
|  |  | KRIT1 |  |  |
|  |  | JAKMIP2 | |  |
|  |  | CLPX |  |  |
|  |  | SYDE1 |  |  |
|  |  | GM10722 | |  |
|  |  | GM31371 | |  |
|  |  | NR1I3 |  |  |
|  |  | DCAF6 |  |  |
|  |  | PSRC1 |  |  |
|  |  | PHF11C |  |  |
|  |  | RCN3 |  |  |
|  |  | DRD3 |  |  |
|  |  | PRPF8 |  |  |
|  |  | NDUFS7 |  |  |
|  |  | ZPLD1 |  |  |
|  |  | PARG |  |  |
|  |  | PLPP7 |  |  |
|  |  | EMB |  |  |
|  |  | OSTF1 |  |  |
|  |  | GNS |  |  |
|  |  | ARPC1A |  |  |
|  |  | SESTD1 |  |  |
|  |  | GM8797 |  |  |
|  |  | MTM1 |  |  |
|  |  | IQUB |  |  |
|  |  | MAGOHB | |  |
|  |  | MAPK7 |  |  |
|  |  | NPHP4 |  |  |
|  |  | SLC2A5 |  |  |
|  |  | CD209G |  |  |
|  |  | ATP13A2 | |  |
|  |  | LRRC7 |  |  |
|  |  | ZBTB7C |  |  |
|  |  | GFOD2 |  |  |
|  |  | FAM124A | |  |
|  |  | NDC1 |  |  |
|  |  | MYH7 |  |  |
|  |  | MIP |  |  |
|  |  | PTCH2 |  |  |
|  |  | TRIM5 |  |  |
|  |  | FAM126B | |  |
|  |  | CHTF8 |  |  |
|  |  | SREK1 |  |  |
|  |  | 9930104L06RIK | |  |
|  |  | RNMT |  |  |
|  |  | OLFR1512 | |  |
|  |  | CYTIP |  |  |
|  |  | URB1 |  |  |
|  |  | CCDC150 | |  |
|  |  | HPX |  |  |
|  |  | GPBAR1 |  |  |
|  |  | ZMYM4 |  |  |
|  |  | UCHL1 |  |  |
|  |  | CD200 |  |  |
|  |  | WASHC3 |  |  |
|  |  | ENKD1 |  |  |
|  |  | IFT43 |  |  |
|  |  | HIST1H2AK | |  |
|  |  | PEX10 |  |  |
|  |  | LRFN2 |  |  |
|  |  | L2HGDH |  |  |
|  |  | CLDN19 |  |  |
|  |  | 1700020A23RIK | |  |
|  |  | RP1 |  |  |
|  |  | LILRA5 |  |  |
|  |  | GM21663 | |  |
|  |  | TCEAL9 |  |  |
|  |  | TMEM262 | |  |
|  |  | STK25 |  |  |
|  |  | 2310030G06RIK | |  |
|  |  | AXL |  |  |
|  |  | ANKRD13A | |  |
|  |  | CYB5R1 |  |  |
|  |  | PGAP1 |  |  |
|  |  | GIMAP6 |  |  |
|  |  | SPI1 |  |  |
|  |  | CDK5R2 |  |  |
|  |  | MYO15 |  |  |
|  |  | NUP160 |  |  |
|  |  | ICA1 |  |  |
|  |  | CELF5 |  |  |
|  |  | B230104I21RIK | |  |
|  |  | PEX6 |  |  |
|  |  | OTUB2 |  |  |
|  |  | PPCS |  |  |
|  |  | TRIM8 |  |  |
|  |  | ZFP300 |  |  |
|  |  | PNPT1 |  |  |
|  |  | ADI1 |  |  |
|  |  | RBPJ |  |  |
|  |  | ANKRD10 | |  |
|  |  | GDPD4 |  |  |
|  |  | TRMT44 |  |  |
|  |  | KNSTRN |  |  |
|  |  | TPH1 |  |  |
|  |  | EPC1 |  |  |
|  |  | COPG2 |  |  |
|  |  | PPP1R12A | |  |
|  |  | CDH1 |  |  |
|  |  | DCAF5 |  |  |
|  |  | ZFP992 |  |  |
|  |  | RNF167 |  |  |
|  |  | GM14124 | |  |
|  |  | TRAPPC6B | |  |
|  |  | ELK1 |  |  |
|  |  | CNPY4 |  |  |
|  |  | ADRB1 |  |  |
|  |  | RGS2 |  |  |
|  |  | PDE6A |  |  |
|  |  | RAP1A |  |  |
|  |  | SEMA3G |  |  |
|  |  | HS6ST3 |  |  |
|  |  | PRICKLE3 | |  |
|  |  | MMAB |  |  |
|  |  | FNIP1 |  |  |
|  |  | IRAK4 |  |  |
|  |  | KAT14 |  |  |
|  |  | FAM53B |  |  |
|  |  | INSRR |  |  |
|  |  | MVK |  |  |
|  |  | CDIPT |  |  |
|  |  | FRK |  |  |
|  |  | NXPE2 |  |  |
|  |  | 2510039O18RIK | |  |
|  |  | APOL8 |  |  |
|  |  | DIMT1 |  |  |
|  |  | DLD |  |  |
|  |  | RNGTT |  |  |
|  |  | CCL12 |  |  |
|  |  | MOB3C |  |  |
|  |  | H2-BL |  |  |
|  |  | LRPPRC |  |  |
|  |  | ZFP30 |  |  |
|  |  | CCDC138 | |  |
|  |  | ZFP53 |  |  |
|  |  | GHDC |  |  |
|  |  | TRIM23 |  |  |
|  |  | UTP18 |  |  |
|  |  | POLR3B |  |  |
|  |  | SAP30 |  |  |
|  |  | HACE1 |  |  |
|  |  | RNF14 |  |  |
|  |  | AGPAT1 |  |  |
|  |  | FIGNL2 |  |  |
|  |  | HEATR4 |  |  |
|  |  | ARHGAP1 | |  |
|  |  | CHRNA5 |  |  |
|  |  | IFT52 |  |  |
|  |  | TSPOAP1 | |  |
|  |  | TMEM163 | |  |
|  |  | MRPL22 |  |  |
|  |  | EXOSC4 |  |  |
|  |  | SDHB |  |  |
|  |  | IDH3G |  |  |
|  |  | ITGB6 |  |  |
|  |  | ODF3L2 |  |  |
|  |  | SEZ6 |  |  |
|  |  | BRD1 |  |  |
|  |  | CHST3 |  |  |
|  |  | MSTN |  |  |
|  |  | LRRC34 |  |  |
|  |  | TNRC6B |  |  |
|  |  | ARCN1 |  |  |
|  |  | BC049715 | |  |
|  |  | ELOF1 |  |  |
|  |  | PNMA2 |  |  |
|  |  | EVI2 |  |  |
|  |  | GID8 |  |  |
|  |  | TMC6 |  |  |
|  |  | HIGD2A |  |  |
|  |  | IL4 |  |  |
|  |  | H2AFJ |  |  |
|  |  | GPX6 |  |  |
|  |  | CACNG4 |  |  |
|  |  | GPR119 |  |  |
|  |  | ZFP287 |  |  |
|  |  | PRR7 |  |  |
|  |  | MFSD2A |  |  |
|  |  | DGKI |  |  |
|  |  | TSPYL3 |  |  |
|  |  | DNAH1 |  |  |
|  |  | ROS1 |  |  |
|  |  | MXD3 |  |  |
|  |  | CINP |  |  |
|  |  | SYNGR2 |  |  |
|  |  | SOX21 |  |  |
|  |  | RPP40 |  |  |
|  |  | RBP1 |  |  |
|  |  | SWSAP1 |  |  |
|  |  | GM28285 | |  |
|  |  | GSTT3 |  |  |
|  |  | SCML4 |  |  |
|  |  | MAP7D3 |  |  |
|  |  | ZFP108 |  |  |
|  |  | RHBDL1 |  |  |
|  |  | P2RY1 |  |  |
|  |  | CCDC90B | |  |
|  |  | FXYD5 |  |  |
|  |  | GRK4 |  |  |
|  |  | DERL2 |  |  |
|  |  | OLFR551 |  |  |
|  |  | H2-T22 |  |  |
|  |  | BAK1 |  |  |
|  |  | NEUROG2 | |  |
|  |  | AEBP2 |  |  |
|  |  | WDR34 |  |  |
|  |  | SNX2 |  |  |
|  |  | FBXL14 |  |  |
|  |  | MCL1 |  |  |
|  |  | ZCRB1 |  |  |
|  |  | POGLUT1 | |  |
|  |  | TERT |  |  |
|  |  | LPXN |  |  |
|  |  | CTBS |  |  |
|  |  | SCRT2 |  |  |
|  |  | METTL4 |  |  |
|  |  | HCST |  |  |
|  |  | SMPX |  |  |
|  |  | SLC4A9 |  |  |
|  |  | JMJD4 |  |  |
|  |  | VSTM2A |  |  |
|  |  | NSRP1 |  |  |
|  |  | 2200002D01RIK | |  |
|  |  | BCL9L |  |  |
|  |  | AVPR2 |  |  |
|  |  | XRRA1 |  |  |
|  |  | PER2 |  |  |
|  |  | ZFP418 |  |  |
|  |  | OLFR560 |  |  |
|  |  | FAM135A | |  |
|  |  | YIPF6 |  |  |
|  |  | SPOP |  |  |
|  |  | SYT11 |  |  |
|  |  | RANBP2 |  |  |
|  |  | LCOR |  |  |
|  |  | ACVR1B |  |  |
|  |  | PKNOX1 |  |  |
|  |  | CNTROB | |  |
|  |  | SLC25A46 | |  |
|  |  | OLFR374 |  |  |
|  |  | SALL1 |  |  |
|  |  | TMEM209 | |  |
|  |  | DUSP27 |  |  |
|  |  | TTC4 |  |  |
|  |  | ZFP977 |  |  |
|  |  | GJA5 |  |  |
|  |  | SDR39U1 | |  |
|  |  | ZFP979 |  |  |
|  |  | CSRNP2 |  |  |
|  |  | SLC27A1 |  |  |
|  |  | MYH9 |  |  |
|  |  | BSG |  |  |
|  |  | DIP2B |  |  |
|  |  | ASPA |  |  |
|  |  | ZFP758 |  |  |
|  |  | SLAMF9 |  |  |
|  |  | BGLAP |  |  |
|  |  | APOL6 |  |  |
|  |  | OLFR312 |  |  |
|  |  | CPLX4 |  |  |
|  |  | KLK7 |  |  |
|  |  | SMIM9 |  |  |
|  |  | ZFP988 |  |  |
|  |  | FCAMR |  |  |
|  |  | GM5936 |  |  |
|  |  | 4930583I09RIK | |  |
|  |  | IFFO2 |  |  |
|  |  | SPIB |  |  |
|  |  | TMEM100 | |  |
|  |  | IGHMBP2 | |  |
|  |  | SNRNP70 | |  |
|  |  | PLK1 |  |  |
|  |  | GM2897 |  |  |
|  |  | ZSCAN12 | |  |
|  |  | TRIM2 |  |  |
|  |  | COX15 |  |  |
|  |  | DDX20 |  |  |
|  |  | N4BP2L2 | |  |
|  |  | 1700024G13RIK | |  |
|  |  | IL27RA |  |  |
|  |  | SIRT3 |  |  |
|  |  | CCDC81 |  |  |
|  |  | DNAH5 |  |  |
|  |  | ZFP607B |  |  |
|  |  | OTOS |  |  |
|  |  | LENG8 |  |  |
|  |  | NDUFA8 |  |  |
|  |  | NRN1 |  |  |
|  |  | ANAPC1 |  |  |
|  |  | FBXO42 |  |  |
|  |  | MSS51 |  |  |
|  |  | LMNTD2 | |  |
|  |  | RABAC1 |  |  |
|  |  | PTPRJ |  |  |
|  |  | MFGE8 |  |  |
|  |  | DRG1 |  |  |
|  |  | EIF4G2 |  |  |
|  |  | PARK7 |  |  |
|  |  | TRIM43C | |  |
|  |  | GM6583 |  |  |
|  |  | GGTA1 |  |  |
|  |  | MRGPRA9 | |  |
|  |  | VMN2R2 |  |  |
|  |  | TRMT10B | |  |
|  |  | GPR62 |  |  |
|  |  | TMPRSS3 | |  |
|  |  | TSX |  |  |
|  |  | ZAR1 |  |  |
|  |  | TRAPPC1 | |  |
|  |  | GPR135 |  |  |
|  |  | CC2D2B |  |  |
|  |  | MORC2B | |  |
|  |  | SH3D21 |  |  |
|  |  | SCAND1 |  |  |
|  |  | AW146154 | |  |
|  |  | UTS2R |  |  |
|  |  | SAMD7 |  |  |
|  |  | PIH1H3B |  |  |
|  |  | NUP155 |  |  |
|  |  | ELOVL7 |  |  |
|  |  | ZFP575 |  |  |
|  |  | SLC5A6 |  |  |
|  |  | MATK |  |  |
|  |  | PPP1R3E | |  |
|  |  | CBLN4 |  |  |
|  |  | B3GNT2 |  |  |
|  |  | SLC17A7 |  |  |
|  |  | CUTC |  |  |
|  |  | ATP10D |  |  |
|  |  | CASQ1 |  |  |
|  |  | PUSL1 |  |  |
|  |  | BTC |  |  |
|  |  | EPHA8 |  |  |
|  |  | DUSP19 |  |  |
|  |  | CCDC97 |  |  |
|  |  | DDX39 |  |  |
|  |  | AKAP8L |  |  |
|  |  | SOAT1 |  |  |
|  |  | TMEM5 |  |  |
|  |  | THOC2 |  |  |
|  |  | CLIC4 |  |  |
|  |  | GM16286 | |  |
|  |  | P2RY13 |  |  |
|  |  | KHDRBS2 | |  |
|  |  | ARF6 |  |  |
|  |  | SPATA33 | |  |
|  |  | NDST1 |  |  |
|  |  | SDCBP |  |  |
|  |  | ATP6V0C | |  |
|  |  | SEC22A |  |  |
|  |  | CDS2 |  |  |
|  |  | H2AL3 |  |  |
|  |  | GGN |  |  |
|  |  | KRT87 |  |  |
|  |  | GM14401 | |  |
|  |  | RRP8 |  |  |
|  |  | NDUFB11 | |  |
|  |  | PAFAH1B3 | |  |
|  |  | DR1 |  |  |
|  |  | PURA |  |  |
|  |  | 2210408I21RIK | |  |
|  |  | MYT1L |  |  |
|  |  | ALKBH6 |  |  |
|  |  | BHLHA15 | |  |
|  |  | CIAPIN1 |  |  |
|  |  | ZFP646 |  |  |
|  |  | ZMYND19 | |  |
|  |  | CENPJ |  |  |
|  |  | FOCAD |  |  |
|  |  | HRK |  |  |
|  |  | SLC39A4 |  |  |
|  |  | MX1 |  |  |
|  |  | MYOM3 |  |  |
|  |  | LIN54 |  |  |
|  |  | E430018J23RIK | |  |
|  |  | ZAP70 |  |  |
|  |  | IRF2BP1 |  |  |
|  |  | SLC1A6 |  |  |
|  |  | TTLL12 |  |  |
|  |  | NOC4L |  |  |
|  |  | MAFF |  |  |
|  |  | BTRC |  |  |
|  |  | MND1 |  |  |
|  |  | SRP14 |  |  |
|  |  | GM17571 | |  |
|  |  | NFATC2IP | |  |
|  |  | MBOAT1 | |  |
|  |  | SSC5D |  |  |
|  |  | ZNRF2 |  |  |
|  |  | MEOX1 |  |  |
|  |  | SNRPB |  |  |
|  |  | AAR2 |  |  |
|  |  | FEM1A |  |  |
|  |  | SPEER4A | |  |
|  |  | 2610002M06RIK | |  |
|  |  | WRNIP1 |  |  |
|  |  | ELL2 |  |  |
|  |  | ARL16 |  |  |
|  |  | VWA1 |  |  |
|  |  | CDH11 |  |  |
|  |  | HECTD3 |  |  |
|  |  | CCDC141 | |  |
|  |  | PNMT |  |  |
|  |  | BORCS6 |  |  |
|  |  | HMBS |  |  |
|  |  | ZFP558 |  |  |
|  |  | EML4 |  |  |
|  |  | MAPK12 |  |  |
|  |  | CD276 |  |  |
|  |  | SOX18 |  |  |
|  |  | SLC35G1 |  |  |
|  |  | ZFP180 |  |  |
|  |  | WDR73 |  |  |
|  |  | PDE5A |  |  |
|  |  | TOX4 |  |  |
|  |  | ATP5B |  |  |
|  |  | PEX12 |  |  |
|  |  | CLASRP |  |  |
|  |  | TPMT |  |  |
|  |  | RPS29 |  |  |
|  |  | EIPR1 |  |  |
|  |  | LYPD2 |  |  |
|  |  | CRYGN |  |  |
|  |  | LYG2 |  |  |
|  |  | TOMM20L | |  |
|  |  | GTF2H5 |  |  |
|  |  | GM45618 | |  |
|  |  | IL34 |  |  |
|  |  | POLR2D |  |  |
|  |  | BPIFC |  |  |
|  |  | GM10642 | |  |
|  |  | GM11232 | |  |
|  |  | RSL24D1 |  |  |
|  |  | PCLAF |  |  |
|  |  | TMEM185A | |  |
|  |  | ATOH1 |  |  |
|  |  | LAMC2 |  |  |
|  |  | HPS5 |  |  |
|  |  | ALDH9A1 | |  |
|  |  | CLCN3 |  |  |
|  |  | CEP164 |  |  |
|  |  | FUK |  |  |
|  |  | 2210016L21RIK | |  |
|  |  | FBXL21 |  |  |
|  |  | KLF6 |  |  |
|  |  | NELFCD |  |  |
|  |  | SMIM15 |  |  |
|  |  | LHPP |  |  |
|  |  | RELL1 |  |  |
|  |  | CALR3 |  |  |
|  |  | YY1 |  |  |
|  |  | D17WSU92E | |  |
|  |  | ANKK1 |  |  |
|  |  | UNC119 |  |  |
|  |  | SIVA1 |  |  |
|  |  | CFAP206 |  |  |
|  |  | MLKL |  |  |
|  |  | RRN3 |  |  |
|  |  | OFD1 |  |  |
|  |  | CLEC12A | |  |
|  |  | ANP32B |  |  |
|  |  | CASS4 |  |  |
|  |  | ARFGAP1 | |  |
|  |  | MRPS6 |  |  |
|  |  | B3GALT6 | |  |
|  |  | GPR12 |  |  |
|  |  | CHST7 |  |  |
|  |  | CAPZB |  |  |
|  |  | HIST2H3C1 | |  |
|  |  | RNF144B | |  |
|  |  | CFL1 |  |  |
|  |  | B3GALT5 | |  |
|  |  | ZDHHC3 |  |  |
|  |  | DEF6 |  |  |
|  |  | DISP1 |  |  |
|  |  | PDK4 |  |  |
|  |  | IL7 |  |  |
|  |  | NMRK1 |  |  |
|  |  | CD109 |  |  |
|  |  | GADD45B | |  |
|  |  | GAL |  |  |
|  |  | ICAM1 |  |  |
|  |  | TMEM175 | |  |
|  |  | RNASET2A | |  |
|  |  | SPINK13 |  |  |
|  |  | PPP6R1 |  |  |
|  |  | EPHB4 |  |  |
|  |  | UCKL1 |  |  |
|  |  | NKAIN2 |  |  |
|  |  | PTGR2 |  |  |
|  |  | GTSF1 |  |  |
|  |  | TECRL |  |  |
|  |  | TOP3B |  |  |
|  |  | LRP10 |  |  |
|  |  | MAP4 |  |  |
|  |  | MRPS18A | |  |
|  |  | SPOUT1 |  |  |
|  |  | PPL |  |  |
|  |  | SEC61A1 |  |  |
|  |  | MAIP1 |  |  |
|  |  | LDB2 |  |  |
|  |  | RPS12-PS3 | |  |
|  |  | FBXO46 |  |  |
|  |  | HRAS |  |  |
|  |  | COPG1 |  |  |
|  |  | IDH2 |  |  |
|  |  | MACROD1 | |  |
|  |  | ZBTB43 |  |  |
|  |  | DCTPP1 |  |  |
|  |  | EDC3 |  |  |
|  |  | TMEM37 |  |  |
|  |  | ADAMTS5 | |  |
|  |  | DHRS1 |  |  |
|  |  | MPPED1 |  |  |
|  |  | SHTN1 |  |  |
|  |  | SOCS3 |  |  |
|  |  | ATP6V0A1 | |  |
|  |  | PRPF39 |  |  |
|  |  | ABI3 |  |  |
|  |  | ANKRD46 | |  |
|  |  | KLHL42 |  |  |
|  |  | AI413582 |  |  |
|  |  | MCTP2 |  |  |
|  |  | DNM1L |  |  |
|  |  | RNF148 |  |  |
|  |  | SEPHS2 |  |  |
|  |  | CABLES1 | |  |
|  |  | XBP1 |  |  |
|  |  | ZFP707 |  |  |
|  |  | ASPRV1 |  |  |
|  |  | NUDT12 |  |  |
|  |  | EDEM1 |  |  |
|  |  | SVS2 |  |  |
|  |  | CHIL3 |  |  |
|  |  | TBX20 |  |  |
|  |  | CYP11B2 | |  |
|  |  | VMN2R111 | |  |
|  |  | STRA6L |  |  |
|  |  | DDX51 |  |  |
|  |  | CRTC1 |  |  |
|  |  | HCN4 |  |  |
|  |  | CSRP2 |  |  |
|  |  | RWDD2B | |  |
|  |  | C1QTNF9 | |  |
|  |  | CNTD1 |  |  |
|  |  | CDC14B |  |  |
|  |  | OTOP3 |  |  |
|  |  | CR1L |  |  |
|  |  | ACER1 |  |  |
|  |  | FABP2 |  |  |
|  |  | DSG1A |  |  |
|  |  | DUOX2 |  |  |
|  |  | OLFR988 |  |  |
|  |  | TFEC |  |  |
|  |  | ACTBL2 |  |  |
|  |  | OLFR672 |  |  |
|  |  | GM10840 | |  |
|  |  | TMSB15B2 | |  |
|  |  | PSME2B |  |  |
|  |  | CYP2AB1 | |  |
|  |  | LYZL6 |  |  |
|  |  | GM13941 | |  |
|  |  | DEFB36 |  |  |
|  |  | PDPN |  |  |
|  |  | PGLYRP2 | |  |
|  |  | WFDC12 |  |  |
|  |  | CD3E |  |  |
|  |  | PKLR |  |  |
|  |  | ELF3 |  |  |
|  |  | PLA2G4D | |  |
|  |  | ALB |  |  |
|  |  | MFSD9 |  |  |
|  |  | GBP2B |  |  |
|  |  | BOC |  |  |
|  |  | LYZL4 |  |  |
|  |  | IVD |  |  |
|  |  | CAPN3 |  |  |
|  |  | AFAP1 |  |  |
|  |  | SLC6A16 |  |  |
|  |  | NOXRED1 | |  |
|  |  | SPRYD7 |  |  |
|  |  | SERTAD3 | |  |
|  |  | PEF1 |  |  |
|  |  | TEF |  |  |
|  |  | APP |  |  |
|  |  | GM10563 | |  |
|  |  | GM9958 |  |  |
|  |  | PANX1 |  |  |
|  |  | TRP53 |  |  |
|  |  | SNIP1 |  |  |
|  |  | ZKSCAN5 | |  |
|  |  | TMSB4X |  |  |
|  |  | OLFR482 |  |  |
|  |  | PHXR2 |  |  |
|  |  | ARL13B |  |  |
|  |  | TMEM213 | |  |
|  |  | TRAK2 |  |  |
|  |  | TNIP1 |  |  |
|  |  | JAM3 |  |  |
|  |  | RPS5 |  |  |
|  |  | FAM221B | |  |
|  |  | CFAP45 |  |  |
|  |  | RUNX1 |  |  |
|  |  | ZFYVE28 | |  |
|  |  | SPTBN2 |  |  |
|  |  | PSD3 |  |  |
|  |  | COPRS |  |  |
|  |  | MGA |  |  |
|  |  | ATP5C1 |  |  |
|  |  | OLFR1434 | |  |
|  |  | 7530416G11RIK | |  |
|  |  | PAX5 |  |  |
|  |  | HAMP2 |  |  |
|  |  | TINF2 |  |  |
|  |  | SLC24A2 |  |  |
|  |  | AQR |  |  |
|  |  | TSPYL5 |  |  |
|  |  | STK11IP |  |  |
|  |  | THAP8 |  |  |
|  |  | CCR7 |  |  |
|  |  | GPN2 |  |  |
|  |  | PTPRK |  |  |
|  |  | PLEC |  |  |
|  |  | PSMD5 |  |  |
|  |  | DCLRE1C | |  |
|  |  | SEMA3D |  |  |
|  |  | CLUAP1 |  |  |
|  |  | FOXJ2 |  |  |
|  |  | CFLAR |  |  |
|  |  | ZFP799 |  |  |
|  |  | MPC2 |  |  |
|  |  | RNLS |  |  |
|  |  | SLC46A3 |  |  |
|  |  | ZBED5 |  |  |
|  |  | ZFP963 |  |  |
|  |  | ZFP57 |  |  |
|  |  | RNF31 |  |  |
|  |  | PPP4R1 |  |  |
|  |  | LRIG2 |  |  |
|  |  | COIL |  |  |
|  |  | CPSF4 |  |  |
|  |  | NOS3 |  |  |
|  |  | OXGR1 |  |  |
|  |  | PIK3R3 |  |  |
|  |  | CMTM8 |  |  |
|  |  | EEF1A2 |  |  |
|  |  | MN1 |  |  |
|  |  | CDC14A |  |  |
|  |  | KHDRBS1 | |  |
|  |  | KCP |  |  |
|  |  | PCDHB15 | |  |
|  |  | ECD |  |  |
|  |  | CCT2 |  |  |
|  |  | VMN2R57 | |  |
|  |  | MGAM |  |  |
|  |  | CFAP53 |  |  |
|  |  | GM5141 |  |  |
|  |  | GLI2 |  |  |
|  |  | CD24A |  |  |
|  |  | MRPS2 |  |  |
|  |  | LIMS1 |  |  |
|  |  | PDSS2 |  |  |
|  |  | PNRC1 |  |  |
|  |  | BPIFB1 |  |  |
|  |  | ZSWIM4 |  |  |
|  |  | COL28A1 | |  |
|  |  | ADAM1B | |  |
|  |  | EBF1 |  |  |
|  |  | SCG5 |  |  |
|  |  | FER1L6 |  |  |
|  |  | MFSD4B5 | |  |
|  |  | MRTO4 |  |  |
|  |  | ARFIP1 |  |  |
|  |  | DDB2 |  |  |
|  |  | CDH2 |  |  |
|  |  | BDH1 |  |  |
|  |  | CTNNBL1 | |  |
|  |  | SLC9A3R1 | |  |
|  |  | ZFP329 |  |  |
|  |  | PKD1 |  |  |
|  |  | HP1BP3 |  |  |
|  |  | CKAP2L |  |  |
|  |  | MRPS30 |  |  |
|  |  | ABO |  |  |
|  |  | LELP1 |  |  |
|  |  | CLEC4D |  |  |
|  |  | CYPT15 |  |  |
|  |  | OLFR178 |  |  |
|  |  | ADAM20 |  |  |
|  |  | ADAM30 |  |  |
|  |  | UQCRC1 |  |  |
|  |  | KLK13 |  |  |
|  |  | PROC |  |  |
|  |  | KRTAP26-1 | |  |
|  |  | CMA1 |  |  |
|  |  | IFI206 |  |  |
|  |  | TINAG |  |  |
|  |  | FAM3B |  |  |
|  |  | OLFR891 |  |  |
|  |  | OLFR884 |  |  |
|  |  | LTA |  |  |
|  |  | E030025P04RIK | |  |
|  |  | VMN2R30 | |  |
|  |  | RERGL |  |  |
|  |  | KLRB1F |  |  |
|  |  | AMBP |  |  |
|  |  | OLFR766-PS1 | |  |
|  |  | BTN1A1 |  |  |
|  |  | OLFR1277 | |  |
|  |  | OLFR453 |  |  |
|  |  | CEACAM19 | |  |
|  |  | GM10770 | |  |
|  |  | OLFR667 |  |  |
|  |  | MESP1 |  |  |
|  |  | CLCA2 |  |  |
|  |  | OLFR639 |  |  |
|  |  | IFNAB |  |  |
|  |  | OLFR1340 | |  |
|  |  | GM26938 | |  |
|  |  | BMP10 |  |  |
|  |  | IL9R |  |  |
|  |  | TAAR5 |  |  |
|  |  | GM10142 | |  |
|  |  | PRODH |  |  |
|  |  | GSDMA |  |  |
|  |  | H2AFB2 |  |  |
|  |  | GM47283 | |  |
|  |  | DEFB20 |  |  |
|  |  | HOXC5 |  |  |
|  |  | TNNC2 |  |  |
|  |  | PRDX6B |  |  |
|  |  | IRX2 |  |  |
|  |  | D13ERTD608E | |  |
|  |  | PLSCR5 |  |  |
|  |  | MILL1 |  |  |
|  |  | KRTAP24-1 | |  |
|  |  | MRGPRB1 | |  |
|  |  | FAM240B | |  |
|  |  | TTC24 |  |  |
|  |  | CCL3 |  |  |
|  |  | LCE1G |  |  |
|  |  | ATP6V1C1 | |  |
|  |  | GM14408 | |  |
|  |  | LHX8 |  |  |
|  |  | CSF1 |  |  |
|  |  | ZFP628 |  |  |
|  |  | CDK5RAP3 | |  |
|  |  | UTRN |  |  |
|  |  | EDAR |  |  |
|  |  | MAN1C1 |  |  |
|  |  | NIPAL3 |  |  |
|  |  | ARHGAP27 | |  |
|  |  | IL5RA |  |  |
|  |  | OPN3 |  |  |
|  |  | GZMM |  |  |
|  |  | CD80 |  |  |
|  |  | RBM12 |  |  |
|  |  | PLA2G2C | |  |
|  |  | DNTTIP2 | |  |
|  |  | MYL2 |  |  |
|  |  | DOCK6 |  |  |
|  |  | PCIF1 |  |  |
|  |  | CHMP6 |  |  |
|  |  | SETDB1 |  |  |
|  |  | MYH8 |  |  |
|  |  | AIFM2 |  |  |
|  |  | HEXA |  |  |
|  |  | ISPD |  |  |
|  |  | SLC22A5 |  |  |
|  |  | CCDC18 |  |  |
|  |  | TBX15 |  |  |
|  |  | MRPL54 |  |  |
|  |  | FMO5 |  |  |
|  |  | ARMC8 |  |  |
|  |  | SH2B1 |  |  |
|  |  | PM20D1 |  |  |
|  |  | TKT |  |  |
|  |  | INTS2 |  |  |
|  |  | CARD14 |  |  |
|  |  | PCDHA12 | |  |
|  |  | SLC16A1 |  |  |
|  |  | EED |  |  |
|  |  | HS1BP3 |  |  |
|  |  | BRSK1 |  |  |
|  |  | TET1 |  |  |
|  |  | HRASLS5 | |  |
|  |  | RAD51AP2 | |  |
|  |  | NGFR |  |  |
|  |  | ADD1 |  |  |
|  |  | GM38431 | |  |
|  |  | CNTN3 |  |  |
|  |  | STOML3 |  |  |
|  |  | CPD |  |  |
|  |  | SEC13 |  |  |
|  |  | EN2 |  |  |
|  |  | TMEM177 | |  |
|  |  | HIST1H1B | |  |
|  |  | TMEM214 | |  |
|  |  | E130116L18RIK | |  |
|  |  | TTYH1 |  |  |
|  |  | CD59A |  |  |
|  |  | LRRC20 |  |  |
|  |  | CENPC1 |  |  |
|  |  | PLPP5 |  |  |
|  |  | PRKAG3 |  |  |
|  |  | EXD2 |  |  |
|  |  | SYDE2 |  |  |
|  |  | EBNA1BP2 | |  |
|  |  | PLD2 |  |  |
|  |  | NSD2 |  |  |
|  |  | IDI1 |  |  |
|  |  | FBRSL1 |  |  |
|  |  | ACSF3 |  |  |
|  |  | TRABD2B | |  |
|  |  | 4933427I04RIK | |  |
|  |  | MFN1 |  |  |
|  |  | PCSK1N |  |  |
|  |  | AGFG2 |  |  |
|  |  | NMRAL1 |  |  |
|  |  | ZFP965 |  |  |
|  |  | METTL24 | |  |
|  |  | ZFP280D |  |  |
|  |  | PCDHGA9 | |  |
|  |  | RBM20 |  |  |
|  |  | DNA2 |  |  |
|  |  | SPHKAP |  |  |
|  |  | JPH1 |  |  |
|  |  | SLC25A37 | |  |
|  |  | TMEM106A | |  |
|  |  | ASH2L |  |  |
|  |  | GEMIN8 |  |  |
|  |  | AGBL1 |  |  |
|  |  | BMYC |  |  |
|  |  | CDKN1A |  |  |
|  |  | HYLS1 |  |  |
|  |  | GM7489 |  |  |
|  |  | GRPR |  |  |
|  |  | AI987944 |  |  |
|  |  | POLR3D |  |  |
|  |  | TGM1 |  |  |
|  |  | PPTC7 |  |  |
|  |  | 6330409D20RIK | |  |
|  |  | ZDHHC15 | |  |
|  |  | SUGP1 |  |  |
|  |  | TPGS2 |  |  |
|  |  | CDC25C |  |  |
|  |  | CAPNS2 |  |  |
|  |  | AP3D1 |  |  |
|  |  | LIN52 |  |  |
|  |  | CHRNA10 | |  |
|  |  | DDI1 |  |  |
|  |  | KMT5A |  |  |
|  |  | MPHOSPH10 | |  |
|  |  | COX6C |  |  |
|  |  | SFI1 |  |  |
|  |  | BIRC3 |  |  |
|  |  | NELFB |  |  |
|  |  | ACY3 |  |  |
|  |  | PPP1R8 |  |  |
|  |  | NXF1 |  |  |
|  |  | GOPC |  |  |
|  |  | SLC33A1 |  |  |
|  |  | TAOK3 |  |  |
|  |  | CYC1 |  |  |
|  |  | REC8 |  |  |
|  |  | SLC26A5 |  |  |
|  |  | MC1R |  |  |
|  |  | ATP5J2 |  |  |
|  |  | CCNJ |  |  |
|  |  | 1700066B19RIK | |  |
|  |  | ST6GALNAC1 | |  |
|  |  | CLEC1A |  |  |
|  |  | CCSER2 |  |  |
|  |  | NUF2 |  |  |
|  |  | MTCH1 |  |  |
|  |  | 1700030K09RIK | |  |
|  |  | ZCWPW1 | |  |
|  |  | BANK1 |  |  |
|  |  | MMACHC | |  |
|  |  | TCEA1 |  |  |
|  |  | DALRD3 |  |  |
|  |  | RRP15 |  |  |
|  |  | IRF8 |  |  |
|  |  | CSNK2A2 | |  |
|  |  | JAKMIP1 | |  |
|  |  | METTL7A3 | |  |
|  |  | NOS1AP |  |  |
|  |  | CDK16 |  |  |
|  |  | H2-M3 |  |  |
|  |  | ATG16L2 | |  |
|  |  | B3GALNT1 | |  |
|  |  | 1190007I07RIK | |  |
|  |  | CMTR1 |  |  |
|  |  | ACAP2 |  |  |
|  |  | MSN |  |  |
|  |  | UNC13B |  |  |
|  |  | LYSMD2 |  |  |
|  |  | SMC3 |  |  |
|  |  | PVR |  |  |
|  |  | SBK1 |  |  |
|  |  | GM6970 |  |  |
|  |  | MRPL38 |  |  |
|  |  | GGA2 |  |  |
|  |  | ZSCAN26 | |  |
|  |  | CLEC4A3 | |  |
|  |  | FAM89B |  |  |
|  |  | SLC7A1 |  |  |
|  |  | APEH |  |  |
|  |  | ABCC8 |  |  |
|  |  | RASAL1 |  |  |
|  |  | NFE2L3 |  |  |
|  |  | TTI1 |  |  |
|  |  | DENND1B | |  |
|  |  | ITGA11 |  |  |
|  |  | LRMDA |  |  |
|  |  | KALRN |  |  |
|  |  | NGB |  |  |
|  |  | HIST1H2AG | |  |
|  |  | SLC16A12 | |  |
|  |  | LSM5 |  |  |
|  |  | SMPD5 |  |  |
|  |  | SNTG1 |  |  |
|  |  | S100G |  |  |
|  |  | NDST2 |  |  |
|  |  | CXCR6 |  |  |
|  |  | PAM |  |  |
|  |  | SOCS7 |  |  |
|  |  | DNMT1 |  |  |
|  |  | NELFA |  |  |
|  |  | AI481877 |  |  |
|  |  | BRCA2 |  |  |
|  |  | H60B |  |  |
|  |  | CDKN2D |  |  |
|  |  | NAT8L |  |  |
|  |  | TIMM50 |  |  |
|  |  | NPSR1 |  |  |
|  |  | PDZK1 |  |  |
|  |  | PEA15A |  |  |
|  |  | PID1 |  |  |
|  |  | MADCAM1 | |  |
|  |  | PRPF6 |  |  |
|  |  | IGFALS |  |  |
|  |  | MMADHC | |  |
|  |  | FGFR3 |  |  |
|  |  | ADAMTS2 | |  |
|  |  | MICU3 |  |  |
|  |  | LRRC6 |  |  |
|  |  | ISM2 |  |  |
|  |  | ATP6AP1 | |  |
|  |  | FAM214A | |  |
|  |  | NASP |  |  |
|  |  | 1700025G04RIK | |  |
|  |  | TEX29 |  |  |
|  |  | ZDHHC7 |  |  |
|  |  | ICOSL |  |  |
|  |  | IL12A |  |  |
|  |  | SDK1 |  |  |
|  |  | HMGN5 |  |  |
|  |  | TSHZ1 |  |  |
|  |  | RRP12 |  |  |
|  |  | PRKCSH |  |  |
|  |  | TRP63 |  |  |
|  |  | CHMP2A |  |  |
|  |  | ACTR5 |  |  |
|  |  | DOCK2 |  |  |
|  |  | ZFP426 |  |  |
|  |  | GPR183 |  |  |
|  |  | MPP7 |  |  |
|  |  | ROR2 |  |  |
|  |  | ING1 |  |  |
|  |  | HES3 |  |  |
|  |  | RRS1 |  |  |
|  |  | SHPRH |  |  |
|  |  | PSMD1 |  |  |
|  |  | POLR3C |  |  |
|  |  | SMS |  |  |
|  |  | THOC5 |  |  |
|  |  | ZKSCAN17 | |  |
|  |  | NUB1 |  |  |
|  |  | MEIS1 |  |  |
|  |  | CLMP |  |  |
|  |  | PUS10 |  |  |
|  |  | TNFRSF4 | |  |
|  |  | PNPLA7 |  |  |
|  |  | PLPPR1 |  |  |
|  |  | LARP4B |  |  |
|  |  | SLC5A7 |  |  |
|  |  | PATZ1 |  |  |
|  |  | SPOPL |  |  |
|  |  | ZFP39 |  |  |
|  |  | SLC16A14 | |  |
|  |  | WDPCP |  |  |
|  |  | OLFR239 |  |  |
|  |  | MXD1 |  |  |
|  |  | MMP24 |  |  |
|  |  | SLFNL1 |  |  |
|  |  | GPC2 |  |  |
|  |  | HIVEP1 |  |  |
|  |  | STOX1 |  |  |
|  |  | ALDH2 |  |  |
|  |  | SLC5A4B | |  |
|  |  | TBX21 |  |  |
|  |  | 1700016C15RIK | |  |
|  |  | ROPN1 |  |  |
|  |  | HAS1 |  |  |
|  |  | ABCB6 |  |  |
|  |  | SLC2A13 |  |  |
|  |  | BICDL1 |  |  |
|  |  | CCDC13 |  |  |
|  |  | MIS18BP1 | |  |
|  |  | FBXO48 |  |  |
|  |  | MKKS |  |  |
|  |  | PRKD1 |  |  |
|  |  | SLC25A34 | |  |
|  |  | DYNC2LI1 | |  |
|  |  | PBX4 |  |  |
|  |  | ABCG2 |  |  |
|  |  | AKR1C18 | |  |
|  |  | MRPL30 |  |  |
|  |  | RC3H1 |  |  |
|  |  | OLIG2 |  |  |
|  |  | PABPC5 |  |  |
|  |  | CDHR4 |  |  |
|  |  | GM5113 |  |  |
|  |  | VPS51 |  |  |
|  |  | ULK4 |  |  |
|  |  | FKRP |  |  |
|  |  | ENOX2 |  |  |
|  |  | PHKG2 |  |  |
|  |  | NFIX |  |  |
|  |  | KCNE4 |  |  |
|  |  | DPP7 |  |  |
|  |  | GALNT4 |  |  |
|  |  | DRP2 |  |  |
|  |  | TMEM241 | |  |
|  |  | FGFR1OP | |  |
|  |  | KCNJ14 |  |  |
|  |  | 0610009B22RIK | |  |
|  |  | D10JHU81E | |  |
|  |  | RLF |  |  |
|  |  | CDH22 |  |  |
|  |  | S100A9 |  |  |
|  |  | UBL4B |  |  |
|  |  | NELL1 |  |  |
|  |  | POMT1 |  |  |
|  |  | 4931406C07RIK | |  |
|  |  | KLHL1 |  |  |
|  |  | LEPR |  |  |
|  |  | SRPX |  |  |
|  |  | GJD3 |  |  |
|  |  | PAK4 |  |  |
|  |  | ING5 |  |  |
|  |  | MRPS7 |  |  |
|  |  | GPR21 |  |  |
|  |  | MTR |  |  |
|  |  | SSC4D |  |  |
|  |  | NRSN2 |  |  |
|  |  | CLCN2 |  |  |
|  |  | TMEM41A | |  |
|  |  | GPR4 |  |  |
|  |  | UBXN10 |  |  |
|  |  | CETN2 |  |  |
|  |  | DDX52 |  |  |
|  |  | 9430097D07RIK | |  |
|  |  | AW549877 | |  |
|  |  | BRD2 |  |  |
|  |  | MFSD12 |  |  |
|  |  | PRKRIP1 | |  |
|  |  | CPZ |  |  |
|  |  | GCKR |  |  |
|  |  | AU022751 | |  |
|  |  | YES1 |  |  |
|  |  | DDAH2 |  |  |
|  |  | SACM1L |  |  |
|  |  | IFT88 |  |  |
|  |  | TBC1D15 | |  |
|  |  | GDA |  |  |
|  |  | COLEC10 | |  |
|  |  | GM15821 | |  |
|  |  | GIMAP9 |  |  |
|  |  | GM1113 |  |  |
|  |  | VIPAS39 |  |  |
|  |  | MIPOL1 |  |  |
|  |  | CDKN3 |  |  |
|  |  | PCDHB9 |  |  |
|  |  | CCDC159 | |  |
|  |  | ITGA2B |  |  |
|  |  | BIN2 |  |  |
|  |  | AKAP1 |  |  |
|  |  | DDX19B |  |  |
|  |  | GTF2B |  |  |
|  |  | MAP1S |  |  |
|  |  | TENT5C |  |  |
|  |  | MYH4 |  |  |
|  |  | NAT8F2 |  |  |
|  |  | TRPC6 |  |  |
|  |  | SLC16A11 | |  |
|  |  | ADPRHL2 | |  |
|  |  | DCUN1D4 | |  |
|  |  | PIGYL |  |  |
|  |  | PCNX2 |  |  |
|  |  | TTC3 |  |  |
|  |  | RAB31 |  |  |
|  |  | KBTBD12 | |  |
|  |  | RPGRIP1L | |  |
|  |  | HAUS6 |  |  |
|  |  | MAP3K15 | |  |
|  |  | UTP20 |  |  |
|  |  | CYBC1 |  |  |
|  |  | RNF133 |  |  |
|  |  | GALNT9 |  |  |
|  |  | PIGT |  |  |
|  |  | LCORL |  |  |
|  |  | STXBP5 |  |  |
|  |  | SLC16A7 |  |  |
|  |  | MMP21 |  |  |
|  |  | UBE2G1 |  |  |
|  |  | WTIP |  |  |
|  |  | WNT16 |  |  |
|  |  | FBXO7 |  |  |
|  |  | GHR |  |  |
|  |  | HGS |  |  |
|  |  | TTLL5 |  |  |
|  |  | SNX30 |  |  |
|  |  | ARAF |  |  |
|  |  | INPP5A |  |  |
|  |  | NRXN1 |  |  |
|  |  | MT2 |  |  |
|  |  | LGR4 |  |  |
|  |  | PFKFB3 |  |  |
|  |  | GPX3 |  |  |
|  |  | ADD2 |  |  |
|  |  | FCGBP |  |  |
|  |  | DEDD |  |  |
|  |  | PPP1R15A | |  |
|  |  | PSMD8 |  |  |
|  |  | WDR35 |  |  |
|  |  | TAPT1 |  |  |
|  |  | PLET1 |  |  |
|  |  | FTL1-PS1 | |  |
|  |  | GM49359 | |  |
|  |  | SMAD3 |  |  |
|  |  | KLHL29 |  |  |
|  |  | VPREB1 |  |  |
|  |  | PSMB8 |  |  |
|  |  | P3H4 |  |  |
|  |  | SARS2 |  |  |
|  |  | GM14322 | |  |
|  |  | SCG3 |  |  |
|  |  | EEF1E1 |  |  |
|  |  | PDHB |  |  |
|  |  | SELENON | |  |
|  |  | TMPO |  |  |
|  |  | TSG101 |  |  |
|  |  | NFIC |  |  |
|  |  | ACADM |  |  |
|  |  | GM15446 | |  |
|  |  | TRPA1 |  |  |
|  |  | PRR18 |  |  |
|  |  | ASB5 |  |  |
|  |  | IKZF4 |  |  |
|  |  | 4930452B06RIK | |  |
|  |  | ALDH3B1 | |  |
|  |  | GGT7 |  |  |
|  |  | TENT4A |  |  |
|  |  | SIX1 |  |  |
|  |  | RSPH14 |  |  |
|  |  | ARHGEF11 | |  |
|  |  | BAG3 |  |  |
|  |  | FRMPD2 |  |  |
|  |  | LRTM2 |  |  |
|  |  | DAB2IP |  |  |
|  |  | LMF2 |  |  |
|  |  | FAM178B | |  |
|  |  | PNMA3 |  |  |
|  |  | F2 |  |  |
|  |  | MTRF1 |  |  |
|  |  | HLTF |  |  |
|  |  | PRDM2 |  |  |
|  |  | PGK1 |  |  |
|  |  | WDR70 |  |  |
|  |  | VMN2R56 | |  |
|  |  | ETFRF1 |  |  |
|  |  | RNF216 |  |  |
|  |  | IFT20 |  |  |
|  |  | POP4 |  |  |
|  |  | CLDN34C1 | |  |
|  |  | COX6B1 |  |  |
|  |  | AP1M1 |  |  |
|  |  | FAM172A | |  |
|  |  | ENO1 |  |  |
|  |  | CEBPA |  |  |
|  |  | FEZ2 |  |  |
|  |  | ENGASE |  |  |
|  |  | LRBA |  |  |
|  |  | PEX11A |  |  |
|  |  | LRWD1 |  |  |
|  |  | TUBB4A |  |  |
|  |  | KCNMB3 | |  |
|  |  | RANBP17 | |  |
|  |  | MBD4 |  |  |
|  |  | NOB1 |  |  |
|  |  | LGI4 |  |  |
|  |  | SLC27A4 |  |  |
|  |  | KRT28 |  |  |
|  |  | NAA35 |  |  |
|  |  | IER5L |  |  |
|  |  | DUSP9 |  |  |
|  |  | EBI3 |  |  |
|  |  | ZFP174 |  |  |
|  |  | LEO1 |  |  |
|  |  | CD274 |  |  |
|  |  | STIM1 |  |  |
|  |  | GM6569 |  |  |
|  |  | PITPNC1 | |  |
|  |  | DTWD2 |  |  |
|  |  | GTF2A1 |  |  |
|  |  | LRRC39 |  |  |
|  |  | POMC |  |  |
|  |  | WDR92 |  |  |
|  |  | DPY19L2 | |  |
|  |  | PBDC1 |  |  |
|  |  | TMEM128 | |  |
|  |  | FN3K |  |  |
|  |  | MYBPHL | |  |
|  |  | SMC6 |  |  |
|  |  | RAC2 |  |  |
|  |  | DNAJB2 |  |  |
|  |  | UBE3C |  |  |
|  |  | TET2 |  |  |
|  |  | AI464131 |  |  |
|  |  | ALOX8 |  |  |
|  |  | CTBP1 |  |  |
|  |  | PDCL2 |  |  |
|  |  | OLFR539 |  |  |
|  |  | SOBP |  |  |
|  |  | USHBP1 |  |  |
|  |  | NAT9 |  |  |
|  |  | PROSER1 | |  |
|  |  | D430041D05RIK | |  |
|  |  | LAMTOR4 | |  |
|  |  | MRPL1 |  |  |
|  |  | ERGIC3 |  |  |
|  |  | TICAM1 |  |  |
|  |  | TESK2 |  |  |
|  |  | CBLL1 |  |  |
|  |  | WDR60 |  |  |
|  |  | ZBTB42 |  |  |
|  |  | GM10775 | |  |
|  |  | SUSD5 |  |  |
|  |  | OLFR235 |  |  |
|  |  | 9930111J21RIK2 | |  |
|  |  | A930002H24RIK | |  |
|  |  | ERI2 |  |  |
|  |  | TYR |  |  |
|  |  | ALDH3B3 | |  |
|  |  | CLEC2I |  |  |
|  |  | ADH7 |  |  |
|  |  | COL4A6 |  |  |
|  |  | GM4756 |  |  |
|  |  | FMO4 |  |  |
|  |  | HINT1 |  |  |
|  |  | CCDC8 |  |  |
|  |  | ADCK1 |  |  |
|  |  | MAFA |  |  |
|  |  | USH1G |  |  |
|  |  | ELMO1 |  |  |
|  |  | GPS2 |  |  |
|  |  | CDC7 |  |  |
|  |  | KIF20B |  |  |
|  |  | DBT |  |  |
|  |  | ABCC1 |  |  |
|  |  | MAP3K19 | |  |
|  |  | PACSIN3 |  |  |
|  |  | ZC3H4 |  |  |
|  |  | ABCF3 |  |  |
|  |  | SHC1 |  |  |
|  |  | CAMKMT | |  |
|  |  | ABAT |  |  |
|  |  | ARHGEF2 | |  |
|  |  | GM8439 |  |  |
|  |  | CDC25A |  |  |
|  |  | NEK7 |  |  |
|  |  | SPAG5 |  |  |
|  |  | SLC7A6OS | |  |
|  |  | SLF2 |  |  |
|  |  | TMEM135 | |  |
|  |  | MYO18A |  |  |
|  |  | 5031439G07RIK | |  |
|  |  | IFITM5 |  |  |
|  |  | HDHD5 |  |  |
|  |  | WNK3 |  |  |
|  |  | NPAT |  |  |
|  |  | LUC7L3 |  |  |
|  |  | GMPR2 |  |  |
|  |  | PROS1 |  |  |
|  |  | RBFOX2 |  |  |
|  |  | TEK |  |  |
|  |  | MLLT11 |  |  |
|  |  | SLC6A19 |  |  |
|  |  | ANO6 |  |  |
|  |  | CEP290 |  |  |
|  |  | SMIM7 |  |  |
|  |  | GPRC5D |  |  |
|  |  | 2410004P03RIK | |  |
|  |  | GNB2 |  |  |
|  |  | GM49355 | |  |
|  |  | PIN4 |  |  |
|  |  | FAM118A | |  |
|  |  | PMPCA |  |  |
|  |  | SLC22A17 | |  |
|  |  | ITGB8 |  |  |
|  |  | KAT2B |  |  |
|  |  | SNX3 |  |  |
|  |  | DCAF13 |  |  |
|  |  | IGFBP5 |  |  |
|  |  | 4933428G20RIK | |  |
|  |  | OTUD1 |  |  |
|  |  | EFEMP2 |  |  |
|  |  | ZCCHC18 | |  |
|  |  | 4931422A03RIK | |  |
|  |  | DXBAY18 | |  |
|  |  | METTL18 | |  |
|  |  | NYAP2 |  |  |
|  |  | ZFP428 |  |  |
|  |  | POMK |  |  |
|  |  | GAS2 |  |  |
|  |  | SDHAF2 |  |  |
|  |  | TCTEX1D1 | |  |
|  |  | CCDC92 |  |  |
|  |  | NKTR |  |  |
|  |  | ABCG5 |  |  |
|  |  | APRT |  |  |
|  |  | DHX16 |  |  |
|  |  | CKLF |  |  |
|  |  | SIMC1 |  |  |
|  |  | ARL4C |  |  |
|  |  | KBTBD8 |  |  |
|  |  | EIF3E |  |  |
|  |  | 4930415O20RIK | |  |
|  |  | DNLZ |  |  |
|  |  | SOX4 |  |  |
|  |  | PTER |  |  |
|  |  | CYSLTR2 | |  |
|  |  | OIT3 |  |  |
|  |  | SUCLG1 |  |  |
|  |  | MRE11A |  |  |
|  |  | CDC42EP3 | |  |
|  |  | JOSD2 |  |  |
|  |  | SAMHD1 |  |  |
|  |  | TBPL1 |  |  |
|  |  | GDPGP1 |  |  |
|  |  | C2CD4A |  |  |
|  |  | HSD3B5 |  |  |
|  |  | CD81 |  |  |
|  |  | HBEGF |  |  |
|  |  | UNC13C |  |  |
|  |  | 4921507P07RIK | |  |
|  |  | TRABD |  |  |
|  |  | REEP1 |  |  |
|  |  | C1QA |  |  |
|  |  | DPH6 |  |  |
|  |  | ZFP235 |  |  |
|  |  | LTBP2 |  |  |
|  |  | RAB5IF |  |  |
|  |  | HDHD3 |  |  |
|  |  | CASP3 |  |  |
|  |  | ASTN2 |  |  |
|  |  | PPP4R2 |  |  |
|  |  | PRKAB2 |  |  |
|  |  | LITAF |  |  |
|  |  | ARMC5 |  |  |
|  |  | NDUFS4 |  |  |
|  |  | RNF214 |  |  |
|  |  | BC028528 | |  |
|  |  | CASP16-PS | |  |
|  |  | RPRD1A |  |  |
|  |  | ADAT2 |  |  |
|  |  | TMEM259 | |  |
|  |  | TGM5 |  |  |
|  |  | GM10295 | |  |
|  |  | GLIPR1L3 | |  |
|  |  | LHFP |  |  |
|  |  | UROD |  |  |
|  |  | CPXM1 |  |  |
|  |  | LPCAT1 |  |  |
|  |  | HSPA1L |  |  |
|  |  | LPIN1 |  |  |
|  |  | GM28372 | |  |
|  |  | PPP1R1C | |  |
|  |  | RUFY3 |  |  |
|  |  | PAPSS1 |  |  |
|  |  | CCDC60 |  |  |
|  |  | CD27 |  |  |
|  |  | BOLA2 |  |  |
|  |  | ASH1L |  |  |
|  |  | SNTA1 |  |  |
|  |  | DEF8 |  |  |
|  |  | C1QL3 |  |  |
|  |  | NTNG2 |  |  |
|  |  | MLEC |  |  |
|  |  | ESRP2 |  |  |
|  |  | ORC6 |  |  |
|  |  | SLC44A4 |  |  |
|  |  | TIMM29 |  |  |
|  |  | PTPRM |  |  |
|  |  | HECA |  |  |
|  |  | L3MBTL2 | |  |
|  |  | MATN3 |  |  |
|  |  | AP2A2 |  |  |
|  |  | CCDC157 | |  |
|  |  | REST |  |  |
|  |  | DST |  |  |
|  |  | APOM |  |  |
|  |  | YRDC |  |  |
|  |  | KPTN |  |  |
|  |  | METHIG1 | |  |
|  |  | ZBTB41 |  |  |
|  |  | CABLES2 | |  |
|  |  | HMGB4 |  |  |
|  |  | RDH1 |  |  |
|  |  | NXPH1 |  |  |
|  |  | TNRC6A |  |  |
|  |  | HDDC2 |  |  |
|  |  | TIMM10B | |  |
|  |  | TMEM252 | |  |
|  |  | SLC26A9 |  |  |
|  |  | NLRC4 |  |  |
|  |  | VWF |  |  |
|  |  | PSMC2 |  |  |
|  |  | PDZK1IP1 | |  |
|  |  | HINT3 |  |  |
|  |  | CFAP70 |  |  |
|  |  | ADGRE1 |  |  |
|  |  | ACOT1 |  |  |
|  |  | PDZD11 |  |  |
|  |  | HIST1H3G | |  |
|  |  | CNDP2 |  |  |
|  |  | GSC2 |  |  |
|  |  | IGSF23 |  |  |
|  |  | CLEC2E |  |  |
|  |  | GM21834 | |  |
|  |  | DKK1 |  |  |
|  |  | OLFR117 |  |  |
|  |  | OAS1H |  |  |
|  |  | OLFR157 |  |  |
|  |  | OLFR1023 | |  |
|  |  | OLFR167 |  |  |
|  |  | OLFR1030 | |  |
|  |  | DLX3 |  |  |
|  |  | RNASE12 | |  |
|  |  | GM5294 |  |  |
|  |  | ETD |  |  |
|  |  | BPIFB3 |  |  |
|  |  | ADAM39 |  |  |
|  |  | SLC7A9 |  |  |
|  |  | NEUROG3 | |  |
|  |  | PRL7C1 |  |  |
|  |  | GM17728 | |  |
|  |  | OLFR784 |  |  |
|  |  | OLFR750 |  |  |
|  |  | OLFR800 |  |  |
|  |  | OLFR982 |  |  |
|  |  | DNAJC5G | |  |
|  |  | VMN2R88 | |  |
|  |  | APOL7C |  |  |
|  |  | VMN1R33 | |  |
|  |  | VMN1R23 | |  |
|  |  | VMN1R89 | |  |
|  |  | GM9507 |  |  |
|  |  | OLFR384 |  |  |
|  |  | OLFR1253 | |  |
|  |  | OLFR1258 | |  |
|  |  | OLFR433 |  |  |
|  |  | CXCR2 |  |  |
|  |  | GM8108 |  |  |
|  |  | OLFR592 |  |  |
|  |  | OLFR612 |  |  |
|  |  | CD300C |  |  |
|  |  | OLFR1331 | |  |
|  |  | OLFR1350 | |  |
|  |  | OLFR1387 | |  |
|  |  | OLFR1380 | |  |
|  |  | OLFR1382 | |  |
|  |  | 4833423E24RIK | |  |
|  |  | GIMAP7 |  |  |
|  |  | GM11009 | |  |
|  |  | GCSAM |  |  |
|  |  | SECTM1A | |  |
|  |  | SOHLH2 |  |  |
|  |  | SPDYE4C | |  |
|  |  | GM11168 | |  |
|  |  | IZUMO3 |  |  |
|  |  | MMP13 |  |  |
|  |  | REG2 |  |  |
|  |  | REG4 |  |  |
|  |  | CEACAM5 | |  |
|  |  | SULT1C1 | |  |
|  |  | PHLDA2 |  |  |
|  |  | ZFP872 |  |  |
|  |  | LY6G |  |  |
|  |  | DPPA2 |  |  |
|  |  | GM47289 | |  |
|  |  | UBE2U |  |  |
|  |  | VMN1R219 | |  |
|  |  | 4933421I07RIK | |  |
|  |  | SPEER2 |  |  |
|  |  | V1RA8 |  |  |
|  |  | TMED11 |  |  |
|  |  | KRT17 |  |  |
|  |  | GLYAT |  |  |
|  |  | TEX21 |  |  |
|  |  | MUP18 |  |  |
|  |  | GM3443 |  |  |
|  |  | SKOR2 |  |  |
|  |  | OBOX5 |  |  |
|  |  | SPINKL |  |  |
|  |  | SPINK2 |  |  |
|  |  | GOSR2 |  |  |
|  |  | WNT2 |  |  |
|  |  | FCRLA |  |  |
|  |  | LACTBL1 | |  |
|  |  | GM12185 | |  |
|  |  | APELA |  |  |
|  |  | SPINK4 |  |  |
|  |  | GMEB1 |  |  |
|  |  | GLYCTK | |  |
|  |  | ZFHX4 |  |  |
|  |  | CRYBG1 |  |  |
|  |  | QK |  |  |
|  |  | TUBA1C |  |  |
|  |  | NDUFS6 |  |  |
|  |  | SSX2IP |  |  |
|  |  | GALK1 |  |  |
|  |  | AI467606 |  |  |
|  |  | SURF4 |  |  |
|  |  | 4933415A04RIK | |  |
|  |  | FANCC |  |  |
|  |  | RAB12 |  |  |
|  |  | RNF151 |  |  |
|  |  | ZFP217 |  |  |
|  |  | ADCY3 |  |  |
|  |  | GRHL3 |  |  |
|  |  | FAM19A1 | |  |
|  |  | RHOA |  |  |
|  |  | STC1 |  |  |
|  |  | GM11808 | |  |
|  |  | ERICH2 |  |  |
|  |  | RAD51C |  |  |
|  |  | COL6A6 |  |  |
|  |  | BIRC6 |  |  |
|  |  | CCDC144B | |  |
|  |  | MTOR |  |  |
|  |  | AQP9 |  |  |
|  |  | PDGFRL |  |  |
|  |  | AHRR |  |  |
|  |  | RPL9 |  |  |
|  |  | SNX6 |  |  |
|  |  | ACADS |  |  |
|  |  | TARBP1 |  |  |
|  |  | PPP1R35 |  |  |
|  |  | CHDH |  |  |
|  |  | HIST1H4C | |  |
|  |  | NUDT14 |  |  |
|  |  | ADH4 |  |  |
|  |  | TMA16 |  |  |
|  |  | INSR |  |  |
|  |  | ADIG |  |  |
|  |  | LIPO1 |  |  |
|  |  | CUL3 |  |  |
|  |  | PLAT |  |  |
|  |  | PRPSAP1 | |  |
|  |  | AGBL3 |  |  |
|  |  | RIPK2 |  |  |
|  |  | TAS2R135 | |  |
|  |  | ADCY9 |  |  |
|  |  | OXLD1 |  |  |
|  |  | FCRL5 |  |  |
|  |  | PHAX |  |  |
|  |  | SNRNP200 | |  |
|  |  | FAM71F2 | |  |
|  |  | SREBF2 |  |  |
|  |  | SLC25A21 | |  |
|  |  | CILP |  |  |
|  |  | ITFG2 |  |  |
|  |  | SORCS3 |  |  |
|  |  | SSU2 |  |  |
|  |  | CYP4F37 |  |  |
|  |  | GPR1 |  |  |
|  |  | CDK10 |  |  |
|  |  | XPO7 |  |  |
|  |  | TNNC1 |  |  |
|  |  | HGH1 |  |  |
|  |  | SERPINA12 | |  |
|  |  | SECISBP2 | |  |
|  |  | METTL1 |  |  |
|  |  | GGACT |  |  |
|  |  | ABCA2 |  |  |
|  |  | SMIM10L2A | |  |
|  |  | RPP38 |  |  |
|  |  | OXTR |  |  |
|  |  | DNAH11 |  |  |
|  |  | HMCN2 |  |  |
|  |  | SCN1A |  |  |
|  |  | FAM122B | |  |
|  |  | SLC38A1 |  |  |
|  |  | MRPL12 |  |  |
|  |  | GPATCH2 | |  |
|  |  | METRNL | |  |
|  |  | SRPK3 |  |  |
|  |  | ORC1 |  |  |
|  |  | SERPINF2 | |  |
|  |  | PPP2R5C | |  |
|  |  | SETX |  |  |
|  |  | CRELD1 |  |  |
|  |  | ZFP983 |  |  |
|  |  | IL1RAPL2 | |  |
|  |  | LACTB2 |  |  |
|  |  | NFE2 |  |  |
|  |  | PRDM12 |  |  |
|  |  | SHARPIN | |  |
|  |  | STAP2 |  |  |
|  |  | KLHL24 |  |  |
|  |  | KLHDC3 |  |  |
|  |  | RBM15 |  |  |
|  |  | COX17 |  |  |
|  |  | GPR75 |  |  |
|  |  | DOK5 |  |  |
|  |  | CORO7 |  |  |
|  |  | DRC7 |  |  |
|  |  | H1FX |  |  |
|  |  | 4930442H23RIK | |  |
|  |  | SYT14 |  |  |
|  |  | SYCP3 |  |  |
|  |  | TBC1D4 |  |  |
|  |  | TRIM66 |  |  |
|  |  | GM11992 | |  |
|  |  | ZC3H13 |  |  |
|  |  | TCF25 |  |  |
|  |  | RPS3 |  |  |
|  |  | SRC |  |  |
|  |  | ADGRE5 |  |  |
|  |  | 2610042L04RIK | |  |
|  |  | RAD21 |  |  |
|  |  | SAMD4B |  |  |
|  |  | EPCAM |  |  |
|  |  | HNRNPK | |  |
|  |  | KDELC2 |  |  |
|  |  | RELT |  |  |
|  |  | FBXO6 |  |  |
|  |  | ERCC6 |  |  |
|  |  | GM11634 | |  |
|  |  | PRL |  |  |
|  |  | OLFR1344 | |  |
|  |  | GM3591 |  |  |
|  |  | A030014E15RIK | |  |
|  |  | SEC14L1 |  |  |
|  |  | SYPL2 |  |  |
|  |  | GM20715 | |  |
|  |  | TBC1D13 | |  |
|  |  | CLPP |  |  |
|  |  | GUCY1B2 | |  |
|  |  | ARMC2 |  |  |
|  |  | GMPS |  |  |
|  |  | 1600002K03RIK | |  |
|  |  | C1GALT1C1 | |  |
|  |  | LRCH3 |  |  |
|  |  | ELF2 |  |  |
|  |  | CDC42BPA | |  |
|  |  | PSEN1 |  |  |
|  |  | ITGAM |  |  |
|  |  | GM3173 |  |  |
|  |  | PMCH |  |  |
|  |  | THEMIS |  |  |
|  |  | DEPDC1A | |  |
|  |  | PLAC8L1 | |  |
|  |  | EYA3 |  |  |
|  |  | TWIST2 |  |  |
|  |  | KCND1 |  |  |
|  |  | MMEL1 |  |  |
|  |  | NUP54 |  |  |
|  |  | DUSP18 |  |  |
|  |  | BACE1 |  |  |
|  |  | CDON |  |  |
|  |  | C3 |  |  |
|  |  | PTGS1 |  |  |
|  |  | ILDR1 |  |  |
|  |  | TRIM17 |  |  |
|  |  | XKR4 |  |  |
|  |  | SPAG1 |  |  |
|  |  | ANG |  |  |
|  |  | CDC37 |  |  |
|  |  | PAIP2 |  |  |
|  |  | TMEM74B | |  |
|  |  | CSGALNACT2 | |  |
|  |  | RPL24 |  |  |
|  |  | TNFRSF14 | |  |
|  |  | NRM |  |  |
|  |  | CCDC115 | |  |
|  |  | FRRS1 |  |  |
|  |  | MYLK3 |  |  |
|  |  | PMP22 |  |  |
|  |  | RGP1 |  |  |
|  |  | DOLK |  |  |
|  |  | RIPPLY2 |  |  |
|  |  | EGFL7 |  |  |
|  |  | LANCL2 |  |  |
|  |  | TTC30A1 | |  |
|  |  | PEPD |  |  |
|  |  | XKR7 |  |  |
|  |  | LARGE2 |  |  |
|  |  | DYNC1LI1 | |  |
|  |  | CREB3L3 | |  |
|  |  | MASTL |  |  |
|  |  | CYP4F16 |  |  |
|  |  | NOM1 |  |  |
|  |  | STIP1 |  |  |
|  |  | GNA15 |  |  |
|  |  | SLCO2B1 | |  |
|  |  | SCARA5 |  |  |
|  |  | LARGE1 |  |  |
|  |  | TMEM268 | |  |
|  |  | CTXN2 |  |  |
|  |  | SLC26A11 | |  |
|  |  | GM6871 |  |  |
|  |  | GM6408 |  |  |
|  |  | AQP6 |  |  |
|  |  | CES4A |  |  |
|  |  | FAM229A | |  |
|  |  | AKR1C12 | |  |
|  |  | CAV3 |  |  |
|  |  | LRTM1 |  |  |
|  |  | C2CD4B |  |  |
|  |  | THG1L |  |  |
|  |  | ACTL9 |  |  |
|  |  | ACBD7 |  |  |
|  |  | CCDC180 | |  |
|  |  | UBXN2B |  |  |
|  |  | FOSL1 |  |  |
|  |  | HK3 |  |  |
|  |  | INSYN1 |  |  |
|  |  | MKS1 |  |  |
|  |  | TLN2 |  |  |
|  |  | GPR180 |  |  |
|  |  | KIF14 |  |  |
|  |  | EXOC1 |  |  |
|  |  | NSMCE2 |  |  |
|  |  | MAPK14 |  |  |
|  |  | CDC27 |  |  |
|  |  | HERC1 |  |  |
|  |  | EZH2 |  |  |
|  |  | ALX3 |  |  |
|  |  | SNAI2 |  |  |
|  |  | TRIB1 |  |  |
|  |  | TLK1 |  |  |
|  |  | STXBP2 |  |  |
|  |  | CTSO |  |  |
|  |  | SRSF2 |  |  |
|  |  | FAM149B | |  |
|  |  | KIF23 |  |  |
|  |  | MADD |  |  |
|  |  | FAM84B |  |  |
|  |  | NDST3 |  |  |
|  |  | ABL2 |  |  |
|  |  | SCARB2 |  |  |
|  |  | FCHO1 |  |  |
|  |  | TDGF1 |  |  |
|  |  | FRG1 |  |  |
|  |  | DMRTC1A | |  |
|  |  | SLC25A43 | |  |
|  |  | BTD |  |  |
|  |  | POT1A |  |  |
|  |  | CCDC130 | |  |
|  |  | KCNJ5 |  |  |
|  |  | STAT5A |  |  |
|  |  | D130040H23RIK | |  |
|  |  | TTC29 |  |  |
|  |  | FGFRL1 |  |  |
|  |  | APOBEC3 | |  |
|  |  | GM9925 |  |  |
|  |  | MED29 |  |  |
|  |  | SLC25A26 | |  |
|  |  | SRSF11 |  |  |
|  |  | MYO15B |  |  |
|  |  | HIST1H2BM | |  |
|  |  | MBTPS2 |  |  |
|  |  | GM17545 | |  |
|  |  | ZFP972 |  |  |
|  |  | IGIP |  |  |
|  |  | SPACA1 |  |  |
|  |  | TTC21A |  |  |
|  |  | GLIS1 |  |  |
|  |  | GM12166 | |  |
|  |  | ATXN2L |  |  |
|  |  | CHRNB4 |  |  |
|  |  | TRPM5 |  |  |
|  |  | CCP110 |  |  |
|  |  | FOXM1 |  |  |
|  |  | DOCK7 |  |  |
|  |  | PHF12 |  |  |
|  |  | MAPK15 |  |  |
|  |  | FANCE |  |  |
|  |  | WARS |  |  |
|  |  | HMGB3 |  |  |
|  |  | PNPLA2 |  |  |
|  |  | MAN2A2 |  |  |
|  |  | KCTD17 |  |  |
|  |  | RBPMS |  |  |
|  |  | CAR14 |  |  |
|  |  | PLBD2 |  |  |
|  |  | ASAP2 |  |  |
|  |  | HUS1 |  |  |
|  |  | SDSL |  |  |
|  |  | CNTN5 |  |  |
|  |  | SMIM17 |  |  |
|  |  | TUBA4A |  |  |
|  |  | ASPSCR1 | |  |
|  |  | VEGFD |  |  |
|  |  | KRT10 |  |  |
|  |  | CASP8 |  |  |
|  |  | 2810459M11RIK | |  |
|  |  | CTDSP1 |  |  |
|  |  | POLA1 |  |  |
|  |  | SPINDOC | |  |
|  |  | SNAPC2 |  |  |
|  |  | IL4RA |  |  |
|  |  | ZMYM2 |  |  |
|  |  | S100A13 |  |  |
|  |  | FABP5 |  |  |
|  |  | SPECC1L | |  |
|  |  | ZBTB39 |  |  |
|  |  | DYNLL1 |  |  |
|  |  | SNX25 |  |  |
|  |  | UCHL5 |  |  |
|  |  | EEPD1 |  |  |
|  |  | UPF2 |  |  |
|  |  | EPM2AIP1 | |  |
|  |  | MICU1 |  |  |
|  |  | NSL1 |  |  |
|  |  | ITGA3 |  |  |
|  |  | OLFR1564 | |  |
|  |  | CLRN2 |  |  |
|  |  | SOS2 |  |  |
|  |  | FLRT3 |  |  |
|  |  | CLDN23 |  |  |
|  |  | CLYBL |  |  |
|  |  | KITL |  |  |
|  |  | GGA1 |  |  |
|  |  | GM11437 | |  |
|  |  | MYBBP1A | |  |
|  |  | IGSF9 |  |  |
|  |  | TM9SF1 |  |  |
|  |  | PPOX |  |  |
|  |  | EIF5A |  |  |
|  |  | SYT13 |  |  |
|  |  | DECR2 |  |  |
|  |  | BAD |  |  |
|  |  | THYN1 |  |  |
|  |  | MAN2B1 |  |  |
|  |  | IMP4 |  |  |
|  |  | SMOX |  |  |
|  |  | EID2B |  |  |
|  |  | WRAP73 |  |  |
|  |  | ADA |  |  |
|  |  | SOD3 |  |  |
|  |  | RNF2 |  |  |
|  |  | FAF2 |  |  |
|  |  | RNF10 |  |  |
|  |  | GEMIN6 |  |  |
|  |  | GSKIP |  |  |
|  |  | 3110040N11RIK | |  |
|  |  | UBE2T |  |  |
|  |  | TRIM21 |  |  |
|  |  | 4930502E18RIK | |  |
|  |  | RNASE1 |  |  |
|  |  | ERRFI1 |  |  |
|  |  | SPC24 |  |  |
|  |  | CRTC3 |  |  |
|  |  | HIST1H2BN | |  |
|  |  | CHST12 |  |  |
|  |  | SVIL |  |  |
|  |  | XPO5 |  |  |
|  |  | TEAD3 |  |  |
|  |  | SMG8 |  |  |
|  |  | TMA7 |  |  |
|  |  | LIPO2 |  |  |
|  |  | ATP8B5 |  |  |
|  |  | LBHD1 |  |  |
|  |  | BCL11A |  |  |
|  |  | COL15A1 | |  |
|  |  | TBCEL |  |  |
|  |  | UBE2C |  |  |
|  |  | AIF1L |  |  |
|  |  | CTPS |  |  |
|  |  | TMEM2 |  |  |
|  |  | L3MBTL3 | |  |
|  |  | DUSP26 |  |  |
|  |  | ABCA12 |  |  |
|  |  | HYOU1 |  |  |
|  |  | SRRT |  |  |
|  |  | R3HDM1 |  |  |
|  |  | ACAA2 |  |  |
|  |  | AHR |  |  |
|  |  | PRC1 |  |  |
|  |  | TEN1 |  |  |
|  |  | GM17949 | |  |
|  |  | SLC25A39 | |  |
|  |  | SLC9A1 |  |  |
|  |  | IFI211 |  |  |
|  |  | VMN1R31 | |  |
|  |  | KLHDC10 | |  |
|  |  | KY |  |  |
|  |  | PTDSS1 |  |  |
|  |  | GPR27 |  |  |
|  |  | STYK1 |  |  |
|  |  | PIK3C2A |  |  |
|  |  | GK2 |  |  |
|  |  | MYO5C |  |  |
|  |  | CDC6 |  |  |
|  |  | VDAC2 |  |  |
|  |  | MBNL3 |  |  |
|  |  | BORCS7 |  |  |
|  |  | ANKRD54 | |  |
|  |  | MFSD10 |  |  |
|  |  | BCKDHB | |  |
|  |  | VRK3 |  |  |
|  |  | TRAPPC9 | |  |
|  |  | GKN3 |  |  |
|  |  | HMGA2 |  |  |
|  |  | RPL21 |  |  |
|  |  | PP2D1 |  |  |
|  |  | ITPA |  |  |
|  |  | APPL1 |  |  |
|  |  | ANKRD9 |  |  |
|  |  | NLGN1 |  |  |
|  |  | ZC3H11A | |  |
|  |  | OLFR671 |  |  |
|  |  | OLFR686 |  |  |
|  |  | TFPI2 |  |  |
|  |  | FAM19A4 | |  |
|  |  | SGCB |  |  |
|  |  | IGF1 |  |  |
|  |  | GALNT6 |  |  |
|  |  | BTBD7 |  |  |
|  |  | RIBC2 |  |  |
|  |  | SPRED3 |  |  |
|  |  | ATP5G2 |  |  |
|  |  | SLC6A1 |  |  |
|  |  | CAR7 |  |  |
|  |  | GM16253 | |  |
|  |  | HMG20B |  |  |
|  |  | ZP3R |  |  |
|  |  | HIST2H2AA1 | |  |
|  |  | PPIG |  |  |
|  |  | PNPO |  |  |
|  |  | CYP2R1 |  |  |
|  |  | CBR2 |  |  |
|  |  | MAP3K14 | |  |
|  |  | TCTN1 |  |  |
|  |  | SELENOV | |  |
|  |  | GOLGA2 |  |  |
|  |  | ITPRIPL1 | |  |
|  |  | UTP11 |  |  |
|  |  | ATP5K |  |  |
|  |  | EXT2 |  |  |
|  |  | AKR7A5 |  |  |
|  |  | FAM222B | |  |
|  |  | SLC25A13 | |  |
|  |  | CCDC170 | |  |
|  |  | BBS9 |  |  |
|  |  | GM20449 | |  |
|  |  | TRIP6 |  |  |
|  |  | MEF2B |  |  |
|  |  | NDUFS5 |  |  |
|  |  | NUP98 |  |  |
|  |  | FAR2 |  |  |
|  |  | GM3667 |  |  |
|  |  | ADRA2C |  |  |
|  |  | IDNK |  |  |
|  |  | ZFP655 |  |  |
|  |  | PIGM |  |  |
|  |  | DAXX |  |  |
|  |  | ECM1 |  |  |
|  |  | RDM1 |  |  |
|  |  | ZFP40 |  |  |
|  |  | 2310033P09RIK | |  |
|  |  | ATG7 |  |  |
|  |  | H2-DMB1 | |  |
|  |  | UBOX5 |  |  |
|  |  | MYADML2 | |  |
|  |  | MCIDAS |  |  |
|  |  | GPAM |  |  |
|  |  | 2610524H06RIK | |  |
|  |  | GNPTAB |  |  |
|  |  | BPHL |  |  |
|  |  | POGK |  |  |
|  |  | RPS9 |  |  |
|  |  | ADCY10 |  |  |
|  |  | JMJD6 |  |  |
|  |  | BRAP |  |  |
|  |  | NKAIN1 |  |  |
|  |  | TBX4 |  |  |
|  |  | WT1 |  |  |
|  |  | ZFP330 |  |  |
|  |  | FXR1 |  |  |
|  |  | TMEM25 |  |  |
|  |  | SORT1 |  |  |
|  |  | FCRLS |  |  |
|  |  | SLU7 |  |  |
|  |  | PSD2 |  |  |
|  |  | EEF1B2 |  |  |
|  |  | CHIC2 |  |  |
|  |  | ASPM |  |  |
|  |  | CYP8B1 |  |  |
|  |  | TERF1 |  |  |
|  |  | ABCG8 |  |  |
|  |  | PHGDH |  |  |
|  |  | ZFP959 |  |  |
|  |  | EXOSC10 | |  |
|  |  | RPL8 |  |  |
|  |  | JCHAIN |  |  |
|  |  | TXNIP |  |  |
|  |  | CFAP20 |  |  |
|  |  | GPR153 |  |  |
|  |  | BCO1 |  |  |
|  |  | FRMD7 |  |  |
|  |  | TMEM159 | |  |
|  |  | COLGALT2 | |  |
|  |  | SMPD1 |  |  |
|  |  | NPM1 |  |  |
|  |  | TMED6 |  |  |
|  |  | SYNGR4 |  |  |
|  |  | LRP2 |  |  |
|  |  | LAMB3 |  |  |
|  |  | FAM151B | |  |
|  |  | TNFSF10 |  |  |
|  |  | LRRC45 |  |  |
|  |  | VPS28 |  |  |
|  |  | PLEKHA1 | |  |
|  |  | CASZ1 |  |  |
|  |  | GM21976 | |  |
|  |  | PSMD11 |  |  |
|  |  | 1700028K03RIK | |  |
|  |  | SCUBE3 |  |  |
|  |  | DLEC1 |  |  |
|  |  | D630003M21RIK | |  |
|  |  | CORO2A |  |  |
|  |  | POMT2 |  |  |
|  |  | OSR1 |  |  |
|  |  | OLFR33 |  |  |
|  |  | HBQ1A |  |  |
|  |  | RRNAD1 |  |  |
|  |  | ARL2 |  |  |
|  |  | SLC25A12 | |  |
|  |  | EXOC6 |  |  |
|  |  | SLC12A8 |  |  |
|  |  | SNRPD2 |  |  |
|  |  | ZFP317 |  |  |
|  |  | MAP3K12 | |  |
|  |  | TLDC1 |  |  |
|  |  | RXFP4 |  |  |
|  |  | ETNK1 |  |  |
|  |  | ARL10 |  |  |
|  |  | STXBP3 |  |  |
|  |  | UCN2 |  |  |
|  |  | ZFP981 |  |  |
|  |  | SYCP2L |  |  |
|  |  | CCDC91 |  |  |
|  |  | GCAT |  |  |
|  |  | STAT5B |  |  |
|  |  | GGPS1 |  |  |
|  |  | MKLN1 |  |  |
|  |  | GM49486 | |  |
|  |  | ZC4H2 |  |  |
|  |  | REXO4 |  |  |
|  |  | PGAM1 |  |  |
|  |  | SGMS1 |  |  |
|  |  | ADAMTSL2 | |  |
|  |  | 5730455P16RIK | |  |
|  |  | PRPF4 |  |  |
|  |  | GMNN |  |  |
|  |  | DHX37 |  |  |
|  |  | EFCAB14 | |  |
|  |  | GSPT1 |  |  |
|  |  | TMEM14C | |  |
|  |  | SCD1 |  |  |
|  |  | FAM117B | |  |
|  |  | PHKA2 |  |  |
|  |  | APLP1 |  |  |
|  |  | ADHFE1 |  |  |
|  |  | SUV39H2 | |  |
|  |  | FEZ1 |  |  |
|  |  | TTC17 |  |  |
|  |  | PPP2R5D | |  |
|  |  | RDH9 |  |  |
|  |  | PLD5 |  |  |
|  |  | ZDHHC6 |  |  |
|  |  | SRL |  |  |
|  |  | SLC26A3 |  |  |
|  |  | CISD2 |  |  |
|  |  | PTPN20 |  |  |
|  |  | NTSR1 |  |  |
|  |  | IFNGR1 |  |  |
|  |  | ESD |  |  |
|  |  | CRABP1 |  |  |
|  |  | RAB26 |  |  |
|  |  | GABBR2 |  |  |
|  |  | QRICH2 |  |  |
|  |  | MATN2 |  |  |
|  |  | ALDOC |  |  |
|  |  | ZFP541 |  |  |
|  |  | GJD2 |  |  |
|  |  | RAB5B |  |  |
|  |  | PRR5 |  |  |
|  |  | EEF1AKMT3 | |  |
|  |  | PLVAP |  |  |
|  |  | ANGPTL6 | |  |
|  |  | LNPEP |  |  |
|  |  | NEU1 |  |  |
|  |  | TSPAN4 |  |  |
|  |  | GM4787 |  |  |
|  |  | MYO3A |  |  |
|  |  | GM12117 | |  |
|  |  | RGS17 |  |  |
|  |  | CCDC191 | |  |
|  |  | SRRD |  |  |
|  |  | LRRC51 |  |  |
|  |  | CYP24A1 | |  |
|  |  | TPTE |  |  |
|  |  | NLRP10 |  |  |
|  |  | TASP1 |  |  |
|  |  | OLFR31 |  |  |
|  |  | CNKSR1 |  |  |
|  |  | EVL |  |  |
|  |  | ZFP27 |  |  |
|  |  | SDR42E1 |  |  |
|  |  | SIX5 |  |  |
|  |  | CLPB |  |  |
|  |  | IL1RL1 |  |  |
|  |  | HIST1H2AI | |  |
|  |  | ZFP553 |  |  |
|  |  | DCAF1 |  |  |
|  |  | CMTM3 |  |  |
|  |  | PHF23 |  |  |
|  |  | RPL37A |  |  |
|  |  | PEX13 |  |  |
|  |  | RPS19 |  |  |
|  |  | DND1 |  |  |
|  |  | FAM71B |  |  |
|  |  | CNR1 |  |  |
|  |  | BC049352 | |  |
|  |  | DIRAS1 |  |  |
|  |  | ANGPTL8 | |  |
|  |  | UQCC3 |  |  |
|  |  | SPATA31D1B | |  |
|  |  | TAL2 |  |  |
|  |  | TUFT1 |  |  |
|  |  | TFPI |  |  |
|  |  | SPATA19 | |  |
|  |  | RRAD |  |  |
|  |  | ADAM4 |  |  |
|  |  | MRPS28 |  |  |
|  |  | ZFP341 |  |  |
|  |  | KIF7 |  |  |
|  |  | SPDL1 |  |  |
|  |  | OVCA2 |  |  |
|  |  | MIS12 |  |  |
|  |  | FAM217A | |  |
|  |  | TTC30B |  |  |
|  |  | RLN1 |  |  |
|  |  | 5330417C22RIK | |  |
|  |  | CHRM3 |  |  |
|  |  | ABCB10 |  |  |
|  |  | SYN1 |  |  |
|  |  | TMEM138 | |  |
|  |  | CENPN |  |  |
|  |  | RPL7L1 |  |  |
|  |  | CHMP4B |  |  |
|  |  | ADRA2B |  |  |
|  |  | USP49 |  |  |
|  |  | KIFC1 |  |  |
|  |  | MAP3K21 | |  |
|  |  | TOM1 |  |  |
|  |  | A630001G21RIK | |  |
|  |  | GJA6 |  |  |
|  |  | TUBB6 |  |  |
|  |  | GYS2 |  |  |
|  |  | FAM120A | |  |
|  |  | EXO5 |  |  |
|  |  | PPFIBP2 |  |  |
|  |  | MGAT3 |  |  |
|  |  | PMS1 |  |  |
|  |  | SHF |  |  |
|  |  | FAM227B | |  |
|  |  | KCNH2 |  |  |
|  |  | ZFP773 |  |  |
|  |  | NSG2 |  |  |
|  |  | MID1 |  |  |
|  |  | TCP10B |  |  |
|  |  | KCNA5 |  |  |
|  |  | SLFN4 |  |  |
|  |  | CFAP221 |  |  |
|  |  | TRIM59 |  |  |
|  |  | REM2 |  |  |
|  |  | FYTTD1 |  |  |
|  |  | HELQ |  |  |
|  |  | 2310022A10RIK | |  |
|  |  | BTBD19 |  |  |
|  |  | KCTD16 |  |  |
|  |  | MYO9B |  |  |
|  |  | RBKS |  |  |
|  |  | PAXBP1 |  |  |
|  |  | CEP68 |  |  |
|  |  | ZNRF1 |  |  |
|  |  | RAB7B |  |  |
|  |  | NRARP |  |  |
|  |  | VMA21 |  |  |
|  |  | FDFT1 |  |  |
|  |  | OSGIN1 |  |  |
|  |  | GM960 |  |  |
|  |  | RPAP2 |  |  |
|  |  | PATE2 |  |  |
|  |  | ATP8B2 |  |  |
|  |  | PFAS |  |  |
|  |  | SRP72 |  |  |
|  |  | CDK11B |  |  |
|  |  | FGD6 |  |  |
|  |  | CCM2L |  |  |
|  |  | CPXM2 |  |  |
|  |  | TYRP1 |  |  |
|  |  | 5330417H12RIK | |  |
|  |  | 4930505A04RIK | |  |
|  |  | TIGD5 |  |  |
|  |  | ACSBG1 |  |  |
|  |  | CDK18 |  |  |
|  |  | SOCS2 |  |  |
|  |  | 1700093K21RIK | |  |
|  |  | PXDN |  |  |
|  |  | CCNE1 |  |  |
|  |  | BDP1 |  |  |
|  |  | PTGER2 |  |  |
|  |  | COPZ1 |  |  |
|  |  | CLDND1 |  |  |
|  |  | PGAP3 |  |  |
|  |  | GULP1 |  |  |
|  |  | ATP6V1G1 | |  |
|  |  | PIK3C3 |  |  |
|  |  | OMP |  |  |
|  |  | WIPI1 |  |  |
|  |  | ENDOD1 |  |  |
|  |  | GSK3B |  |  |
|  |  | CASQ2 |  |  |
|  |  | CNPY2 |  |  |
|  |  | COL17A1 | |  |
|  |  | ARHGAP35 | |  |
|  |  | 9-Sep |  |  |
|  |  | CABCOCO1 | |  |
|  |  | PSKH1 |  |  |
|  |  | ZFP90 |  |  |
|  |  | PCBD1 |  |  |
|  |  | CD151 |  |  |
|  |  | DNTTIP1 | |  |
|  |  | CHRNB3 |  |  |
|  |  | 1700001O22RIK | |  |
|  |  | BET1L |  |  |
|  |  | VMN1R4 |  |  |
|  |  | CALCA |  |  |
|  |  | POU4F1 |  |  |
|  |  | ZFP78 |  |  |
|  |  | CDK4 |  |  |
|  |  | GM2174 |  |  |
|  |  | PON2 |  |  |
|  |  | 2810006K23RIK | |  |
|  |  | GXYLT1 |  |  |
|  |  | HC |  |  |
|  |  | MAP3K11 | |  |
|  |  | P2RX7 |  |  |
|  |  | OLFR78 |  |  |
|  |  | SOCS6 |  |  |
|  |  | AP3B2 |  |  |
|  |  | OTOF |  |  |
|  |  | SLC6A6 |  |  |
|  |  | 2610507B11RIK | |  |
|  |  | OSBP |  |  |
|  |  | AGGF1 |  |  |
|  |  | KPNA1 |  |  |
|  |  | LRRC59 |  |  |
|  |  | SMIM8 |  |  |
|  |  | AOX3 |  |  |
|  |  | GLB1 |  |  |
|  |  | ORAI1 |  |  |
|  |  | PARP1 |  |  |
|  |  | RTN1 |  |  |
|  |  | LVRN |  |  |
|  |  | BECN1 |  |  |
|  |  | DSPP |  |  |
|  |  | MCRIP2 |  |  |
|  |  | TARS2 |  |  |
|  |  | B230307C23RIK | |  |
|  |  | SLC5A8 |  |  |
|  |  | TMEM42 |  |  |
|  |  | 4-Mar |  |  |
|  |  | TRIM30B | |  |
|  |  | STMN4 |  |  |
|  |  | ARNT |  |  |
|  |  | OSBPL8 |  |  |
|  |  | GPRC5A |  |  |
|  |  | C1QTNF6 | |  |
|  |  | REP15 |  |  |
|  |  | GPR89 |  |  |
|  |  | MAP2K3 |  |  |
|  |  | TMEM168 | |  |
|  |  | INPP5K |  |  |
|  |  | SERPINA3F | |  |
|  |  | DCAF12L1 | |  |
|  |  | P4HA2 |  |  |
|  |  | CCDC42 |  |  |
|  |  | RMND5A | |  |
|  |  | CBFB |  |  |
|  |  | PEMT |  |  |
|  |  | LOX |  |  |
|  |  | EVA1A |  |  |
|  |  | MXRA7 |  |  |
|  |  | NAXE |  |  |
|  |  | TTLL6 |  |  |
|  |  | PARD6G |  |  |
|  |  | CAR9 |  |  |
|  |  | PHLDA3 |  |  |
|  |  | SPNS1 |  |  |
|  |  | KHDRBS3 | |  |
|  |  | KATNAL2 | |  |
|  |  | PRELID3B | |  |
|  |  | TTK |  |  |
|  |  | VKORC1L1 | |  |
|  |  | ANKS3 |  |  |
|  |  | GCH1 |  |  |
|  |  | PGM5 |  |  |
|  |  | CFAP77 |  |  |
|  |  | PLA2G2D | |  |
|  |  | EVA1B |  |  |
|  |  | LASP1 |  |  |
|  |  | ZFP654 |  |  |
|  |  | ZFP408 |  |  |
|  |  | PPIC |  |  |
|  |  | DEPDC7 |  |  |
|  |  | RGS3 |  |  |
|  |  | RAE1 |  |  |
|  |  | CLINT1 |  |  |
|  |  | EGLN2 |  |  |
|  |  | FLNB |  |  |
|  |  | PHB2 |  |  |
|  |  | MTHFR |  |  |
|  |  | ERBB2 |  |  |
|  |  | GM14698 | |  |
|  |  | HDX |  |  |
|  |  | GM45871 | |  |
|  |  | SPINK7 |  |  |
|  |  | OLFR317 |  |  |
|  |  | TAF2 |  |  |
|  |  | FAM69C |  |  |
|  |  | FBXL20 |  |  |
|  |  | GALNT16 | |  |
|  |  | NDP |  |  |
|  |  | ERMARD | |  |
|  |  | FTO |  |  |
|  |  | GM10521 | |  |
|  |  | STRA6 |  |  |
|  |  | PCSK4 |  |  |
|  |  | TMPRSS5 | |  |
|  |  | RELB |  |  |
|  |  | TEDDM3 | |  |
|  |  | MEX3B |  |  |
|  |  | BLVRB |  |  |
|  |  | NPRL3 |  |  |
|  |  | SLC2A2 |  |  |
|  |  | ABCD4 |  |  |
|  |  | PRAF2 |  |  |
|  |  | MYOD1 |  |  |
|  |  | GM21738 | |  |
|  |  | ZFP112 |  |  |
|  |  | NOVA1 |  |  |
|  |  | ATF2 |  |  |
|  |  | KRT77 |  |  |
|  |  | TRIM39 |  |  |
|  |  | CYP2A5 |  |  |
|  |  | RWDD1 |  |  |
|  |  | FAR1 |  |  |
|  |  | SLC1A4 |  |  |
|  |  | SP8 |  |  |
|  |  | 4930404H24RIK | |  |
|  |  | NQO2 |  |  |
|  |  | HDAC5 |  |  |
|  |  | PCED1B |  |  |
|  |  | APTX |  |  |
|  |  | CFAP61 |  |  |
|  |  | ZFP938 |  |  |
|  |  | SPATA1 |  |  |
|  |  | 9430015G10RIK | |  |
|  |  | IAH1 |  |  |
|  |  | ETNPPL |  |  |
|  |  | FHL3 |  |  |
|  |  | MTERF3 |  |  |
|  |  | GADD45G | |  |
|  |  | CTCF |  |  |
|  |  | ERN1 |  |  |
|  |  | SLC38A10 | |  |
|  |  | UPK1B |  |  |
|  |  | HNRNPUL2 | |  |
|  |  | RAI14 |  |  |
|  |  | G6PC3 |  |  |
|  |  | 1700064H15RIK | |  |
|  |  | RBBP4 |  |  |
|  |  | PTTG1 |  |  |
|  |  | DENND4C | |  |
|  |  | TRPV4 |  |  |
|  |  | SUPT4A |  |  |
|  |  | CDK2 |  |  |
|  |  | CADM3 |  |  |
|  |  | CENPE |  |  |
|  |  | GLA |  |  |
|  |  | CDCP1 |  |  |
|  |  | MRPL32 |  |  |
|  |  | GM3029 |  |  |
|  |  | LCTL |  |  |
|  |  | RAET1D |  |  |
|  |  | DOHH |  |  |
|  |  | SRP54A |  |  |
|  |  | CHSY1 |  |  |
|  |  | REG3A |  |  |
|  |  | TXNDC5 |  |  |
|  |  | PARVB |  |  |
|  |  | 1700020N01RIK | |  |
|  |  | RECQL4 |  |  |
|  |  | CCDC121 | |  |
|  |  | CDCA8 |  |  |
|  |  | PORCN |  |  |
|  |  | S1PR3 |  |  |
|  |  | COG2 |  |  |
|  |  | AP1G1 |  |  |
|  |  | SCAPER |  |  |
|  |  | QTRT1 |  |  |
|  |  | UHRF1 |  |  |
|  |  | DGAT2 |  |  |
|  |  | NPFF |  |  |
|  |  | SLC35F1 |  |  |
|  |  | ERI3 |  |  |
|  |  | ALG1 |  |  |
|  |  | FSCN2 |  |  |
|  |  | SRM |  |  |
|  |  | HSBP1 |  |  |
|  |  | AKAP11 |  |  |
|  |  | TUBB3 |  |  |
|  |  | LAMTOR3 | |  |
|  |  | RHOT1 |  |  |
|  |  | SHISAL2B | |  |
|  |  | BEGAIN |  |  |
|  |  | RNF145 |  |  |
|  |  | AASS |  |  |
|  |  | ZFP358 |  |  |
|  |  | NSFL1C |  |  |
|  |  | 2310061I04RIK | |  |
|  |  | LIMCH1 |  |  |
|  |  | TANK |  |  |
|  |  | GM14393 | |  |
|  |  | WARS2 |  |  |
|  |  | EVPL |  |  |
|  |  | INTS13 |  |  |
|  |  | NLRP3 |  |  |
|  |  | PSMC5 |  |  |
|  |  | CLDN3 |  |  |
|  |  | STARD3NL | |  |
|  |  | GM9833 |  |  |
|  |  | 2300009A05RIK | |  |
|  |  | MPHOSPH6 | |  |
|  |  | HSPG2 |  |  |
|  |  | ETF1 |  |  |
|  |  | FAM183B | |  |
|  |  | CCDC86 |  |  |
|  |  | PF4 |  |  |
|  |  | ZFP472 |  |  |
|  |  | CD2AP |  |  |
|  |  | PXMP4 |  |  |
|  |  | SYNC |  |  |
|  |  | RGS11 |  |  |
|  |  | RTEL1 |  |  |
|  |  | PCARE |  |  |
|  |  | RAD51D |  |  |
|  |  | ART3 |  |  |
|  |  | DNAJC18 | |  |
|  |  | KRI1 |  |  |
|  |  | DIABLO |  |  |
|  |  | ZFP454 |  |  |
|  |  | KPNB1 |  |  |
|  |  | DDIT3 |  |  |
|  |  | HADHB |  |  |
|  |  | ELAC1 |  |  |
|  |  | ZFP82 |  |  |
|  |  | MIDN |  |  |
|  |  | GPI1 |  |  |
|  |  | SMNDC1 |  |  |
|  |  | LGALS4 |  |  |
|  |  | IRAK2 |  |  |
|  |  | CEP44 |  |  |
|  |  | CYP4F17 |  |  |
|  |  | ANKRD27 | |  |
|  |  | ZFP111 |  |  |
|  |  | GABRR3 |  |  |
|  |  | NFU1 |  |  |
|  |  | LHFPL1 |  |  |
|  |  | MARCKS | |  |
|  |  | MYL12A |  |  |
|  |  | B3GALT1 | |  |
|  |  | TRAPPC3 | |  |
|  |  | CYP4F18 |  |  |
|  |  | FBXO38 |  |  |
|  |  | ACSS2 |  |  |
|  |  | ZFP512 |  |  |
|  |  | BUD31 |  |  |
|  |  | FAM234A | |  |
|  |  | PTBP1 |  |  |
|  |  | INO80C |  |  |
|  |  | THAP4 |  |  |
|  |  | ACAT3 |  |  |
|  |  | DCK |  |  |
|  |  | PLATR17 | |  |
|  |  | NFKBIL1 | |  |
|  |  | APH1C |  |  |
|  |  | PPP6C |  |  |
|  |  | MOB4 |  |  |
|  |  | BAG1 |  |  |
|  |  | BRF2 |  |  |
|  |  | SCGB1C1 | |  |
|  |  | UBA1 |  |  |
|  |  | CCDC62 |  |  |
|  |  | METTL2 |  |  |
|  |  | COPS9 |  |  |
|  |  | GM10762 | |  |
|  |  | MAGOH |  |  |
|  |  | PERM1 |  |  |
|  |  | GM37240 | |  |
|  |  | SPDYA |  |  |
|  |  | SLC36A2 |  |  |
|  |  | ACADSB |  |  |
|  |  | TBC1D32 | |  |
|  |  | AAGAB |  |  |
|  |  | CNOT4 |  |  |
|  |  | NEURL1B | |  |
|  |  | MFSD2B |  |  |
|  |  | HIST1H2BH | |  |
|  |  | ZDHHC13 | |  |
|  |  | AGAP1 |  |  |
|  |  | BLOC1S1 | |  |
|  |  | SGSM2 |  |  |
|  |  | NRF1 |  |  |
|  |  | GM266 |  |  |
|  |  | PGPEP1L | |  |
|  |  | SMYD1 |  |  |
|  |  | ECHDC3 |  |  |
|  |  | DNAJC1 |  |  |
|  |  | CORT |  |  |
|  |  | MB21D1 |  |  |
|  |  | CAPN2 |  |  |
|  |  | KCTD7 |  |  |
|  |  | FAM189B | |  |
|  |  | OLFR99 |  |  |
|  |  | TXNDC9 |  |  |
|  |  | MICU2 |  |  |
|  |  | GM21188 | |  |
|  |  | COPS3 |  |  |
|  |  | TMEM208 | |  |
|  |  | RGS1 |  |  |
|  |  | ZWINT |  |  |
|  |  | UNC13D |  |  |
|  |  | GAB3 |  |  |
|  |  | ZKSCAN7 | |  |
|  |  | ZFP184 |  |  |
|  |  | SELENOO | |  |
|  |  | PRR3 |  |  |
|  |  | THSD1 |  |  |
|  |  | CHMP1A |  |  |
|  |  | GPR179 |  |  |
|  |  | SERPINA3N | |  |
|  |  | MROH2A | |  |
|  |  | MANEAL | |  |
|  |  | GUCD1 |  |  |
|  |  | HIST1H2BK | |  |
|  |  | FLNA |  |  |
|  |  | MAP3K5 |  |  |
|  |  | MYO1G |  |  |
|  |  | GM1123 |  |  |
|  |  | LHFPL3 |  |  |
|  |  | OPA3 |  |  |
|  |  | KCNA10 |  |  |
|  |  | BPIFB4 |  |  |
|  |  | SH2D1A |  |  |
|  |  | SGO2B |  |  |
|  |  | AREG |  |  |
|  |  | PIRA2 |  |  |
|  |  | GM11444 | |  |
|  |  | EMC9 |  |  |
|  |  | DQX1 |  |  |
|  |  | TNIK |  |  |
|  |  | GM572 |  |  |
|  |  | CDC42EP2 | |  |
|  |  | MAPK10 |  |  |
|  |  | AKR1C21 | |  |
|  |  | UBE2V2 |  |  |
|  |  | ANAPC7 |  |  |
|  |  | BC024063 | |  |
|  |  | SSTR4 |  |  |
|  |  | NEO1 |  |  |
|  |  | PHKG1 |  |  |
|  |  | LSM7 |  |  |
|  |  | ILDR2 |  |  |
|  |  | RUVBL1 |  |  |
|  |  | ZFP11 |  |  |
|  |  | 5430427O19RIK | |  |
|  |  | NFKBIB |  |  |
|  |  | DENND2D | |  |
|  |  | ZKSCAN6 | |  |
|  |  | SLC39A2 |  |  |
|  |  | VMN2R7 |  |  |
|  |  | IHH |  |  |
|  |  | DNAJC15 | |  |
|  |  | GM17304 | |  |
|  |  | IGSF11 |  |  |
|  |  | HACD4 |  |  |
|  |  | MIEF2 |  |  |
|  |  | SP6 |  |  |
|  |  | CALR |  |  |
|  |  | GM527 |  |  |
|  |  | TMEM154 | |  |
|  |  | HBQ1B |  |  |
|  |  | FBLN7 |  |  |
|  |  | SAMD1 |  |  |
|  |  | CHCHD3 |  |  |
|  |  | NAGK |  |  |
|  |  | NHLRC1 |  |  |
|  |  | YWHAZ |  |  |
|  |  | CYP20A1 | |  |
|  |  | ABHD14A | |  |
|  |  | GM17334 | |  |
|  |  | TULP4 |  |  |
|  |  | ACAT2 |  |  |
|  |  | DTX3 |  |  |
|  |  | GBP9 |  |  |
|  |  | LUM |  |  |
|  |  | ASCL4 |  |  |
|  |  | ADAM7 |  |  |
|  |  | TMEM39A | |  |
|  |  | MSRA |  |  |
|  |  | EEF1AKMT1 | |  |
|  |  | NAT8F4 |  |  |
|  |  | HMGCS2 |  |  |
|  |  | CNOT10 |  |  |
|  |  | TTC1 |  |  |
|  |  | IGF2BP2 |  |  |
|  |  | DIDO1 |  |  |
|  |  | RASSF5 |  |  |
|  |  | CTF2 |  |  |
|  |  | LACC1 |  |  |
|  |  | SDE2 |  |  |
|  |  | LURAP1L | |  |
|  |  | EIF2S1 |  |  |
|  |  | PPP1CB |  |  |
|  |  | CCDC163 | |  |
|  |  | ARHGAP45 | |  |
|  |  | TSN |  |  |
|  |  | KIF15 |  |  |
|  |  | 2900011O08RIK | |  |
|  |  | GLB1L |  |  |
|  |  | LY96 |  |  |
|  |  | SLC44A3 |  |  |
|  |  | NCAPD3 |  |  |
|  |  | SLC48A1 |  |  |
|  |  | 2310009B15RIK | |  |
|  |  | SLC39A7 |  |  |
|  |  | AMPD3 |  |  |
|  |  | B230219D22RIK | |  |
|  |  | SLC16A9 |  |  |
|  |  | AGPAT5 |  |  |
|  |  | GLG1 |  |  |
|  |  | GM5447 |  |  |
|  |  | 1700001C02RIK | |  |
|  |  | BC051019 | |  |
|  |  | CIZ1 |  |  |
|  |  | OTUD3 |  |  |
|  |  | ZFP697 |  |  |
|  |  | CAPZA1 |  |  |
|  |  | XKR6 |  |  |
|  |  | MCF2L |  |  |
|  |  | GM4952 |  |  |
|  |  | POLR2M |  |  |
|  |  | B230217C12RIK | |  |
|  |  | FCOR |  |  |
|  |  | MOB1A |  |  |
|  |  | U2SURP |  |  |
|  |  | OLFR878 |  |  |
|  |  | ZFP772 |  |  |
|  |  | JADE3 |  |  |
|  |  | ZMYND10 | |  |
|  |  | SSMEM1 |  |  |
|  |  | ATP13A1 | |  |
|  |  | RPS21 |  |  |
|  |  | PPP1R1A | |  |
|  |  | PDCD6IP | |  |
|  |  | SPATA2 |  |  |
|  |  | EGLN1 |  |  |
|  |  | RNF138 |  |  |
|  |  | ARHGEF37 | |  |
|  |  | ID1 |  |  |
|  |  | MAPK3 |  |  |
|  |  | GPBP1L1 | |  |
|  |  | RNF141 |  |  |
|  |  | PAN3 |  |  |
|  |  | PNKP |  |  |
|  |  | ZFP263 |  |  |
|  |  | SLC5A10 |  |  |
|  |  | RECK |  |  |
|  |  | AMOTL1 |  |  |
|  |  | SPNS3 |  |  |
|  |  | EMX1 |  |  |
|  |  | RUBCN |  |  |
|  |  | TSSK2 |  |  |
|  |  | UCP3 |  |  |
|  |  | SERPINB6C | |  |
|  |  | LZIC |  |  |
|  |  | EXOSC8 |  |  |
|  |  | FANCF |  |  |
|  |  | TROVE2 |  |  |
|  |  | MBNL2 |  |  |
|  |  | SCFD2 |  |  |
|  |  | FAM192A | |  |
|  |  | 6030408B16RIK | |  |
|  |  | MYH11 |  |  |
|  |  | PPIP5K2 |  |  |
|  |  | PDCD5 |  |  |
|  |  | ADAMTS15 | |  |
|  |  | NADSYN1 | |  |
|  |  | SERINC3 |  |  |
|  |  | AIM2 |  |  |
|  |  | GPR137 |  |  |
|  |  | ING3 |  |  |
|  |  | PLPP2 |  |  |
|  |  | 0610037L13RIK | |  |
|  |  | CSTF2 |  |  |
|  |  | CCDC25 |  |  |
|  |  | SMAD1 |  |  |
|  |  | SKIDA1 |  |  |
|  |  | AGTR1B |  |  |
|  |  | FAM186A | |  |
|  |  | TANC2 |  |  |
|  |  | CACNB1 |  |  |
|  |  | MYO1F |  |  |
|  |  | ANGEL2 |  |  |
|  |  | ELAC2 |  |  |
|  |  | FBXL4 |  |  |
|  |  | GNAT2 |  |  |
|  |  | MPG |  |  |
|  |  | KCTD10 |  |  |
|  |  | GOLT1B |  |  |
|  |  | SP5 |  |  |
|  |  | TFAM |  |  |
|  |  | LCMT2 |  |  |
|  |  | TMEM102 | |  |
|  |  | GM27029 | |  |
|  |  | RNF114 |  |  |
|  |  | TTC21B |  |  |
|  |  | IFT122 |  |  |
|  |  | NR2C1 |  |  |
|  |  | ADORA3 |  |  |
|  |  | TMEM150B | |  |
|  |  | CETN3 |  |  |
|  |  | TNN |  |  |
|  |  | ZFP933 |  |  |
|  |  | SLC9B1 |  |  |
|  |  | CDC23 |  |  |
|  |  | PPIF |  |  |
|  |  | TSTD1 |  |  |
|  |  | HIST2H4 |  |  |
|  |  | SH2D4B |  |  |
|  |  | CS |  |  |
|  |  | 1700029H14RIK | |  |
|  |  | COQ7 |  |  |
|  |  | MRFAP1 |  |  |
|  |  | OLFR250 |  |  |
|  |  | IQCF5 |  |  |
|  |  | GM10549 | |  |
|  |  | BEST2 |  |  |
|  |  | GM49394 | |  |
|  |  | SPATC1L | |  |
|  |  | SPATS1 |  |  |
|  |  | TMEM94 |  |  |
|  |  | CACNB2 |  |  |
|  |  | ABCA17 |  |  |
|  |  | ZCCHC9 |  |  |
|  |  | RASSF8 |  |  |
|  |  | ADAMTS13 | |  |
|  |  | NECAP1 |  |  |
|  |  | SLC26A10 | |  |
|  |  | IL17RC |  |  |
|  |  | MAD1L1 |  |  |
|  |  | HSPB6 |  |  |
|  |  | AKT2 |  |  |
|  |  | TMEM117 | |  |
|  |  | RER1 |  |  |
|  |  | OLFR110 |  |  |
|  |  | OLFR288 |  |  |
|  |  | CYP11B1 | |  |
|  |  | ABHD16B | |  |
|  |  | GM9736 |  |  |
|  |  | TNFSF15 |  |  |
|  |  | MUC16 |  |  |
|  |  | GDF6 |  |  |
|  |  | ZFP990 |  |  |
|  |  | CD300LB | |  |
|  |  | RIMS1 |  |  |
|  |  | BTNL9 |  |  |
|  |  | ROCK1 |  |  |
|  |  | TCEA3 |  |  |
|  |  | WDR37 |  |  |
|  |  | ATG4D |  |  |
|  |  | CDK20 |  |  |
|  |  | TSPAN3 |  |  |
|  |  | 9130019O22RIK | |  |
|  |  | CENPI |  |  |
|  |  | MGAT1 |  |  |
|  |  | B130006D01RIK | |  |
|  |  | MTIF2 |  |  |
|  |  | ZFP942 |  |  |
|  |  | GPT |  |  |
|  |  | RNF38 |  |  |
|  |  | DCTN1 |  |  |
|  |  | HSPB2 |  |  |
|  |  | GBX2 |  |  |
|  |  | TMEM161B | |  |
|  |  | INO80B |  |  |
|  |  | BCORL1 |  |  |
|  |  | CDK2AP1 | |  |
|  |  | NUDT18 |  |  |
|  |  | ARID3C |  |  |
|  |  | NSF |  |  |
|  |  | TSC22D2 | |  |
|  |  | PKD1L3 |  |  |
|  |  | AOX4 |  |  |
|  |  | FAXC |  |  |
|  |  | RICTOR |  |  |
|  |  | SORBS2 |  |  |
|  |  | OTULINL | |  |
|  |  | IL17RA |  |  |
|  |  | BC055324 | |  |
|  |  | RUSC1 |  |  |
|  |  | YAF2 |  |  |
|  |  | BBS5 |  |  |
|  |  | TMEM97 |  |  |
|  |  | RAC1 |  |  |
|  |  | TIPARP |  |  |
|  |  | B3GNT8 |  |  |
|  |  | VSTM5 |  |  |
|  |  | CXXC1 |  |  |
|  |  | SMIM24 |  |  |
|  |  | AMZ2 |  |  |
|  |  | ATF1 |  |  |
|  |  | KRBA1 |  |  |
|  |  | NUDT11 |  |  |
|  |  | IDUA |  |  |
|  |  | SHC3 |  |  |
|  |  | SAT1 |  |  |
|  |  | B4GALNT3 | |  |
|  |  | HAGHL |  |  |
|  |  | TRIP11 |  |  |
|  |  | EMC4 |  |  |
|  |  | GM29609 | |  |
|  |  | ARHGDIG | |  |
|  |  | DNASE1L1 | |  |
|  |  | ECT2L |  |  |
|  |  | A26C2 |  |  |
|  |  | BPIFB6 |  |  |
|  |  | GCHFR |  |  |
|  |  | OLFR1511 | |  |
|  |  | PRSS38 |  |  |
|  |  | GM45194 | |  |
|  |  | FOXE1 |  |  |
|  |  | GP1BB |  |  |
|  |  | OLFR1140 | |  |
|  |  | OLFR1336 | |  |
|  |  | GIMAP3 |  |  |
|  |  | GM281 |  |  |
|  |  | AYM1 |  |  |
|  |  | BC147527 | |  |
|  |  | PDXDC1 |  |  |
|  |  | ENTPD5 |  |  |
|  |  | PGAM2 |  |  |
|  |  | NIN |  |  |
|  |  | CYTH2 |  |  |
|  |  | GTF3C3 |  |  |
|  |  | EXOC2 |  |  |
|  |  | STEAP3 |  |  |
|  |  | ADGRB2 |  |  |
|  |  | ARID3A |  |  |
|  |  | CNDP1 |  |  |
|  |  | LRRC32 |  |  |
|  |  | CUL1 |  |  |
|  |  | CCDC59 |  |  |
|  |  | NOL3 |  |  |
|  |  | SMIM26 |  |  |
|  |  | RGS7 |  |  |
|  |  | CD72 |  |  |
|  |  | TIMM10 |  |  |
|  |  | MEAF6 |  |  |
|  |  | CTDP1 |  |  |
|  |  | ENO4 |  |  |
|  |  | ZFP934 |  |  |
|  |  | TDRD7 |  |  |
|  |  | NFAM1 |  |  |
|  |  | ZFP644 |  |  |
|  |  | MTAP |  |  |
|  |  | MTX1 |  |  |
|  |  | MEIOC |  |  |
|  |  | OTX1 |  |  |
|  |  | MINK1 |  |  |
|  |  | DDX55 |  |  |
|  |  | DDX39B |  |  |
|  |  | SHISA9 |  |  |
|  |  | EDRF1 |  |  |
|  |  | ZBTB21 |  |  |
|  |  | THPO |  |  |
|  |  | GPATCH11 | |  |
|  |  | D11WSU47E | |  |
|  |  | BPNT1 |  |  |
|  |  | TRHR2 |  |  |
|  |  | LDLR |  |  |
|  |  | BMPER |  |  |
|  |  | SLC43A3 |  |  |
|  |  | OLFML2A | |  |
|  |  | TMEM120A | |  |
|  |  | GMIP |  |  |
|  |  | HARS2 |  |  |
|  |  | P3H1 |  |  |
|  |  | RCC1L |  |  |
|  |  | PHLPP2 |  |  |
|  |  | 1500015O10RIK | |  |
|  |  | EMX2 |  |  |
|  |  | RPP30 |  |  |
|  |  | TXNL4A |  |  |
|  |  | HK1 |  |  |
|  |  | ZGRF1 |  |  |
|  |  | IMMT |  |  |
|  |  | ECI1 |  |  |
|  |  | CSNKA2IP | |  |
|  |  | MYBL1 |  |  |
|  |  | RALYL |  |  |
|  |  | LIG3 |  |  |
|  |  | CRADD |  |  |
|  |  | ODF3 |  |  |
|  |  | MARK1 |  |  |
|  |  | HVCN1 |  |  |
|  |  | BPIFA1 |  |  |
|  |  | RWDD4A | |  |
|  |  | TMEM56 |  |  |
|  |  | TGM7 |  |  |
|  |  | 1110032A03RIK | |  |
|  |  | CHD8 |  |  |
|  |  | GM42791 | |  |
|  |  | MLXIP |  |  |
|  |  | KMT2E |  |  |
|  |  | SCGB3A1 | |  |
|  |  | ALLC |  |  |
|  |  | MED6 |  |  |
|  |  | CMC1 |  |  |
|  |  | TMEM9B | |  |
|  |  | OLFR524 |  |  |
|  |  | EGR3 |  |  |
|  |  | GM3047 |  |  |
|  |  | OIT1 |  |  |
|  |  | THEM5 |  |  |
|  |  | PCMT1 |  |  |
|  |  | GSTM3 |  |  |
|  |  | OLFR118 |  |  |
|  |  | GULO |  |  |
|  |  | OLFR1016 | |  |
|  |  | GM17079 | |  |
|  |  | GM17067 | |  |
|  |  | CEBPE |  |  |
|  |  | KRTAP3-3 | |  |
|  |  | GM30083 | |  |
|  |  | SYT8 |  |  |
|  |  | MRGPRA2B | |  |
|  |  | MRGPRA2A | |  |
|  |  | FLG |  |  |
|  |  | 9230104L09RIK | |  |
|  |  | IQCF3 |  |  |
|  |  | KLK14 |  |  |
|  |  | GP2 |  |  |
|  |  | MIOX |  |  |
|  |  | LCN11 |  |  |
|  |  | SAPCD1 |  |  |
|  |  | MAGEA10 | |  |
|  |  | PRICKLE4 | |  |
|  |  | GM11938 | |  |
|  |  | OLFR875 |  |  |
|  |  | GM7298 |  |  |
|  |  | GM47969 | |  |
|  |  | NPPB |  |  |
|  |  | 5830411N06RIK | |  |
|  |  | GM10318 | |  |
|  |  | OLFR970 |  |  |
|  |  | OLFR995 |  |  |
|  |  | OLFR998 |  |  |
|  |  | GM7168 |  |  |
|  |  | GM10330 | |  |
|  |  | PRODH2 |  |  |
|  |  | BOLL |  |  |
|  |  | DNAJB8 |  |  |
|  |  | OLFR1173 | |  |
|  |  | VMN1R25 | |  |
|  |  | VMN1R16 | |  |
|  |  | OLFR344 |  |  |
|  |  | AMBN |  |  |
|  |  | NMS |  |  |
|  |  | BTBD35F25 | |  |
|  |  | NPS |  |  |
|  |  | OLFR1493-PS1 | |  |
|  |  | OLFR361 |  |  |
|  |  | VMN1R87 | |  |
|  |  | VMN1R80 | |  |
|  |  | VMN1R81 | |  |
|  |  | GM9573 |  |  |
|  |  | OLFR1276 | |  |
|  |  | GM8212 |  |  |
|  |  | OLFR1284 | |  |
|  |  | CACNG7 |  |  |
|  |  | OLFR430 |  |  |
|  |  | OLFR434 |  |  |
|  |  | GM20425 | |  |
|  |  | FOXL1 |  |  |
|  |  | ASB12 |  |  |
|  |  | OLFR1156 | |  |
|  |  | OLFR532 |  |  |
|  |  | OLFR531 |  |  |
|  |  | ATG2A |  |  |
|  |  | GM10715 | |  |
|  |  | OLFR576 |  |  |
|  |  | OLFR572 |  |  |
|  |  | SPESP1 |  |  |
|  |  | MPTX1 |  |  |
|  |  | PRN |  |  |
|  |  | OLFR1450 | |  |
|  |  | IFNE |  |  |
|  |  | OLFR695 |  |  |
|  |  | ASCL3 |  |  |
|  |  | TRPC2 |  |  |
|  |  | APON |  |  |
|  |  | IFNA2 |  |  |
|  |  | OLFR1346 | |  |
|  |  | OLFR1371 | |  |
|  |  | TUBAL3 |  |  |
|  |  | CLDN34C3 | |  |
|  |  | GM11077 | |  |
|  |  | RBP7 |  |  |
|  |  | LILRA6 |  |  |
|  |  | MUP7 |  |  |
|  |  | BC048671 | |  |
|  |  | UROC1 |  |  |
|  |  | H2AL1A |  |  |
|  |  | TAS2R119 | |  |
|  |  | TAS2R125 | |  |
|  |  | TMPRSS13 | |  |
|  |  | ASGR2 |  |  |
|  |  | ADGRG4 |  |  |
|  |  | NRXN2 |  |  |
|  |  | CRYGA |  |  |
|  |  | RFX6 |  |  |
|  |  | ZFP936 |  |  |
|  |  | ZFP987 |  |  |
|  |  | GGCX |  |  |
|  |  | GTF2A1L | |  |
|  |  | KRTAP10-10 | |  |
|  |  | SULT2A7 | |  |
|  |  | VMN1R196 | |  |
|  |  | CYPT1 |  |  |
|  |  | TNP2 |  |  |
|  |  | XLR5B |  |  |
|  |  | TMEM233 | |  |
|  |  | DPPA4 |  |  |
|  |  | GM1330 |  |  |
|  |  | S100A2 |  |  |
|  |  | GM10024 | |  |
|  |  | CD207 |  |  |
|  |  | PDILT |  |  |
|  |  | CYP3A16 | |  |
|  |  | AA792892 | |  |
|  |  | MAT2A |  |  |
|  |  | DYNAP |  |  |
|  |  | PIH1D3 |  |  |
|  |  | NAT3 |  |  |
|  |  | FBXW25 |  |  |
|  |  | WFDC6A |  |  |
|  |  | CYP2B23 | |  |
|  |  | ALKAL1 |  |  |
|  |  | CMTM2B | |  |
|  |  | TRIML2 |  |  |
|  |  | SPINT5 |  |  |
|  |  | FNDC3C1 | |  |
|  |  | RNASEL |  |  |
|  |  | POFUT2 |  |  |
|  |  | CUZD1 |  |  |
|  |  | COL4A4 |  |  |
|  |  | TMEM169 | |  |
|  |  | GM4353 |  |  |
|  |  | USP5 |  |  |
|  |  | SCAMP3 |  |  |
|  |  | TEX35 |  |  |
|  |  | PIH1D2 |  |  |
|  |  | GOLGB1 |  |  |
|  |  | SUGP2 |  |  |
|  |  | SERPINE2 | |  |
|  |  | EVC |  |  |
|  |  | UBE2S |  |  |
|  |  | FZD8 |  |  |
|  |  | 1700001K19RIK | |  |
|  |  | UBE2QL1 | |  |
|  |  | GNA11 |  |  |
|  |  | GIGYF2 |  |  |
|  |  | PBLD2 |  |  |
|  |  | PADI6 |  |  |
|  |  | ARL14EP | |  |
|  |  | NAIP2 |  |  |
|  |  | 2210011C24RIK | |  |
|  |  | SPATA25 | |  |
|  |  | ENDOG |  |  |
|  |  | HIST1H2AF | |  |
|  |  | BRMS1L |  |  |
|  |  | PLCXD1 |  |  |
|  |  | ACSM3 |  |  |
|  |  | CFI |  |  |
|  |  | GUCA2A |  |  |
|  |  | GM3239 |  |  |
|  |  | IL1RL2 |  |  |
|  |  | ZFP148 |  |  |
|  |  | FAM20A |  |  |
|  |  | RAVER1 |  |  |
|  |  | BC048679 | |  |
|  |  | EOMES |  |  |
|  |  | QPRT |  |  |
|  |  | FLI1 |  |  |
|  |  | PEAR1 |  |  |
|  |  | ACOT12 |  |  |
|  |  | FUT10 |  |  |
|  |  | PMM2 |  |  |
|  |  | AGBL5 |  |  |
|  |  | LGALS12 | |  |
|  |  | MED21 |  |  |
|  |  | LENG9 |  |  |
|  |  | AMD2 |  |  |
|  |  | PECAM1 |  |  |
|  |  | SUCO |  |  |
|  |  | LSM3 |  |  |
|  |  | WFDC1 |  |  |
|  |  | SLCO6C1 | |  |
|  |  | GPR19 |  |  |
|  |  | PCDHGC4 | |  |
|  |  | PCDHB5 |  |  |
|  |  | RNF41 |  |  |
|  |  | MERTK |  |  |
|  |  | ZCCHC4 |  |  |
|  |  | ARNT2 |  |  |
|  |  | LENG1 |  |  |
|  |  | FGF18 |  |  |
|  |  | ITGB3 |  |  |
|  |  | TEX52 |  |  |
|  |  | POFUT1 |  |  |
|  |  | DNAL1 |  |  |
|  |  | NOLC1 |  |  |
|  |  | SLC4A2 |  |  |
|  |  | ZBED3 |  |  |
|  |  | L1TD1 |  |  |
|  |  | GATA6 |  |  |
|  |  | TLE4 |  |  |
|  |  | NDUFC2 |  |  |
|  |  | IPP |  |  |
|  |  | BCOR |  |  |
|  |  | ITM2B |  |  |
|  |  | CARD10 |  |  |
|  |  | 4930524B15RIK | |  |
|  |  | 2610021A01RIK | |  |
|  |  | DIS3L |  |  |
|  |  | L3MBTL1 | |  |
|  |  | RHBDD1 |  |  |
|  |  | TRIM44 |  |  |
|  |  | DHFR |  |  |
|  |  | MAPK4 |  |  |
|  |  | TRIM36 |  |  |
|  |  | GNG12 |  |  |
|  |  | NOX3 |  |  |
|  |  | ZFAND2B | |  |
|  |  | WDR91 |  |  |
|  |  | OGFOD2 |  |  |
|  |  | SCN2A |  |  |
|  |  | XLR3A |  |  |
|  |  | THAP2 |  |  |
|  |  | BCL2L1 |  |  |
|  |  | USP44 |  |  |
|  |  | PCDHGA1 | |  |
|  |  | DBNDD2 |  |  |
|  |  | NSD1 |  |  |
|  |  | TOP3A |  |  |
|  |  | CHD4 |  |  |
|  |  | APH1B |  |  |
|  |  | NBL1 |  |  |
|  |  | KRTCAP2 | |  |
|  |  | FSD1L |  |  |
|  |  | ATP11B |  |  |
|  |  | GM9803 |  |  |
|  |  | MANEA |  |  |
|  |  | SERP1 |  |  |
|  |  | SHROOM4 | |  |
|  |  | SHROOM3 | |  |
|  |  | RSRC1 |  |  |
|  |  | CLNK |  |  |
|  |  | ZFP689 |  |  |
|  |  | CYP2J7 |  |  |
|  |  | NTRK2 |  |  |
|  |  | PEX16 |  |  |
|  |  | IQCG |  |  |
|  |  | WDR63 |  |  |
|  |  | FAM24B |  |  |
|  |  | TCF15 |  |  |
|  |  | NOP2 |  |  |
|  |  | ANKRD23 | |  |
|  |  | EYA2 |  |  |
|  |  | SAV1 |  |  |
|  |  | CEL |  |  |
|  |  | ZFP398 |  |  |
|  |  | LOXHD1 |  |  |
|  |  | LFNG |  |  |
|  |  | NAP1L5 |  |  |
|  |  | RYK |  |  |
|  |  | ATP6V1F | |  |
|  |  | DNMT3A | |  |
|  |  | TADA2A |  |  |
|  |  | EPHX1 |  |  |
|  |  | ANKRD34A | |  |
|  |  | PCGF3 |  |  |
|  |  | TMEM147 | |  |
|  |  | ARHGAP23 | |  |
|  |  | GRM1 |  |  |
|  |  | CARD9 |  |  |
|  |  | CLSTN3 |  |  |
|  |  | GM19345 | |  |
|  |  | NICN1 |  |  |
|  |  | C1RL |  |  |
|  |  | FAM83H |  |  |
|  |  | FAH |  |  |
|  |  | DGAT2L6 | |  |
|  |  | CK137956 | |  |
|  |  | 2700097O09RIK | |  |
|  |  | RHPN1 |  |  |
|  |  | RASSF4 |  |  |
|  |  | FN3KRP |  |  |
|  |  | GM340 |  |  |
|  |  | SLC22A18 | |  |
|  |  | PROCR |  |  |
|  |  | RPS20 |  |  |
|  |  | LRP3 |  |  |
|  |  | CYSTM1 |  |  |
|  |  | EHMT2 |  |  |
|  |  | ZFP617 |  |  |
|  |  | ARHGEF17 | |  |
|  |  | VARS2 |  |  |
|  |  | AJAP1 |  |  |
|  |  | CPED1 |  |  |
|  |  | HS3ST5 |  |  |
|  |  | CARM1 |  |  |
|  |  | HIST1H2AB | |  |
|  |  | VWA8 |  |  |
|  |  | ENPP2 |  |  |
|  |  | PRRT1 |  |  |
|  |  | BET1 |  |  |
|  |  | ZBTB25 |  |  |
|  |  | NAA25 |  |  |
|  |  | ATG4B |  |  |
|  |  | TFAP2B |  |  |
|  |  | FAM114A1 | |  |
|  |  | USP19 |  |  |
|  |  | SYNE2 |  |  |
|  |  | GM18336 | |  |
|  |  | TIMD4 |  |  |
|  |  | GM6377 |  |  |
|  |  | GM7138 |  |  |
|  |  | ART5 |  |  |
|  |  | DNASE2B | |  |
|  |  | PRSS1 |  |  |
|  |  | CYP19A1 | |  |
|  |  | TRIM56 |  |  |
|  |  | TCAF2 |  |  |
|  |  | KCNH4 |  |  |
|  |  | YIPF3 |  |  |
|  |  | MT-ND1 |  |  |
|  |  | TTC39A |  |  |
|  |  | CDHR3 |  |  |
|  |  | RTTN |  |  |
|  |  | TSPAN8 |  |  |
|  |  | CMPK2 |  |  |
|  |  | CEBPZ |  |  |
|  |  | TAF7 |  |  |
|  |  | TMX3 |  |  |
|  |  | LY6D |  |  |
|  |  | ZBTB44 |  |  |
|  |  | FAM216B | |  |
|  |  | CNGB1 |  |  |
|  |  | NCBP1 |  |  |
|  |  | FAM207A | |  |
|  |  | ARF3 |  |  |
|  |  | ACAP3 |  |  |
|  |  | PPP2CA |  |  |
|  |  | SAG |  |  |
|  |  | PHF20L1 |  |  |
|  |  | SRSF4 |  |  |
|  |  | CCDC136 | |  |
|  |  | UNC50 |  |  |
|  |  | NHLRC3 |  |  |
|  |  | PRSS27 |  |  |
|  |  | ZFP780B |  |  |
|  |  | FAM174B | |  |
|  |  | MCM3 |  |  |
|  |  | IFITM7 |  |  |
|  |  | MRPS25 |  |  |
|  |  | ACTL6B |  |  |
|  |  | VPS13B |  |  |
|  |  | NDUFV2 |  |  |
|  |  | FGD3 |  |  |
|  |  | COG3 |  |  |
|  |  | IGDCC3 |  |  |
|  |  | ZFP369 |  |  |
|  |  | RNFT1 |  |  |
|  |  | MSC |  |  |
|  |  | KLK9 |  |  |
|  |  | PKIG |  |  |
|  |  | NOL10 |  |  |
|  |  | VCAN |  |  |
|  |  | BCHE |  |  |
|  |  | APOC3 |  |  |
|  |  | GM5689 |  |  |
|  |  | OLFR237-PS1 | |  |
|  |  | CTLA4 |  |  |
|  |  | 1700012B07RIK | |  |
|  |  | ABCB11 |  |  |
|  |  | SPAM1 |  |  |
|  |  | CLEC4F |  |  |
|  |  | CLEC4E |  |  |
|  |  | CFD |  |  |
|  |  | CGA |  |  |
|  |  | SPACA4 |  |  |
|  |  | GM5426 |  |  |
|  |  | CRP |  |  |
|  |  | OLFR165 |  |  |
|  |  | GM7970 |  |  |
|  |  | RDH19 |  |  |
|  |  | C130032M10RIK | |  |
|  |  | GM21983 | |  |
|  |  | FAM25C |  |  |
|  |  | SPATA31D1C | |  |
|  |  | SERPINA3B | |  |
|  |  | RAB3A |  |  |
|  |  | KAZN |  |  |
|  |  | GM17604 | |  |
|  |  | GM15097 | |  |
|  |  | ATP5A1 |  |  |
|  |  | PRM3 |  |  |
|  |  | FUT1 |  |  |
|  |  | ELOBL |  |  |
|  |  | GM28051 | |  |
|  |  | ESX1 |  |  |
|  |  | HPD |  |  |
|  |  | GZMA |  |  |
|  |  | OLFR775 |  |  |
|  |  | PRAME |  |  |
|  |  | VMN2R102 | |  |
|  |  | PSG19 |  |  |
|  |  | PSG28 |  |  |
|  |  | MS4A3 |  |  |
|  |  | GM6034 |  |  |
|  |  | LIM2 |  |  |
|  |  | MS4A1 |  |  |
|  |  | OLFR889 |  |  |
|  |  | OLFR808 |  |  |
|  |  | OLFR862 |  |  |
|  |  | OLFR1505 | |  |
|  |  | OLFR1513 | |  |
|  |  | GM47985 | |  |
|  |  | CXCL13 |  |  |
|  |  | 1700025F22RIK | |  |
|  |  | ACSBG2 |  |  |
|  |  | VMN2R97 | |  |
|  |  | VMN1R37 | |  |
|  |  | ZDHHC8 |  |  |
|  |  | VMN1R15 | |  |
|  |  | OLFR322 |  |  |
|  |  | OLFR324 |  |  |
|  |  | CALHM3 |  |  |
|  |  | VMN1R88 | |  |
|  |  | OLFR1250 | |  |
|  |  | TNFSF14 |  |  |
|  |  | GM20403 | |  |
|  |  | FOXB2 |  |  |
|  |  | GM21761 | |  |
|  |  | KLRA7 |  |  |
|  |  | ZC2HC1B | |  |
|  |  | HORMAD1 | |  |
|  |  | OLFR1132 | |  |
|  |  | OLFR591 |  |  |
|  |  | OLFR512 |  |  |
|  |  | OLFR1497 | |  |
|  |  | UTS2B |  |  |
|  |  | OLFR655 |  |  |
|  |  | OLFR699 |  |  |
|  |  | OLFR1369-PS1 | |  |
|  |  | DHRS9 |  |  |
|  |  | CLCA1 |  |  |
|  |  | OLFR1378 | |  |
|  |  | CCL19 |  |  |
|  |  | NADK2 |  |  |
|  |  | EFCAB3 |  |  |
|  |  | GM11020 | |  |
|  |  | C130073F10RIK | |  |
|  |  | CES1F |  |  |
|  |  | ZSWIM2 |  |  |
|  |  | SLFN8 |  |  |
|  |  | GM36176 | |  |
|  |  | MMP12 |  |  |
|  |  | WFDC15A | |  |
|  |  | REG1 |  |  |
|  |  | A1BG |  |  |
|  |  | H2-Q10 |  |  |
|  |  | MYOT |  |  |
|  |  | ZFP980 |  |  |
|  |  | ZFP967 |  |  |
|  |  | GM1587 |  |  |
|  |  | OLFR71 |  |  |
|  |  | INS2 |  |  |
|  |  | RTL8A |  |  |
|  |  | DEFB25 |  |  |
|  |  | VMN1R228 | |  |
|  |  | GJB4 |  |  |
|  |  | LECT2 |  |  |
|  |  | CALM4 |  |  |
|  |  | NLRP4C |  |  |
|  |  | PPM1N |  |  |
|  |  | H1FOO |  |  |
|  |  | GPIHBP1 | |  |
|  |  | ODF1 |  |  |
|  |  | PGM2 |  |  |
|  |  | CYP4A10 | |  |
|  |  | GM3486 |  |  |
|  |  | HRH4 |  |  |
|  |  | GM14548 | |  |
|  |  | TRIM58 |  |  |
|  |  | BTNL1 |  |  |
|  |  | OBOX7 |  |  |
|  |  | GALNTL5 | |  |
|  |  | RPTN |  |  |
|  |  | CD8B1 |  |  |
|  |  | 4930558K02RIK | |  |
|  |  | SH2D4A |  |  |
|  |  | CAMK2N2 | |  |
|  |  | ATG4C |  |  |
|  |  | C330018D20RIK | |  |
|  |  | SLC29A1 |  |  |
|  |  | ALPK3 |  |  |
|  |  | TSR1 |  |  |
|  |  | APOBR |  |  |
|  |  | VMN2R6 |  |  |
|  |  | GM5039 |  |  |
|  |  | GM5096 |  |  |
|  |  | GZMB |  |  |
|  |  | HAMP |  |  |
|  |  | OLFR653 |  |  |
|  |  | PADI1 |  |  |
|  |  | GM11361 | |  |
|  |  | AASDH |  |  |
|  |  | JUNB |  |  |
|  |  | SNX32 |  |  |
|  |  | FNDC5 |  |  |
|  |  | RPS6KA1 | |  |
|  |  | CYP2D22 | |  |
|  |  | GTF2I |  |  |
|  |  | LRG1 |  |  |
|  |  | ROBO2 |  |  |
|  |  | RAP1B |  |  |
|  |  | TBC1D14 | |  |
|  |  | POLR2K |  |  |
|  |  | ETS1 |  |  |
|  |  | TM9SF4 |  |  |
|  |  | HCFC2 |  |  |
|  |  | RPP14 |  |  |
|  |  | HIST1H3E | |  |
|  |  | HGSNAT |  |  |
|  |  | PLEKHO1 | |  |
|  |  | BMX |  |  |
|  |  | SNX14 |  |  |
|  |  | UBXN11 |  |  |
|  |  | CDK7 |  |  |
|  |  | PITHD1 |  |  |
|  |  | USP13 |  |  |
|  |  | VASN |  |  |
|  |  | PRCD |  |  |
|  |  | CHCHD4 |  |  |
|  |  | TIMELESS | |  |
|  |  | PHOSPHO1 | |  |
|  |  | CHD2 |  |  |
|  |  | ACOT11 |  |  |
|  |  | ZXDB |  |  |
|  |  | DUSP1 |  |  |
|  |  | RHOG |  |  |
|  |  | SDCBP2 |  |  |
|  |  | AMDHD1 | |  |
|  |  | HSF2 |  |  |
|  |  | MAP3K8 |  |  |
|  |  | PDK3 |  |  |
|  |  | TCAM1 |  |  |
|  |  | SLC38A2 |  |  |
|  |  | FLT3 |  |  |
|  |  | FAM205A1 | |  |
|  |  | LLGL1 |  |  |
|  |  | DNAH7C |  |  |
|  |  | MEPCE |  |  |
|  |  | SNCA |  |  |
|  |  | TFR2 |  |  |
|  |  | SLC35F4 |  |  |
|  |  | COLCA2 |  |  |
|  |  | CENPV |  |  |
|  |  | HIPK3 |  |  |
|  |  | BCL6B |  |  |
|  |  | CTNND1 |  |  |
|  |  | NACA |  |  |
|  |  | MSH6 |  |  |
|  |  | MPHOSPH8 | |  |
|  |  | RUFY2 |  |  |
|  |  | CAPN5 |  |  |
|  |  | CHRNA2 |  |  |
|  |  | ADAM23 |  |  |
|  |  | FAM167B | |  |
|  |  | SLC6A5 |  |  |
|  |  | METAP1 |  |  |
|  |  | RNF222 |  |  |
|  |  | TMCO5B | |  |
|  |  | CLDN8 |  |  |
|  |  | GTSF2 |  |  |
|  |  | GM43517 | |  |
|  |  | LEMD2 |  |  |
|  |  | RNF5 |  |  |
|  |  | LGALS1 |  |  |
|  |  | MNT |  |  |
|  |  | NDUFAF7 | |  |
|  |  | TRAPPC5 | |  |
|  |  | NUP107 |  |  |
|  |  | RIT1 |  |  |
|  |  | CRBN |  |  |
|  |  | CMAH |  |  |
|  |  | WDR25 |  |  |
|  |  | ZFP629 |  |  |
|  |  | TES3-PS |  |  |
|  |  | CCER2 |  |  |
|  |  | PKN2 |  |  |
|  |  | PFN4 |  |  |
|  |  | ZFP382 |  |  |
|  |  | CBX4 |  |  |
|  |  | EPHA4 |  |  |
|  |  | NUDT16L1 | |  |
|  |  | TMEM106C | |  |
|  |  | NEFM |  |  |
|  |  | SPATA2L | |  |
|  |  | AFG3L2 |  |  |
|  |  | PSMB9 |  |  |
|  |  | PTRHD1 |  |  |
|  |  | HYAL3 |  |  |
|  |  | IZUMO1 |  |  |
|  |  | HS3ST6 |  |  |
|  |  | IRX5 |  |  |
|  |  | SAMSN1 |  |  |
|  |  | 1190002N15RIK | |  |
|  |  | RALGAPA1 | |  |
|  |  | RAB27B |  |  |
|  |  | SPAG7 |  |  |
|  |  | NFKBID |  |  |
|  |  | GM5580 |  |  |
|  |  | FIZ1 |  |  |
|  |  | CBX2 |  |  |
|  |  | CLIP4 |  |  |
|  |  | HMGCR |  |  |
|  |  | DNAJC19 | |  |
|  |  | ZFP65 |  |  |
|  |  | LRP4 |  |  |
|  |  | ABLIM1 |  |  |
|  |  | PROK2 |  |  |
|  |  | CGGBP1 |  |  |
|  |  | DDX43 |  |  |
|  |  | PLK3 |  |  |
|  |  | AMMECR1 | |  |
|  |  | PDIA5 |  |  |
|  |  | HSPH1 |  |  |
|  |  | METTL5 |  |  |
|  |  | ABCD3 |  |  |
|  |  | NAA30 |  |  |
|  |  | MTMR14 | |  |
|  |  | 4933405O20RIK | |  |
|  |  | GM14325 | |  |
|  |  | ENPP4 |  |  |
|  |  | SELENOM | |  |
|  |  | HIP1R |  |  |
|  |  | ZFP954 |  |  |
|  |  | NXF3 |  |  |
|  |  | GBE1 |  |  |
|  |  | RAD18 |  |  |
|  |  | KCNF1 |  |  |
|  |  | NAT8 |  |  |
|  |  | ISOC2A |  |  |
|  |  | ADCYAP1 | |  |
|  |  | NDUFB6 |  |  |
|  |  | PITPNB |  |  |
|  |  | CDC42SE1 | |  |
|  |  | GPR55 |  |  |
|  |  | SLC35B4 |  |  |
|  |  | REEP3 |  |  |
|  |  | DDR1 |  |  |
|  |  | SLC51A |  |  |
|  |  | ALG10B |  |  |
|  |  | MSH4 |  |  |
|  |  | ALG2 |  |  |
|  |  | OLFR1428 | |  |
|  |  | CIT |  |  |
|  |  | BANF1 |  |  |
|  |  | FMNL3 |  |  |
|  |  | SERPINA10 | |  |
|  |  | UBE3B |  |  |
|  |  | VIRMA |  |  |
|  |  | RCN2 |  |  |
|  |  | HSP90B1 |  |  |
|  |  | PCDHB3 |  |  |
|  |  | PLEKHM1 | |  |
|  |  | ZFP663 |  |  |
|  |  | ANGPTL7 | |  |
|  |  | JMJD1C |  |  |
|  |  | STBD1 |  |  |
|  |  | GNB1L |  |  |
|  |  | CHST2 |  |  |
|  |  | STRC |  |  |
|  |  | FAM76B |  |  |
|  |  | SMG5 |  |  |
|  |  | BIRC7 |  |  |
|  |  | ATP5E |  |  |
|  |  | VTA1 |  |  |
|  |  | CD244A |  |  |
|  |  | NAMPT |  |  |
|  |  | URGCP |  |  |
|  |  | GM9955 |  |  |
|  |  | MUT |  |  |
|  |  | PPIH |  |  |
|  |  | 2-Mar |  |  |
|  |  | RBM39 |  |  |
|  |  | GTF3C6 |  |  |
|  |  | PATL1 |  |  |
|  |  | 1810013L24RIK | |  |
|  |  | RTF1 |  |  |
|  |  | GSTM1 |  |  |
|  |  | MTFR2 |  |  |
|  |  | ZFP395 |  |  |
|  |  | FBXL13 |  |  |
|  |  | SEPSECS | |  |
|  |  | NUDT9 |  |  |
|  |  | RGL1 |  |  |
|  |  | MAP2K1 |  |  |
|  |  | GAP43 |  |  |
|  |  | PLEKHM2 | |  |
|  |  | CATSPERZ | |  |
|  |  | FAM69A |  |  |
|  |  | TSTA3 |  |  |
|  |  | UBE2N |  |  |
|  |  | LHX5 |  |  |
|  |  | CD177 |  |  |
|  |  | OLFR10 |  |  |
|  |  | POU4F3 |  |  |
|  |  | TCTA |  |  |
|  |  | GPR85 |  |  |
|  |  | SLC35G2 |  |  |
|  |  | HEBP2 |  |  |
|  |  | DDX23 |  |  |
|  |  | MAD2L1 |  |  |
|  |  | BCO2 |  |  |
|  |  | SENP3 |  |  |
|  |  | SPIRE2 |  |  |
|  |  | NAA60 |  |  |
|  |  | ST3GAL3 | |  |
|  |  | NKAPD1 |  |  |
|  |  | DDX31 |  |  |
|  |  | ZEB1 |  |  |
|  |  | MKL2 |  |  |
|  |  | BTBD6 |  |  |
|  |  | KRT23 |  |  |
|  |  | CDYL2 |  |  |
|  |  | USP47 |  |  |
|  |  | GPS1 |  |  |
|  |  | PCSK9 |  |  |
|  |  | PIWIL1 |  |  |
|  |  | MARF1 |  |  |
|  |  | ZFP322A |  |  |
|  |  | VPS4A |  |  |
|  |  | IFIT1BL1 | |  |
|  |  | LRP2BP |  |  |
|  |  | TNFAIP8L1 | |  |
|  |  | MRPL16 |  |  |
|  |  | AP1B1 |  |  |
|  |  | STK38L |  |  |
|  |  | RRM2B |  |  |
|  |  | VWA5B2 |  |  |
|  |  | CAR5A |  |  |
|  |  | CCAR1 |  |  |
|  |  | FOXRED1 | |  |
|  |  | PLK5 |  |  |
|  |  | IRF9 |  |  |
|  |  | SRSF9 |  |  |
|  |  | ERN2 |  |  |
|  |  | MASP1 |  |  |
|  |  | NQO1 |  |  |
|  |  | LCP2 |  |  |
|  |  | MED8 |  |  |
|  |  | CCDC6 |  |  |
|  |  | KANK1 |  |  |
|  |  | ZFAND4 |  |  |
|  |  | SH2D7 |  |  |
|  |  | USP16 |  |  |
|  |  | KRT40 |  |  |
|  |  | CAPN8 |  |  |
|  |  | ADPRHL1 | |  |
|  |  | PIRT |  |  |
|  |  | AATK |  |  |
|  |  | GM10767 | |  |
|  |  | SLC25A19 | |  |
|  |  | SP7 |  |  |
|  |  | CPE |  |  |
|  |  | MYD88 |  |  |
|  |  | STAG2 |  |  |
|  |  | NXPE3 |  |  |
|  |  | RPS19BP1 | |  |
|  |  | KLHDC2 |  |  |
|  |  | NUP133 |  |  |
|  |  | SCAMP5 |  |  |
|  |  | MPEG1 |  |  |
|  |  | GM10037 | |  |
|  |  | PSPH |  |  |
|  |  | PANK4 |  |  |
|  |  | GM3468 |  |  |
|  |  | ERF |  |  |
|  |  | FAM228A | |  |
|  |  | FMNL1 |  |  |
|  |  | RHNO1 |  |  |
|  |  | NCOR1 |  |  |
|  |  | TUBB5 |  |  |
|  |  | ZFP433 |  |  |
|  |  | MZT1 |  |  |
|  |  | MINDY1 |  |  |
|  |  | SLC9A5 |  |  |
|  |  | NHLRC4 |  |  |
|  |  | TEX11 |  |  |
|  |  | WDCP |  |  |
|  |  | NAIF1 |  |  |
|  |  | MRPL14 |  |  |
|  |  | LMNTD1 | |  |
|  |  | MINOS1 |  |  |
|  |  | PRPS2 |  |  |
|  |  | PPP1R16B | |  |
|  |  | KLHL21 |  |  |
|  |  | ALDOB |  |  |
|  |  | KLRA2 |  |  |
|  |  | CCL25 |  |  |
|  |  | 3110009E18RIK | |  |
|  |  | THEGL |  |  |
|  |  | IL1R2 |  |  |
|  |  | TMEM184A | |  |
|  |  | ODF3B |  |  |
|  |  | ADAMTS16 | |  |
|  |  | SYNE1 |  |  |
|  |  | 9030025P20RIK | |  |
|  |  | STX18 |  |  |
|  |  | CLK2 |  |  |
|  |  | EIF4A3 |  |  |
|  |  | SRP19 |  |  |
|  |  | UXT |  |  |
|  |  | RPL17 |  |  |
|  |  | CRAMP1L | |  |
|  |  | 1110002E22RIK | |  |
|  |  | GOLGA7 |  |  |
|  |  | ZC3H12B | |  |
|  |  | AKAP12 |  |  |
|  |  | JARID2 |  |  |
|  |  | TGDS |  |  |
|  |  | SIX4 |  |  |
|  |  | SMG7 |  |  |
|  |  | ZPBP |  |  |
|  |  | MFSD13A | |  |
|  |  | SEC31B |  |  |
|  |  | BC003965 | |  |
|  |  | ARHGAP4 | |  |
|  |  | A930009A15RIK | |  |
|  |  | HMGN3 |  |  |
|  |  | ZFP429 |  |  |
|  |  | PLS1 |  |  |
|  |  | CORIN |  |  |
|  |  | IGFBP1 |  |  |
|  |  | MEP1B |  |  |
|  |  | ADAM21 |  |  |
|  |  | COX6A2 |  |  |
|  |  | XYLB |  |  |
|  |  | ACBD3 |  |  |
|  |  | FAM133B | |  |
|  |  | ADGB |  |  |
|  |  | TMCO1 |  |  |
|  |  | PRELID1 |  |  |
|  |  | TMEM116 | |  |
|  |  | CHRAC1 |  |  |
|  |  | SNAP91 |  |  |
|  |  | CDHR2 |  |  |
|  |  | OLFR691 |  |  |
|  |  | PTGIR |  |  |
|  |  | GM8369 |  |  |
|  |  | FAM243 |  |  |
|  |  | AKR1B7 |  |  |
|  |  | SLC17A2 |  |  |
|  |  | HORMAD2 | |  |
|  |  | OLFR1329 | |  |
|  |  | F930015N05RIK | |  |
|  |  | GM37419 | |  |
|  |  | ADGRG7 |  |  |
|  |  | 4930535I16RIK | |  |
|  |  | TEX33 |  |  |
|  |  | 8030462N17RIK | |  |
|  |  | WFDC21 |  |  |
|  |  | UGT2B1 |  |  |
|  |  | TRIM55 |  |  |
|  |  | DCSTAMP | |  |
|  |  | SCG2 |  |  |
|  |  | TM2D2 |  |  |
|  |  | TRIP4 |  |  |
|  |  | RWDD2A | |  |
|  |  | NAPB |  |  |
|  |  | TSSK3 |  |  |
|  |  | ZBTB26 |  |  |
|  |  | TNPO3 |  |  |
|  |  | LEKR1 |  |  |
|  |  | TATDN1 |  |  |
|  |  | MYOG |  |  |
|  |  | PIKFYVE | |  |
|  |  | GOT1L1 |  |  |
|  |  | NOTUM |  |  |
|  |  | AHCTF1 |  |  |
|  |  | RFX4 |  |  |
|  |  | ZFP119A |  |  |
|  |  | MORC2A | |  |
|  |  | HIBCH |  |  |
|  |  | TEX43 |  |  |
|  |  | PRELID3A | |  |
|  |  | SOS1 |  |  |
|  |  | ZC3HAV1L | |  |
|  |  | MTMR9 |  |  |
|  |  | RPP25L |  |  |
|  |  | VPS16 |  |  |
|  |  | ARF2 |  |  |
|  |  | NDUFA4 |  |  |
|  |  | ACE3 |  |  |
|  |  | PPP1R14D | |  |
|  |  | PAX2 |  |  |
|  |  | HSD17B2 | |  |
|  |  | TFAP2E |  |  |
|  |  | WDR31 |  |  |
|  |  | RSPH4A |  |  |
|  |  | COMP |  |  |
|  |  | D030056L22RIK | |  |
|  |  | UGP2 |  |  |
|  |  | QRFPR |  |  |
|  |  | RRBP1 |  |  |
|  |  | KYAT1 |  |  |
|  |  | GRAMD1B | |  |
|  |  | RLIM |  |  |
|  |  | CBY1 |  |  |
|  |  | ZMYND11 | |  |
|  |  | PLEKHG3 | |  |
|  |  | LSM8 |  |  |
|  |  | RAB38 |  |  |
|  |  | WDR76 |  |  |
|  |  | COPS4 |  |  |
|  |  | SENP5 |  |  |
|  |  | DGKA |  |  |
|  |  | ATL2 |  |  |
|  |  | CCNDBP1 | |  |
|  |  | TRIM28 |  |  |
|  |  | PTBP2 |  |  |
|  |  | GRM2 |  |  |
|  |  | CASP4 |  |  |
|  |  | FARS2 |  |  |
|  |  | AIDA |  |  |
|  |  | SRMS |  |  |
|  |  | DNAJB3 |  |  |
|  |  | CCDC27 |  |  |
|  |  | SCD2 |  |  |
|  |  | HPCA |  |  |
|  |  | ADRA1A |  |  |
|  |  | GPATCH8 | |  |
|  |  | EMG1 |  |  |
|  |  | GATA2 |  |  |
|  |  | TAP1 |  |  |
|  |  | APC2 |  |  |
|  |  | XYLT2 |  |  |
|  |  | PRR19 |  |  |
|  |  | OAS3 |  |  |
|  |  | EFNB3 |  |  |
|  |  | DCUN1D5 | |  |
|  |  | ATP1B4 |  |  |
|  |  | CCL24 |  |  |
|  |  | GM5431 |  |  |
|  |  | TAPBP |  |  |
|  |  | SIDT1 |  |  |
|  |  | APLNR |  |  |
|  |  | PTPRN2 |  |  |
|  |  | ALKBH7 |  |  |
|  |  | INAVA |  |  |
|  |  | HIST1H2BL | |  |
|  |  | KLHL36 |  |  |
|  |  | TCHP |  |  |
|  |  | RNF8 |  |  |
|  |  | MAZ |  |  |
|  |  | FTSJ1 |  |  |
|  |  | 9430069I07RIK | |  |
|  |  | STX12 |  |  |
|  |  | TMEM256 | |  |
|  |  | OSMR |  |  |
|  |  | CTNNAL1 | |  |
|  |  | BMP2K |  |  |
|  |  | ZFP511 |  |  |
|  |  | STRADA |  |  |
|  |  | LCLAT1 |  |  |
|  |  | F9 |  |  |
|  |  | GIT2 |  |  |
|  |  | LRRCC1 |  |  |
|  |  | PFN1 |  |  |
|  |  | RPS6KB1 | |  |
|  |  | SPATA45 | |  |
|  |  | RASSF6 |  |  |
|  |  | BC004004 | |  |
|  |  | VPS29 |  |  |
|  |  | RETSAT |  |  |
|  |  | PDCD6 |  |  |
|  |  | PTGER3 |  |  |
|  |  | ZFP677 |  |  |
|  |  | METTL23 | |  |
|  |  | COX11 |  |  |
|  |  | BGLAP2 |  |  |
|  |  | SKINT1 |  |  |
|  |  | ASB10 |  |  |
|  |  | KANK3 |  |  |
|  |  | VMAC |  |  |
|  |  | ARHGEF12 | |  |
|  |  | RPS11 |  |  |
|  |  | 4930522L14RIK | |  |
|  |  | BRIP1 |  |  |
|  |  | CLTA |  |  |
|  |  | ANKS1B |  |  |
|  |  | EFCAB11 | |  |
|  |  | PCDHB2 |  |  |
|  |  | DOK2 |  |  |
|  |  | GUCY2C |  |  |
|  |  | VSTM2B |  |  |
|  |  | EXOC7 |  |  |
|  |  | DUPD1 |  |  |
|  |  | GM3854 |  |  |
|  |  | CYM |  |  |
|  |  | GM10974 | |  |
|  |  | PLA2R1 |  |  |
|  |  | HAS2 |  |  |
|  |  | BAG6 |  |  |
|  |  | PKDREJ |  |  |
|  |  | TRMT5 |  |  |
|  |  | TPO |  |  |
|  |  | DAPK2 |  |  |
|  |  | RFXANK |  |  |
|  |  | TSKS |  |  |
|  |  | CBY3 |  |  |
|  |  | OTC |  |  |
|  |  | NME6 |  |  |
|  |  | ATG10 |  |  |
|  |  | ACOT3 |  |  |
|  |  | HSP90AA1 | |  |
|  |  | NFATC4 |  |  |
|  |  | MFSD5 |  |  |
|  |  | VPS37B |  |  |
|  |  | PIP5K1A |  |  |
|  |  | CHST15 |  |  |
|  |  | LRRC72 |  |  |
|  |  | MPIG6B |  |  |
|  |  | TRIL |  |  |
|  |  | PTRH2 |  |  |
|  |  | ECE2 |  |  |
|  |  | ANKRD2 |  |  |
|  |  | RHBDL2 |  |  |
|  |  | AP5S1 |  |  |
|  |  | NR4A1 |  |  |
|  |  | GMPPA |  |  |
|  |  | AOX2 |  |  |
|  |  | GPATCH1 | |  |
|  |  | SMIM27 |  |  |
|  |  | TMCO3 |  |  |
|  |  | FER |  |  |
|  |  | GSTM5 |  |  |
|  |  | ITGA9 |  |  |
|  |  | VAV1 |  |  |
|  |  | MAPRE2 |  |  |
|  |  | BRCC3 |  |  |
|  |  | 4933407L21RIK | |  |
|  |  | APH1A |  |  |
|  |  | EHBP1 |  |  |
|  |  | BNC2 |  |  |
|  |  | CRNKL1 |  |  |
|  |  | SNCG |  |  |
|  |  | GYS1 |  |  |
|  |  | SEC16A |  |  |
|  |  | SFRP2 |  |  |
|  |  | CCDC96 |  |  |
|  |  | 1700023F06RIK | |  |
|  |  | PTPRT |  |  |
|  |  | METAP2 |  |  |
|  |  | WDR75 |  |  |
|  |  | HCRTR2 |  |  |
|  |  | CHN2 |  |  |
|  |  | UTP6 |  |  |
|  |  | FMC1 |  |  |
|  |  | TMEM121 | |  |
|  |  | CRYAA |  |  |
|  |  | ASPDH |  |  |
|  |  | PRLHR |  |  |
|  |  | SPTSSB |  |  |
|  |  | SLC7A4 |  |  |
|  |  | PHKA1 |  |  |
|  |  | TMEM119 | |  |
|  |  | NCBP2 |  |  |
|  |  | MED1 |  |  |
|  |  | MCPH1 |  |  |
|  |  | COMMD7 | |  |
|  |  | ATP9A |  |  |
|  |  | OLFR1034 | |  |
|  |  | NMU |  |  |
|  |  | ADGRF1 |  |  |
|  |  | TNFRSF13C | |  |
|  |  | GM4847 |  |  |
|  |  | SHISA5 |  |  |
|  |  | CDCA2 |  |  |
|  |  | UFSP1 |  |  |
|  |  | TOR1A |  |  |
|  |  | P4HB |  |  |
|  |  | LIG1 |  |  |
|  |  | MDFIC |  |  |
|  |  | BC005624 | |  |
|  |  | 4930426L09RIK | |  |
|  |  | CFAP299 |  |  |
|  |  | ABHD10 |  |  |
|  |  | AURKAIP1 | |  |
|  |  | TAF12 |  |  |
|  |  | LINS1 |  |  |
|  |  | DNAJB7 |  |  |
|  |  | GNPDA1 |  |  |
|  |  | CYB5D2 |  |  |
|  |  | ARMCX2 | |  |
|  |  | ZFP791 |  |  |
|  |  | ZFP182 |  |  |
|  |  | SETDB2 |  |  |
|  |  | FAM217B | |  |
|  |  | DGKQ |  |  |
|  |  | HBA-A1 |  |  |
|  |  | TMEM255B | |  |
|  |  | CCDC167 | |  |
|  |  | WFDC17 |  |  |
|  |  | DPF2 |  |  |
|  |  | HP |  |  |
|  |  | OLIG1 |  |  |
|  |  | UBIAD1 |  |  |
|  |  | TEX261 |  |  |
|  |  | KCNRG |  |  |
|  |  | NIT2 |  |  |
|  |  | UBE2E3 |  |  |
|  |  | COQ10A |  |  |
|  |  | ACSL6 |  |  |
|  |  | CD52 |  |  |
|  |  | 1700037C18RIK | |  |
|  |  | CLDN12 |  |  |
|  |  | DMWD |  |  |
|  |  | HS3ST4 |  |  |
|  |  | GM11273 | |  |
|  |  | STMND1 |  |  |
|  |  | CCR6 |  |  |
|  |  | PIN1 |  |  |
|  |  | RPL32 |  |  |
|  |  | HTRA3 |  |  |
|  |  | A |  |  |
|  |  | SLC41A1 |  |  |
|  |  | MORC3 |  |  |
|  |  | DZIP1 |  |  |
|  |  | TMCO6 |  |  |
|  |  | DTX2 |  |  |
|  |  | GCC1 |  |  |
|  |  | SMIM18 |  |  |
|  |  | ASB15 |  |  |
|  |  | OLFR1427 | |  |
|  |  | OLFR58 |  |  |
|  |  | RAB7 |  |  |
|  |  | ALOX5AP | |  |
|  |  | ACAD9 |  |  |
|  |  | SMARCE1 | |  |
|  |  | EID3 |  |  |
|  |  | MINDY4B-PS | |  |
|  |  | FKBPL |  |  |
|  |  | CDK6 |  |  |
|  |  | FBXO21 |  |  |
|  |  | TBC1D16 | |  |
|  |  | ZFP563 |  |  |
|  |  | OLFR316 |  |  |
|  |  | SLC22A6 |  |  |
|  |  | CATIP |  |  |
|  |  | ADCY4 |  |  |
|  |  | TRIM75 |  |  |
|  |  | DBX2 |  |  |
|  |  | PIGH |  |  |
|  |  | ESR1 |  |  |
|  |  | POLE4 |  |  |
|  |  | FANCD2 |  |  |
|  |  | RDH10 |  |  |
|  |  | CTNNA1 |  |  |
|  |  | XRN1 |  |  |
|  |  | OSBPL11 | |  |
|  |  | TMPRSS7 | |  |
|  |  | MCM10 |  |  |
|  |  | SLC36A4 |  |  |
|  |  | CCT8L1 |  |  |
|  |  | IL7R |  |  |
|  |  | PDIA6 |  |  |
|  |  | DAAM2 |  |  |
|  |  | LRP8 |  |  |
|  |  | GRIN2C |  |  |
|  |  | OLFR458 |  |  |
|  |  | DHX29 |  |  |
|  |  | STX11 |  |  |
|  |  | PHYKPL |  |  |
|  |  | USP1 |  |  |
|  |  | CCNF |  |  |
|  |  | CCL5 |  |  |
|  |  | MKRN2OS | |  |
|  |  | PNMA1 |  |  |
|  |  | FBXL19 |  |  |
|  |  | PDSS1 |  |  |
|  |  | FRAT1 |  |  |
|  |  | GM3752 |  |  |
|  |  | EHD2 |  |  |
|  |  | RILP |  |  |
|  |  | NPTX2 |  |  |
|  |  | FAM151A | |  |
|  |  | UBE2W |  |  |
|  |  | DNAAF3 |  |  |
|  |  | SLIRP |  |  |
|  |  | OLFR464 |  |  |
|  |  | SERPINB1A | |  |
|  |  | AXIN2 |  |  |
|  |  | ZFP451 |  |  |
|  |  | BPIFB9B |  |  |
|  |  | CPN1 |  |  |
|  |  | EML5 |  |  |
|  |  | CHKA |  |  |
|  |  | CTF1 |  |  |
|  |  | RTL1 |  |  |
|  |  | HBB-Y |  |  |
|  |  | OLFR555 |  |  |
|  |  | GABRA6 |  |  |
|  |  | ZFP784 |  |  |
|  |  | KRT86 |  |  |
|  |  | GM5916 |  |  |
|  |  | A730071L15RIK | |  |
|  |  | ZFP768 |  |  |
|  |  | GEMIN5 |  |  |
|  |  | A730017C20RIK | |  |
|  |  | SYCE2 |  |  |
|  |  | PSMB1 |  |  |
|  |  | CHGB |  |  |
|  |  | DKK2 |  |  |
|  |  | PELO |  |  |
|  |  | DNMT3B | |  |
|  |  | NR1H3 |  |  |
|  |  | EMC8 |  |  |
|  |  | ABCC2 |  |  |
|  |  | ACSM4 |  |  |
|  |  | DRAM1 |  |  |
|  |  | FASL |  |  |
|  |  | GRB7 |  |  |
|  |  | PLEKHA7 | |  |
|  |  | CSNK1G3 | |  |
|  |  | TMPRSS2 | |  |
|  |  | PTOV1 |  |  |
|  |  | MIB1 |  |  |
|  |  | SLC24A1 |  |  |
|  |  | CDV3 |  |  |
|  |  | PHACTR3 | |  |
|  |  | PTPN11 |  |  |
|  |  | GCNT4 |  |  |
|  |  | GPX7 |  |  |
|  |  | CDRT4 |  |  |
|  |  | VKORC1 |  |  |
|  |  | KCNK7 |  |  |
|  |  | FAM204A | |  |
|  |  | ZFP526 |  |  |
|  |  | CRIPT |  |  |
|  |  | METTL21A | |  |
|  |  | SLC20A2 |  |  |
|  |  | VAT1L |  |  |
|  |  | GM10031 | |  |
|  |  | HERPUD1 | |  |
|  |  | NPPA |  |  |
|  |  | CASP6 |  |  |
|  |  | KCTD3 |  |  |
|  |  | CYB561 |  |  |
|  |  | SLC25A33 | |  |
|  |  | FXYD6 |  |  |
|  |  | PSMB6 |  |  |
|  |  | CYR61 |  |  |
|  |  | KCNV2 |  |  |
|  |  | PTGES2 |  |  |
|  |  | USP30 |  |  |
|  |  | CEACAM16 | |  |
|  |  | GM21994 | |  |
|  |  | IRF6 |  |  |
|  |  | TBC1D12 | |  |
|  |  | PLCD3 |  |  |
|  |  | TBC1D2 |  |  |
|  |  | LRCH1 |  |  |
|  |  | GM14403 | |  |
|  |  | 3-Sep |  |  |
|  |  | FKBP5 |  |  |
|  |  | ERP29 |  |  |
|  |  | ADSS |  |  |
|  |  | USP53 |  |  |
|  |  | PPP1R10 |  |  |
|  |  | RXFP2 |  |  |
|  |  | SKIV2L2 |  |  |
|  |  | CD99L2 |  |  |
|  |  | GRWD1 |  |  |
|  |  | TXNL1 |  |  |
|  |  | LYRM4 |  |  |
|  |  | SGSH |  |  |
|  |  | OGFOD1 |  |  |
|  |  | ZFP687 |  |  |
|  |  | 1700019A02RIK | |  |
|  |  | OLFR559 |  |  |
|  |  | CLEC4A1 | |  |
|  |  | ACACB |  |  |
|  |  | PDZD2 |  |  |
|  |  | DOK7 |  |  |
|  |  | CYB5D1 |  |  |
|  |  | WDR24 |  |  |
|  |  | ETFBKMT | |  |
|  |  | ZFP637 |  |  |
|  |  | RRM1 |  |  |
|  |  | SHCBP1L | |  |
|  |  | DIP2A |  |  |
|  |  | NXPH2 |  |  |
|  |  | GREB1L |  |  |
|  |  | ZFP777 |  |  |
|  |  | MPP5 |  |  |
|  |  | TBC1D20 | |  |
|  |  | GM2244 |  |  |
|  |  | PHETA2 |  |  |
|  |  | 2900055J20RIK | |  |
|  |  | ING2 |  |  |
|  |  | 4933424G06RIK | |  |
|  |  | WFDC18 |  |  |
|  |  | TSC22D4 | |  |
|  |  | TOR4A |  |  |
|  |  | RABL3 |  |  |
|  |  | GAMT |  |  |
|  |  | MRM3 |  |  |
|  |  | RSL1 |  |  |
|  |  | TESC |  |  |
|  |  | TKTL1 |  |  |
|  |  | VNN1 |  |  |
|  |  | GOLPH3L | |  |
|  |  | RGS18 |  |  |
|  |  | IFNK |  |  |
|  |  | IFT80 |  |  |
|  |  | PCDHGB4 | |  |
|  |  | EDN1 |  |  |
|  |  | FARSB |  |  |
|  |  | 4930402H24RIK | |  |
|  |  | SULT5A1 | |  |
|  |  | ARHGDIA | |  |
|  |  | DNAH3 |  |  |
|  |  | EFHD1 |  |  |
|  |  | FABP4 |  |  |
|  |  | DNAJC17 | |  |
|  |  | POLG |  |  |
|  |  | GM5475 |  |  |
|  |  | PVRIG |  |  |
|  |  | SLN |  |  |
|  |  | NR2E1 |  |  |
|  |  | EPHX3 |  |  |
|  |  | GM49027 | |  |
|  |  | PPIE |  |  |
|  |  | MAGI2 |  |  |
|  |  | PABPN1 |  |  |
|  |  | HSBP1L1 | |  |
|  |  | GM11111 | |  |
|  |  | GM1553 |  |  |
|  |  | LRIT3 |  |  |
|  |  | GM5089 |  |  |
|  |  | KLHDC8A | |  |
|  |  | PBLD1 |  |  |
|  |  | NXF2 |  |  |
|  |  | HSD17B13 | |  |
|  |  | CHMP4C |  |  |
|  |  | TLE2 |  |  |
|  |  | PCDHGB1 | |  |
|  |  | LRRC71 |  |  |
|  |  | PDXK |  |  |
|  |  | GABRQ |  |  |
|  |  | D16ERTD472E | |  |
|  |  | SPATA4 |  |  |
|  |  | HAUS8 |  |  |
|  |  | FAM110B | |  |
|  |  | ABHD12B | |  |
|  |  | ADAM19 |  |  |
|  |  | 4930518I15RIK | |  |
|  |  | PELP1 |  |  |
|  |  | H2AFB3 |  |  |
|  |  | CLIP3 |  |  |
|  |  | RINL |  |  |
|  |  | ZBTB46 |  |  |
|  |  | PCSK6 |  |  |
|  |  | B4GALT7 | |  |
|  |  | GAPDH |  |  |
|  |  | ELFN1 |  |  |
|  |  | PRPF18 |  |  |
|  |  | COL5A2 |  |  |
|  |  | RAB11A |  |  |
|  |  | IGTP |  |  |
|  |  | TPP1 |  |  |
|  |  | CCT5 |  |  |
|  |  | CEACAM1 | |  |
|  |  | TMEM69 |  |  |
|  |  | PBX3 |  |  |
|  |  | POLR2B |  |  |
|  |  | COA6 |  |  |
|  |  | GCNT1 |  |  |
|  |  | PSPC1 |  |  |
|  |  | MED11 |  |  |
|  |  | PCF11 |  |  |
|  |  | FAM24A |  |  |
|  |  | THEM4 |  |  |
|  |  | USE1 |  |  |
|  |  | RACK1 |  |  |
|  |  | SLC23A2 |  |  |
|  |  | ANO10 |  |  |
|  |  | KCNH6 |  |  |
|  |  | SLC9A3R2 | |  |
|  |  | TMEM251 | |  |
|  |  | TUBE1 |  |  |
|  |  | MCRIP1 |  |  |
|  |  | CDC26 |  |  |
|  |  | ZBTB4 |  |  |
|  |  | PCDHGA5 | |  |
|  |  | BICRAL |  |  |
|  |  | R3HDM2 |  |  |
|  |  | SART3 |  |  |
|  |  | ASF1A |  |  |
|  |  | FPR2 |  |  |
|  |  | TNFSFM13 | |  |
|  |  | OLFR1317 | |  |
|  |  | CCT6B |  |  |
|  |  | PHC2 |  |  |
|  |  | RRAS |  |  |
|  |  | EIF4E |  |  |
|  |  | FAM160B1 | |  |
|  |  | IFNAR1 |  |  |
|  |  | ARV1 |  |  |
|  |  | ATP6V0E | |  |
|  |  | TLR7 |  |  |
|  |  | MGST2 |  |  |
|  |  | TBK1 |  |  |
|  |  | OLFR76 |  |  |
|  |  | GM9936 |  |  |
|  |  | CBX5 |  |  |
|  |  | ELF1 |  |  |
|  |  | RPS12 |  |  |
|  |  | CEP135 |  |  |
|  |  | MCM8 |  |  |
|  |  | ZFP207 |  |  |
|  |  | ZSCAN21 | |  |
|  |  | VPS33B |  |  |
|  |  | MSH3 |  |  |
|  |  | LIN37 |  |  |
|  |  | RPL7A |  |  |
|  |  | GM14288 | |  |
|  |  | NAGA |  |  |
|  |  | KDM8 |  |  |
|  |  | DENND6B | |  |
|  |  | KYAT3 |  |  |
|  |  | SUPT20 |  |  |
|  |  | NOP53 |  |  |
|  |  | SNX24 |  |  |
|  |  | GM14391 | |  |
|  |  | PELI2 |  |  |
|  |  | RFX8 |  |  |
|  |  | ADAM22 |  |  |
|  |  | MMP23 |  |  |
|  |  | MAGI1 |  |  |
|  |  | DAPK3 |  |  |
|  |  | MYEF2 |  |  |
|  |  | FAM32A |  |  |
|  |  | ESYT1 |  |  |
|  |  | SLF1 |  |  |
|  |  | NRG1 |  |  |
|  |  | GM34302 | |  |
|  |  | TMED4 |  |  |
|  |  | CLEC10A | |  |
|  |  | ANKRD24 | |  |
|  |  | PPP1R12C | |  |
|  |  | PRKD3 |  |  |
|  |  | PUS7 |  |  |
|  |  | VAMP3 |  |  |
|  |  | FKBP4 |  |  |
|  |  | ADAM10 |  |  |
|  |  | STX8 |  |  |
|  |  | QSOX1 |  |  |
|  |  | DNAJC30 | |  |
|  |  | GALNT10 | |  |
|  |  | MARK3 |  |  |
|  |  | CDKL2 |  |  |
|  |  | URM1 |  |  |
|  |  | MARVELD3 | |  |
|  |  | EDA |  |  |
|  |  | SAC3D1 |  |  |
|  |  | YEATS4 |  |  |
|  |  | PEX1 |  |  |
|  |  | NDUFB9 |  |  |
|  |  | NODAL |  |  |
|  |  | RAB13 |  |  |
|  |  | KANSL2 |  |  |
|  |  | CLEC16A | |  |
|  |  | CIR1 |  |  |
|  |  | HIST1H3I | |  |
|  |  | RNF212B | |  |
|  |  | TRIM13 |  |  |
|  |  | PAQR5 |  |  |
|  |  | LIME1 |  |  |
|  |  | GM10131 | |  |
|  |  | MMGT2 |  |  |
|  |  | CCNG1 |  |  |
|  |  | MITD1 |  |  |
|  |  | PCDHB18 | |  |
|  |  | MBOAT2 | |  |
|  |  | SFN |  |  |
|  |  | GM3696 |  |  |
|  |  | RHOC |  |  |
|  |  | ZC3H6 |  |  |
|  |  | PEX3 |  |  |
|  |  | DPP4 |  |  |
|  |  | GIPC2 |  |  |
|  |  | SKA3 |  |  |
|  |  | CYBA |  |  |
|  |  | LY6G6F |  |  |
|  |  | RPL10 |  |  |
|  |  | ZFP52 |  |  |
|  |  | FAM72A |  |  |
|  |  | DOCK5 |  |  |
|  |  | IL11 |  |  |
|  |  | HIST1H4A | |  |
|  |  | TEKT4 |  |  |
|  |  | CTNND2 |  |  |
|  |  | QRICH1 |  |  |
|  |  | WWC1 |  |  |
|  |  | RDH16 |  |  |
|  |  | SRD5A2 |  |  |
|  |  | MSH5 |  |  |
|  |  | KCTD2 |  |  |
|  |  | KMO |  |  |
|  |  | GM4791 |  |  |
|  |  | HIST1H2BG | |  |
|  |  | RNPC3 |  |  |
|  |  | ATP6V1A | |  |
|  |  | CEP120 |  |  |
|  |  | CMTM5 |  |  |
|  |  | BEND3 |  |  |
|  |  | SETD2 |  |  |
|  |  | IRX1 |  |  |
|  |  | POLH |  |  |
|  |  | GM42669 | |  |
|  |  | GM10518 | |  |
|  |  | GM9972 |  |  |
|  |  | CEACAM10 | |  |
|  |  | VMN1R209 | |  |
|  |  | MAB21L2 | |  |
|  |  | APOA2 |  |  |
|  |  | FBXO5 |  |  |
|  |  | GOLPH3 |  |  |
|  |  | GAB2 |  |  |
|  |  | STRN4 |  |  |
|  |  | SLC2A10 |  |  |
|  |  | ZFP944 |  |  |
|  |  | EMILIN3 |  |  |
|  |  | YTHDF1 |  |  |
|  |  | GPR37 |  |  |
|  |  | ITFG1 |  |  |
|  |  | NDUFA5 |  |  |
|  |  | MUC3A |  |  |
|  |  | ATG101 |  |  |
|  |  | TRAM1L1 | |  |
|  |  | TLR3 |  |  |
|  |  | EHHADH | |  |
|  |  | SNX19 |  |  |
|  |  | OXT |  |  |
|  |  | LHX3 |  |  |
|  |  | INSIG2 |  |  |
|  |  | GM10681 | |  |
|  |  | GM8225 |  |  |
|  |  | OLFR561 |  |  |
|  |  | BMP8A |  |  |
|  |  | SLC12A6 |  |  |
|  |  | EMC7 |  |  |
|  |  | SRI |  |  |
|  |  | SNX31 |  |  |
|  |  | MAPKAP1 | |  |
|  |  | CCDC173 | |  |
|  |  | GPR20 |  |  |
|  |  | OLFR556 |  |  |
|  |  | GM6993 |  |  |
|  |  | DOXL2 |  |  |
|  |  | AFM |  |  |
|  |  | DIO1 |  |  |
|  |  | ILTIFB |  |  |
|  |  | GM5592 |  |  |
|  |  | 4933409G03RIK | |  |
|  |  | GH |  |  |
|  |  | OLFR211 |  |  |
|  |  | OLFR229 |  |  |
|  |  | SLCO1A1 | |  |
|  |  | GM17027 | |  |
|  |  | FSCB |  |  |
|  |  | APOA4 |  |  |
|  |  | FSHR |  |  |
|  |  | OLFR275 |  |  |
|  |  | GM6594 |  |  |
|  |  | GM17521 | |  |
|  |  | SERPINA16 | |  |
|  |  | GPR141 |  |  |
|  |  | SERPINB9C | |  |
|  |  | GM5108 |  |  |
|  |  | KRTAP9-3 | |  |
|  |  | OOEP |  |  |
|  |  | GIP |  |  |
|  |  | GP6 |  |  |
|  |  | LRRC30 |  |  |
|  |  | GM42421 | |  |
|  |  | 1700069L16RIK | |  |
|  |  | GM17416 | |  |
|  |  | SPRR1A |  |  |
|  |  | FETUB |  |  |
|  |  | GABRP |  |  |
|  |  | FAM205A2 | |  |
|  |  | OLFR765 |  |  |
|  |  | CD209E |  |  |
|  |  | GJA10 |  |  |
|  |  | MS4A5 |  |  |
|  |  | OLFR881 |  |  |
|  |  | OLFR985 |  |  |
|  |  | OR5BS1P | |  |
|  |  | FAM71E2 | |  |
|  |  | HIST1H2BA | |  |
|  |  | VMN2R82 | |  |
|  |  | 4932415D10RIK | |  |
|  |  | GM10945 | |  |
|  |  | OLFR338 |  |  |
|  |  | VMN1R57 | |  |
|  |  | AICDA |  |  |
|  |  | CSF2 |  |  |
|  |  | PPP2R5B | |  |
|  |  | VMN1R86 | |  |
|  |  | OLFR1260 | |  |
|  |  | OLFR406 |  |  |
|  |  | RASGRP2 | |  |
|  |  | OLFR437 |  |  |
|  |  | OLFR452 |  |  |
|  |  | OLFR459 |  |  |
|  |  | OLFR441 |  |  |
|  |  | FOXE3 |  |  |
|  |  | MRPS23 |  |  |
|  |  | OLFR1128 | |  |
|  |  | OLFR1120 | |  |
|  |  | OLFR521 |  |  |
|  |  | RHOX2F |  |  |
|  |  | OLFR1324 | |  |
|  |  | MUC20 |  |  |
|  |  | MCPT4 |  |  |
|  |  | KCTD19 |  |  |
|  |  | NR1H5 |  |  |
|  |  | MS4A10 |  |  |
|  |  | CES2F |  |  |
|  |  | VSIG4 |  |  |
|  |  | SECTM1B | |  |
|  |  | GM26727 | |  |
|  |  | IL10 |  |  |
|  |  | UNCX |  |  |
|  |  | KLK1B3 |  |  |
|  |  | CRYGE |  |  |
|  |  | OLFR271-PS1 | |  |
|  |  | GM12845 | |  |
|  |  | PITPNM1 | |  |
|  |  | CEP170B |  |  |
|  |  | GM10234 | |  |
|  |  | GM2888 |  |  |
|  |  | OLFR60 |  |  |
|  |  | GM595 |  |  |
|  |  | GM597 |  |  |
|  |  | NCCRP1 |  |  |
|  |  | TRAP1A |  |  |
|  |  | OLFR47 |  |  |
|  |  | MBD3L1 |  |  |
|  |  | LIPO5 |  |  |
|  |  | UTF1 |  |  |
|  |  | GM2431 |  |  |
|  |  | BTBD35F7 | |  |
|  |  | 4930503B20RIK | |  |
|  |  | GM14226 | |  |
|  |  | GM15517 | |  |
|  |  | KRTAP12-1 | |  |
|  |  | GM3646 |  |  |
|  |  | GM3685 |  |  |
|  |  | KRT78 |  |  |
|  |  | GM15217 | |  |
|  |  | AUTS2 |  |  |
|  |  | PLAC1 |  |  |
|  |  | SPIC |  |  |
|  |  | GM4894 |  |  |
|  |  | MRGPRA3 | |  |
|  |  | 1700018B08RIK | |  |
|  |  | PADI3 |  |  |
|  |  | GM3409 |  |  |
|  |  | CD3G |  |  |
|  |  | GM13277 | |  |
|  |  | 4930433I11RIK | |  |
|  |  | GM26602 | |  |
|  |  | GM13305 | |  |
|  |  | GM38655 | |  |
|  |  | 4921530L21RIK | |  |
|  |  | KLF17 |  |  |
|  |  | OBP2B |  |  |
|  |  | ZHX1 |  |  |
|  |  | REG3D |  |  |
|  |  | RANBP10 | |  |
|  |  | DNAJB5 |  |  |
|  |  | CDK9 |  |  |
|  |  | ANKRD63 | |  |
|  |  | GM3839 |  |  |
|  |  | TPX2 |  |  |
|  |  | SNX13 |  |  |
|  |  | MPP6 |  |  |
|  |  | HEATR6 |  |  |
|  |  | KCNK12 |  |  |
|  |  | INPPL1 |  |  |
|  |  | MCRS1 |  |  |
|  |  | NCOA5 |  |  |
|  |  | NOX1 |  |  |
|  |  | RMDN1 |  |  |
|  |  | VHL |  |  |
|  |  | GTPBP8 |  |  |
|  |  | PIFO |  |  |
|  |  | TEKT2 |  |  |
|  |  | TEKT5 |  |  |
|  |  | PELI1 |  |  |
|  |  | OAS1G |  |  |
|  |  | SFT2D2 |  |  |
|  |  | FGF23 |  |  |
|  |  | PIR |  |  |
|  |  | 1500009L16RIK | |  |
|  |  | NDUFB7 |  |  |
|  |  | DCXR |  |  |
|  |  | HENMT1 | |  |
|  |  | GKAP1 |  |  |
|  |  | TGS1 |  |  |
|  |  | SLC25A40 | |  |
|  |  | HPS1 |  |  |
|  |  | MYH2 |  |  |
|  |  | CCIN |  |  |
|  |  | IL6 |  |  |
|  |  | 6030468B19RIK | |  |
|  |  | CALHM5 |  |  |
|  |  | 2410131K14RIK | |  |
|  |  | ETL4 |  |  |
|  |  | SMC5 |  |  |
|  |  | LRFN4 |  |  |
|  |  | WRB |  |  |
|  |  | VARS |  |  |
|  |  | TMEM165 | |  |
|  |  | FOXL2 |  |  |
|  |  | ADAM5 |  |  |
|  |  | KLHL41 |  |  |
|  |  | LRFN5 |  |  |
|  |  | OLFR538 |  |  |
|  |  | PLA2G4F | |  |
|  |  | AI661453 |  |  |
|  |  | MAML1 |  |  |
|  |  | SHE |  |  |
|  |  | ISOC2B |  |  |
|  |  | ADAMTS10 | |  |
|  |  | KIF13A |  |  |
|  |  | PNLIP |  |  |
|  |  | TMUB2 |  |  |
|  |  | KCTD11 |  |  |
|  |  | HIST2H2AB | |  |
|  |  | NUDT22 |  |  |
|  |  | NT5M |  |  |
|  |  | ATP5MPL | |  |
|  |  | LAPTM5 |  |  |
|  |  | LUZP1 |  |  |
|  |  | MELTF |  |  |
|  |  | ZFP787 |  |  |
|  |  | BIRC5 |  |  |
|  |  | 3830406C13RIK | |  |
|  |  | TIMM44 |  |  |
|  |  | ZC3H12A | |  |
|  |  | ALDH3A1 | |  |
|  |  | KTN1 |  |  |
|  |  | GM20661 | |  |
|  |  | YIPF7 |  |  |
|  |  | HRG |  |  |
|  |  | MYF5 |  |  |
|  |  | ZSCAN29 | |  |
|  |  | SPATS2L | |  |
|  |  | KLHL26 |  |  |
|  |  | FZD9 |  |  |
|  |  | GTF2IRD1 | |  |
|  |  | CLPTM1L | |  |
|  |  | DHX36 |  |  |
|  |  | COPS6 |  |  |
|  |  | LRRD1 |  |  |
|  |  | SND1 |  |  |
|  |  | PKHD1 |  |  |
|  |  | TBL3 |  |  |
|  |  | ZFR2 |  |  |
|  |  | POMGNT2 | |  |
|  |  | FAM229B | |  |
|  |  | ZFP781 |  |  |
|  |  | DSC1 |  |  |
|  |  | RAB23 |  |  |
|  |  | SLC35B1 |  |  |
|  |  | MRPL28 |  |  |
|  |  | 1700019D03RIK | |  |
|  |  | DUSP7 |  |  |
|  |  | 1700012B09RIK | |  |
|  |  | WDR19 |  |  |
|  |  | VMN1R32 | |  |
|  |  | CRKL |  |  |
|  |  | RPS27 |  |  |
|  |  | DDX10 |  |  |
|  |  | UPP1 |  |  |
|  |  | PGLS |  |  |
|  |  | GTF2H3 |  |  |
|  |  | NEMP1 |  |  |
|  |  | ZFP276 |  |  |
|  |  | ASB16 |  |  |
|  |  | SCAP |  |  |
|  |  | FKBP9 |  |  |
|  |  | CLEC2L |  |  |
|  |  | AGAP2 |  |  |
|  |  | UBE2D2A | |  |
|  |  | MAGEB18 | |  |
|  |  | OLFR1425 | |  |
|  |  | OLFR1437 | |  |
|  |  | AQP7 |  |  |
|  |  | GM36864 | |  |
|  |  | ZFP316 |  |  |
|  |  | KIF6 |  |  |
|  |  | STK17B |  |  |
|  |  | CARS |  |  |
|  |  | ZFP385B |  |  |
|  |  | AARS2 |  |  |
|  |  | RNF125 |  |  |
|  |  | GAS6 |  |  |
|  |  | PPIL2 |  |  |
|  |  | CYP26A1 | |  |
|  |  | USB1 |  |  |
|  |  | GTPBP6 |  |  |
|  |  | CFAP43 |  |  |
|  |  | SDF2 |  |  |
|  |  | FAS |  |  |
|  |  | ARHGEF33 | |  |
|  |  | LGR6 |  |  |
|  |  | ESPN |  |  |
|  |  | ARHGAP44 | |  |
|  |  | CCNO |  |  |
|  |  | CORO2B |  |  |
|  |  | GMFB |  |  |
|  |  | SPAG11B | |  |
|  |  | ZP1 |  |  |
|  |  | APOL11B | |  |
|  |  | VMN1R21 | |  |
|  |  | ZFP92 |  |  |
|  |  | VMN2R53 | |  |
|  |  | SOGA3 |  |  |
|  |  | SPATA5 |  |  |
|  |  | B3GALNT2 | |  |
|  |  | XRCC4 |  |  |
|  |  | FBXO15 |  |  |
|  |  | 1700011L22RIK | |  |
|  |  | SLC10A5 |  |  |
|  |  | PRDX1 |  |  |
|  |  | NMT1 |  |  |
|  |  | DDX56 |  |  |
|  |  | RBMXL1 |  |  |
|  |  | ADK |  |  |
|  |  | PTPN13 |  |  |
|  |  | CD7 |  |  |
|  |  | GM39653 | |  |
|  |  | NEK11 |  |  |
|  |  | TSSK4 |  |  |
|  |  | MAN1B1 |  |  |
|  |  | PON1 |  |  |
|  |  | MRPS14 |  |  |
|  |  | POM121 |  |  |
|  |  | PLA1A |  |  |
|  |  | KIF24 |  |  |
|  |  | CCNT2 |  |  |
|  |  | RTN3 |  |  |
|  |  | FASTKD3 | |  |
|  |  | GM10134 | |  |
|  |  | TOGARAM1 | |  |
|  |  | MRPL46 |  |  |
|  |  | MLPH |  |  |
|  |  | NAALAD2 | |  |
|  |  | GM6169 |  |  |
|  |  | BCAR1 |  |  |
|  |  | MEGF10 |  |  |
|  |  | ZBED4 |  |  |
|  |  | LIPE |  |  |
|  |  | DUS2 |  |  |
|  |  | TAF1B |  |  |
|  |  | UBE2I |  |  |
|  |  | PTPN3 |  |  |
|  |  | CHIT1 |  |  |
|  |  | GTF2IRD2 | |  |
|  |  | MANSC1 |  |  |
|  |  | WDFY4 |  |  |
|  |  | ADAR |  |  |
|  |  | SRSF7 |  |  |
|  |  | ANAPC16 | |  |
|  |  | CHIC1 |  |  |
|  |  | KDELR3 |  |  |
|  |  | SCRN3 |  |  |
|  |  | PRKX |  |  |
|  |  | SEC24C |  |  |
|  |  | DEGS2 |  |  |
|  |  | SLC25A42 | |  |
|  |  | TAS1R1 |  |  |
|  |  | UBE2F |  |  |
|  |  | GCFC2 |  |  |
|  |  | RNF112 |  |  |
|  |  | 4933405L10RIK | |  |
|  |  | LRIF1 |  |  |
|  |  | NUBP2 |  |  |
|  |  | RAB3GAP1 | |  |
|  |  | GBA2 |  |  |
|  |  | TTLL8 |  |  |
|  |  | STKLD1 |  |  |
|  |  | RBM7 |  |  |
|  |  | SGPP1 |  |  |
|  |  | COX7A2 |  |  |
|  |  | MCM3AP | |  |
|  |  | POLD2 |  |  |
|  |  | ZFP7 |  |  |
|  |  | GOLGA1 |  |  |
|  |  | MRPL11 |  |  |
|  |  | CCDC162 | |  |
|  |  | ZFP819 |  |  |
|  |  | ENY2 |  |  |
|  |  | CAPN9 |  |  |
|  |  | AMZ1 |  |  |
|  |  | CYP4F15 |  |  |
|  |  | CREBZF |  |  |
|  |  | RFX5 |  |  |
|  |  | TUBA1A |  |  |
|  |  | GM20594 | |  |
|  |  | EVI5L |  |  |
|  |  | CACYBP |  |  |
|  |  | CCDC77 |  |  |
|  |  | INCENP |  |  |
|  |  | SLCO4C1 | |  |
|  |  | NOL9 |  |  |
|  |  | ADAMTS12 | |  |
|  |  | HMGB2 |  |  |
|  |  | OLFR1535 | |  |
|  |  | RALA |  |  |
|  |  | CSRNP1 |  |  |
|  |  | CPEB3 |  |  |
|  |  | DENND2C | |  |
|  |  | RNF170 |  |  |
|  |  | CD200R4 |  |  |
|  |  | SCGB1B30 | |  |
|  |  | DNTT |  |  |
|  |  | SLC25A29 | |  |
|  |  | TRMU |  |  |
|  |  | MMRN2 |  |  |
|  |  | KLF16 |  |  |
|  |  | IFI204 |  |  |
|  |  | CALCRL |  |  |
|  |  | PREPL |  |  |
|  |  | INHBB |  |  |
|  |  | EIF1B |  |  |
|  |  | ARAP1 |  |  |
|  |  | UTP14A |  |  |
|  |  | GIPC1 |  |  |
|  |  | LIMD2 |  |  |
|  |  | GNL3L |  |  |
|  |  | TEX13A |  |  |
|  |  | UGT1A6A | |  |
|  |  | SSH3 |  |  |
|  |  | PPFIA1 |  |  |
|  |  | AFG1L |  |  |
|  |  | PIK3R5 |  |  |
|  |  | RIOK3 |  |  |
|  |  | PRPF4B |  |  |
|  |  | CFAP54 |  |  |
|  |  | LYSMD1 |  |  |
|  |  | NUP35 |  |  |
|  |  | EXOC3 |  |  |
|  |  | VDR |  |  |
|  |  | DSTYK |  |  |
|  |  | ZC3H18 |  |  |
|  |  | GM9970 |  |  |
|  |  | ACKR3 |  |  |
|  |  | RELA |  |  |
|  |  | KMT5B |  |  |
|  |  | DUSP5 |  |  |
|  |  | KBTBD7 |  |  |
|  |  | TOMT |  |  |
|  |  | ZFP947 |  |  |
|  |  | FXYD1 |  |  |
|  |  | ASRGL1 |  |  |
|  |  | TMEM126A | |  |
|  |  | KCNS2 |  |  |
|  |  | DFFB |  |  |
|  |  | AP2M1 |  |  |
|  |  | TBC1D24 | |  |
|  |  | SURF1 |  |  |
|  |  | MRPL52 |  |  |
|  |  | ITPK1 |  |  |
|  |  | MRPL50 |  |  |
|  |  | GLMP |  |  |
|  |  | GRM7 |  |  |
|  |  | HINFP |  |  |
|  |  | CYREN |  |  |
|  |  | GM10406 | |  |
|  |  | IRGC1 |  |  |
|  |  | IQCK |  |  |
|  |  | TRUB2 |  |  |
|  |  | ZKSCAN1 | |  |
|  |  | HNRNPF |  |  |
|  |  | PDCD2L |  |  |
|  |  | MAMLD1 | |  |
|  |  | CAR15 |  |  |
|  |  | ZFP523 |  |  |
|  |  | RPS3A1 |  |  |
|  |  | EEF1AKMT4 | |  |
|  |  | FAM135B | |  |
|  |  | RAMP1 |  |  |
|  |  | TRIM11 |  |  |
|  |  | ZC3H10 |  |  |
|  |  | SELPLG |  |  |
|  |  | HES5 |  |  |
|  |  | SFRP5 |  |  |
|  |  | T2 |  |  |
|  |  | FAM129C | |  |
|  |  | CSF2RB |  |  |
|  |  | SFT2D3 |  |  |
|  |  | COL6A5 |  |  |
|  |  | 1810043G02RIK | |  |
|  |  | GM17349 | |  |
|  |  | RSPO2 |  |  |
|  |  | PUF60 |  |  |
|  |  | SPRY2 |  |  |
|  |  | RAG1 |  |  |
|  |  | CDC5L |  |  |
|  |  | ARL6 |  |  |
|  |  | THEMIS2 | |  |
|  |  | ARSI |  |  |
|  |  | GM1141 |  |  |
|  |  | RAD23A |  |  |
|  |  | ZBTB10 |  |  |
|  |  | FANCL |  |  |
|  |  | VASH1 |  |  |
|  |  | ZFPL1 |  |  |
|  |  | CFAP74 |  |  |
|  |  | BRWD1 |  |  |
|  |  | CLCN7 |  |  |
|  |  | BCAT2 |  |  |
|  |  | CUBN |  |  |
|  |  | SLC35C2 |  |  |
|  |  | RBMS2 |  |  |
|  |  | HSP90AB1 | |  |
|  |  | PREP |  |  |
|  |  | TMEM110 | |  |
|  |  | HCN3 |  |  |
|  |  | LSM14B |  |  |
|  |  | AK9 |  |  |
|  |  | OLFR70 |  |  |
|  |  | FNBP1 |  |  |
|  |  | SYT15 |  |  |
|  |  | SIAH1B |  |  |
|  |  | ZFP950 |  |  |
|  |  | DNASE2A | |  |
|  |  | DKC1 |  |  |
|  |  | PFDN4 |  |  |
|  |  | AVPR1A |  |  |
|  |  | XPR1 |  |  |
|  |  | LRRC41 |  |  |
|  |  | TEFM |  |  |
|  |  | SMC1B |  |  |
|  |  | FAM47E |  |  |
|  |  | SLC38A3 |  |  |
|  |  | BACH1 |  |  |
|  |  | TMEM190 | |  |
|  |  | LRRC29 |  |  |
|  |  | PCP2 |  |  |
|  |  | ZKSCAN3 | |  |
|  |  | MRPL24 |  |  |
|  |  | PRPF40B | |  |
|  |  | OLFR543 |  |  |
|  |  | CHRNA6 |  |  |
|  |  | VPS53 |  |  |
|  |  | GM39115 | |  |
|  |  | CH25H |  |  |
|  |  | OLFR1366 | |  |
|  |  | TMEM8 |  |  |
|  |  | SEC11C |  |  |
|  |  | EEF1D |  |  |
|  |  | ERICH5 |  |  |
|  |  | LLPH |  |  |
|  |  | ZFP445 |  |  |
|  |  | GM15155 | |  |
|  |  | NCAN |  |  |
|  |  | RABEP2 |  |  |
|  |  | AMHR2 |  |  |
|  |  | NAA10 |  |  |
|  |  | CCDC58 |  |  |
|  |  | PTMA |  |  |
|  |  | SNRPN |  |  |
|  |  | LIPC |  |  |
|  |  | GM29427 | |  |
|  |  | LRRN4 |  |  |
|  |  | ESCO2 |  |  |
|  |  | FBXO25 |  |  |
|  |  | CEP97 |  |  |
|  |  | MED13 |  |  |
|  |  | FOXJ1 |  |  |
|  |  | SLC5A5 |  |  |
|  |  | S1PR4 |  |  |
|  |  | HTRA2 |  |  |
|  |  | KLF8 |  |  |
|  |  | ALG3 |  |  |
|  |  | GM32742 | |  |
|  |  | GHRHR |  |  |
|  |  | GM5662 |  |  |
|  |  | TEX19.1 |  |  |
|  |  | CYP21A1 | |  |
|  |  | PRL3C1 |  |  |
|  |  | IL22RA2 |  |  |
|  |  | GM32802 | |  |
|  |  | GM5460 |  |  |
|  |  | VMN2R4 |  |  |
|  |  | VMN2R3 |  |  |
|  |  | MEPE |  |  |
|  |  | H2-M10.3 | |  |
|  |  | OAS1E |  |  |
|  |  | OLFR136 |  |  |
|  |  | OLFR130 |  |  |
|  |  | RNF186 |  |  |
|  |  | OLFR156 |  |  |
|  |  | OLFR1012 | |  |
|  |  | 1700031F05RIK | |  |
|  |  | OLFR1175-PS | |  |
|  |  | SLCO1A6 | |  |
|  |  | GAL3ST4 | |  |
|  |  | OLFR281 |  |  |
|  |  | SERPINA1F | |  |
|  |  | ADAM25 |  |  |
|  |  | GPR142 |  |  |
|  |  | FGG |  |  |
|  |  | SPEM1 |  |  |
|  |  | GM15056 | |  |
|  |  | IL1F9 |  |  |
|  |  | GM17657 | |  |
|  |  | GM17654 | |  |
|  |  | OLFR105-PS | |  |
|  |  | IL1RN |  |  |
|  |  | VMN1R5 |  |  |
|  |  | PRM2 |  |  |
|  |  | GM6358 |  |  |
|  |  | AGXT2 |  |  |
|  |  | LRRC15 |  |  |
|  |  | AKR1D1 |  |  |
|  |  | 4932414N04RIK | |  |
|  |  | GFRA3 |  |  |
|  |  | GM8773 |  |  |
|  |  | OLFR701 |  |  |
|  |  | VEZF1 |  |  |
|  |  | KCNK16 |  |  |
|  |  | OLFR883 |  |  |
|  |  | OLFR1507 | |  |
|  |  | OLFR1509 | |  |
|  |  | HNF4A |  |  |
|  |  | TCSTV1 |  |  |
|  |  | FMR1NB |  |  |
|  |  | PRSS43 |  |  |
|  |  | GM10306 | |  |
|  |  | OLFR923 |  |  |
|  |  | OLFR974 |  |  |
|  |  | OLFR984 |  |  |
|  |  | PRSS33 |  |  |
|  |  | PRSS34 |  |  |
|  |  | PRSS39 |  |  |
|  |  | 4933403O08RIK | |  |
|  |  | VMN2R20 | |  |
|  |  | VMN2R18 | |  |
|  |  | GM10436 | |  |
|  |  | GM7075 |  |  |
|  |  | OTOP1 |  |  |
|  |  | MEOX2 |  |  |
|  |  | VMN2R76 | |  |
|  |  | VMN2R52 | |  |
|  |  | VMN1R35 | |  |
|  |  | OLFR314 |  |  |
|  |  | GM10959 | |  |
|  |  | OLFR320 |  |  |
|  |  | VMN1R56 | |  |
|  |  | SSXB3 |  |  |
|  |  | FSCN3 |  |  |
|  |  | TEX101 |  |  |
|  |  | OLFR1216 | |  |
|  |  | OLFR373 |  |  |
|  |  | GM9508 |  |  |
|  |  | CCDC178 | |  |
|  |  | TNFSF11 |  |  |
|  |  | FOXA2 |  |  |
|  |  | FOXA3 |  |  |
|  |  | FOXB1 |  |  |
|  |  | GM21789 | |  |
|  |  | GM21748 | |  |
|  |  | KLRB1 |  |  |
|  |  | OLFR483 |  |  |
|  |  | CYSRT1 |  |  |
|  |  | OLFR1136 | |  |
|  |  | OLFR1131 | |  |
|  |  | OLFR1161 | |  |
|  |  | OLFR525 |  |  |
|  |  | OLFR527 |  |  |
|  |  | OLFR568 |  |  |
|  |  | AKNAD1 |  |  |
|  |  | OLFR510 |  |  |
|  |  | 1700025C18RIK | |  |
|  |  | OLFR1404 | |  |
|  |  | OLFR1415 | |  |
|  |  | OLFR1448 | |  |
|  |  | MORC1 |  |  |
|  |  | OLFR670 |  |  |
|  |  | OLFR693 |  |  |
|  |  | OLFR681 |  |  |
|  |  | OLFR618 |  |  |
|  |  | OLFR635 |  |  |
|  |  | MSMB |  |  |
|  |  | OLFR1314 | |  |
|  |  | SLC22A26 | |  |
|  |  | SLC22A14 | |  |
|  |  | GM9195 |  |  |
|  |  | CHRNA9 |  |  |
|  |  | U90926 |  |  |
|  |  | ESP23 |  |  |
|  |  | MUC4 |  |  |
|  |  | MS4A18 |  |  |
|  |  | CES1H |  |  |
|  |  | CES1G |  |  |
|  |  | MUP3 |  |  |
|  |  | GSG1L2 |  |  |
|  |  | OLFR721-PS1 | |  |
|  |  | GCM2 |  |  |
|  |  | CYCT |  |  |
|  |  | 4930435E12RIK | |  |
|  |  | PNLIPRP1 | |  |
|  |  | WBP2NL |  |  |
|  |  | RDH8 |  |  |
|  |  | DMBT1 |  |  |
|  |  | TAAR4 |  |  |
|  |  | THEMIS3 | |  |
|  |  | CD200R3 |  |  |
|  |  | FBP2 |  |  |
|  |  | CRYGB |  |  |
|  |  | OCSTAMP | |  |
|  |  | TNFSF4 |  |  |
|  |  | MYL3 |  |  |
|  |  | LY6C2 |  |  |
|  |  | PLA2G2A | |  |
|  |  | LATS1 |  |  |
|  |  | GATA5 |  |  |
|  |  | GATA4 |  |  |
|  |  | VMN1R193 | |  |
|  |  | CNPY1 |  |  |
|  |  | CRISP1 |  |  |
|  |  | GM12569 | |  |
|  |  | HOXA5 |  |  |
|  |  | EDDM3B | |  |
|  |  | ECI3 |  |  |
|  |  | 8030411F24RIK | |  |
|  |  | PCK1 |  |  |
|  |  | GM438 |  |  |
|  |  | OLFR30 |  |  |
|  |  | SHISAL2A | |  |
|  |  | VMN1R218 | |  |
|  |  | VMN1R225 | |  |
|  |  | GM498 |  |  |
|  |  | VMN1R208 | |  |
|  |  | VMN1R213 | |  |
|  |  | OLFR6 |  |  |
|  |  | HOXC9 |  |  |
|  |  | HOXD4 |  |  |
|  |  | NPY6R |  |  |
|  |  | GM14214 | |  |
|  |  | AIPL1 |  |  |
|  |  | RLN3 |  |  |
|  |  | SLC5A9 |  |  |
|  |  | KRT31 |  |  |
|  |  | OLIG3 |  |  |
|  |  | GM16506 | |  |
|  |  | TEX44 |  |  |
|  |  | TEX28 |  |  |
|  |  | NLRP1A |  |  |
|  |  | P2RY10 |  |  |
|  |  | GM4841 |  |  |
|  |  | UGT2B34 | |  |
|  |  | GM853 |  |  |
|  |  | MRGPRA4 | |  |
|  |  | TEX13C3 | |  |
|  |  | FBXW24 |  |  |
|  |  | GM4788 |  |  |
|  |  | BCL2A1B | |  |
|  |  | UGT2B5 |  |  |
|  |  | CD69 |  |  |
|  |  | GM13272 | |  |
|  |  | TRIM42 |  |  |
|  |  | UNC93A |  |  |
|  |  | GM26620 | |  |
|  |  | CYP2A12 | |  |
|  |  | ZDHHC19 | |  |
|  |  | KRTAP1-3 | |  |
|  |  | GM26661 | |  |
|  |  | GM39566 | |  |
|  |  | CYP4F39 |  |  |
|  |  | GM13030 | |  |
|  |  | 2010005H15RIK | |  |
|  |  | GM3115 |  |  |
|  |  | CCNB1IP1 | |  |
|  |  | OBP2A |  |  |
|  |  | GM5724 |  |  |
|  |  | NEPN |  |  |
|  |  | GM3095 |  |  |
|  |  | GORASP1 | |  |
|  |  | CLN5 |  |  |
|  |  | HIST1H4M | |  |
|  |  | LTA4H |  |  |
|  |  | WWP1 |  |  |
|  |  | DPF1 |  |  |
|  |  | CTNNA3 |  |  |
|  |  | MXRA8 |  |  |
|  |  | PRPF3 |  |  |
|  |  | SH3TC2 |  |  |
|  |  | FUT4 |  |  |
|  |  | PNMA5 |  |  |
|  |  | NFXL1 |  |  |
|  |  | RBFA |  |  |
|  |  | MRPS21 |  |  |
|  |  | SPAG6L |  |  |
|  |  | SLC38A5 |  |  |
|  |  | IGBP1 |  |  |
|  |  | NGP |  |  |
|  |  | LRCH4 |  |  |
|  |  | TNIP3 |  |  |
|  |  | UGT1A5 |  |  |
|  |  | GM5878 |  |  |
|  |  | NAPSA |  |  |
|  |  | ARID5B |  |  |
|  |  | AXDND1 |  |  |
|  |  | CYP2J9 |  |  |
|  |  | NUS1 |  |  |
|  |  | ETAA1 |  |  |
|  |  | GSTT1 |  |  |
|  |  | CRYZL2 |  |  |
|  |  | IMP3 |  |  |
|  |  | SH3BGRL | |  |
|  |  | WDR27 |  |  |
|  |  | SLC35E2 |  |  |
|  |  | TMEM156 | |  |
|  |  | DEUP1 |  |  |
|  |  | GOSR1 |  |  |
|  |  | TACR1 |  |  |
|  |  | ART2B |  |  |
|  |  | GM5239 |  |  |
|  |  | GM6020 |  |  |
|  |  | ICOS |  |  |
|  |  | OLFR668 |  |  |
|  |  | TMPRSS15 | |  |
|  |  | GPRC6A |  |  |
|  |  | WFDC5 |  |  |
|  |  | LTB4R2 |  |  |
|  |  | KDM5B |  |  |
|  |  | PAPD4 |  |  |
|  |  | LMTK3 |  |  |
|  |  | CBX7 |  |  |
|  |  | PSMB7 |  |  |
|  |  | LZTFL1 |  |  |
|  |  | RCOR2 |  |  |
|  |  | MAP1LC3B | |  |
|  |  | SYCE1L |  |  |
|  |  | GM1673 |  |  |
|  |  | DUS1L |  |  |
|  |  | AI837181 |  |  |
|  |  | KLHDC7A | |  |
|  |  | MYO1A |  |  |
|  |  | EMD |  |  |
|  |  | TMEM129 | |  |
|  |  | CLN8 |  |  |
|  |  | ADIPOR1 | |  |
|  |  | SYNRG |  |  |
|  |  | DBF4 |  |  |
|  |  | HEATR9 |  |  |
|  |  | ZFP3 |  |  |
|  |  | IKBKAP |  |  |
|  |  | RPS4X |  |  |
|  |  | MUS81 |  |  |
|  |  | MYO19 |  |  |
|  |  | INHBE |  |  |
|  |  | FAM98C |  |  |
|  |  | SERPINB1C | |  |
|  |  | KIF18A |  |  |
|  |  | ISLR |  |  |
|  |  | RPL5 |  |  |
|  |  | GPR139 |  |  |
|  |  | INPP5D |  |  |
|  |  | CYP11A1 | |  |
|  |  | CDK1 |  |  |
|  |  | ARMH4 |  |  |
|  |  | FBXL15 |  |  |
|  |  | SLC25A36 | |  |
|  |  | OLFR777 |  |  |
|  |  | BIRC2 |  |  |
|  |  | PUM1 |  |  |
|  |  | SENP2 |  |  |
|  |  | PBP2 |  |  |
|  |  | RSL1D1 |  |  |
|  |  | GM14685 | |  |
|  |  | CNKSR3 |  |  |
|  |  | PRDM10 |  |  |
|  |  | TMEM109 | |  |
|  |  | WWTR1 |  |  |
|  |  | SERPINC1 | |  |
|  |  | 1300017J02RIK | |  |
|  |  | NAT2 |  |  |
|  |  | CES1D |  |  |
|  |  | SCARB1 |  |  |
|  |  | PLEKHH3 | |  |
|  |  | WDR18 |  |  |
|  |  | CD83 |  |  |
|  |  | ADAM32 |  |  |
|  |  | MORN2 |  |  |
|  |  | MMP25 |  |  |
|  |  | MTCH2 |  |  |
|  |  | ST13 |  |  |
|  |  | ALDH18A1 | |  |
|  |  | RMDN2 |  |  |
|  |  | RNF180 |  |  |
|  |  | F3 |  |  |
|  |  | ARMCX6 | |  |
|  |  | GPR84 |  |  |
|  |  | SEC61B |  |  |
|  |  | UTP3 |  |  |
|  |  | CBFA2T3 | |  |
|  |  | MB |  |  |
|  |  | BORCS8 |  |  |
|  |  | IBTK |  |  |
|  |  | CDH4 |  |  |
|  |  | FAM214B | |  |
|  |  | SLC32A1 |  |  |
|  |  | RFTN2 |  |  |
|  |  | TLNRD1 |  |  |
|  |  | D930048N14RIK | |  |
|  |  | OLFR1424 | |  |
|  |  | 2700062C07RIK | |  |
|  |  | WRN |  |  |
|  |  | SNAP29 |  |  |
|  |  | HIKESHI |  |  |
|  |  | RNF128 |  |  |
|  |  | MICALL2 | |  |
|  |  | GM10097 | |  |
|  |  | RPS10 |  |  |
|  |  | COPS2 |  |  |
|  |  | HMGN1 |  |  |
|  |  | ASAP1 |  |  |
|  |  | FOXG1 |  |  |
|  |  | CCR1 |  |  |
|  |  | NCOA7 |  |  |
|  |  | ZSCAN20 | |  |
|  |  | CTC1 |  |  |
|  |  | CREB1 |  |  |
|  |  | C1QTNF7 | |  |
|  |  | GTF2H2 |  |  |
|  |  | CAPN7 |  |  |
|  |  | GM6665 |  |  |
|  |  | IL1A |  |  |
|  |  | JAK1 |  |  |
|  |  | ANO8 |  |  |
|  |  | SPP2 |  |  |
|  |  | EME2 |  |  |
|  |  | PI16 |  |  |
|  |  | HMGCLL1 | |  |
|  |  | CHMP5 |  |  |
|  |  | FSD1 |  |  |
|  |  | PNPLA6 |  |  |
|  |  | HSPB3 |  |  |
|  |  | BHLHB9 |  |  |
|  |  | TYK2 |  |  |
|  |  | ZFP493 |  |  |
|  |  | ARMH3 |  |  |
|  |  | AHDC1 |  |  |
|  |  | LEXM |  |  |
|  |  | TCAF3 |  |  |
|  |  | GPR26 |  |  |
|  |  | TOMM22 | |  |
|  |  | 1110051M20RIK | |  |
|  |  | TTPA |  |  |
|  |  | UQCR11 |  |  |
|  |  | RTBDN |  |  |
|  |  | VMN2R112 | |  |
|  |  | ACAP1 |  |  |
|  |  | RNASEH2A | |  |
|  |  | NR2F1 |  |  |
|  |  | YJU2 |  |  |
|  |  | TCP10A |  |  |
|  |  | ERO1L |  |  |
|  |  | RSPH3A |  |  |
|  |  | VDAC3 |  |  |
|  |  | CYP26C1 | |  |
|  |  | COL26A1 | |  |
|  |  | NOD2 |  |  |
|  |  | ZFP446 |  |  |
|  |  | ISY1 |  |  |
|  |  | CCDC106 | |  |
|  |  | OCLN |  |  |
|  |  | BTAF1 |  |  |
|  |  | BEX4 |  |  |
|  |  | SVOPL |  |  |
|  |  | MYBPC1 |  |  |
|  |  | ZFYVE1 |  |  |
|  |  | MAN1A2 |  |  |
|  |  | RNASE13 | |  |
|  |  | 9430038I01RIK | |  |
|  |  | PLOD1 |  |  |
|  |  | GNL1 |  |  |
|  |  | ARL15 |  |  |
|  |  | ATF6B |  |  |
|  |  | TUT4 |  |  |
|  |  | PLP2 |  |  |
|  |  | WDR47 |  |  |
|  |  | LSM12 |  |  |
|  |  | TMEM198B | |  |
|  |  | NTF5 |  |  |
|  |  | STAG3 |  |  |
|  |  | SCHIP1 |  |  |
|  |  | GM20518 | |  |
|  |  | GP5 |  |  |
|  |  | HSD11B2 | |  |
|  |  | TMEM161A | |  |
|  |  | PRKG1 |  |  |
|  |  | CCNC |  |  |
|  |  | CSDC2 |  |  |
|  |  | MRS2 |  |  |
|  |  | SENP6 |  |  |
|  |  | HPSE2 |  |  |
|  |  | CCSER1 |  |  |
|  |  | CTSA |  |  |
|  |  | DPYSL2 |  |  |
|  |  | RAB3B |  |  |
|  |  | PTDSS2 |  |  |
|  |  | CNTN6 |  |  |
|  |  | TMEM239 | |  |
|  |  | CRYAB |  |  |
|  |  | GPATCH3 | |  |
|  |  | SCRIB |  |  |
|  |  | PIRB |  |  |
|  |  | ZFP141 |  |  |
|  |  | TJP3 |  |  |
|  |  | UBE2J2 |  |  |
|  |  | TCTEX1D4 | |  |
|  |  | RENBP |  |  |
|  |  | RAB39 |  |  |
|  |  | TRPC4AP | |  |
|  |  | PTHLH |  |  |
|  |  | CUTA |  |  |
|  |  | API5 |  |  |
|  |  | NDUFA9 |  |  |
|  |  | MAP3K9 |  |  |
|  |  | FAM174A | |  |
|  |  | MTMR3 |  |  |
|  |  | HR |  |  |
|  |  | CFTR |  |  |
|  |  | NINJ1 |  |  |
|  |  | PSIP1 |  |  |
|  |  | CYP2T4 |  |  |
|  |  | ACTL6A |  |  |
|  |  | D130052B06RIK | |  |
|  |  | OLFR1420 | |  |
|  |  | B4GALNT2 | |  |
|  |  | ALDOA |  |  |
|  |  | LCA5L |  |  |
|  |  | UBR3 |  |  |
|  |  | APIP |  |  |
|  |  | GNA12 |  |  |
|  |  | EFHC1 |  |  |
|  |  | DNAIC1 |  |  |
|  |  | CCNK |  |  |
|  |  | PAPD5 |  |  |
|  |  | ZP2 |  |  |
|  |  | SLURP1 |  |  |
|  |  | BTN2A2 |  |  |
|  |  | MLXIPL |  |  |
|  |  | TEX47 |  |  |
|  |  | TMEM91 |  |  |
|  |  | ATOX1 |  |  |
|  |  | GHRH |  |  |
|  |  | MALRD1 |  |  |
|  |  | TYW3 |  |  |
|  |  | HADHA |  |  |
|  |  | GM4131 |  |  |
|  |  | F13B |  |  |
|  |  | OSR2 |  |  |
|  |  | RPL27-PS3 | |  |
|  |  | GCNT3 |  |  |
|  |  | PGC |  |  |
|  |  | HCRT |  |  |
|  |  | CABS1 |  |  |
|  |  | TEX16 |  |  |
|  |  | PDZD7 |  |  |
|  |  | ARFGEF2 | |  |
|  |  | ACMSD |  |  |
|  |  | SP1 |  |  |
|  |  | IKZF1 |  |  |
|  |  | LEPROT |  |  |
|  |  | HMGXB4 | |  |
|  |  | OBSL1 |  |  |
|  |  | CFAP100 |  |  |
|  |  | PSTK |  |  |
|  |  | ZFP846 |  |  |
|  |  | THEG |  |  |
|  |  | CHKB |  |  |
|  |  | GM17662 | |  |
|  |  | ERV3 |  |  |
|  |  | GM9639 |  |  |
|  |  | IL22 |  |  |
|  |  | OLFR90 |  |  |
|  |  | RASEF |  |  |
|  |  | EDN2 |  |  |
|  |  | ERICH4 |  |  |
|  |  | HSH2D |  |  |
|  |  | CMTM2A | |  |
|  |  | NDUFA3 |  |  |
|  |  | GPR108 |  |  |
|  |  | TMEM60 |  |  |
|  |  | SLC34A1 |  |  |
|  |  | TNFSF8 |  |  |
|  |  | TRIM38 |  |  |
|  |  | NFATC3 |  |  |
|  |  | HIVEP2 |  |  |
|  |  | BBIP1 |  |  |
|  |  | AKIRIN1 |  |  |
|  |  | BLOC1S3 | |  |
|  |  | TMEM249 | |  |
|  |  | GM20696 | |  |
|  |  | PRCP |  |  |
|  |  | PGLYRP1 | |  |
|  |  | LINGO3 |  |  |
|  |  | ZRSR1 |  |  |
|  |  | QSOX2 |  |  |
|  |  | ST3GAL4 | |  |
|  |  | GDPD3 |  |  |
|  |  | SACS |  |  |
|  |  | FGF2 |  |  |
|  |  | GM1604A | |  |
|  |  | AC144408.3 | |  |
|  |  | PEG12 |  |  |
|  |  | OPRM1 |  |  |
|  |  | CHMP1B |  |  |
|  |  | UTP14B |  |  |
|  |  | MPDU1 |  |  |
|  |  | STX3 |  |  |
|  |  | CREM |  |  |
|  |  | 1700028P14RIK | |  |
|  |  | GID4 |  |  |
|  |  | SIRT7 |  |  |
|  |  | TRIM47 |  |  |
|  |  | PRPF38A | |  |
|  |  | 1600014C10RIK | |  |
|  |  | GNAO1 |  |  |
|  |  | LDHAL6B | |  |
|  |  | SLC23A1 |  |  |
|  |  | 2010111I01RIK | |  |
|  |  | GM3164 |  |  |
|  |  | KLHL20 |  |  |
|  |  | LIN7B |  |  |
|  |  | TMSB15L | |  |
|  |  | CD164 |  |  |
|  |  | USP9X |  |  |
|  |  | ARL9 |  |  |
|  |  | ATP5J |  |  |
|  |  | TIFAB |  |  |
|  |  | ACAD8 |  |  |
|  |  | GAS2L1 |  |  |
|  |  | HAUS5 |  |  |
|  |  | ZFP882 |  |  |
|  |  | CACNA2D3 | |  |
|  |  | SLC35F3 |  |  |
|  |  | ZMYM6 |  |  |
|  |  | HSPA1A |  |  |
|  |  | GM45785 | |  |
|  |  | E2F3 |  |  |
|  |  | ASAP3 |  |  |
|  |  | WDR78 |  |  |
|  |  | LAX1 |  |  |
|  |  | UQCR10 |  |  |
|  |  | GM4707 |  |  |
|  |  | ABHD13 |  |  |
|  |  | GLB1L2 |  |  |
|  |  | SLC2A3 |  |  |
|  |  | TMC4 |  |  |
|  |  | WFIKKN2 | |  |
|  |  | PHF6 |  |  |
|  |  | RIN3 |  |  |
|  |  | MYDGF |  |  |
|  |  | TRPV2 |  |  |
|  |  | STAG1 |  |  |
|  |  | ABHD6 |  |  |
|  |  | TLE6 |  |  |
|  |  | REC114 |  |  |
|  |  | PIBF1 |  |  |
|  |  | OXR1 |  |  |
|  |  | ORC2 |  |  |
|  |  | OLFML1 |  |  |
|  |  | PRMT5 |  |  |
|  |  | PIANP |  |  |
|  |  | CDC40 |  |  |
|  |  | TRIM43A | |  |
|  |  | UBAC2 |  |  |
|  |  | VPS25 |  |  |
|  |  | OLFR898 |  |  |
|  |  | OLFR688 |  |  |
|  |  | 1700001J03RIK | |  |
|  |  | GPAT4 |  |  |
|  |  | NR1H2 |  |  |
|  |  | TSPYL4 |  |  |
|  |  | SGPP2 |  |  |
|  |  | BFSP2 |  |  |
|  |  | SLC25A30 | |  |
|  |  | ZFYVE19 | |  |
|  |  | EIF5A2 |  |  |
|  |  | CDIPTOS | |  |
|  |  | TMEM237 | |  |
|  |  | IFI27 |  |  |
|  |  | PARP12 |  |  |
|  |  | PIGK |  |  |
|  |  | UBE2K |  |  |
|  |  | TAF5L |  |  |
|  |  | DAZAP1 |  |  |
|  |  | 1810024B03RIK | |  |
|  |  | EXOC4 |  |  |
|  |  | TTC7B |  |  |
|  |  | HNRNPLL | |  |
|  |  | ZFP975 |  |  |
|  |  | CCR9 |  |  |
|  |  | ZFP442 |  |  |
|  |  | DUSP22 |  |  |
|  |  | YWHAE |  |  |
|  |  | SUPT5 |  |  |
|  |  | PINLYP |  |  |
|  |  | EGFLAM |  |  |
|  |  | FAM83C |  |  |
|  |  | PHLDA1 |  |  |
|  |  | PTP4A3 |  |  |
|  |  | TBX1 |  |  |
|  |  | PAQR4 |  |  |
|  |  | RABL2 |  |  |
|  |  | TMEM17 |  |  |
|  |  | UQCRQ |  |  |
|  |  | TTC32 |  |  |
|  |  | 4921539E11RIK | |  |
|  |  | SULT6B1 | |  |
|  |  | NGRN |  |  |
|  |  | RRAGA |  |  |
|  |  | TLR13 |  |  |
|  |  | NUCB2 |  |  |
|  |  | GM10260 | |  |
|  |  | MKRN3 |  |  |
|  |  | BTBD1 |  |  |
|  |  | AW209491 | |  |
|  |  | TMEM176B | |  |
|  |  | MFAP3L |  |  |
|  |  | UMPS |  |  |
|  |  | ELOVL4 |  |  |
|  |  | FGD1 |  |  |
|  |  | LIFR |  |  |
|  |  | TRHDE |  |  |
|  |  | COG6 |  |  |
|  |  | AKT1S1 |  |  |
|  |  | DLGAP2 |  |  |
|  |  | TECR |  |  |
|  |  | PHC3 |  |  |
|  |  | KPNA6 |  |  |
|  |  | ARFGEF3 | |  |
|  |  | ZFP931 |  |  |
|  |  | EIF4G1 |  |  |
|  |  | AGER |  |  |
|  |  | ZFYVE27 | |  |
|  |  | ARHGDIB | |  |
|  |  | CISD3 |  |  |
|  |  | KCNA6 |  |  |
|  |  | WDR45B |  |  |
|  |  | TULP1 |  |  |
|  |  | SLC38A8 |  |  |
|  |  | EGFL6 |  |  |
|  |  | RYR1 |  |  |
|  |  | RPL29 |  |  |
|  |  | CSTF1 |  |  |
|  |  | CAMK1D | |  |
|  |  | CISD1 |  |  |
|  |  | SAFB2 |  |  |
|  |  | ABCC3 |  |  |
|  |  | ANKRD11 | |  |
|  |  | COPS7B |  |  |
|  |  | TIMP1 |  |  |
|  |  | SKI |  |  |
|  |  | PRAP1 |  |  |
|  |  | TPM2 |  |  |
|  |  | ATP1A4 |  |  |
|  |  | TUT7 |  |  |
|  |  | RBBP8 |  |  |
|  |  | TEX13C2 | |  |
|  |  | LGALSL |  |  |
|  |  | FBXL22 |  |  |
|  |  | SMIM1 |  |  |
|  |  | PKN3 |  |  |
|  |  | RANBP9 |  |  |
|  |  | MEA1 |  |  |
|  |  | TK1 |  |  |
|  |  | XKR5 |  |  |
|  |  | MFSD14B | |  |
|  |  | APOE |  |  |
|  |  | ZFP691 |  |  |
|  |  | CPEB1 |  |  |
|  |  | TEX30 |  |  |
|  |  | DMAP1 |  |  |
|  |  | C330007P06RIK | |  |
|  |  | CPTP |  |  |
|  |  | PSMA2 |  |  |
|  |  | A330070K13RIK | |  |
|  |  | STOML2 |  |  |
|  |  | AATF |  |  |
|  |  | IL17B |  |  |
|  |  | CCT8 |  |  |
|  |  | CIP2A |  |  |
|  |  | TMEM174 | |  |
|  |  | GM17509 | |  |
|  |  | GM10226 | |  |
|  |  | POLR2L |  |  |
|  |  | CSPG5 |  |  |
|  |  | IL23R |  |  |
|  |  | HTR3B |  |  |
|  |  | G6PC |  |  |
|  |  | ACOD1 |  |  |
|  |  | M1AP |  |  |
|  |  | CATSPER4 | |  |
|  |  | B930094E09RIK | |  |
|  |  | STXBP5L | |  |
|  |  | SVIP |  |  |
|  |  | 1700022I11RIK | |  |
|  |  | SCN11A |  |  |
|  |  | 1810032O08RIK | |  |
|  |  | NUPR1 |  |  |
|  |  | RABIF |  |  |
|  |  | TXNDC17 | |  |
|  |  | IFT140 |  |  |
|  |  | PRPF19 |  |  |
|  |  | SMPD4 |  |  |
|  |  | IQCE |  |  |
|  |  | LIMD1 |  |  |
|  |  | UGT1A7C | |  |
|  |  | 2410004B18RIK | |  |
|  |  | ACLY |  |  |
|  |  | ADAMTSL4 | |  |
|  |  | ZC3H15 |  |  |
|  |  | POLR1B |  |  |
|  |  | CMC4 |  |  |
|  |  | L3MBTL4 | |  |
|  |  | DIRC2 |  |  |
|  |  | CAMKV |  |  |
|  |  | RDX |  |  |
|  |  | HSD3B2 |  |  |
|  |  | OMA1 |  |  |
|  |  | CTGF |  |  |
|  |  | IL17RB |  |  |
|  |  | CD82 |  |  |
|  |  | PEX5L |  |  |
|  |  | ZSCAN2 |  |  |
|  |  | RBM6 |  |  |
|  |  | STIL |  |  |
|  |  | PHACTR4 | |  |
|  |  | SCNN1A |  |  |
|  |  | D230025D16RIK | |  |
|  |  | SLC19A3 |  |  |
|  |  | ACOX2 |  |  |
|  |  | NUPL2 |  |  |
|  |  | RILPL2 |  |  |
|  |  | RNF103 |  |  |
|  |  | 4930544D05RIK | |  |
|  |  | TBCC |  |  |
|  |  | BOK |  |  |
|  |  | MAP7D2 |  |  |
|  |  | FNDC11 |  |  |
|  |  | CCPG1 |  |  |
|  |  | ARFIP2 |  |  |
|  |  | TENT5B |  |  |
|  |  | 1700030J22RIK | |  |
|  |  | ARAP3 |  |  |
|  |  | FCRLB |  |  |
|  |  | CTSK |  |  |
|  |  | H2-K1 |  |  |
|  |  | CFAP57 |  |  |
|  |  | RAB2A |  |  |
|  |  | PARD6A |  |  |
|  |  | SEC23B |  |  |
|  |  | NT5C1B |  |  |
|  |  | ITGB2L |  |  |
|  |  | MPND |  |  |
|  |  | RWDD3 |  |  |
|  |  | RETREG3 | |  |
|  |  | CSTB |  |  |
|  |  | SLX4 |  |  |
|  |  | LPO |  |  |
|  |  | PLB1 |  |  |
|  |  | CC2D1A |  |  |
|  |  | TGFBR1 |  |  |
|  |  | LRRC58 |  |  |
|  |  | PARVA |  |  |
|  |  | DNAJC9 |  |  |
|  |  | RPA1 |  |  |
|  |  | TRPM8 |  |  |
|  |  | FNDC7 |  |  |
|  |  | PSME1 |  |  |
|  |  | ZFP759 |  |  |
|  |  | POC1A |  |  |
|  |  | SQOR |  |  |
|  |  | GRINA |  |  |
|  |  | NRN1L |  |  |
|  |  | USP29 |  |  |
|  |  | THUMPD3 | |  |
|  |  | TPR |  |  |
|  |  | TICAM2 |  |  |
|  |  | LRRC24 |  |  |
|  |  | PXMP2 |  |  |
|  |  | BICD2 |  |  |
|  |  | GM10604 | |  |
|  |  | ATP6V0A4 | |  |
|  |  | PITPNA |  |  |
|  |  | GBP11 |  |  |
|  |  | ZFP568 |  |  |
|  |  | GM9844 |  |  |
|  |  | XK |  |  |
|  |  | RHOT2 |  |  |
|  |  | HSD11B1 | |  |
|  |  | ARRDC1 |  |  |
|  |  | RIPOR1 |  |  |
|  |  | EBP |  |  |
|  |  | DNAJC24 | |  |
|  |  | E130311K13RIK | |  |
|  |  | TNFAIP2 |  |  |
|  |  | PLAU |  |  |
|  |  | C77080 |  |  |
|  |  | FBXO8 |  |  |
|  |  | IL5 |  |  |
|  |  | WIPF1 |  |  |
|  |  | PCID2 |  |  |
|  |  | PNLDC1 |  |  |
|  |  | GRM4 |  |  |
|  |  | CCNI |  |  |
|  |  | SCMH1 |  |  |
|  |  | HS6ST2 |  |  |
|  |  | INPP5E |  |  |
|  |  | GNG10 |  |  |
|  |  | BZW2 |  |  |
|  |  | PAWR |  |  |
|  |  | LRRC42 |  |  |
|  |  | ATXN7 |  |  |
|  |  | B3GALT2 | |  |
|  |  | FOXR2 |  |  |
|  |  | LAMTOR2 | |  |
|  |  | SRBD1 |  |  |
|  |  | MAPRE3 |  |  |
|  |  | DCUN1D1 | |  |
|  |  | SMYD4 |  |  |
|  |  | ENTPD3 |  |  |
|  |  | STRN3 |  |  |
|  |  | LRRN4CL | |  |
|  |  | WDR12 |  |  |
|  |  | TSGA10IP | |  |
|  |  | OSGEP |  |  |
|  |  | ADAT1 |  |  |
|  |  | NUP62 |  |  |
|  |  | CYSLTR1 | |  |
|  |  | SPG11 |  |  |
|  |  | KIF13B |  |  |
|  |  | GM29797 | |  |
|  |  | TXLNA |  |  |
|  |  | KCTD5 |  |  |
|  |  | SSXB5 |  |  |
|  |  | ALKBH5 |  |  |
|  |  | ARMH2 |  |  |
|  |  | BST1 |  |  |
|  |  | APLN |  |  |
|  |  | KLHL34 |  |  |
|  |  | COL5A3 |  |  |
|  |  | AGPAT3 |  |  |
|  |  | RPL35A |  |  |
|  |  | RCL1 |  |  |
|  |  | GREM1 |  |  |
|  |  | TRAM2 |  |  |
|  |  | GTF2A2 |  |  |
|  |  | GPR173 |  |  |
|  |  | SFRP4 |  |  |
|  |  | AGFG1 |  |  |
|  |  | LAP3 |  |  |
|  |  | ZBTB12 |  |  |
|  |  | TTC26 |  |  |
|  |  | GNL3 |  |  |
|  |  | TPM3 |  |  |
|  |  | SGF29 |  |  |
|  |  | LRRC75B | |  |
|  |  | OAT |  |  |
|  |  | WDR20 |  |  |
|  |  | SLC35B3 |  |  |
|  |  | TST |  |  |
|  |  | LYST |  |  |
|  |  | PPIA |  |  |
|  |  | OLFR690 |  |  |
|  |  | MTA1 |  |  |
|  |  | MOCS1 |  |  |
|  |  | CCNE2 |  |  |
|  |  | TUBG1 |  |  |
|  |  | INTS8 |  |  |
|  |  | PRELID2 |  |  |
|  |  | CRB3 |  |  |
|  |  | PRMT8 |  |  |
|  |  | SUMO1 |  |  |
|  |  | OXA1L |  |  |
|  |  | CPB1 |  |  |
|  |  | DNAL4 |  |  |
|  |  | TXNDC12 | |  |
|  |  | ZFP105 |  |  |
|  |  | BLVRA |  |  |
|  |  | SIDT2 |  |  |
|  |  | CATSPER2 | |  |
|  |  | PPP2R2A | |  |
|  |  | MFSD4B1 | |  |
|  |  | NLRC3 |  |  |
|  |  | SGIP1 |  |  |
|  |  | SRF |  |  |
|  |  | DCTN2 |  |  |
|  |  | TM9SF2 |  |  |
|  |  | MBOAT4 | |  |
|  |  | IMPDH2 |  |  |
|  |  | 3425401B19RIK | |  |
|  |  | ATR |  |  |
|  |  | ZBTB24 |  |  |
|  |  | DRG2 |  |  |
|  |  | MAML3 |  |  |
|  |  | GM2004 |  |  |
|  |  | TRAPPC11 | |  |
|  |  | USP3 |  |  |
|  |  | ADGRL1 |  |  |
|  |  | SLC45A2 |  |  |
|  |  | D430042O09RIK | |  |
|  |  | EPC2 |  |  |
|  |  | VPS35 |  |  |
|  |  | PKDCC |  |  |
|  |  | MRPL43 |  |  |
|  |  | DNAJA4 |  |  |
|  |  | CYP4F14 |  |  |
|  |  | FZD10 |  |  |
|  |  | CITED4 |  |  |
|  |  | MCM7 |  |  |
|  |  | LIN9 |  |  |
|  |  | ODF2L |  |  |
|  |  | TAF1A |  |  |
|  |  | KNTC1 |  |  |
|  |  | VEPH1 |  |  |
|  |  | 4930451I11RIK | |  |
|  |  | CPT1B |  |  |
|  |  | GAREM2 |  |  |
|  |  | RBM24 |  |  |
|  |  | SPSB2 |  |  |
|  |  | PDCD1LG2 | |  |
|  |  | CCDC110 | |  |
|  |  | INTS3 |  |  |
|  |  | SRSF6 |  |  |
|  |  | HTRA4 |  |  |
|  |  | 5430419D17RIK | |  |
|  |  | CILP2 |  |  |
|  |  | DDX27 |  |  |
|  |  | TIGAR |  |  |
|  |  | MEF2A |  |  |
|  |  | EIF2B4 |  |  |
|  |  | GDI1 |  |  |
|  |  | LRRC25 |  |  |
|  |  | GM9821 |  |  |
|  |  | CLTC |  |  |
|  |  | TRO |  |  |
|  |  | FAM166A | |  |
|  |  | CCDC149 | |  |
|  |  | CAMSAP1 | |  |
|  |  | SPX |  |  |
|  |  | MRAP |  |  |
|  |  | MBD1 |  |  |
|  |  | FCGRT |  |  |
|  |  | HSD3B6 |  |  |
|  |  | PAXX |  |  |
|  |  | E2F2 |  |  |
|  |  | SMIM4 |  |  |
|  |  | PSMG4 |  |  |
|  |  | SNX21 |  |  |
|  |  | MPV17 |  |  |
|  |  | USP25 |  |  |
|  |  | ELP2 |  |  |
|  |  | CTNNB1 |  |  |
|  |  | MELK |  |  |
|  |  | HPGD |  |  |
|  |  | OLFR550 |  |  |
|  |  | SLC38A11 | |  |
|  |  | CCDC74A | |  |
|  |  | UBE2A |  |  |
|  |  | MPLKIP |  |  |
|  |  | ZFP385A |  |  |
|  |  | PRIMA1 |  |  |
|  |  | COG7 |  |  |
|  |  | ZBTB8B |  |  |
|  |  | ISCU |  |  |
|  |  | PAF1 |  |  |
|  |  | LRRC3 |  |  |
|  |  | MOSPD3 |  |  |
|  |  | SHC2 |  |  |
|  |  | PATE1 |  |  |
|  |  | PDGFB |  |  |
|  |  | LRRC43 |  |  |
|  |  | H2AFY2 |  |  |
|  |  | HDAC7 |  |  |
|  |  | OSBPL9 |  |  |
|  |  | WNT3 |  |  |
|  |  | TNNT2 |  |  |
|  |  | PTGER4 |  |  |
|  |  | ARMC9 |  |  |
|  |  | UBE2L6 |  |  |
|  |  | CITED2 |  |  |
|  |  | CKM |  |  |
|  |  | PRKCI |  |  |
|  |  | 2010109I03RIK | |  |
|  |  | TRAF3IP2 | |  |
|  |  | MDK |  |  |
|  |  | VPS52 |  |  |
|  |  | NIFK |  |  |
|  |  | ZFP853 |  |  |
|  |  | 4933430I17RIK | |  |
|  |  | F11R |  |  |
|  |  | LMO2 |  |  |
|  |  | FOPNL |  |  |
|  |  | CETN4 |  |  |
|  |  | DENND4B | |  |
|  |  | PSMC1 |  |  |
|  |  | MED19 |  |  |
|  |  | LTV1 |  |  |
|  |  | PPM1M |  |  |
|  |  | BUB3 |  |  |
|  |  | AES |  |  |
|  |  | NAP1L1 |  |  |
|  |  | CSNK1E |  |  |
|  |  | NCLN |  |  |
|  |  | GM10800 | |  |
|  |  | CARMIL2 | |  |
|  |  | ATIC |  |  |
|  |  | KHDC1B |  |  |
|  |  | WASHC4 |  |  |
|  |  | ACOT6 |  |  |
|  |  | FKBP7 |  |  |
|  |  | MOK |  |  |
|  |  | PICALM |  |  |
|  |  | SLC25A41 | |  |
|  |  | SF3A1 |  |  |
|  |  | ZFP260 |  |  |
|  |  | PEX11G |  |  |
|  |  | AOX1 |  |  |
|  |  | CHRNE |  |  |
|  |  | NMD3 |  |  |
|  |  | MEGF9 |  |  |
|  |  | LSM10 |  |  |
|  |  | LEMD3 |  |  |
|  |  | SLC16A6 |  |  |
|  |  | KLHL6 |  |  |
|  |  | TIMM17A | |  |
|  |  | GM29666 | |  |
|  |  | WDR55 |  |  |
|  |  | CSAD |  |  |
|  |  | GM7072 |  |  |
|  |  | MAP2K6 |  |  |
|  |  | ZFP964 |  |  |
|  |  | GFM1 |  |  |
|  |  | SNX8 |  |  |
|  |  | NOSIP |  |  |
|  |  | PIPOX |  |  |
|  |  | GM14443 | |  |
|  |  | BCAS3 |  |  |
|  |  | QDPR |  |  |
|  |  | TMEM242 | |  |
|  |  | MCF2 |  |  |
|  |  | FANCA |  |  |
|  |  | BCKDK |  |  |
|  |  | HSPB7 |  |  |
|  |  | LARS |  |  |
|  |  | DNAJC13 | |  |
|  |  | CD48 |  |  |
|  |  | TRAF3IP3 | |  |
|  |  | SLC40A1 |  |  |
|  |  | ANKMY2 | |  |
|  |  | MS4A6D |  |  |
|  |  | C8G |  |  |
|  |  | GM10073 | |  |
|  |  | FMN2 |  |  |
|  |  | UNG |  |  |
|  |  | ITGB4 |  |  |
|  |  | CXCL5 |  |  |
|  |  | RPL36A-PS1 | |  |
|  |  | PI4K2B |  |  |
|  |  | MGAT2 |  |  |
|  |  | CTXN3 |  |  |
|  |  | NGDN |  |  |
|  |  | GALNTL6 | |  |
|  |  | MON1A |  |  |
|  |  | GM49405 | |  |
|  |  | BCAP31 |  |  |
|  |  | TXNDC11 | |  |
|  |  | PTGER1 |  |  |
|  |  | SLC24A4 |  |  |
|  |  | ADH5 |  |  |
|  |  | INF2 |  |  |
|  |  | CRHBP |  |  |
|  |  | NR5A1 |  |  |
|  |  | CASD1 |  |  |
|  |  | ANKDD1B | |  |
|  |  | PCDHA9 |  |  |
|  |  | SLC8B1 |  |  |
|  |  | GM29735 | |  |
|  |  | CHCHD1 |  |  |
|  |  | AP3S2 |  |  |
|  |  | PWWP2A | |  |
|  |  | GM10964 | |  |
|  |  | MC5R |  |  |
|  |  | DNAJC4 |  |  |
|  |  | GM6793 |  |  |
|  |  | GPT2 |  |  |
|  |  | ERG28 |  |  |
|  |  | HEBP1 |  |  |
|  |  | MLLT10 |  |  |
|  |  | GPATCH2L | |  |
|  |  | NAIP1 |  |  |
|  |  | RPL10A |  |  |
|  |  | LDLRAP1 | |  |
|  |  | FXYD4 |  |  |
|  |  | JRK |  |  |
|  |  | TTC9 |  |  |
|  |  | SAMD4 |  |  |
|  |  | TAB1 |  |  |
|  |  | MSLNL |  |  |
|  |  | ALG12 |  |  |
|  |  | SASS6 |  |  |
|  |  | C9ORF72 | |  |
|  |  | MYL12B |  |  |
|  |  | NT5DC2 |  |  |
|  |  | MEF2D |  |  |
|  |  | CDK13 |  |  |
|  |  | FYN |  |  |
|  |  | CSNK1A1 | |  |
|  |  | APOA1 |  |  |
|  |  | COX6B2 |  |  |
|  |  | DIEXF |  |  |
|  |  | GM3055 |  |  |
|  |  | LTBP3 |  |  |
|  |  | GLIPR1 |  |  |
|  |  | KCNK5 |  |  |
|  |  | G2E3 |  |  |
|  |  | FBXO9 |  |  |
|  |  | HIST2H2AC | |  |
|  |  | ANKS6 |  |  |
|  |  | NMB |  |  |
|  |  | AFG3L1 |  |  |
|  |  | RRH |  |  |
|  |  | OTUB1 |  |  |
|  |  | OXNAD1 |  |  |
|  |  | INO80 |  |  |
|  |  | SH3BP1 |  |  |
|  |  | SPACA6 |  |  |
|  |  | SSTR1 |  |  |
|  |  | ADAMTS18 | |  |
|  |  | GM2974 |  |  |
|  |  | CENPO |  |  |
|  |  | SELENOW | |  |
|  |  | GALNS |  |  |
|  |  | CEP63 |  |  |
|  |  | PPCDC |  |  |
|  |  | AKAP17B | |  |
|  |  | LSR |  |  |
|  |  | ARID3B |  |  |
|  |  | GLUD1 |  |  |
|  |  | LDHA |  |  |
|  |  | 1700020D05RIK | |  |
|  |  | AMD1 |  |  |
|  |  | LOXL4 |  |  |
|  |  | FBXW10 |  |  |
|  |  | MGAT5B | |  |
|  |  | MLLT1 |  |  |
|  |  | GANAB |  |  |
|  |  | MED7 |  |  |
|  |  | GYPC |  |  |
|  |  | CDT1 |  |  |
|  |  | HIST1H2BE | |  |
|  |  | TRAPPC8 | |  |
|  |  | SLC18A3 |  |  |
|  |  | NPHS1 |  |  |
|  |  | TMCO5 |  |  |
|  |  | PIMREG |  |  |
|  |  | BRINP3 |  |  |
|  |  | ST6GALNAC6 | |  |
|  |  | PYGL |  |  |
|  |  | MRPL33 |  |  |
|  |  | NETO1 |  |  |
|  |  | SDF2L1 |  |  |
|  |  | SAA2 |  |  |
|  |  | ZC3H7A |  |  |
|  |  | PCMTD2 |  |  |
|  |  | LY86 |  |  |
|  |  | TDRD1 |  |  |
|  |  | ATP6V0E2 | |  |
|  |  | DHX8 |  |  |
|  |  | TROAP |  |  |
|  |  | NR1H4 |  |  |
|  |  | KHNYN |  |  |
|  |  | ZYG11B |  |  |
|  |  | EXD1 |  |  |
|  |  | INPP1 |  |  |
|  |  | AHCY |  |  |
|  |  | AZI2 |  |  |
|  |  | KANK4 |  |  |
|  |  | NDUFA10 | |  |
|  |  | ZCCHC17 | |  |
|  |  | PLA2G6 |  |  |
|  |  | ARMT1 |  |  |
|  |  | EAPP |  |  |
|  |  | SGO1 |  |  |
|  |  | DESI1 |  |  |
|  |  | NEGR1 |  |  |
|  |  | POU3F1 |  |  |
|  |  | PROB1 |  |  |
|  |  | OLA1 |  |  |
|  |  | AP4M1 |  |  |
|  |  | ECHDC2 |  |  |
|  |  | ZDHHC2 |  |  |
|  |  | GLYCAM1 | |  |
|  |  | SLC4A1AP | |  |
|  |  | GLT28D2 | |  |
|  |  | PIP4P2 |  |  |
|  |  | SEMA4D |  |  |
|  |  | SAXO2 |  |  |
|  |  | NECTIN2 | |  |
|  |  | RASGRP3 | |  |
|  |  | RPS16 |  |  |
|  |  | ABHD16A | |  |
|  |  | BCS1L |  |  |
|  |  | C1QL1 |  |  |
|  |  | UBA6 |  |  |
|  |  | THAP3 |  |  |
|  |  | NANOS3 |  |  |
|  |  | SIX3 |  |  |
|  |  | 9130008F23RIK | |  |
|  |  | VMN2R5 |  |  |
|  |  | IGLL1 |  |  |
|  |  | VMN1R30 | |  |
|  |  | 4930578I06RIK | |  |
|  |  | KLRK1 |  |  |
|  |  | OLFR1431 | |  |
|  |  | TOPAZ1 |  |  |
|  |  | TMCO2 |  |  |
|  |  | NR2E3 |  |  |
|  |  | 4921509C19RIK | |  |
|  |  | GATA3 |  |  |
|  |  | PAX1 |  |  |
|  |  | METTL7B | |  |
|  |  | CRYBA2 |  |  |
|  |  | GM3250 |  |  |
|  |  | IFIT2 |  |  |
|  |  | KCNIP2 |  |  |
|  |  | ANKLE2 |  |  |
|  |  | 1600012H06RIK | |  |
|  |  | ACO1 |  |  |
|  |  | NDUFAF6 | |  |
|  |  | MAP2K4 |  |  |
|  |  | DNAJC28 | |  |
|  |  | MAP2K7 |  |  |
|  |  | PPP2R5A | |  |
|  |  | GATAD2B | |  |
|  |  | AIF1 |  |  |
|  |  | NOP16 |  |  |
|  |  | SPTY2D1 | |  |
|  |  | B4GALNT4 | |  |
|  |  | ABCC10 |  |  |
|  |  | NNMT |  |  |
|  |  | TRMT6 |  |  |
|  |  | NKPD1 |  |  |
|  |  | NVL |  |  |
|  |  | A130010J15RIK | |  |
|  |  | BLOC1S2 | |  |
|  |  | CBARP |  |  |
|  |  | EHBP1L1 | |  |
|  |  | GGT5 |  |  |
|  |  | STT3B |  |  |
|  |  | RELCH |  |  |
|  |  | CNTFR |  |  |
|  |  | ATPAF2 |  |  |
|  |  | RAB20 |  |  |
|  |  | HFE2 |  |  |
|  |  | FMR1 |  |  |
|  |  | ELL |  |  |
|  |  | SLC5A11 |  |  |
|  |  | SF3A3 |  |  |
|  |  | EBF3 |  |  |
|  |  | GORASP2 | |  |
|  |  | FAM208A | |  |
|  |  | FXYD2 |  |  |
|  |  | CLPTM1 |  |  |
|  |  | GPR65 |  |  |
|  |  | OLFR1440 | |  |
|  |  | IL17RE |  |  |
|  |  | AVPI1 |  |  |
|  |  | NPHP3 |  |  |
|  |  | PLN |  |  |
|  |  | 4430402I18RIK | |  |
|  |  | SPG7 |  |  |
|  |  | GDF10 |  |  |
|  |  | PRKRA |  |  |
|  |  | UMODL1 | |  |
|  |  | LIX1 |  |  |
|  |  | CLEC4N |  |  |
|  |  | OPCML |  |  |
|  |  | TMEM167B | |  |
|  |  | CAND2 |  |  |
|  |  | SLC10A3 |  |  |
|  |  | KLK8 |  |  |
|  |  | OGG1 |  |  |
|  |  | NRBP1 |  |  |
|  |  | TRA2B |  |  |
|  |  | CAGE1 |  |  |
|  |  | TEDDM1A | |  |
|  |  | CEP131 |  |  |
|  |  | SLC44A2 |  |  |
|  |  | ALKBH2 |  |  |
|  |  | MAATS1 |  |  |
|  |  | ELF5 |  |  |
|  |  | PDGFA |  |  |
|  |  | FBXO16 |  |  |
|  |  | KIF1A |  |  |
|  |  | C130060K24RIK | |  |
|  |  | ZFP414 |  |  |
|  |  | SLC46A1 |  |  |
|  |  | BOP1 |  |  |
|  |  | PRSS50 |  |  |
|  |  | SDHD |  |  |
|  |  | KPNA4 |  |  |
|  |  | 9530068E07RIK | |  |
|  |  | TXNDC2 |  |  |
|  |  | ACAA1A |  |  |
|  |  | MT1 |  |  |
|  |  | SLC35A3 |  |  |
|  |  | MYOCD |  |  |
|  |  | LIX1L |  |  |
|  |  | TTC36 |  |  |
|  |  | RING1 |  |  |
|  |  | HIC2 |  |  |
|  |  | FKBP1A |  |  |
|  |  | TMEM106B | |  |
|  |  | NIPA1 |  |  |
|  |  | PSMG1 |  |  |
|  |  | FABP12 |  |  |
|  |  | CCL6 |  |  |
|  |  | KLHDC4 |  |  |
|  |  | CACFD1 |  |  |
|  |  | TMEM30C | |  |
|  |  | CNOT1 |  |  |
|  |  | NUTF2-PS1 | |  |
|  |  | RPS6KL1 | |  |
|  |  | ADAMTSL3 | |  |
|  |  | ABCA8B |  |  |
|  |  | RGS12 |  |  |
|  |  | POMGNT1 | |  |
|  |  | PCBD2 |  |  |
|  |  | GPR107 |  |  |
|  |  | S1PR1 |  |  |
|  |  | SF3A2 |  |  |
|  |  | RNF138RT1 | |  |
|  |  | MSANTD2 | |  |
|  |  | GM14327 | |  |
|  |  | FASTKD5 | |  |
|  |  | KDM2A |  |  |
|  |  | GRAP |  |  |
|  |  | LIPH |  |  |
|  |  | SMC1A |  |  |
|  |  | RBM8A2 |  |  |
|  |  | ZFP932 |  |  |
|  |  | MIEN1 |  |  |
|  |  | CCDC61 |  |  |
|  |  | USP37 |  |  |
|  |  | DDX4 |  |  |
|  |  | ZFP956 |  |  |
|  |  | ZFP280B |  |  |
|  |  | SDS |  |  |
|  |  | ZFP719 |  |  |
|  |  | HBA-A2 |  |  |
|  |  | HSD17B12 | |  |
|  |  | RAB11FIP2 | |  |
|  |  | HERC4 |  |  |
|  |  | TMOD3 |  |  |
|  |  | SMAD5 |  |  |
|  |  | FUOM |  |  |
|  |  | USP20 |  |  |
|  |  | ZBP1 |  |  |
|  |  | RPUSD3 |  |  |
|  |  | TBC1D10A | |  |
|  |  | RABGGTB | |  |
|  |  | HNRNPD | |  |
|  |  | HIRA |  |  |
|  |  | SNX10 |  |  |
|  |  | MYO3B |  |  |
|  |  | CNOT6L |  |  |
|  |  | PCDHGA8 | |  |
|  |  | RPRD1B |  |  |
|  |  | LHX6 |  |  |
|  |  | HINT2 |  |  |
|  |  | FAM209 |  |  |
|  |  | ZFP976 |  |  |
|  |  | XRCC6 |  |  |
|  |  | STAMBPL1 | |  |
|  |  | TMBIM4 |  |  |
|  |  | UBE2Z |  |  |
|  |  | ZBTB34 |  |  |
|  |  | NAB1 |  |  |
|  |  | OLFR544 |  |  |
|  |  | KCNE1L |  |  |
|  |  | SLC37A2 |  |  |
|  |  | IFNLR1 |  |  |
|  |  | MYOZ2 |  |  |
|  |  | MBTPS1 |  |  |
|  |  | PPP1R36 |  |  |
|  |  | ZFP869 |  |  |
|  |  | KHK |  |  |
|  |  | MORN4 |  |  |
|  |  | TFAP4 |  |  |
|  |  | GM21885 | |  |
|  |  | IARS |  |  |
|  |  | PCDHB14 | |  |
|  |  | RRP7A |  |  |
|  |  | NOL6 |  |  |
|  |  | RARRES2 | |  |
|  |  | AAMP |  |  |
|  |  | TMEM160 | |  |
|  |  | CSNK1G1 | |  |
|  |  | SAPCD2 |  |  |
|  |  | GAK |  |  |
|  |  | CLCA3A1 | |  |
|  |  | LRAT |  |  |
|  |  | ZFP583 |  |  |
|  |  | DUSP6 |  |  |
|  |  | EPB42 |  |  |
|  |  | HPS3 |  |  |
|  |  | MYNN |  |  |
|  |  | BC024978 | |  |
|  |  | 1700013F07RIK | |  |
|  |  | HBP1 |  |  |
|  |  | NDUFA11 | |  |
|  |  | FBL |  |  |
|  |  | CEP350 |  |  |
|  |  | RUSC2 |  |  |
|  |  | CCDC50 |  |  |
|  |  | SH3BGRL3 | |  |
|  |  | METTL21C | |  |
|  |  | FRMPD1 |  |  |
|  |  | AOC3 |  |  |
|  |  | COX6A1 |  |  |
|  |  | ERI1 |  |  |
|  |  | A530016L24RIK | |  |
|  |  | WDR82 |  |  |
|  |  | USP15 |  |  |
|  |  | CBX6 |  |  |
|  |  | PSMB4 |  |  |
|  |  | MCUR1 |  |  |
|  |  | NOD1 |  |  |
|  |  | DDX24 |  |  |
|  |  | CARNMT1 | |  |
|  |  | MANF |  |  |
|  |  | TADA1 |  |  |
|  |  | NXT2 |  |  |
|  |  | CCDC17 |  |  |
|  |  | CHD7 |  |  |
|  |  | ANKRD53 | |  |
|  |  | PXYLP1 |  |  |
|  |  | TRIM26 |  |  |
|  |  | TTC12 |  |  |
|  |  | MAPT |  |  |
|  |  | GPR137C | |  |
|  |  | ELP5 |  |  |
|  |  | MRPL58 |  |  |
|  |  | CUTAL |  |  |
|  |  | ZFP993 |  |  |
|  |  | NPL |  |  |
|  |  | 4930590J08RIK | |  |
|  |  | CLIP1 |  |  |
|  |  | EDA2R |  |  |
|  |  | MEI1 |  |  |
|  |  | COL11A2 | |  |
|  |  | GRB2 |  |  |
|  |  | SLCO4A1 | |  |
|  |  | GM35549 | |  |
|  |  | WDR83 |  |  |
|  |  | CEP78 |  |  |
|  |  | RPL27 |  |  |
|  |  | ZFAND3 |  |  |
|  |  | ITGAE |  |  |
|  |  | CEP128 |  |  |
|  |  | CCDC152 | |  |
|  |  | TRIM35 |  |  |
|  |  | 4931406B18RIK | |  |
|  |  | CCDC83 |  |  |
|  |  | MAP4K3 |  |  |
|  |  | ANKRD49 | |  |
|  |  | MAP3K20 | |  |
|  |  | SMIM11 |  |  |
|  |  | FAM160A2 | |  |
|  |  | TXLNG |  |  |
|  |  | MIF4GD |  |  |
|  |  | SLC25A51 | |  |
|  |  | THOC1 |  |  |
|  |  | SEMA6B |  |  |
|  |  | NHLRC2 |  |  |
|  |  | GM28042 | |  |
|  |  | MID1IP1 |  |  |
|  |  | PRMT6 |  |  |
|  |  | ISL1 |  |  |
|  |  | GM5640 |  |  |
|  |  | BPI |  |  |
|  |  | BSX |  |  |
|  |  | GM17428 | |  |
|  |  | OLFR877 |  |  |
|  |  | VMN2R93 | |  |
|  |  | NRK |  |  |
|  |  | OLFR1129 | |  |
|  |  | OLFR564 |  |  |
|  |  | FFAR4 |  |  |
|  |  | GM10273 | |  |
|  |  | OLFR39 |  |  |
|  |  | ZP3 |  |  |
|  |  | FGL1 |  |  |
|  |  | GAL3ST2C | |  |
|  |  | SPC25 |  |  |
|  |  | RECQL |  |  |
|  |  | DNAH6 |  |  |
|  |  | SLC6A8 |  |  |
|  |  | PXK |  |  |
|  |  | SHISA2 |  |  |
|  |  | DLG4 |  |  |
|  |  | SMAD2 |  |  |
|  |  | PHF10 |  |  |
|  |  | TRIM24 |  |  |
|  |  | CCNT1 |  |  |
|  |  | CCDC127 | |  |
|  |  | LCMT1 |  |  |
|  |  | SERGEF |  |  |
|  |  | ALKBH1 |  |  |
|  |  | SNX16 |  |  |
|  |  | TMEM45B | |  |
|  |  | SLC16A4 |  |  |
|  |  | OTUD7A |  |  |
|  |  | ST6GAL1 | |  |
|  |  | ATP8A2 |  |  |
|  |  | SREBF1 |  |  |
|  |  | GSDME |  |  |
|  |  | FAM126A | |  |
|  |  | PLEK2 |  |  |
|  |  | CWF19L1 | |  |
|  |  | CAST |  |  |
|  |  | PZP |  |  |
|  |  | TMPRSS6 | |  |
|  |  | IDH3B |  |  |
|  |  | CLNS1A |  |  |
|  |  | KIF3C |  |  |
|  |  | ALOX5 |  |  |
|  |  | MCMDC2 | |  |
|  |  | TANGO6 |  |  |
|  |  | GM30191 | |  |
|  |  | GM35315 | |  |
|  |  | KDELR1 |  |  |
|  |  | PAX6 |  |  |
|  |  | LTB4R1 |  |  |
|  |  | GM3558 |  |  |
|  |  | CD300A |  |  |
|  |  | UBC |  |  |
|  |  | TONSL |  |  |
|  |  | BEX2 |  |  |
|  |  | NTAN1 |  |  |
|  |  | ATN1 |  |  |
|  |  | SYMPK |  |  |
|  |  | MSTO1 |  |  |
|  |  | TRAFD1 |  |  |
|  |  | GNG5 |  |  |
|  |  | KLF2 |  |  |
|  |  | SUFU |  |  |
|  |  | HIST2H3B | |  |
|  |  | 4930539E08RIK | |  |
|  |  | PLD3 |  |  |
|  |  | TTC27 |  |  |
|  |  | RFESD |  |  |
|  |  | POC1B |  |  |
|  |  | AP2S1 |  |  |
|  |  | SCOC |  |  |
|  |  | FAM20C |  |  |
|  |  | EXOC3L4 | |  |
|  |  | GM12216 | |  |
|  |  | ANXA8 |  |  |
|  |  | NSUN6 |  |  |
|  |  | TMEM141 | |  |
|  |  | MTF2 |  |  |
|  |  | MS4A7 |  |  |
|  |  | NEIL2 |  |  |
|  |  | PCDHB17 | |  |
|  |  | CYP17A1 | |  |
|  |  | RETN |  |  |
|  |  | MALSU1 |  |  |
|  |  | MCTS1 |  |  |
|  |  | OAZ1 |  |  |
|  |  | FNTB |  |  |
|  |  | CHRNB2 |  |  |
|  |  | MASP2 |  |  |
|  |  | DEAF1 |  |  |
|  |  | STX17 |  |  |
|  |  | PODXL |  |  |
|  |  | SNAI1 |  |  |
|  |  | TLR4 |  |  |
|  |  | C1QTNF2 | |  |
|  |  | SPIN4 |  |  |
|  |  | ELFN2 |  |  |
|  |  | PPAT |  |  |
|  |  | CHRM1 |  |  |
|  |  | HIST1H2AC | |  |
|  |  | PDCL |  |  |
|  |  | ABHD17A | |  |
|  |  | MT-CO2 |  |  |
|  |  | SEC31A |  |  |
|  |  | TMEM170 | |  |
|  |  | CCDC68 |  |  |
|  |  | TM4SF20 | |  |
|  |  | LY6G5B |  |  |
[truncated: 66,471 more chars]
